# The Geography of Recent Genetic Ancestry across Europe

**DOI:** 10.1371/journal.pbio.1001555

**Published:** 2013-05-07

**Authors:** Peter Ralph, Graham Coop

**Affiliations:** Department of Evolution and Ecology & Center for Population Biology, University of California, Davis, California, United States of America; The Wellcome Trust Sanger Institute, United Kingdom

## Abstract

The recent genealogical history of human populations is a complex mosaic formed by individual migration, large-scale population movements, and other demographic events. Population genomics datasets can provide a window into this recent history, as rare traces of recent shared genetic ancestry are detectable due to long segments of shared genomic material. We make use of genomic data for 2,257 Europeans (in the Population Reference Sample [POPRES] dataset) to conduct one of the first surveys of recent genealogical ancestry over the past 3,000 years at a continental scale. We detected 1.9 million shared long genomic segments, and used the lengths of these to infer the distribution of shared ancestors across time and geography. We find that a pair of modern Europeans living in neighboring populations share around 2–12 genetic common ancestors from the last 1,500 years, and upwards of 100 genetic ancestors from the previous 1,000 years. These numbers drop off exponentially with geographic distance, but since these genetic ancestors are a tiny fraction of common genealogical ancestors, individuals from opposite ends of Europe are still expected to share millions of common genealogical ancestors over the last 1,000 years. There is also substantial regional variation in the number of shared genetic ancestors. For example, there are especially high numbers of common ancestors shared between many eastern populations that date roughly to the migration period (which includes the Slavic and Hunnic expansions into that region). Some of the lowest levels of common ancestry are seen in the Italian and Iberian peninsulas, which may indicate different effects of historical population expansions in these areas and/or more stably structured populations. Population genomic datasets have considerable power to uncover recent demographic history, and will allow a much fuller picture of the close genealogical kinship of individuals across the world.

## Abstract 2

A genomic survey of recent genealogical relatedness reveals the close ties of kinship and the impact of events across the past 3,000 years of European history.

## Author Summary

Few of us know our family histories more than a few generations back. It is therefore easy to overlook the fact that we are all distant cousins, related to one another via a vast network of relationships. Here we use genome-wide data from European individuals to investigate these relationships over the past 3,000 years, by looking for long stretches of genome that are shared between pairs of individuals through their inheritance from common genetic ancestors. We quantify this ubiquitous recent common ancestry, showing for instance that even pairs of individuals from opposite ends of Europe share hundreds of genetic common ancestors over this time period. Despite this degree of commonality, there are also striking regional differences. Southeastern Europeans, for example, share large numbers of common ancestors that date roughly to the era of the Slavic and Hunnic expansions around 1,500 years ago, while most common ancestors that Italians share with other populations lived longer than 2,500 years ago. The study of long stretches of shared genetic material promises to uncover rich information about many aspects of recent population history.

## Introduction

Even seemingly unrelated humans are distant cousins to each other, as all members of a species are related to each other through a vastly ramified family tree (their pedigree). We can see traces of these relationships in genetic data when individuals inherit shared genetic material from a common ancestor. Traditionally, population genetics has studied the distant bulk of these genetic relationships, which in humans typically date from hundreds of thousands of years ago (e.g., [1],[2]). Such studies have provided deep insights into the origins of modern humans (e.g., [3]), and into recent admixture between diverged populations (e.g., [4],[5]).

Although most such genetic relationships among individuals are very old, some individuals are related on far shorter time scales. Indeed, given that each individual has 2*^n^* ancestors from *n* generations ago, theoretical considerations suggest that all humans are related genealogically to each other over surprisingly short time scales [6],[7]. We are usually unaware of these close genealogical ties, as few of us have knowledge of family histories more than a few generations back, and these ancestors often do not contribute any genetic material to us [8]. However, in large samples we can hope to identify genetic evidence of more recent relatedness, and so obtain insight into the population history of the past tens of generations. Here we investigate such patterns of recent relatedness in a large European dataset.

The past several thousand years are replete with events that may have had significant impact on modern European relatedness, such as the Neolithic expansion of farming, the Roman empire, or the more recent expansions of the Slavs and the Vikings. Our current understanding of these events is deduced from archaeological, linguistic, cultural, historical, and genetic evidence, with widely varying degrees of certainty. However, the demographic and genealogical impact of these events is still uncertain (e.g., [9]). Genetic data describing the breadth of genealogical relationships can therefore add another dimension to our understanding of these historical events.

Work from uniparentally inherited markers (mtDNA and Y chromosomes) has improved our understanding of human demographic history (e.g., [10]). However, interpretation of these markers is difficult since they only record a single lineage of each individual (the maternal and paternal lineages, respectively), rather than the entire distribution of ancestors. Genome-wide genotyping and sequencing datasets have the potential to provide a much richer picture of human history, as we can learn simultaneously about the diversity of ancestors that contributed to each individual's genome.

A number of genome-wide studies have begun to reveal quantitative insights into recent human history [11]. Within Europe, the first two principal axes of variation of the matrix of genotypes are closely related to a rotation of latitude and longitude [12]–[14], as would be expected if patterns of ancestry are mostly shaped by local migration [15]. Other work has revealed a slight decrease in diversity running from south-to-north in Europe, with the highest haplotype and allelic diversity in the Iberian peninsula (e.g., [14],[16],[17]), and the lowest haplotype diversity in England and Ireland [18]. Recently, progress has also been made using genotypes of ancient individuals to understand the prehistory of Europe [19]–[21]. However, we currently have little sense of the time scale of the historical events underlying modern geographic patterns of relatedness, nor the degrees of genealogical relatedness they imply.

In this article, we analyze those rare long chunks of genome that are shared between pairs of individuals due to inheritance from recent common ancestors, to obtain a detailed view of the geographic structure of recent relatedness. To determine the time scale of these relationships, we develop methodology that uses the lengths of shared genomic segments to infer the distribution of the ages of these recent common ancestors. We find that even geographically distant Europeans share ubiquitous common ancestry within the past 1,000 years, and show that common ancestry from the past 3,000 years is a result of both local migration and large-scale historical events. We find considerable structure below the country level in sharing of recent ancestry, lending further support to the idea that looking at runs of shared ancestry can identify very subtle population structure (e.g., [22]).

Our method for inferring ages of common ancestors is conceptually similar to the work of [23], who use total amount of long runs of shared genome to fit simple parametric models of recent history, as well as to [3] and [24], who use information from short runs of shared genome to infer demographic history over much longer time scales. Other conceptually similar work includes [25] and [26], who used the length distribution of admixture tracts to fit parametric models of historical admixture. We rely less on discrete, idealized populations or parametric demographic models than these other works, and describe continuous geographic structure by obtaining average numbers of common ancestors shared by many populations across time in a relatively nonparametric fashion.

### Definitions: Genetic Ancestry and Identity by Descent

**Figure 1:**
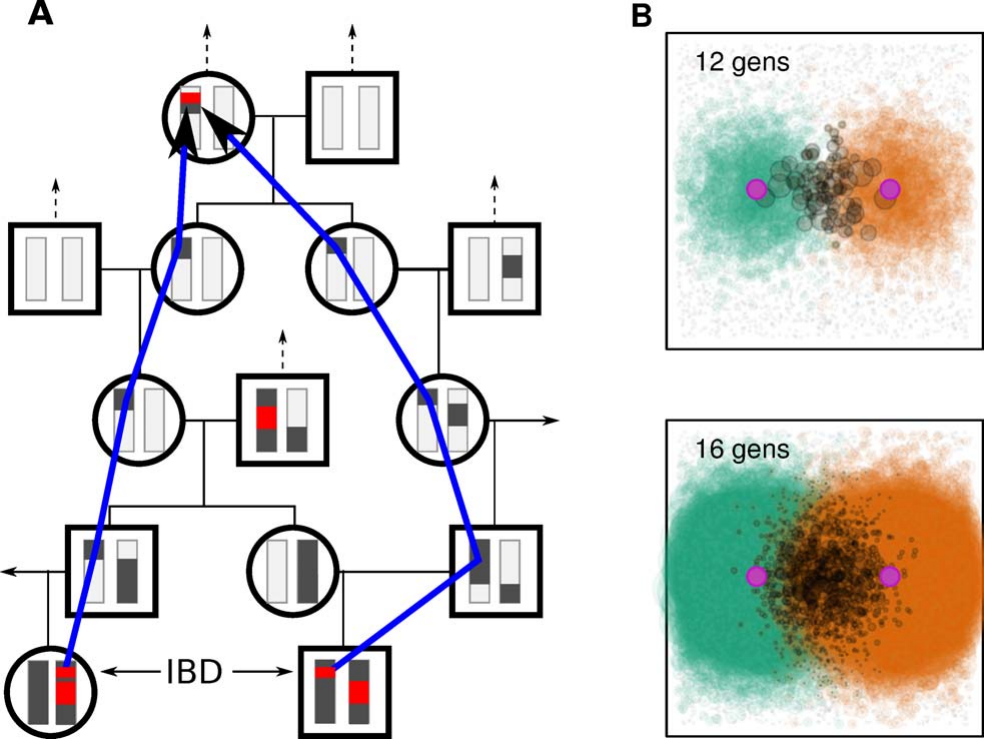
The spread of genetic ancestry. (A) A hypothetical portion of the pedigree relating two sampled individuals, which shows six of their genealogical common ancestors, with the portions of ancestral chromosomes from which the sampled individuals have inherited shaded grey. The IBD blocks they have inherited from the two genetic common ancestors are colored red, and the blue arrow denotes the path through the pedigree along which one of these IBD blocks was inherited. (B) Cartoon of the spatial locations of ancestors of two individuals—circle size is proportional to likelihood of genetic contribution, and shared ancestors are marked in grey. Note that common ancestors are likely located between the two, and their distribution becomes more diffuse further back in time.

We can only hope to learn from genetic data about those common ancestors from whom two individuals have both inherited the same genomic region. If a pair of individuals have both inherited some genomic region from a common ancestor, that ancestor is called a “genetic common ancestor,” and the genomic region is shared “identical by descent” (IBD) by the two. Here we define an “IBD block” to be a contiguous segment of genome inherited (on at least one chromosome) from a shared common ancestor without intervening recombination (see Figure 1A). A more usual definition of IBD restricts to those segments inherited from some prespecified set of “founder” individuals (e.g., [8],[27],[28]), but we allow ancestors to be arbitrarily far back in time. Under our definition, everyone is IBD everywhere, but mostly on very short, old segments [29]. We measure lengths of IBD segments in units of Morgans (M) or centiMorgans (cM), where 1 Morgan is defined to be the distance over which an average of one recombination (i.e., a crossover) occurs per meiosis. Segments of IBD are broken up over time by recombination, which implies that older shared ancestry tends to result in shorter shared IBD blocks.

Sufficiently long segments of IBD can be identified as long, contiguous regions over which the two individuals are identical (or nearly identical) at a set of single nucleotide polymorphisms (SNPs) that segregate in the population. Formal, model-based methods to infer IBD are only computationally feasible for very recent ancestry (e.g., [30]), but recently, fast heuristic algorithms have been developed that can be applied to thousands of samples typed on genotyping chips (e.g., [31],[32]).

The relationship between numbers of long, shared segments of genome, numbers of genetic common ancestors, and numbers of genealogical common ancestors can be difficult to envision. Since everyone has exactly two biological parents, every individual has exactly 2*^n^* paths of length *n* meioses leading back through their pedigree, each such path ending in a grand*^n^* ^–1^parent. However, due to Mendelian segregation and limited recombination, genetic material will only be passed down along a small subset of these paths [8]. As *n* grows, these paths proliferate rapidly and so the genealogical paths of two individuals soon overlap significantly. (These points are illustrated in Figure 1.) By observing the number of shared genomic blocks, we learn about the degree to which their genealogies overlap, or the number of common ancestors from which both individuals have inherited genetic material.

At least one parent of each genetic common ancestor of two individuals is also a genetic common ancestor, so the number of genetic common ancestors at each point back in time is strictly increasing. A more relevant quantity is the rate of appearance of *most recent* common genetic ancestors. This quantity can be much more intuitive, and is closely related to the coalescent rate [33], as we demonstrate later. For this reason, when we say “genetic common ancestor” or “rate of genetic common ancestry,” we are referring to only the *most recent* genetic common ancestors from which the individuals in question inherited their shared segments of genome.

## Results

We applied the fastIBD method, implemented in BEAGLE v3.3 [31], to the European subset of the Population Reference Sample (POPRES) dataset (dbgap accession phs000145.v1.p1, [34]), which includes language and country-of-origin data for several thousand Europeans genotyped at 500,000 SNPs. Our simulations showed that we have good power to detect long IBD blocks (probability of detection 50% for blocks longer than 2 cM, rising to 98% for blocks longer than 4 cM), and a low false positive rate (discussed further in the Materials and Methods section). We excluded from our analyses individuals who reported grandparents originating from non-European countries or more than one distinct country (and refer to the remainder as “Europeans”). After removing obvious outlier individuals and close relatives, we were left with 2,257 individuals who we grouped using reported country of origin and language into 40 populations, listed with sample sizes and average IBD levels in Table 1. For geographic analyses, we located each population at the largest population city in the appropriate region. Pairs of individuals in this dataset were found to share a total of 1.9 million segments of IBD, an average of 0.74 per pair of individuals, or 831 per individual. The mean length of these blocks was 2.5 cM, the median was 2.1 cM, and the 25th and 75th quantiles are 1.5 cM and 2.9 cM, respectively. The majority of pairs sharing some IBD shared only a single block of IBD (94%). The total length of IBD blocks an individual shares with all others ranged between 30% and 250% (average 128%) of the length of the genome (greater than 100% is possible as individuals may share IBD blocks with more than one other at the same genomic location).

**Table 1:** Populations, abbreviations, sample sizes (*n*), mean number of IBD blocks shared by a pair of individuals from that population (“self”), and mean IBD rate averaged across all other populations (“other”), sorted by regional groupings described in the text.

Group	Abbreviation	*n*	Self	Other
*E group*				
Albania	AL	9	14.5	v
Austria	AT	14	1.3	0.9
Bosnia	BO	9	4.1	1.6
Bulgaria	BG	1	—	1.3
Croatia	HR	9	2.8	1.6
Czech Republic	CZ	9	2.1	1.3
Greece	EL	5	1.8	0.9
Hungary	HU	19	1.9	1.2
Kosovo	KO	15	9.9	1.7
Montenegro	ME	1	–	1.8
Macedonia	MA	4	2.5	0.4
Poland	PL	22	3.8	1.5
Romania	RO	14	2.1	1.2
Russia	RU	6	4.3	1.4
Slovenia	SI	2	5.0	1.3
Serbia	RS	11	2.7	1.5
Slovakia	SK	1	—	0.7
Ukraine	UA	1	—	1.5
Yugoslavia	YU	10	3.4	1.5
*TC group*				
Cyprus	CY	3	2.7	0.4
Turkey	TR	4	2.2	0.5
*N group*				
Denmark	DK	1	—	0.9
Finland	FI	1	—	1.2
Latvia	LV	1	—	1.6
Norway	NO	2	2.0	0.8
Sweden	SE	10	3.4	1.0
*W group*				
Belgium	BE	37	1.1	0.6
England	EN	22	1.3	0.7
France	FR	86	0.7	0.5
Germany	DE	71	1.1	0.9
Ireland	IE	60	2.6	0.6
Netherlands	NL	17	1.9	0.7
Scotland	SC	5	2.2	0.7
Swiss French	CHf	839	1.3	0.6
Swiss German	CHd	103	1.6	0.6
Switzerland	CH	17	1.1	0.5
United Kingdom	UK	358	1.2	0.7
*I group*				
Italy	IT	213	0.6	0.5
Portugal	PT	115	1.9	0.5
Spain	ES	130	1.5	0.4

The observed genomic density of long IBD blocks (per cM) can be affected by recent selection [35] and by cis-acting recombination modifiers. We find that the local density of IBD blocks of all lengths is relatively constant across the genome, but in certain regions the length distribution is systematically perturbed (see Figure S1), including around certain centromeres and the large inversion on chromosome 8 [36], also seen by [35]. Somewhat surprisingly, the MHC does not show an unusual pattern of IBD, despite having shown up in other genomic scans for IBD [35],[37]. However, there are a few other regions where differences in IBD rate are not predicted by differences in SNP density. Notably, there are two regions, on chromosomes 15 and 16, which are nearly as extreme in their deviations in IBD as the inversion on chromosome 8, and may also correspond to large inversions segregating in the sample. These only make up a small portion of the genome, and do not significantly affect our other analyses (and so are not removed); we leave further analysis for future work.

### Substructure and Recent Migrants

We should expect significant within-population variability, as modern countries are relatively recent constructions of diverse assemblages of languages and heritages. To assess the uniformity of ancestry within populations, we used a permutation test to measure, for each pair of populations *x* and *y*, the uniformity with which relationships with *x* are distributed across individuals from *y*. Most comparisons show statistically significant heterogeneity (Figure S2), which is probably due to population substructure (as well as correlations introduced by the pedigree). A notable exception is that nearly all populations showed no significant heterogeneity of numbers of common ancestors with Italian samples, suggesting that most common ancestors shared with Italy lived longer ago than the time that structure within modern-day countries formed.

**Figure 2:**
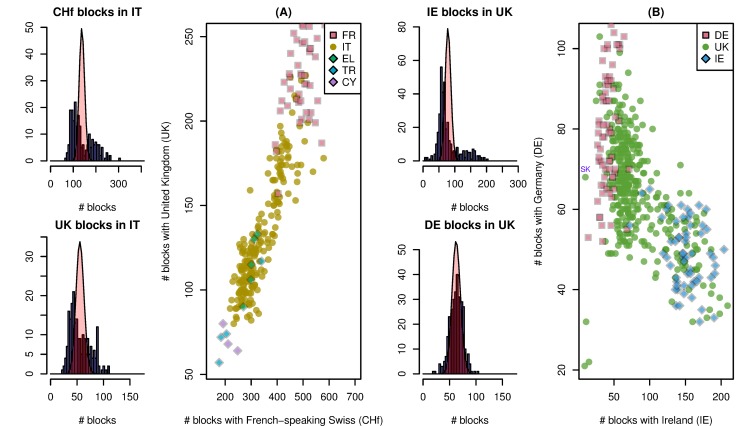
Substructure in (A) Italian and (B) U.K. samples. The leftmost plots of (A) show histograms of the numbers of IBD blocks that each Italian sample shares with any French-speaking Swiss (top) and anyone from the United Kingdom (bottom), overlaid with the expected distribution (Poisson) if there was no dependence between blocks. Next is shown a scatterplot of numbers of blocks shared with French-speaking Swiss and U.K. samples, for all samples from France, Italy, Greece, Turkey, and Cyprus. We see that the numbers of recent ancestors each Italian shares with the French-speaking Swiss and with the United Kingdom are both bimodal, and that these two are positively correlated, ranging continuously between values typical for Turkey/Cyprus and for France. Figure (B) is similar, showing that the substructure within the United Kingdom is part of a continuous trend ranging from Germany to Ireland. The outliers visible in the scatterplot of Figure 2B are easily explained as individuals with immigrant recent ancestors—the three outlying U.K. individuals in the lower left share many more blocks with Italians than all other U.K. samples, and the individual labeled “SK” is a clear outlier for the number of blocks shared with the Slovakian sample.

Two of the more striking examples of substructure are illustrated in Figure 2. Here, we see that variation within countries can be reflective of continuous variation in ancestry that spans a broader geographic region, crossing geographic, political, and linguistic boundaries. Figure 2A shows the distinctly bimodal distribution of numbers of IBD blocks that each Italian shares with both French-speaking Swiss and the United Kingdom, and that these numbers are strongly correlated. Furthermore, the amount that Italians share with these two populations varies continuously from values typical for Turkey and Cyprus, to values typical for France and Switzerland. Interestingly, the Greek samples (EL) place near the middle of the Italian gradient. It is natural to guess that there is a north-south gradient of recency of common ancestry along the length of Italy, and that southern Italy has been historically more closely connected to the eastern Mediterranean.

In contrast, within samples from the United Kingdom and nearby regions, we see a negative correlation between numbers of blocks shared with Irish and numbers of blocks shared with Germans. From our data, we do not know if this substructure is also geographically arranged within the United Kingdom (our sample of which may include individuals from Northern Ireland). However, an obvious explanation of this pattern is that individuals within the United Kingdom differ in the number of recent ancestors shared with Irish, and that individuals with less Irish ancestry have a larger portion of their recent ancestry shared with Germans. This suggests that there is variation across the United Kingdom—perhaps a geographic gradient—in terms of the amount of Celtic versus Germanic ancestry.

The first two principal components of the matrix of genotypes, after suitable manipulations, can reproduce the geographic positions of European populations (e.g., [12]–[14]). Therefore, it is natural to compare the structure we see within populations in terms of IBD sharing to the positions on the principal components map. (A PCA map of these populations, produced by EIGENSTRAT [38], is shown in Figure S4.) It is not known what the geographic resolution of the principal components map is, but if relative positions within populations is meaningful, then comparison of IBD to PCA can stand in for comparison to geography. Indeed, as seen in Figures S5 and S6, the substructure of Figure 2 correlates well with the position on certain principal components, further suggesting that the structure is geographically meaningful. Conversely, since the substructure we see is highly statistically significant, this demonstrates that the scatter of positions within populations on the European PCA map is at least in part signal, rather than noise.

### Europe-Wide Patterns of Relatedness

**Figure 3:**
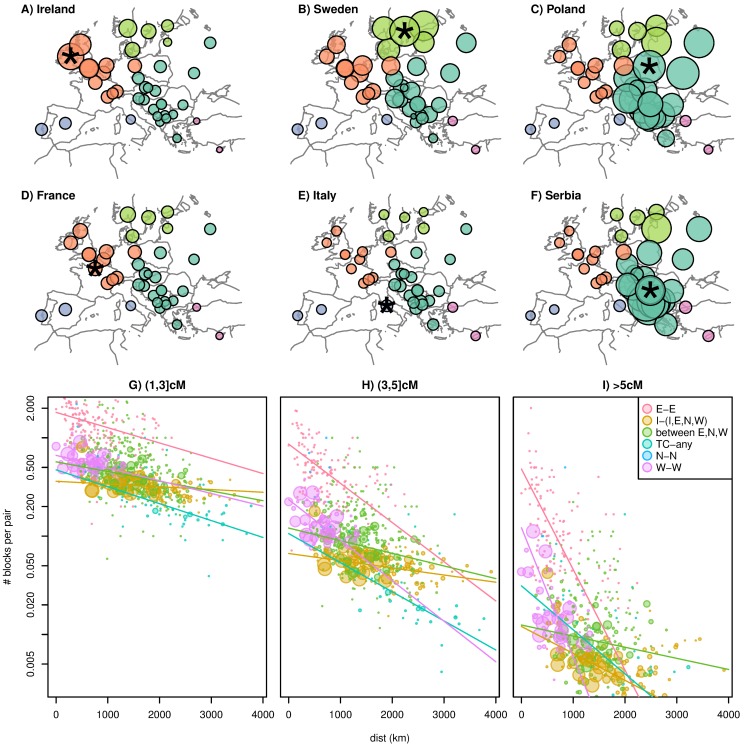
Geographic decay of recent relatedness. In all figures, colors give categories based on the regional groupings of Table 1. (A–F) The area of the circle located on a particular population is proportional to the mean number of IBD blocks of length at least 1 cM shared between random individuals chosen from that population and the population named in the label (also marked with a star). Both regional variation of overall IBD rates and gradual geographic decay are apparent. (G–I) Mean number of IBD blocks of lengths 1–3 cM (oldest), 3–5 cM, and >5 cM (youngest), respectively, shared by a pair of individuals across all pairs of populations; the area of the point is proportional to sample size (number of distinct pairs), capped at a reasonable value; and lines show an exponential decay fit to each category (using a Poisson GLM weighted by sample size). Comparisons with no shared IBD are used in the fit but not shown in the figure (due to the log scale). “E–E,” “N–N,” and “W–W” denote any two populations both in the E, N, or W grouping, respectively; “TC-any” denotes any population paired with Turkey or Cyprus; “I-(I,E,N,W)” denotes Italy, Spain, or Portugal paired with any population except Turkey or Cyprus; and “between E,N,W” denotes the remaining pairs (when both populations are in E, N, or W, but the two are in different groups). The exponential fit for the N–N points is not shown due to the very small sample size. See Figure S8 for an SVG version of these plots where it is possible to identify individual points.

Individuals usually share the highest number of IBD blocks with others from the same population, with some exceptions. For example, individuals in the United Kingdom share more IBD blocks on average, and hence more close genetic ancestors, with individuals from Ireland than with other individuals from the United Kingdom (1.26 versus 1.09 blocks at least 1 cM per pair, Mann-Whitney *p*<10^−10^), and Germans share similarly more with Polish than with other Germans (1.24 versus 1.05, *p = *5.7×10^−6^), a pattern which could be due to recent asymmetric migration from a smaller population into a larger population. In Figure 3 we depict the geography of rates of IBD sharing between populations—that is, the average number of IBD blocks shared by a randomly chosen pair of individuals. Above, maps show the IBD rate relative to certain chosen populations, and below, all pairwise sharing rates are plotted against the geographic distance separating the populations. It is evident that geographic proximity is a major determinant of IBD sharing (and hence recent relatedness), with the rate of pairwise IBD decreasing relatively smoothly as the geographic separation of the pair of populations increases. Note that even populations represented by only a single sample are included, as these showed a surprisingly consistent signal despite the small sample size.

Superimposed on this geographic decay there is striking regional variation in rates of IBD. To further explore this variation, we divided the populations into the four groups listed in Table 1, using geographic location and correlations in the pattern of IBD sharing with other populations (shown in Figure S7). These five groupings are defined as: Europe “E,” lying to the east of Germany and Austria; Europe “N,” lying to the north of Germany and Poland; Europe “W,” to the west of Germany and Austria (inclusive); the Iberian and Italian peninsulas “I”; and Turkey/Cyprus “TC.” Although the general pattern of regional IBD variation is strong, none of these groups have sharp boundaries—for instance, Germany, Austria, and Slovakia are intermediate between E and W. Furthermore, we suspect that the Italian and Iberian peninsulas likely do not group together because of higher shared ancestry with each other, but rather because of similarly low rates of IBD with other European populations. The overall mean IBD rates between these regions are shown in Table 2, and comparisons between different groupings are colored differently in Figure 3G–I, showing that rates of IBD sharing between E populations and between N populations average a factor of about three higher than other comparisons at similar distances. Such a large difference in the rates of IBD sharing between regions cannot be explained by plausible differences in false positive rates or power between populations, since this pattern holds even at the longest length scales, where block identification is nearly perfect.

**Table 2:** Rates of IBD within and between each geographic grouping given in Table 1.

IBD Rate	E	I	N	TC	W
E	2.57	0.44	0.99	0.62	0.53
I	0.44	0.80	0.43	0.41	0.45
N	0.99	0.43	2.62	0.33	0.86
TC	0.62	0.41	0.33	1.43	0.25
W	0.53	0.45	0.86	0.25	0.93

To better understand IBD within these groupings, we show in Figure 3G–I how average numbers of IBD blocks shared, in three different length categories, depend on the geographic distance separating the two populations. Even without taking into account regional variation, mean numbers of shared IBD blocks decay exponentially with distance, and further structure is revealed by breaking out populations by the regional groupings described above. The exponential decays shown for each pair of groupings emphasize how the decay of IBD with distance becomes more rapid for longer blocks. This is expected under models where migration is mostly local, since as one looks further back in time, the distribution of each individual's ancestors is less concentrated around the individual's location (recall Figure 1B). Therefore, the expected number of ancestors shared by a pair of individuals decreases as the geographic distance between the pair increases; and this decrease is faster for more recent ancestry.

This wider spread of older blocks can also explain why the decay of IBD with distance varies significantly by region even if dispersal rates have been relatively constant. For instance, the gradual decay of sharing with the Iberian and Italian peninsulas could occur because these blocks are inherited from much longer ago than blocks of similar lengths shared by individuals in other populations.

Conversely, there is a high level of sharing for “E–E” relationships over a broad range of distances. This is especially true for our shortest (oldest) blocks: individuals in our E grouping share on average more short blocks with individuals in distant E populations than do pairs of individuals in the same W population. We argue below that this is because modern individuals in these locations have a larger proportion of their ancestors in a relatively small population that subsequently expanded.

Having seen the continent-wide patterns of IBD in Figure 3, it is natural to wonder if similar information is contained in single-site summaries of relatedness, such as mean Identity by State (IBS) values across European populations. The mean IBS between populations 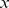 and 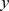 is defined as the probability that two randomly chosen alleles from *x* and *y* are identical (“By State”), averaging over SNPs and individuals. In the analogous plot of IBS against geographic distance (Figure S9), some of the patterns seen in Figure 3 are present, and some are not. For instance, there is a continuous decay with geographic distance (linear, not exponential), and comparisons to the southern “I” group and to Cyprus/Turkey are even more well-separated below the others. On the other hand, the “E–E” comparisons do not show higher IBS than the bulk of the remaining comparisons.

### Ages and Numbers of Common Ancestors

Each block of genome shared IBD by a pair of individuals represents genetic material inherited from one of their genetic common ancestors. Since the distribution of lengths of IBD blocks differs depending on the age of the ancestors—for example, older blocks tend to be shorter—it is possible to use the distribution of lengths of IBD blocks to infer numbers of most recent pairwise genetic common ancestors back through time averaged across pairs of individuals. For this inference, we restricted to blocks longer than 2 cM, where we had good power to detect true IBD blocks. We obtain dates in units of generations in the past, and for ease of discussion convert these to years ago (ya) by taking the mean human generation time to be 30 years [39].

#### Nature of the results on age inference

There are two major difficulties to overcome, however. First, detection is noisy: we do not detect all IBD segments (especially shorter ones), and some of our IBD segments are false positives. This problem can be overcome by careful estimation and modeling of error, described in the Materials and Methods section. The second problem is more serious and unavoidable: the inference problem is extremely “ill conditioned” (in the sense of [40]), meaning in this case that there are many possible histories of shared ancestry that fit the data nearly equally well. For this reason, there is a fairly large, unavoidable limit to the temporal resolution, but we still obtain a good deal of useful information.

**Figure 4:**
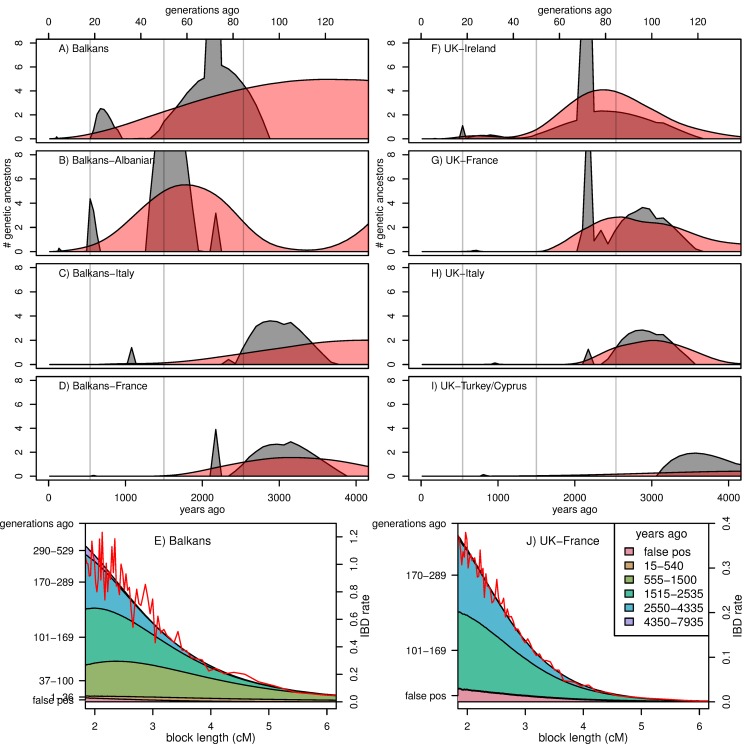
Estimated average number of most recent genetic common ancestors per generation back through time. Estimated average number of most recent genetic common ancestors per generation back through time shared by (A) pairs of individuals from “the Balkans” (former Yugoslavia, Bulgaria, Romania, Croatia, Bosnia, Montenegro, Macedonia, Serbia, and Slovenia, excluding Albanian speakers) and shared by one individual from the Balkans with one individual from (B) Albanian-speaking populations, (C) Italy, or (D) France. The black distribution is the maximum likelihood fit; shown in red is smoothest solution that still fits the data, as described in the Materials and Methods. (E) shows the observed IBD length distribution for pairs of individuals from the Balkans (red curve), along with the distribution predicted by the smooth (red) distribution in (A), as a stacked area plot partitioned by time period in which the common ancestor lived. The partitions with significant contribution are labeled on the left vertical axis (in generations ago), and the legend in (J) gives the same partitions, in years ago; the vertical scale is given on the right vertical axis. The second column of figures (F–J) is similar, except that comparisons are relative to samples from the United Kingdom.

**Figure 5:**
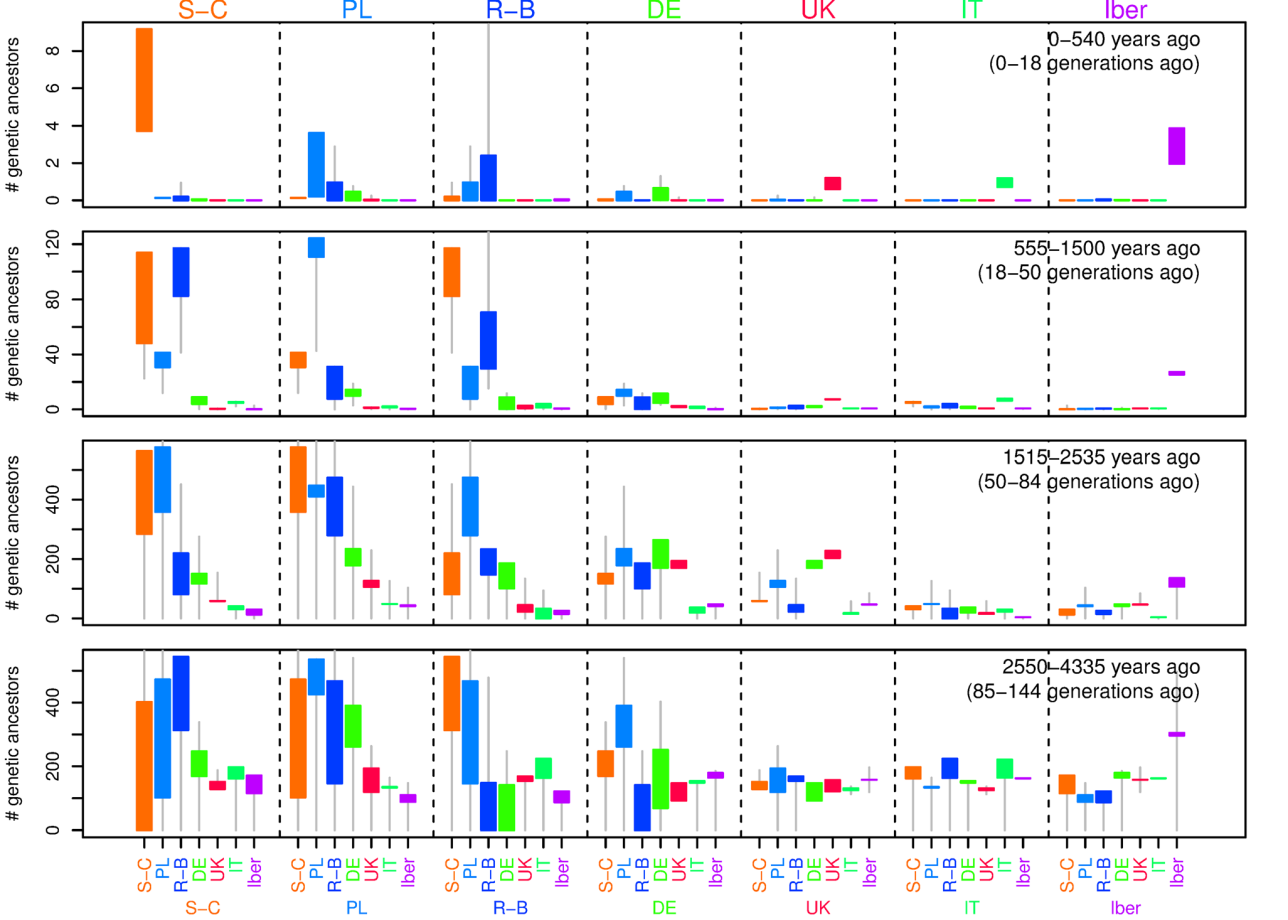
Estimated average total numbers of genetic common ancestors shared per pair of individuals in various pairs of populations, in roughly the time periods 0–500 ya, 500–1,500 ya, 1,500–2,500 ya, and 2,500–4,300 ya. We have combined some populations to obtain larger sample sizes: “S-C” denotes Serbo-Croatian speakers in former Yugoslavia, “PL” denotes Poland, “R-B” denotes Romania and Bulgaria, “DE” denotes Germany, “UK” denotes the United Kingdom, “IT” denotes Italy, and “Iber” denotes Spain and Portugal. For instance, the green bars in the leftmost panels tell us that Serbo-Croatian speakers and Germans most likely share 0–0.25 most recent genetic common ancestor from the last 500 years, 3–12 from the period 500–1,500 years ago, 120–150 from 1500–2,500 ya, and 170–250 from 2,500–4,400 ya. Although the lower bounds appear to extend to zero, they are significantly above zero in nearly all cases except for the most recent period 0–540 ya.

We deal with this uncertainty by describing the set of histories (i.e., historical numbers of common genetic ancestors) that are consistent with the data, summarized in two ways. First, it is useful to look at individual consistent histories, which gives a sense of recurrent patterns and possible historical signals. Figure 4 shows for several populations both the best-fitting history (in black) and the smoothest history that still fits the data (in red). We can make general statements if they hold across all (or most) consistent histories. Second, we can summarize the entire set of consistent histories by finding confidence intervals (bounds) for the total number of common ancestors aggregated in certain time periods. These are shown in Figure 5, giving estimates (colored bands) and bounds (vertical lines) for the total numbers of genetic common ancestors in each of three time periods, roughly 0–500 ya, 500–1,500 ya, and 1,500–2,500 ya (“ya” denotes “years ago”). Figure S12 (and S13) is a version of Figure 5 with more populations (in coalescent units, respectively), and plots analogous to Figure 4 for all these histories are shown in Figure S16. For a precise description of the problem and our methods, see the Materials and Methods section. We validated the method through simulation (details in Text S1), and found that it performed well to the temporal resolution discussed here. We note that in simulations where the population size changes smoothly, the maximum likelihood solution is often overly peaky, whereas the smoothed solution can smear out the signal of rapid change in population size. In light of that we encourage the reader to view truth as lying somewhere between these two solutions, and to not overinterpret specific peaks in the maximum likelihood, which may occur due to numerical properties of the inference. That said, there are a number of sharp peaks in common ancestry shared across many population comparisons older than 2,000 ya, which may potentially indicate demographic events in a shared ancestral population. A more thorough investigation of these older shared signals would potentially need a more model-based approach, so we restrict ourselves here to talking about the broad differences between the distribution of common shared ancestors between regions.

The time periods we use for these bounds are quite large, but this is unavoidable, because of a trade-off between temporal resolution and uncertainty in numbers of common ancestors. Also note that the lower bounds on numbers of common ancestors during each time interval are often close to zero. This is because one can (roughly speaking) obtain a history with equally good fit by moving ancestors from that time interval into the neighboring ones, resulting in peaks on either side of the selected time interval (see Figure S14), even though these do not generally reflect realistic histories. The reader should also bear in mind that we do not depict the dependence of uncertainty between intervals.

#### Results of age inference

In Figure 4 we show how the age and number of shared pairwise genetic common ancestors changes as we move away from the Balkans (left column) and the United Kingdom (right column), along with two examples of how the observed block length distribution is composed of ancestry from different depths. [The average number of shared pairwise genetic common ancestors from generation *n* is the probability that the most recent common ancestor of a pair at a single site lived in generation *n* (i.e., the coalescent rate) multiplied by the expected number of segments that recombination has broken a pair of individuals' genomes into that many generations back, as shown in the Materials and Methods section.] More plots of this form are shown in Figure S16, and coalescent rates between pairs of populations are shown in the (equivalent) Figure S15.

Most detectable recent common ancestors lived between 1,500 and 2,500 years ago, and only a small proportion of blocks longer than 2 cM are inherited from longer ago than 4,000 years. Obviously, there are a vast number of genetic common ancestors older than this, but the blocks inherited from such common ancestors are sufficiently unlikely to be longer than 2 cM that we do not detect many. For the most part, blocks longer than 4 cM come from 500–1,500 years ago, and blocks longer than 10 cM from the last 500 years.

In most cases, only pairs within the same population are likely to share genetic common ancestors within the last 500 years. Exceptions are generally neighboring populations (e.g., United Kingdom and Ireland). During the period 500–1,500 ya, individuals typically share tens to hundreds of genetic common ancestors with others in the same or nearby populations, although some distant populations have very low rates. Longer ago than 1,500 ya, pairs of individuals from any part of Europe share hundreds of genetic ancestors in common, and some share significantly more.

#### Regional variation: Interesting cases

We now examine some of the more striking patterns we see in more detail.

There is relatively little common ancestry shared between the Italian peninsula and other locations, and what there is seems to derive mostly from longer ago than 2,500 ya. An exception is that Italy and the neighboring Balkan populations share small but significant numbers of common ancestors in the last 1,500 years, as seen in Figures S16 and S17S17. The rate of genetic common ancestry between pairs of Italian individuals seems to have been fairly constant for the past 2,500 years, which combined with significant structure within Italy suggests a constant exchange of migrants between coherent subpopulations.

Patterns for the Iberian peninsula are similar, with both Spain and Portugal showing very few common ancestors with other populations over the last 2,500 years. However, the rate of IBD sharing within the peninsula is much higher than within Italy—during the last 1,500 years the Iberian peninsula shares fewer than two genetic common ancestors with other populations, compared to roughly 30 per pair within the peninsula; Italians share on average only about eight with each other during this period.

The higher rates of IBD between populations in the “E” grouping shown in Figure 3 seem to derive mostly from ancestors living 1,500–2,500 ya, but also show increased numbers from 500–1,500 ya, as shown in Figure 5 and Figure S17. For comparison, the IBD rate is high enough that even geographically distant individuals in these eastern populations share about as many common ancestors as do two Irish or two French-speaking Swiss.

By far the highest rates of IBD within any populations is found between Albanian speakers—around 90 ancestors from 0–500 ya, and around 600 ancestors from 500–1,500 ya (so high that we left them out of Figure 5; see Figure S12). Beyond 1,500 ya, the rates of IBD drop to levels typical for other populations in the eastern grouping.

There are clear differences in the number and timing of genetic common ancestors shared by individuals from different parts of Europe, These differences reflect the impact of major historical and demographic events, superimposed against a background of local migration and generally high genealogical relatedness across Europe. We now turn to discuss possible causes and implications of these results.

## Discussion

Genetic common ancestry within the last 2,500 years across Europe has been shaped by diverse demographic and historical events. There are both continental trends, such as a decrease of shared ancestry with distance; regional patterns, such as higher IBD in eastern and northern populations; and diverse outlying signals. We have furthermore quantified numbers of genetic common ancestors that populations share with each other back through time, albeit with a (unavoidably) coarse temporal resolution. These numbers are intriguing not only because of the differences between populations, which reflect historical events, but the high degree of implied genealogical commonality between even geographically distant populations.

### 

#### Ubiquity of common ancestry

We have shown that typical pairs of individuals drawn from across Europe have a good chance of sharing long stretches of identity by descent, even when they are separated by thousands of kilometers. We can furthermore conclude that pairs of individuals across Europe are reasonably likely to share common genetic ancestors within the last 1,000 years, and are certain to share many within the last 2,500 years. From our numerical results, the average number of genetic common ancestors from the last 1,000 years shared by individuals living at least 2,000 km apart is about 1/32 (and at least 1/80); between 1,000 and 2,000ya they share about one; and between 2,000 and 3,000 ya they share above 10. Since the chance is small that any genetic material has been transmitted along a particular genealogical path from ancestor to descendent more than eight generations deep [8]—about .008 at 240 ya, and 2.5×10^−7^ at 480 ya—this implies, conservatively, thousands of shared genealogical ancestors in only the last 1,000 years even between pairs of individuals separated by large geographic distances. At first sight this result seems counterintuitive. However, as 1,000 years is about 33 generations, and 2^33^ ≈10^10^ is far larger than the size of the European population, so long as populations have mixed sufficiently, by 1,000 years ago everyone (who left descendants) would be an ancestor of every present-day European. Our results are therefore one of the first genomic demonstrations of the counterintuitive but necessary fact that all Europeans are genealogically related over very short time periods, and lends substantial support to models predicting close and ubiquitous common ancestry of all modern humans [7].

The fact that most people alive today in Europe share nearly the same set of (European, and possibly world-wide) ancestors from only 1,000 years ago seems to contradict the signals of long-term, albeit subtle, population genetic structure within Europe (e.g., [13],[14]). These two facts can be reconciled by the fact that even though the distribution of ancestors (as cartooned in Figure 1B) has spread to cover the continent, there remain differences in degree of relatedness of modern individuals to these ancestral individuals. For example, someone in Spain may be related to an ancestor in the Iberian peninsula through perhaps 1,000 different routes back through the pedigree, but to an ancestor in the Baltic region by only 10 different routes, so that the probability that this Spanish individual inherited genetic material from the Iberian ancestor is roughly 100 times higher. This allows the amount of genetic material shared by pairs of extant individuals to vary even if the set of ancestors is constant.

#### Relation to single-site summaries

Other work has studied fine-scale differentiation between populations within Europe based on statistics such as *F_ST_*, IBS (e.g., [14],[18]), or PCA [13], that summarize in various ways single-marker correlations, averaged across loci. Like rates of IBD, these measures of differentiation can be thought of as weighted averages of past coalescent rates [41]–[44], but take much of their information from much more distant times (tens of thousands of generations). As expected, we have seen both strong consistency between these measures and IBD (e.g., the decay with geographic distance), as well as distinct patterns (e.g., higher sharing in eastern Europe). These results highlight the fact that long segments of IBD contain information about much more recent events than do single-site summaries, information that can be leveraged to learn about the timing of these events.

#### Limitations of sampling

A concern about our results is that the European individuals in the POPRES dataset were all sampled in either Lausanne or London. This might bias our results, for instance, if an immigrant community originated mostly from a particular small portion of their home population, thereby sharing a particularly high number of recent common ancestors with each other. We see remarkably little evidence that this is the case: there is a high degree of consistency in numbers of IBD blocks shared across samples from each population, and between neighboring populations. For instance, we could argue that the high degree of shared common ancestry among Albanian speakers was because most of these sampled originated from a small area rather than uniformly across Albania and Kosovo. However, this would not explain the high rate of IBD between Albanian speakers and neighboring populations. Even populations from which we only have one or two samples, which we at first assumed would be unusably noisy, provide generally reliable, consistent patterns, as evidenced by, for example, Figure S3.

Conversely, it might be a concern that individuals sampled in Lausanne or London are more likely to have recent ancestors more widely dispersed than is typical for their population of origin. This is a possibility we cannot discard, and if true, would mean there is more structure within Europe than what we detect. However, by the incredibly rapid spread of ancestry, this is unlikely to have an effect over more than a few generations and so does not pose a serious concern about our results about the ubiquitous levels of common ancestry. Fine-scale geographic sampling of Europe as a whole is needed to address these issues, and these efforts are underway in a number of populations (e.g., [45]–[48]).

Finally, we have necessarily taken a narrow view of European ancestry as we have restricted our sample to individuals who are not outliers with respect to genetic ancestry, and when possible to those having all four grandparents drawn from the same county. Clearly the ancestry of Europeans is far more diverse than those represented here, but such steps seemed necessary to make best initial use of this dataset.

#### Ages of particular common ancestors

We have shown that the problem of inferring the average distribution of genetic common ancestors back through time has a large degree of fundamental uncertainty. The data effectively leave a large number of degrees of freedom unspecified, so one must either describe the set of possible histories, as we do, and/or use prior information to restrict these degrees of freedom.

A related but far more intractable problem is to make a good guess of how long ago a *certain* shared genetic common ancestor lived, as personal genome services would like to do, for instance: if you and I share a 10 cM block of genome IBD, when did our most recent common ancestor likely live? Since the mean length of an IBD block inherited from five generations ago is 10 cM, we might expect the average age of the ancestor of a 10 cM block to be from around five generations. However, a direct calculation from our results says that the typical age of a 10 cM block shared by two individuals from the United Kingdom is between 32 and 52 generations (depending on the inferred distribution used). This discrepancy results from the fact that you are *a priori* much more likely to share a common genetic ancestor further in the past, and this acts to skew our answers away from the naive expectation—even though it is unlikely that a 10 cM block is inherited from a particular shared ancestor from 40 generations ago, there are a great number of such older shared ancestors. This also means that estimated ages must depend drastically on the populations' shared histories: for instance, the age of such a block shared by someone from the United Kingdom with someone from Italy is even older, usually from around 60 generations ago. This may not apply to ancestors from the past very few (perhaps less than eight) generations, from whom we expect to inherit multiple long blocks—in this case, we can hope to infer a specific genealogical relationship with reasonable certainty (e.g., [49],[50]), although even then care must be taken to exclude the possibility that these multiple blocks have not been inherited from distinct common ancestors.

Although the sharing of a long genomic segment can be an intriguing sign of some recent shared ancestry, the ubiquity of shared genealogical ancestry only tens of generations ago across Europe (and likely the world, [7]) makes such sharing unsurprising, and assignment to particular genealogical relationships impossible. What is informative about these chance sharing events from distant ancestors is that they provide a fine-scale view of an individual's distribution of ancestors (e.g., Figure 3), and that in aggregate they can provide an unprecedented view into even small-scale human demographic history.

#### Where do your *n* ^th^ cousins live?

Our results also offer a way to understand the geographic location of individuals of a given degree of relatedness. The values of Figure 5 (and Figure S12) can be interpreted as the distribution of distant cousins for any reference population—for instance, the set of bars for Poland (“PL”) in the top row shows that a randomly chosen distant cousin of a Polish individual with the common ancestor living in the past 500 years most likely lives in Poland but has a reasonable chance of living in the Balkan peninsula or Germany. Here “randomly chosen” means chosen randomly proportional to the paths through the pedigree—concretely, take a random walk back through the pedigree to an ancestor in the appropriate time period, and then take a random walk back down. If one starts in Poland, then the chance of arriving in, say, Romania is proportional to the average number of (genetic) common ancestors shared by a pair from Poland and Romania, which is exactly the number estimated in Figure 5.

### The Signal of History

As we have shown, patterns of IBD provide ample but noisy geographic and temporal signals, which can then be connected to historical events. Rigorously making such connections is difficult, due to the complex recent history of Europe, controversy about the demographic significance of many events, and uncertainties in inferring the ages of common ancestors. Nonetheless, our results can be plausibly connected to several historical and demographic events.

#### The migration period

One of the striking patterns we see is the relatively high level of sharing of IBD between pairs of individuals across eastern Europe, as high or higher than that observed within other, much smaller populations. This is consistent with these individuals having a comparatively large proportion of ancestry drawn from a relatively small population that expanded over a large geographic area. The “smooth” estimates of Figure 4 (and more generally Figures 5 and S17) suggest that this increase in ancestry stems from around 1,000–2,000 ya, since during this time pairs of eastern individuals are expected to share a substantial number of common ancestors, while this is only true of pairs of noneastern individuals if they are from the same population. For example, even individuals from widely separated eastern populations share about the same amount of IBD as do two Irish individuals (see Figure S3), suggesting that this ancestral population may have been relatively small.

This evidence is consistent with the idea that these populations derive a substantial proportion of their ancestry from various groups that expanded during the “migration period” from the fourth through ninth centuries [51]. This period begins with the Huns moving into eastern Europe towards the end of the fourth century, establishing an empire including modern-day Hungary and Romania, and continues in the fifth century as various Germanic groups moved into and ruled much of the western Roman empire. This was followed by the expansion of the Slavic populations into regions of low population density beginning in the sixth century, reaching their maximum by the 10th century [52]. The eastern populations with high rates of IBD are highly coincident with the modern distribution of Slavic languages, so it is natural to speculate that much of the higher rates were due to this expansion. The inclusion of (non-Slavic speaking) Hungary and Romania in the group of eastern populations sharing high IBD could indicate the effect of other groups (e.g., the Huns) on ancestry in these regions, or because some of the same group of people who elsewhere are known as Slavs adopted different local cultures in those regions. Greece and Albania are also part of this putative signal of expansion, which could be because the Slavs settled in part of these areas (with unknown demographic effect), or because of subsequent population exchange. However, additional work and methods would be needed to verify this hypothesis.

The highest levels of IBD sharing are found in the Albanian-speaking individuals (from Albania and Kosovo), an increase in common ancestry deriving from the last 1,500 years. This suggests that a reasonable proportion of the ancestors of modern-day Albanian speakers (at least those represented in POPRES) are drawn from a relatively small, cohesive population that has persisted for at least the last 1,500 years. These individuals share similar but slightly higher numbers of common ancestors with nearby populations than do individuals in other parts of Europe (see Figure S3), implying that these Albanian speakers have not been a particularly isolated population so much as a small one. Furthermore, our Greek and Macedonian samples share much higher numbers of common ancestors with Albanian speakers than with other neighbors, possibly a result of historical migrations, or else perhaps smaller effects of the Slavic expansion in these populations. It is also interesting to note that the sampled Italians share nearly as much IBD with Albanian speakers as with each other. The Albanian language is a Indo-European language without other close relatives [53] that persisted through periods when neighboring languages were strongly influenced by Latin or Greek, suggesting an intriguing link between linguistic and genealogical history in this case.

#### Italy, Iberia, and France

On the other hand, we find that France and the Italian and Iberian peninsulas have the lowest rates of genetic common ancestry in the last 1,500 years (other than Turkey and Cyprus), and are the regions of continental Europe thought to have been least affected by the Slavic and Hunnic migrations. These regions were, however, moved into by Germanic tribes (e.g., the Goths, Ostrogoths, and Vandals), which suggests that perhaps the Germanic migrations/invasions of these regions entailed a smaller degree of population replacement than the Slavic and/or Hunnic, or perhaps that the Germanic groups were less genealogically cohesive. This is consistent with the argument that the Slavs moved into relatively depopulated areas, while Gothic “migrations” may have been takeovers by small groups of extant populations [54],[55].

In addition to the very few genetic common ancestors that Italians share both with each other and with other Europeans, we have seen significant modern substructure within Italy (i.e., Figure 2) that predates most of this common ancestry, and estimate that most of the common ancestry shared between Italy and other populations is older than about 2,300 years (Figure S16). Also recall that most populations show no substructure with regards to the number of blocks shared with Italians, implying that the common ancestors other populations share with Italy predate divisions within these other populations. This suggests significant old substructure and large population sizes within Italy, strong enough that different groups within Italy share as little recent common ancestry as other distinct, modern-day countries, substructure that was not homogenized during the migration period. These patterns could also reflect in part geographic isolation within Italy as well as a long history of settlement of Italy from diverse sources.

In contrast to Italy, the rate of sharing of IBD within the Iberian peninsula is similar to that within other populations in Europe. There is furthermore much less evidence of substructure within our Iberian samples than within the Italians, as shown in Figure S2. This suggests that the reduced rate of shared ancestry is due to geographic isolation (by distance and/or the Pyrenees) rather than long-term stable substructure within the peninsula.

#### Future directions

Our results show that patterns of recent identity by descent both provide evidence of ubiquitous shared common ancestry and hold the potential to shed considerable light on the complex history of Europe. However, these inferences also quickly run up against a fundamental limit to our ability to infer pairwise rates of recent common genetic ancestry. In order to make a fuller model of European history, we will need to make use of diverse sources of genomic information from large samples, including IBD segments and rare variants [17],[56], and develop methods that can more fully utilize this information across more than pairs of populations. Another profound difficulty is that Europe—and indeed any large continental region—has such complex layers of history, through which ancestry has mixed so greatly, that attempts to connect genetic signals in extant individuals to particular historical events requires the corroboration of other sources of information from many disciplines. For example, the ability to isolate ancient autosomal DNA from individuals who lived during these time periods (as do [20],[21]) will help to overcome some of these profound difficulties. More generally, the quickly falling cost of sequencing, along with the development of new methods, will shed light on the recent demographic and genealogical history of populations of recombining organisms, human and otherwise.

## Materials and Methods

### Description of Data and Data Cleaning

We used the two European subsets of the POPRES dataset—the CoLaus subset, collected in Lausanne, Switzerland, and the LOLIPOP subset, collected in London, England; the dataset is described in [34]. Those collected in Lausanne reported parental and grandparental country of origin; those collected in London did not. We followed [13] in assigning each sample to the common grandparental country of origin when available, and discarding samples whose parents or grandparents were reported as originating in different countries. We took further steps to restrict to individuals whose grandparents came from the same geographic region, first performing principal components analysis on the data using SMARTPCA [57], and excluding 41 individuals who clustered with populations outside Europe (the majority of such were already excluded by self-reported non-European grandparents). These individuals certainly represent an important part of the recent genetic ancestry of Europe, but are excluded because we aim to study events stemming from older patterns of gene flow, and because we do not model the whole-genome dependencies in recently admixed genomes.

We then used PLINK's inference of the fraction of single-marker IBD (Z0, Z1, and Z2; [58]) to identify very close relatives, finding 25 pairs that are first cousins or closer (including duplicated samples), and excluded one individual from each pair. We grouped samples into populations mostly by reported country, but also used reported language in a few cases. Because of the large Swiss sample, we split this group into three by language: French-speaking (CHf), German-speaking (CHd), or other (CH). Many samples reported grandparents from Yugoslavia; when possible we assigned these to a modern-day country by language, and when this was ambiguous or missing, we assigned these to “Yugoslavia.” Most samples from the United Kingdom reported this as their country of origin; however, the few that reported “England” or “Scotland” were assigned this label. This left us with 2,257 individuals from 40 populations; for sample sizes, see Table 1. Table S1 further breaks this down, and unambiguously gives the composition of each population. Physical distances were converted to genetic distances using the hg36 map, and the average human generation time was taken to be 30 years [39].

All figures were produced in R [59], with color palettes from packages colorspace [60] and RColorBrewer [61]. Code implementing all methods described below is provided in Text S2, and is also distributed along with IBD block data sufficient to reproduce the historical analyses through http://www.github.com/petrelharp/euroibd and in the Dryad digital repository [62].

### Calling IBD Blocks

**Figure 6:**
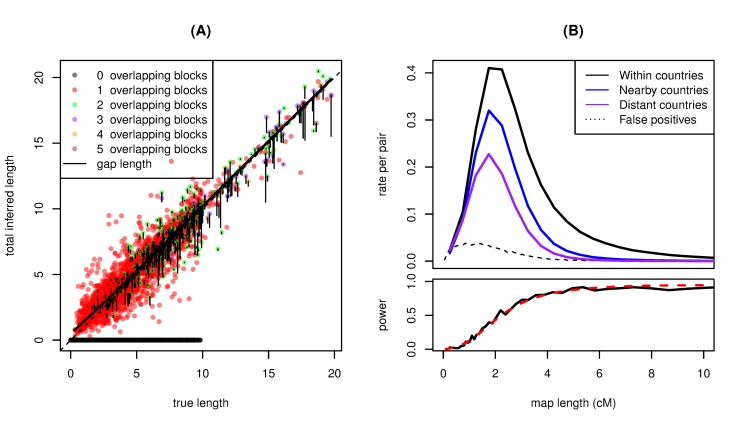
Power and false positive analysis. (A) Bias in inferred length with lines *x* = *y* (dotted) and a loess fit (solid). Each point is a segment of true IBD (copied between individuals), showing its true length and inferred length after postprocessing. Color shows the number of distinct, nonoverlapping segments found by BEAGLE, and the length of the vertical line gives the total length of gaps between such segments that BEAGLE falsely inferred was not IBD (these gaps are corrected by our postprocessing). (B) Estimated false positive rate as a function of length. Observed rates of IBD blocks, per pair and per cM, are also displayed for the purpose of comparison. “Nearby” and “Distant” means IBD between pairs of populations closer and farther away than 1,000 km, respectively. Below, the estimated power as a function of length (black line), together with the parametric fit *c*(*x*) of equation (1) (red dotted curve).

To find blocks of IBD, we used fastIBD (implemented in BEAGLE; [31]), which records putative genomic segments shared IBD by pairs of individuals, along with a score indicating the strength of support. As suggested by the authors, in all cases we ran the algorithm 10 times with different random seeds, and postprocessed the results to obtain IBD blocks. Based on our power simulations described below, we modified the postprocessing procedure recommended by [31] to deal with spurious gaps or breaks introduced into long blocks of IBD by low marker density or switch error, as follows: We called IBD segments by first removing any segments not overlapping a segment seen in at least one other run (as suggested by [31], except with no score cutoff); then merging any two segments separated by a gap shorter than at least one of the segments and no more than 5 cM long; and finally discarding any merged segments that did not contain a subsegment with score below 10^−9^. As shown in Figure 6, this resulted in a false positive rate of between 8–15% across length categories, and a power of at least 70% above 1 cM, reaching 95% by 4 cM. After postprocessing, we were left with 1.9 million IBD blocks, 1 million of which were at least 2 cM long (at which length we estimate 85% power and a 10% false positive rate).

### Power and False Positive Simulations

All methods to identify haplotypic IBD rely on identifying long regions of near identical haplotypes between pairs of individuals (referred to as identical by state, IBS). However, long IBS haplotypes could potentially also result from the concatenation of multiple shorter blocks of true IBD. While such runs can contain important information about deeper population history (e.g., [3],[24]), we view them as a false positives as they do not represent single haplotypes shared without intervening recombination. The chance of such a false positive IBD segment decreases as the genetic length of shared haplotype increases. However, the density of informative markers also plays a role, because such markers are necessary to infer regions of IBS.

#### Power

If we are to have a reasonable false positive rate, we must accept imperfect power. Power will also vary with the density and informativeness of markers and length of segment considered. For example, it is intuitive that segments of genome containing many rare alleles are easier to identify as IBD. Conversely, rare immigrant segments from a population with different allele frequencies may, if they are shared by multiple individuals within the population, cause higher false positive rates. For these reasons, when estimating statistical power and false positive rate, it is important to use a dataset as similar to the one under consideration as possible. Therefore, to determine appropriate postprocessing criteria and to estimate our statistical power, we constructed a dataset similar to the POPRES with known shared IBD segments as follows: we copied haploid segments randomly between 60 trio-phased individuals of European descent (using only one from each trio) from the HapMap dataset (haplotypes from release #21, 17/07/06 [63]), reoriented alleles to match the strand orientation of POPRES, substituted these for 60 individuals from Switzerland in the POPRES data, and ran BEAGLE on the result as before. These segments were copied between single chromosomes of randomly chosen individuals, for random lengths 0.5–20 cM, with gaps of at least 2 cM between adjacent segments and without copying between the same two individuals twice in a row. When copying, we furthermore introduced genotyping error by flipping alleles independently with a probability of .002 and marking the allele missing with a probability of .023 (error rates were determined from duplicated individuals in the sample as given by [34]). An important feature of the inferred data was that BEAGLE often reported true segments longer than about 5 cM as two or more shorter segments separated by a short gap, which led us to merge blocks as described above.

#### Length bias

We also need a reasonably accurate assessment of our bias and false positive rates for our inference of numbers of genetic ancestors from the IBD length spectrum. Although the estimated IBD lengths were approximately unbiased, we fit a parametric model to the relationship between true and inferred lengths after removing inferred blocks less than 1 cM long. A true IBD block of length *x* is missed entirely with probability 1–*c*(*x*), and is otherwise inferred to have length 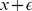; with probability γ(*x*), the error 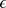 is positive; otherwise it is negative and conditioned to be less than *x*. In either case, 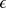 is exponentially distributed; if 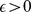, its mean is 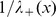, while if 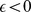, its (unconditional) mean is 1/*λ*_−_(*x*). The parametric forms were chosen by examination of the data; these are, with final parameter values:
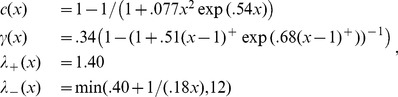
(1)where *z* ^+^ = max(*z*,0). The parameters were found by maximum likelihood, using constrained optimization as implemented in the R package optim [59] separately on three independent pieces: the parameters in *c*(*x*) and γ(*x*), the parameters in *λ*_−_, and finally the parameters in *λ*_+_; the fit is shown in Figure S10.

#### False positive rate

To estimate the false positive rate, we randomly shuffled segments of diploid genome between individuals from the same population (only those 12 populations with at least 19 samples) so that any run of IBD longer than about 0.5 cM would be broken up among many individuals. Specifically, as we read along the genome we output diploid genotypes in random order; we shuffled this order by exchanging the identity of each output individual with another at independent increments chosen uniformly between 0.1 and 0.2 cM. This ensured that no output individual had a continuous run of length longer than 0.2 cM copied from a single input individual, while also preserving linkage on scales shorter than 0.1 cM. The results are shown in Figure 6B; from these we estimate that the mean density of false positives *x* cM long per pair and per cM is approximately:
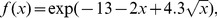
(2)a parametric form again chosen by examination of the data and fit by maximum likelihood.

We found that overall, the false positive rate was around 1/10th of the observed rate, except for very long blocks (longer than 5 cM or so, where it was close to zero), and for very short blocks (less than 1 cM, where it approached 0.4). As fastIBD depends on estimating underlying haplotype frequencies, it is expected to have a higher false positive rate in populations that are more differentiated from the rest of the sample. There was significant variation in false positive rate between different populations, with Spain, Portugal, and Italy showing significantly higher false positive rates than the other populations we examined (see Figure S11). This variation was significant only for blocks shorter than 2 cM across all population pairs, with the exception of pairs of Portuguese individuals, where the upwards bias may be significant as high as 4 cM.

#### Differential sample sizes

Finally, one concern is that as fastIBD calls IBD based on a model of haplotype frequencies in the sample, it may be unduly affected by the large-scale sample size variation across the POPRES sample. In particular, the French-speaking Swiss sample is very large, which could lead to systematic bias in calling IBD in populations closely to the Swiss samples. To investigate this, we randomly discarded 745 French-speaking Swiss (all but 100 of these), and a random sampling of the remaining populations (removing 812 in total, leaving 1,445). We then ran BEAGLE on chromosome 1 of these individuals, postprocessing in the same way as for the full sample. Reassuringly, there was high concordance between the two—we found that 98% (95%) of the blocks longer than 2 cM found in the analysis with the full dataset (respectively, with the subset) were found in both analyses. Overall, more blocks were found by the analysis with the smaller dataset; however, by adjusting the score cutoff by a fixed amount, this difference could be removed, leaving nearly identical length distributions between the two analyses. This is a known attribute of the fastIBD algorithm, and can alternatively be avoided by adjusting the model complexity (S. Browning, personal communication).

We then tested the extent to which the effect of sample size varied by population, for IBD blocks in several length categories (binning block lengths at 1, 2, 4, and 10 cM). Suppose that *F_xy_* is the number of IBD blocks found between populations *x* and *y* in the analysis of the full dataset, and *S_xy_* is the number found in the analysis of the smaller dataset (counted between the same individuals each time). We then assume that *F_xy_* and *S_xy_* are Poisson with mean 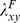 and 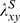, respectively, so that conditioned on N*_xy_ = F_xy_*+*S_xy_* (the total number of blocks), *S_xy_* is binomial with parameters N*_xy_* and 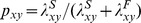. We are looking for deviations from the null model under which the effect of smaller sample size affects all population pairs equally, so that 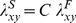 for some constant *C*. We therefore fit a binomial GLM [64] with a logit link, with terms corresponding to each population—in other words:
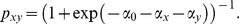
We found statistically significant variation by population (i.e., several nonzero *α_x_*), but all effect sizes were in the range of 0–4%; estimated parameters are listed in Table S2. Notably, the coefficient corresponding to the French-speaking Swiss (the population with the largest change in sample size) was fairly small. We also fit the model not assuming additivity when *x* = *y*—that is, adding coefficients *α_xx_* to the formula for *p_xx_*—but these were not significant. These results suggest that sample size variation across the POPRES data has only minor effects on the distribution of IBD blocks shared across populations.

### IBD Rates along the Genome

To look for regions of unusual levels of IBD and to examine our assumption of uniformity, we compared the density of IBD tracts of different lengths along the genome, in Figure S1. To do this, we first divided blocks up into nonoverlapping bins based on length, with cutpoints at 1, 2.5, 4, 6, 8, and 10 cM. We then computed at each SNP the number of IBD blocks in each length bin that covered that site. To control for the effect of nearby SNP density on the ability to detect IBD, we then computed the residuals of a linear regression predicting number of overlapping IBD blocks using the density of SNPs within 3 cM. To compare between bins, we then normalized these residuals, subtracting the mean and dividing by the standard deviation; these “z-scores” for each SNP are shown in Figure S1.

### Correlations in IBD Rates across Populations

We noted repeated patterns of IBD sharing across multiple populations (seen in Figure S3), in which certain sets of populations tended to show similar patterns of sharing. To quantify this, we computed correlations between mean numbers of IBD blocks; in Figure S7, we show correlations in numbers of blocks of various lengths. Specifically, if *I*(*x,y*) is the mean number of IBD blocks of the given length shared by an individual from population *x* with a (different) individual from population *y*, there are *n* populations, and 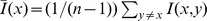, then Figure S7 shows for each *x* and *y*:
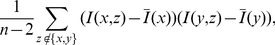
(3)the (Pearson) correlation between *I*(*x,z*) and *I*(*y,z*) ranging across 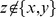. Other choices of block lengths are similar, although shorter blocks show higher overall correlations (due in part to false positives) and longer blocks show lower overall correlations (as rates are noisier, and sharing is more restricted to nearby populations). The geographic groupings of Table 1 were then chosen by visual inspection.

### Substructure

We assessed the overall degree of substructure within each population, by measuring, for each *x* and *y*, the degree of inhomogeneity across individuals of population *x* for shared ancestry with population *y*. We measured inhomogeneity by the standard deviation in number of blocks shared with population *y*, across individuals of population *x*. We assessed the significance by a permutation test, randomly reassigning each block shared between *x* and *y* to a individual chosen uniformly from population *x*, and recomputing the standard deviation, 1,000 times. (If there are *k* blocks shared between *x* and *y* and *m* individuals in population *x*, this is equivalent to putting *k* balls in *m* boxes, tallying how many balls are in each box, and computing the sample standard deviation of the resulting list of numbers.) Note that some degree of inhomogeneity of shared ancestry is expected even within randomly mating populations, due to randomness of the relationship between individuals in the pedigree. These effects are likely to be small if the relationships are suitably deep, but this is still an area of active research [50],[65]. The resulting *p* values are shown in Figure S2. We did not analyze these in detail, particularly as we had limited power to detect substructure in populations with few samples, but note that a large proportion (47%) of the population pairs showed greater inhomogeneity than in all 1,000 permuted samples (i.e., *p*<.001). Some comparisons even with many samples in both populations (where we have considerable power to detect even subtle inhomogeneity) showed no structure whatsoever—in particular, the distribution of numbers of Italian IBD blocks shared by Swiss individuals is not distinguishable from Poisson, indicating a high degree of homogeneity of Italian ancestry across Switzerland.

### Single-Site Summaries

To assess the single marker measures of relatedness across the POPRES sample, we calculated pairwise identity by state, the probability that two alleles sampled at random from a pair of individuals are identical, averaged across SNPs. This was calculated for all pairs of individuals using the “–genome” option in PLINK v1.07 [58], and is shown in Figure S9 with points colored as in Figure 3.

We also calculated principal components of the POPRES genotype data using the EIGENSOFT package v3.0 [57], which were used in identifying outlying individuals and in producing Figures S4, S5, and S6.

### Inferring Ages of Common Ancestors

Here, our aim is to use the distribution of IBD block lengths to infer how long ago the genetic common ancestors were alive from which these IBD blocks were inherited. A pair of individuals who share a block of IBD of genetic length at least *x* have each inherited contiguous regions of genome from a single common ancestor *n* generations ago that overlap for length at least *x*. If we start with the population pedigree, those ancestors from which the two individuals might have inherited IBD blocks are those that can be connected to both by paths through the pedigree. The distribution of possible IBD blocks is determined by the number of links (i.e., the number of meioses) occurring along the two paths.

Throughout the article we informally often refer to ancestors living a certain “number of generations in the past” as if humans were semelparous with a fixed lifetime. Keeping with this, it is natural to write the number of IBD blocks shared by a pair of individuals as the sum over past generations of the number of IBD blocks inherited from that generation. In other words, if N(*x*) is the number of IBD blocks of genetic length at least *x* shared by two individual chromosomes, and N*_n_*(*x*) is the number of such IBD blocks inherited by the two along paths through the pedigree having a total of *n* meioses, then 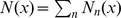. Therefore, averaging over possible choices of pairs of individuals, the mean number of shared IBD blocks can be similarly partitioned as:
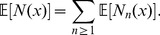
(4)In each successive generation in the past, each chromosome is broken up into successively more pieces, each of which has been inherited along a different path through the pedigree, and any two such pieces of the two individual chromosomes that overlap and are inherited from the same ancestral chromosome contribute one block of IBD. Therefore, the mean number of IBD blocks coming from *n*/2 generations ago is the mean number of possible blocks multiplied by the probability that a particular block is actually inherited by both individuals from the same genealogical ancestor in generation *n*/2. Allowing for overlapping generations, the first part we denote by K(*n*,*x*), the mean number of pieces of length at least *x* obtained by cutting the chromosome at the recombination sites of *n* meioses, and the second part we denote by *μ*(*n*), the probability that the two chromosomes have inherited at a particular site along a path of total length *n* meioses (e.g., their common ancestor at that site lived *n*/2 generations ago). Multiplying these and summing over possible paths, we have that:
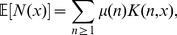
(5)that is, the mean rate of IBD is a linear function of the distribution of the time back to the most recent common ancestor averaged across sites. The distribution *μ*(*n*) is more precisely known as the coalescent time distribution [66],[67], in its obvious adaptation to population pedigrees. As a first application, note that the distribution of ages of IBD blocks above a given length *x* depends strongly on the demographic history—a fraction 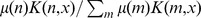 of these are from paths *n* meioses long.

Furthermore, it is easy to calculate that for a chromosome of genetic length *G*:
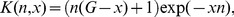
(6)assuming homogeneous Poisson recombination on the genetic map (as well as constancy of the map and ignoring the effect of interference, which is reasonable for the range of 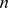 we consider). The mean number of IBD blocks of length at least *x* shared by a pair of individuals across the entire genome is then obtained by summing equation (5) across all chromosomes, and multiplying by 4 (for the four possible chromosome pairs).

Equations (5) and (6) give the relationship between lengths of shared IBD blocks and how long ago the ancestor lived from whom these blocks are inherited. Our goal is to invert this relationship to learn about *μ*(*n*), and hence the ages of the common ancestors underlying our observed distribution of IBD block lengths. To do this, we first need to account for sampling noise and estimation error. Suppose we are looking at IBD blocks shared between any of a set of *n_p_* pairs of individuals, and assume that N(*y*), the number of *observed* IBD blocks shared between any of those pairs of length at least *y*, is Poisson distributed with mean *n_p_M*(*y*), where:
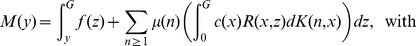
(7)

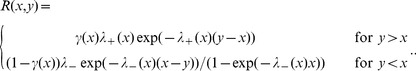
(8)Here the false positive rate *f*(*z*), power *c*(*x*), and the components of the error kernel *R*(*x*,*z*) are estimated as above, with parametric forms given in equations (2) and (1). The Poisson assumption has been examined elsewhere (e.g., [27],[49]) and is reasonable because there is a very small chance of having inherited a block from each pair of shared genealogical ancestors; there a great number of these, and if these events are sufficiently independent, the Poisson distribution will be a good approximation (see, e.g., [68]). If this holds for each pair of individuals, the total number of IBD blocks is also Poisson distributed, with *M* given by the mean of this number across all constituent pairs. (Note that this does *not* assume that each pair of individuals has the same mean number, and therefore does not assume that our set of pairs are a homogeneous population.)

We have therefore a likelihood model for the data, with demographic history (parametrized by 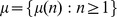) as free parameters. Unfortunately, the problem of inferring *μ* is ill-conditioned (unsurprising due to its similarity of the kernel (6) to the Laplace transform, see [69]), which in this context means that the likelihood surface is flat in certain directions (“ridged”): for each IBD block distribution N(*x*), there is a large set of coalescent time distributions *μ*(*n*) that fit the data equally well. A common problem in such problems is that the unconstrained maximum likelihood solution is wildly oscillatory; in our case, the unconstrained solution is not so obviously wrong, since we are helped considerably by the knowledge that *μ*≥0. For reviews of approaches to such ill-conditioned inverse problems, see, for example, [40] or [70]; the problem is also known as “data unfolding” in particle physics [71]. If one is concerned with finding a point estimate of *μ*, most approaches add an additional penalty to the likelihood, which is known as “regularization” [72] or “ridge regression” [73]. However, our goal is parametric inference, and so we must describe the limits of the “ridge” in the likelihood surface in various directions (which can be seen as maximum *a posteriori* estimates under priors of various strengths).

To do this, we first discretize the data, so that N_i_ is the number of IBD blocks shared by any of a total of *n_p_* distinct pairs of individuals with inferred genetic lengths falling between *x_i_*_–1_ and *x_i_*. We restrict to blocks having a minimum length of 2 cM long, so that *x_0_* = 2. To find a discretization so that each N*_i_* has roughly equal variance, we choose *x_i_* by first dividing the range of block lengths into 100 bins with equal numbers of blocks falling in each, discard any bins longer than 1 cM, and divide the remainder of the range up into 1 cM chunks. To further reduce computational time, we also discretize time, effectively requiring *μ_n_* to be constant on each interval 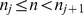, with 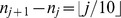, for 1≤*j*≤360—so the resolution is finest for recent times, and the maximum time depth considered is 6,660 meioses, or 99,900 years ago. (The discretization and upper bound on time depth were found to not affect our results.) We then compute by numerical integration (using the function integrate in R) the matrix *L* discretizing the kernel given in equation (7), so that 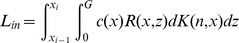 is the kernel that applied to *μ* gives the mean number of true IBD blocks per pair observed with lengths between *x_i_*_–1_ and *x_i_*, and 
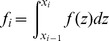
 is the mean number of false positives per pair with lengths in the same interval. We then sum across chromosomes, as before. The likelihood of the data is thus:
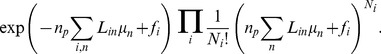
(9)To the (negative) log likelihood we add a penalization *γ*, after rescaling by the number of pairs *n_p_* (which does not affect the result but makes penalization strengths comparable between pairs of populations), and use numerical optimization (the L-BFGS-B method in optim; [59]) to minimize the resulting functional (which omits terms independent of *μ*):
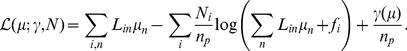
(10)Often we will fix the functional form of the penalization and vary its strength, so that *γ*(*μ*) = *γ_0_z*(*μ*), in which case we will write 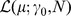 for 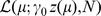.

For instance, the leftmost panels in Figure 4 show the minimizing solutions *μ* for *γ*(*μ*) = 0 (no penalization) and for 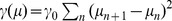 (“roughness” penalization). Because our aim is to describe extremal reasonable estimates *μ*, in this and in other cases, we have chosen the strength of penalization *γ*_0_ to be “as large as is reasonable,” choosing the largest *γ*_0_ such that the minimizing *μ* has log likelihood differing by no more than two units from the unconstrained optimum. This choice of cutoff can be justified as in [74], gave quite similar answers to other methods, and performed well on simulated population histories (see Text S1). This can be thought of as taking the strongest prior that still gives us “reasonable” maximum *a posteriori* answers. Note that the optimization is over *nonnegative* distributions *μ* also satisfying 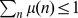 (although the latter condition does not enter in practice).

We would also like to determine bounds on total numbers of shared genetic ancestors who lived during particular time intervals, by determining, for example, the minimum and maximum numbers of such ancestors that are consistent with the data. Such bounds are shown in Figure 5. To obtain a lower bound for the time period between *n*_1_ and *n*_2_ generations, we penalized the total amount of shared ancestry during this interval, using the penalizations 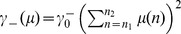, and choosing 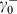 to give a drop of 2 log likelihood units, as described above. The lower bound is then the total amount of coalescence 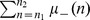 for 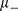 minimizing 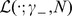. The upper bound is found by penalizing total shared ancestry *outside* this interval—that is, by applying the penalization 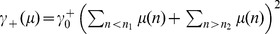. It is almost always the case that lower bounds are zero, since there is sufficient wiggle room in the likelihood surface to explain the observed block length distribution using peaks just below *n*_1_ and above *n*_2_. Examples are shown in Figure S14. On the other hand, upper bounds seem fairly reliable.

In the above we have assumed that the minimizer of 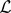 is unique, thus glossing over, for example, finding appropriate starting points for the optimization. In practice, we obtained good starting points by solving the natural approximating least-squares problem, using quadprog [75] in R. We then evaluated uniqueness of the minimizer by using different starting points, and found that if necessary, adding only a very small penalization term was enough to ensure convergence to a unique solution.

#### Testing the method

To test this method, we implemented a whole-genome coalescent IBD simulator, and applied our inference methods to the results under various demography scenarios. We also used these simulations to evaluate the sensitivity of our method to misestimation of power or false positive rates. The simulations, and the results, are described in Text S1; in all cases, the simulations showed that the method performed well to the level of uncertainty discussed throughout the text and confirmed our understanding of the method described above. We also found that misestimation of false positive rate only affects estimated numbers of common ancestors by a comparable amount, and that misestimation of blocks less than 4 cM long mostly affects estimates older than about 2,000 years. Therefore, if our false positive rates above 2 cM are off by 10% (the range that seems reasonable), which would change our estimated numbers of blocks by about 1%, this would only change our estimated numbers of shared ancestors by a few percent.

#### Extending to shorter blocks

We only used blocks longer than 2 cM to infer ages of common ancestors, in part because the model we use does not seem to fit the data below this threshold. Attempts to apply the methods to all blocks longer than 1 cM reveals that there is no history of rates of common ancestry that, under this model, produces a block length distribution reasonably close to the one observed—small but significant deviations occur below about 2 cM. This occurs probably in part because our estimate of false positive rate is expected to be less accurate at these short lengths. Furthermore, our model does not explicitly model the overlap of multiple short IBD segments to create a long segment deriving from different ancestors, which could start to have a significant effect at short lengths. (The effect on long blocks we model as error in length estimation.) This could be incorporated into a model (in a way analogous to [3]), but consideration of when several contiguous blocks of IBD might have few enough differences to be detected as a long IBD block quickly runs into the need for a model of IBD detection, which we here treat as a black box. Use of these shorter blocks, which would allow inference of older ancestry, will need different methods, and probably sequencing rather than genotyping data.

### Numbers of Common Ancestors

Estimated numbers of genetic common ancestors can be found by simply solving for N(0) using an estimate of *μ*(*n*) in equations (5) and (6) (still restricting to genetic ancestors on the autosomes). These tell us that given the distribution *μ*(*n*), the mean number of genetic common ancestors coming from generation *n*/2—that is, the mean number of IBD blocks of *any* length inherited from such common ancestors—is 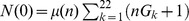, where G_k_ is the total sex-averaged genetic length of the *k^th^* human chromosome. Since the total sex-averaged map length of the human autosomes is about 32 Morgans, this is about 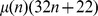. This procedure has been used in Figures 4 and 5.

Converting shared IBD blocks to numbers of shared *genealogical* common ancestors is more problematic. Suppose that modern-day individuals *a* and *b* both have *c* as a grand*^n^* ^–1^parent. Using equation (6) at *x* = 0, we know that the mean number of blocks that *a* and *b* both inherit from *c* is *r*(2*n*), with 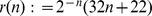, since each block has chance 2^−2*n*^ of being inherited across 2*n* meioses. First treat the endpoints of each distinct path of length *n* back through the pedigree as a grand*^n^* ^–1^parent, so that everyone has exactly 2*^n^* grand*^n^* ^–1^parents, and some ancestors will be grand*^n^* ^–1^parents many times over. Then if *a* and *b* share *m* genetic grand*^n^* ^–1^parents, a moment estimator for the number of genealogical grand*^n^* ^–1^parents is *m*/*r*(*n*). However, the geometric growth of *r*(*n*) means that small uncertainties in *n* have large effects on the estimated numbers of genealogical common ancestors—and we have large uncertainties in *n*.

Despite these difficulties, we can still get some order-of-magnitude estimates. For instance, we estimate that someone from Hungary shares on average about five genetic common ancestors with someone from the United Kingdom between 18 and 50 generations ago. Since 1/*r*(36) = 5.8×10^7^, we would conservatively estimate that for every genetic common ancestor there are tens of millions of genealogical common ancestors. Most of these ancestors must be genealogical common ancestors many times over, but these must still represent at least thousands of distinct individuals.

## Supporting Information

Figure S1 Normalized density of IBD blocks of different lengths, corrected for SNP density, across all autosomes (see Materials and Methods for details). Marked with a grey bar and “c” are the centromeres, and marked with “8p” is a large, segregating inversion [36]. The grey curve along the bottom shows normalized SNP density.
(PDF)

Figure S2Two measures of overdispersal of block numbers across individuals (i.e., substructure): Suppose we have *n* individuals from population *x*, and N*_iy_* is the number of IBD blocks of length at least 1 cM that individual *i* shares with anyone from population *y*. Our statistic of substructure within *x* with respect to *y* is the variance of these numbers, 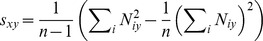. We obtained a “null” distribution for this statistic by randomly reassigning all blocks shared between *x* and *y* to an individual from *x*, and used this to evaluate the strength and the statistical significance of this substructure. (A) Histogram of the “*p* value,” the proportion of 1,000 replicates that showed a variance greater than or equal to the observed variance *s_xy_*, for all pairs of populations *x* and *y* with at least 10 individuals in population *y*. (B) The “*z* score,” which is observed value *s_xy_* minus mean value divided by standard deviation, estimated using 1,000 replicates. The population *x* is shown on the vertical axis, with text labels giving *y*, so for instance, Italians show much more substructure with most other populations than do Irish. Note that sample size still has a large effect—it is easier to see substructure with respect to the Swiss French (*x* = CHf) because the large number of Swiss French samples allows greater resolution. A vertical line is shown at *z = *5. Only pairs of populations with at least three samples in country *x* and 10 samples in country *y* are shown. Because of the log scale, only pairs with a positive *z* score are shown, but no comparisons had *z*<−2.5, and only three had *z*<−2.(PDF)

Figure S3 (A) Mean numbers of IBD blocks of length at least 1 cM per pair of individuals, shown as a modified Cleveland dotchart, with ±2 standard deviations shown as horizontal lines. For instance, on the bottom row we see that someone from the United Kingdom shares on average about one IBD block with someone else from the United Kingdom and slightly less than 0.2 blocks with someone from Turkey. Note that in most cases, the distribution of block numbers is fairly concentrated, and that nearby populations show quite similar patterns.
(PDF)

Figure S4 The positions of our sample on the first two principal components of the genotype matrix, as produced by EIGENSTRAT [38]. Population centroids are marked by text and a transparent circle. Note the correspondence to a map of Europe, after a rotation and flip.
(PDF)

Figure S5 Comparison of Figure 2A in the main text to Figure S4—the axes are self-explanatory; the colors and symbols are the same as in Figure 2A.
(PDF)

Figure S6 Comparison of Figure 2B in the main text to Figure S4—the axes are self-explanatory; the colors and symbols are the same as in Figure 2B. The four outlying U.K. individuals are, as in Figure 2B, three who share a very high number of IBD blocks with Italians, and one who shares a very high number with the Slovakian sample.
(PDF)

Figure S7Correlations in IBD rates, for six different length windows (omitted length windows are similar). If there are *n* populations, *I*(*x*,*y*) is the mean number of blocks in the given length range shared by a pair from populations *x* and *y*, and 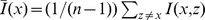, shown is 
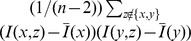
.(PDF)

Figure S8 The same plot as Figure 3G–I, but rendered as an SVG figure with tooltips that allow identification of individual points (using R [61])—open the file in a reasonably compliant browser (e.g., Firefox, Opera) or SVG browser (e.g., squiggle) and hover the mouse over a point of interest to see the label.
(SVG)

Figure S9 Mean IBS (“Identity by State”) against geographic distance, calculated using plink [58] as described in the main text, using the same groups and fitting the same curves as in Figure 3 of the main text. The lowest set of points, roughly following a line, are mean IBS with Turkey; unlike with IBD, mean IBS with Cyprus was significantly higher. In fact, the other rough line of points (between the comparisons to Turkey and the orange points) is almost entirely mean IBS with Cyprus, as well as mean IBS to Slovakia. Since Slovakia is only represented by a single individual in the dataset, we cannot reach further conclusions.
(PDF)

Figure S10Goodness-of-fit for our estimated error distribution—points show data from simulations (described in the text), and lines show the parametric forms of equation (1). Each simulated IBD block of length *x* was either found by BEAGLE (and passed our filters) or was not; and if it was found, it had inferred length 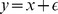, that is, with length error 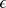. The top figure shows the probability that a segment of a given length is missed entirely (and 1– *c*(*x*)) in green, the probability that 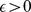 given the segment was found (and γ(*x*)) in black, and the probability that 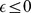 given the segment was found (and 1 – γ(*x*)) in red. The second figure shows the probability density of all positive 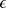 (in black, with 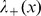), and probability densities of positive 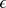 for various categories of true length *x* (colors). The third figure is similar to the second, except that it shows negative 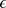. Note that blocks with inferred length *y*<1 were omitted.(PDF)

Figure S11 Estimated false positive rates per pair, compared to the observed rate, as a function of block length. The black dotted curves show the mean number of IBD blocks per pair observed in the false positive simulations (see Materials and Methods), per centiMorgan, binned at 0, 1.2, 1.5, 1.8, 2.1, 2.4, 2.7, 3.2, 4.5, and 7.5 cM, and the parametric fit described in the text. The colored curves show the same quantity, separately for each pair of country comparisons, with the extreme values labeled. No comparisons other than Portugal–Portugal show any significant deviations from the parametric fit above 2 cM. For comparison, the black solid curve shows the mean observed IBD rate across the same set of individuals; note that, for example, the false positive rate for pairs of Portuguese individuals is higher than this at short lengths because the observed IBD rate between Portuguese at short block lengths is much higher than the overall mean.
(PDF)

Figure S12 Estimated total numbers of genetic common ancestors shared by various pairs of populations, in roughly the time periods 0–500 ya, 500–1,500 ya, 1,500–2,500 ya, and 2,500–4,300 ya. The population groupings are: “AL,” Albanian speakers (Albania and Kosovo); “S-C,” Serbo-Croatian speakers in Bosnia, Croatia, Serbia, Montenegro, and Yugoslavia; “R-B,” Romania and Bulgaria; “UK,” United Kingdom, England, Scotland, Wales; “Iber,” Spain and Portugal; “Bel,” Belgium and the Netherlands; “Bal,” Latvia, Finland, Sweden, Norway, and Denmark; and denotes a single population with the same abbreviations as in Table 1 otherwise.
(PDF)

Figure S13 For those who are used to thinking in effective population sizes, the equivalent figure to Figure S12, except with coalescent rate on the vertical axis, rather than numbers of most recent genetic common ancestors.
(PDF)

Figure S14 An example of the set of consistent histories (as coalescent distributions *μ*(*n*)) used to find upper and lower bounds in Figures S12 and Figure 5. The example shown is Poland–Germany, “MLE” is the maximum likelihood history, “smooth” is the smoothest consistent history, and the remaining plots show the histories giving lower and upper bounds for the referenced time intervals (in numbers of generations). In each case, the segment of time on which we are looking for a bound is shaded.
(PDF)

Figure S15 For those who are used to thinking in effective population sizes, the equivalent figure to Figure 4, except with coalescent rate on the vertical axis, rather than numbers of most recent genetic common ancestors.
(PDF)

Figure S16 The maximum likelihood history (grey) and smoothest consistent history (red) for all pairs of population groupings of Figure S12 (including those of Figure 5). Each panel is analogous to a panel of Figure 4; time scale is given by vertical grey lines every 500 years. For these plots on a larger scale, see Figure S17.
(PDF)

Figure S17 All inversions shown in Figure S16, one per page (225 pages total). There is one page per pair of comparisons used in Figure 5. On each page, there is one large plot, showing 10 distinct consistent histories (numbers of genetic ancestors back through time), and below are 10 histograms of IBD block length, one for each consistent history, showing both the observed distribution and the partitioning of blocks into age categories predicted by that history. The names of the two groupings are shown in the upper right: “pointy” is the unconstrained maximum likelihood solution; “smooth” is the smoothest consistent history; “*a*–*b* lower” is the history used to find the lower bound for the time period *a*–*b* generations ago in Figure 5; and “*a*–*b* upper” is the history used to find the corresponding upper bound. Each of these are described in more detail in the Materials and Methods section.
(PDF)

Text S1 Description of validation by simulation of the age inference method.
(PDF)

Text S2 Compressed archive of code used to process data and produce all figures.
(GZ) Click here for additional data file.

Table S1 The composition of our populations. “COUNTRY_SELF” is the reported country of origin; “COUNTRY_GFOLX” is the country of origin of all reported grandparents (individuals with reported grandparents from different countries were removed); “PRIMARY_LANGUAGE” is the reported primary language; “Population” is our population label; and *n* gives the number of individuals falling in this category.
(PDF)

Table S2Estimated coefficients describing the effect of changing population sample size, as described in the text (Materials and Methods, “Differential Sample Sizes”). Stars denote statistical significance: “*” corresponds to *p*<.05 and “**” corresponds to *p*<.01. The coefficients are from a binomial GLM with a logit link function, applied to the number of IBD segments detected in the same set of individuals run with and without an additional 812 individuals. For instance, the top three entries in the left column tell us that if *F* is the number of segments greater than 1 cM found between Albanian and Austrian individuals in analysis with the full dataset, and *S* is the corresponding number in the analysis with only the subset, that the model predicts that 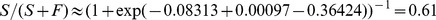 (plus binomial sampling noise). Note that coefficients producing effect sizes larger than 4% (e.g., Austria for 0–1 cM) all correspond to populations with small sample sizes, and are not significant.(PDF)

## Abbreviations

IBD: identity by descent
SNP: single nucleotide polymorphism
ya: years ago
