# Supplementary figures and images for: The Geography of Recent Genetic Ancestry across Europe
Source: PLoS Biol. 2013 May 7;11(5):e1001555. doi: 10.1371/journal.pbio.1001555 (PMC3646727; doi:10.1371/journal.pbio.1001555)

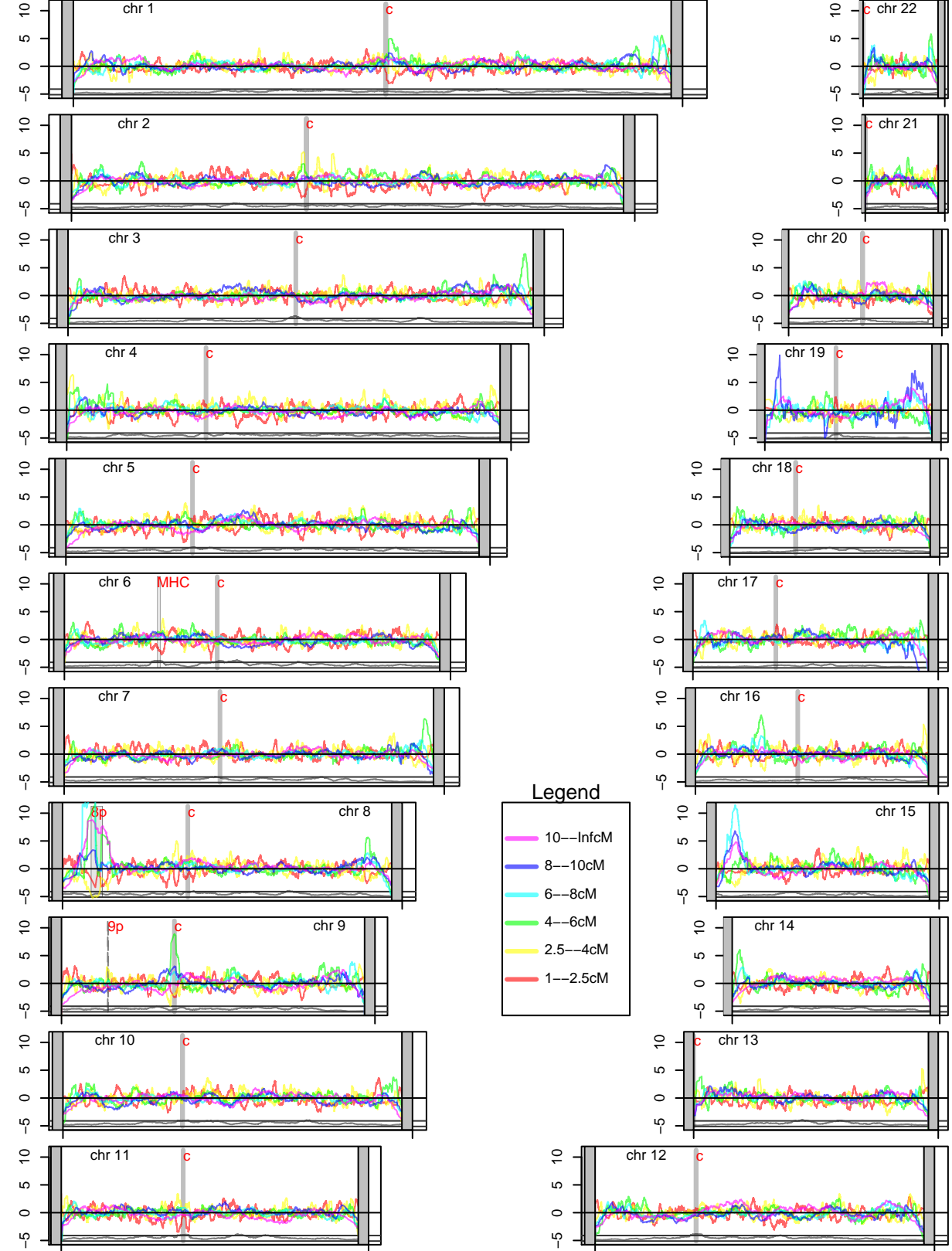

Supplement: Figure S1 — Normalized density of IBD blocks of different lengths, corrected for SNP density, across all autosomes (see Materials and Methods for details). Marked with a grey bar and “c” are the centromeres, and marked with “8p” is a large, segregating inversion [36]. The grey curve along the bottom shows normalized SNP density. (PDF) [file pbio.1001555.s001.pdf]

## A) Significance of substructure

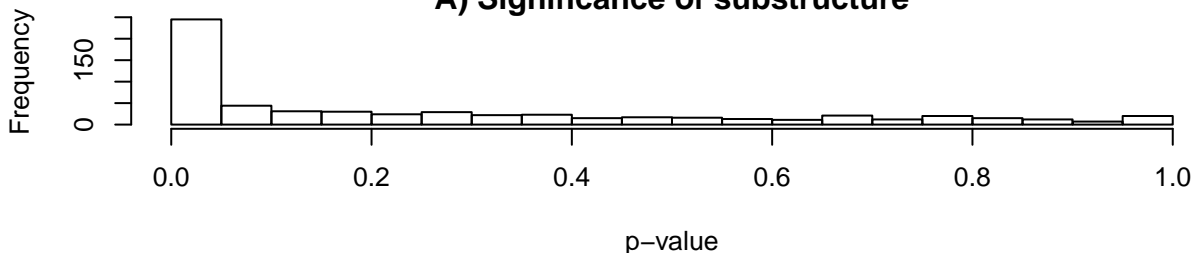

## B) Degree of substructure

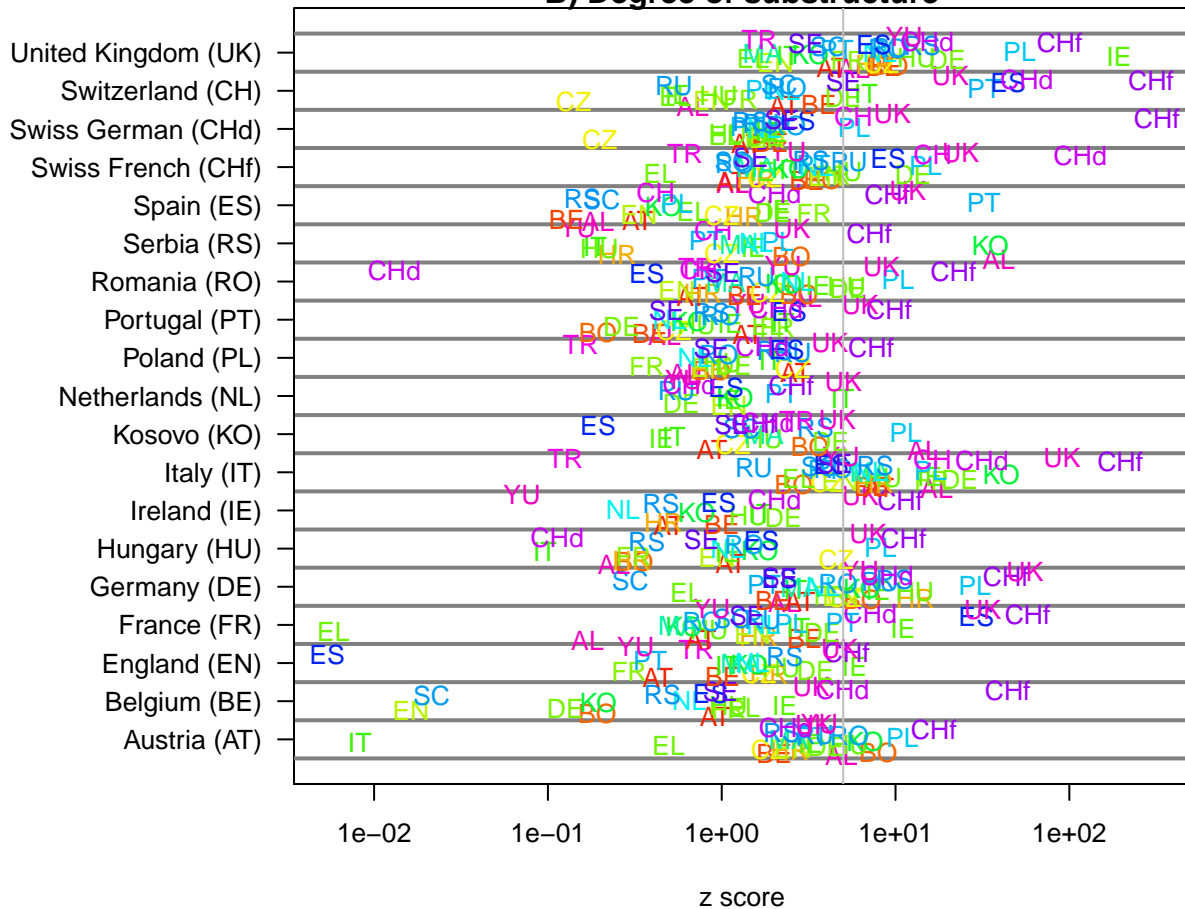

Supplement: Figure S2 — Two measures of overdispersal of block numbers across individuals (i.e., substructure): Suppose we have n individuals from population x, and Niy is the number of IBD blocks of length at least 1 cM that individual i shares with anyone from population y. Our statistic of substructure within x with respect to y is the variance of these numbers, . We obtained a “null” distribution for this statistic by randomly reassigning all blocks shared between x and y to an individual from x, and used this to evaluate the strength and the statistical significance of this substructure. (A) Histogram of the “p value,” the proportion of 1,000 replicates that showed a variance greater than or equal to the observed variance sxy, for all pairs of populations x and y with at least 10 individuals in population y. (B) The “z score,” which is observed value sxy minus mean value divided by standard deviation, estimated using 1,000 replicates. The population x is shown on the vertical axis, with text labels giving y, so for instance, Italians show much more substructure with most other populations than do Irish. Note that sample size still has a large effect—it is easier to see substructure with respect to the Swiss French (x = CHf) because the large number of Swiss French samples allows greater resolution. A vertical line is shown at z = 5. Only pairs of populations with at least three samples in country x and 10 samples in country y are shown. Because of the log scale, only pairs with a positive z score are shown, but no comparisons had z<−2.5, and only three had z<−2. (PDF) [file pbio.1001555.s002.pdf]

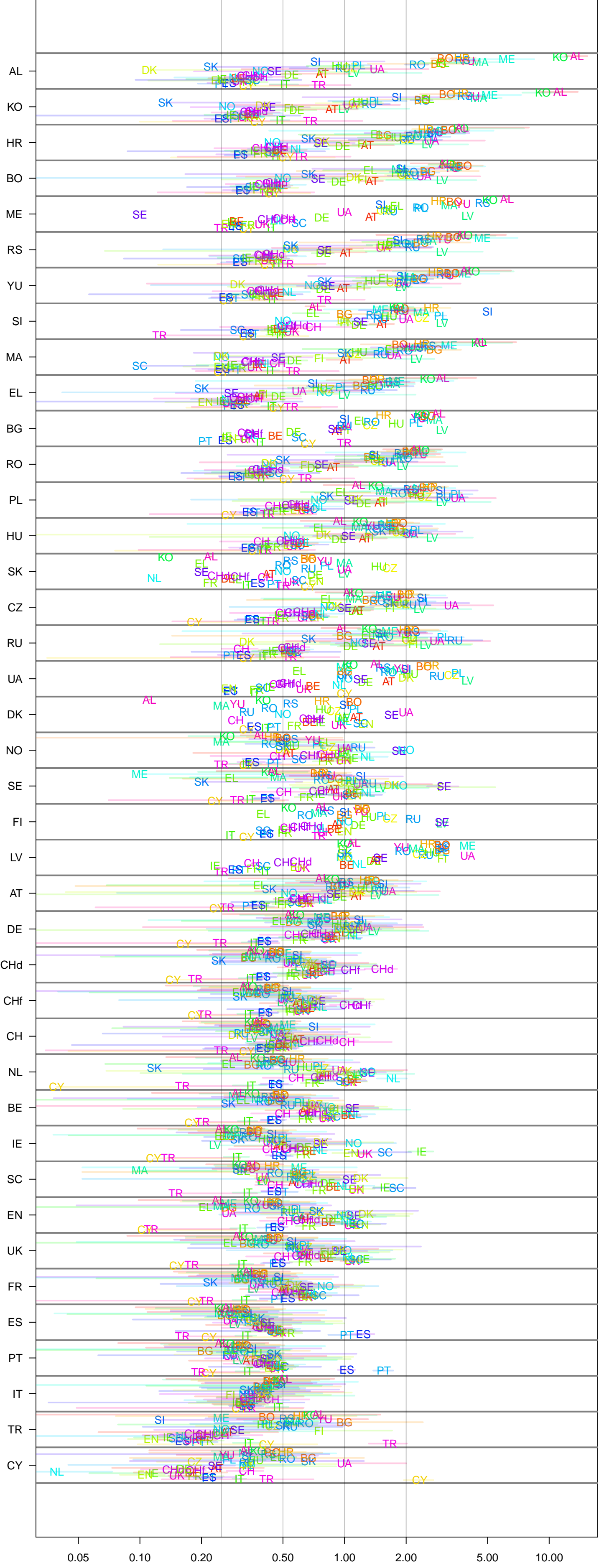

rate per pair

Supplement: Figure S3 — (A) Mean numbers of IBD blocks of length at least 1 cM per pair of individuals, shown as a modified Cleveland dotchart, with ±2 standard deviations shown as horizontal lines. For instance, on the bottom row we see that someone from the United Kingdom shares on average about one IBD block with someone else from the United Kingdom and slightly less than 0.2 blocks with someone from Turkey. Note that in most cases, the distribution of block numbers is fairly concentrated, and that nearby populations show quite similar patterns. (PDF) [file pbio.1001555.s003.pdf]

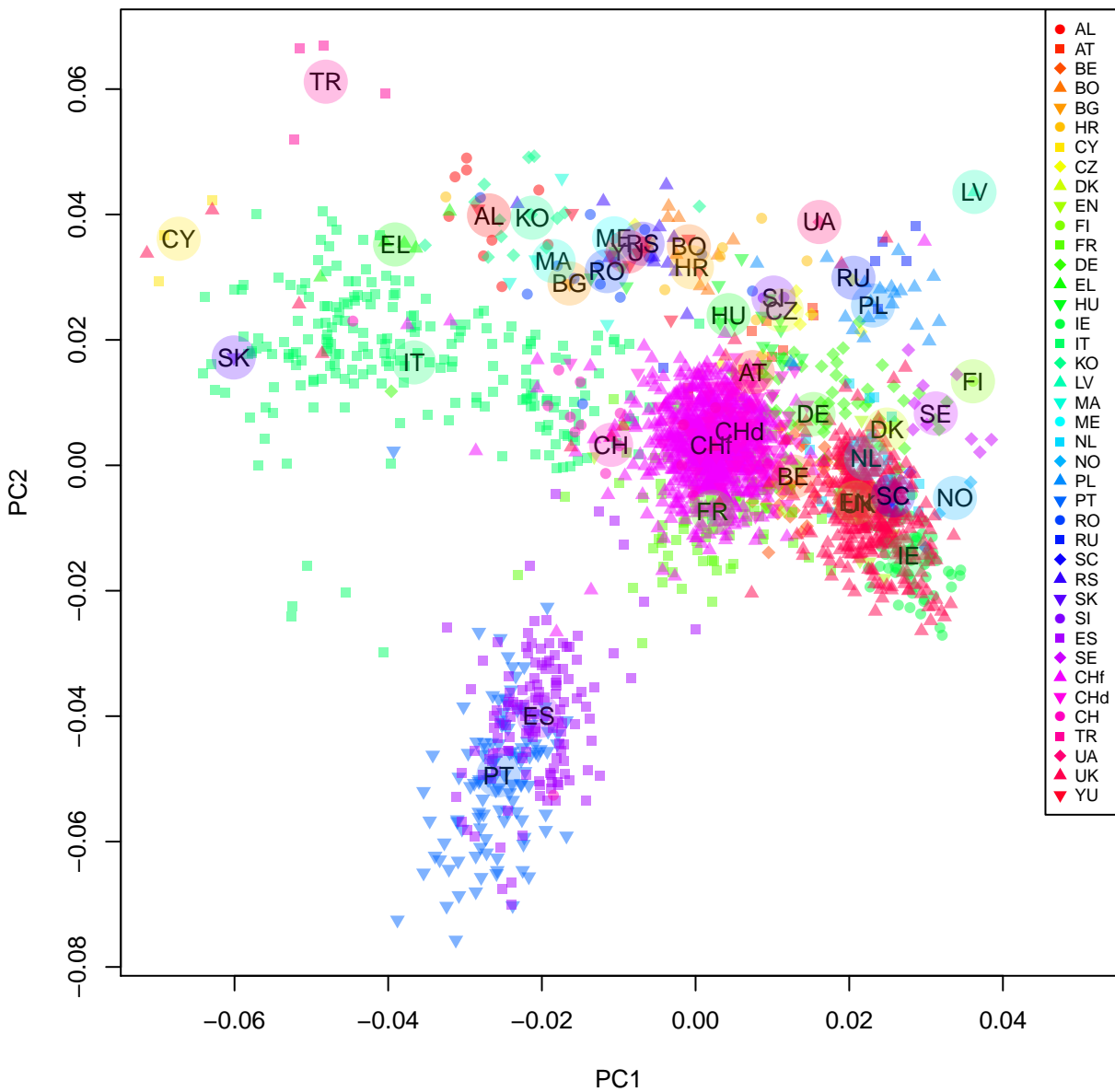

Supplement: Figure S4 — The positions of our sample on the first two principal components of the genotype matrix, as produced by EIGENSTRAT [38]. Population centroids are marked by text and a transparent circle. Note the correspondence to a map of Europe, after a rotation and flip. (PDF) [file pbio.1001555.s004.pdf]

# blocks with UK

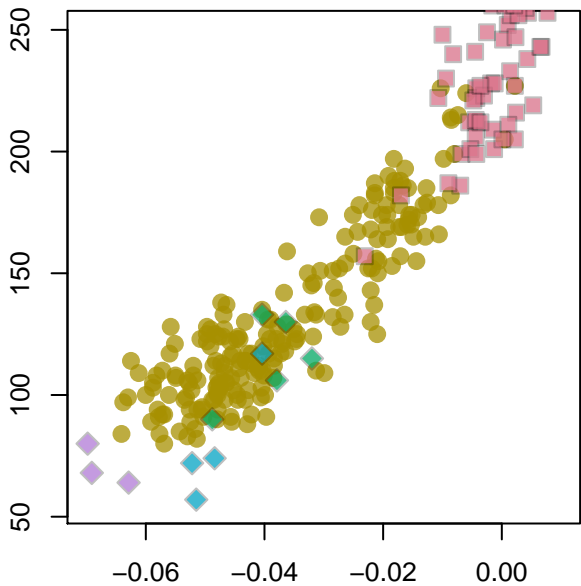

PC1

# blocks with UK

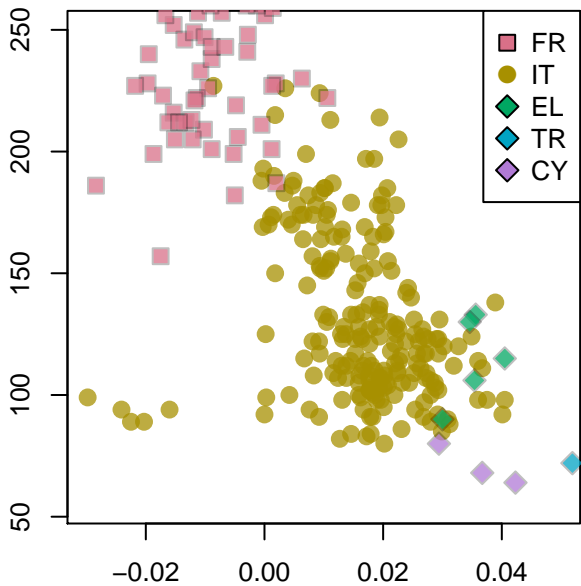

PC2

# blocks with CHF

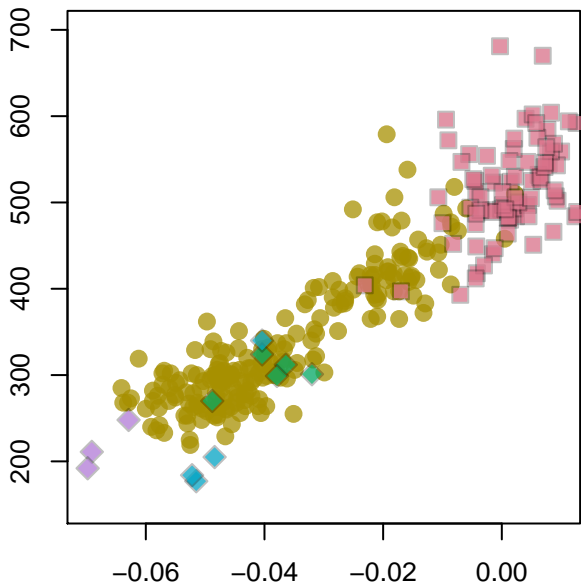

PC1

# blocks with CHF

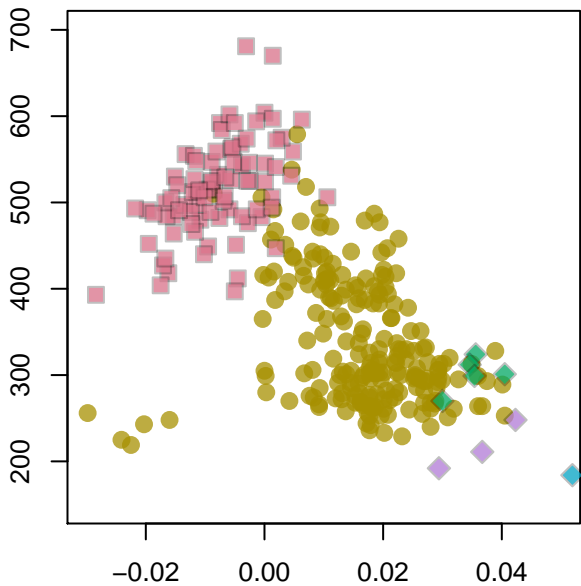

PC2

Supplement: Figure S5 — Comparison of Figure 2A in the main text to Figure S4—the axes are self-explanatory; the colors and symbols are the same as in Figure 2A. (PDF) [file pbio.1001555.s005.pdf]

# blocks with Germany

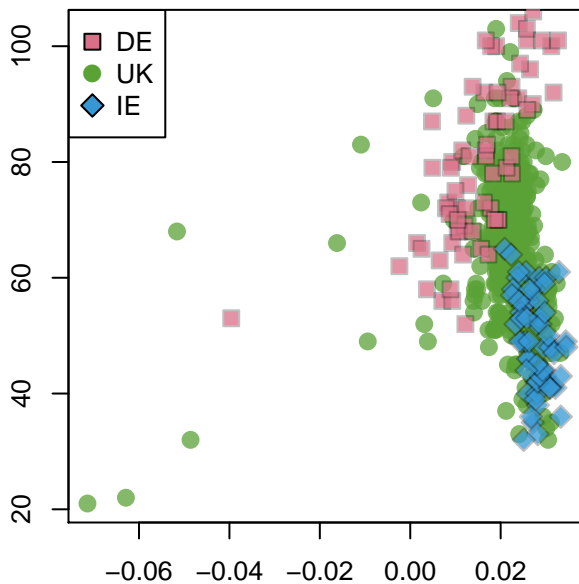

# blocks with Germany

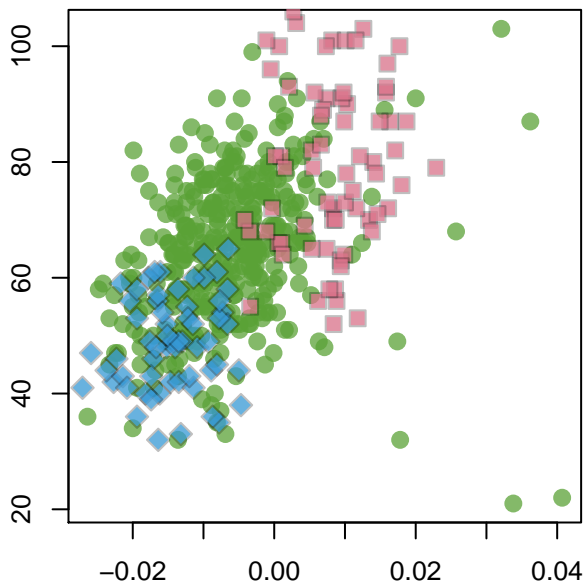

# blocks with Ireland

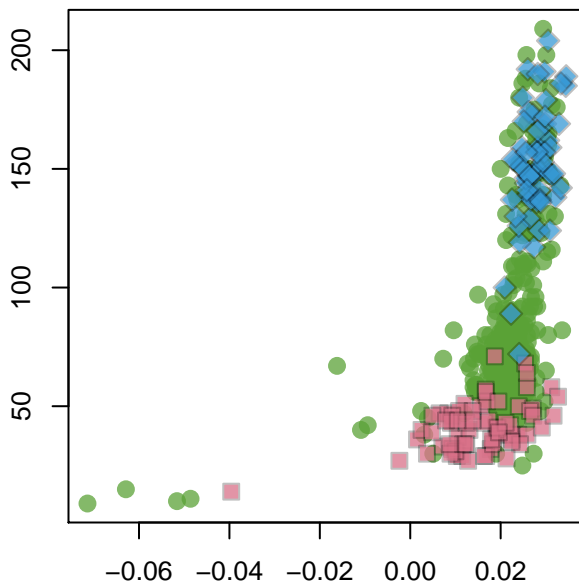

# blocks with Ireland

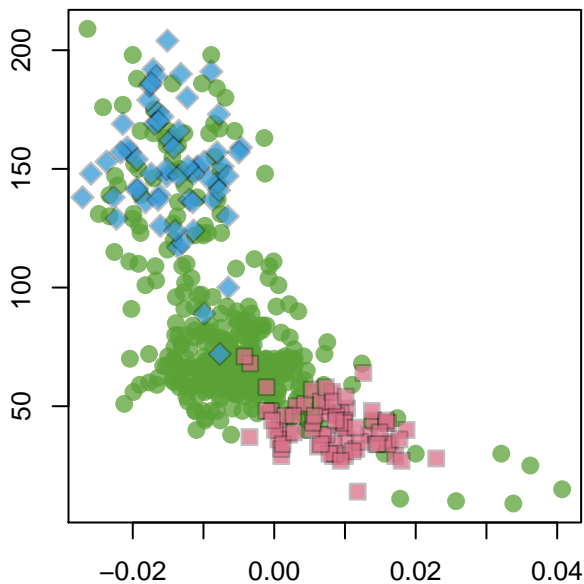

Supplement: Figure S6 — Comparison of Figure 2B in the main text to Figure S4—the axes are self-explanatory; the colors and symbols are the same as in Figure 2B. The four outlying U.K. individuals are, as in Figure 2B, three who share a very high number of IBD blocks with Italians, and one who shares a very high number with the Slovakian sample. (PDF) [file pbio.1001555.s006.pdf]

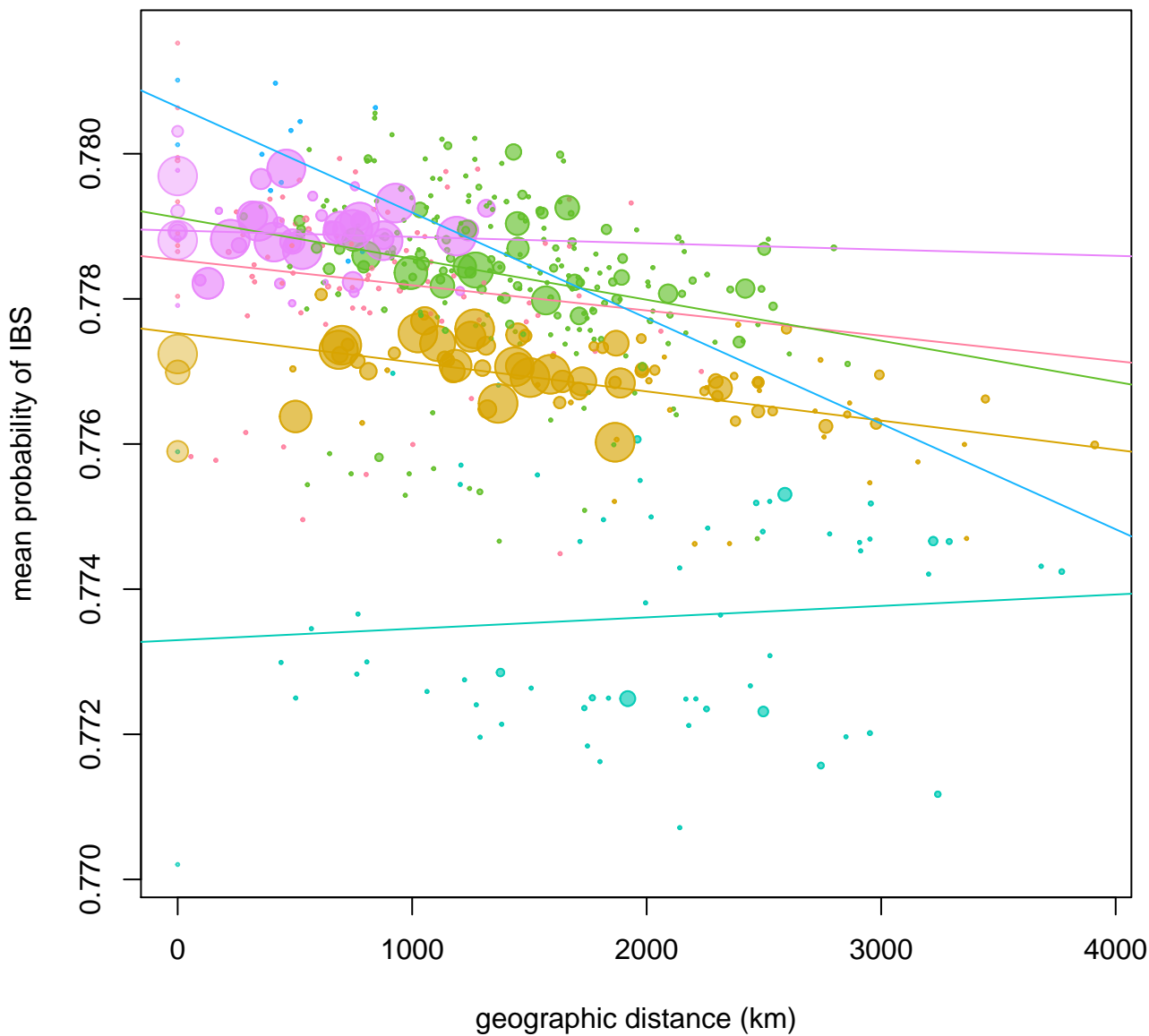

Supplement: Figure S9 — Mean IBS (“Identity by State”) against geographic distance, calculated using plink [58] as described in the main text, using the same groups and fitting the same curves as in Figure 3 of the main text. The lowest set of points, roughly following a line, are mean IBS with Turkey; unlike with IBD, mean IBS with Cyprus was significantly higher. In fact, the other rough line of points (between the comparisons to Turkey and the orange points) is almost entirely mean IBS with Cyprus, as well as mean IBS to Slovakia. Since Slovakia is only represented by a single individual in the dataset, we cannot reach further conclusions. (PDF) [file pbio.1001555.s009.pdf]

fitted forms for  $c(x)$  and  $\gamma(x)$ 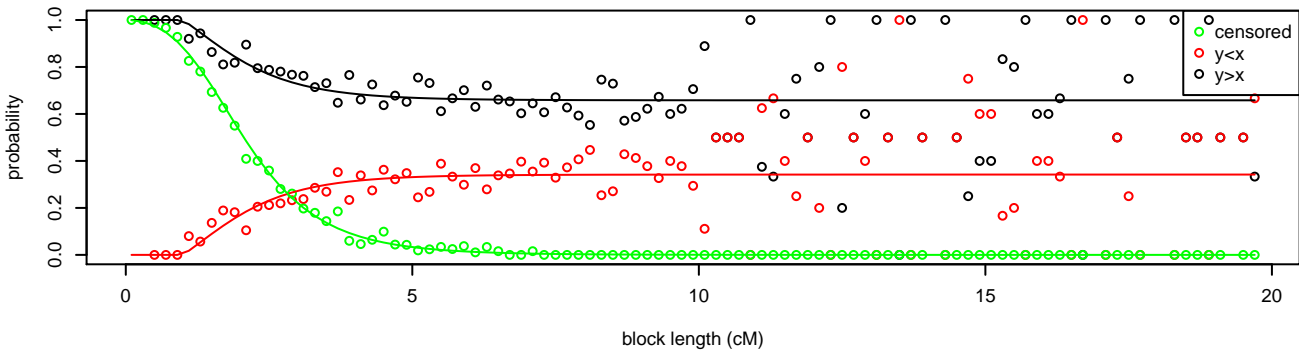fitted form for  $\lambda_+(x)$ 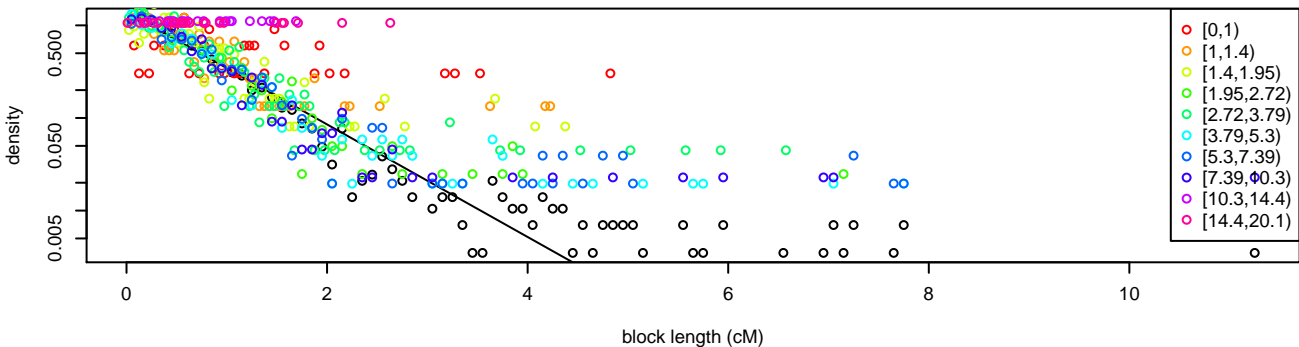fitted form for  $\lambda_-(x)$ 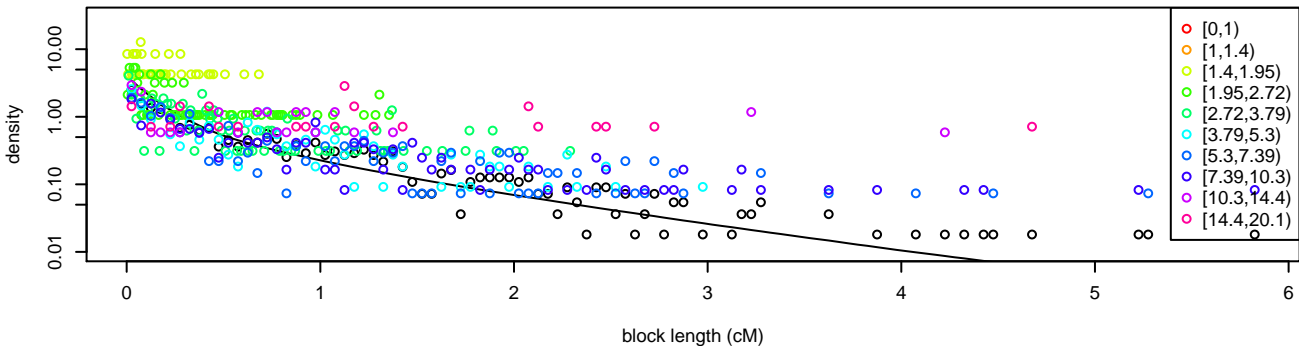

Supplement: Figure S10 — Goodness-of-fit for our estimated error distribution—points show data from simulations (described in the text), and lines show the parametric forms of equation (1). Each simulated IBD block of length x was either found by BEAGLE (and passed our filters) or was not; and if it was found, it had inferred length , that is, with length error . The top figure shows the probability that a segment of a given length is missed entirely (and 1– c(x)) in green, the probability that given the segment was found (and γ(x)) in black, and the probability that given the segment was found (and 1 – γ(x)) in red. The second figure shows the probability density of all positive (in black, with ), and probability densities of positive for various categories of true length x (colors). The third figure is similar to the second, except that it shows negative . Note that blocks with inferred length y<1 were omitted. (PDF) [file pbio.1001555.s010.pdf]

## False positive rates

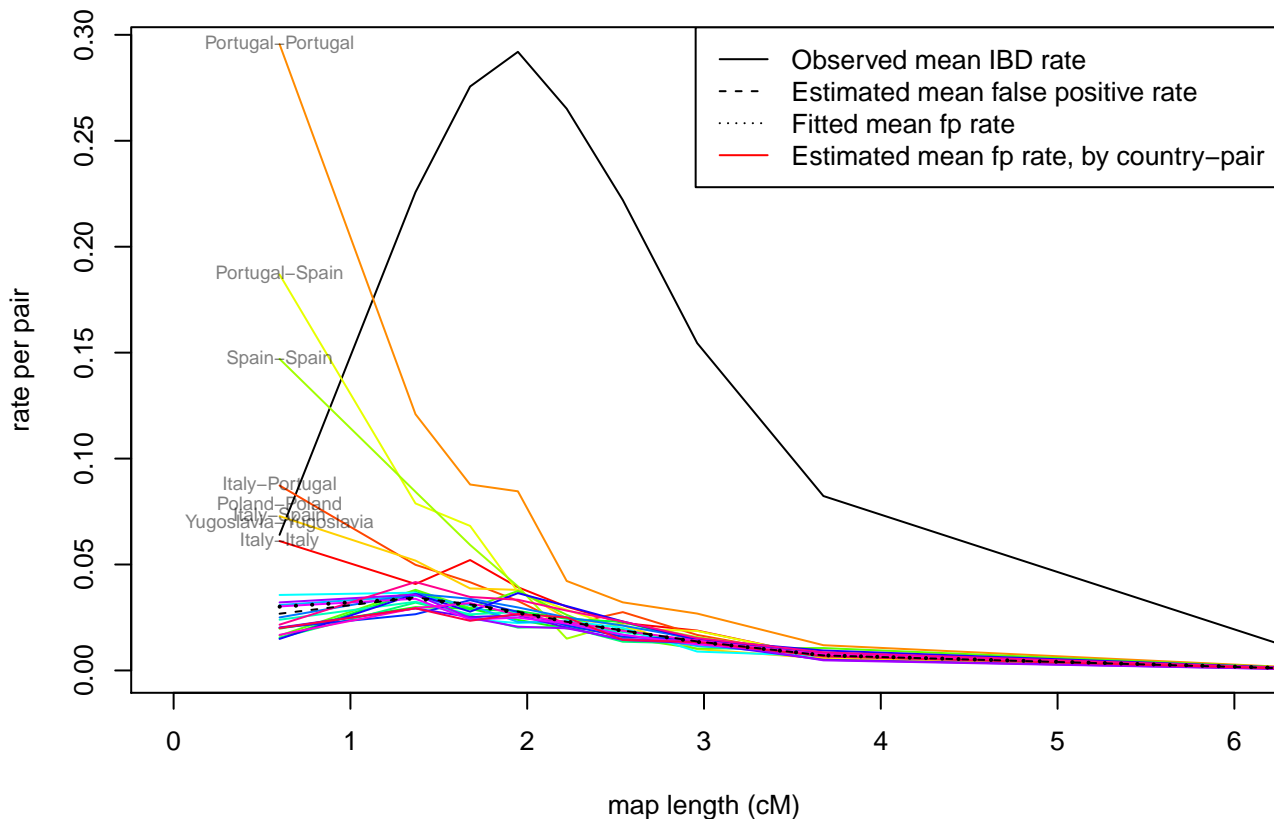

Supplement: Figure S11 — Estimated false positive rates per pair, compared to the observed rate, as a function of block length. The black dotted curves show the mean number of IBD blocks per pair observed in the false positive simulations (see Materials and Methods), per centiMorgan, binned at 0, 1.2, 1.5, 1.8, 2.1, 2.4, 2.7, 3.2, 4.5, and 7.5 cM, and the parametric fit described in the text. The colored curves show the same quantity, separately for each pair of country comparisons, with the extreme values labeled. No comparisons other than Portugal–Portugal show any significant deviations from the parametric fit above 2 cM. For comparison, the black solid curve shows the mean observed IBD rate across the same set of individuals; note that, for example, the false positive rate for pairs of Portuguese individuals is higher than this at short lengths because the observed IBD rate between Portuguese at short block lengths is much higher than the overall mean. (PDF) [file pbio.1001555.s011.pdf]

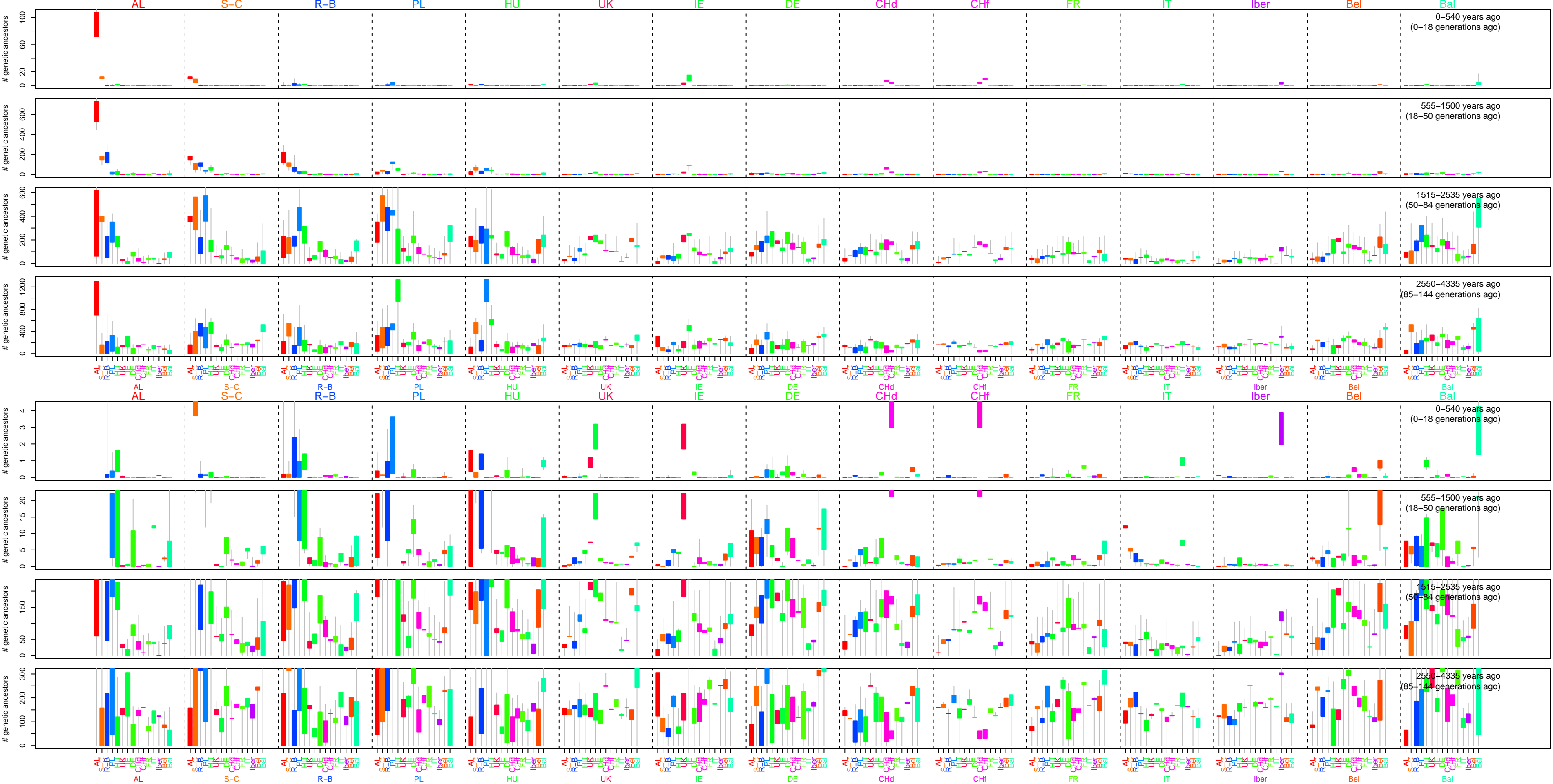

Supplement: Figure S12 — Estimated total numbers of genetic common ancestors shared by various pairs of populations, in roughly the time periods 0–500 ya, 500–1,500 ya, 1,500–2,500 ya, and 2,500–4,300 ya. The population groupings are: “AL,” Albanian speakers (Albania and Kosovo); “S-C,” Serbo-Croatian speakers in Bosnia, Croatia, Serbia, Montenegro, and Yugoslavia; “R-B,” Romania and Bulgaria; “UK,” United Kingdom, England, Scotland, Wales; “Iber,” Spain and Portugal; “Bel,” Belgium and the Netherlands; “Bal,” Latvia, Finland, Sweden, Norway, and Denmark; and denotes a single population with the same abbreviations as in Table 1 otherwise. (PDF) [file pbio.1001555.s012.pdf]

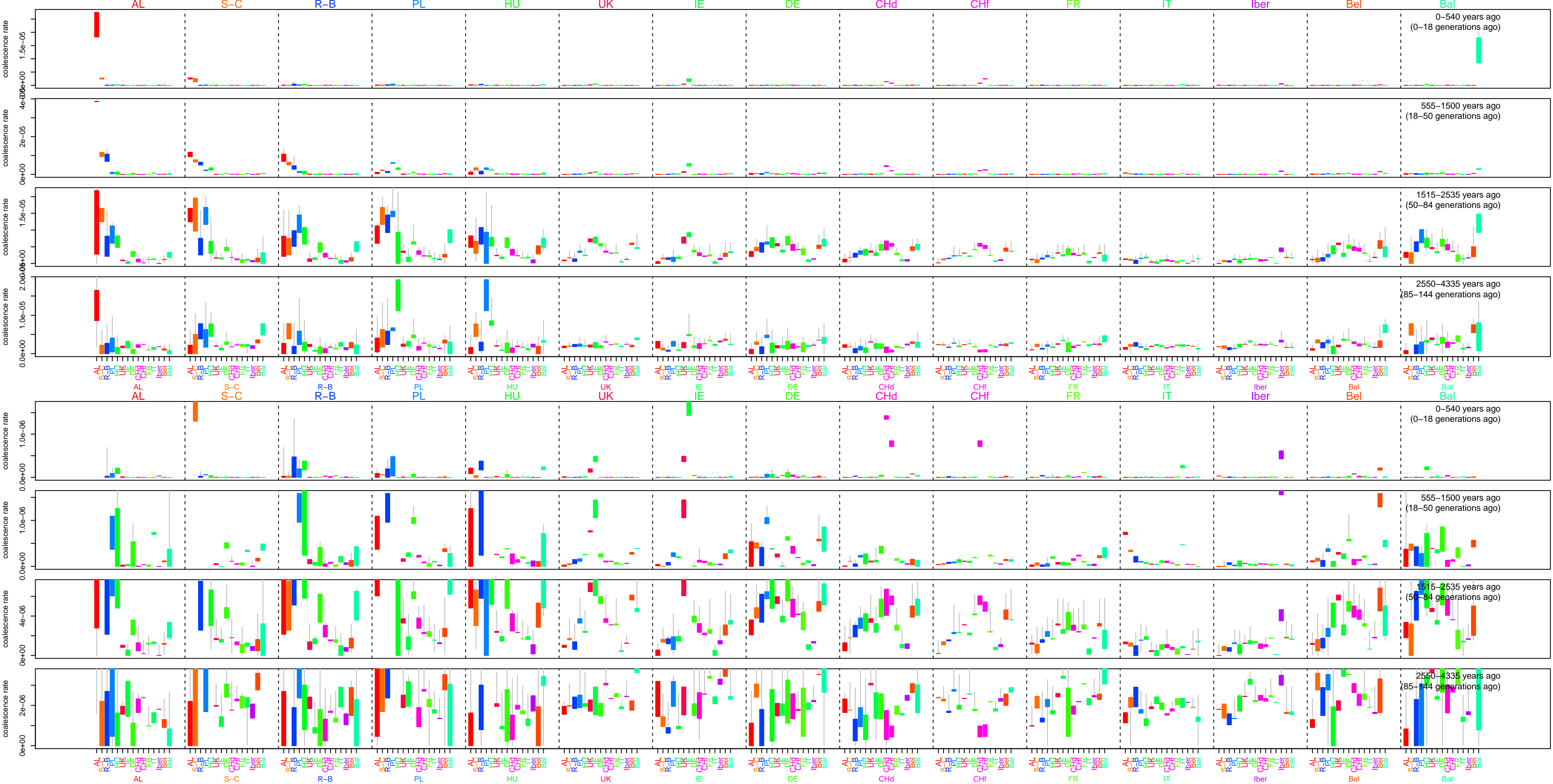

Supplement: Figure S13 — For those who are used to thinking in effective population sizes, the equivalent figure to Figure S12, except with coalescent rate on the vertical axis, rather than numbers of most recent genetic common ancestors. (PDF) [file pbio.1001555.s013.pdf]

# Germany-Poland consistent histories

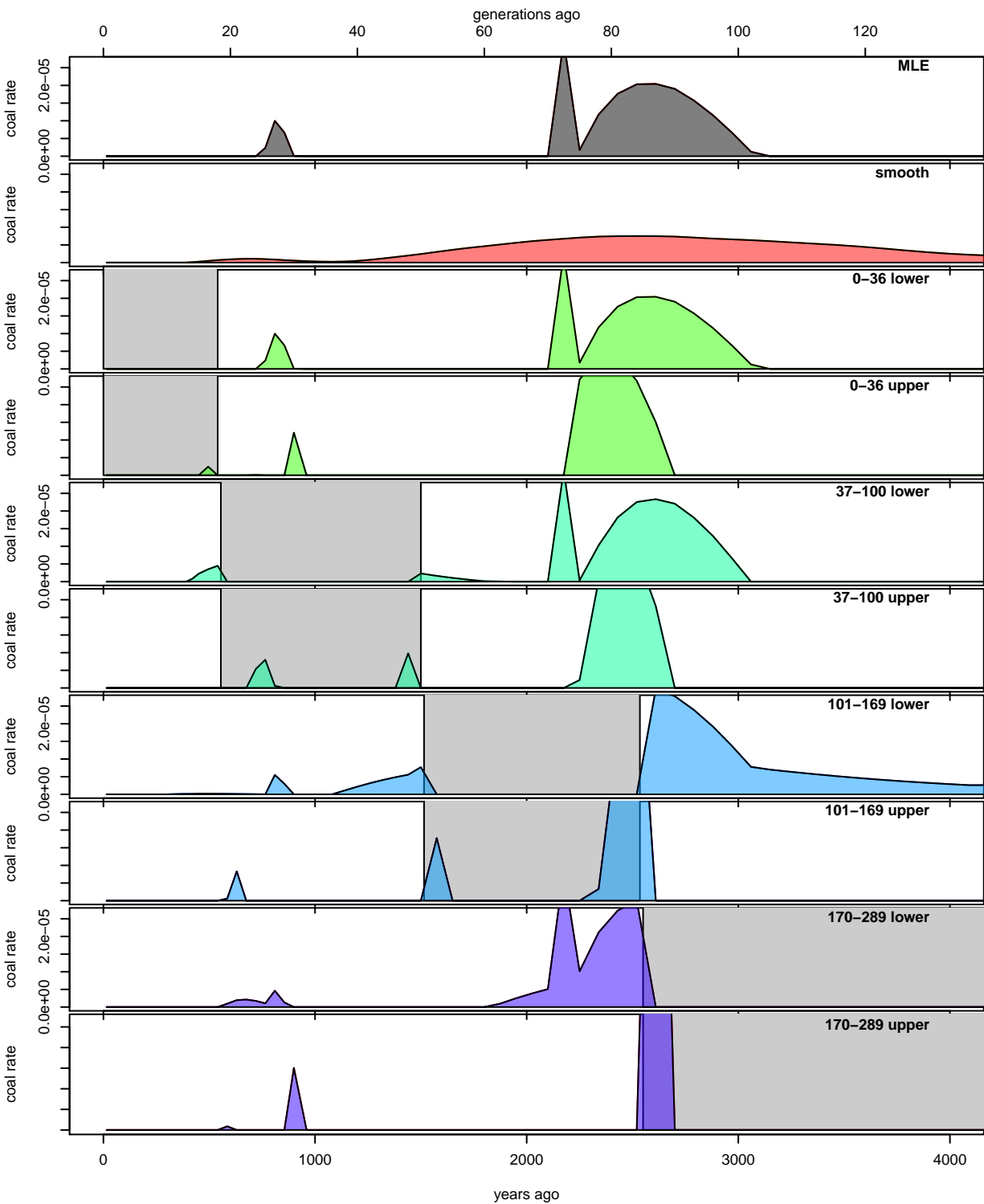

Supplement: Figure S14 — An example of the set of consistent histories (as coalescent distributions μ(n)) used to find upper and lower bounds in Figures S12 and Figure 5. The example shown is Poland–Germany, “MLE” is the maximum likelihood history, “smooth” is the smoothest consistent history, and the remaining plots show the histories giving lower and upper bounds for the referenced time intervals (in numbers of generations). In each case, the segment of time on which we are looking for a bound is shaded. (PDF) [file pbio.1001555.s014.pdf]

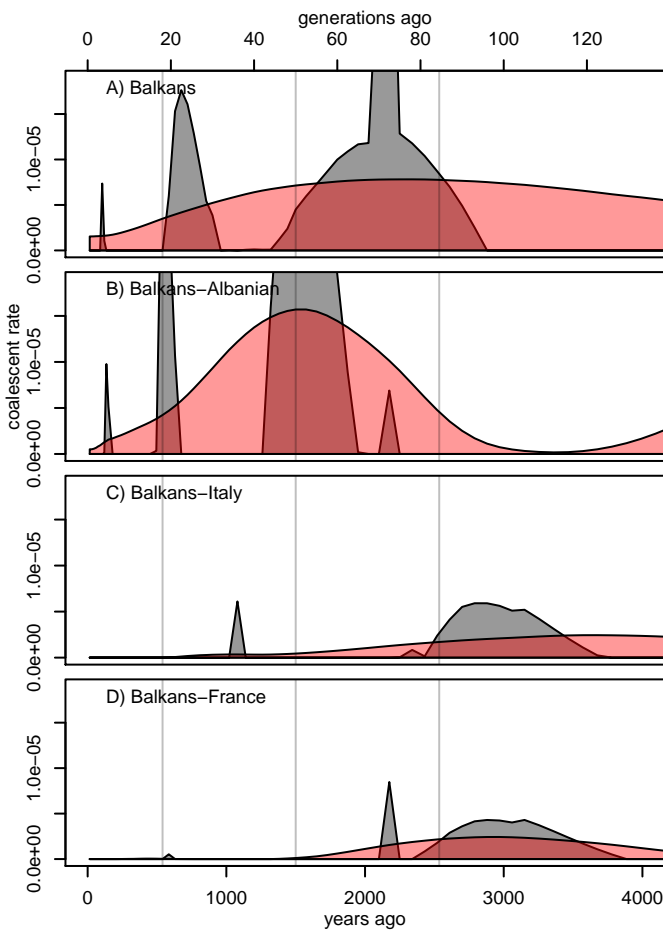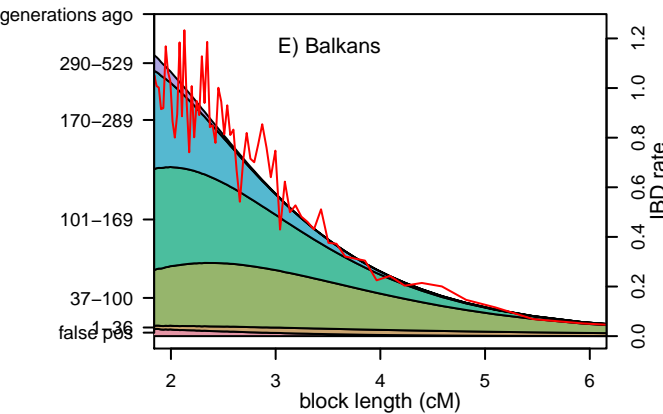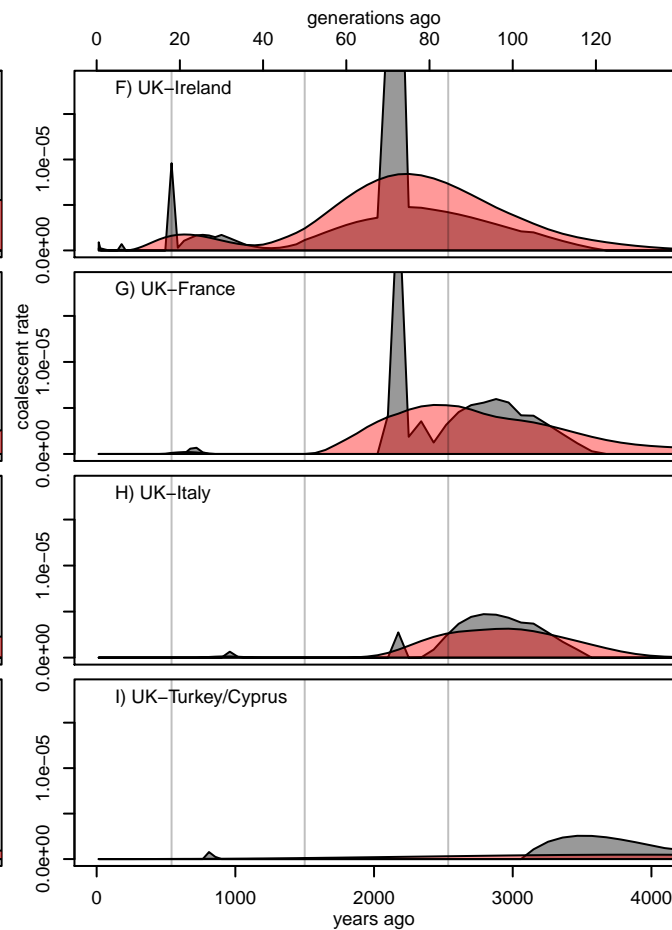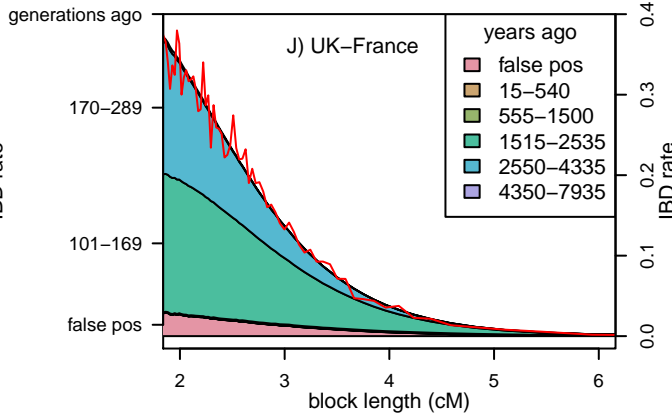

Supplement: Figure S15 — For those who are used to thinking in effective population sizes, the equivalent figure to Figure 4, except with coalescent rate on the vertical axis, rather than numbers of most recent genetic common ancestors. (PDF) [file pbio.1001555.s015.pdf]

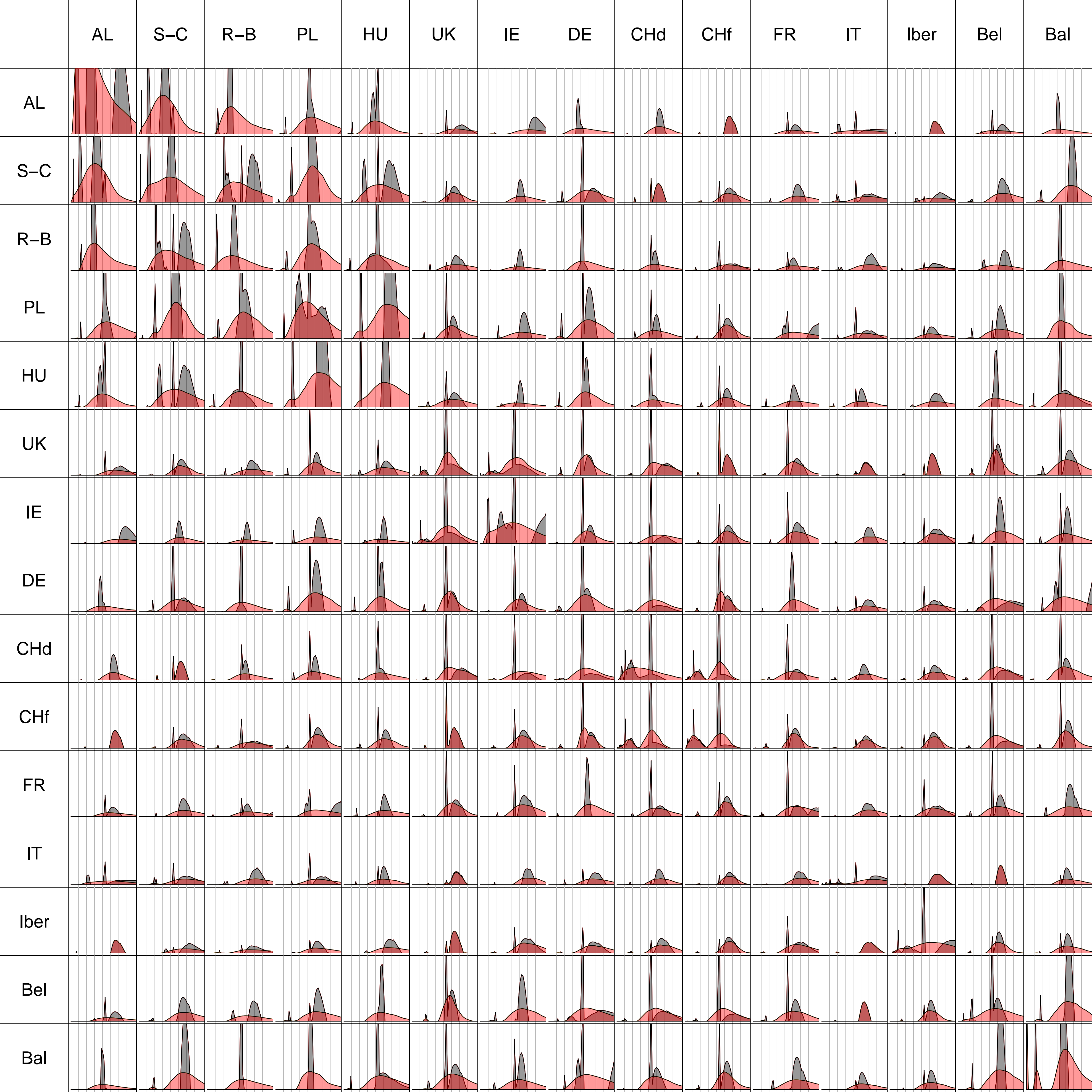

Supplement: Figure S16 — The maximum likelihood history (grey) and smoothest consistent history (red) for all pairs of population groupings of Figure S12 (including those of Figure 5). Each panel is analogous to a panel of Figure 4; time scale is given by vertical grey lines every 500 years. For these plots on a larger scale, see Figure S17. (PDF) [file pbio.1001555.s016.pdf]

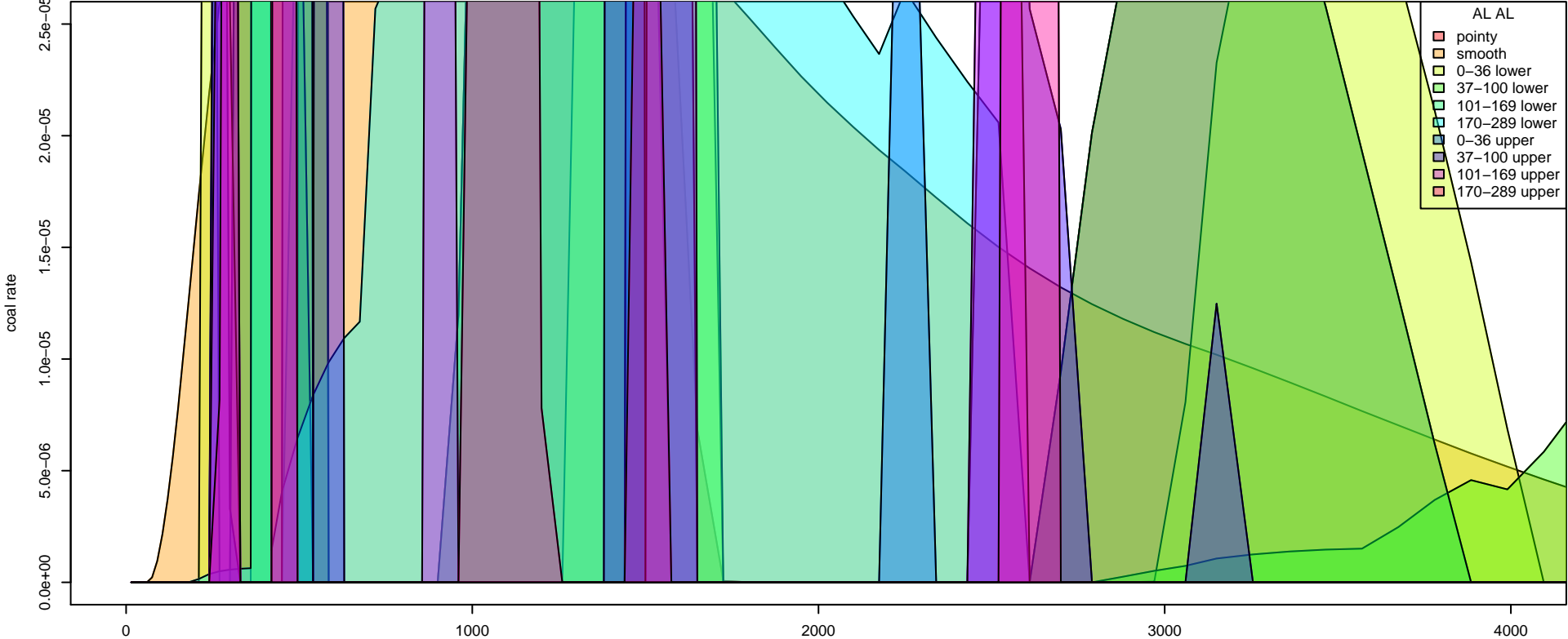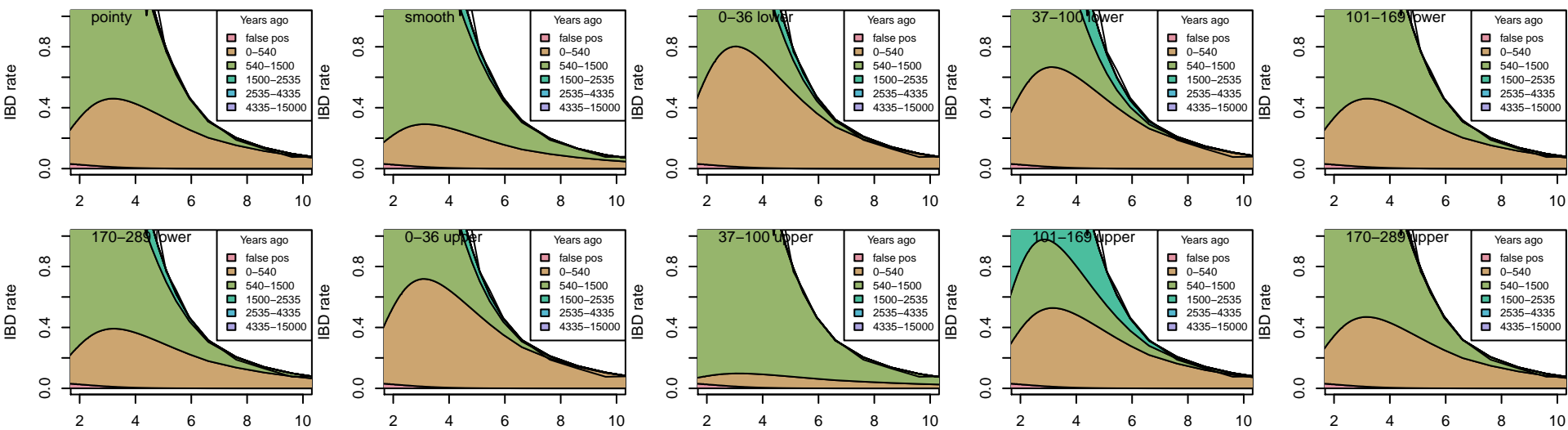

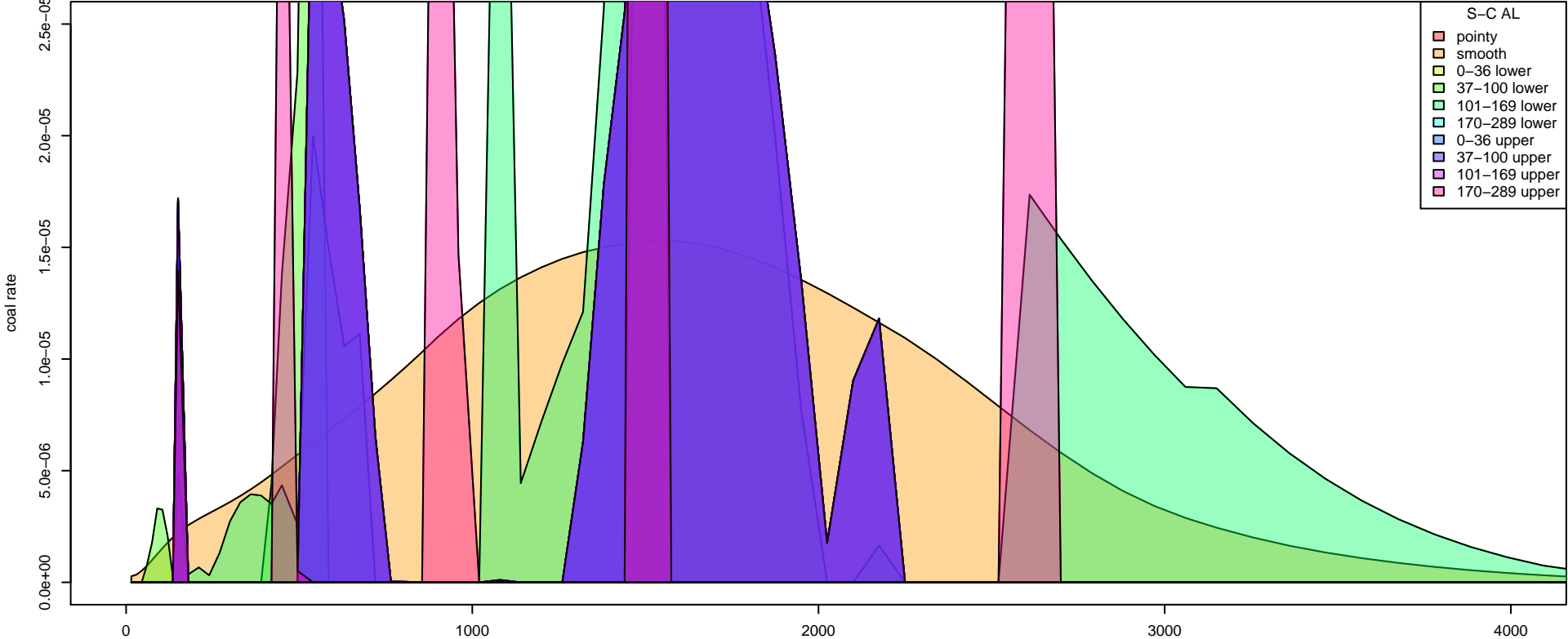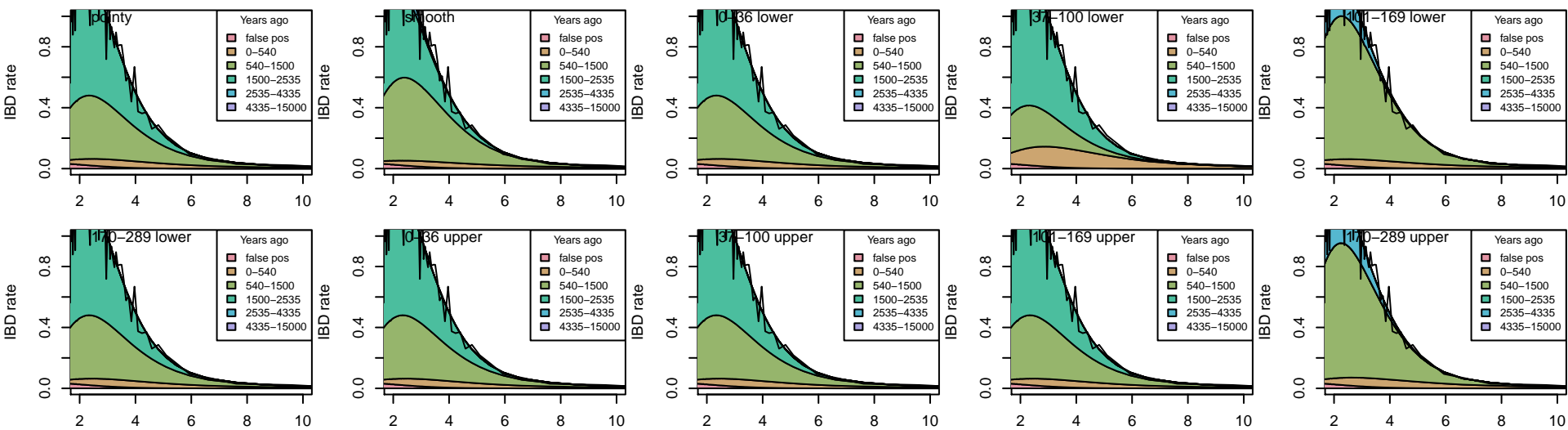

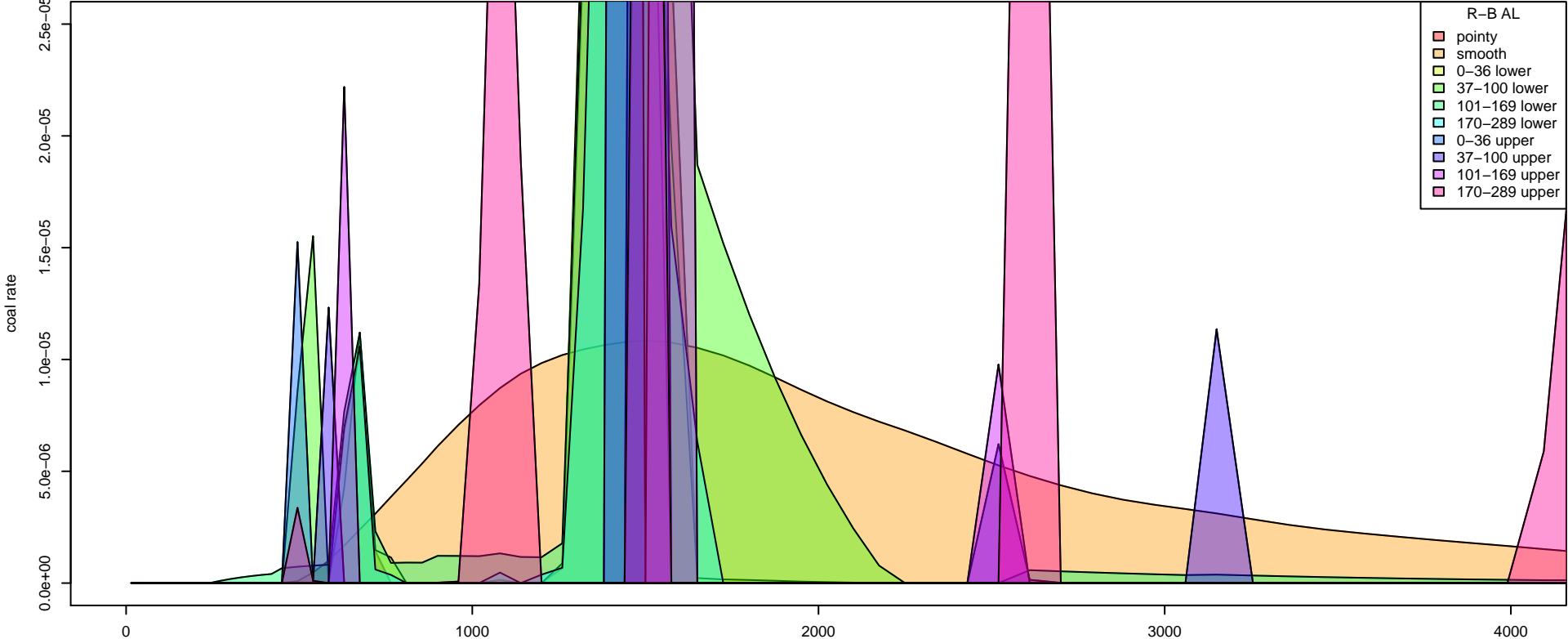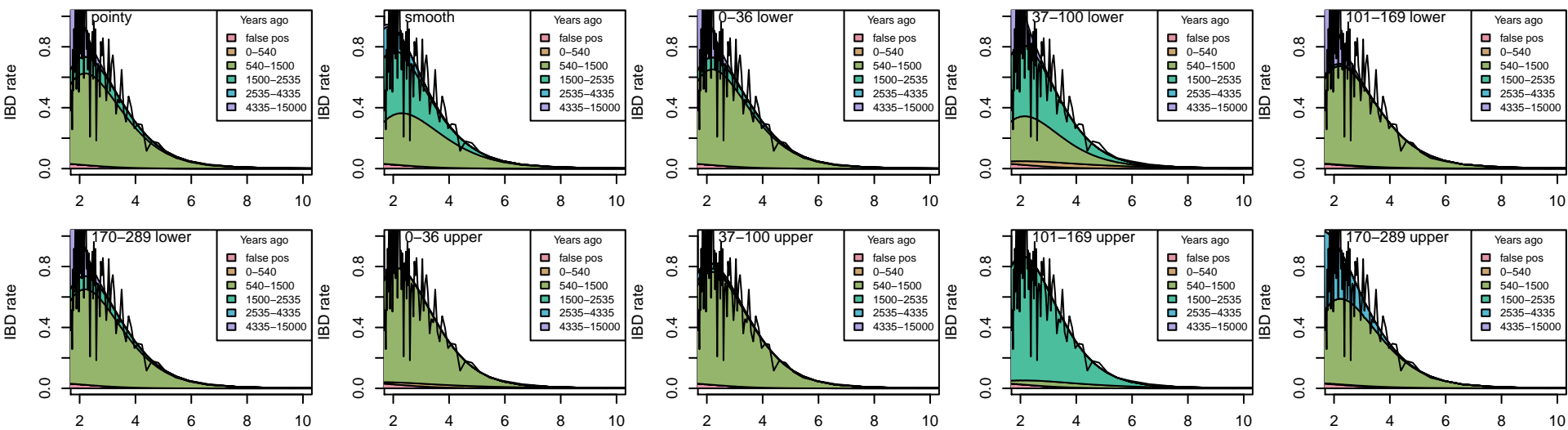

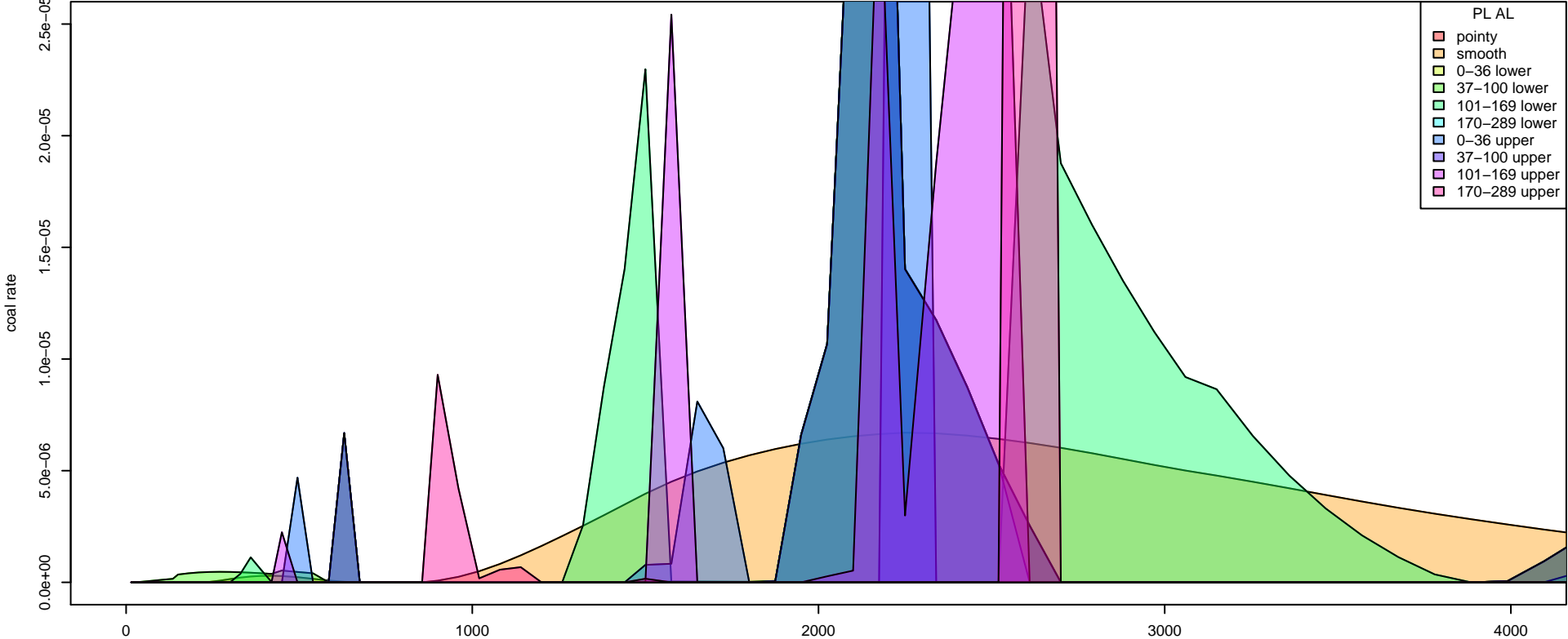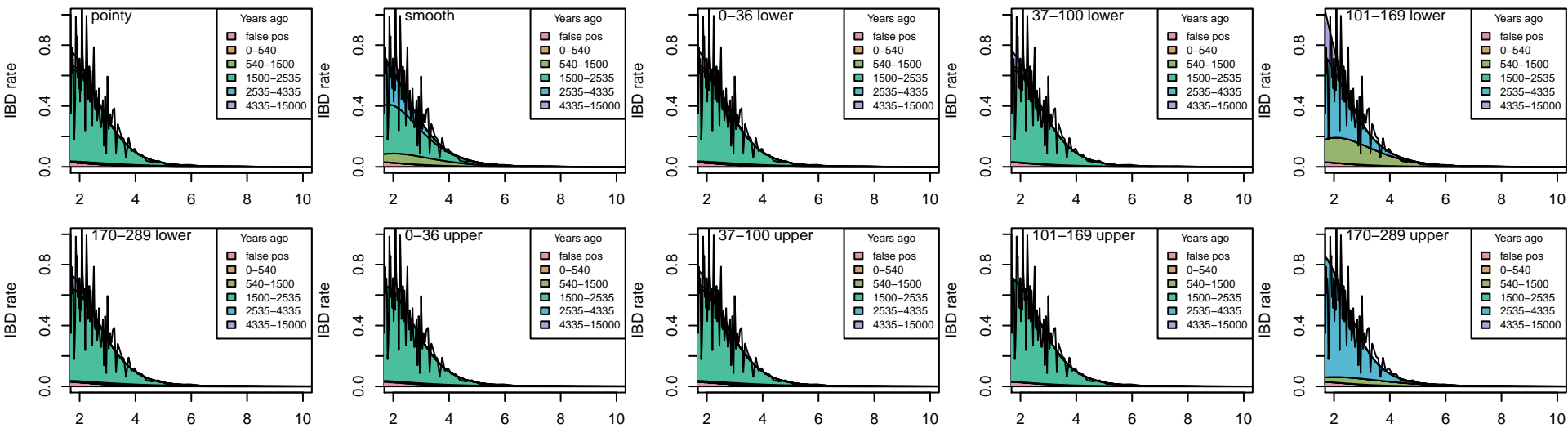

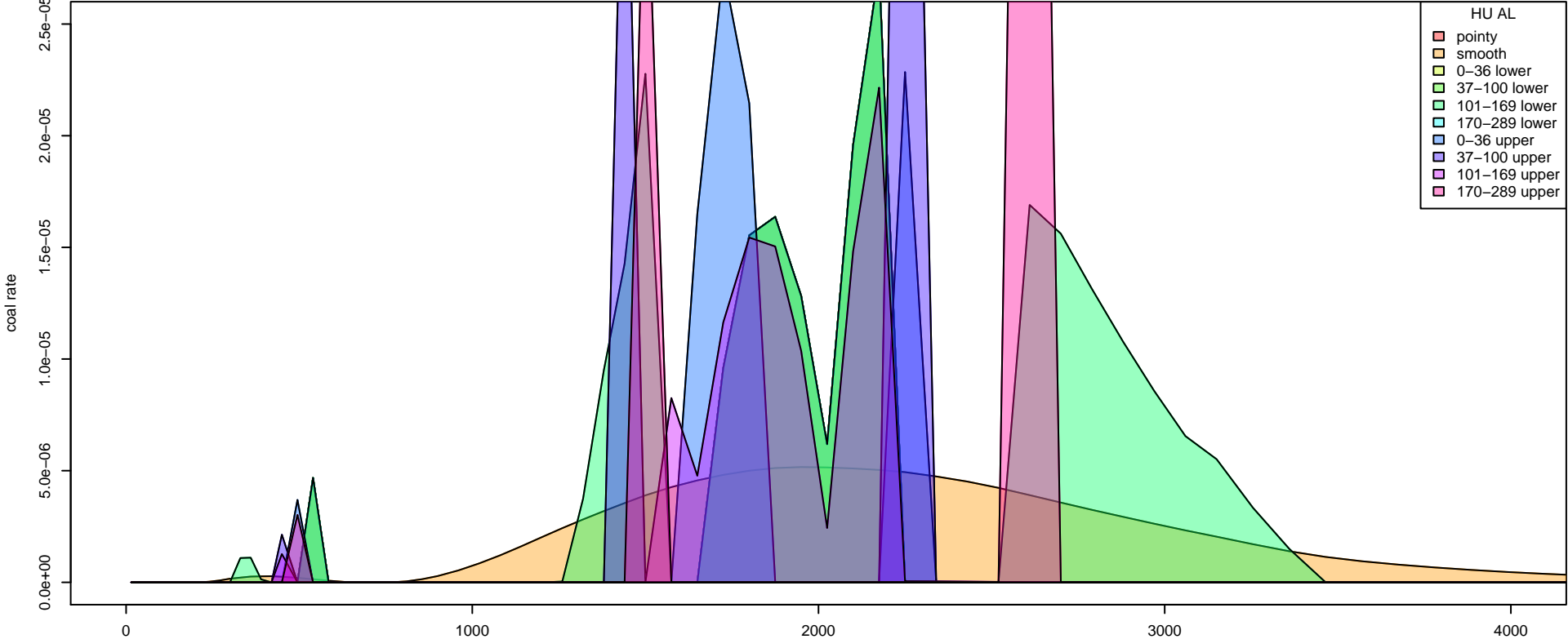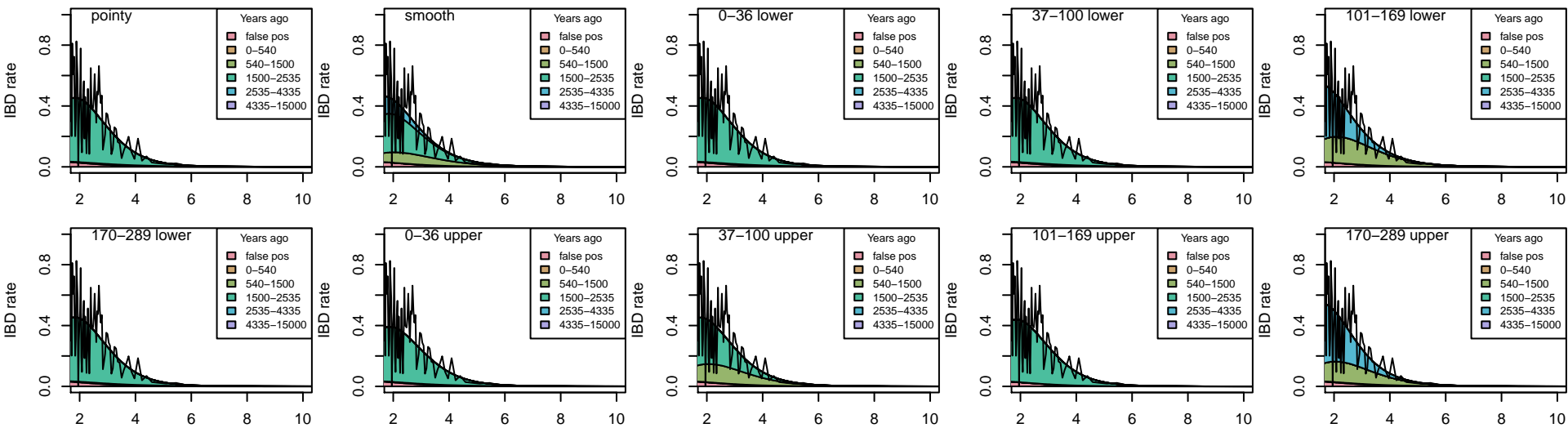

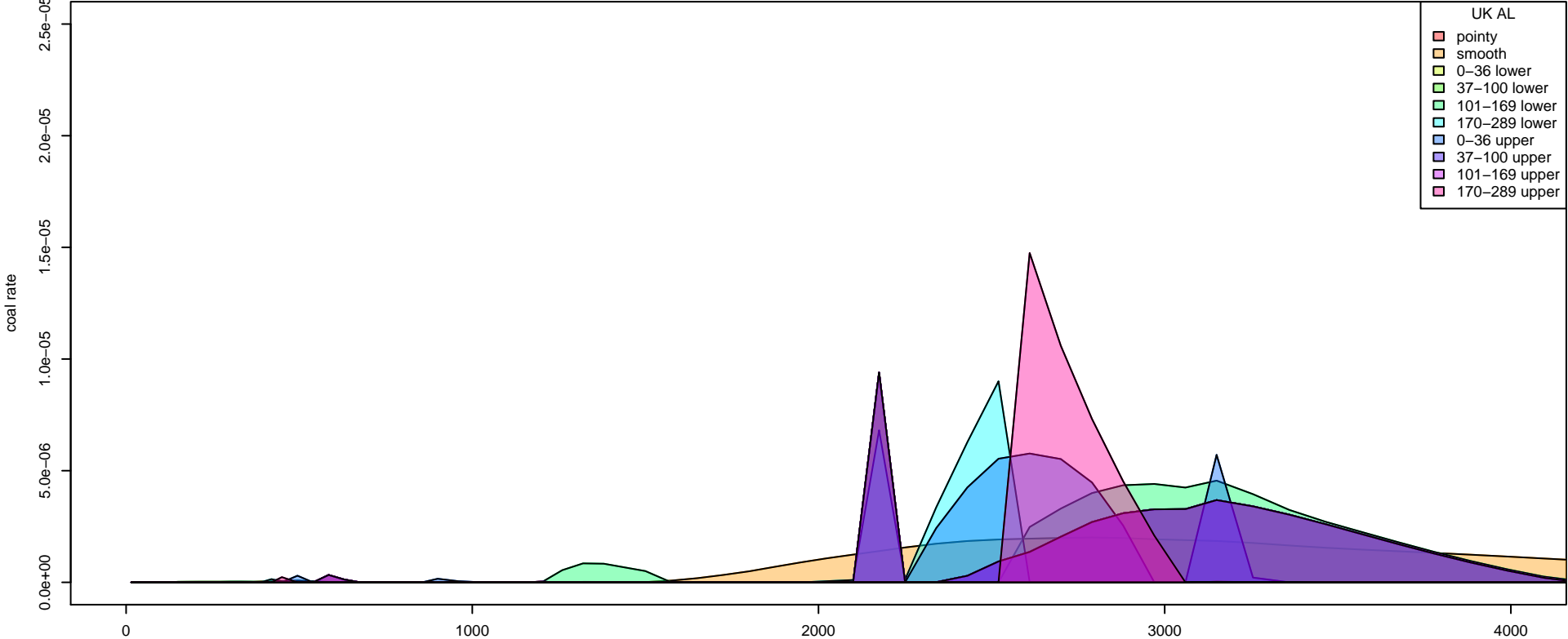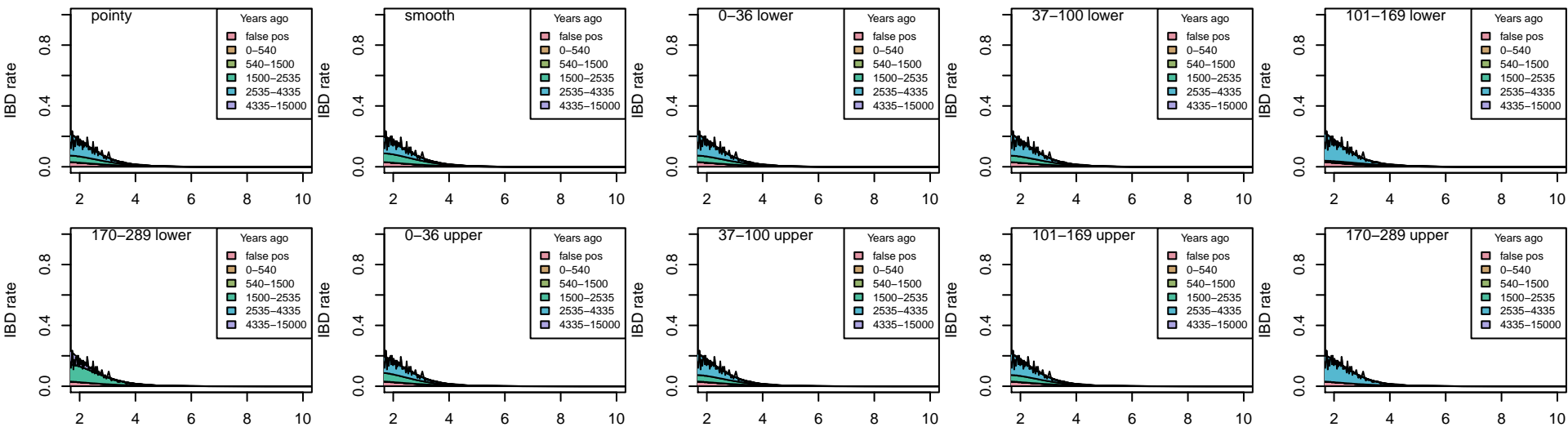

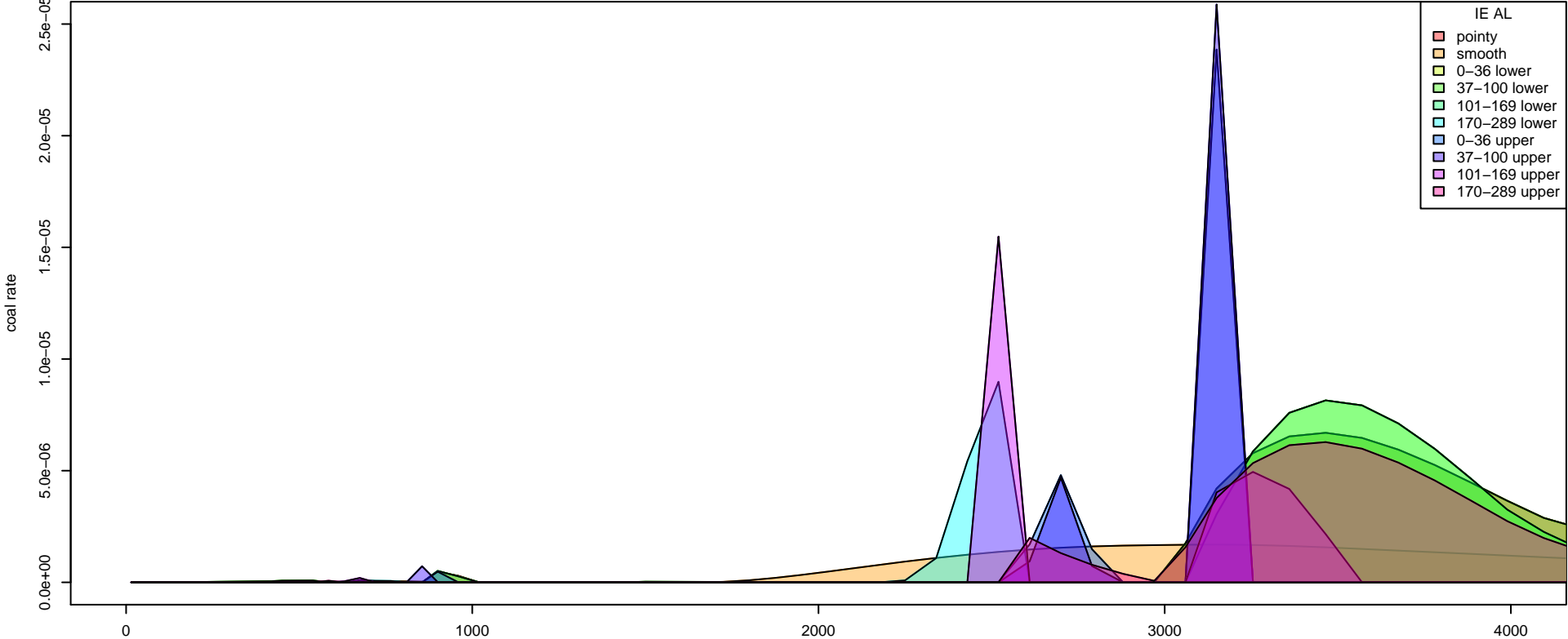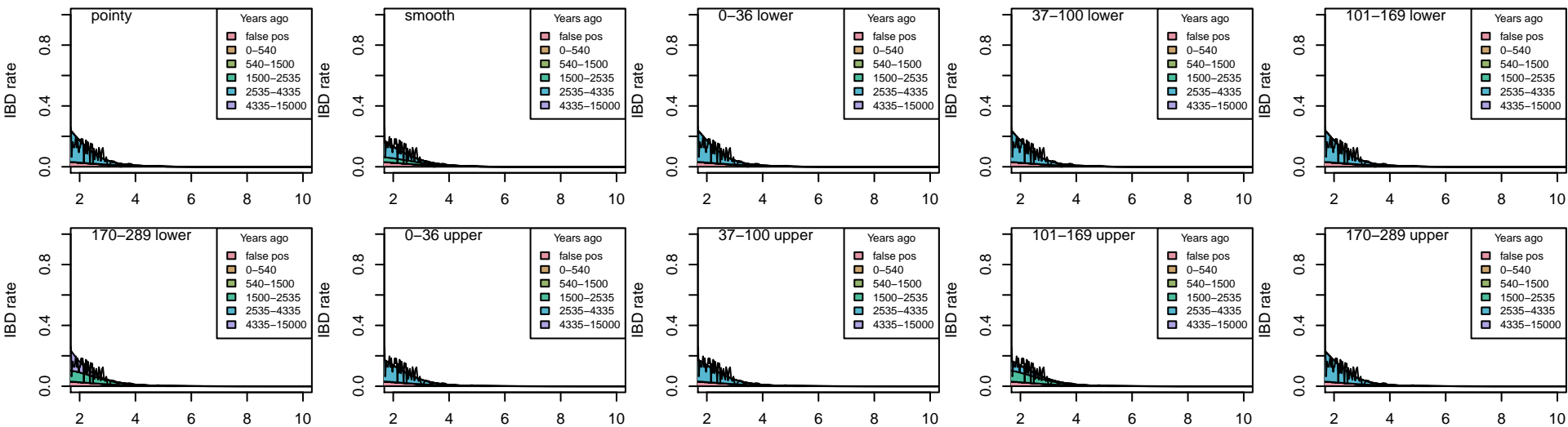

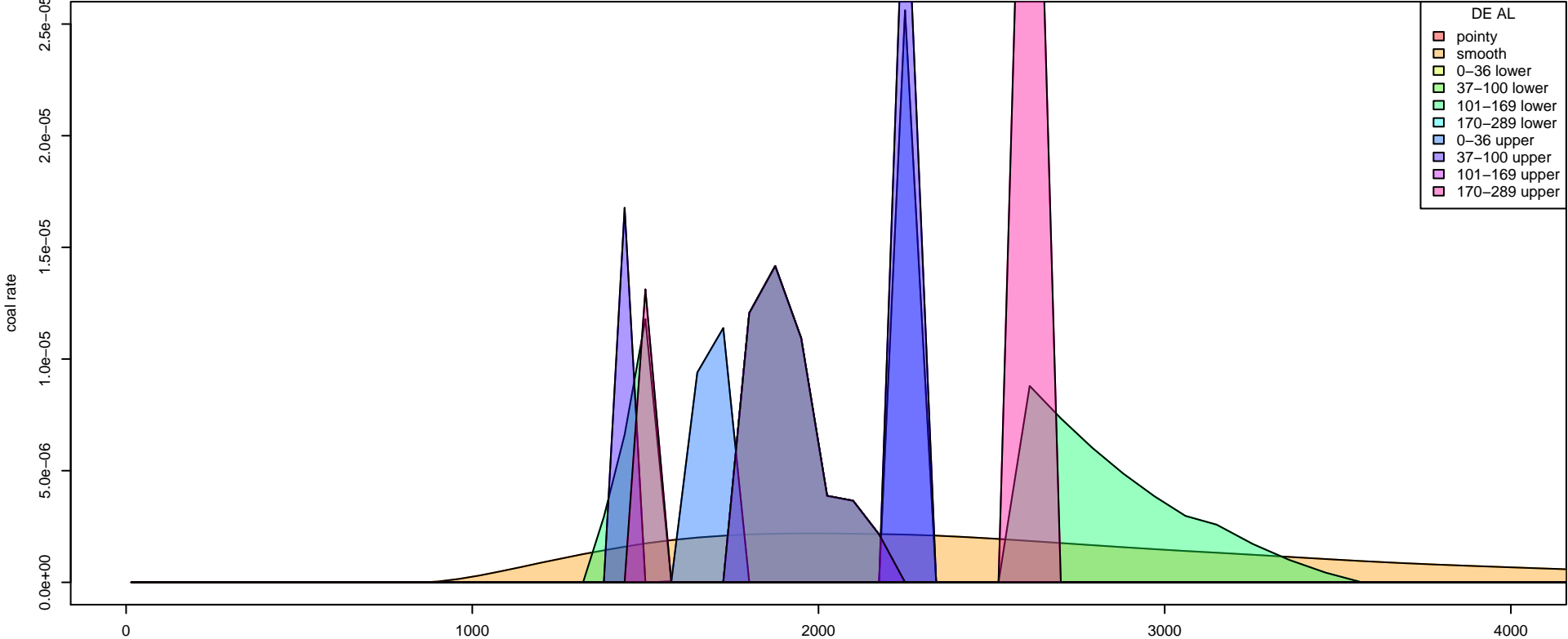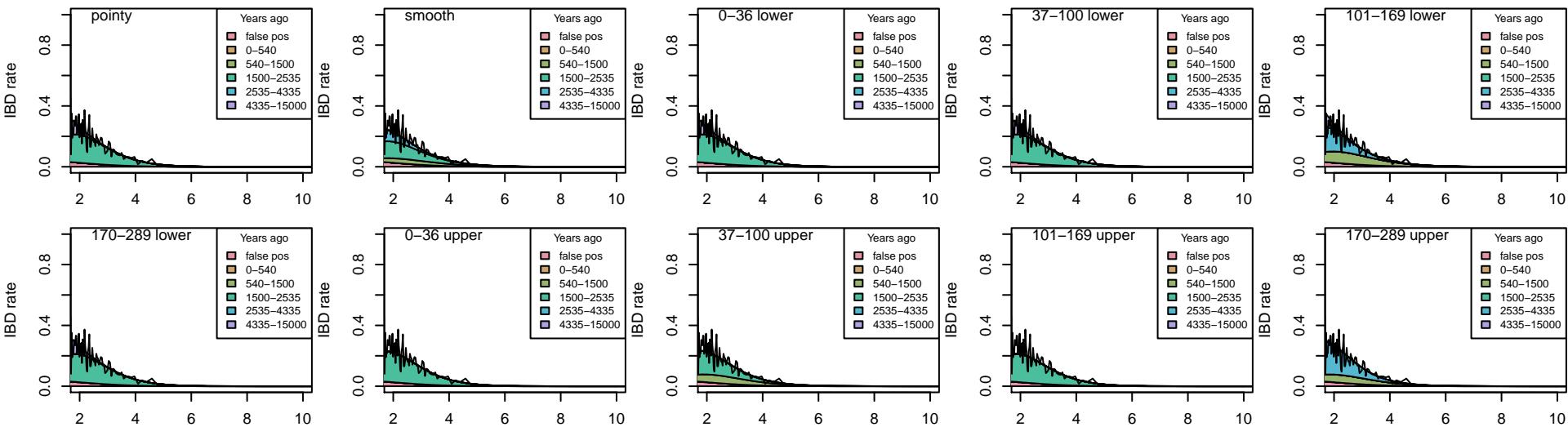

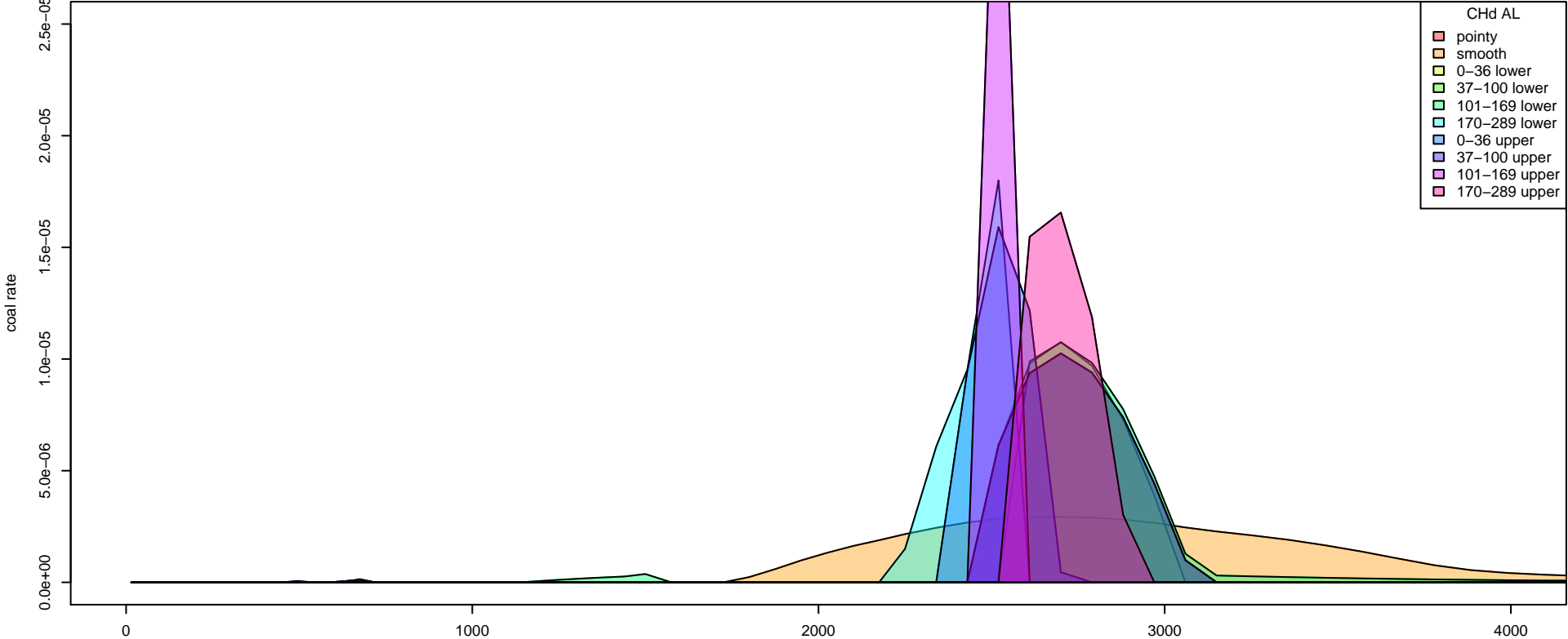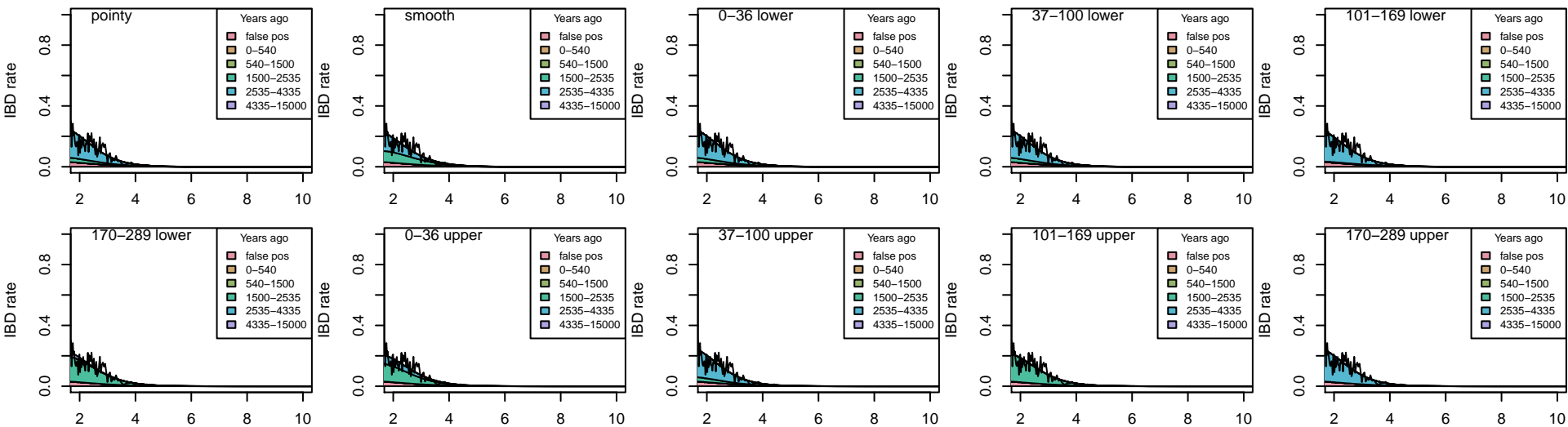

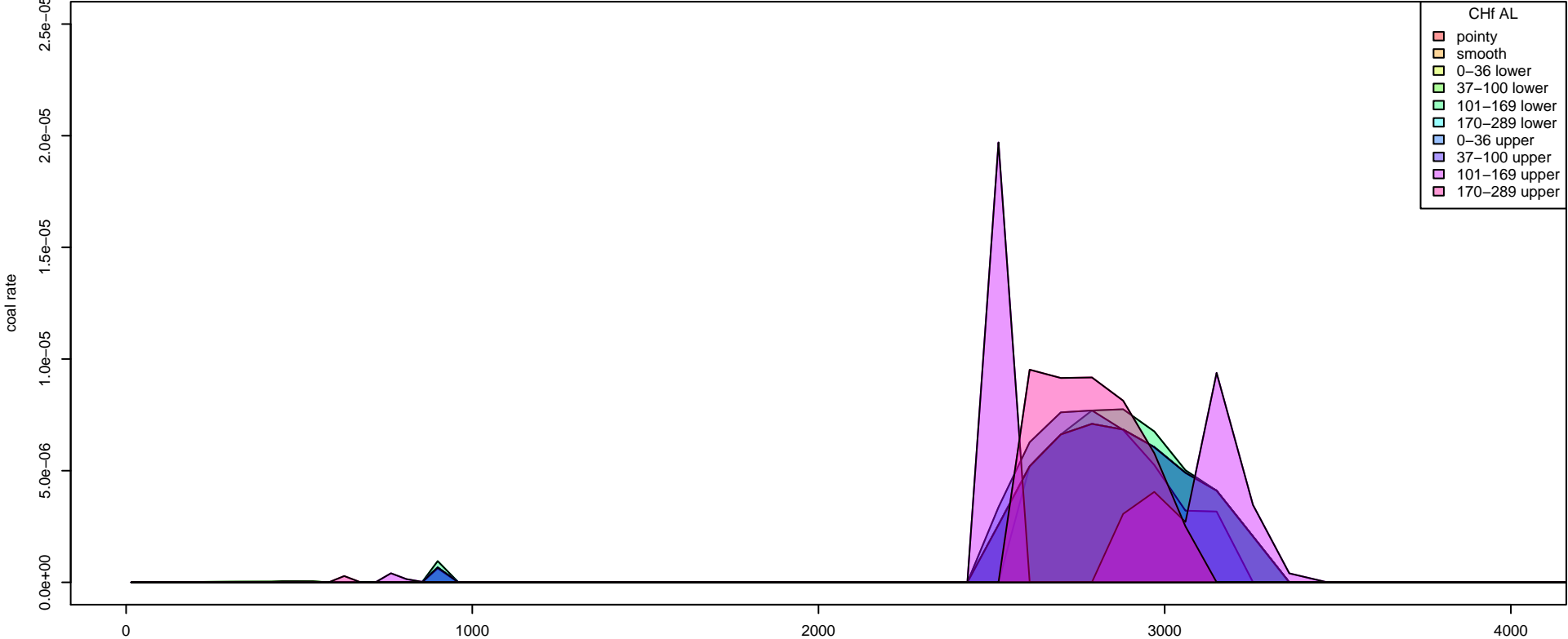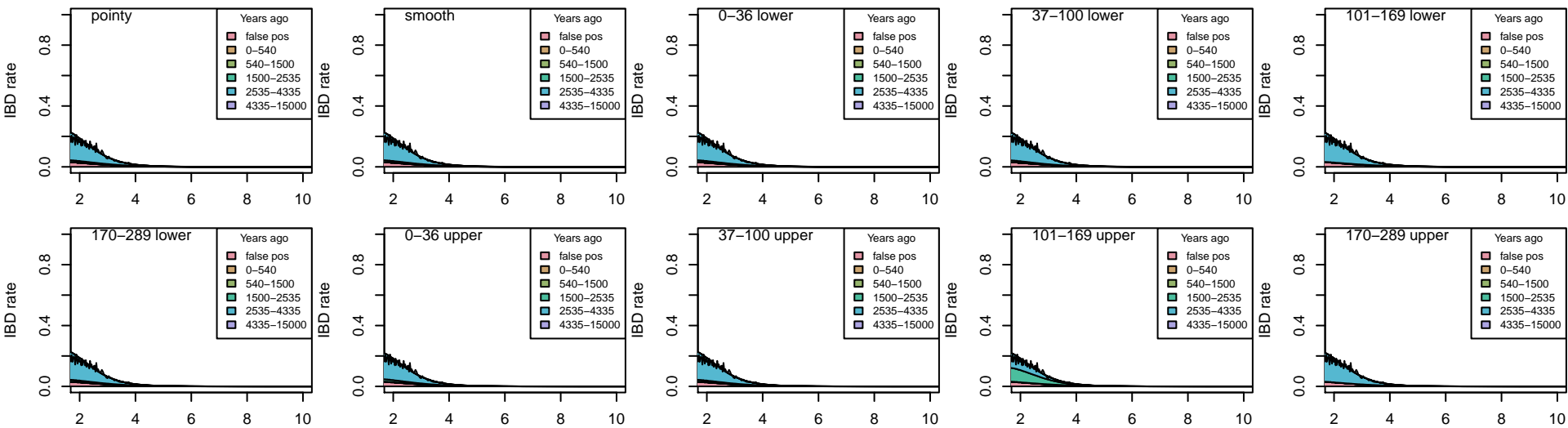

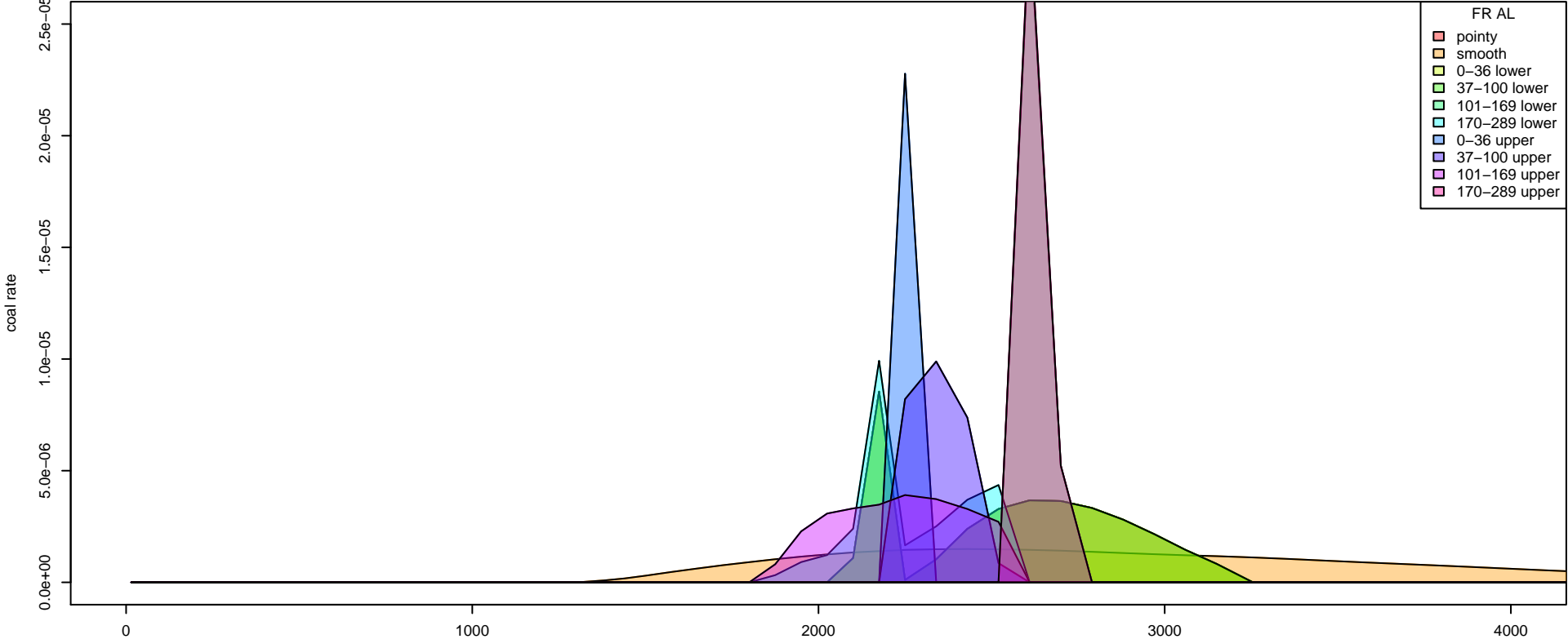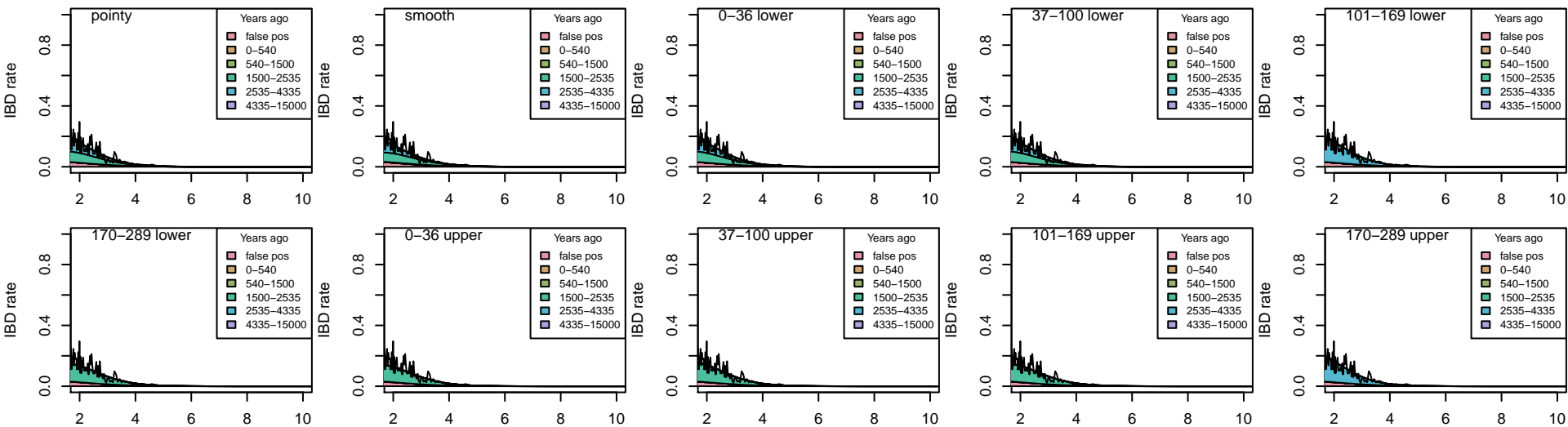

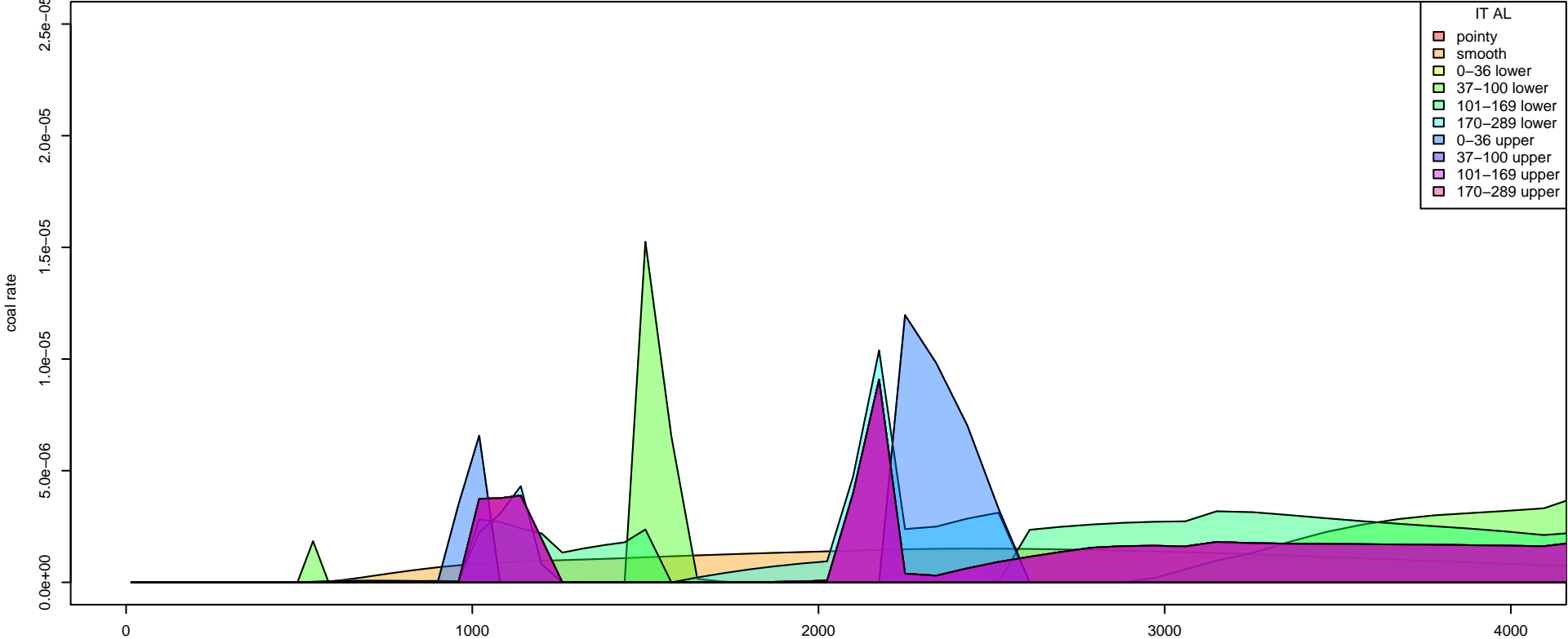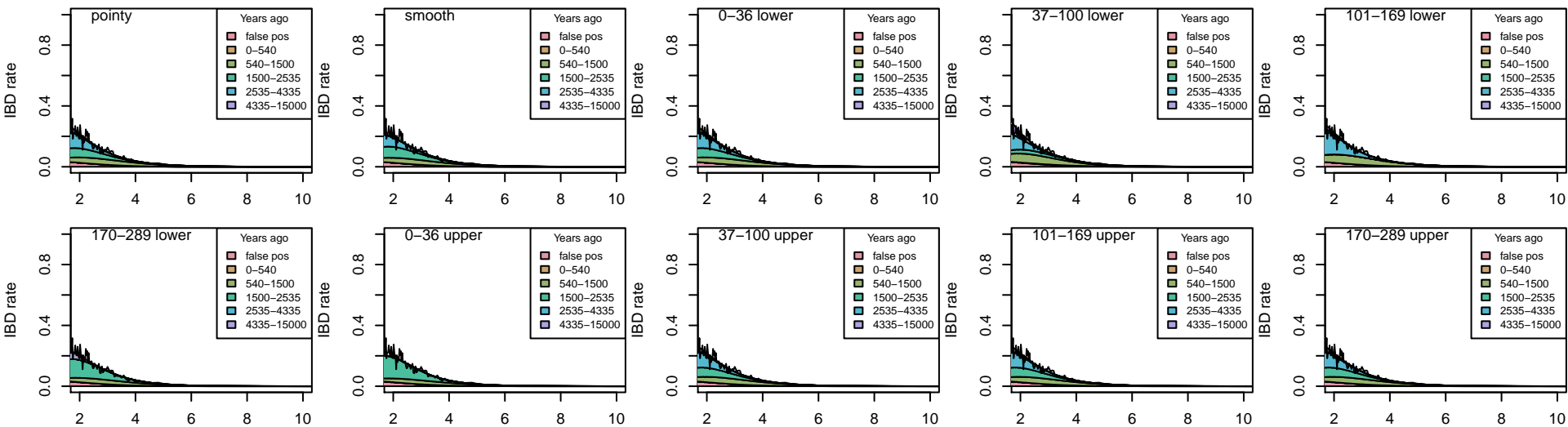

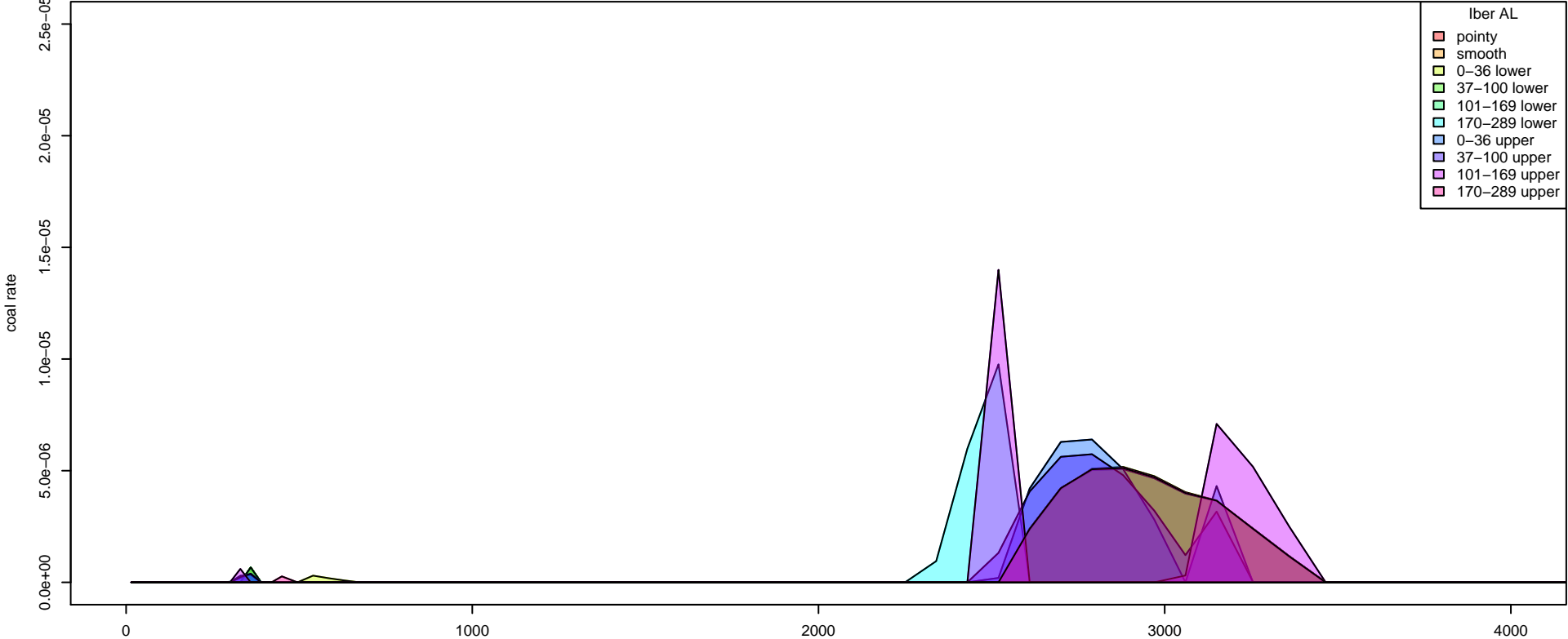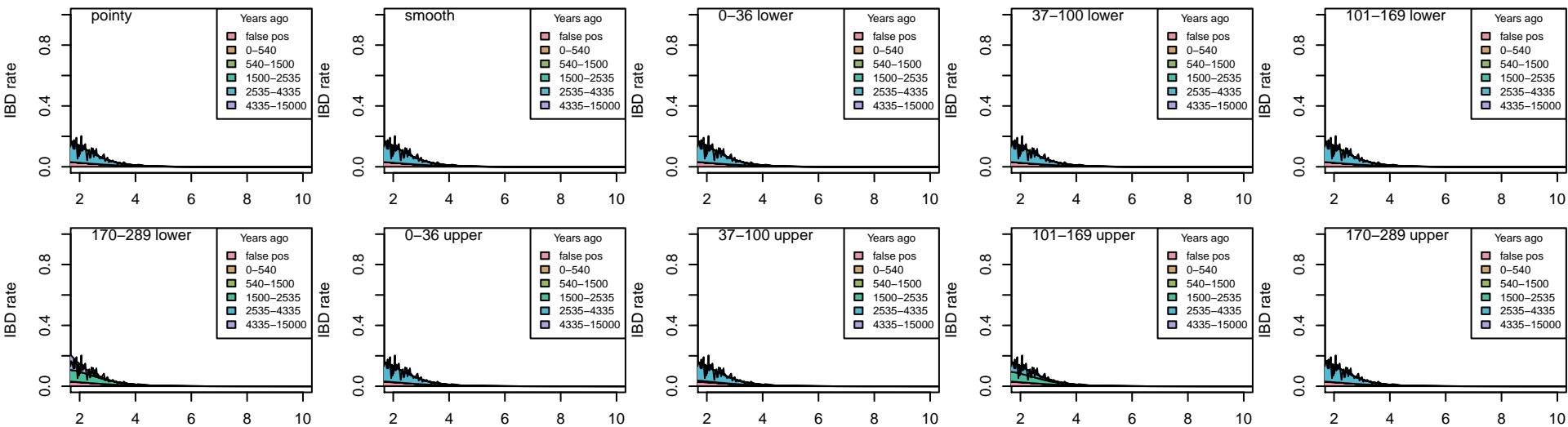

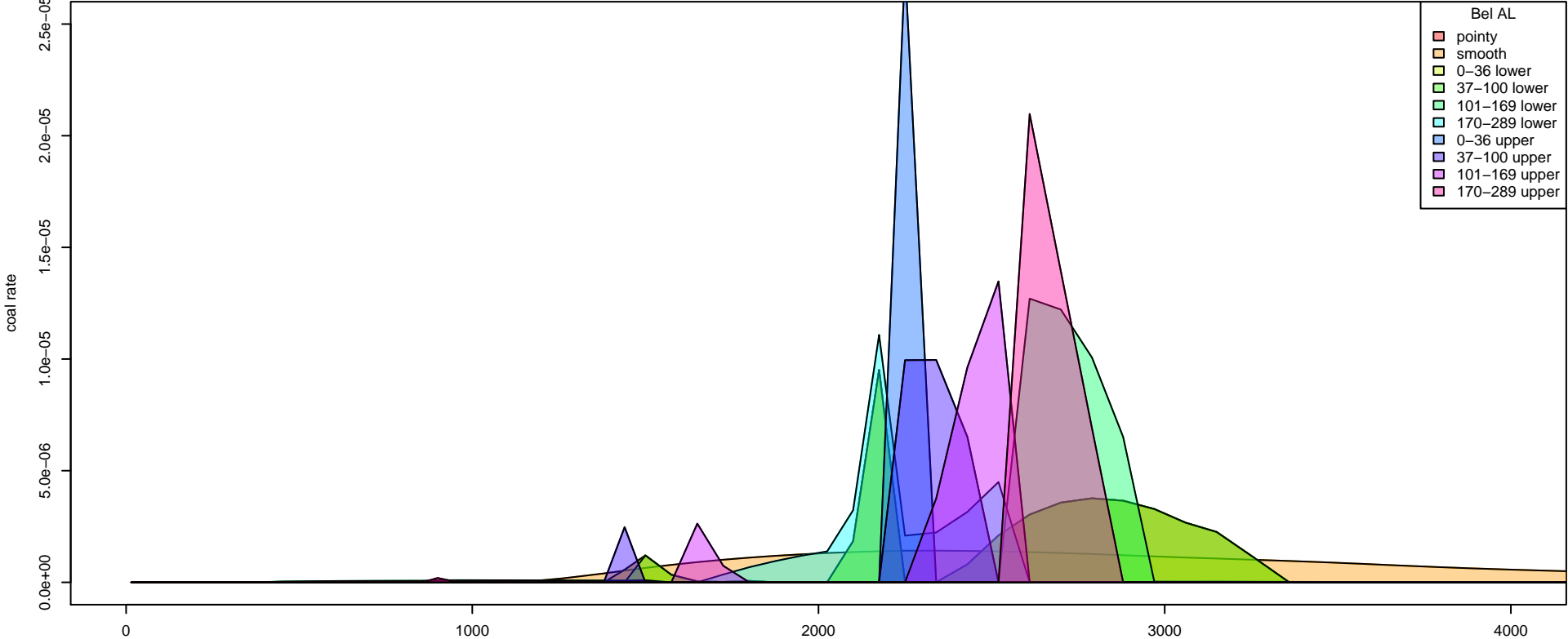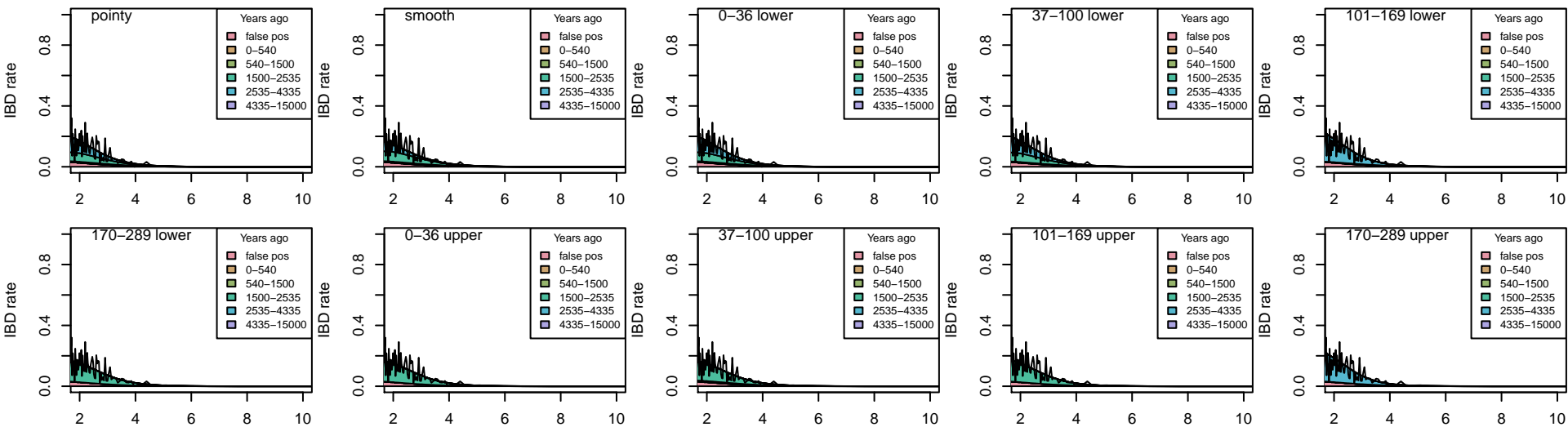

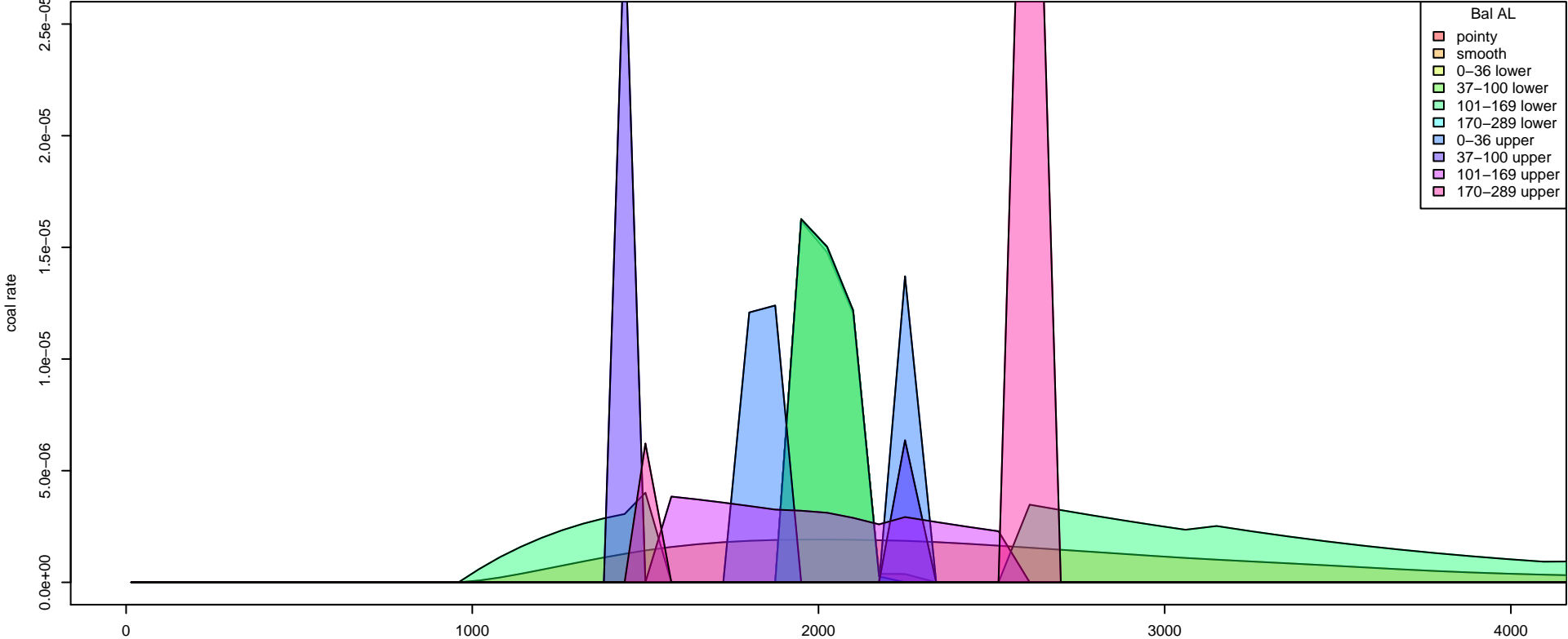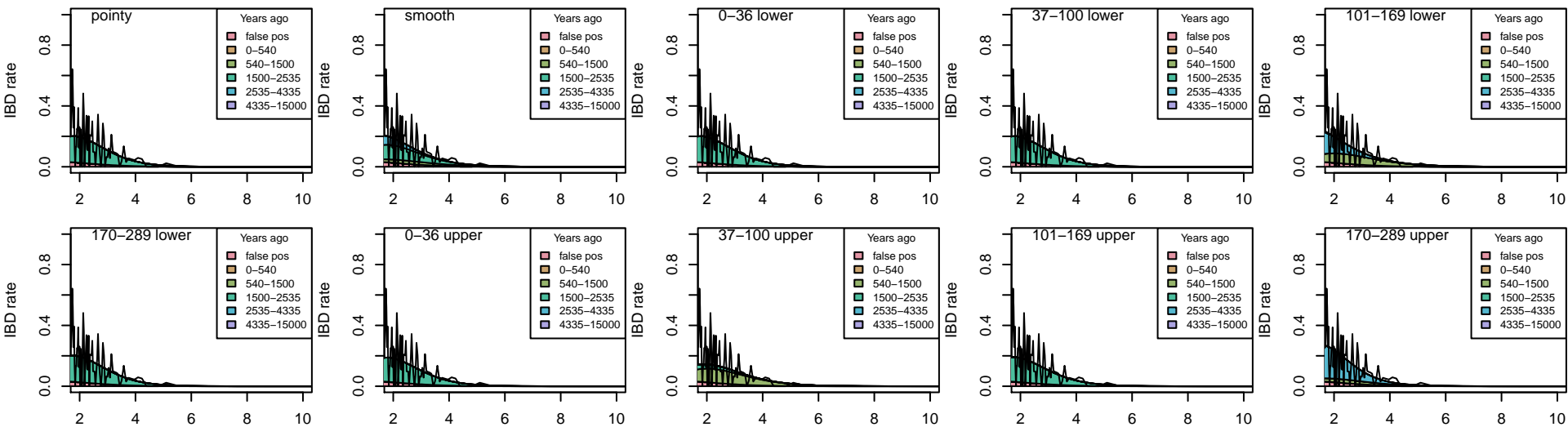

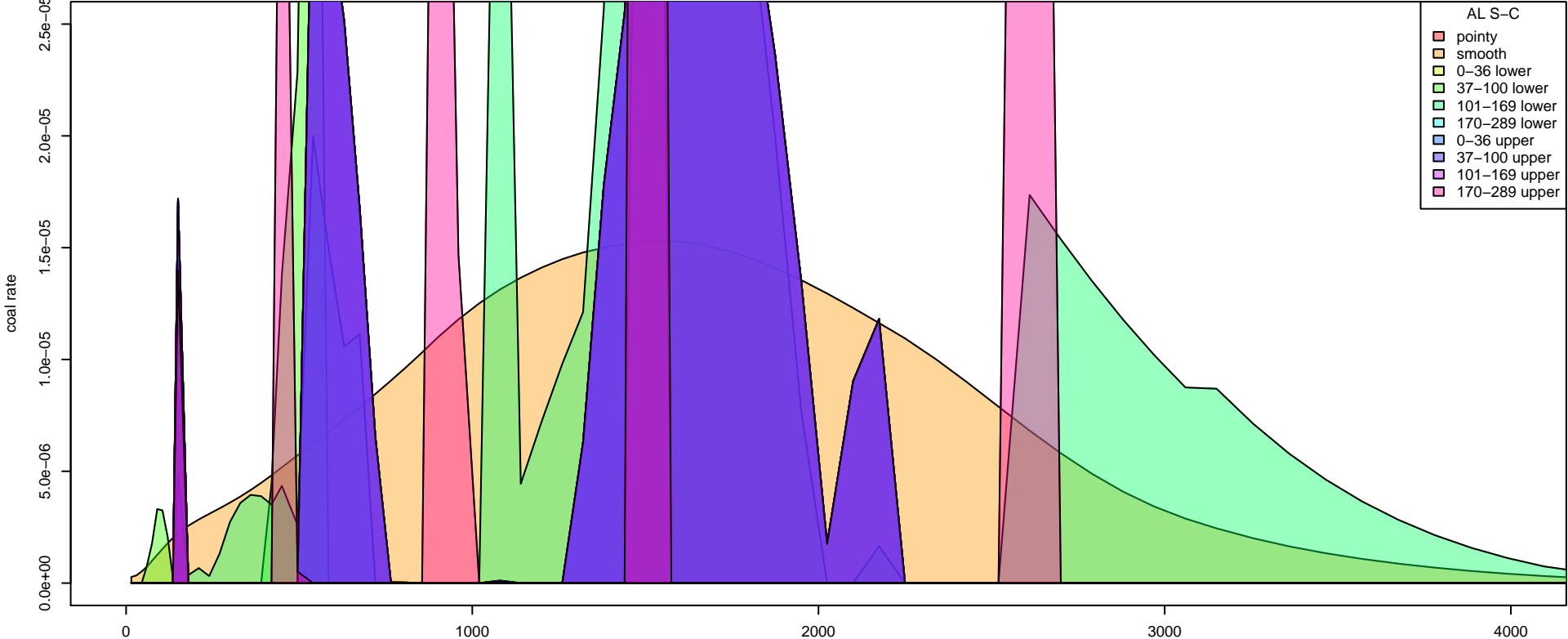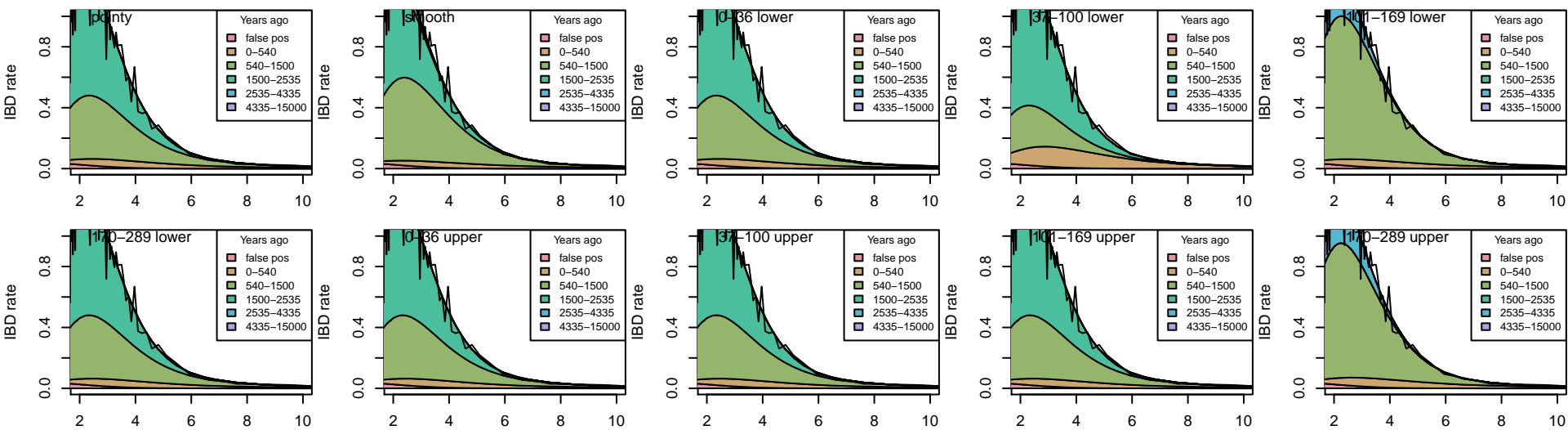

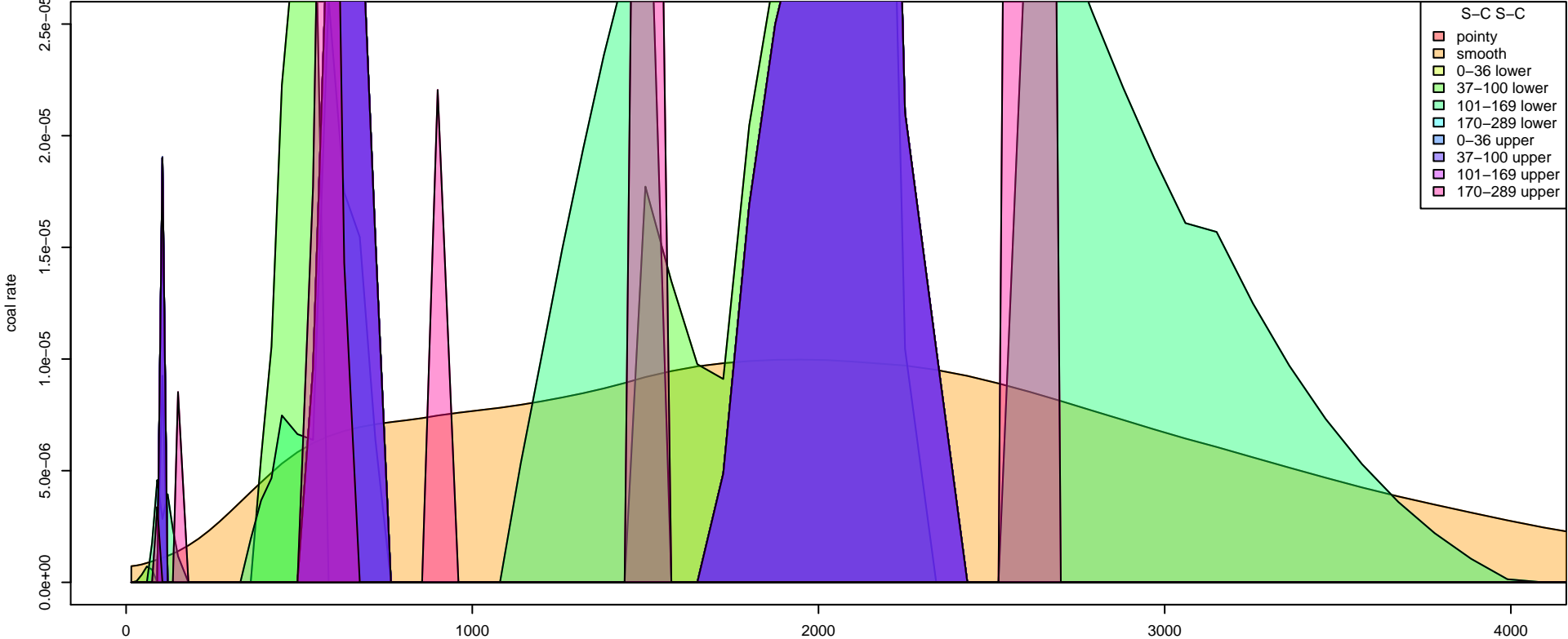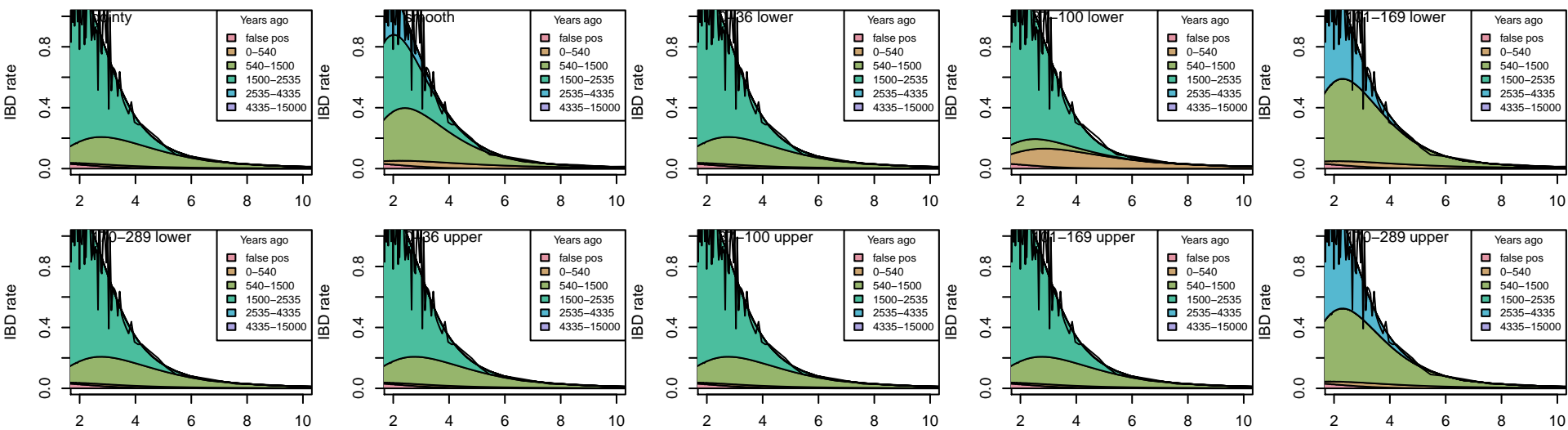

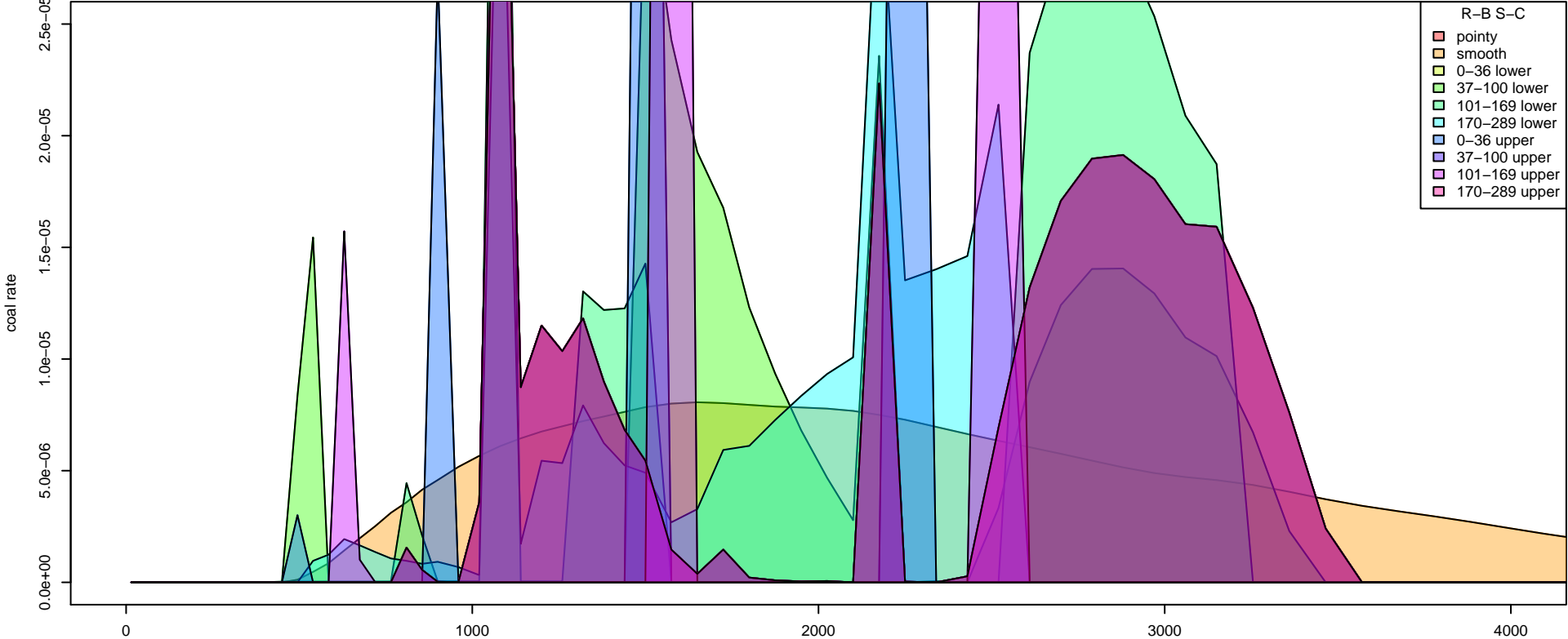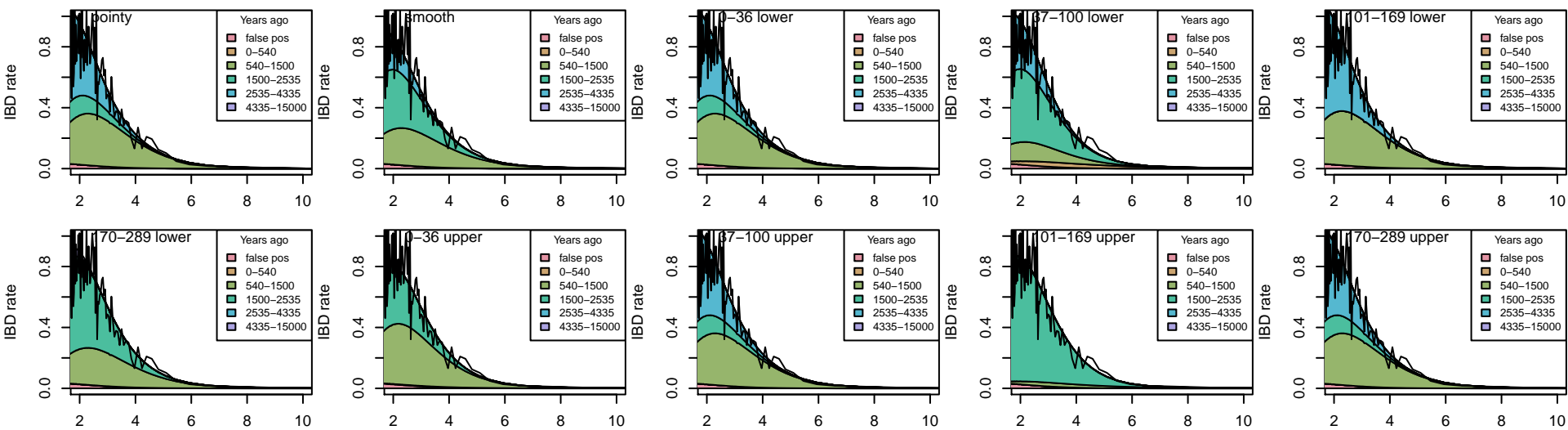

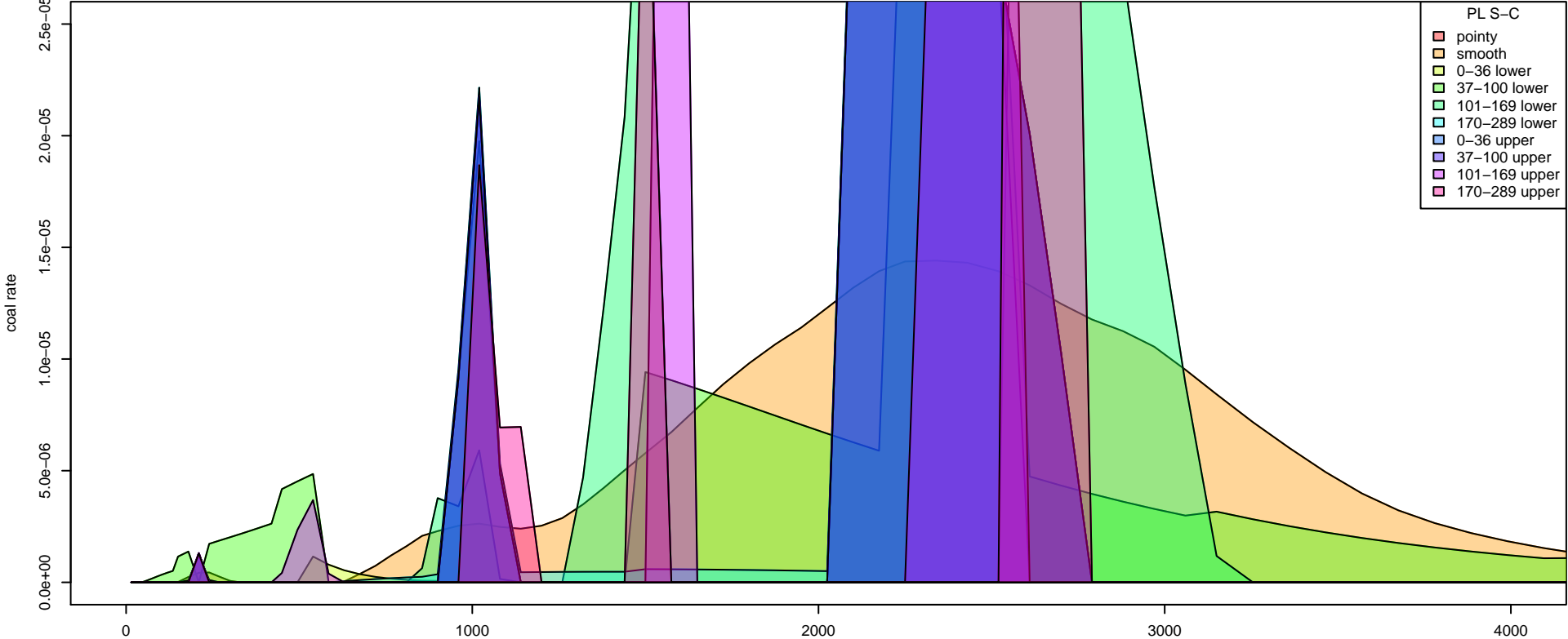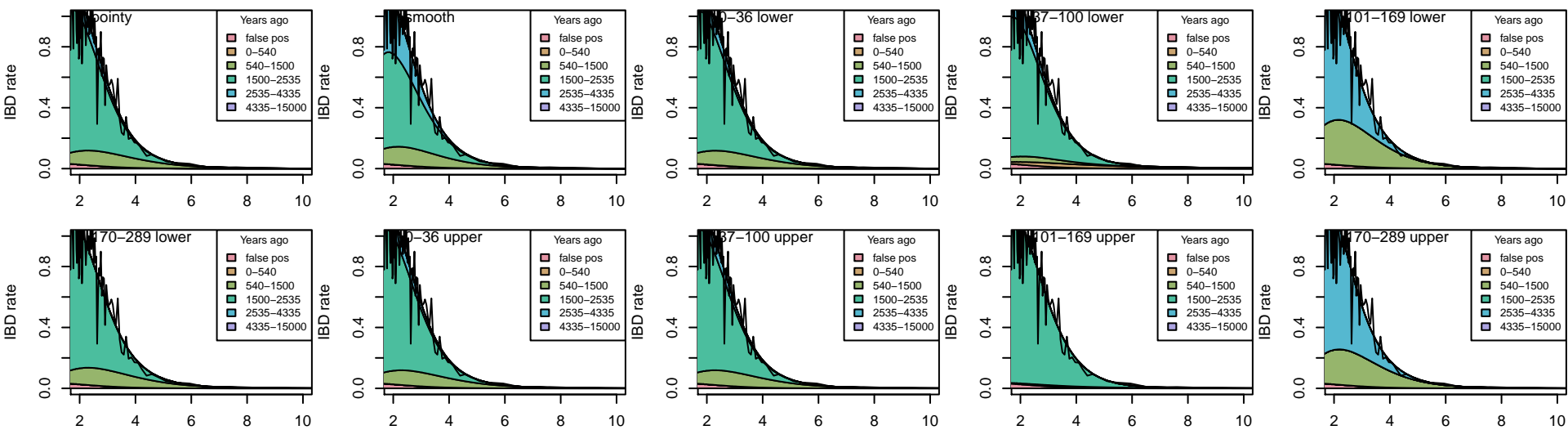

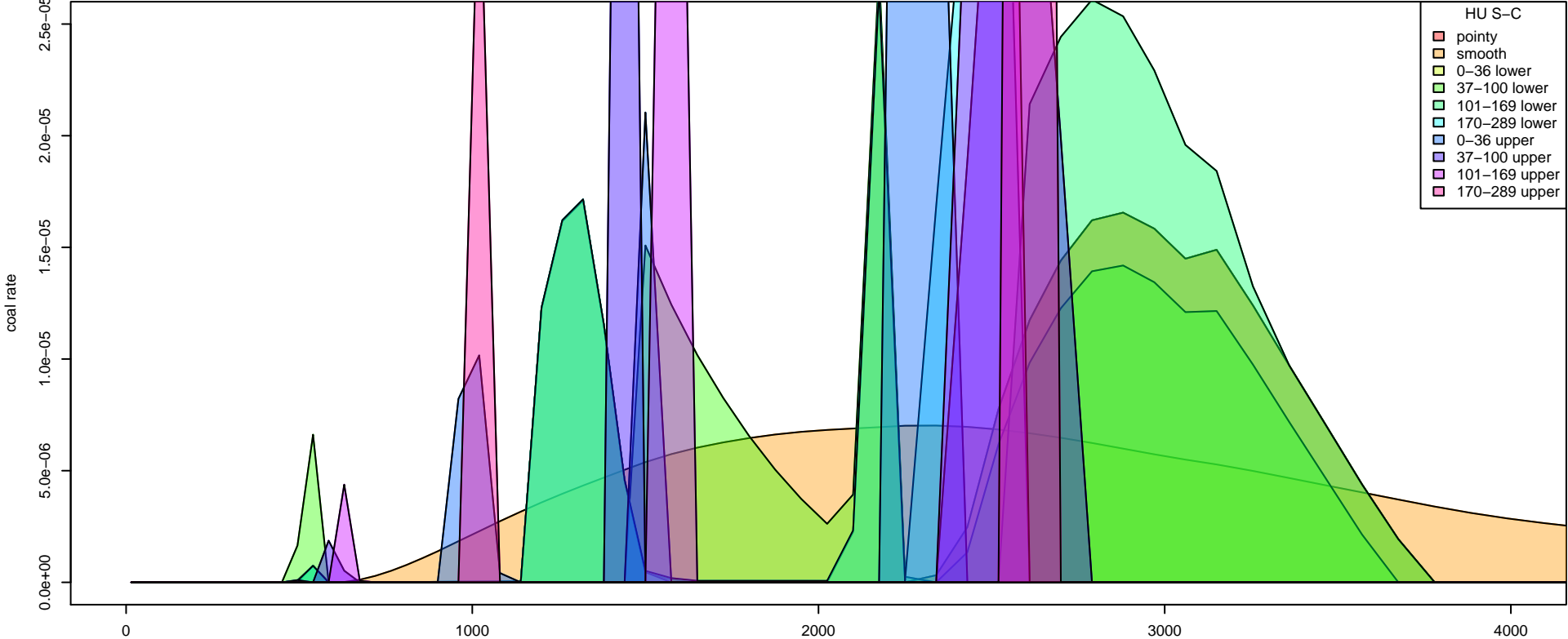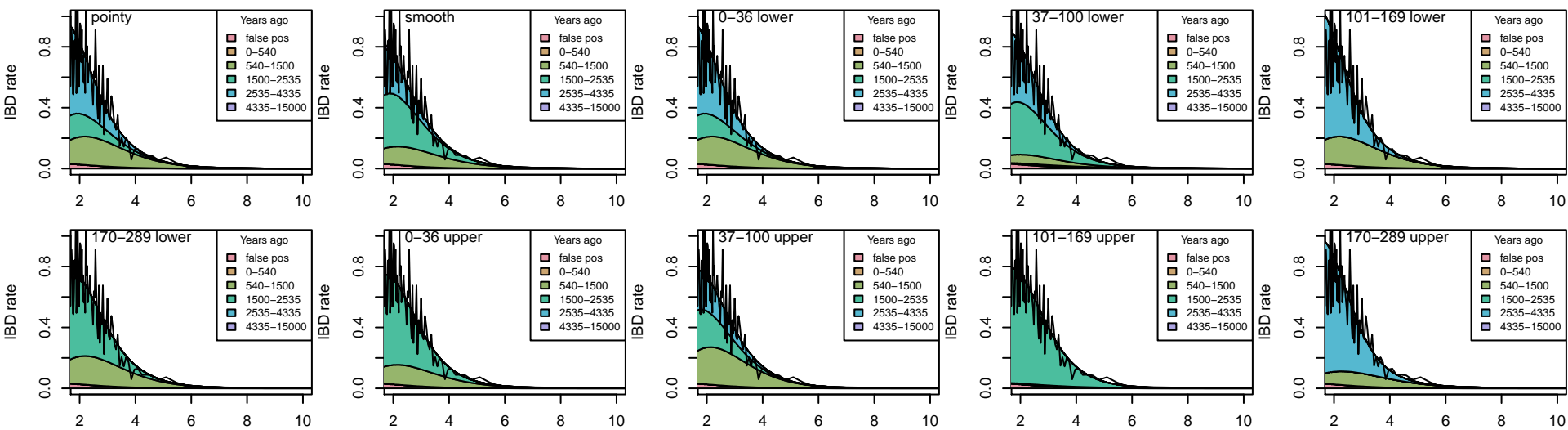

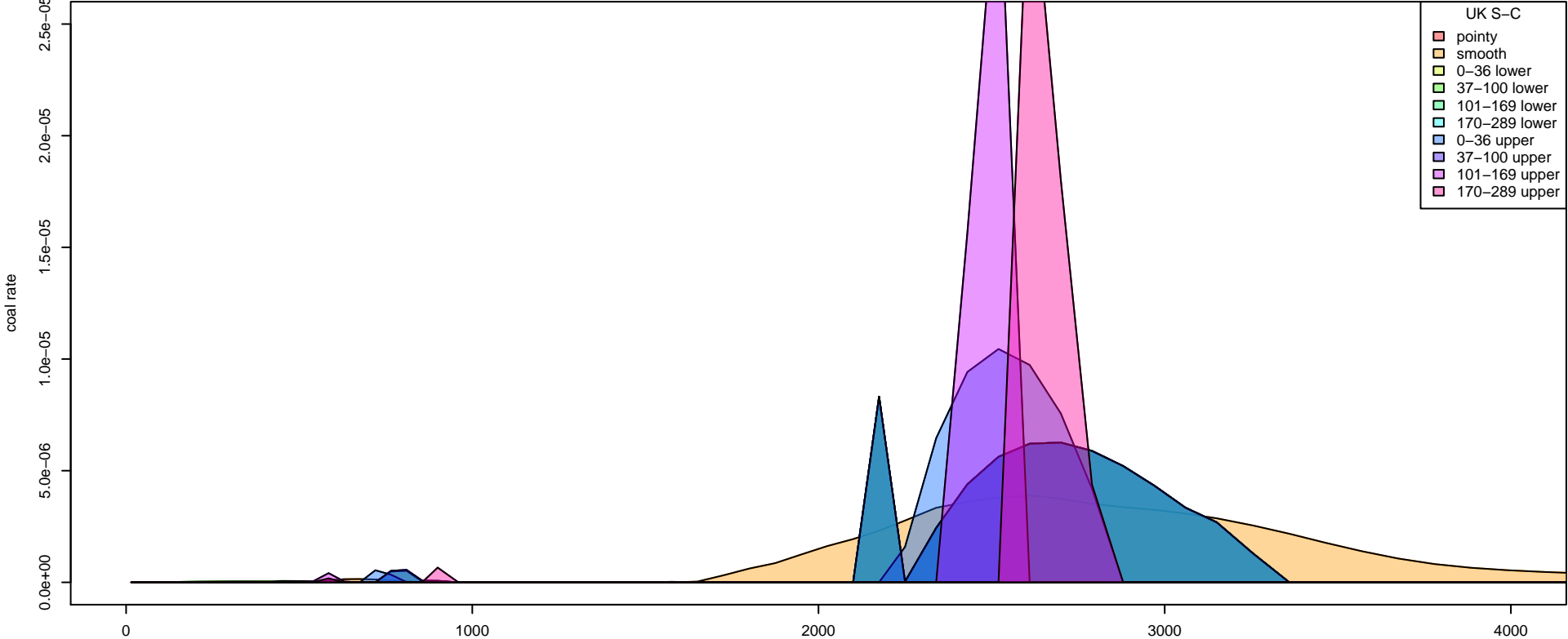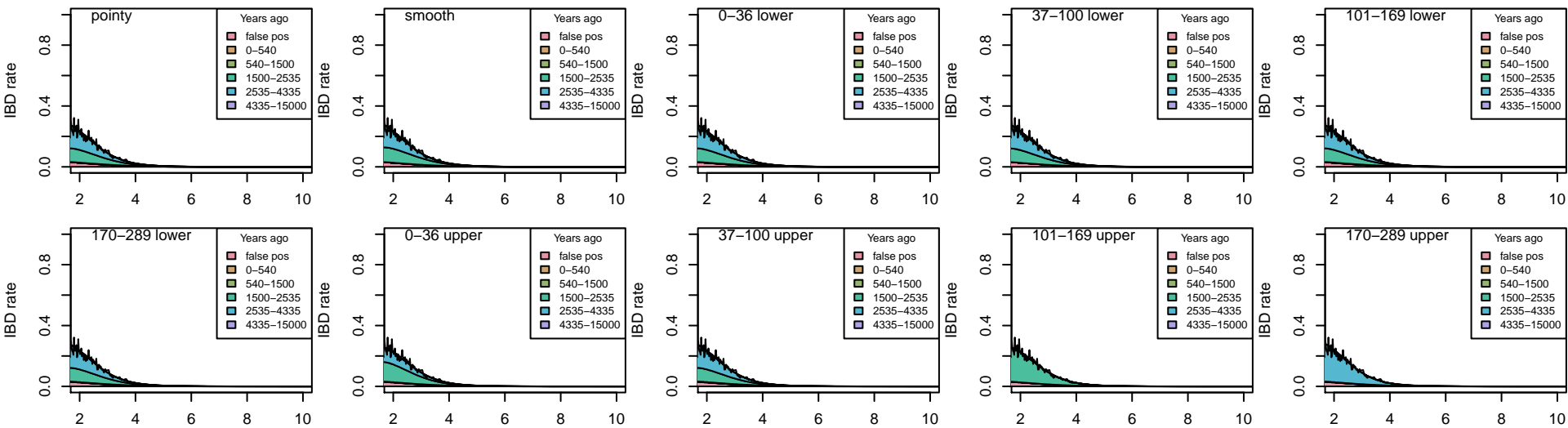

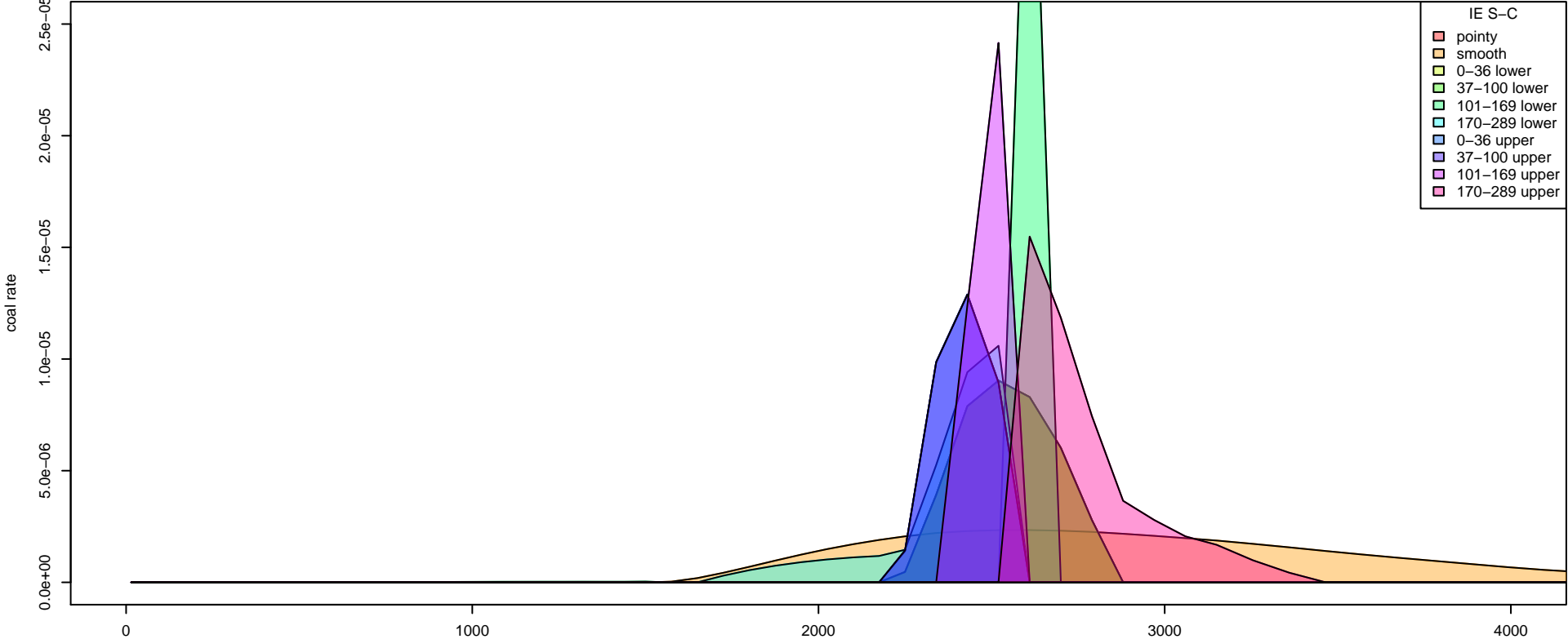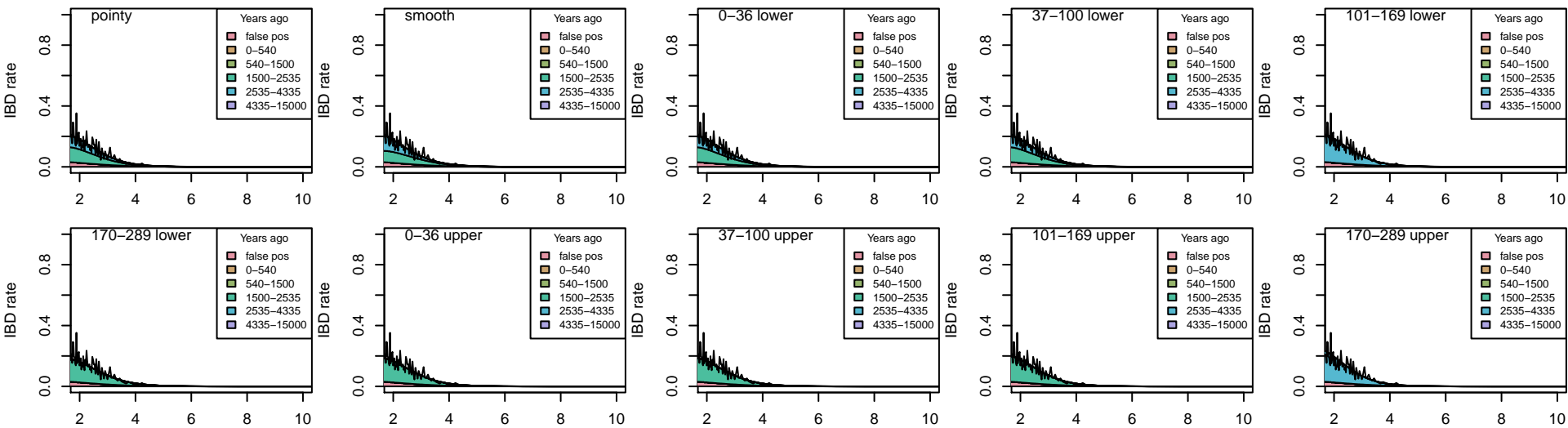

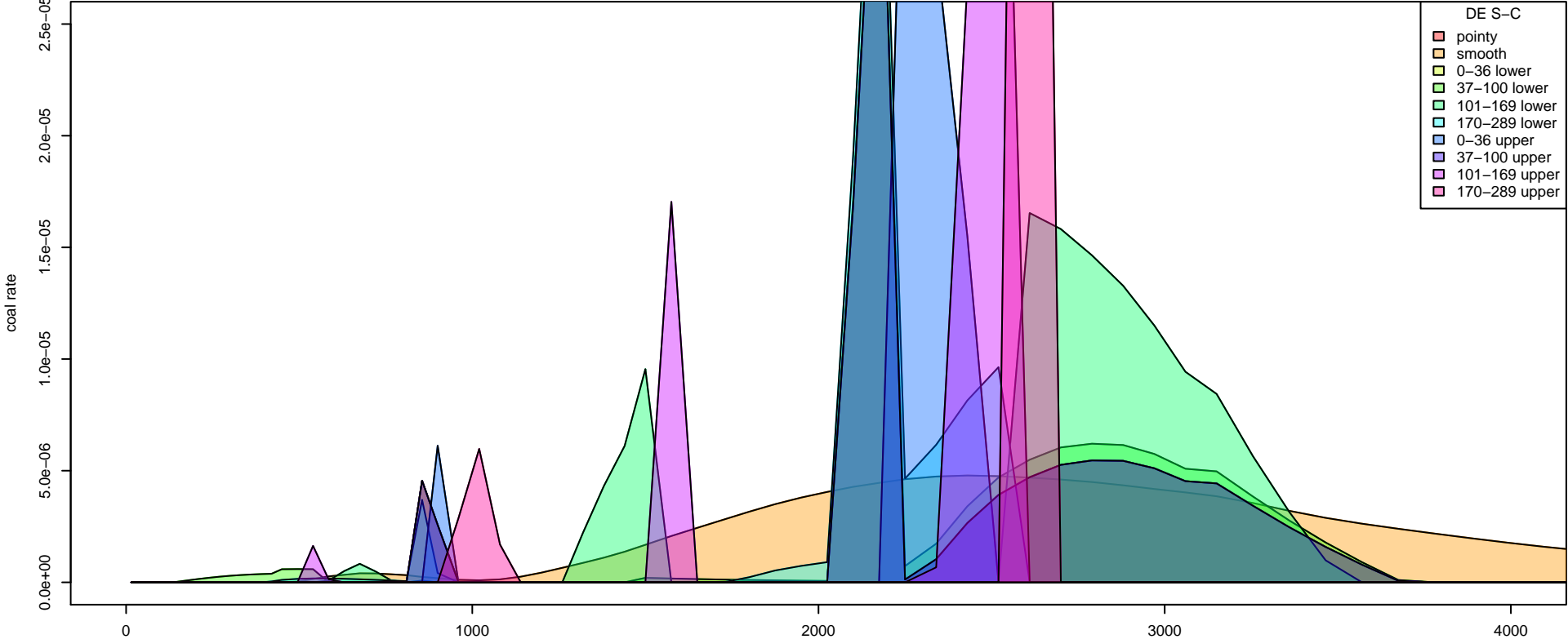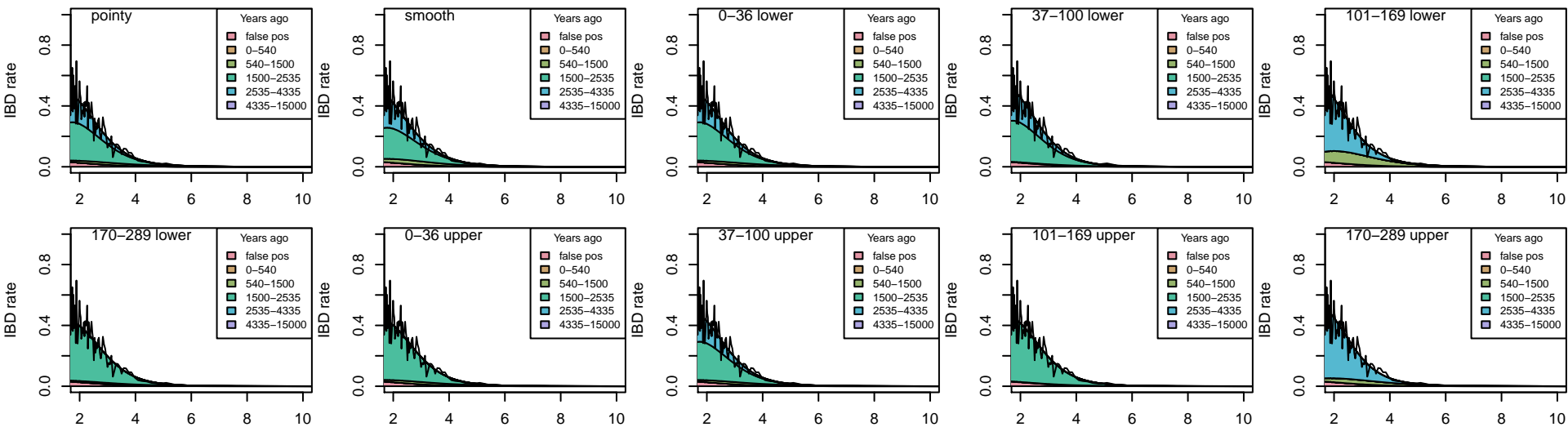

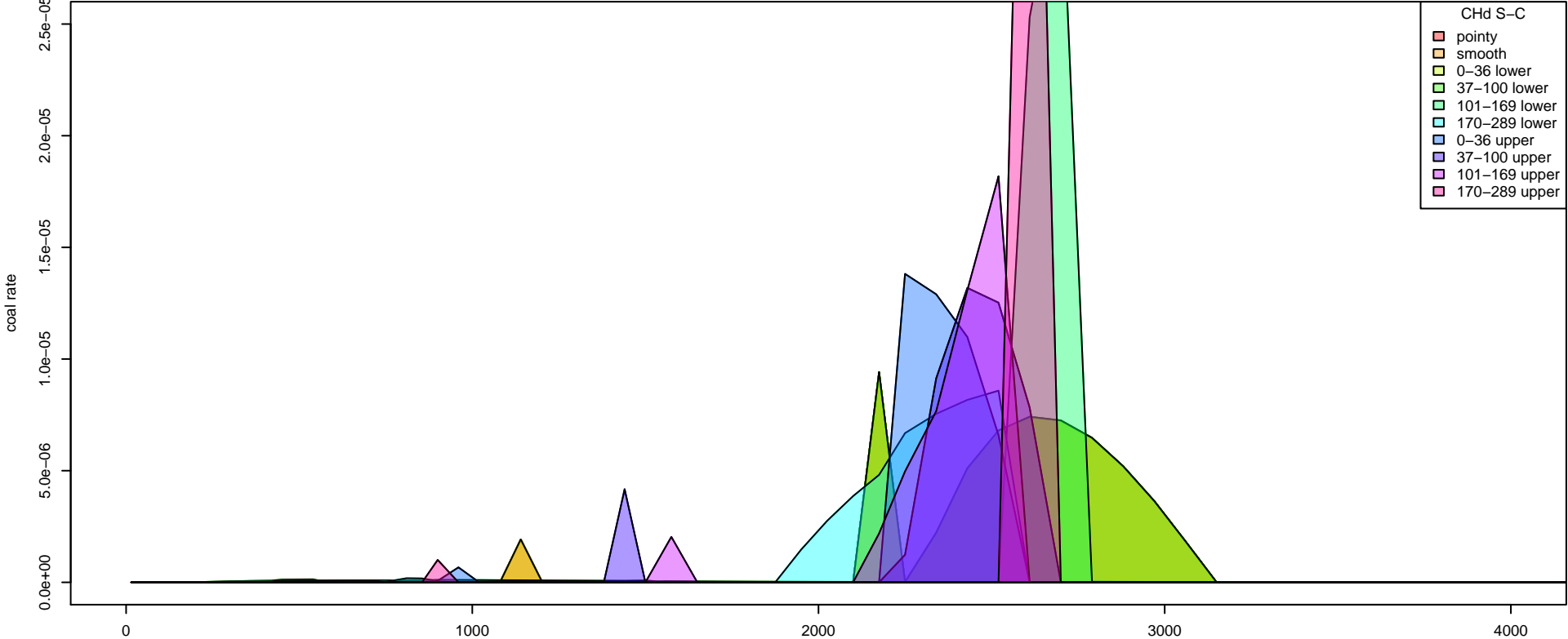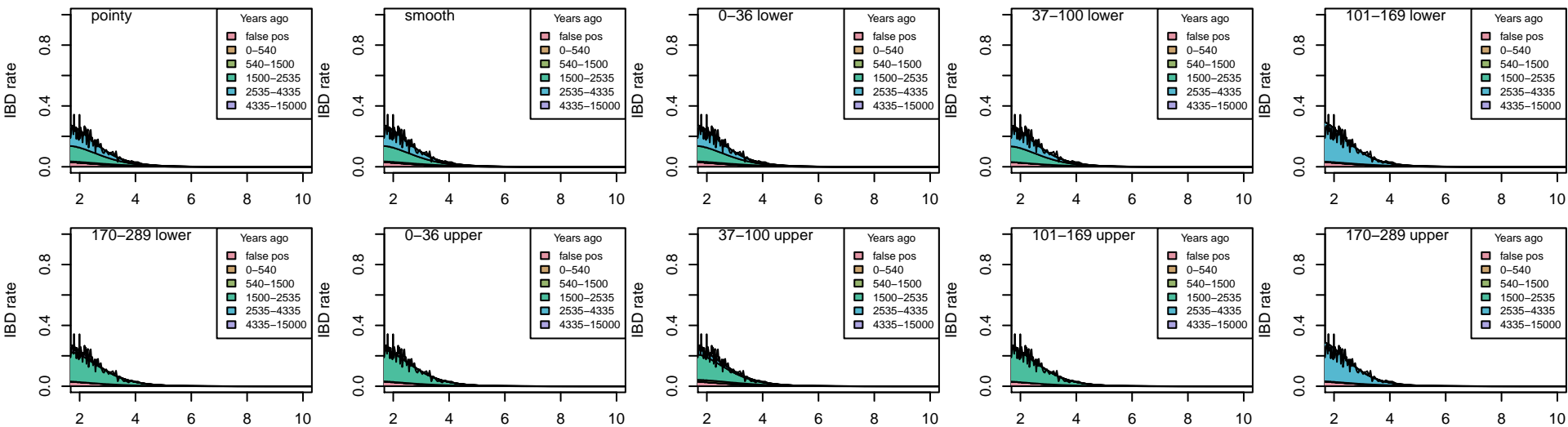

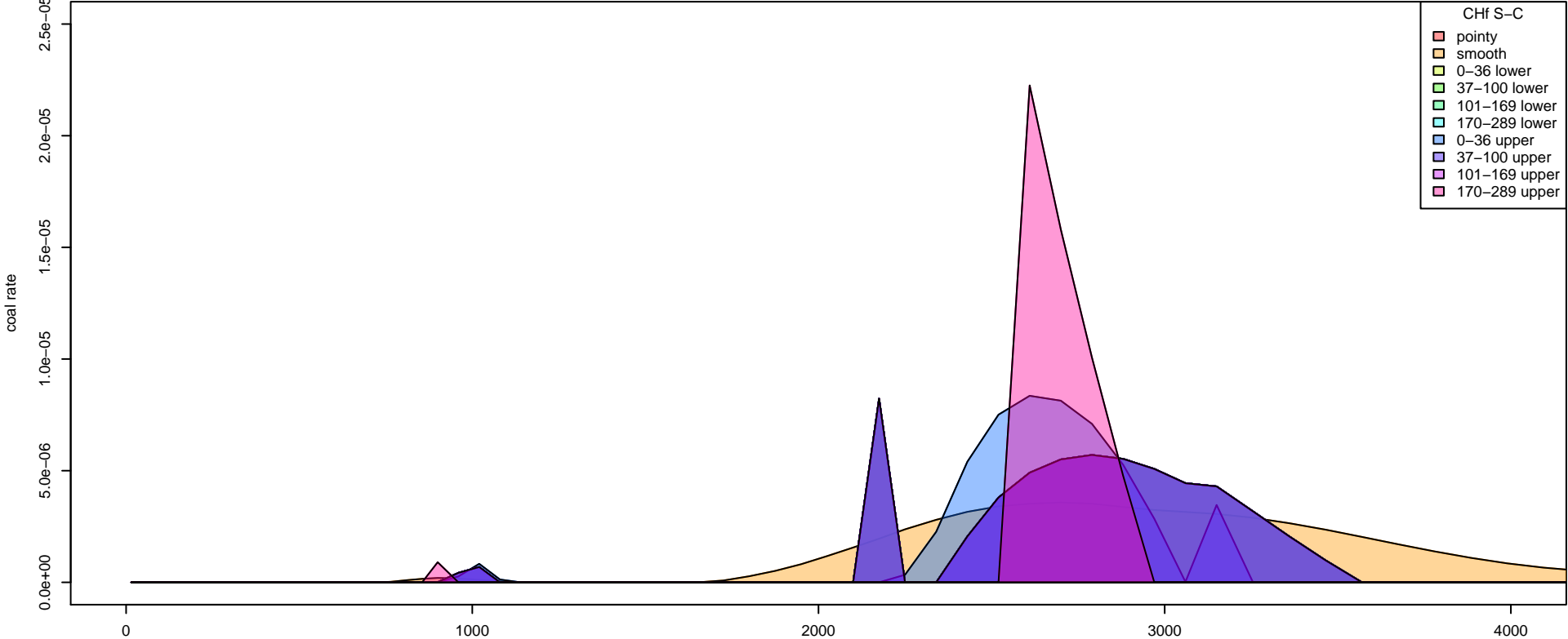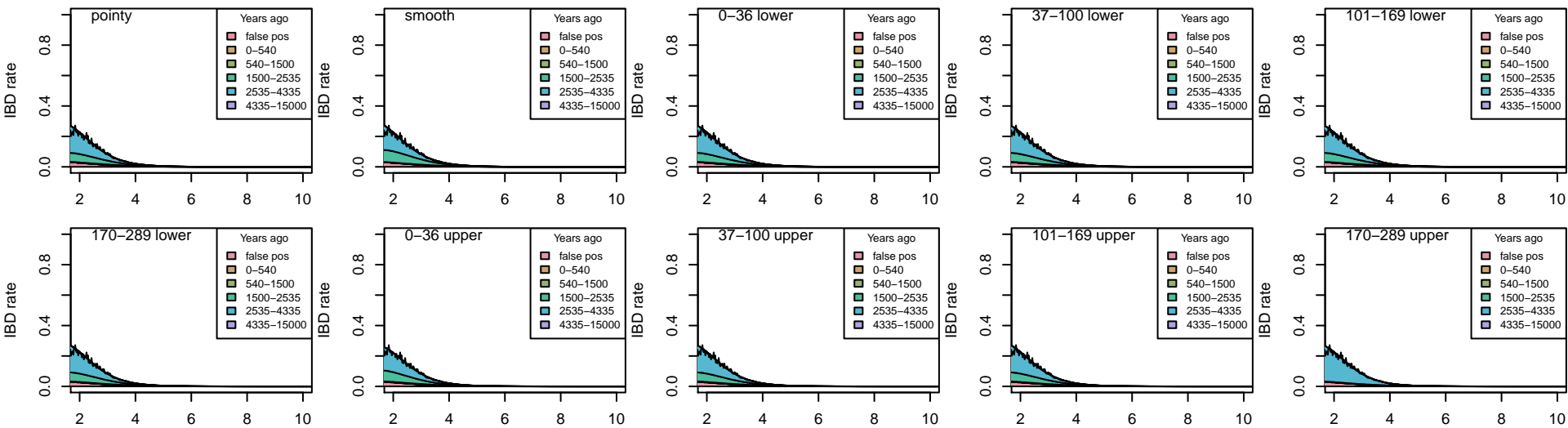

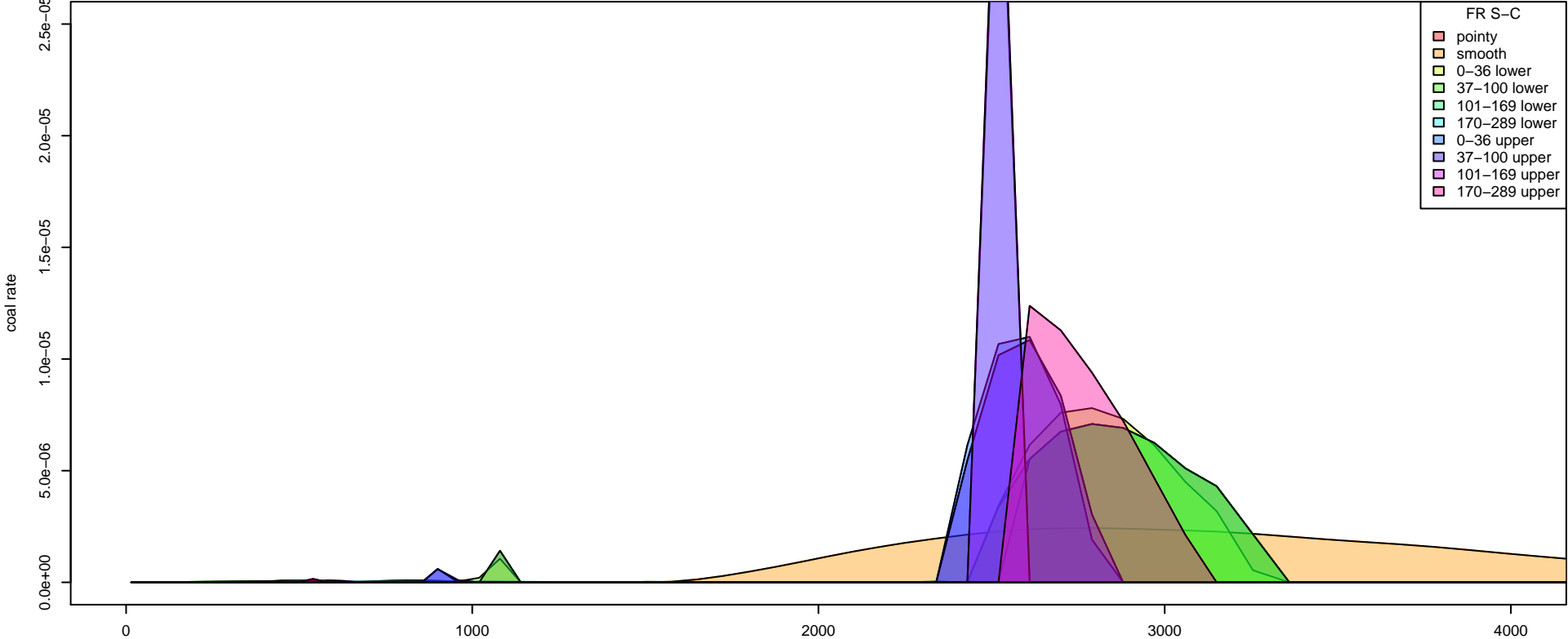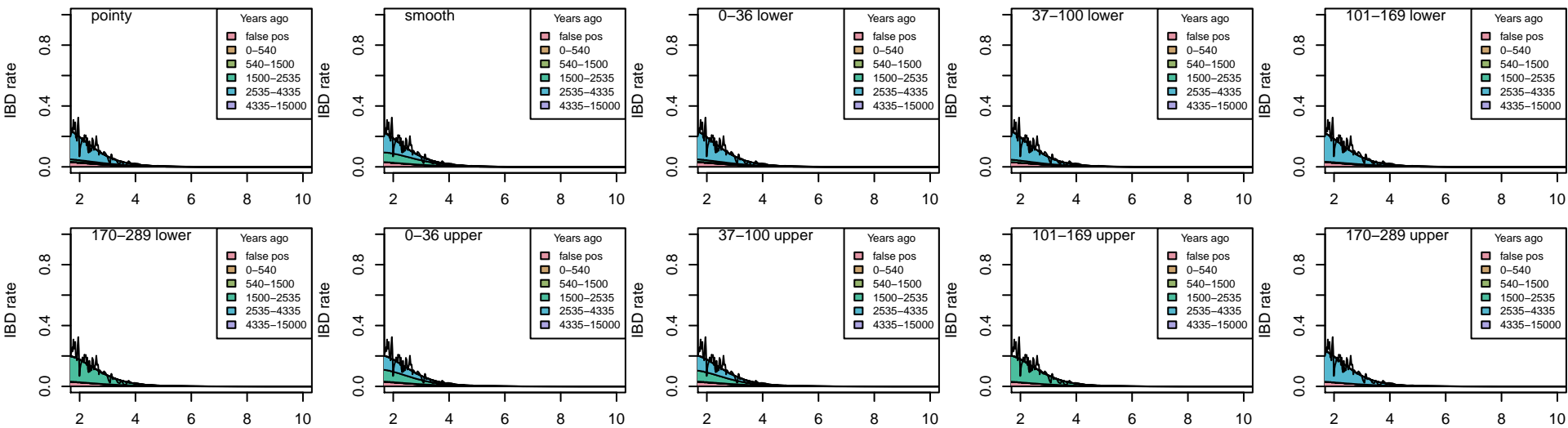

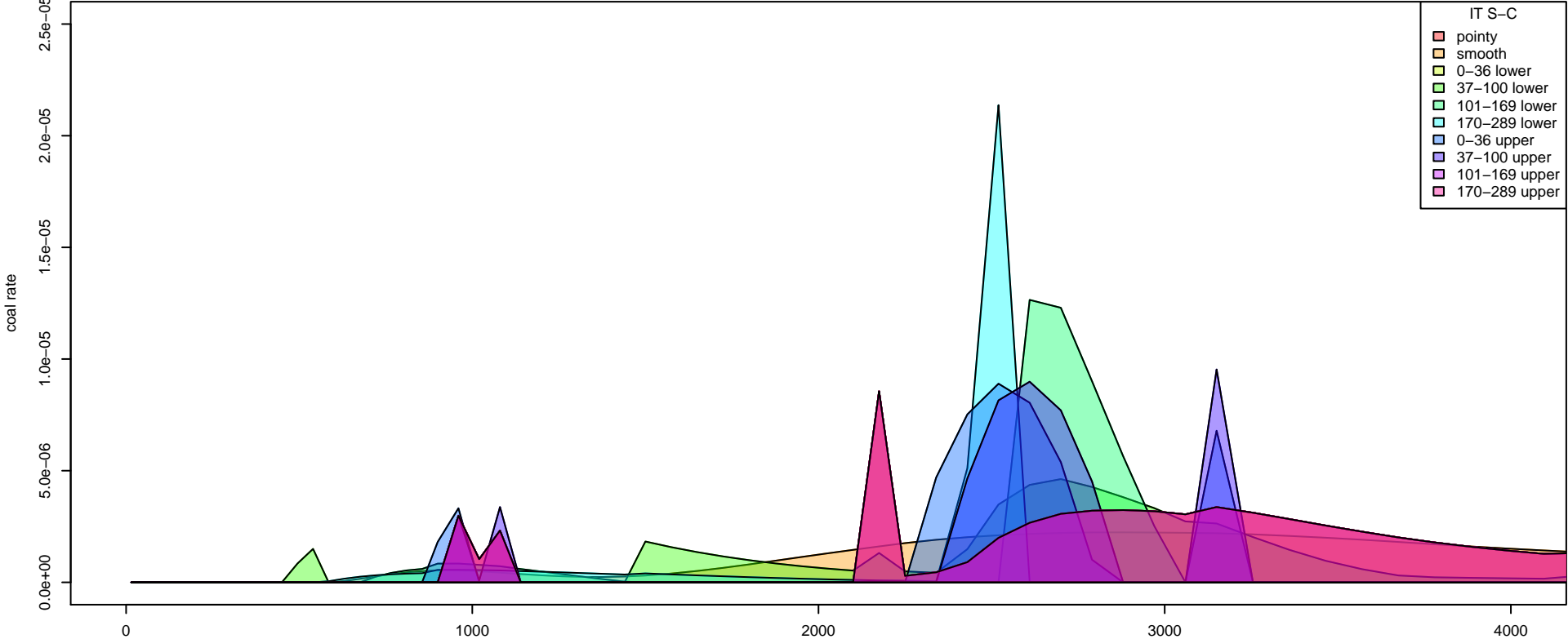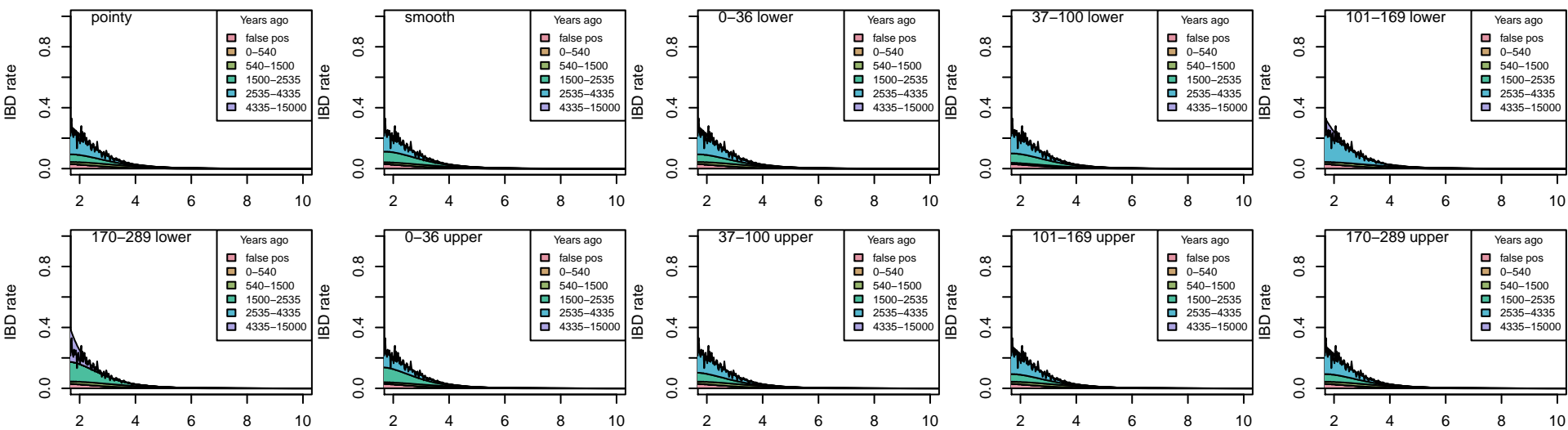

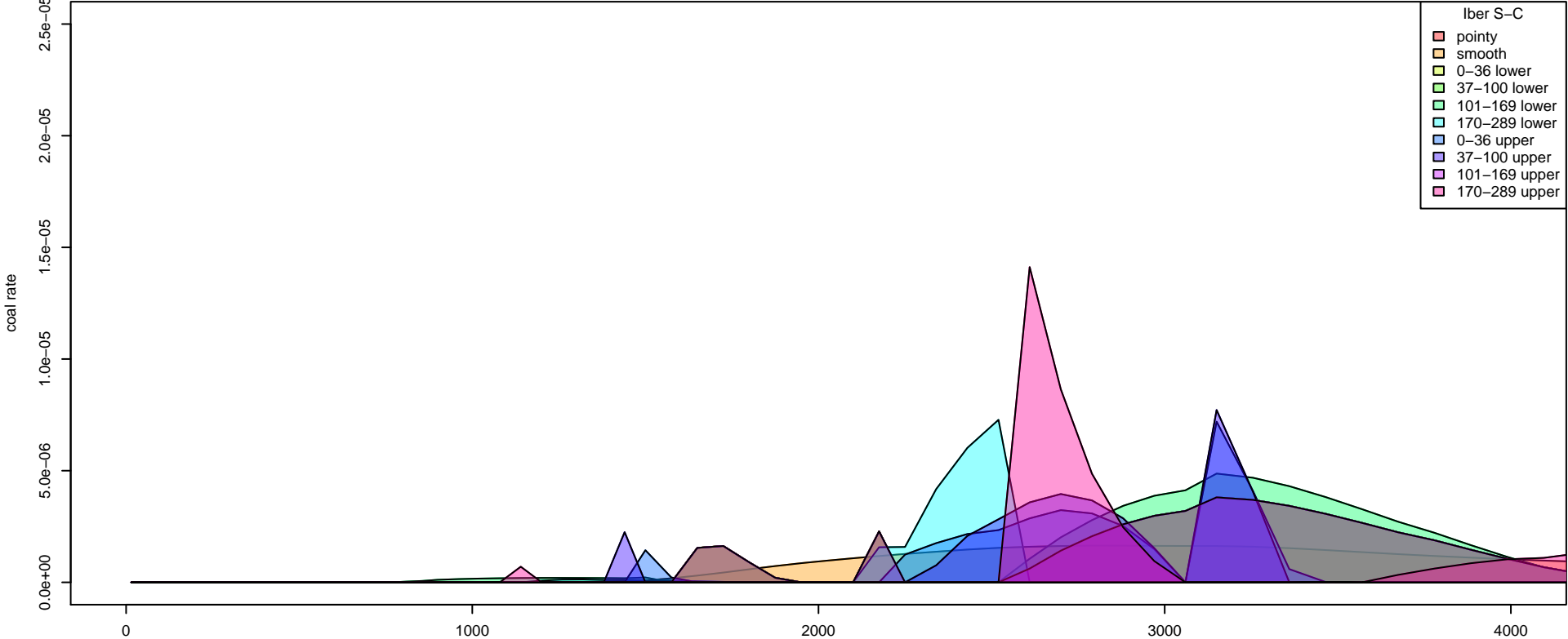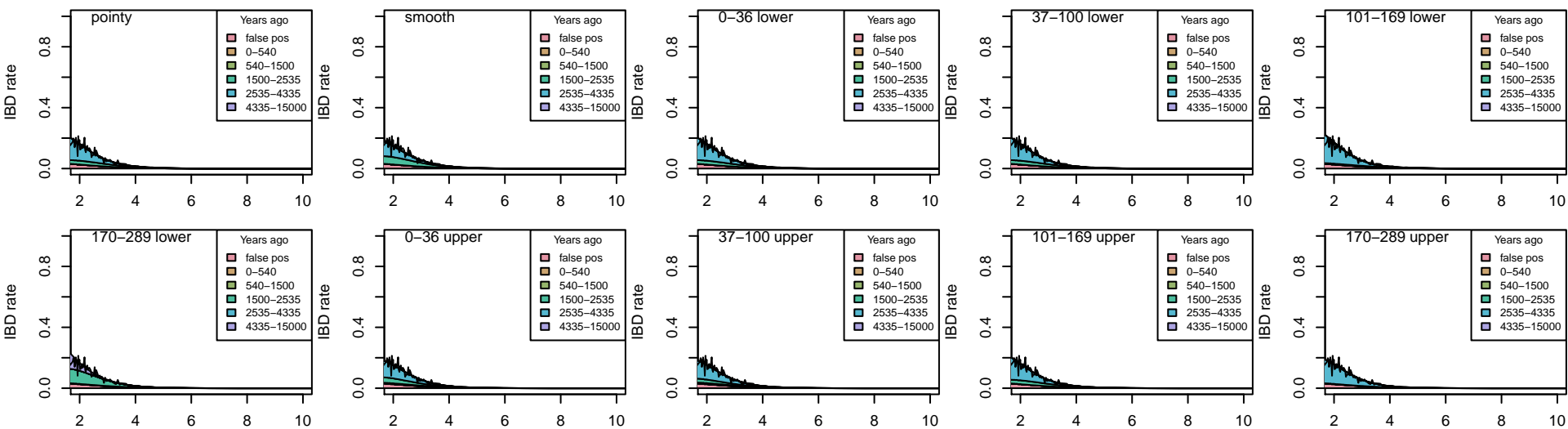

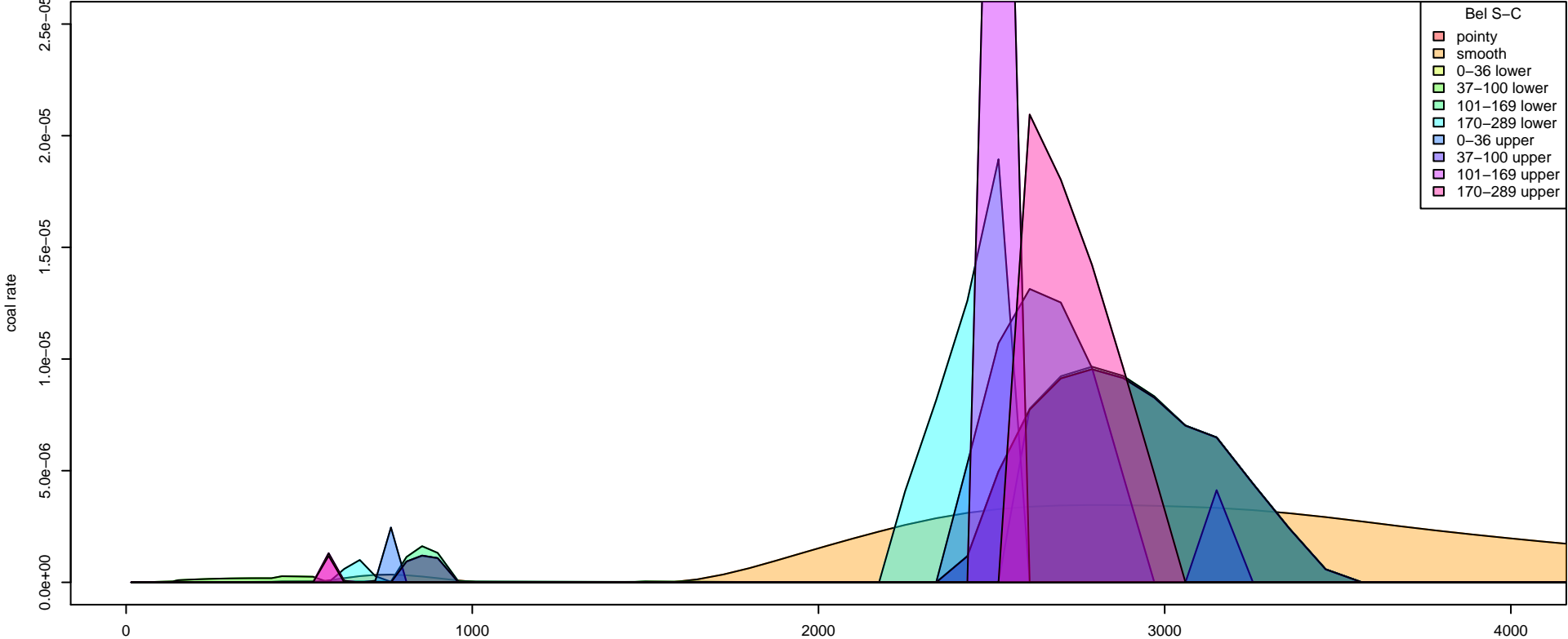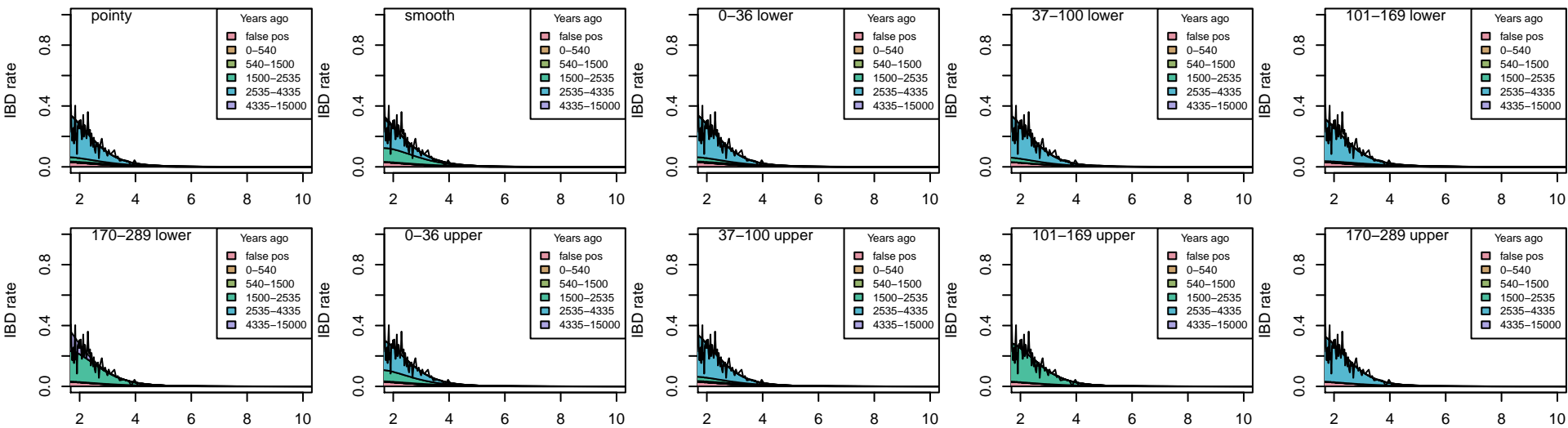

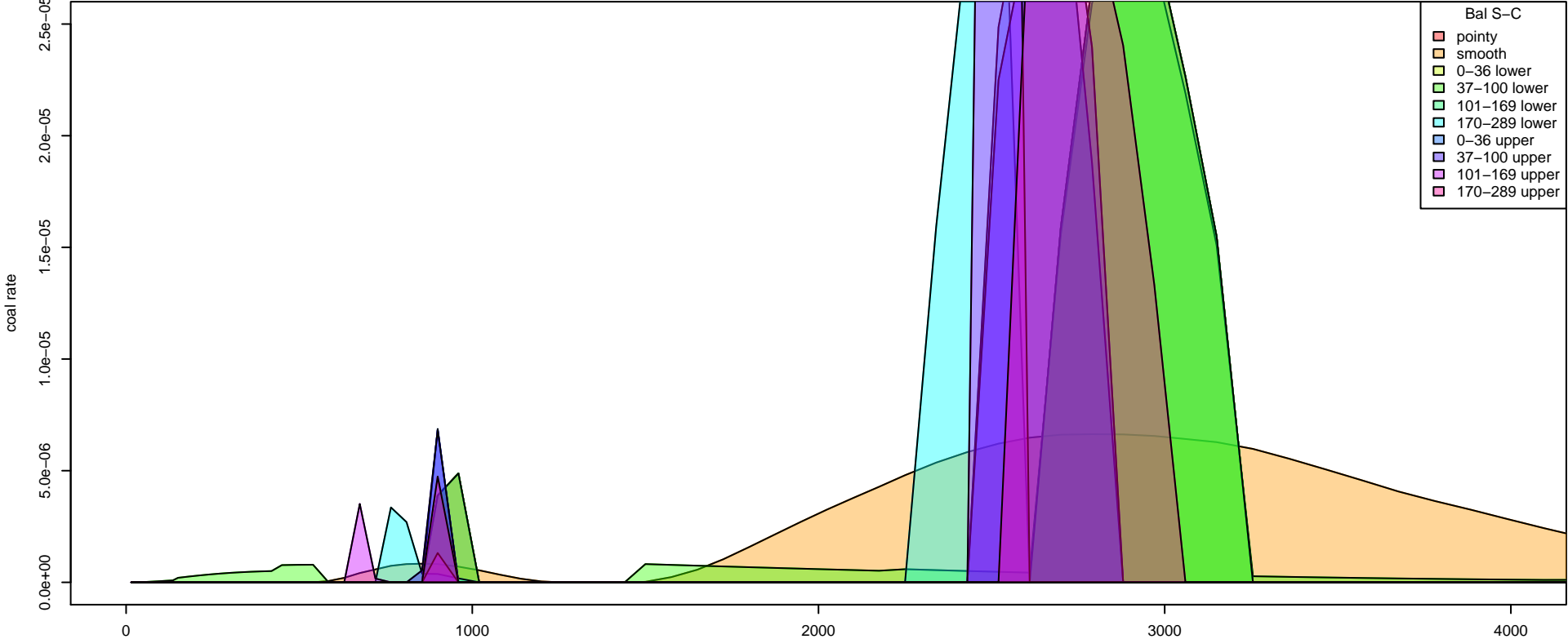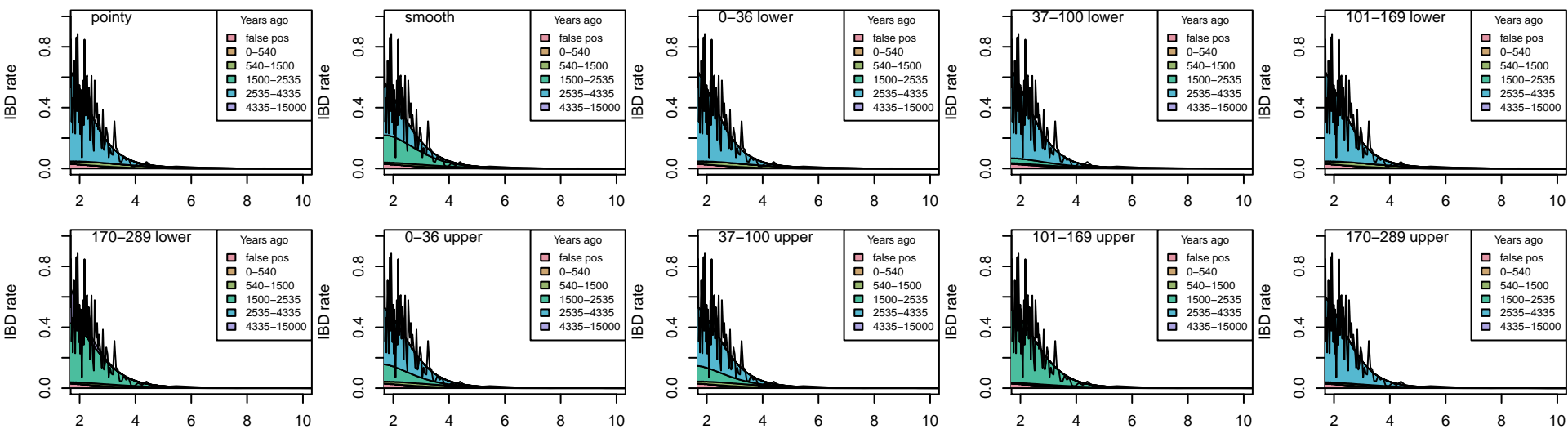

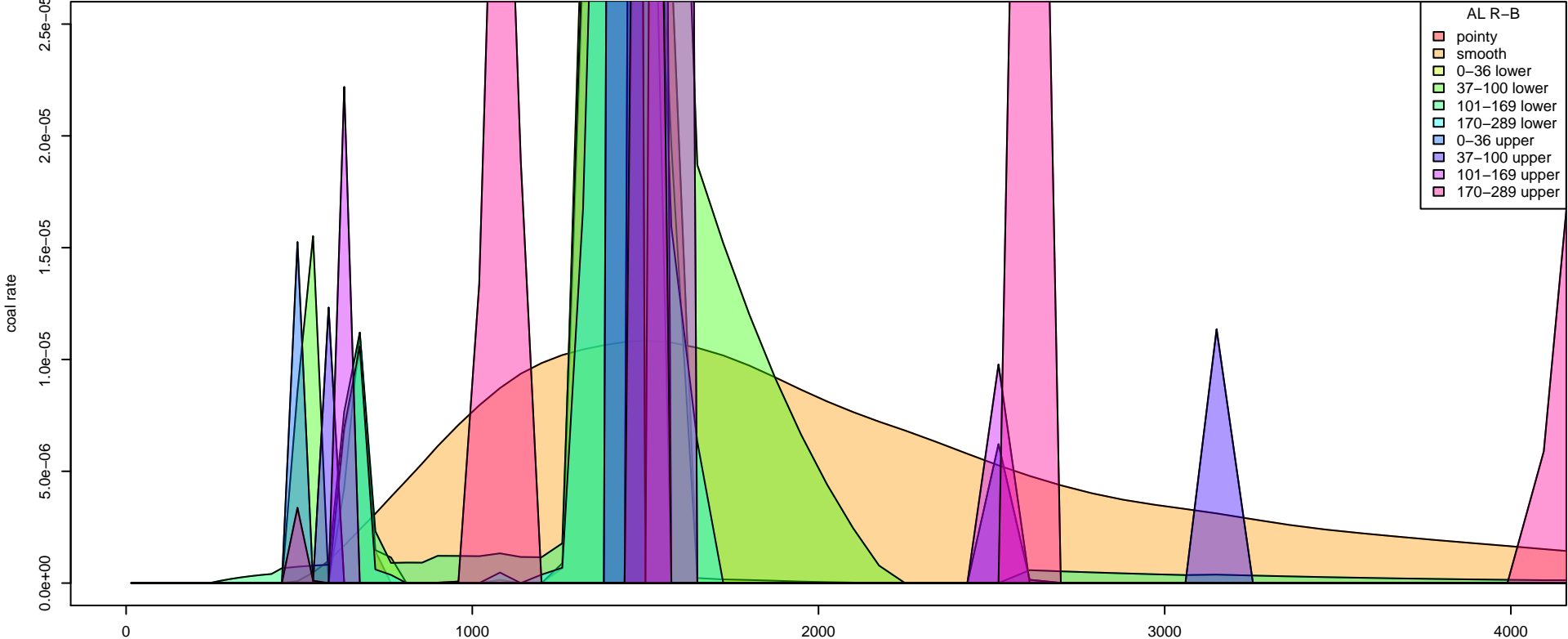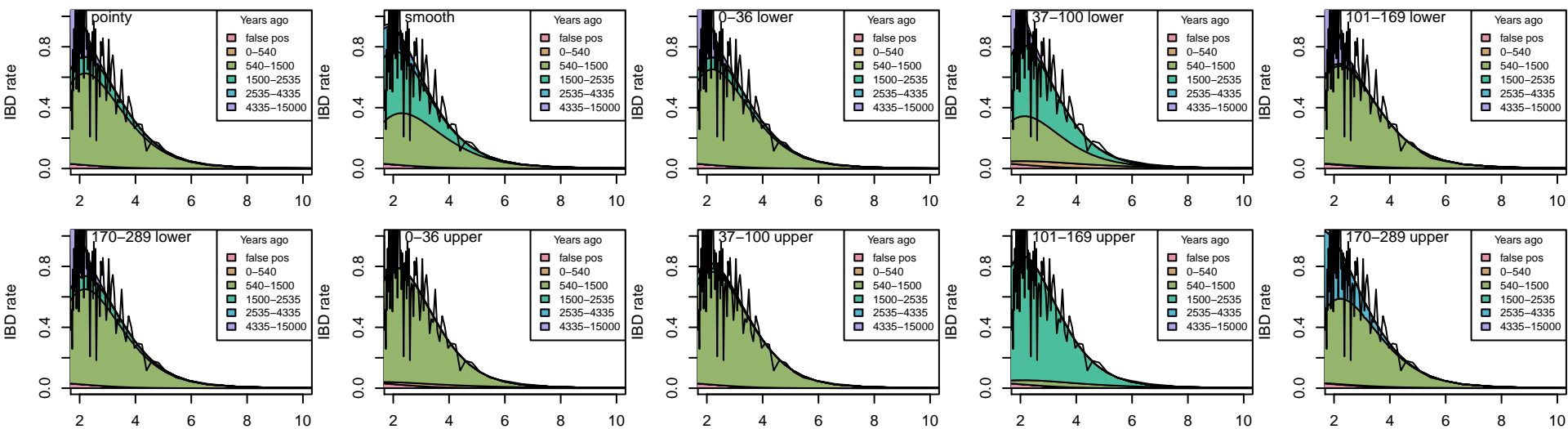

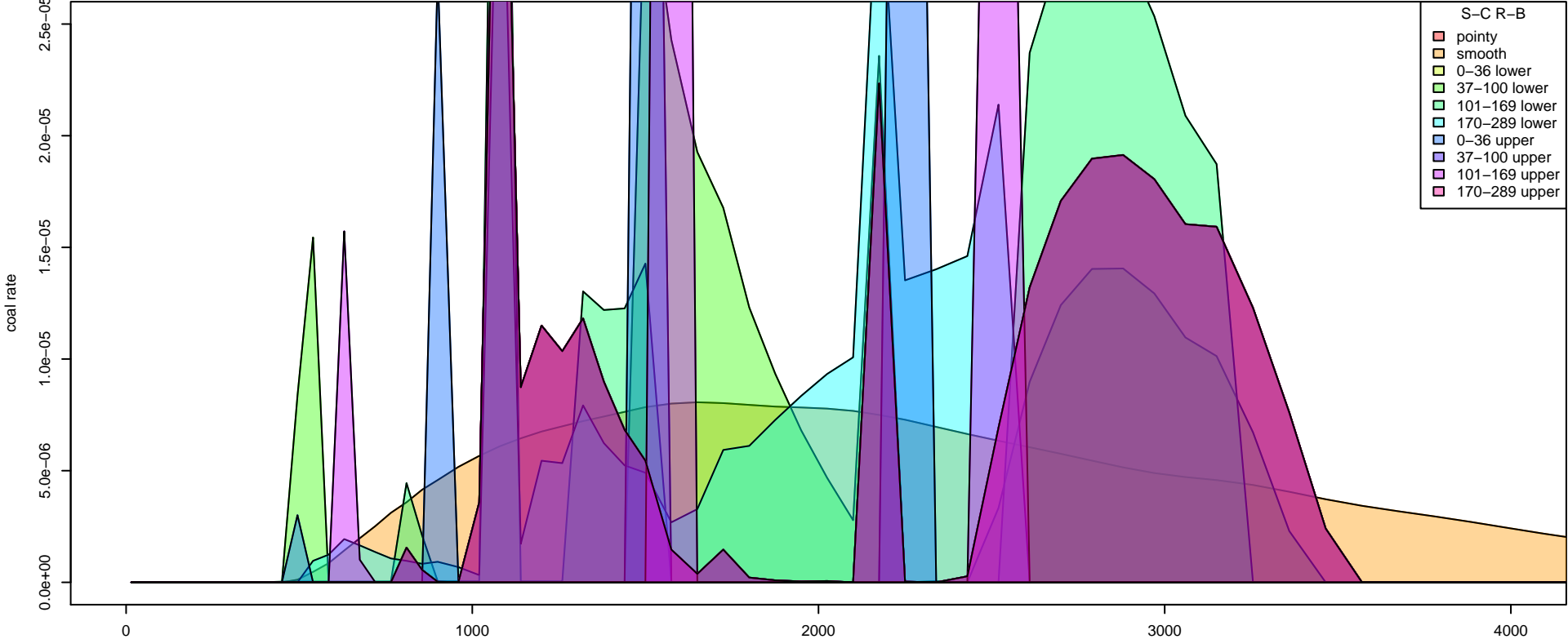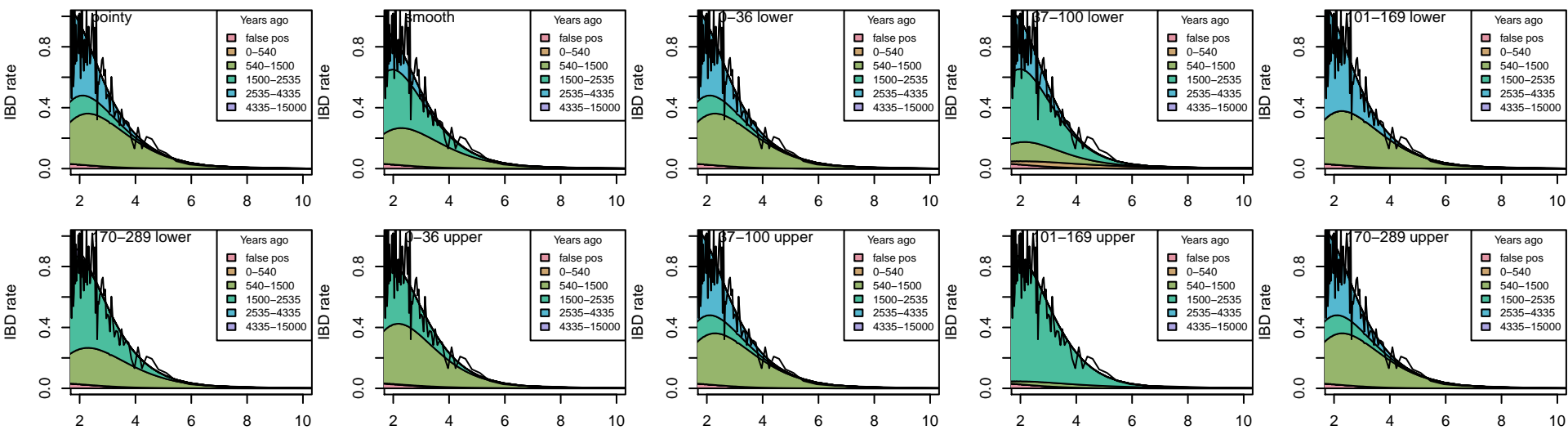

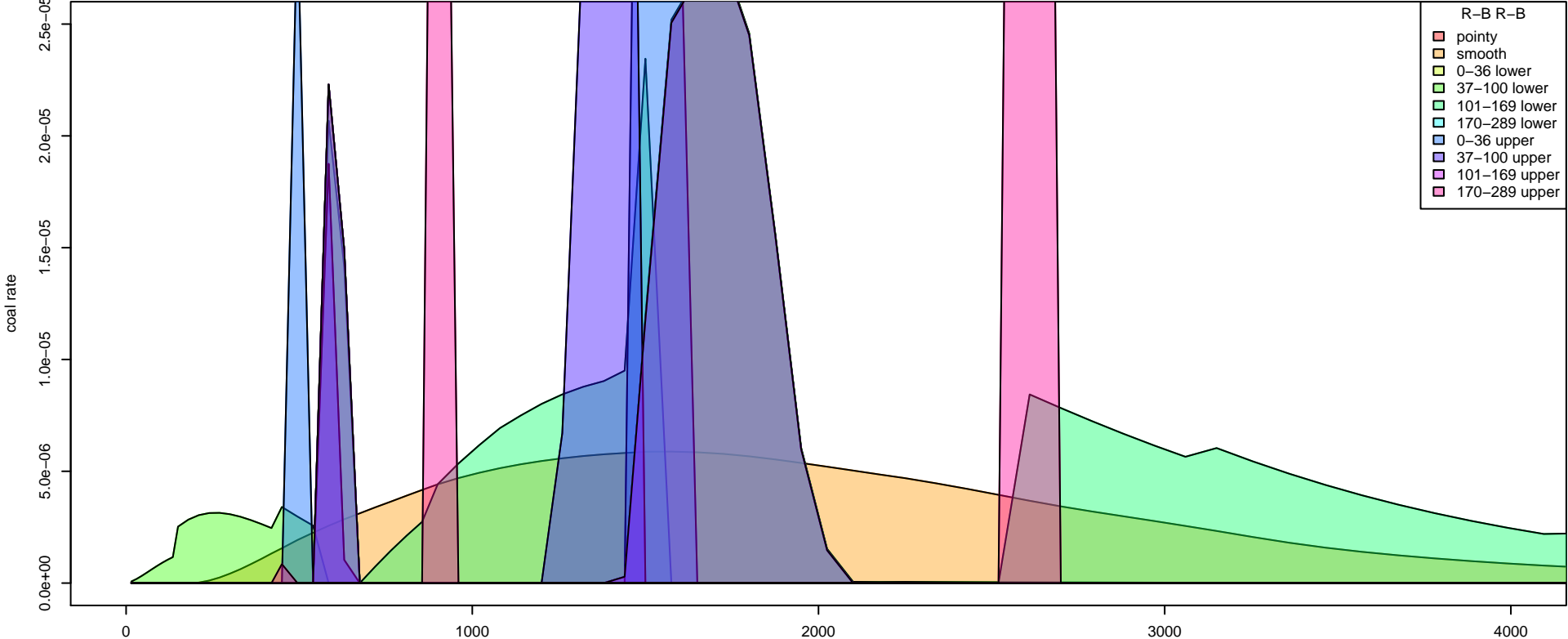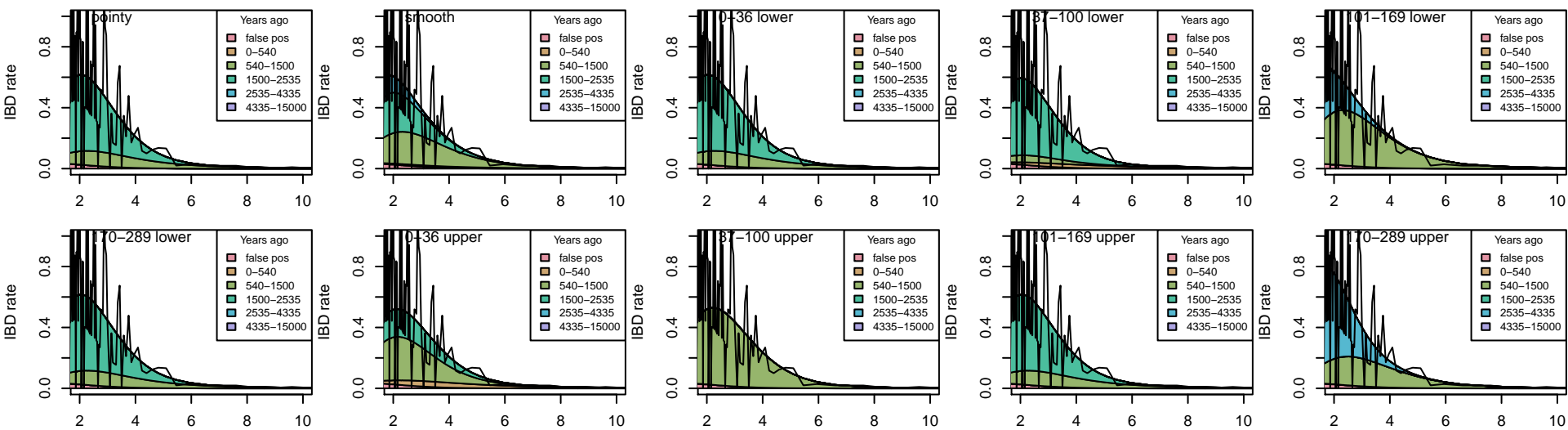

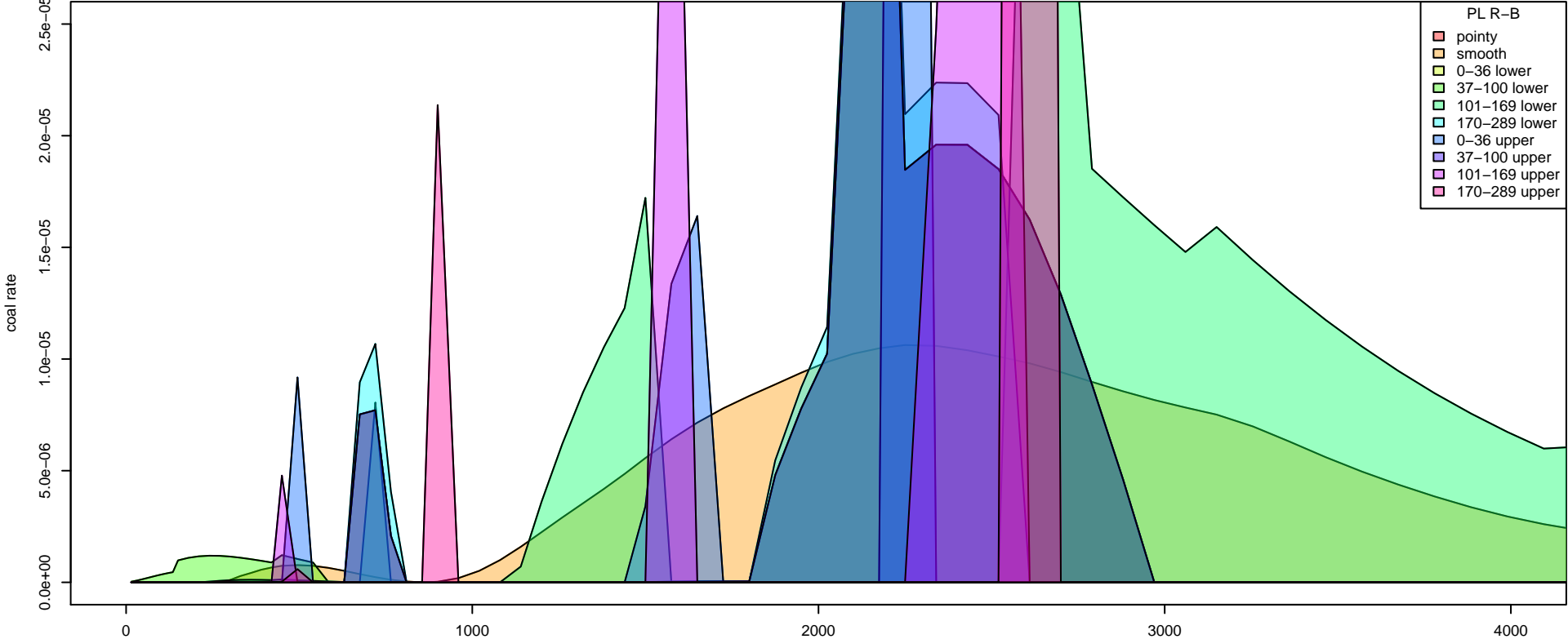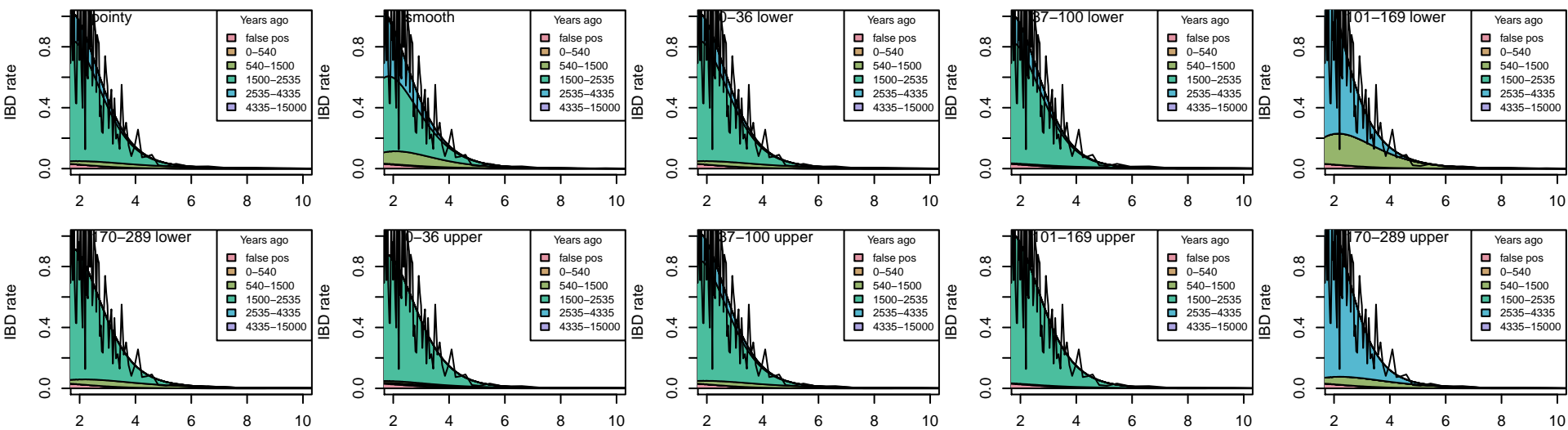

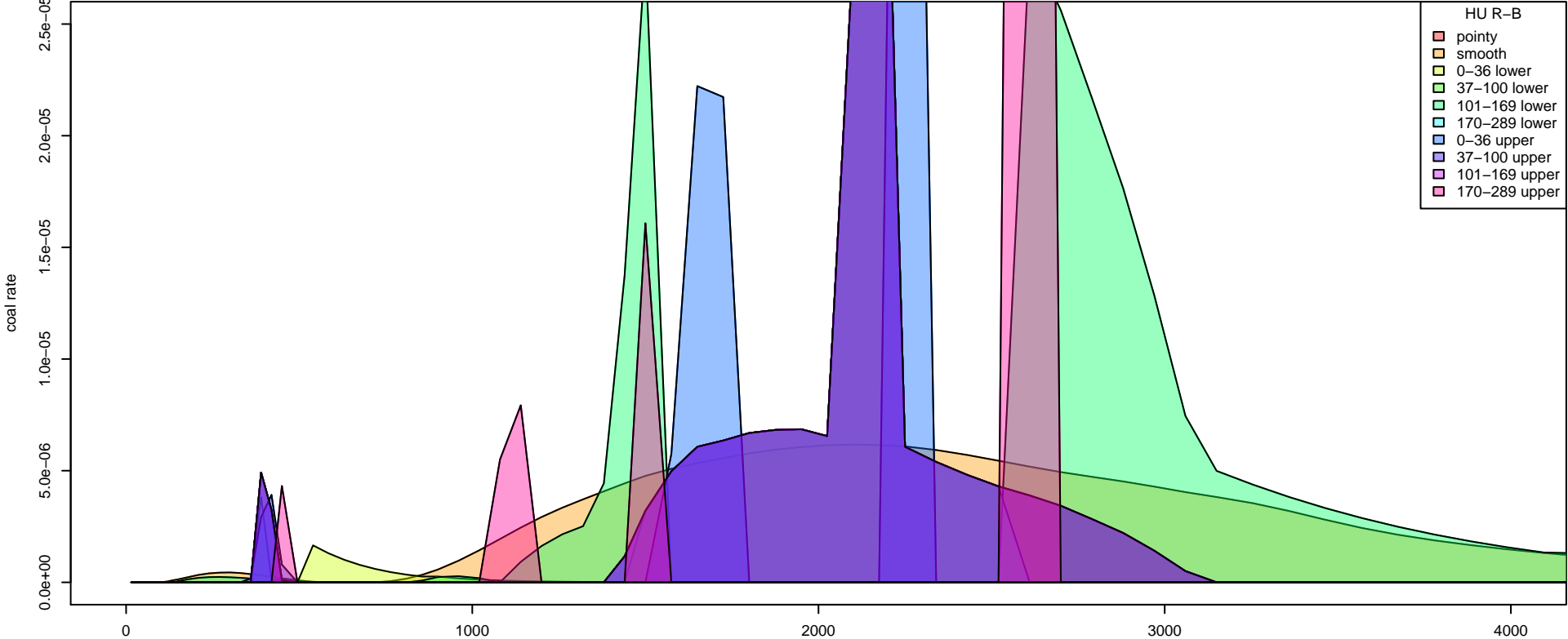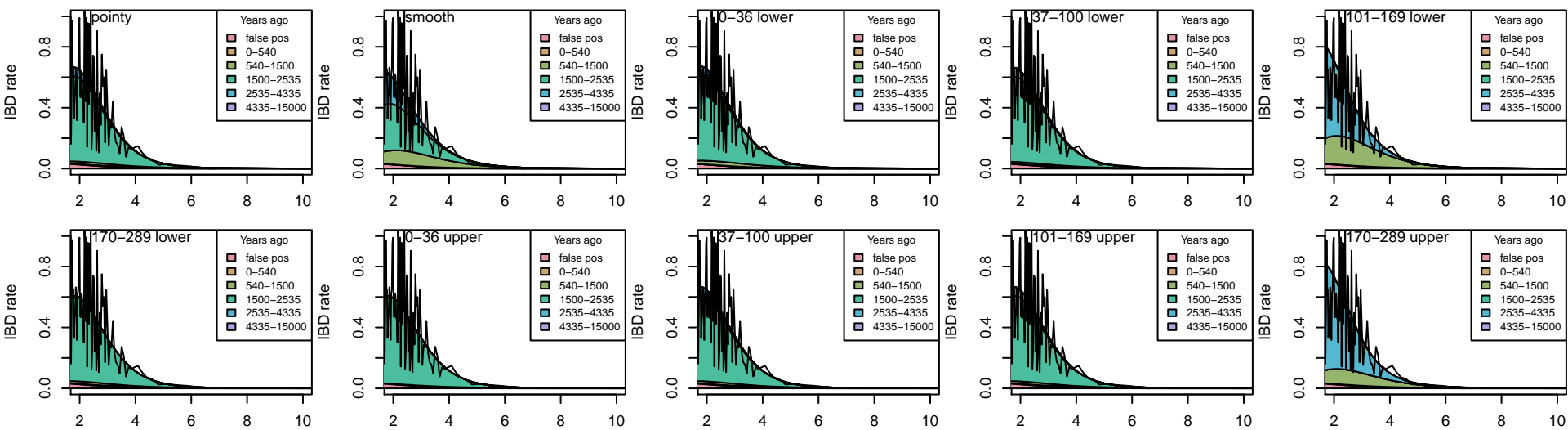

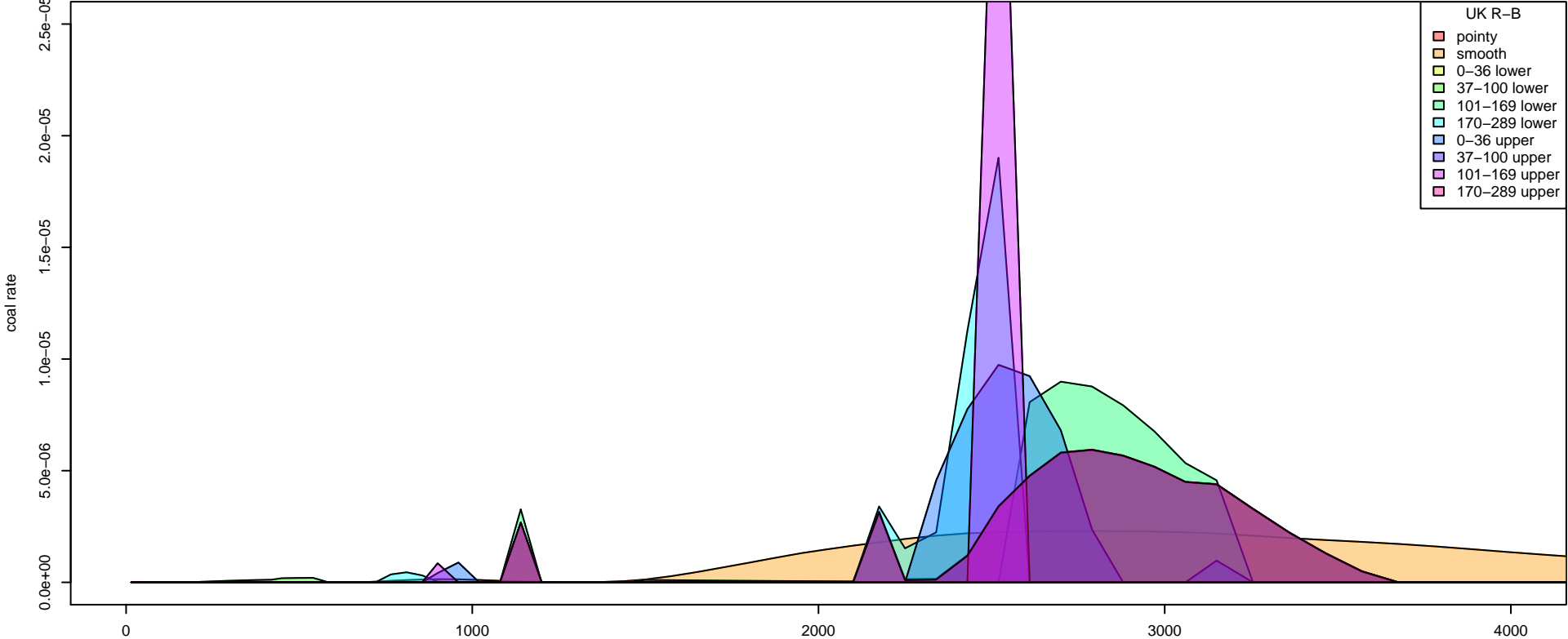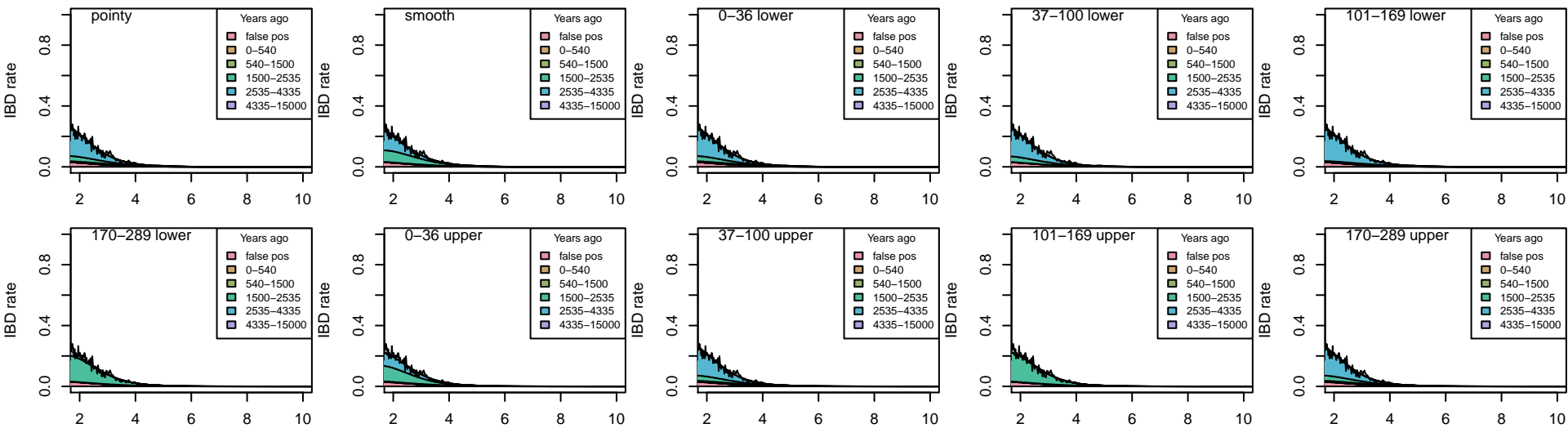

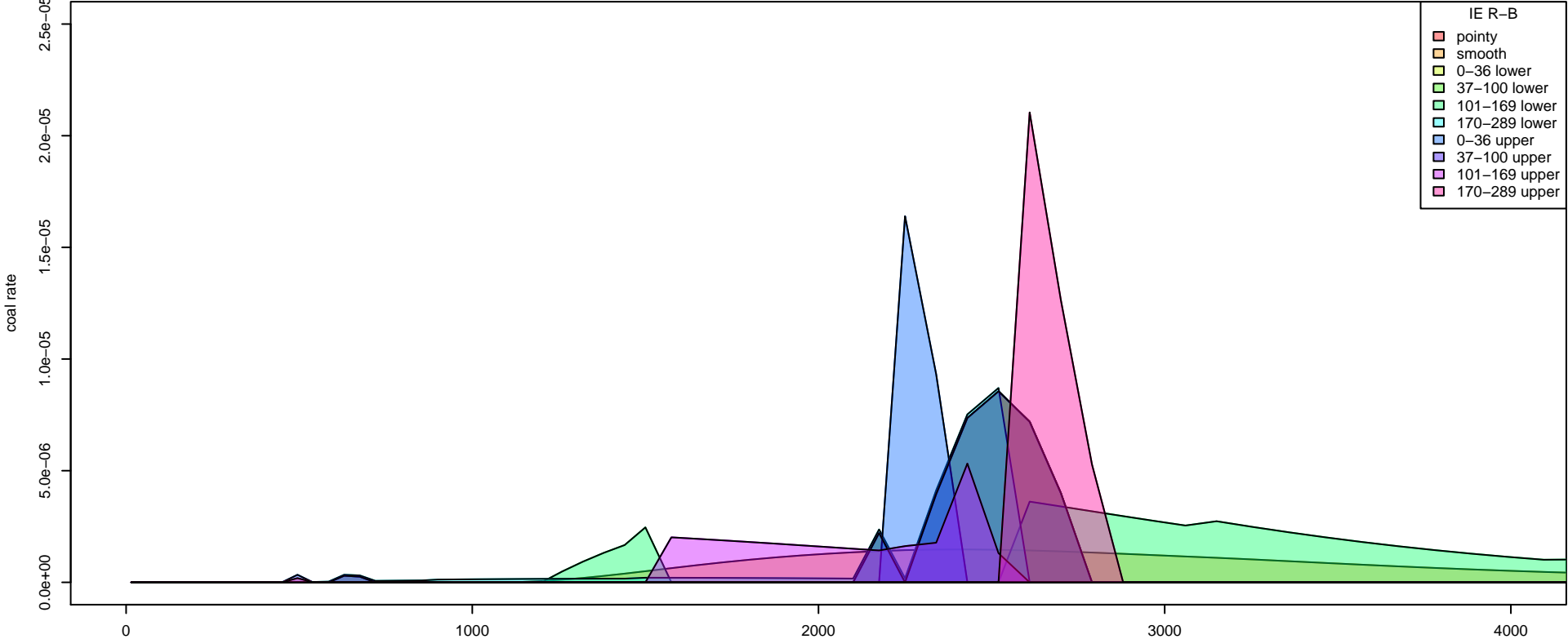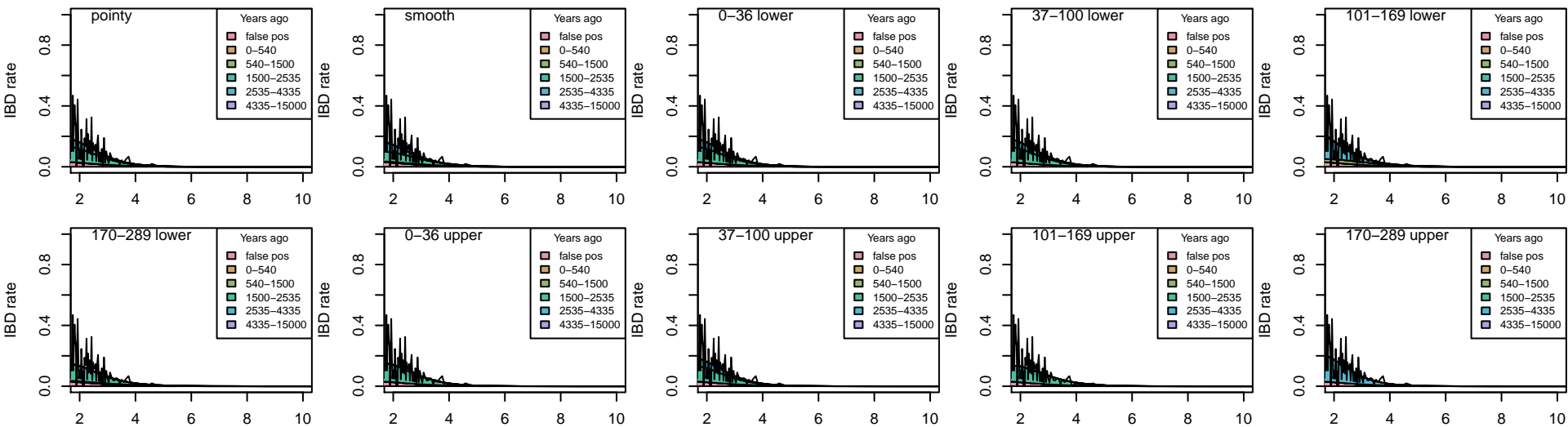

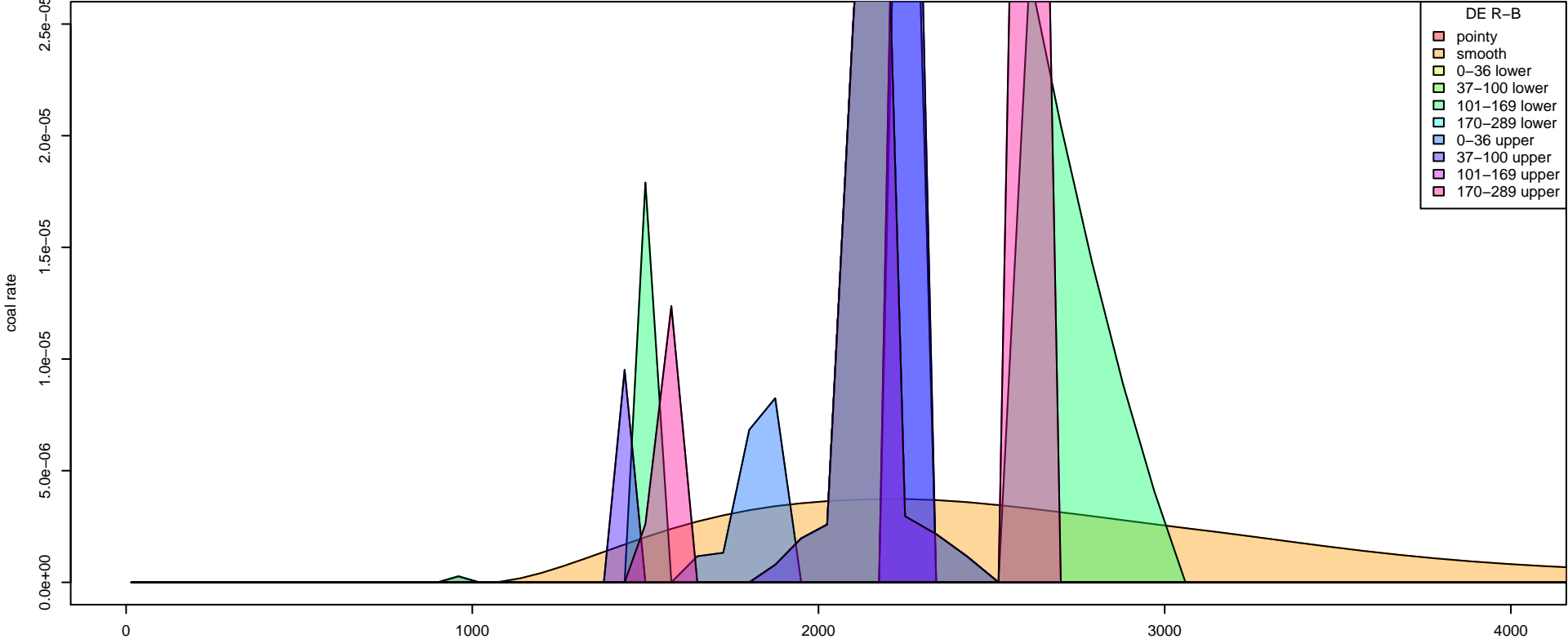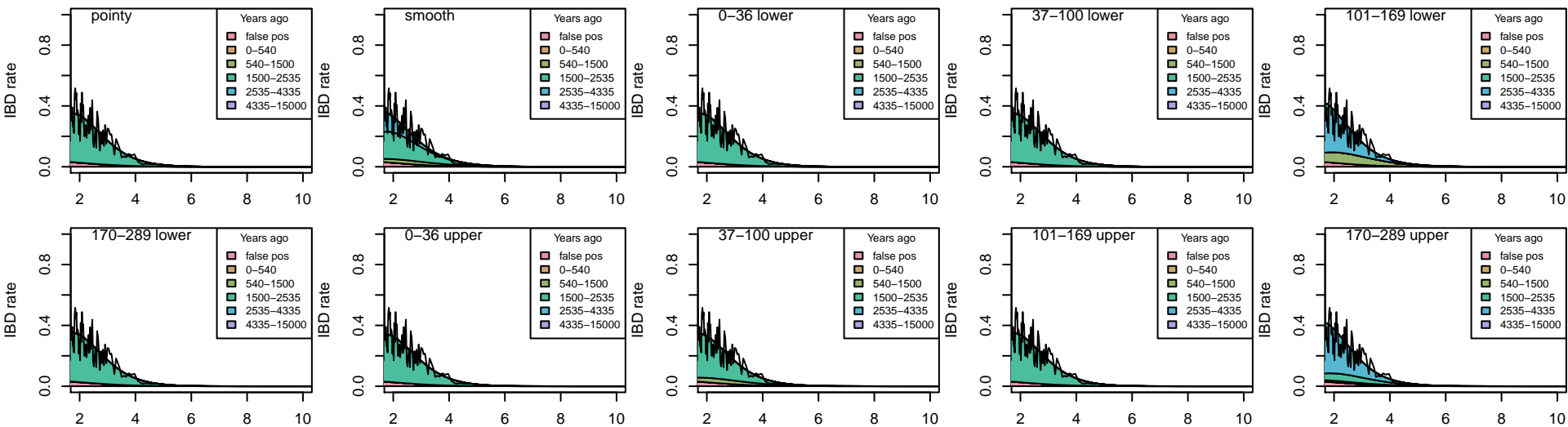

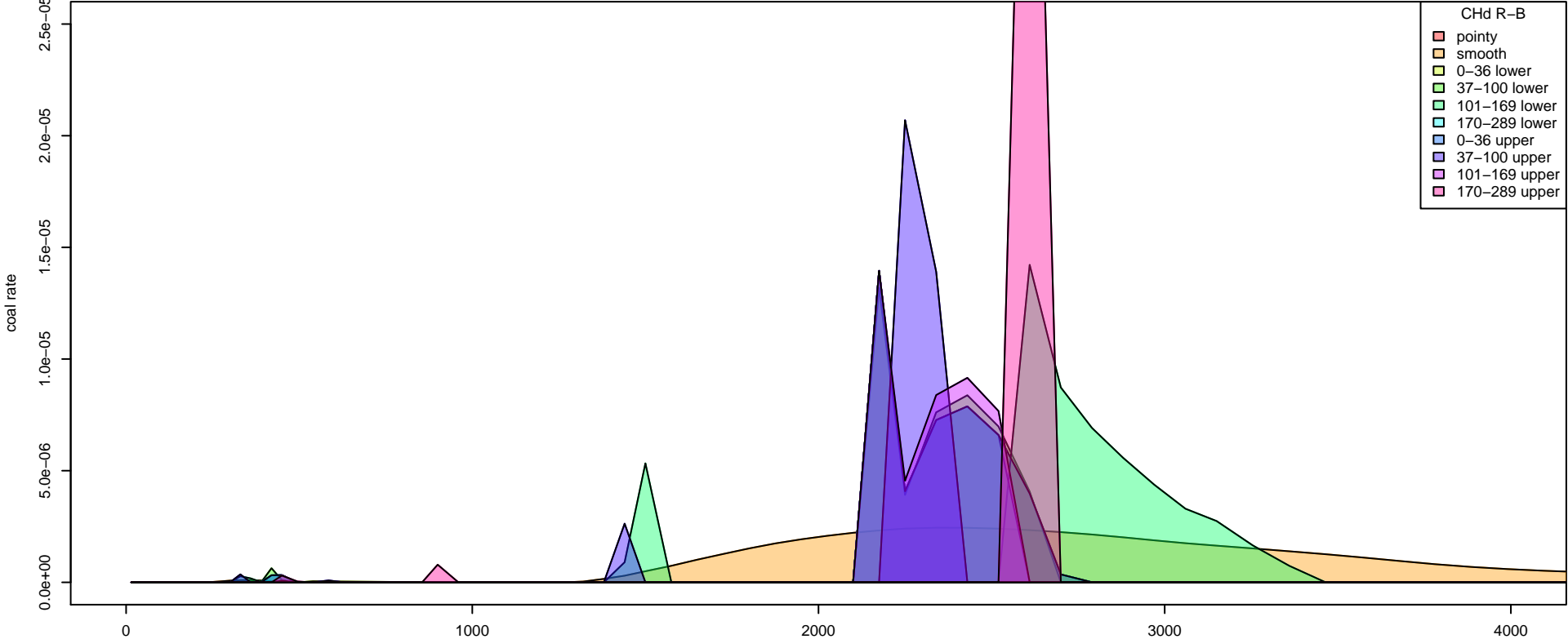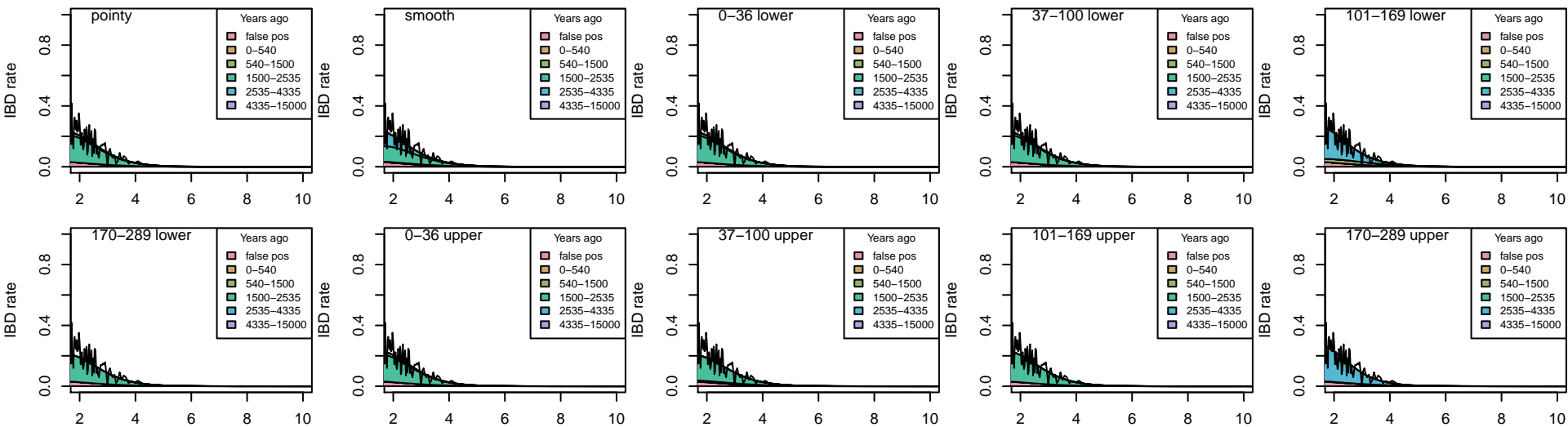

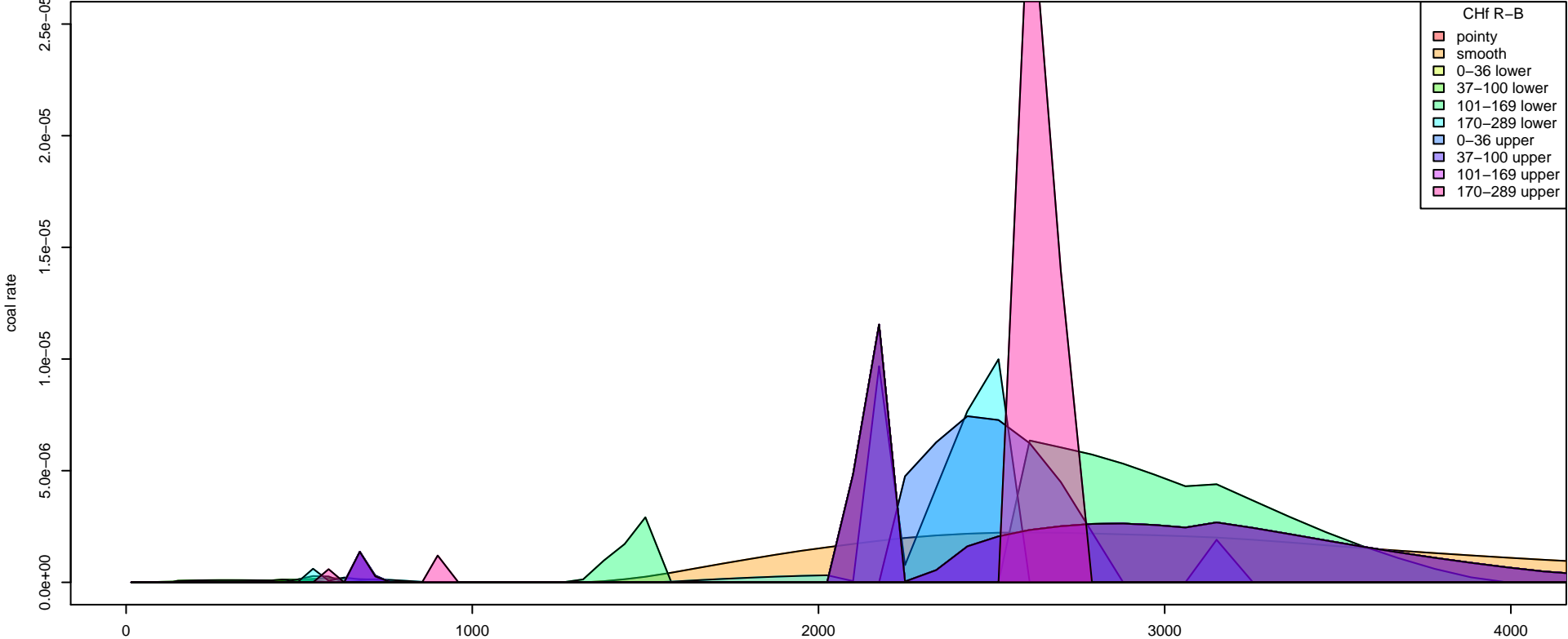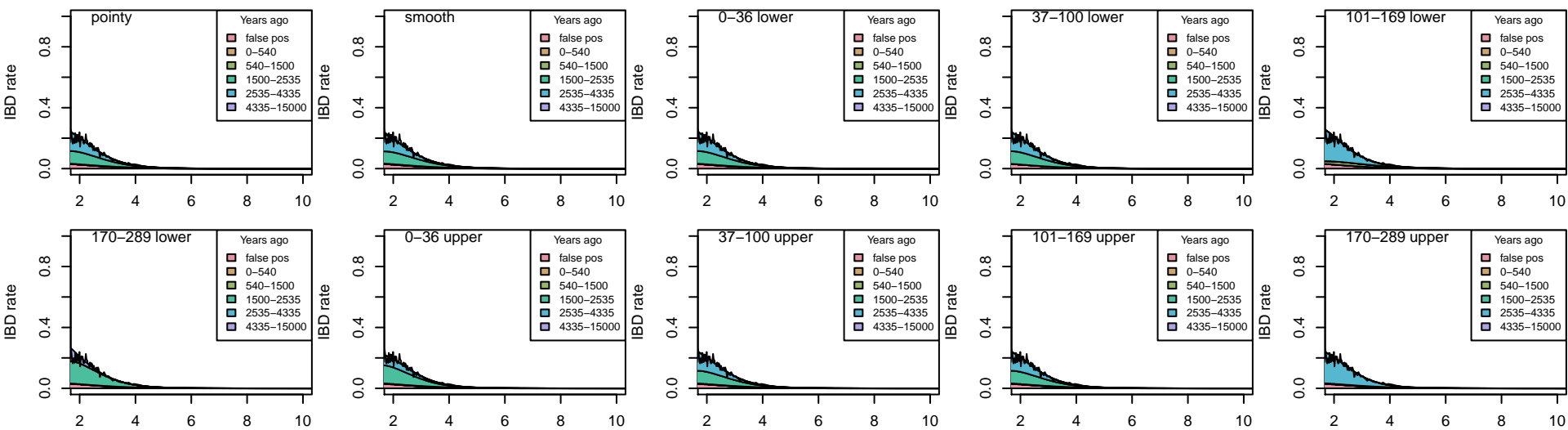

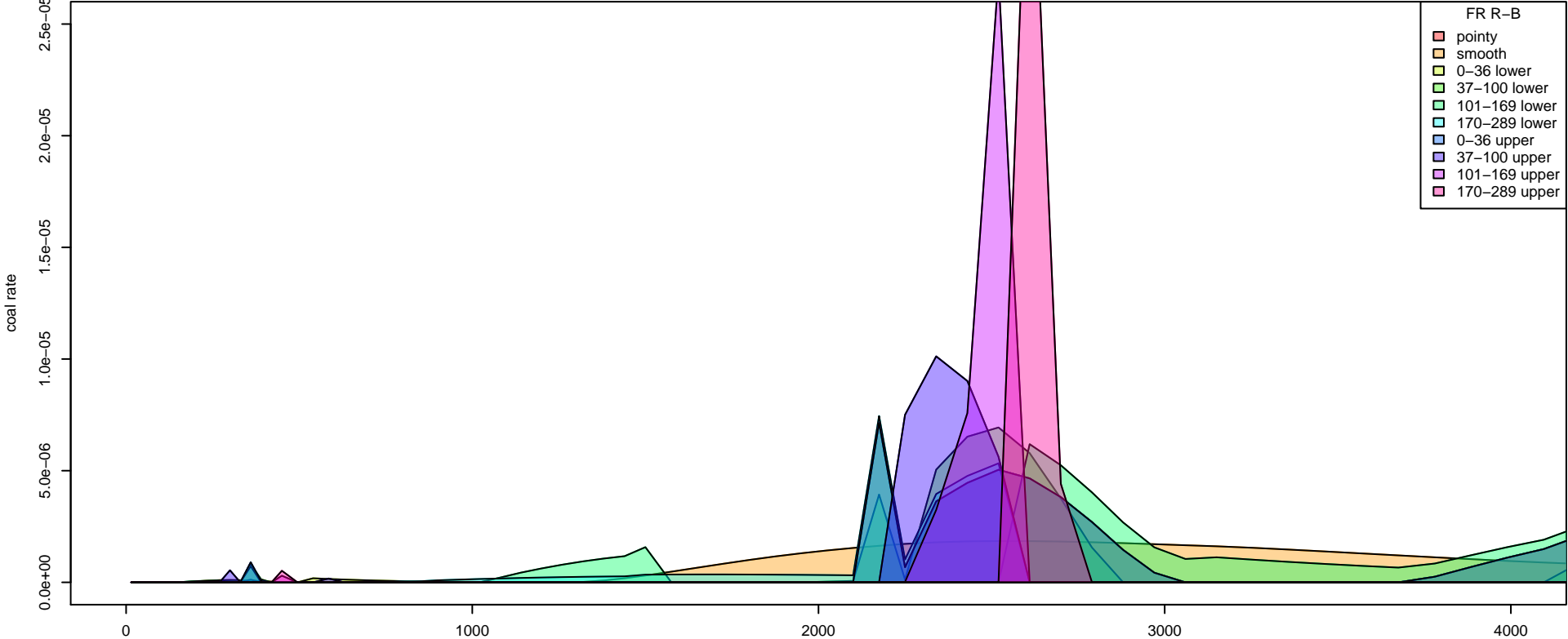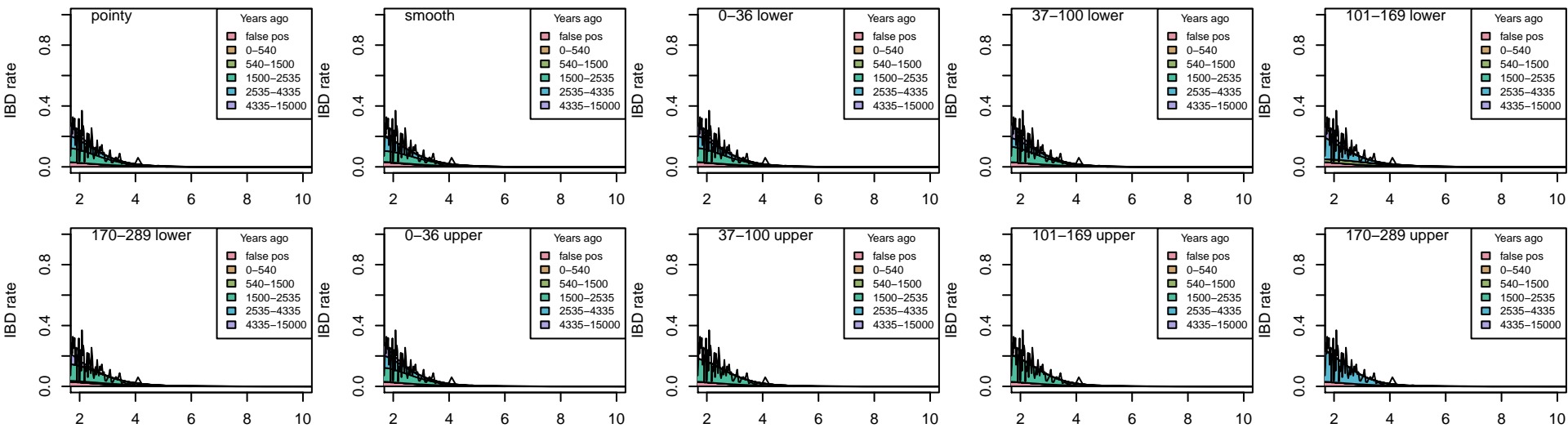

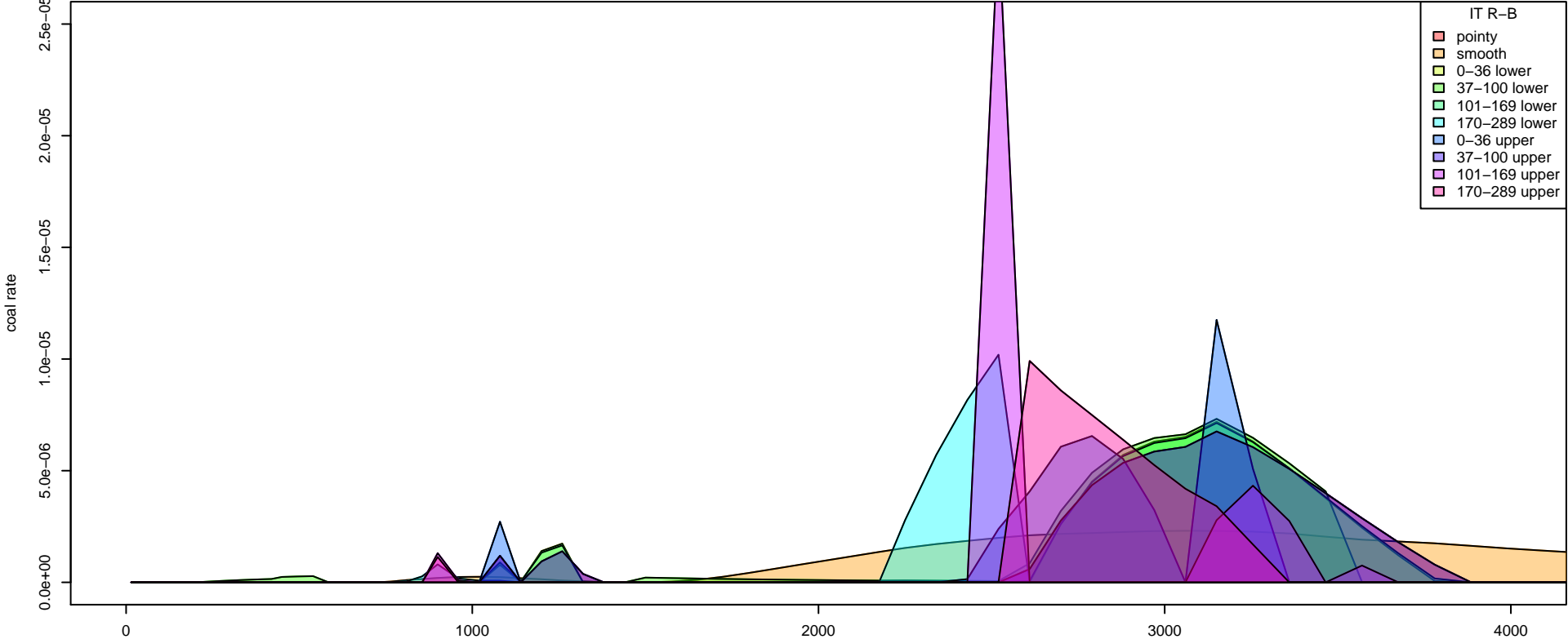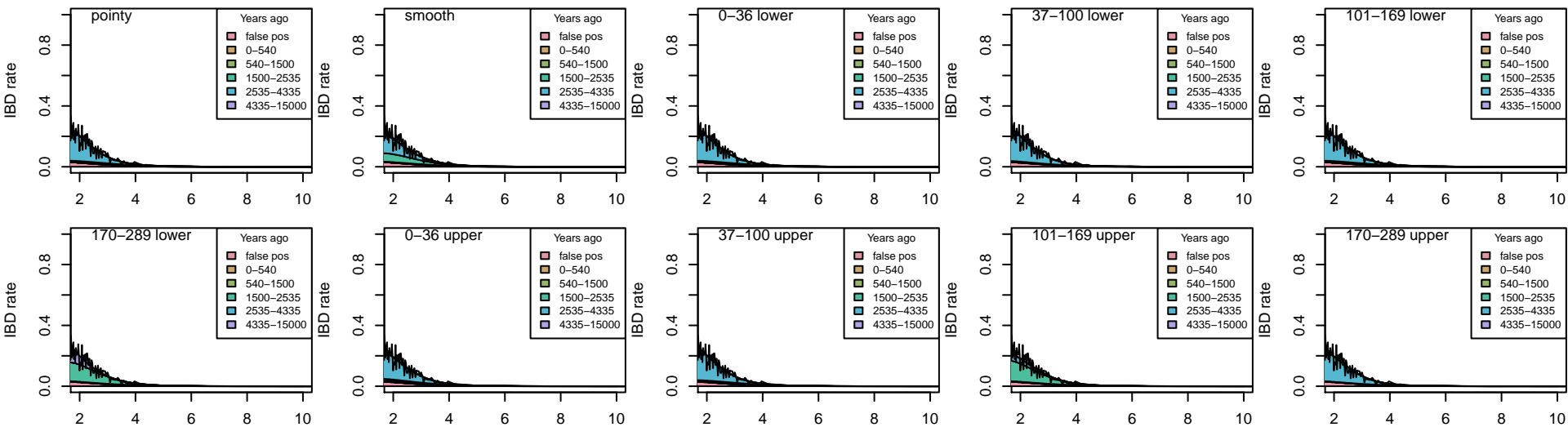

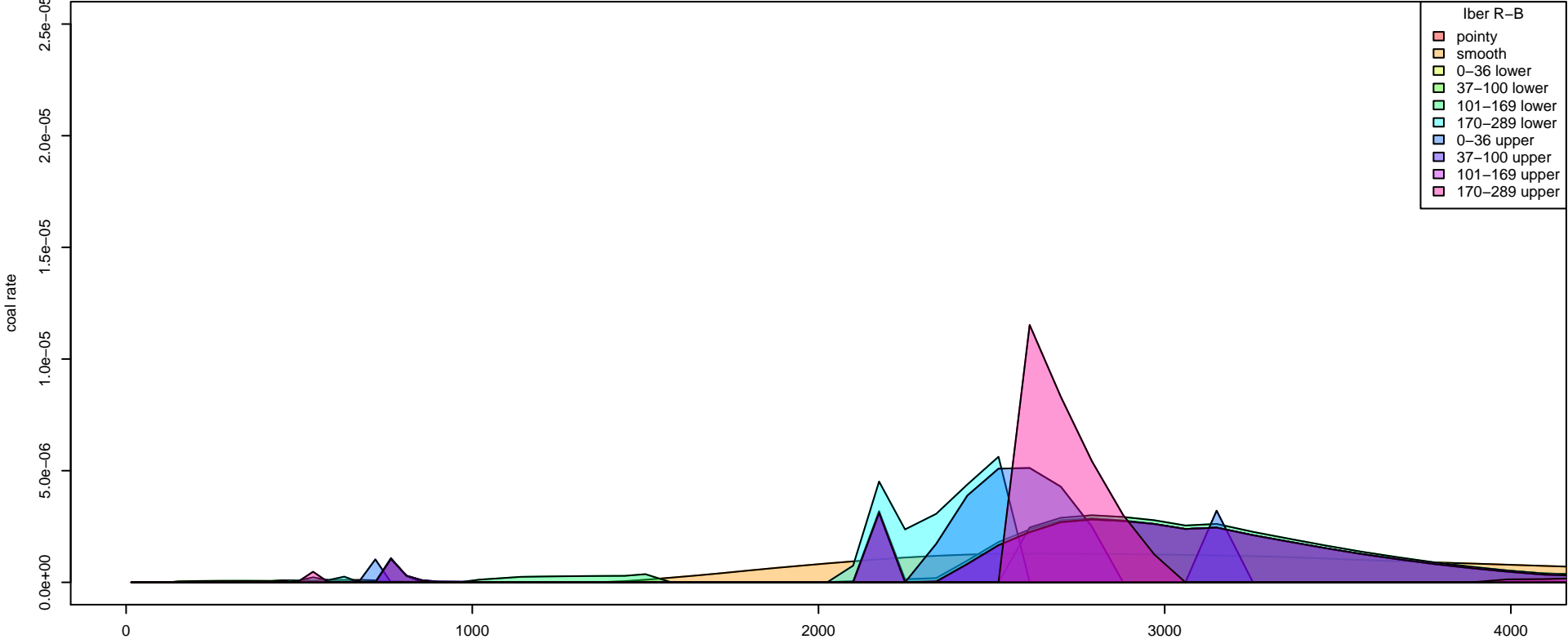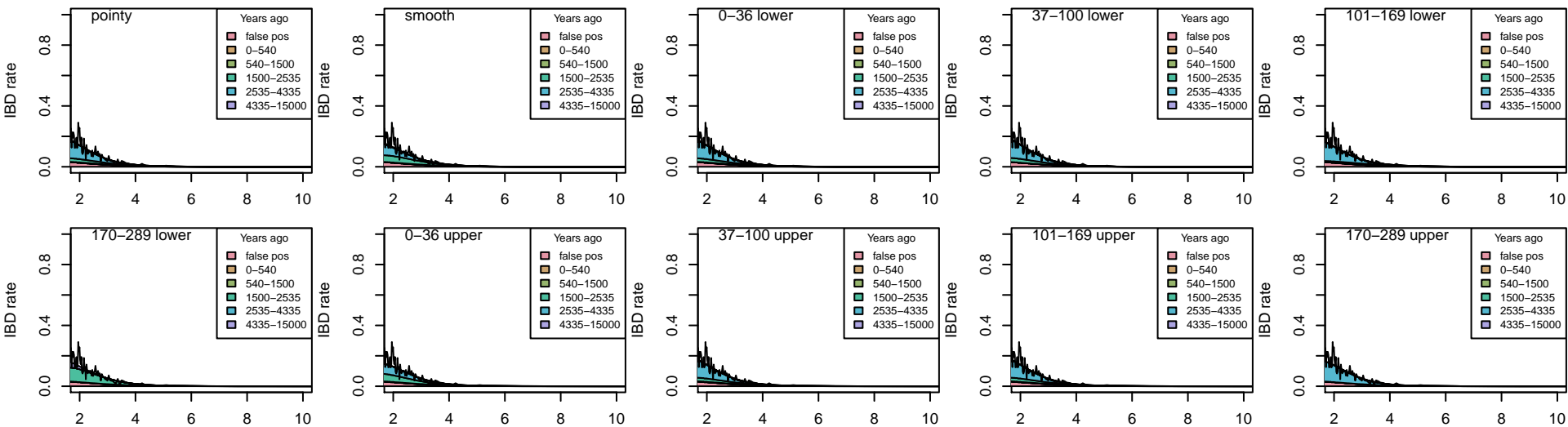

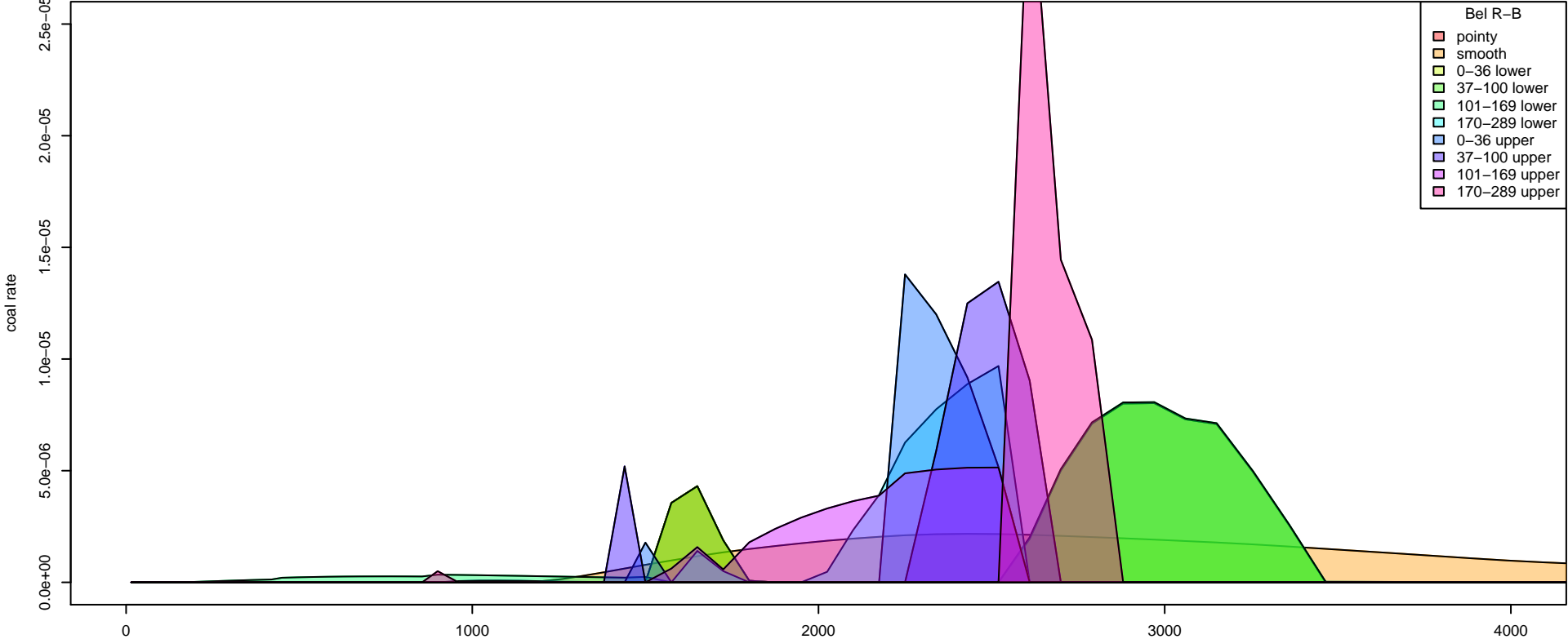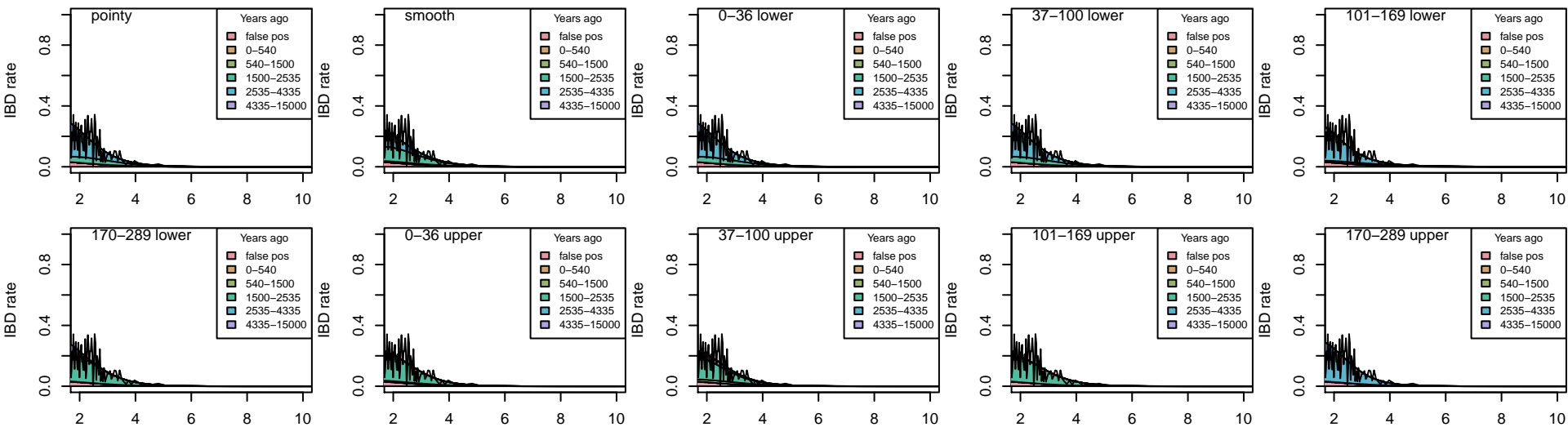

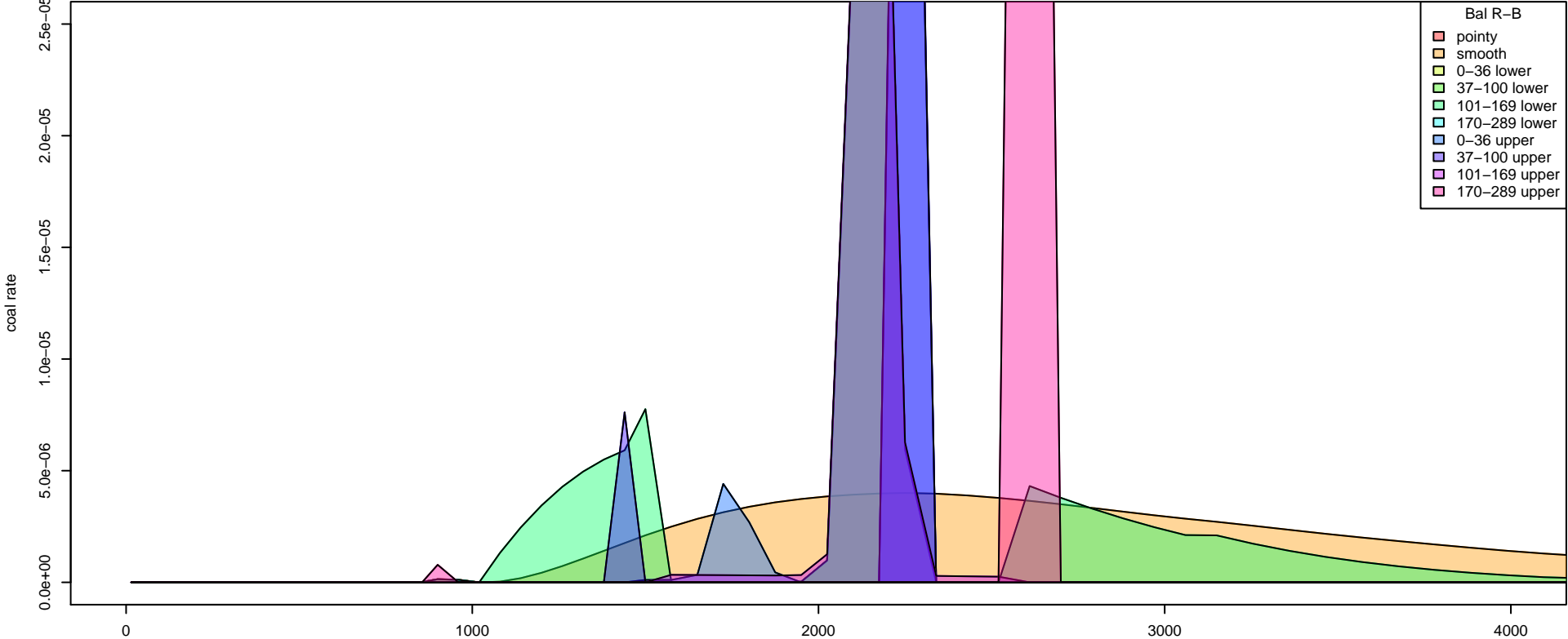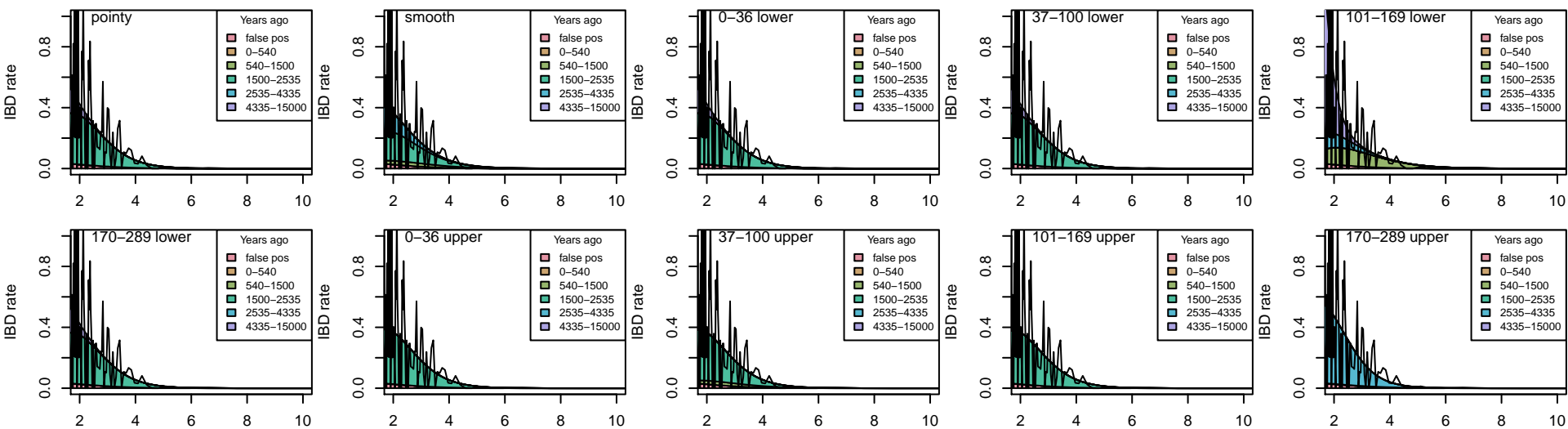

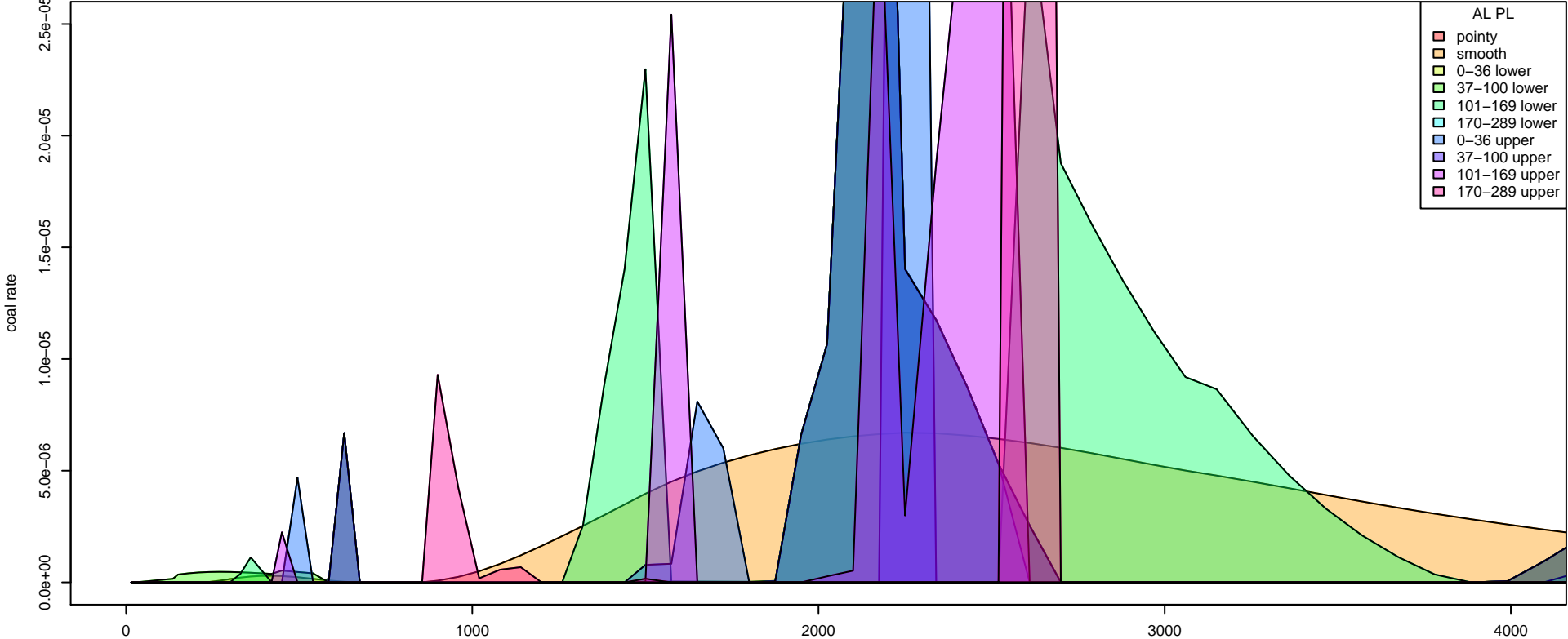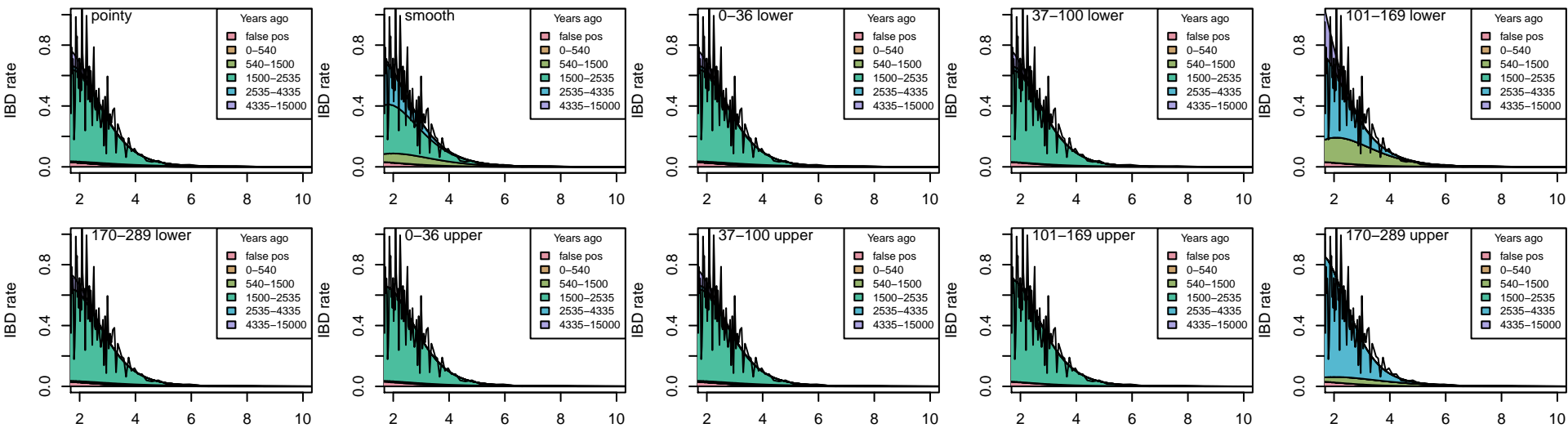

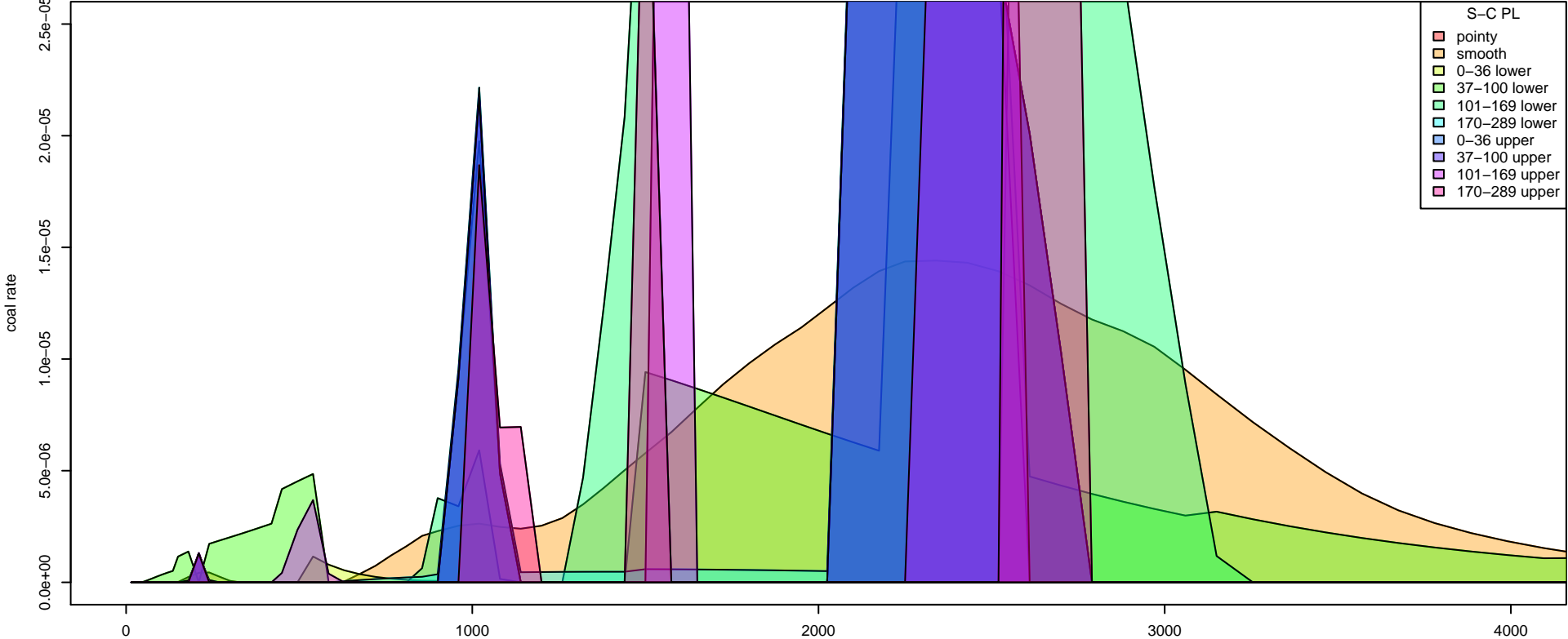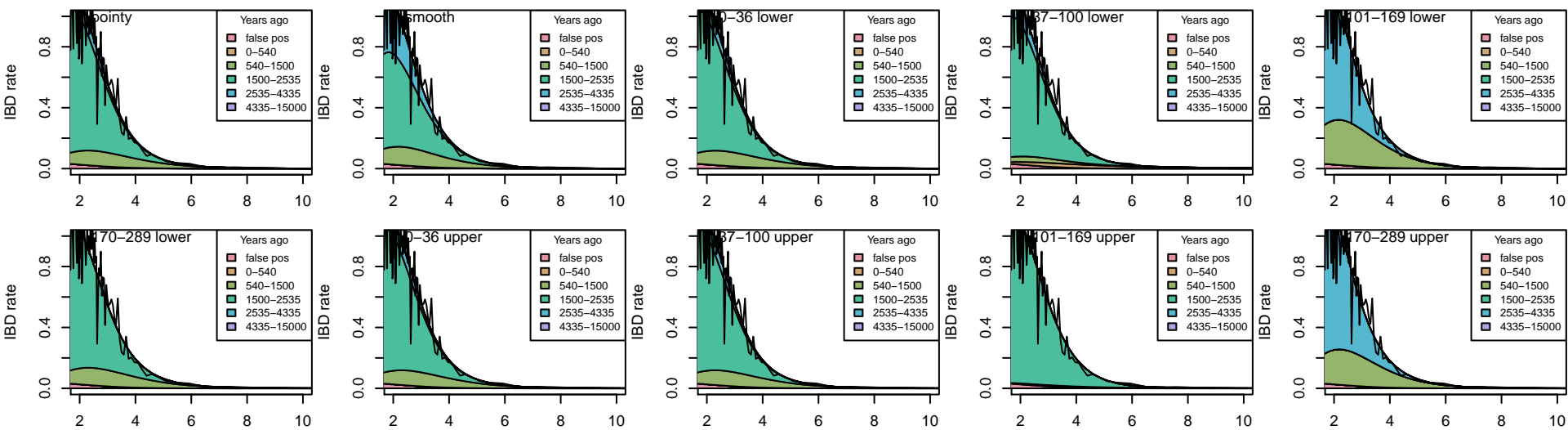

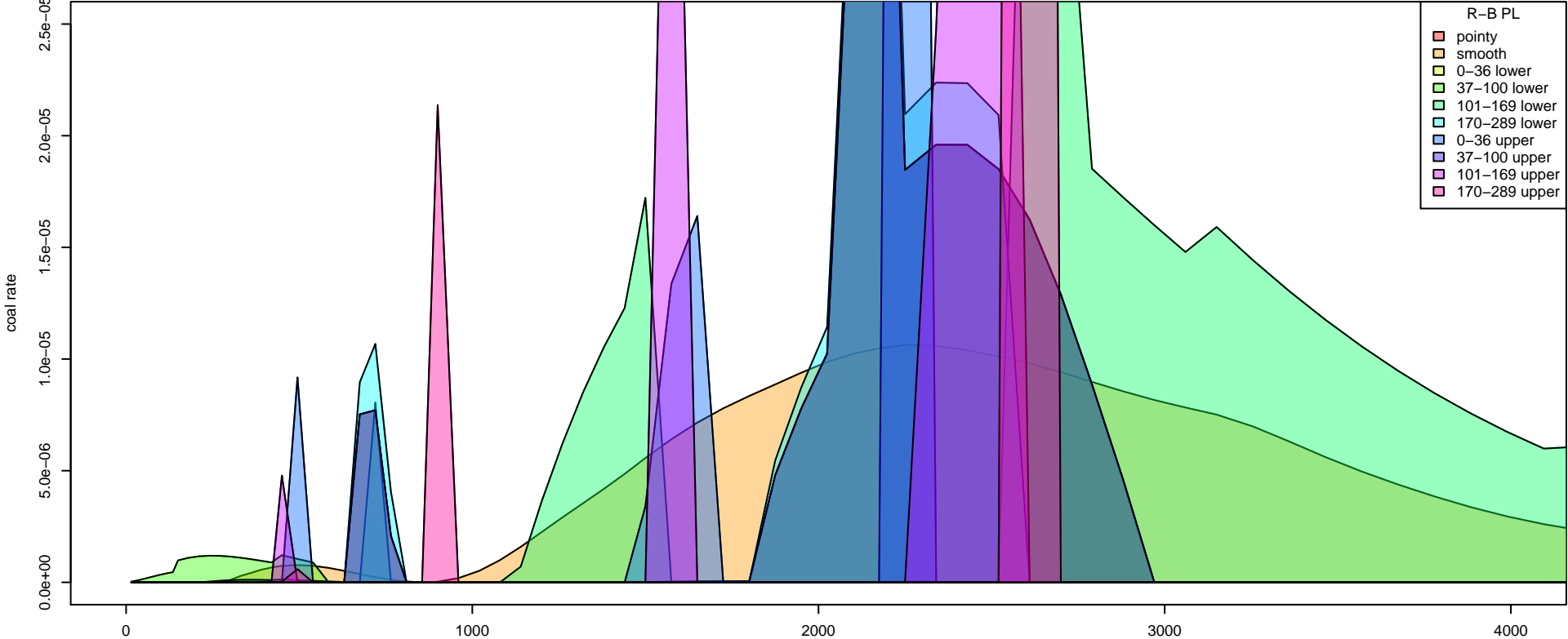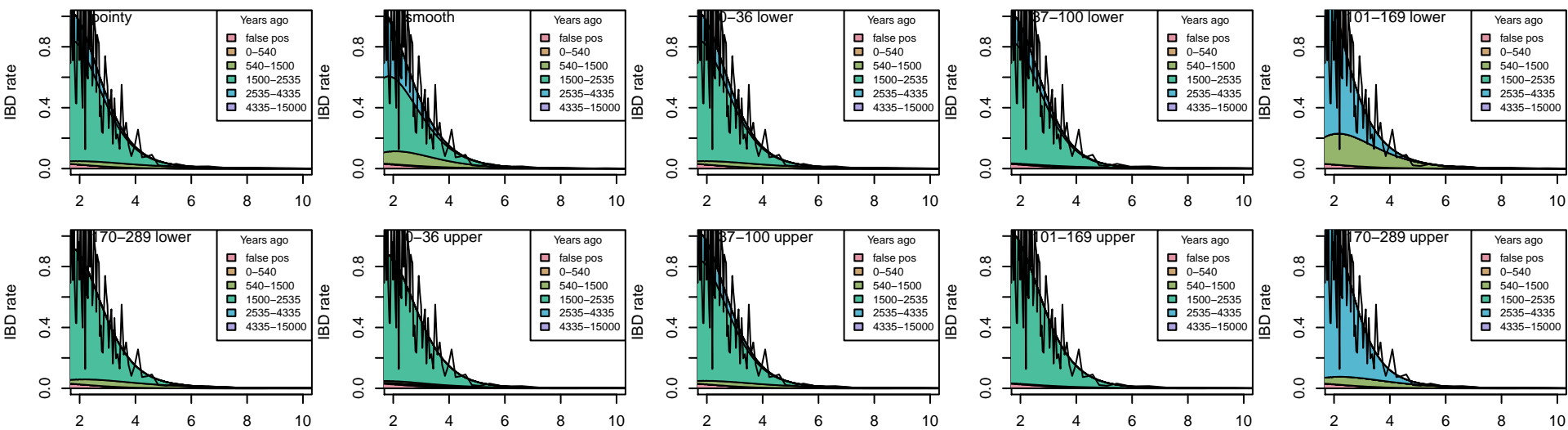

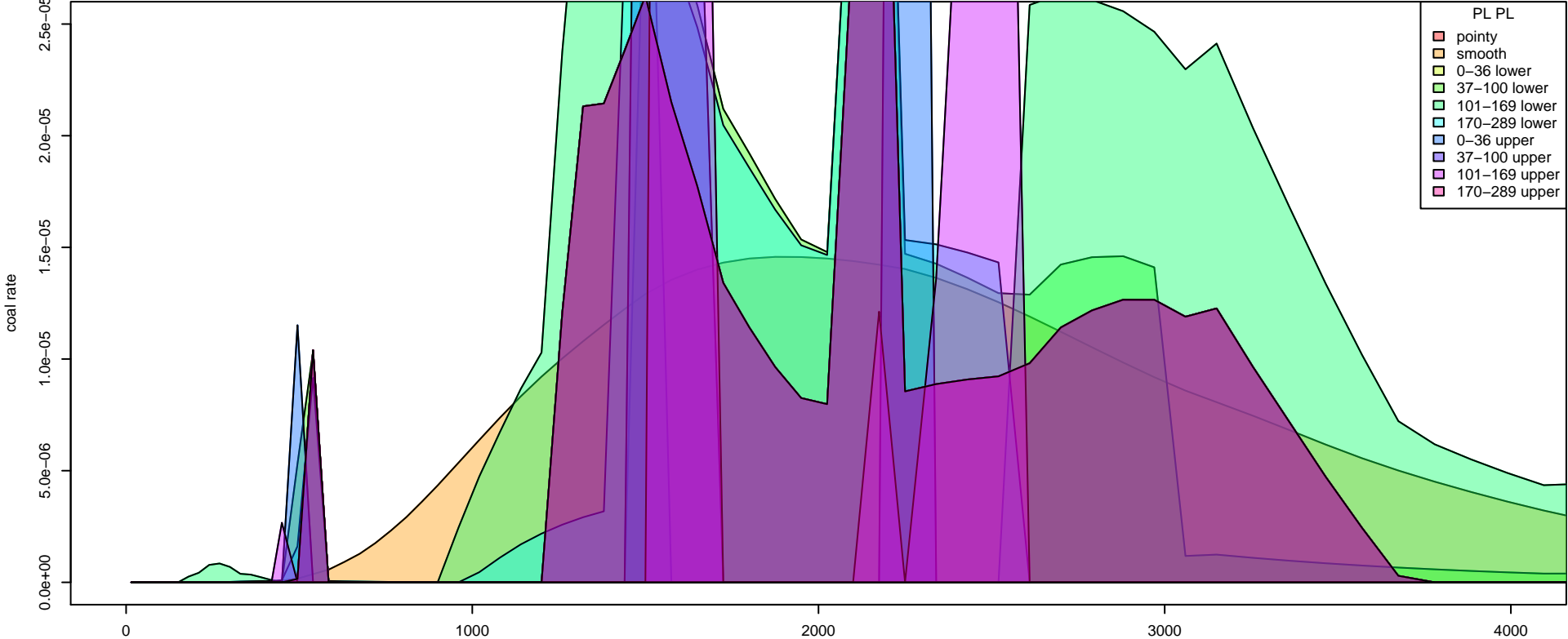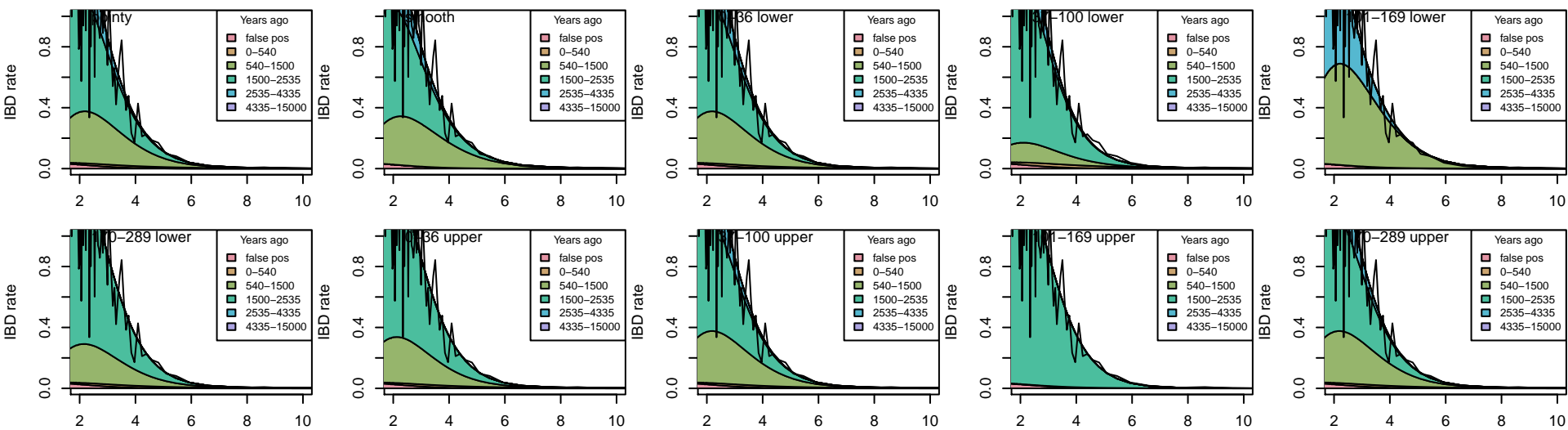

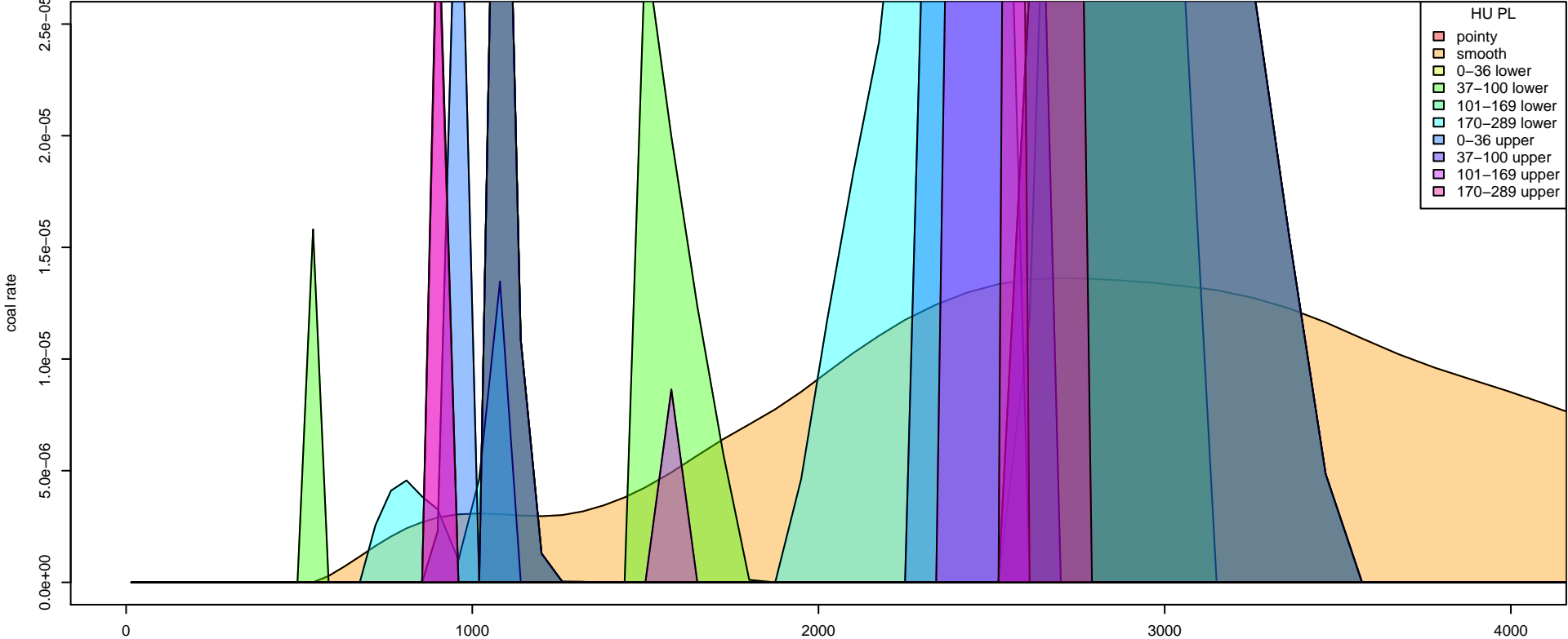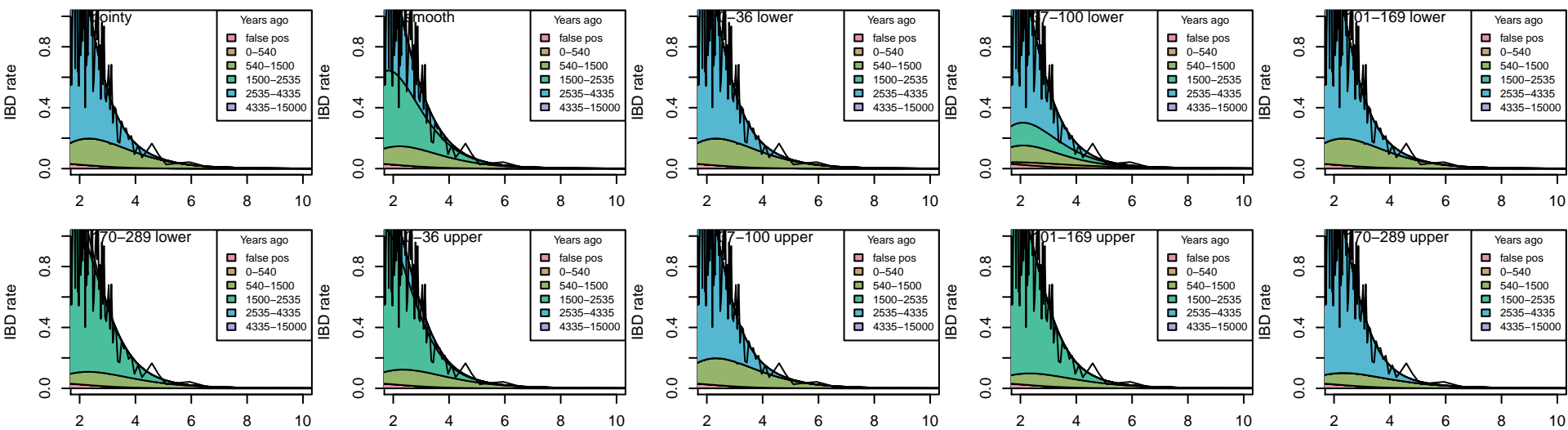

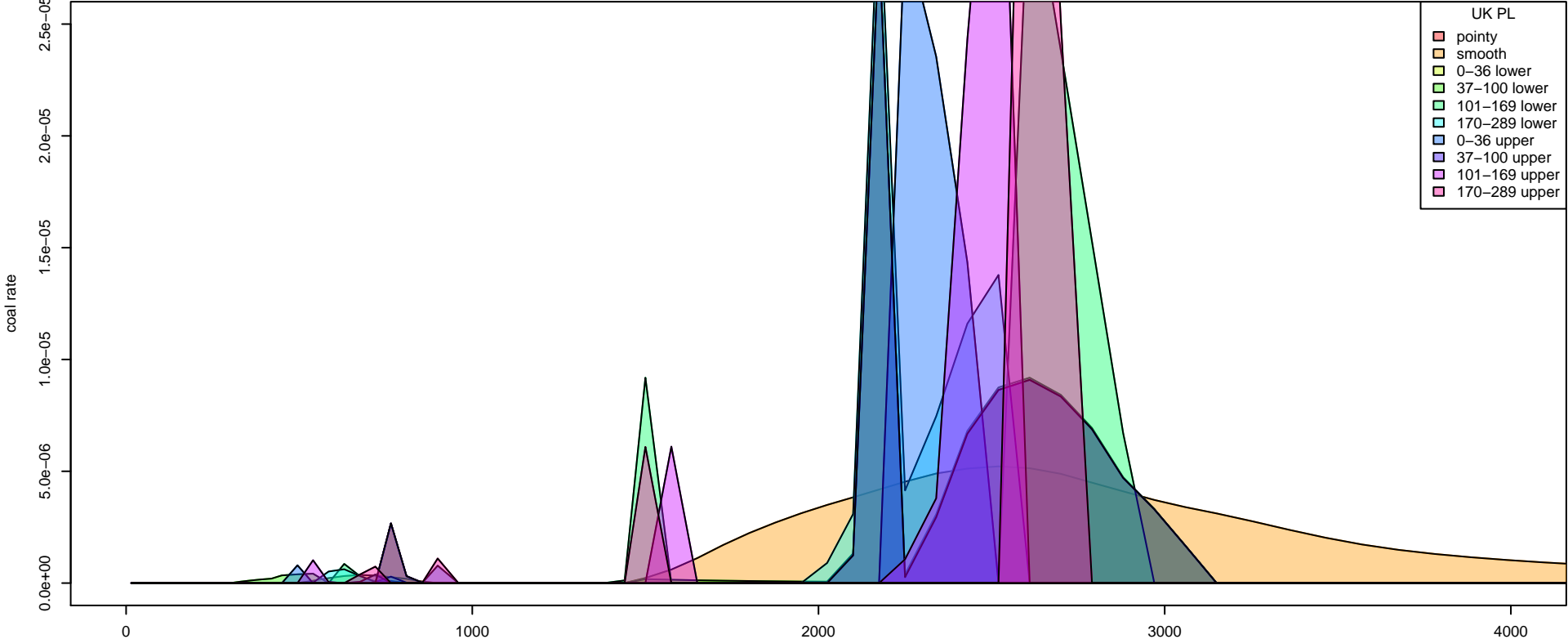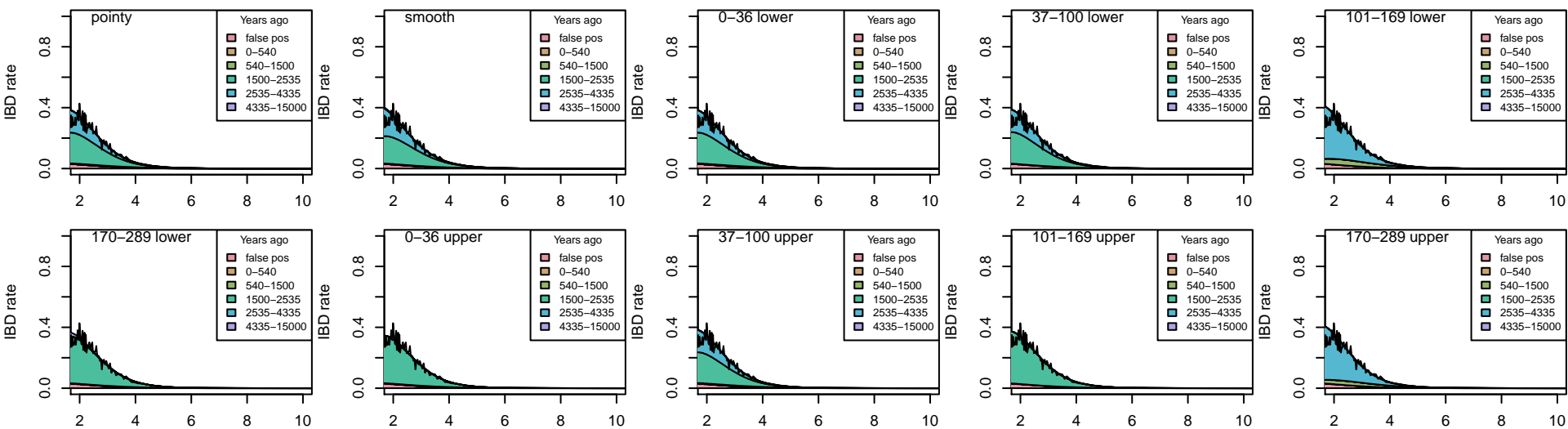

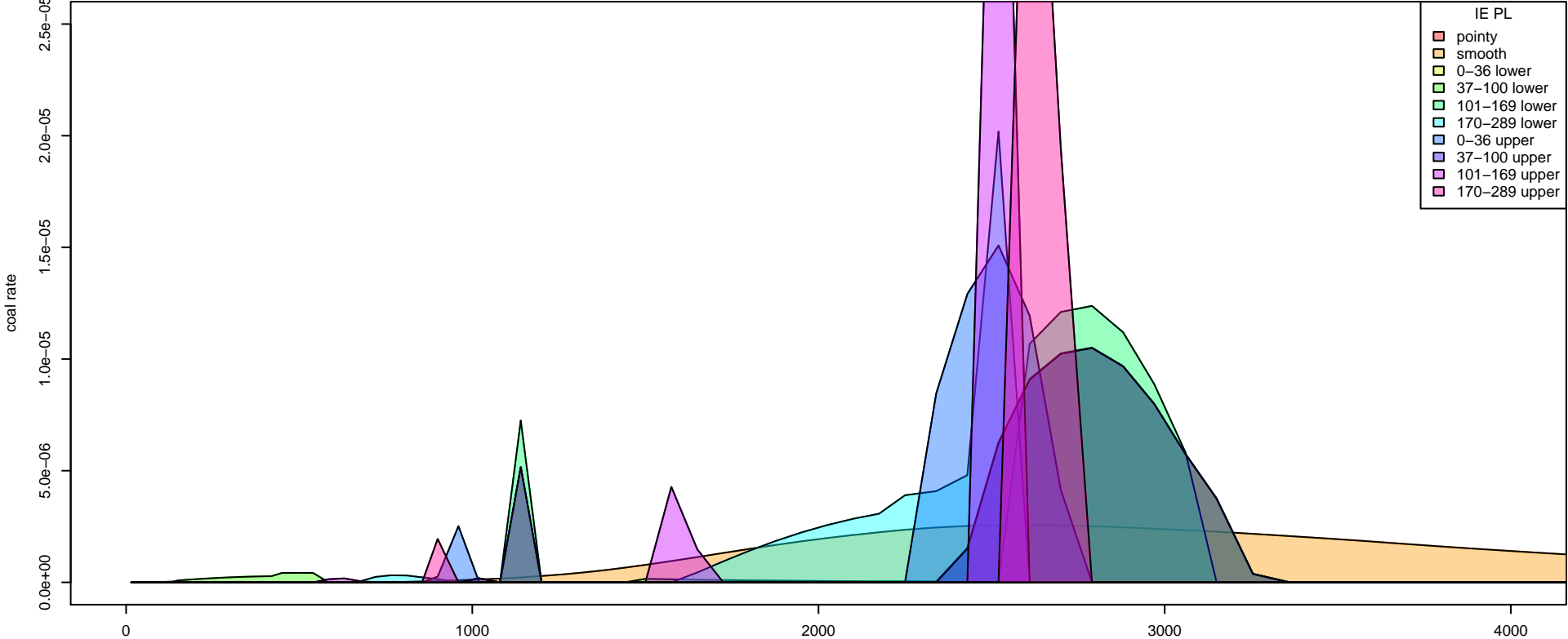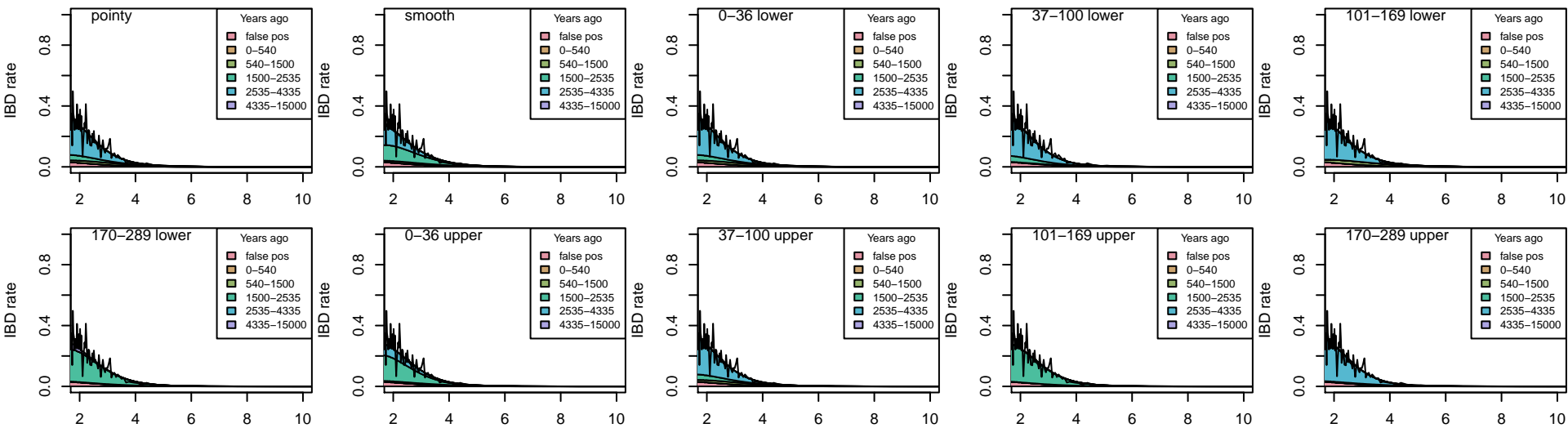

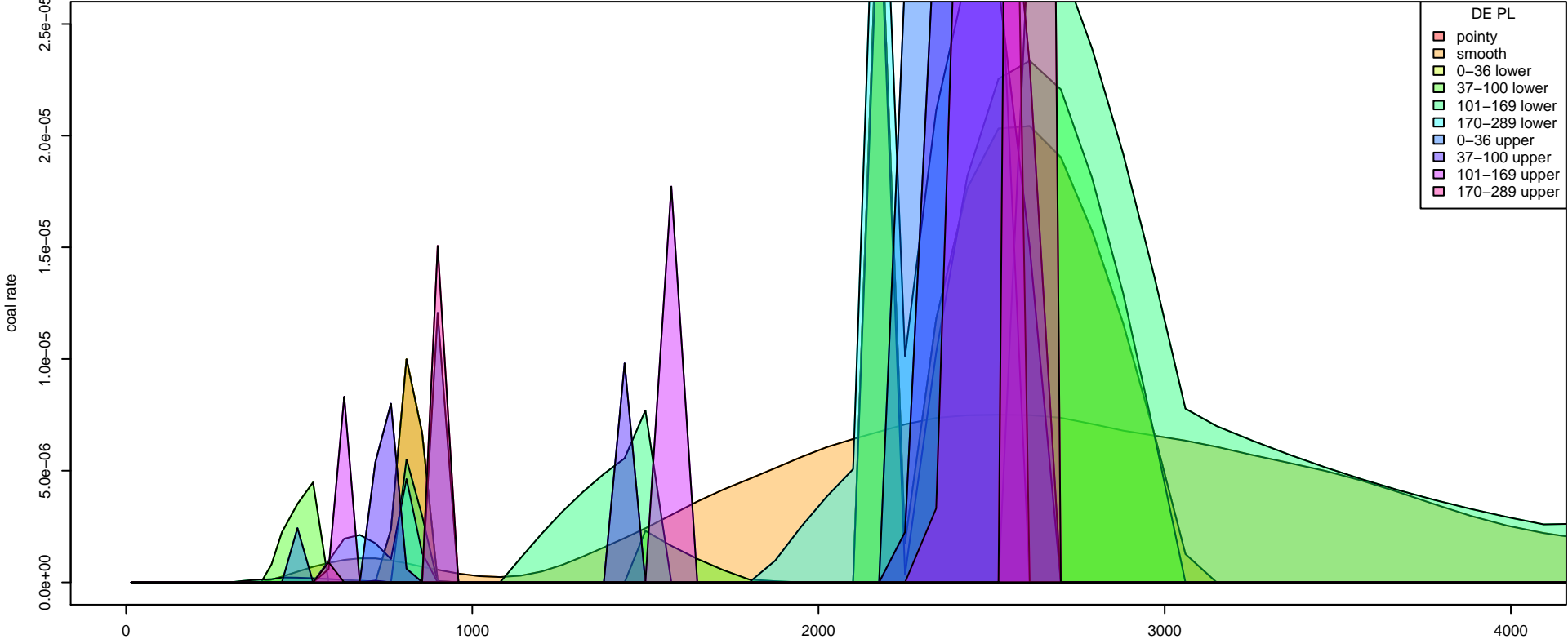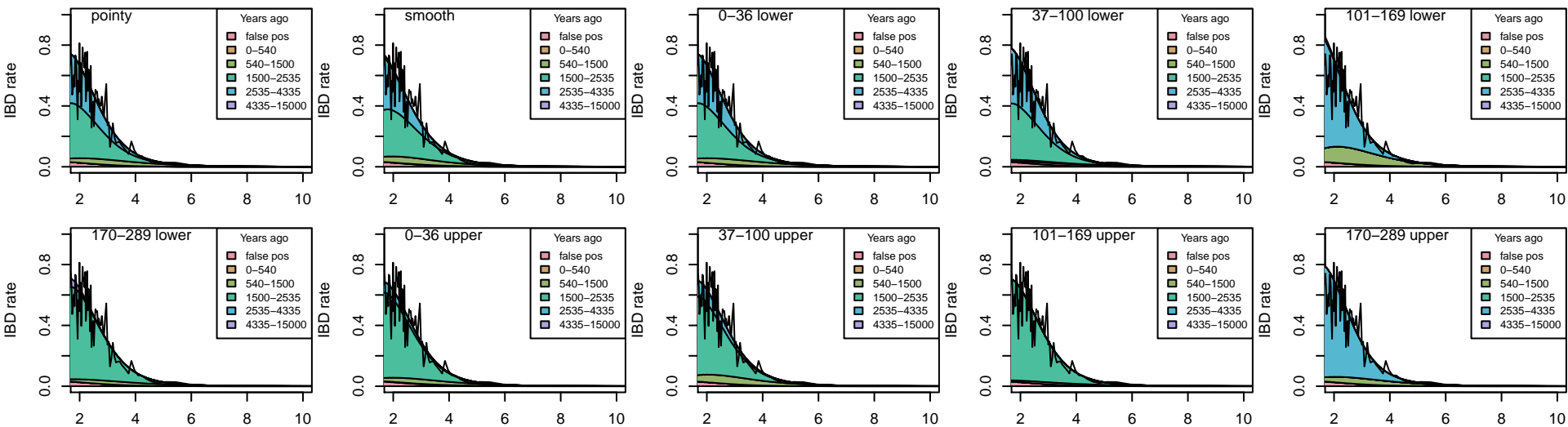

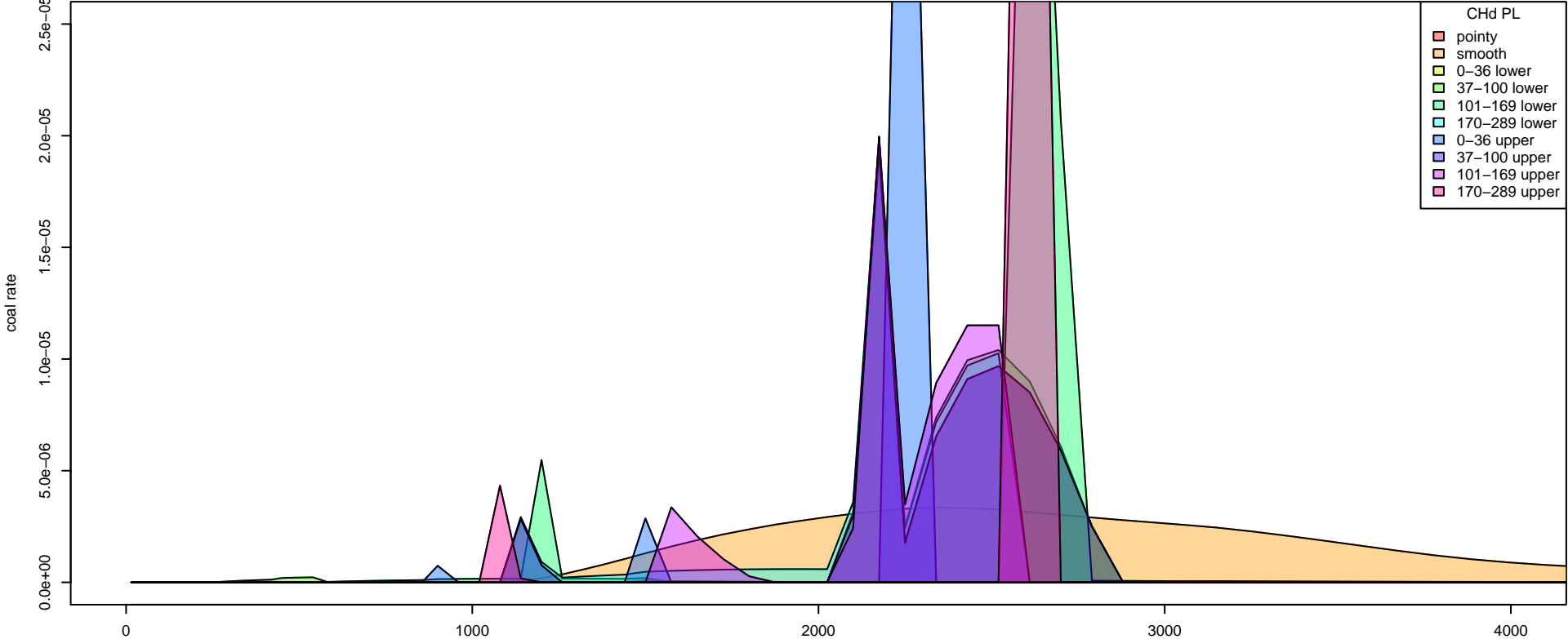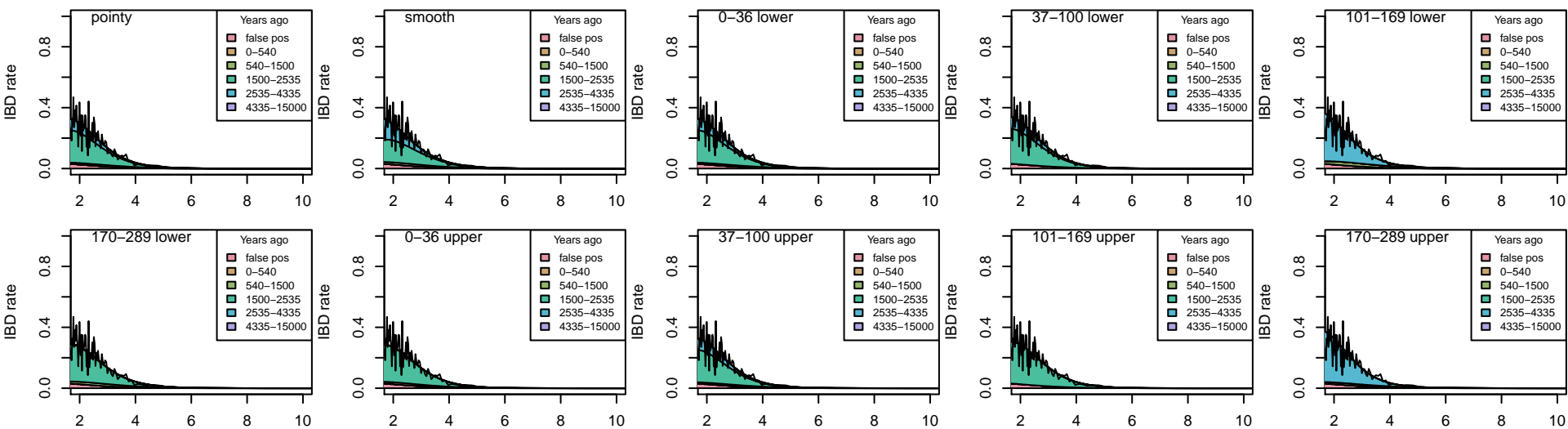

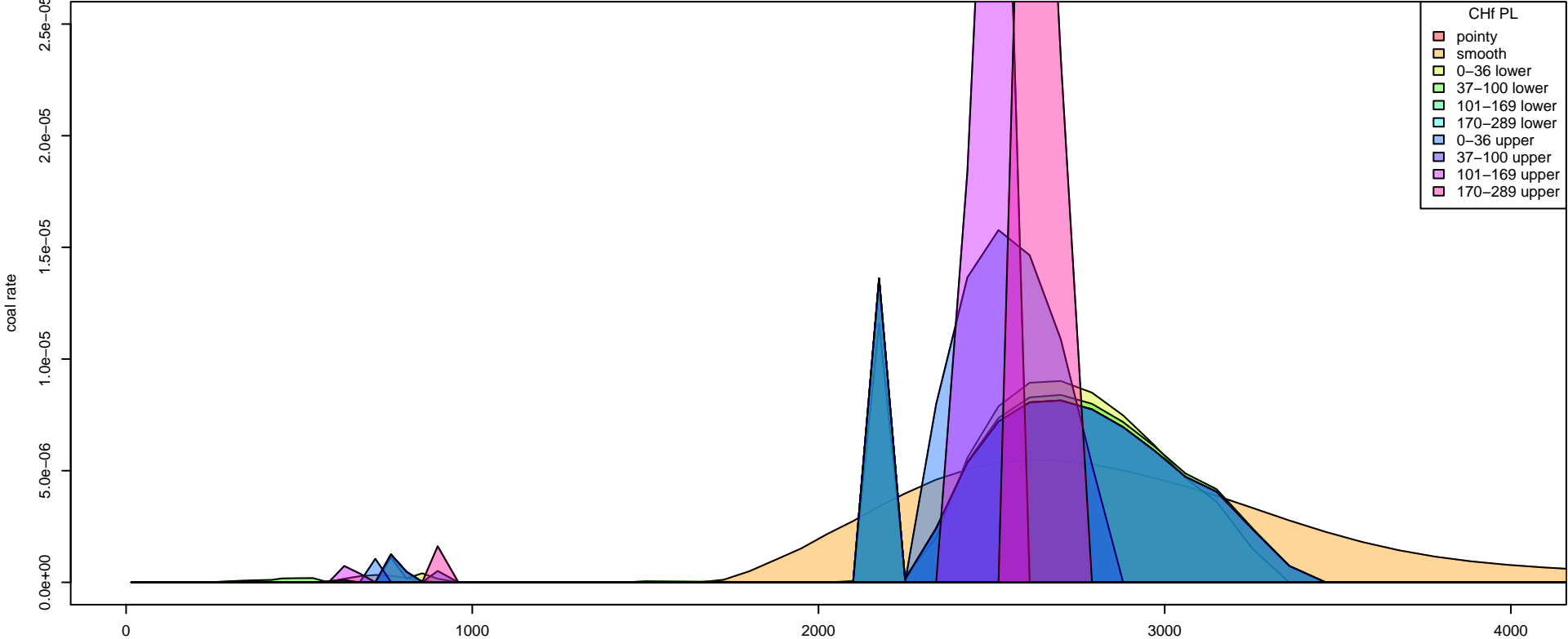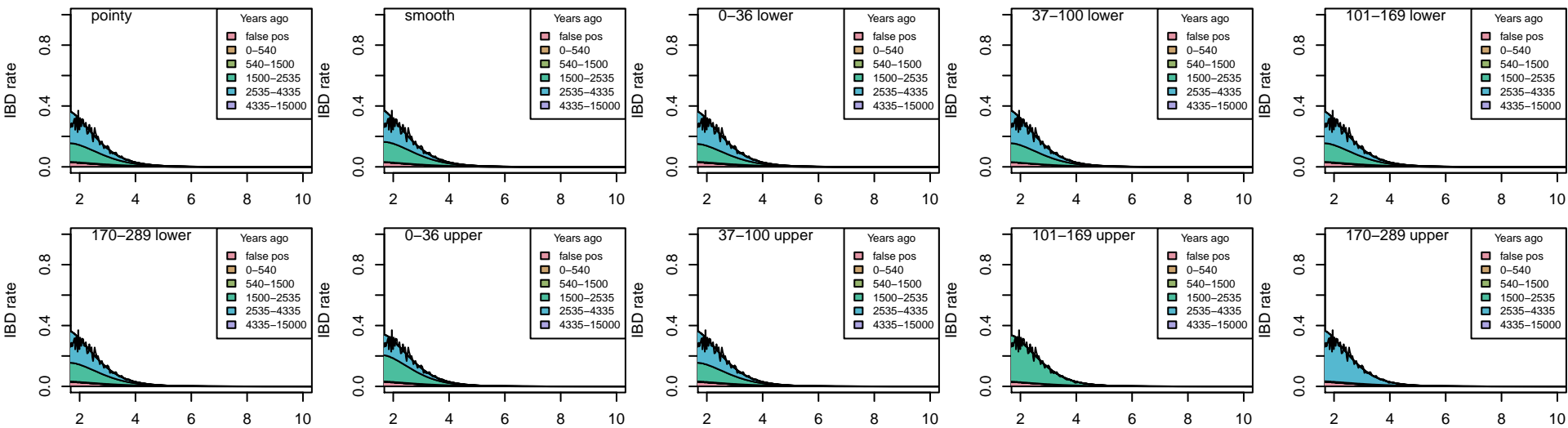

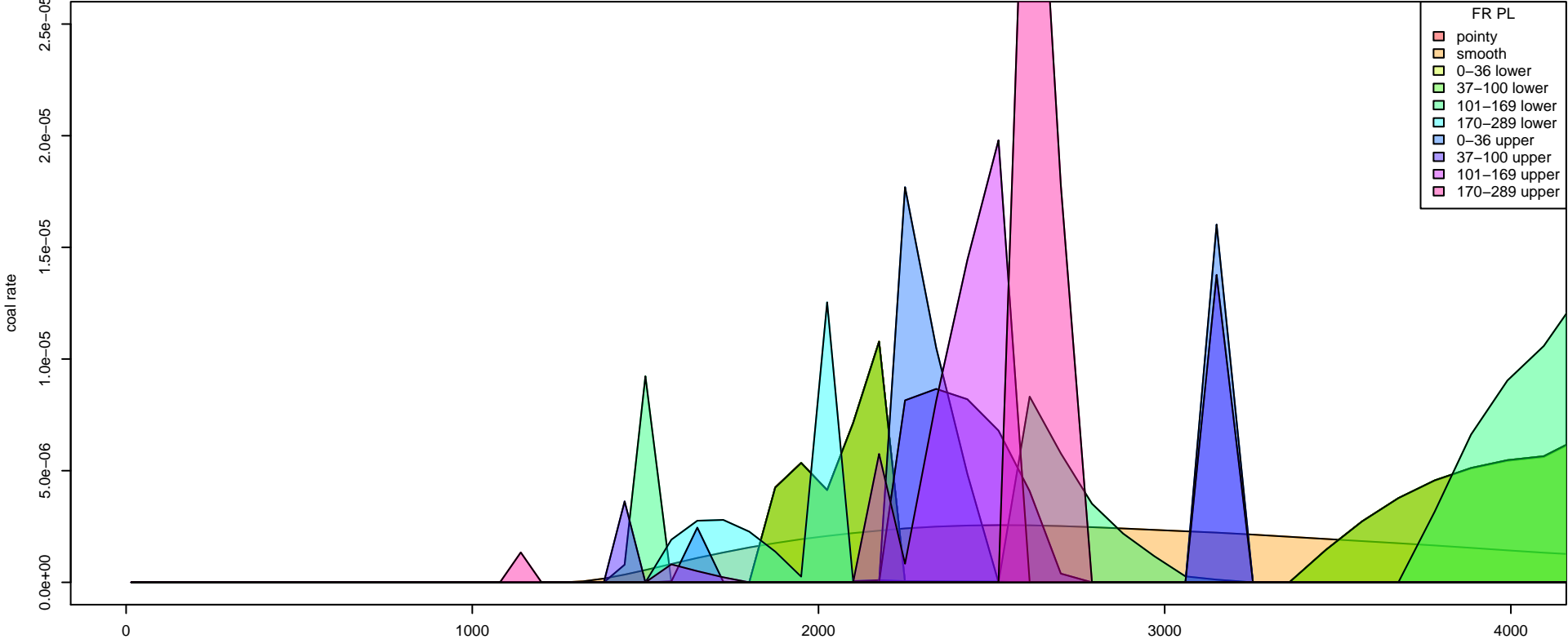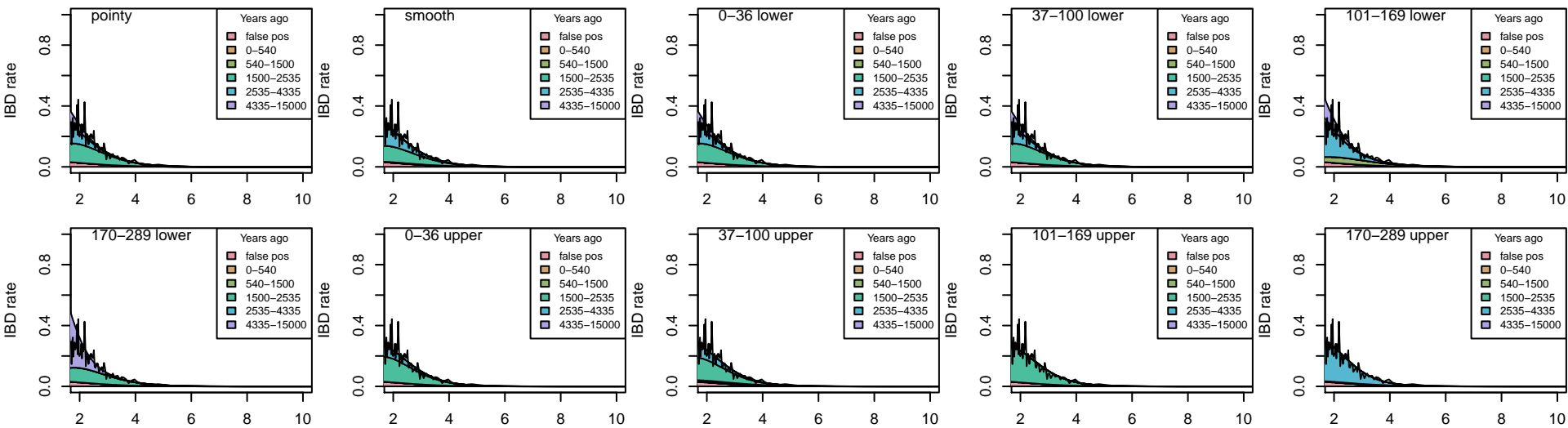

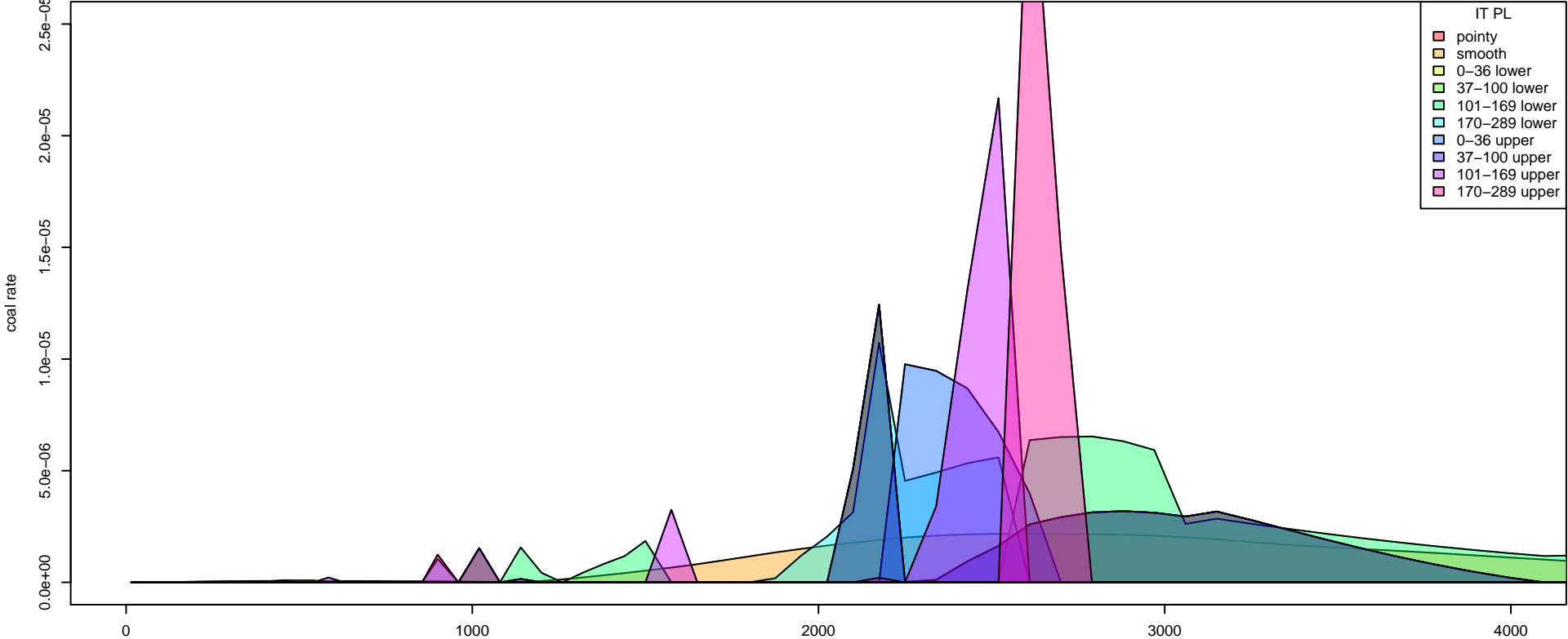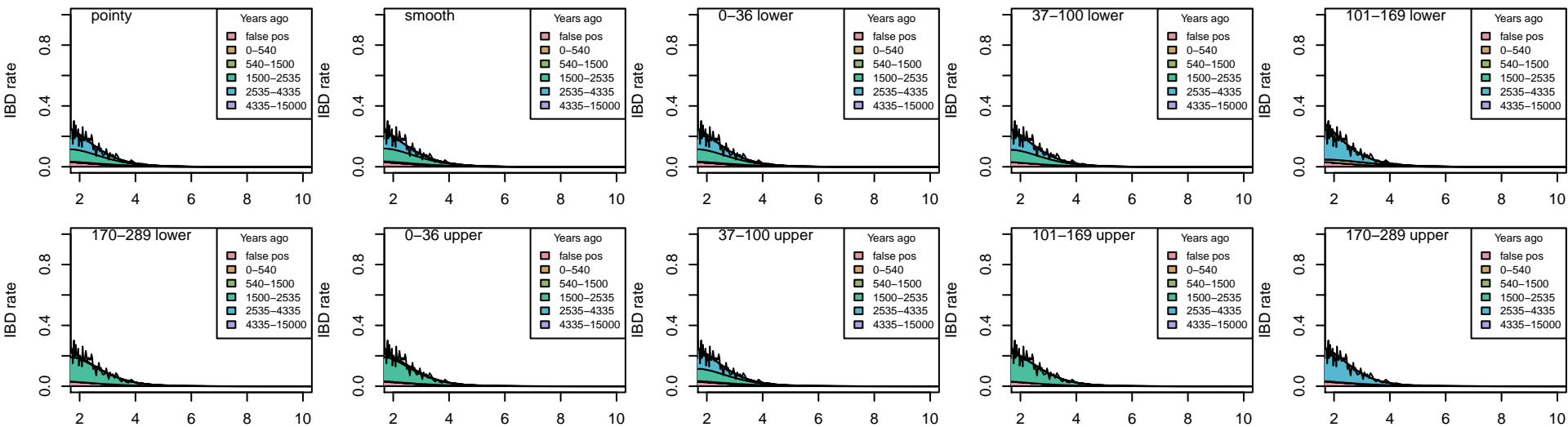

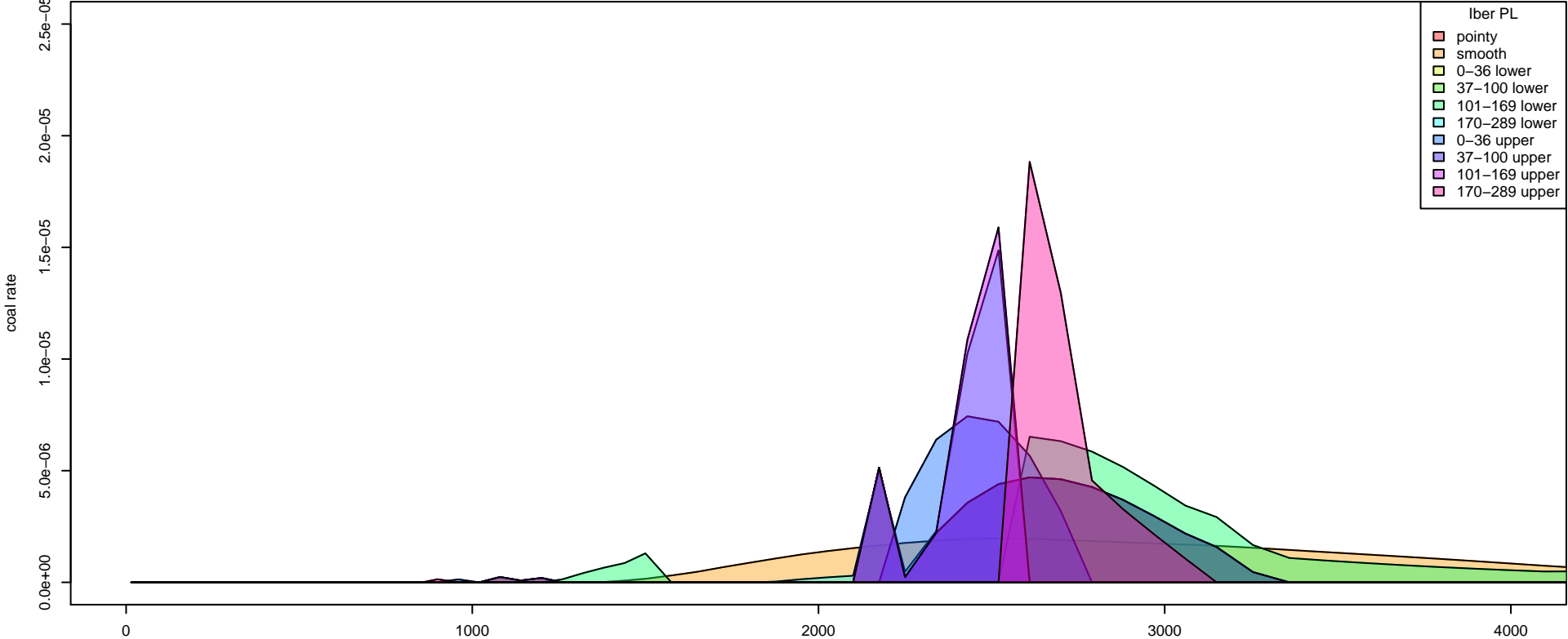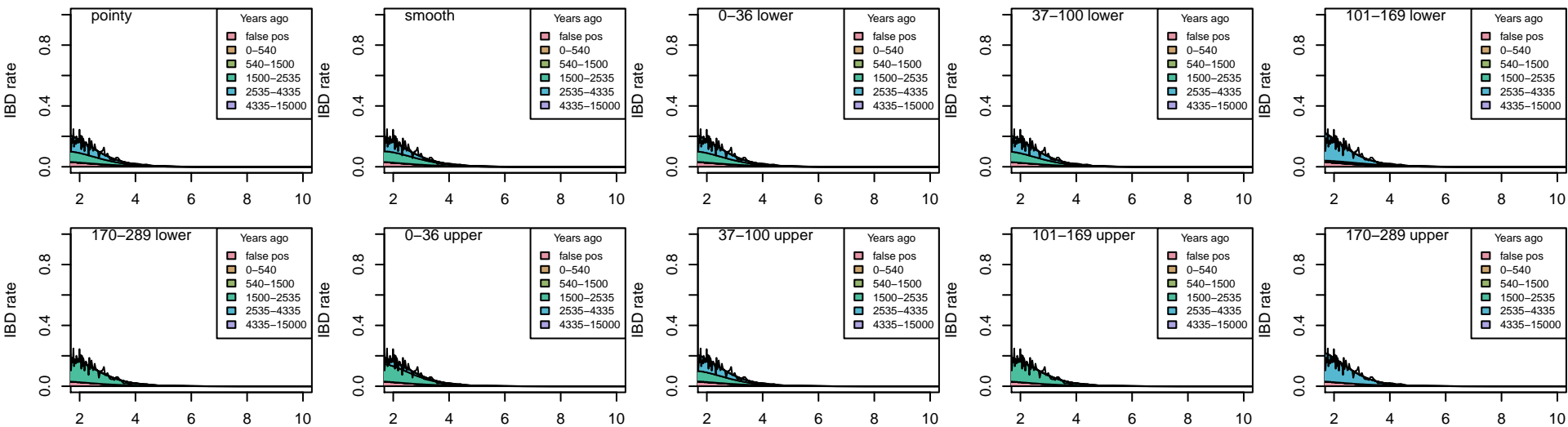

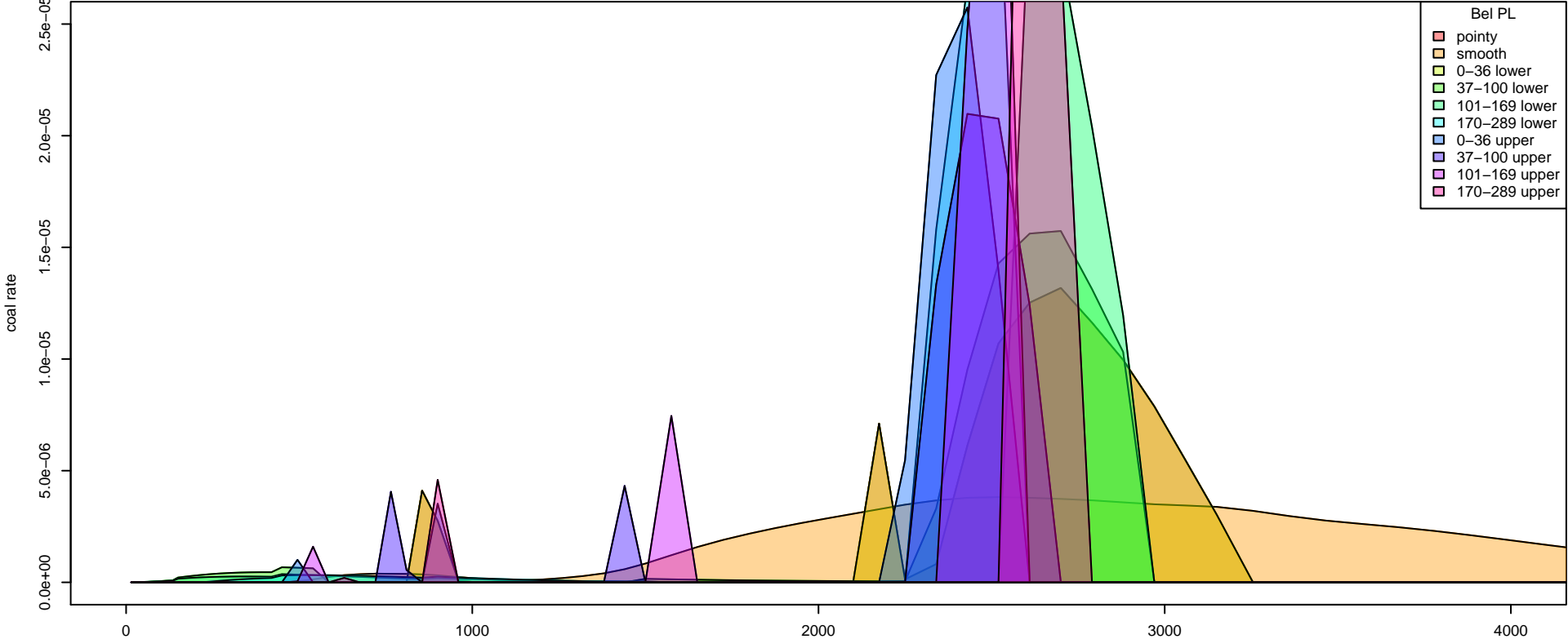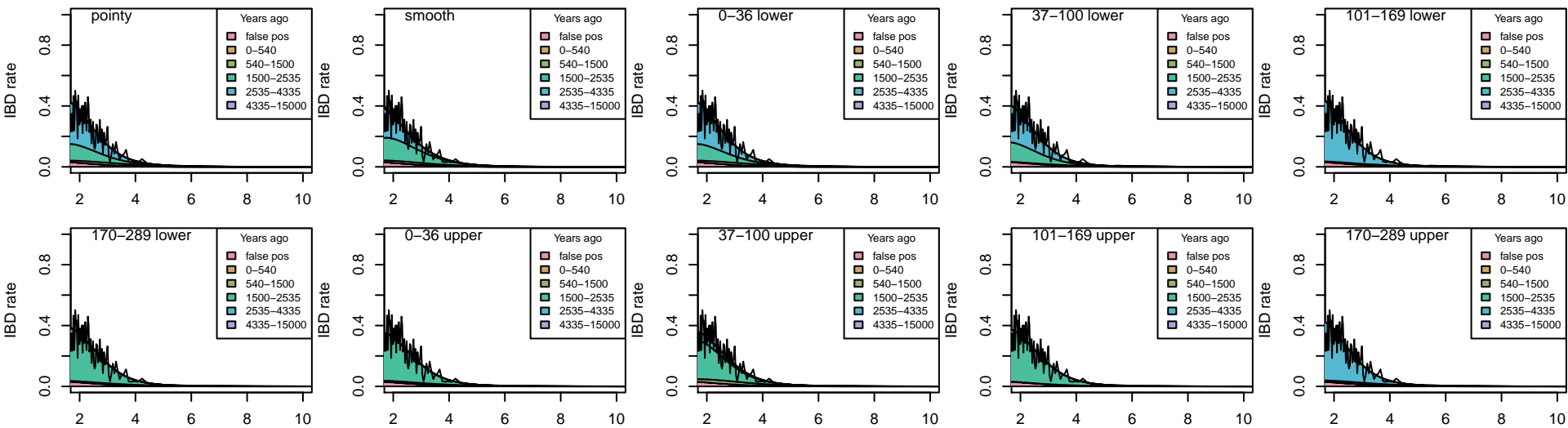

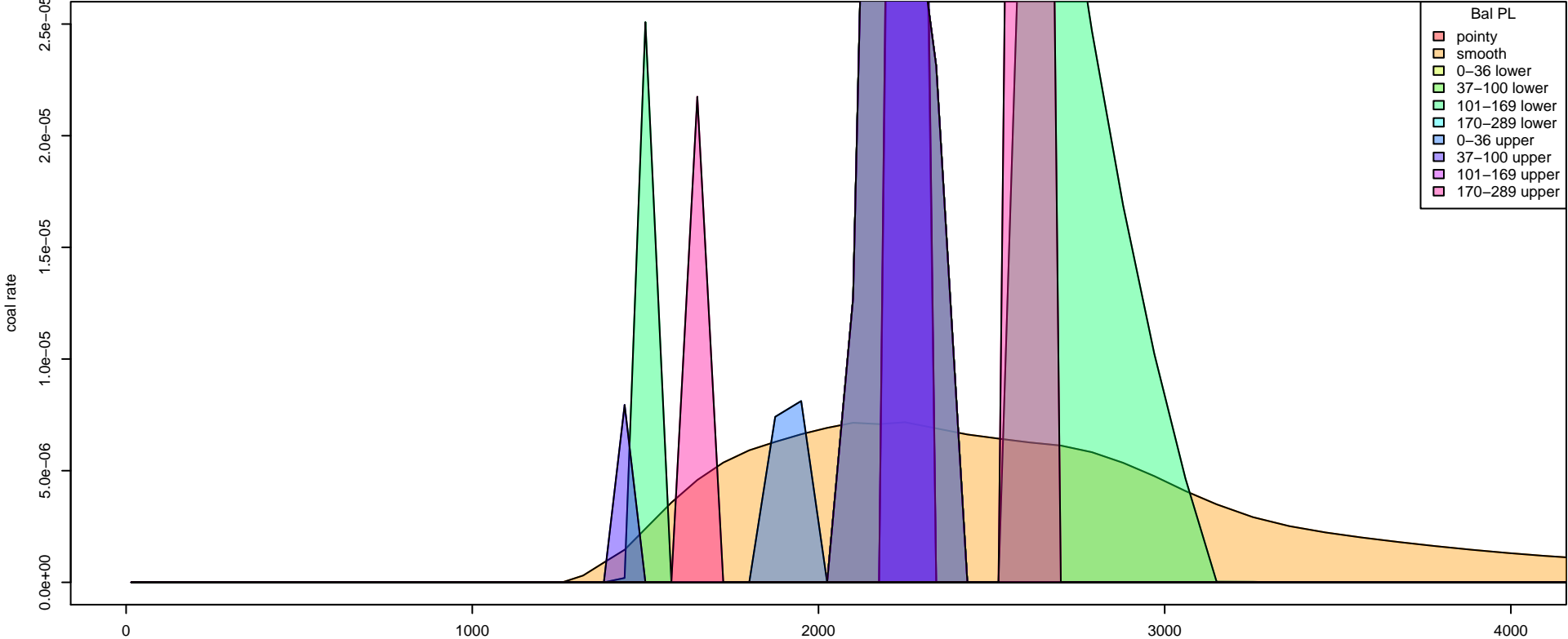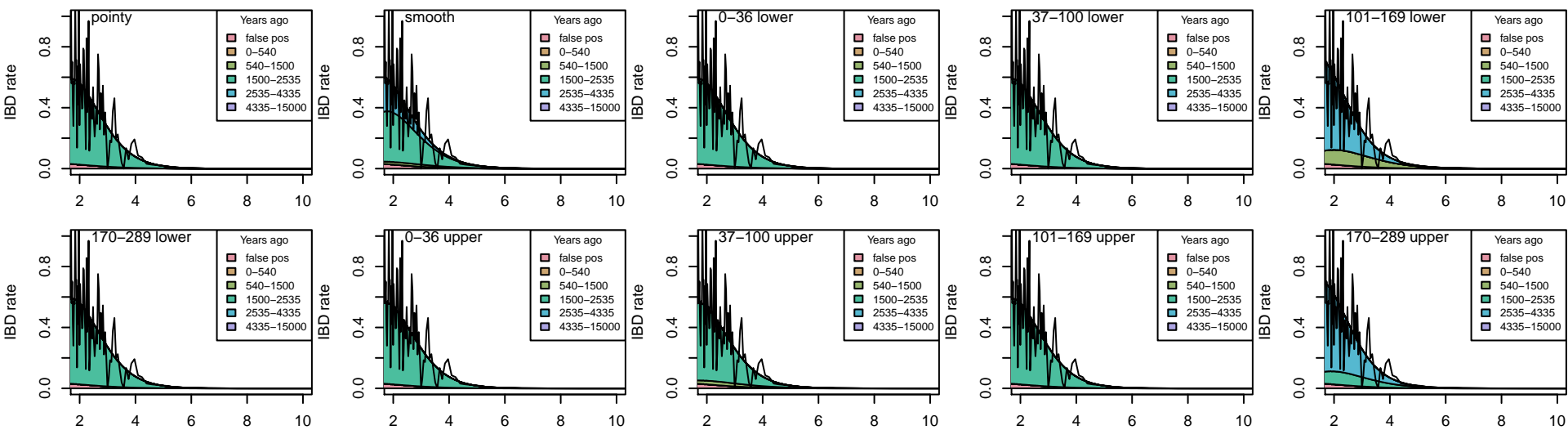

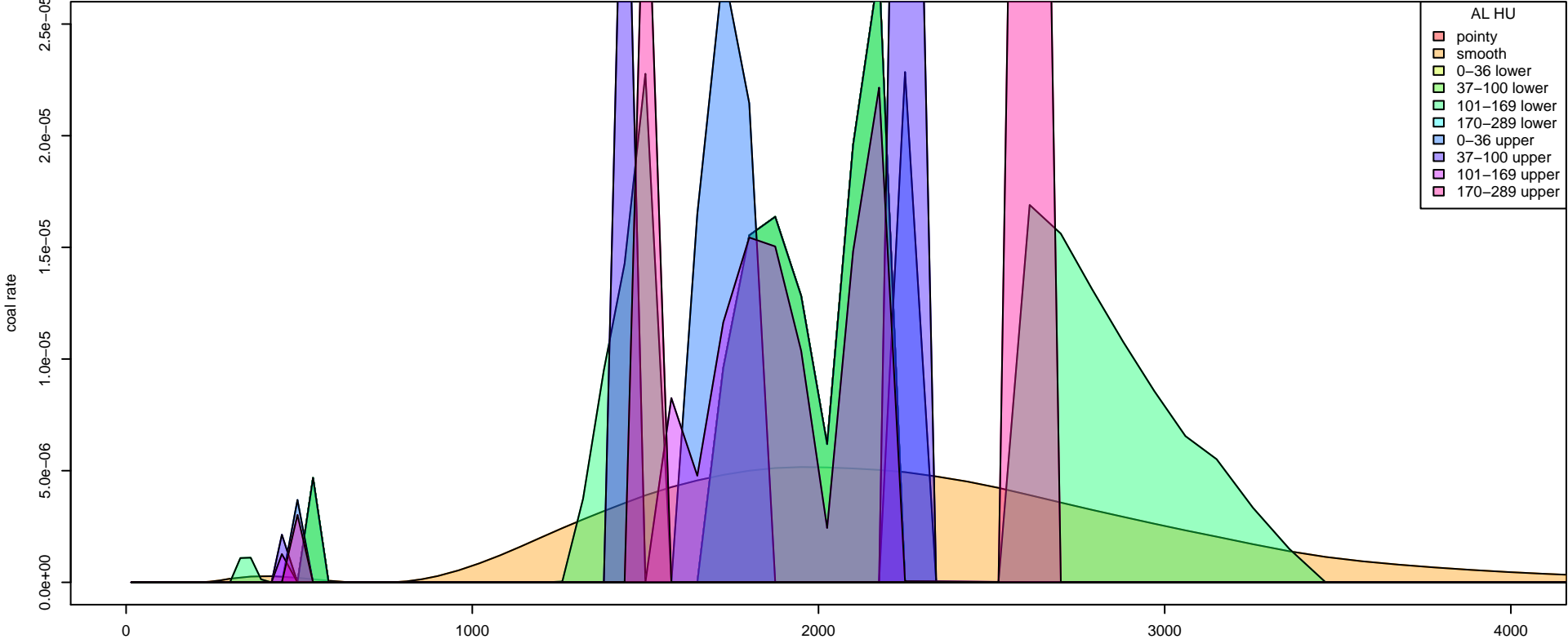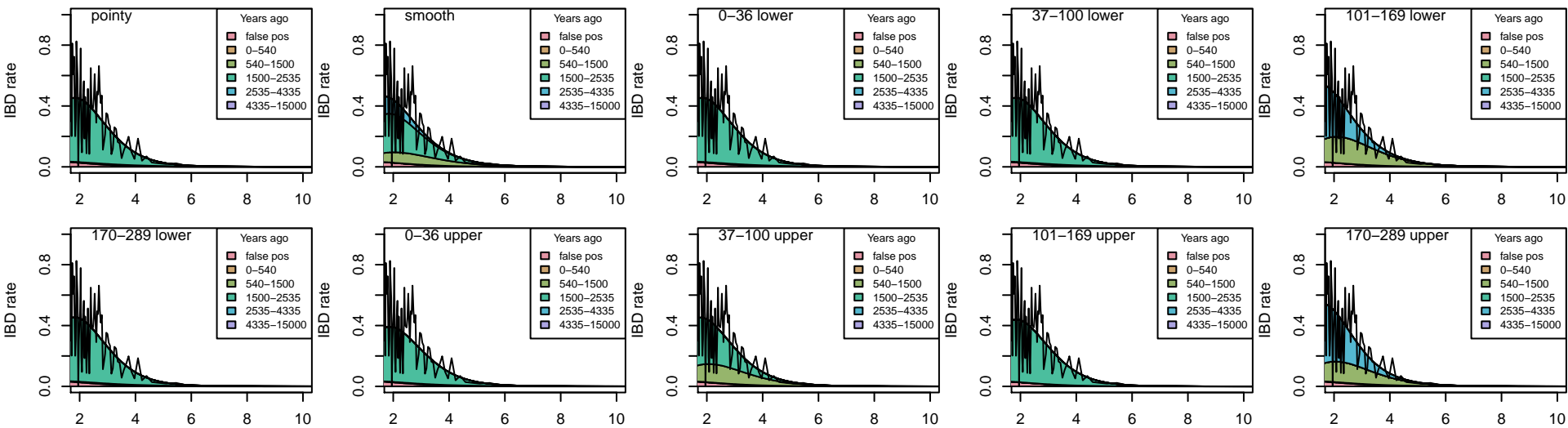

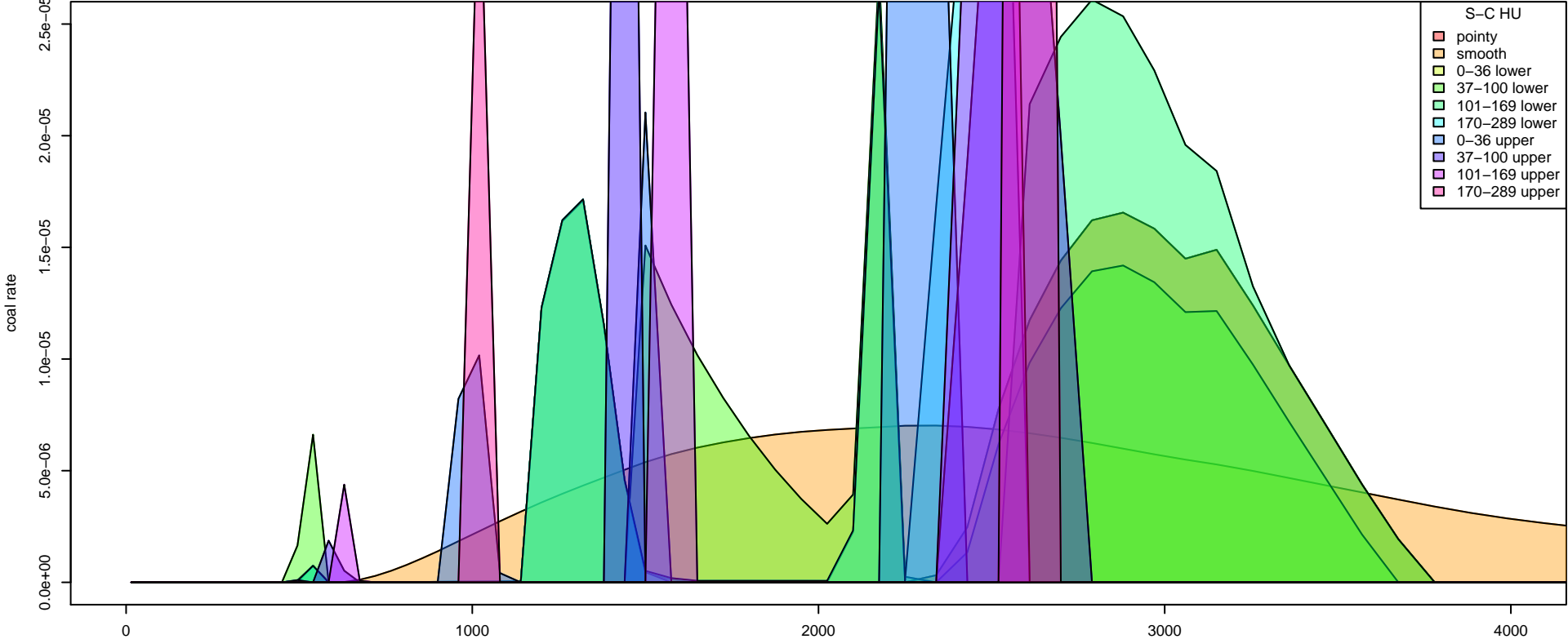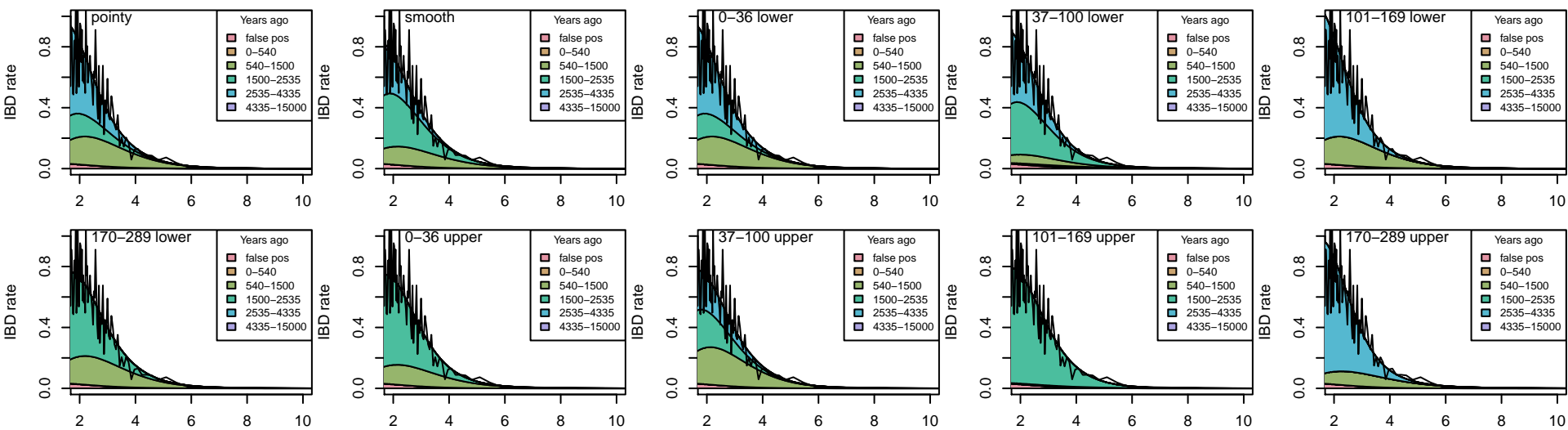

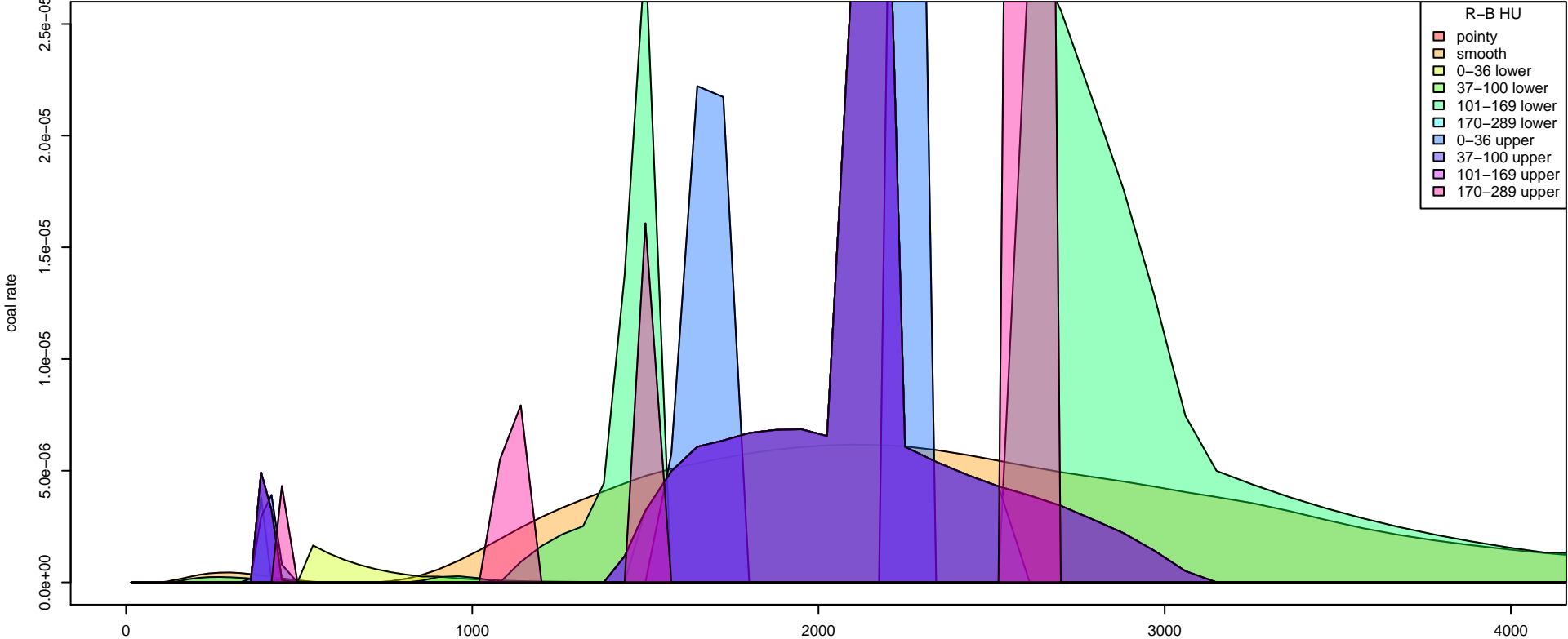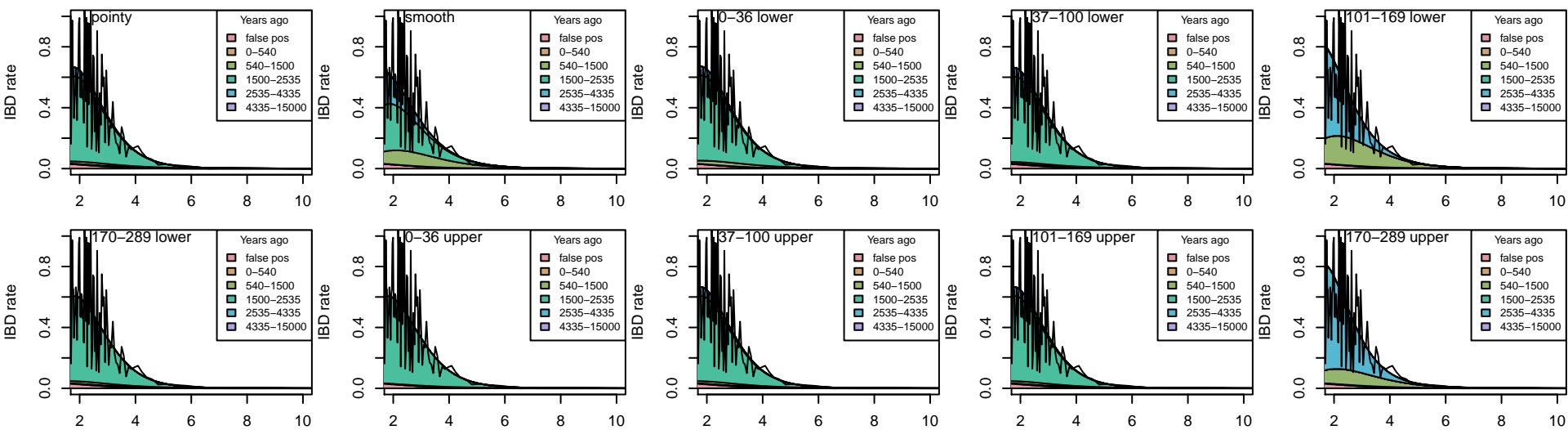

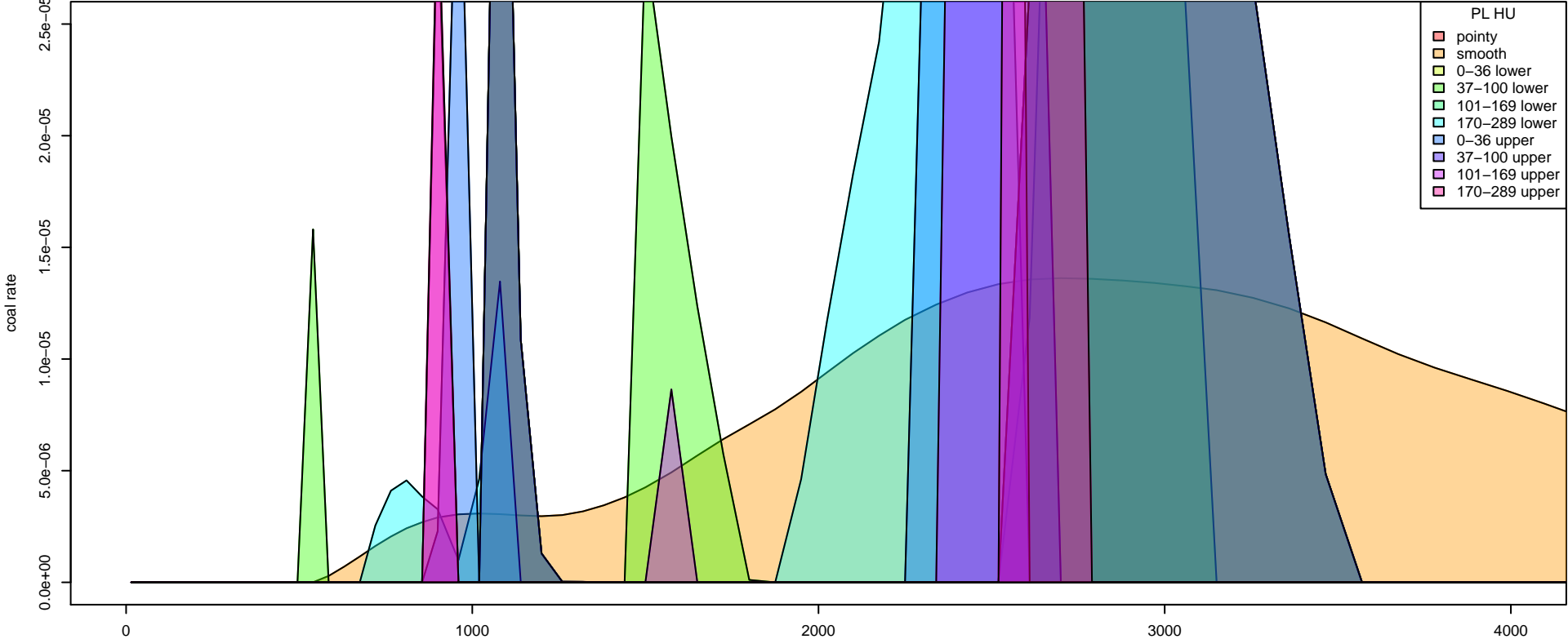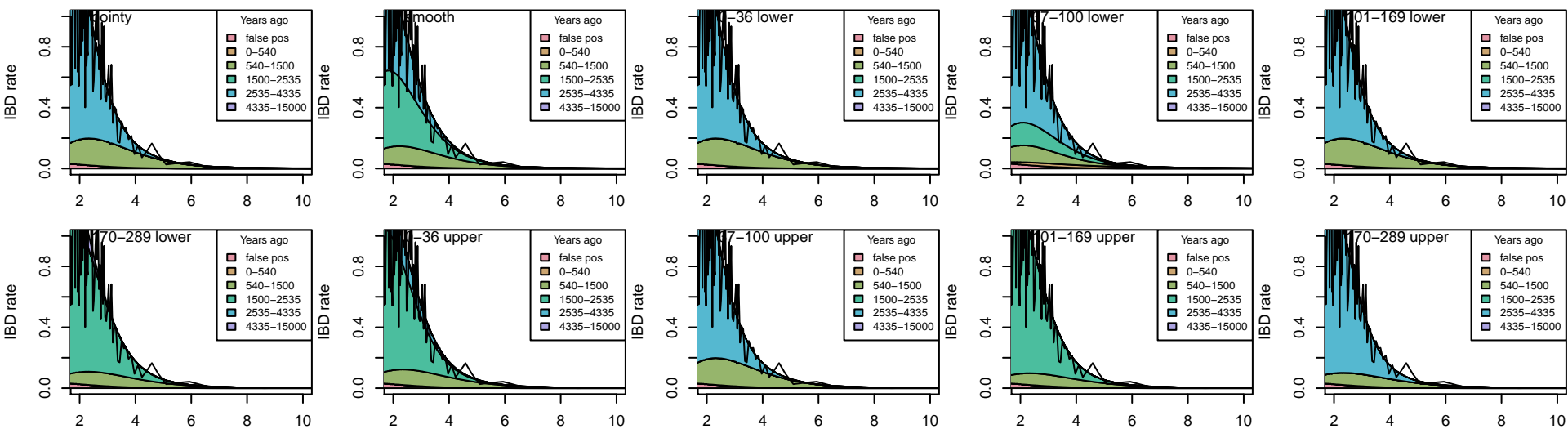

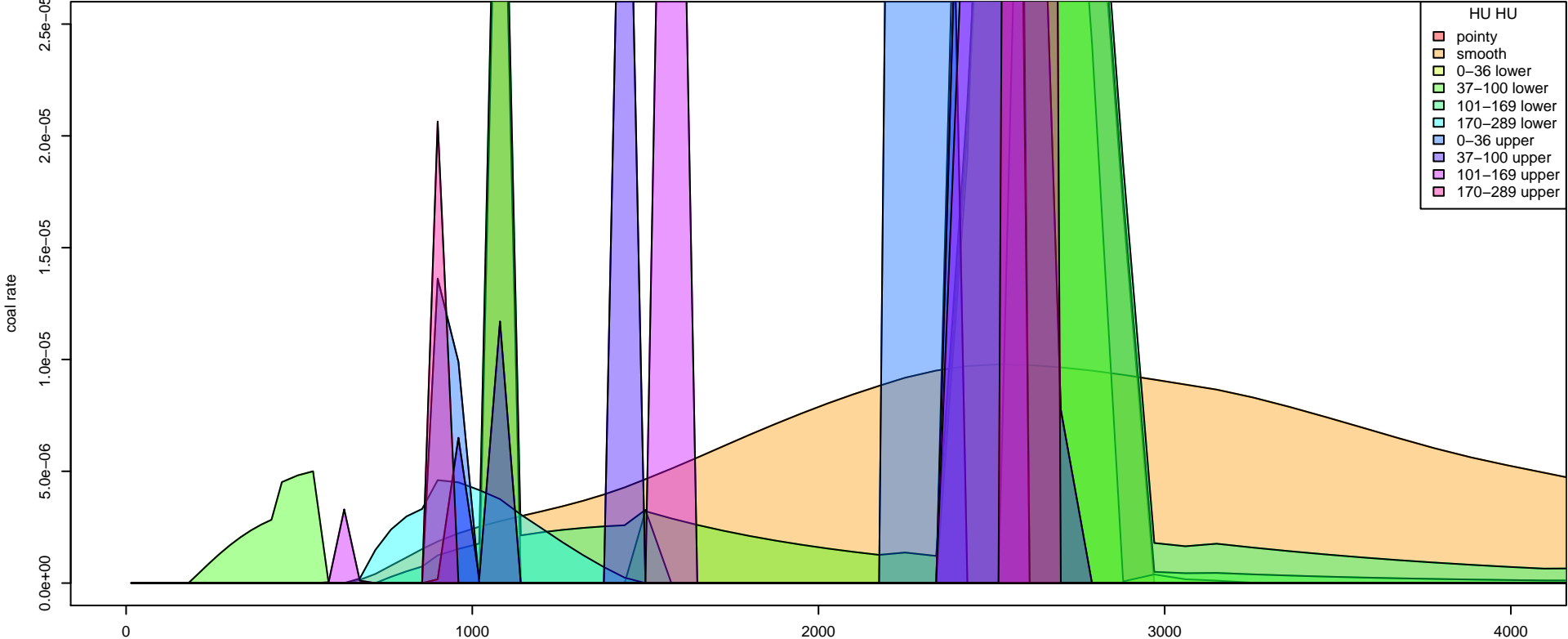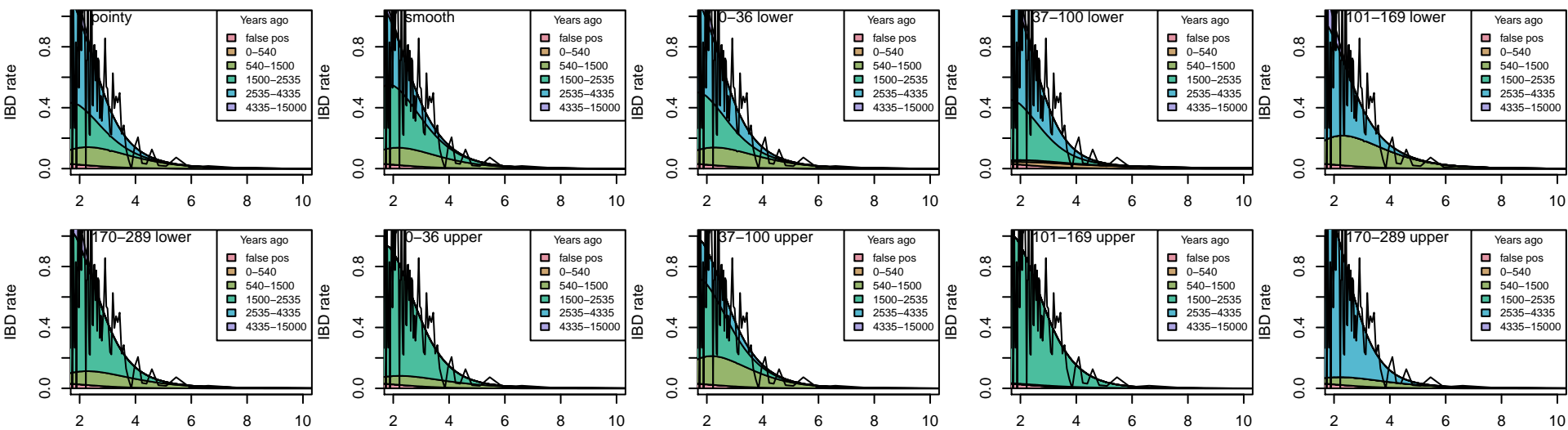

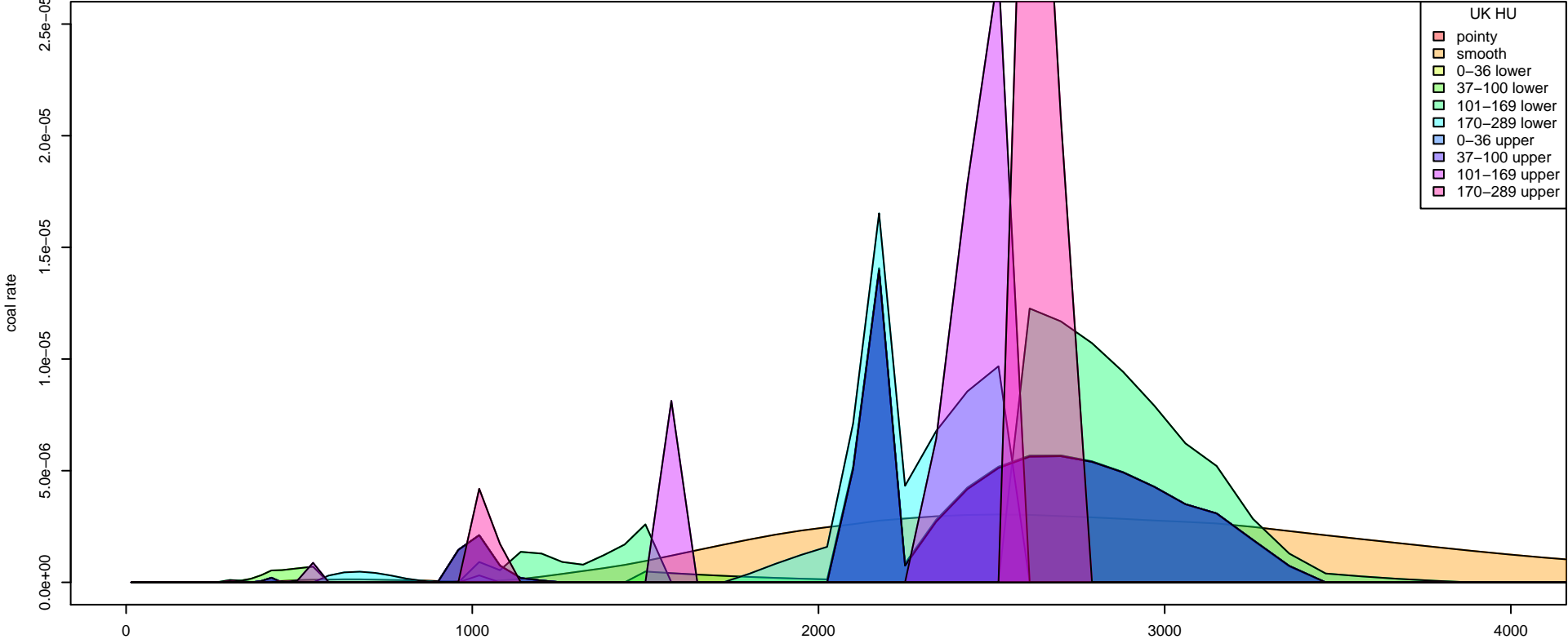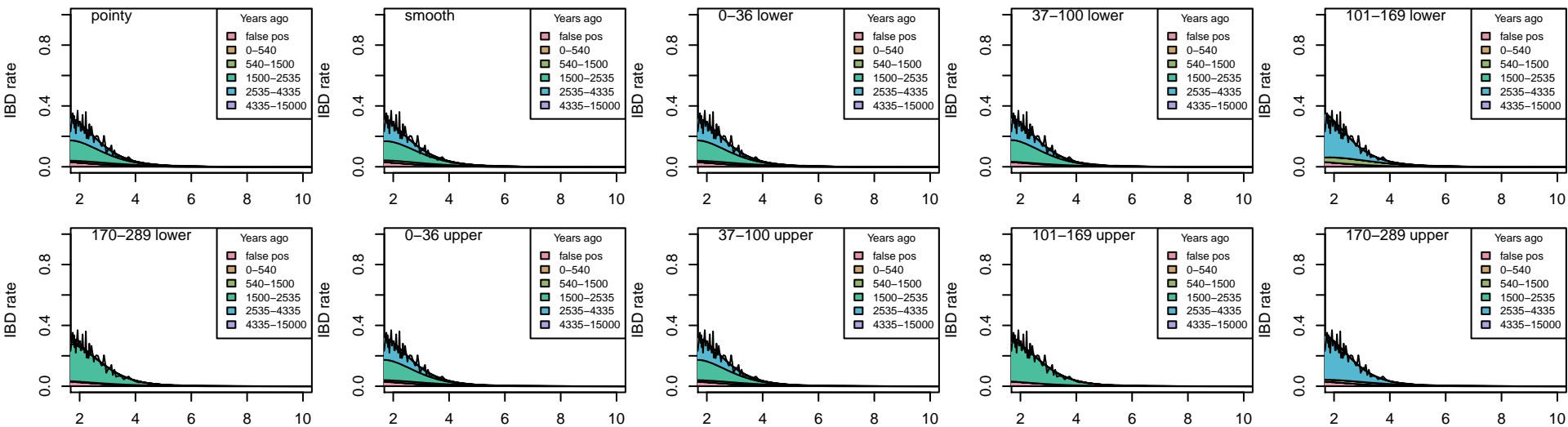

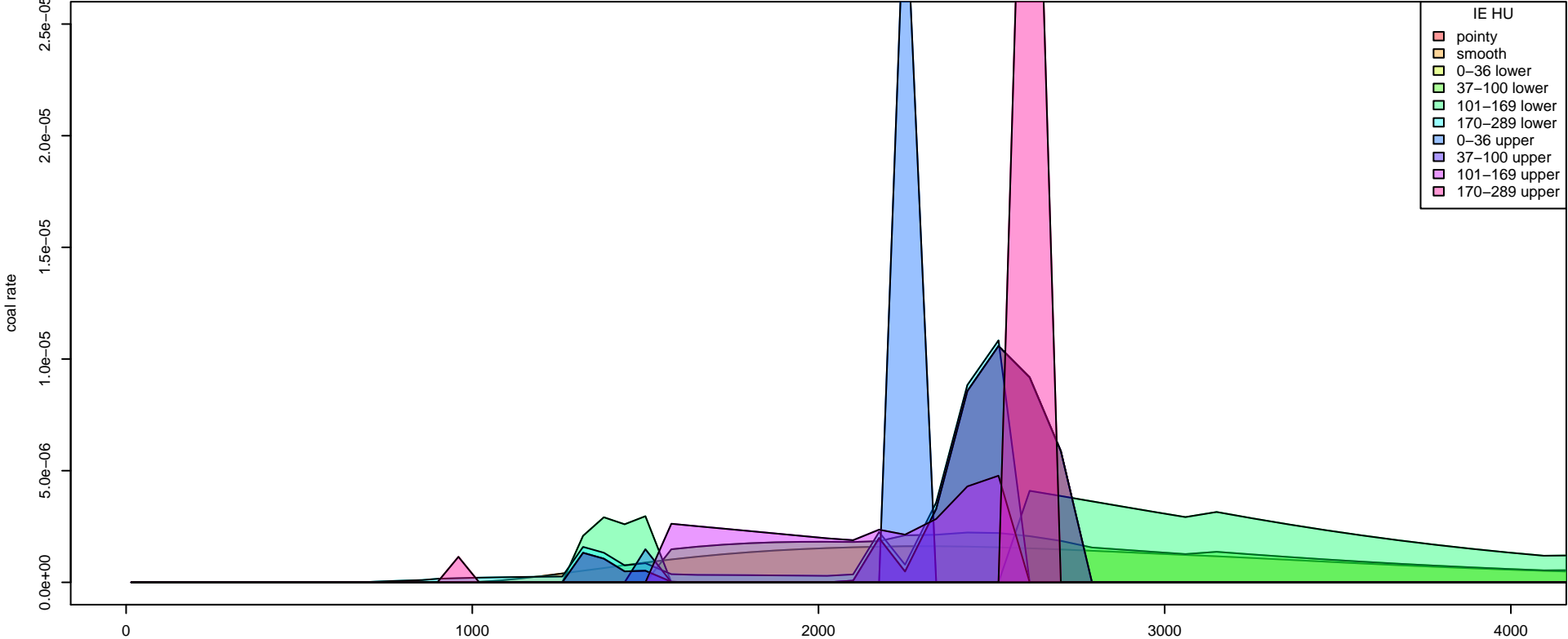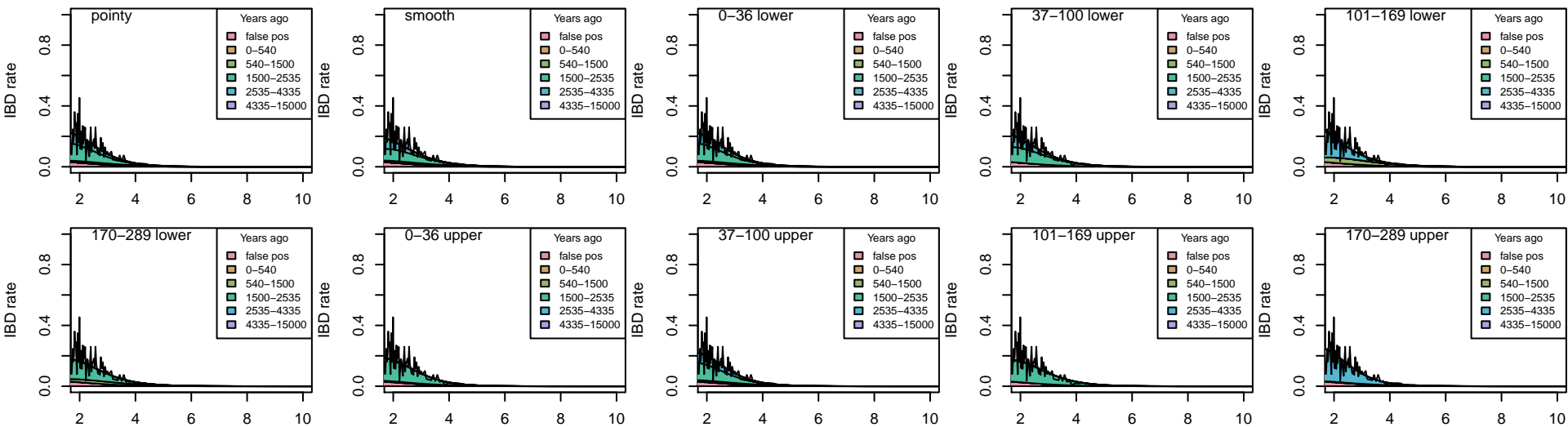

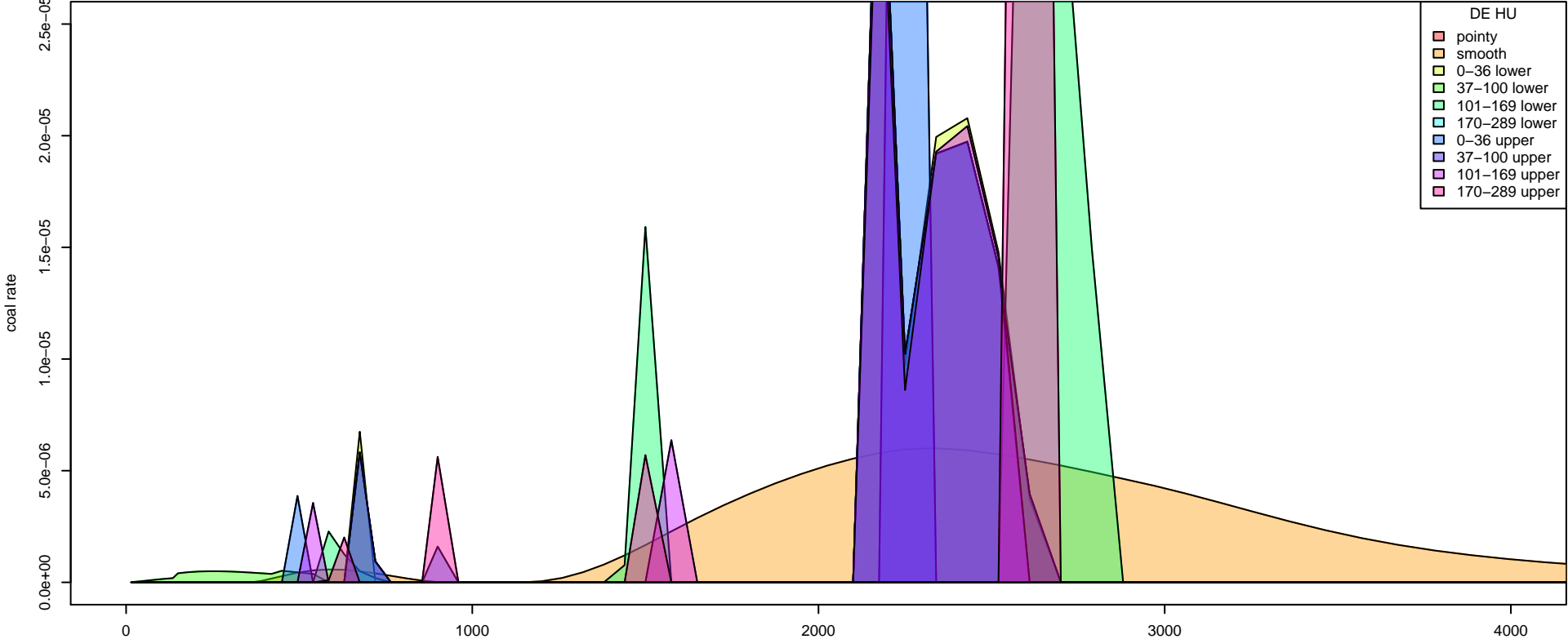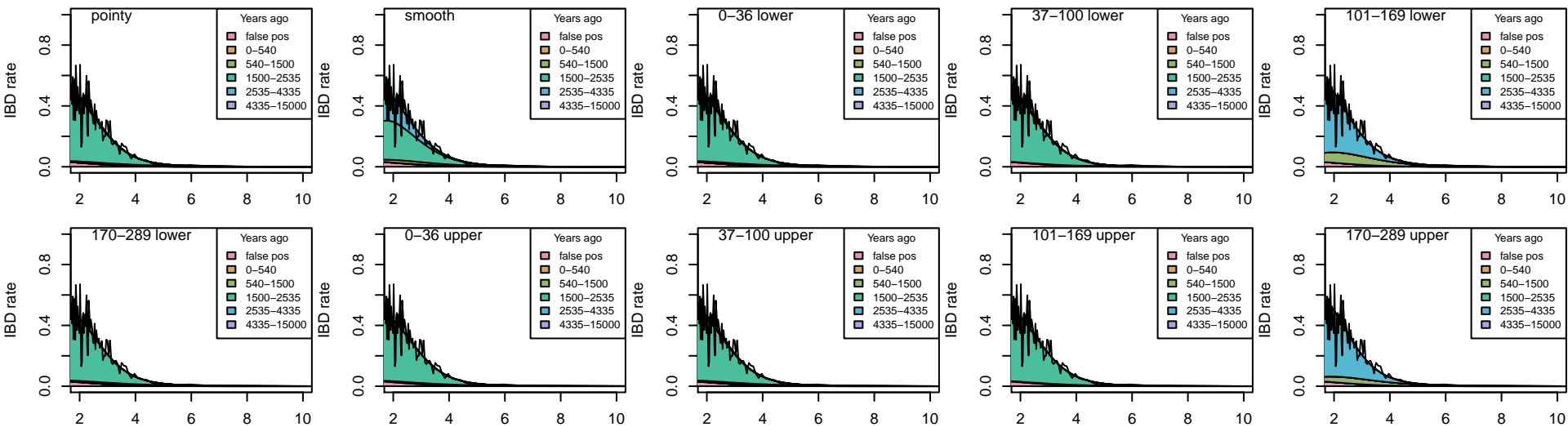

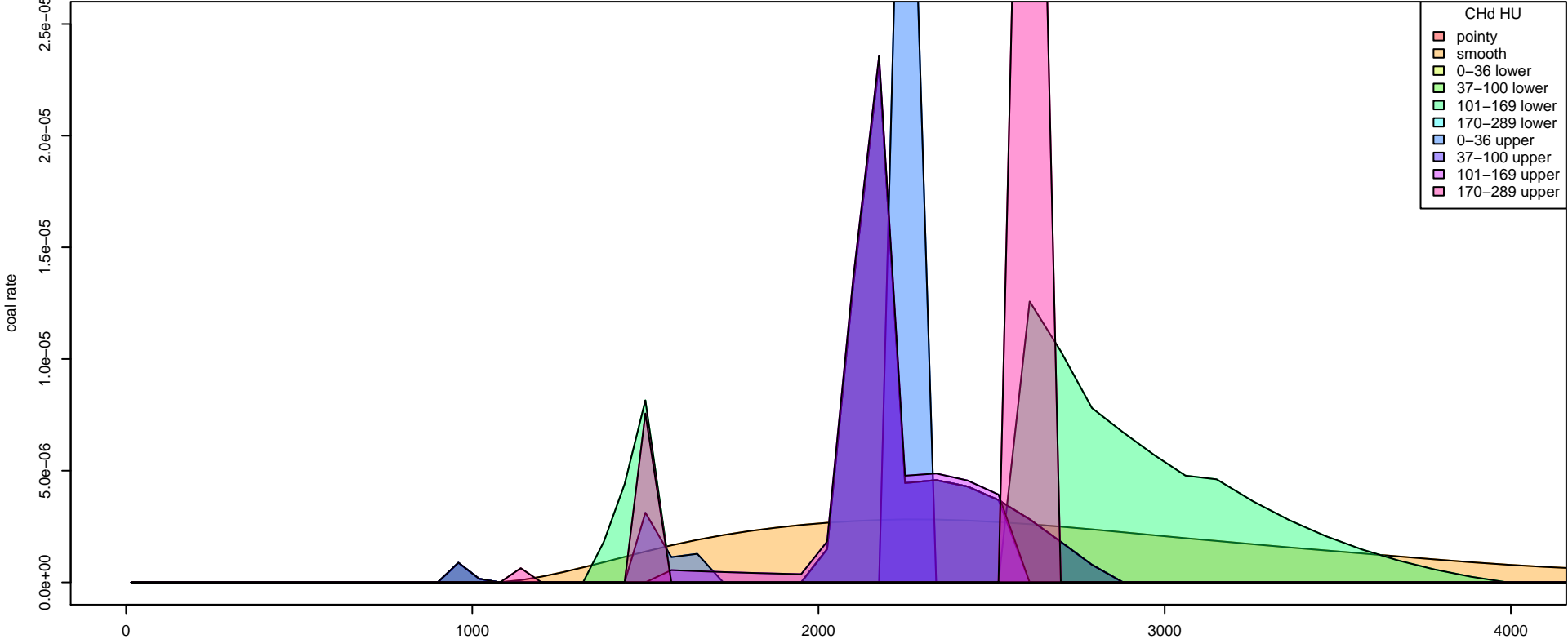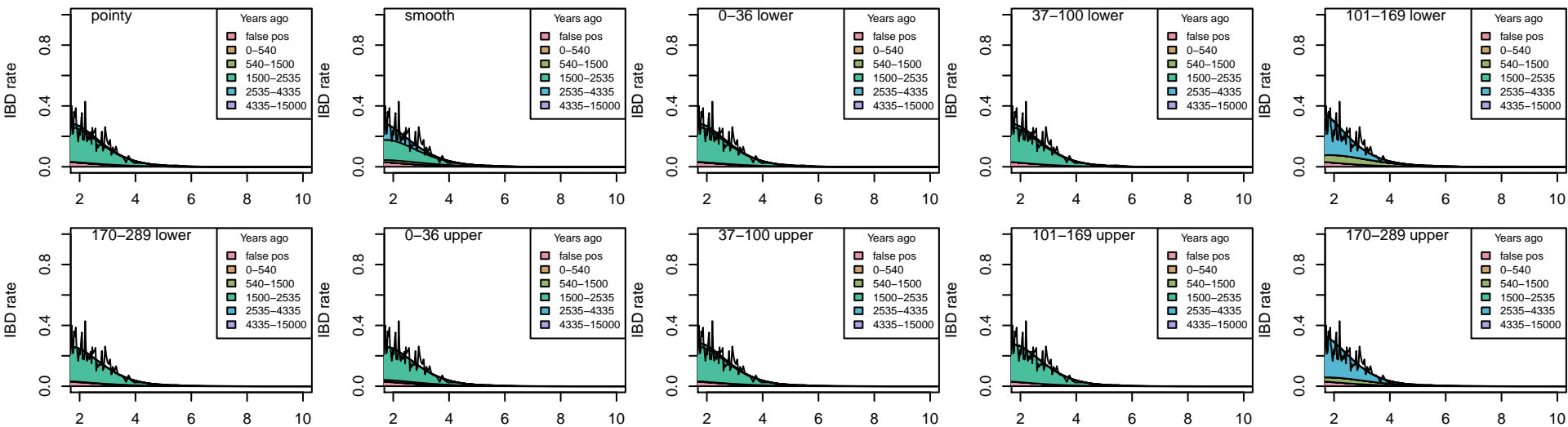

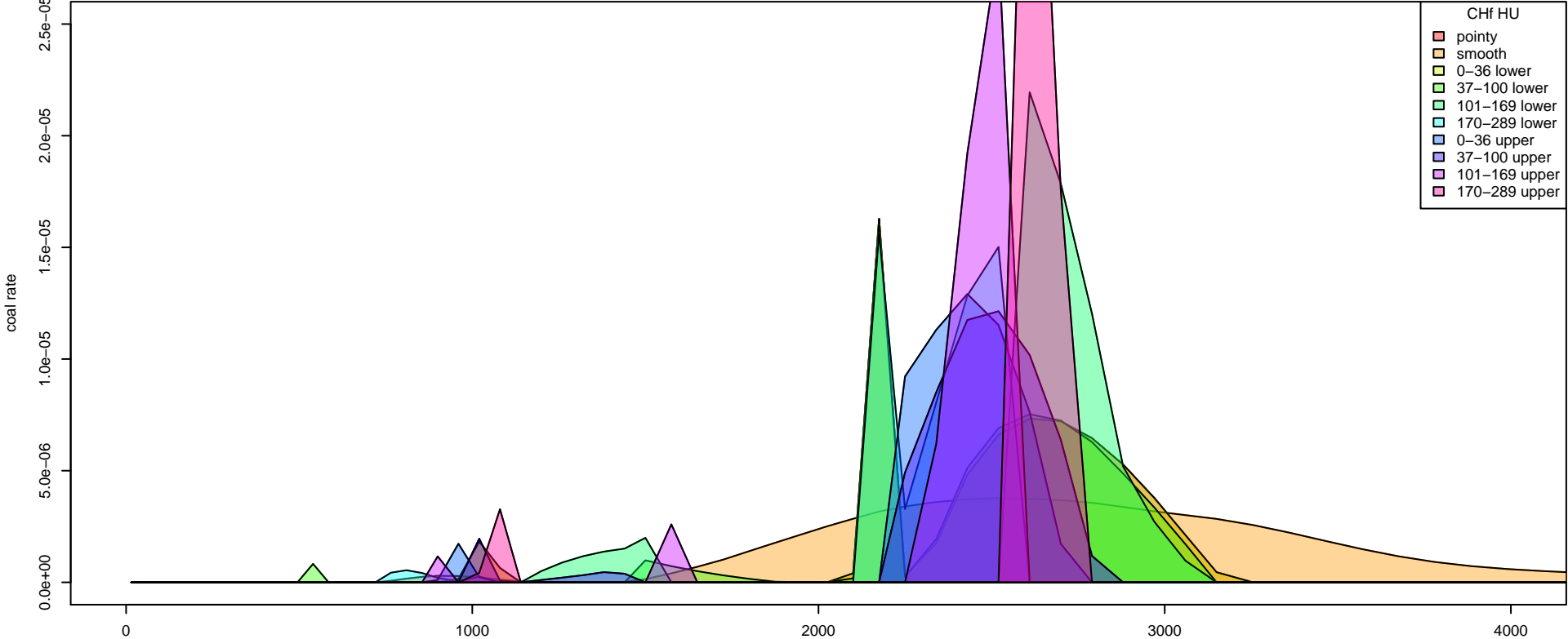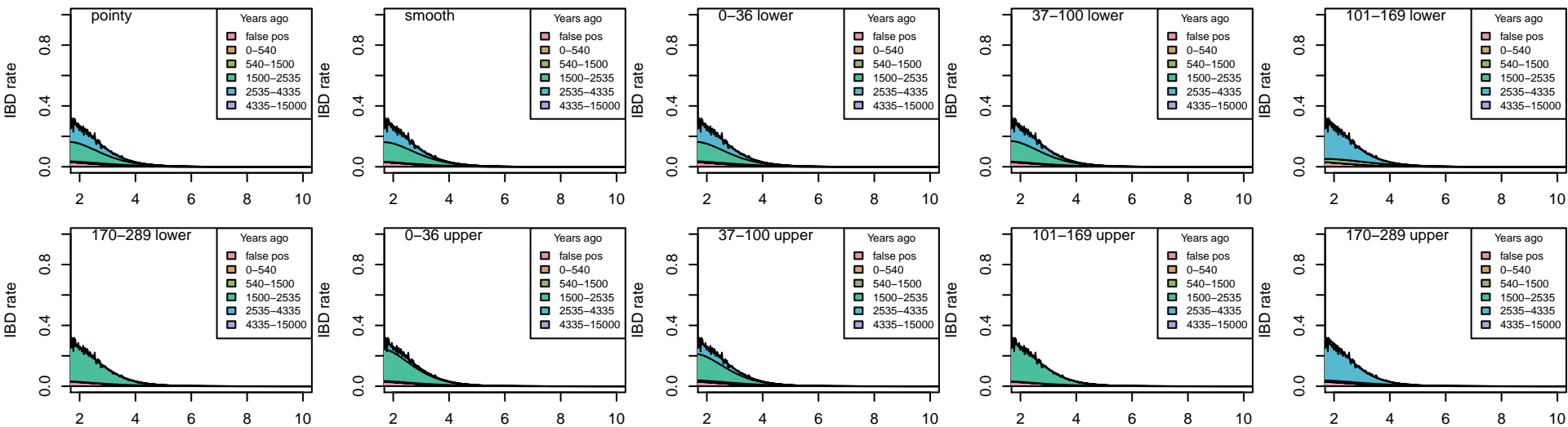

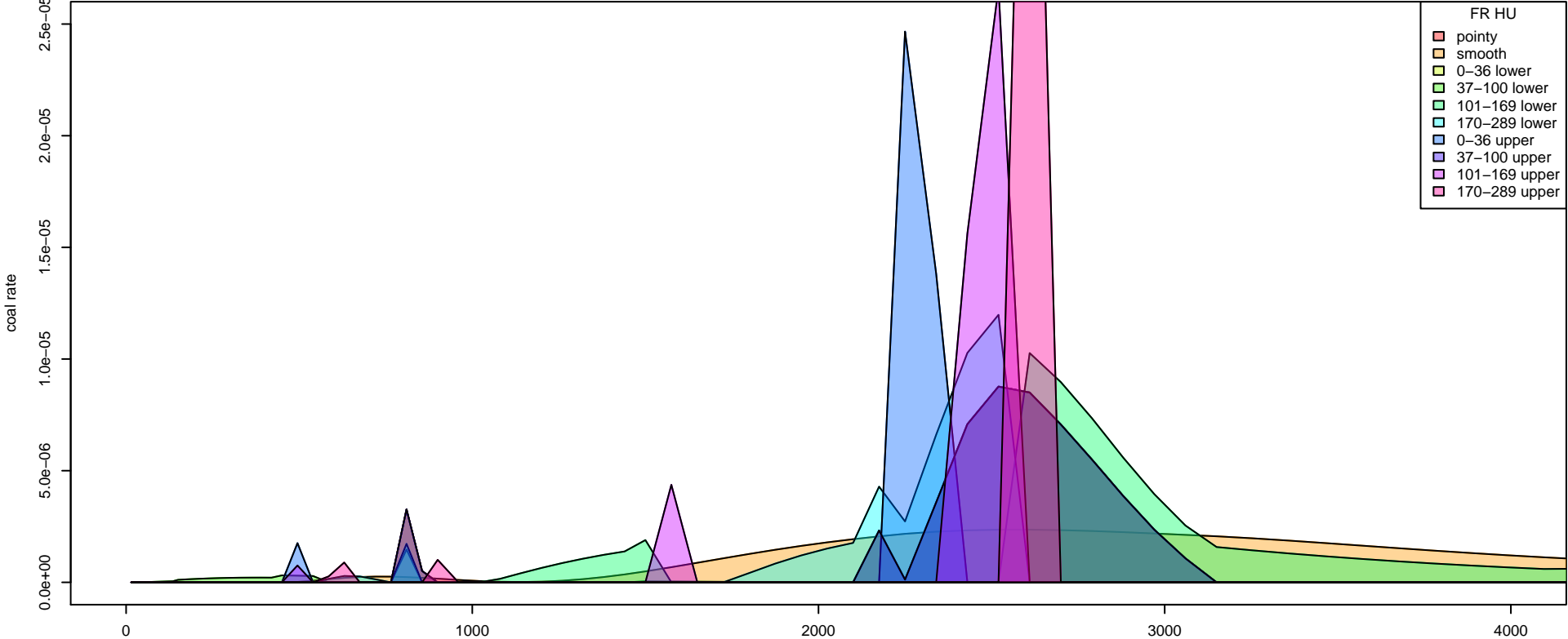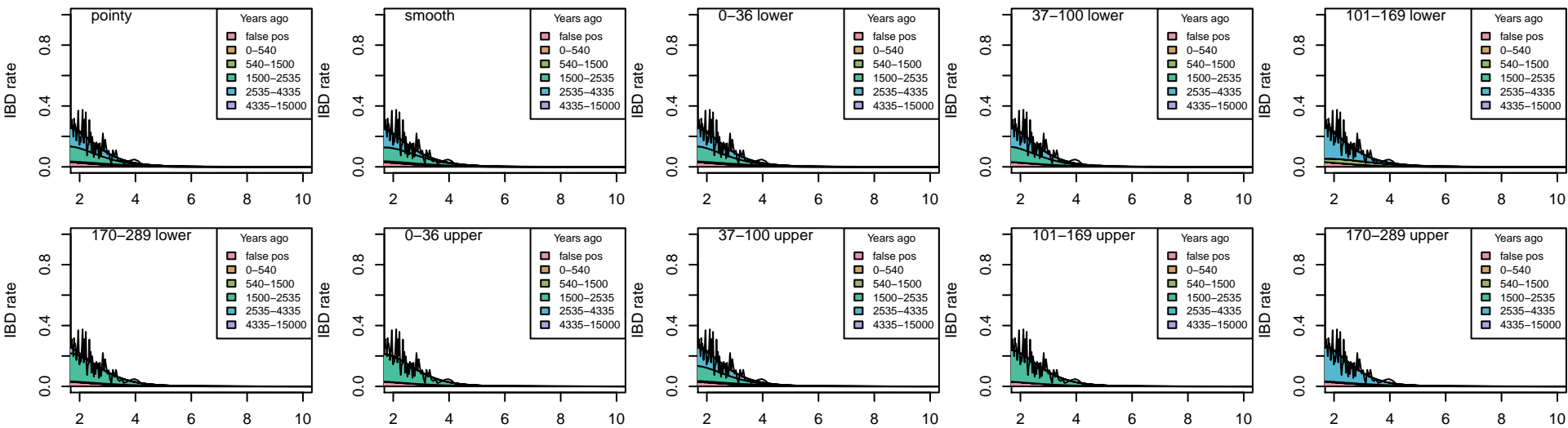

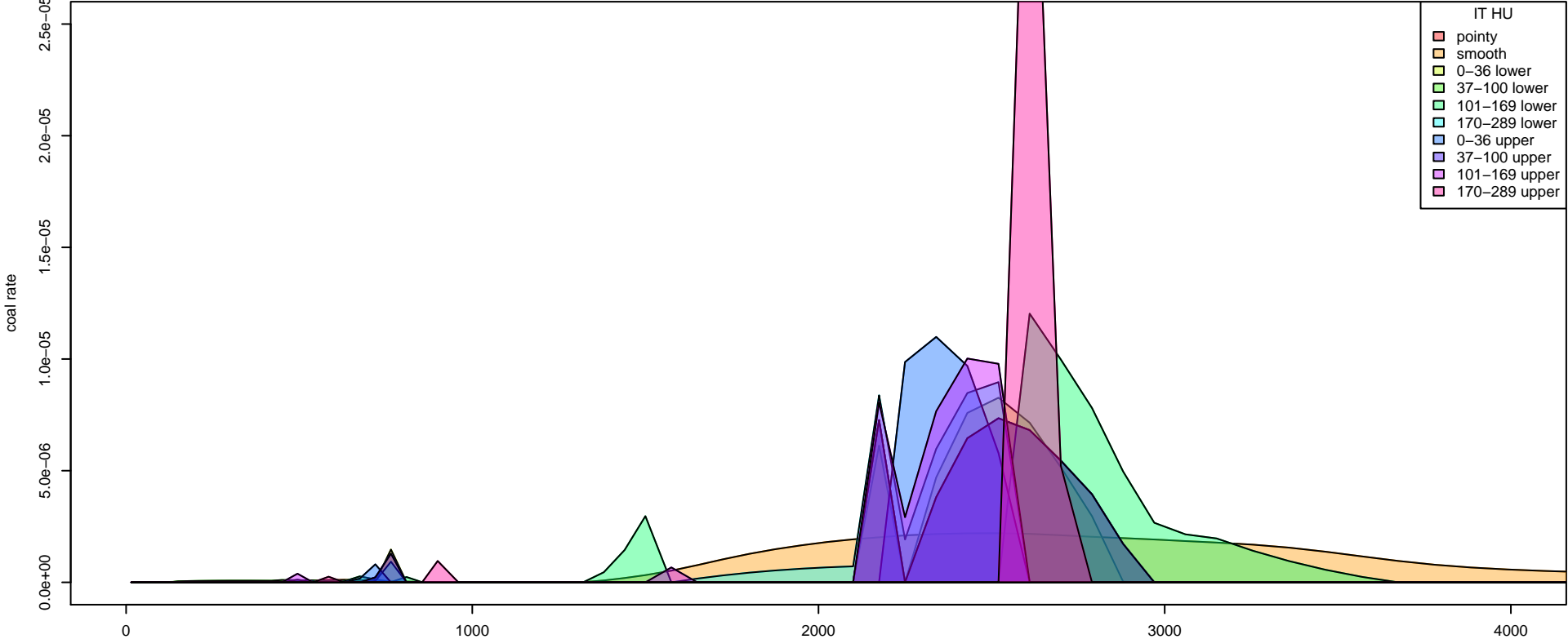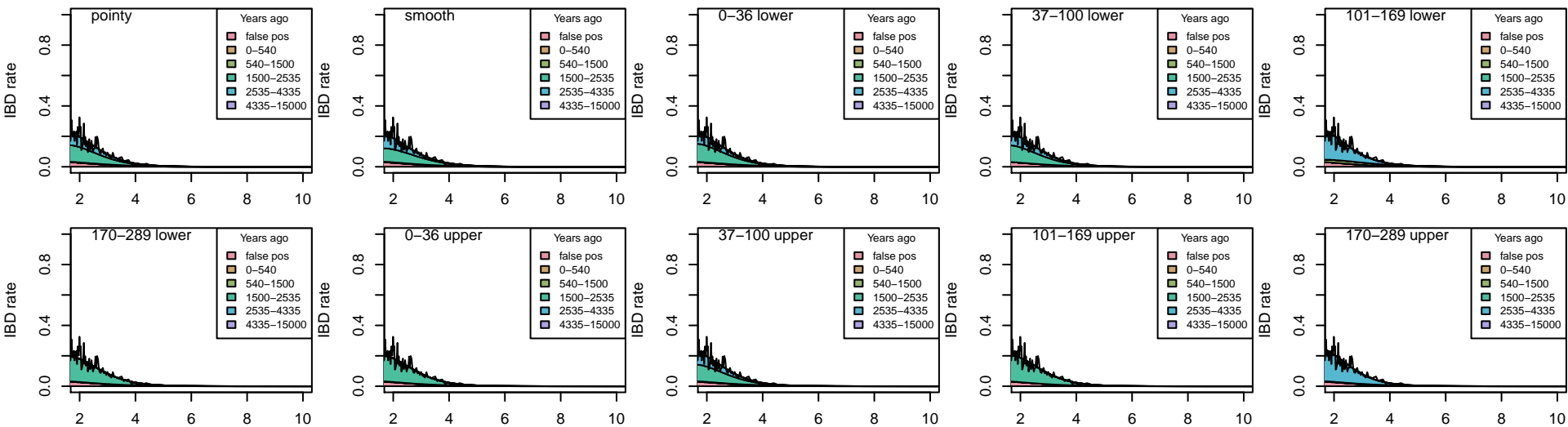

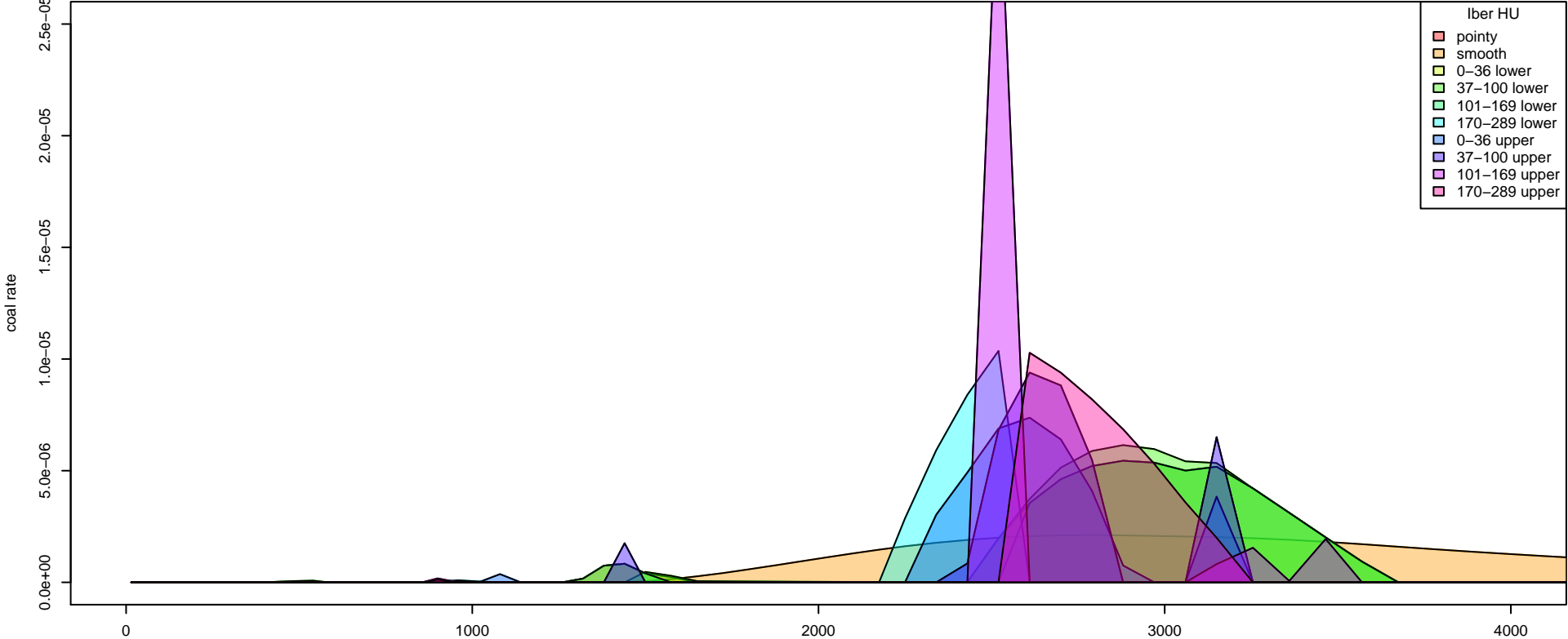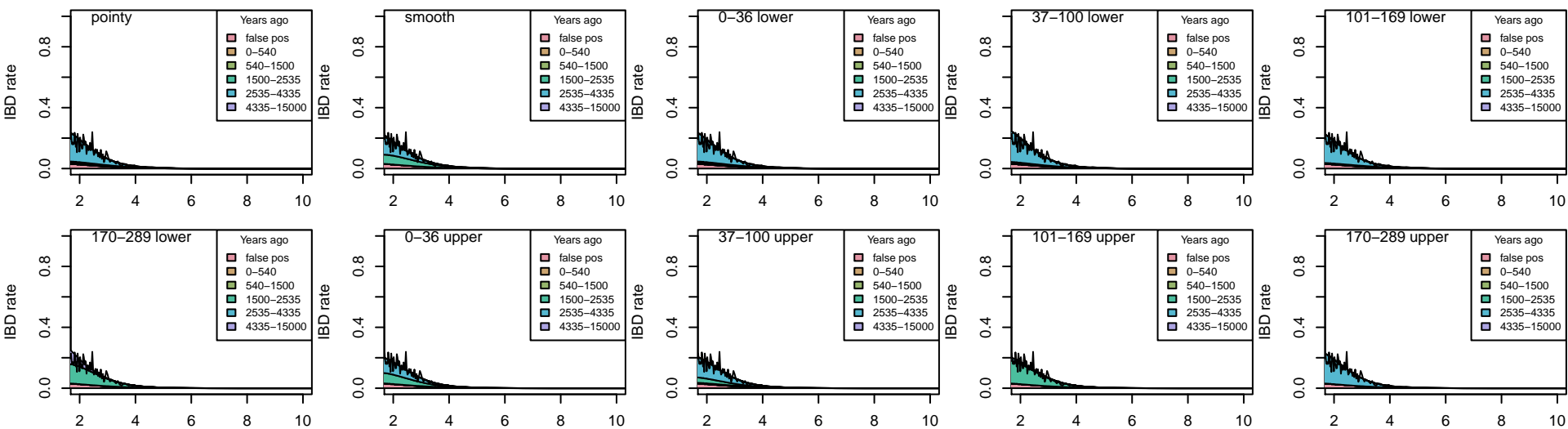

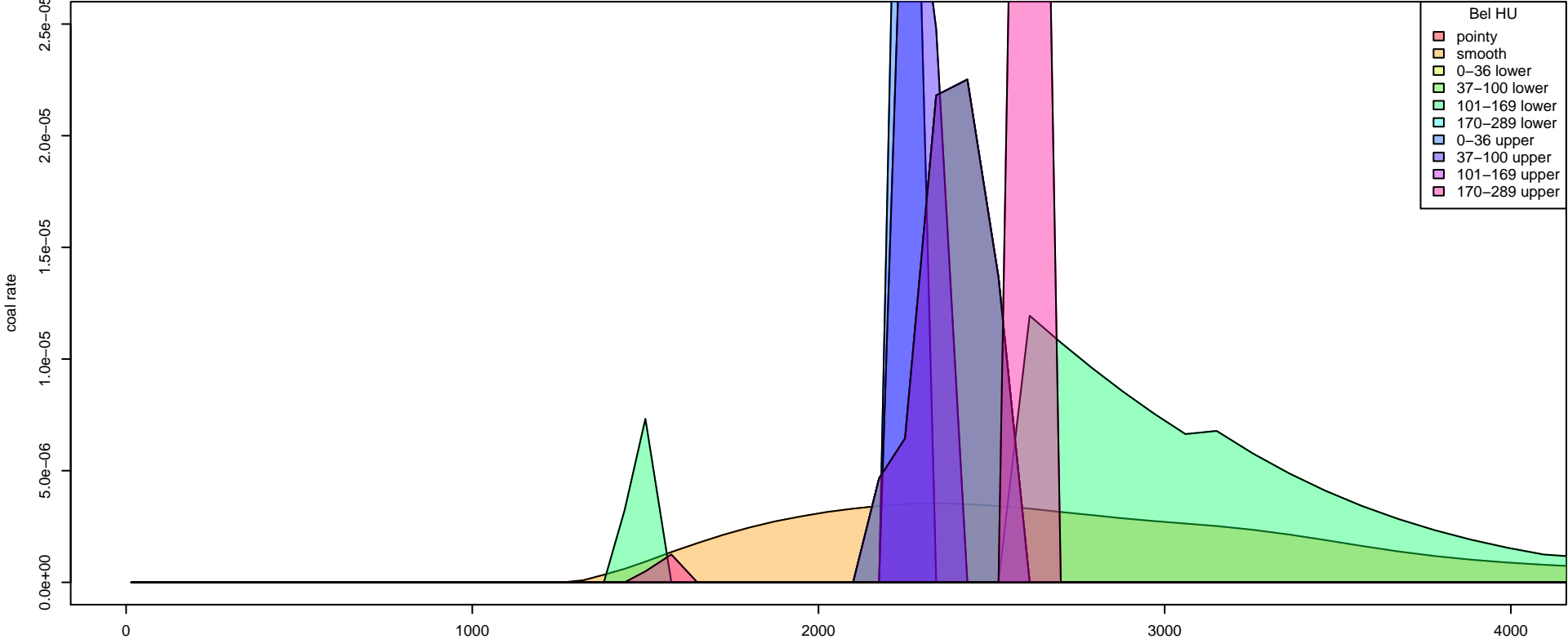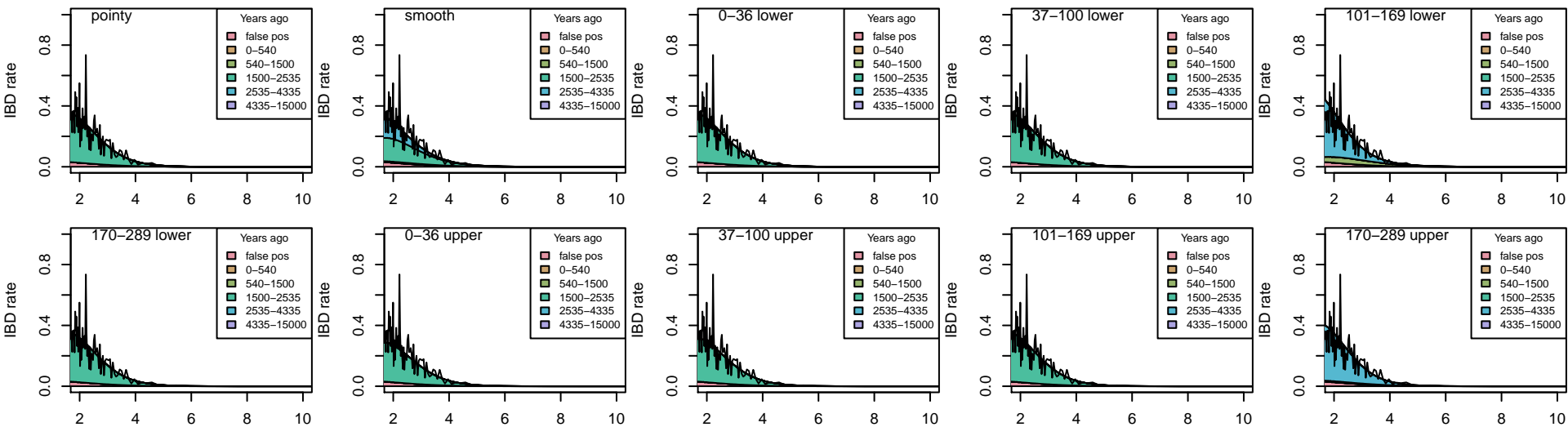

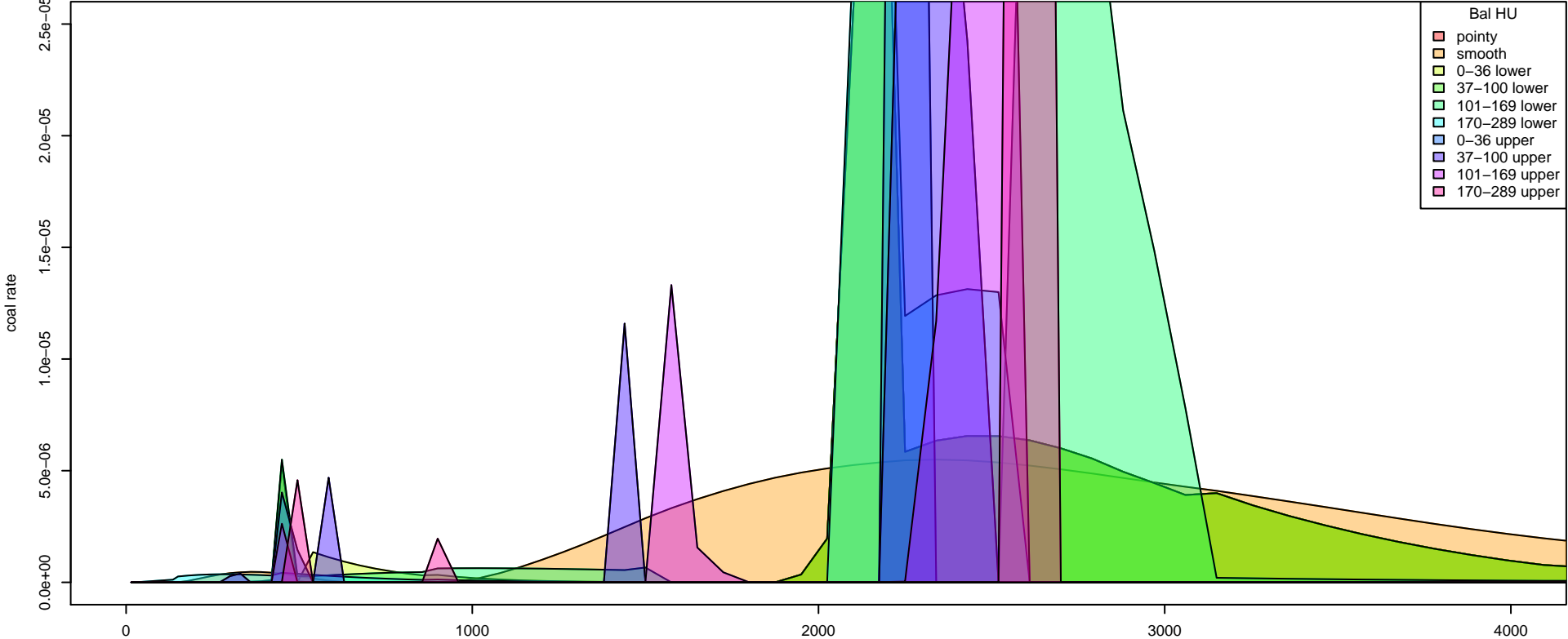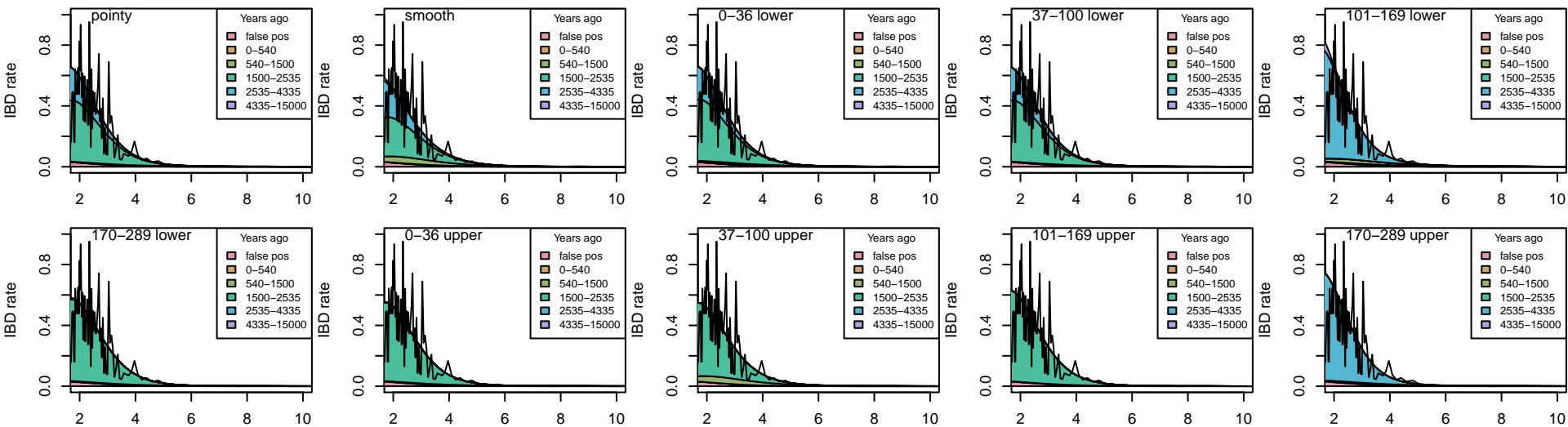

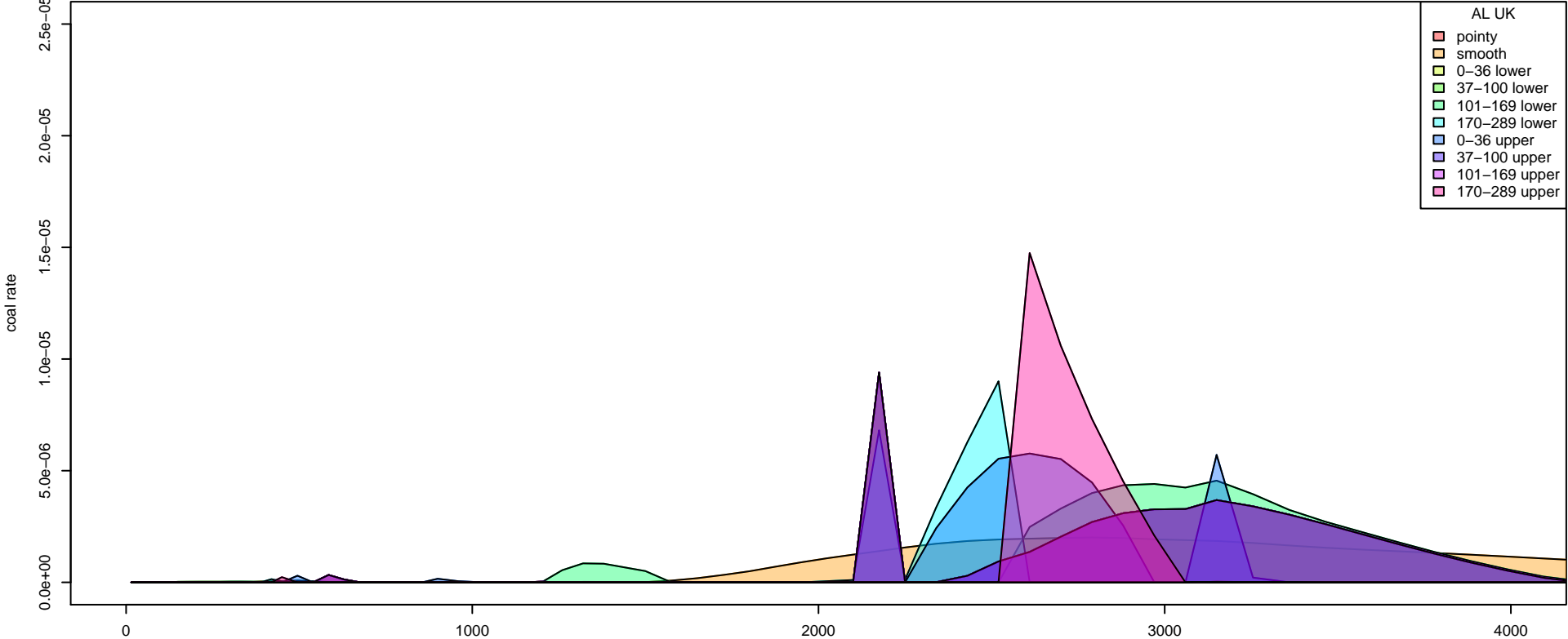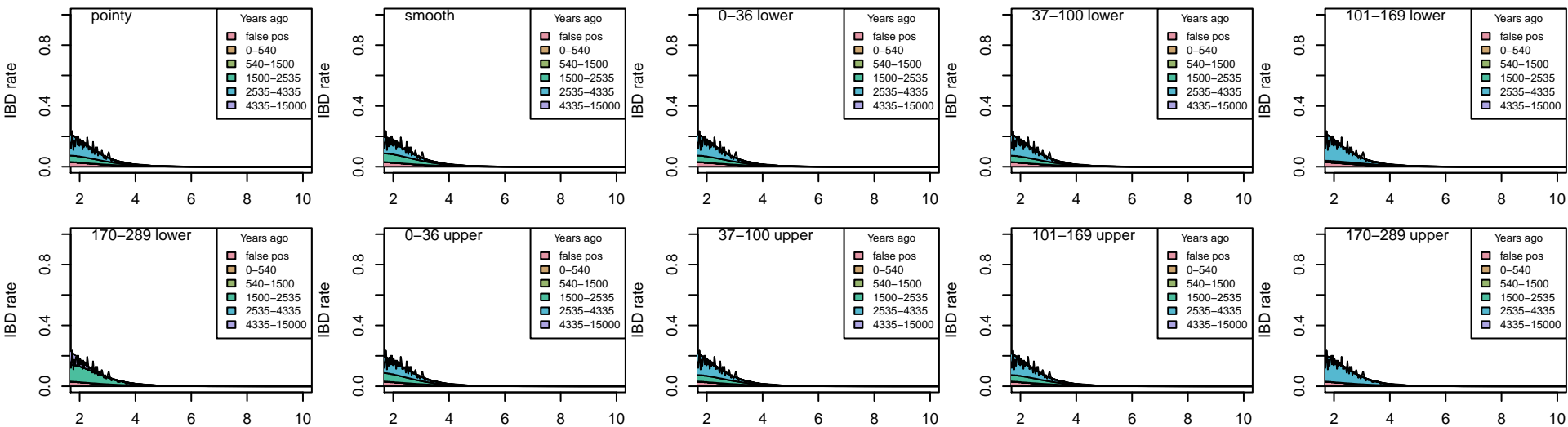

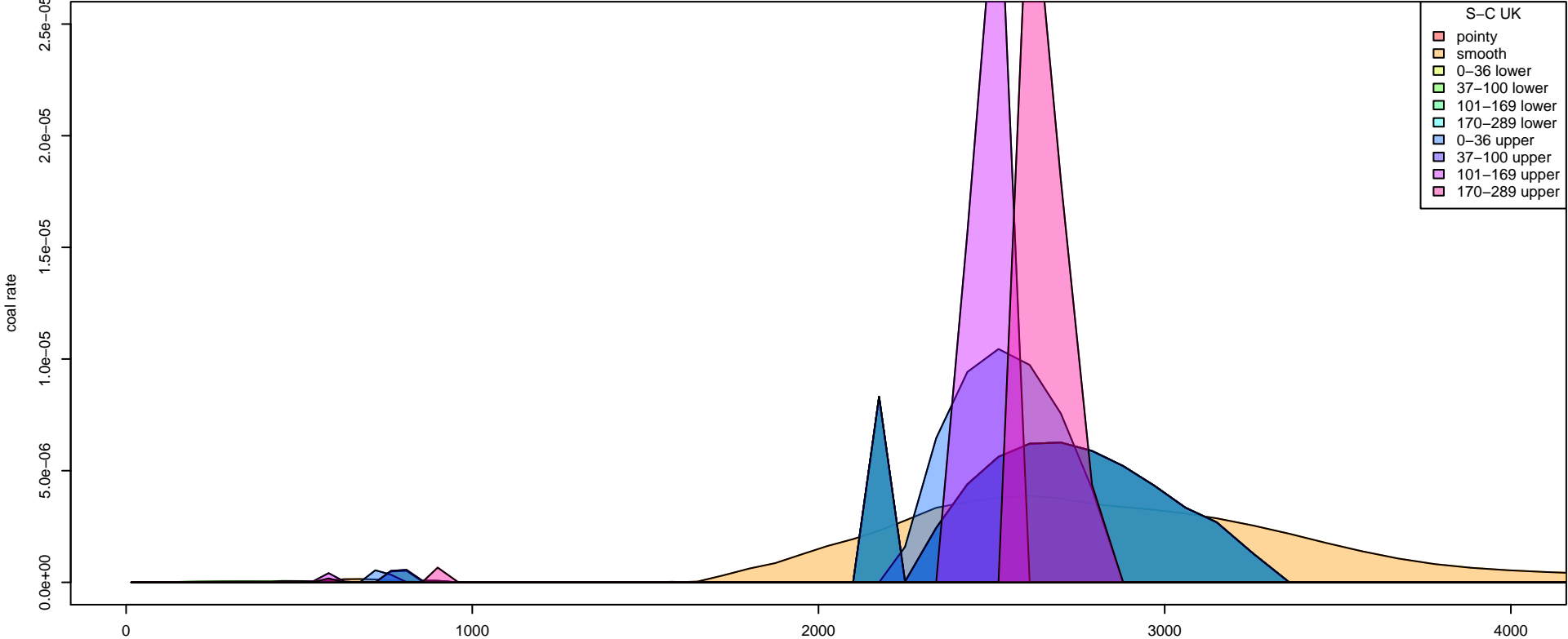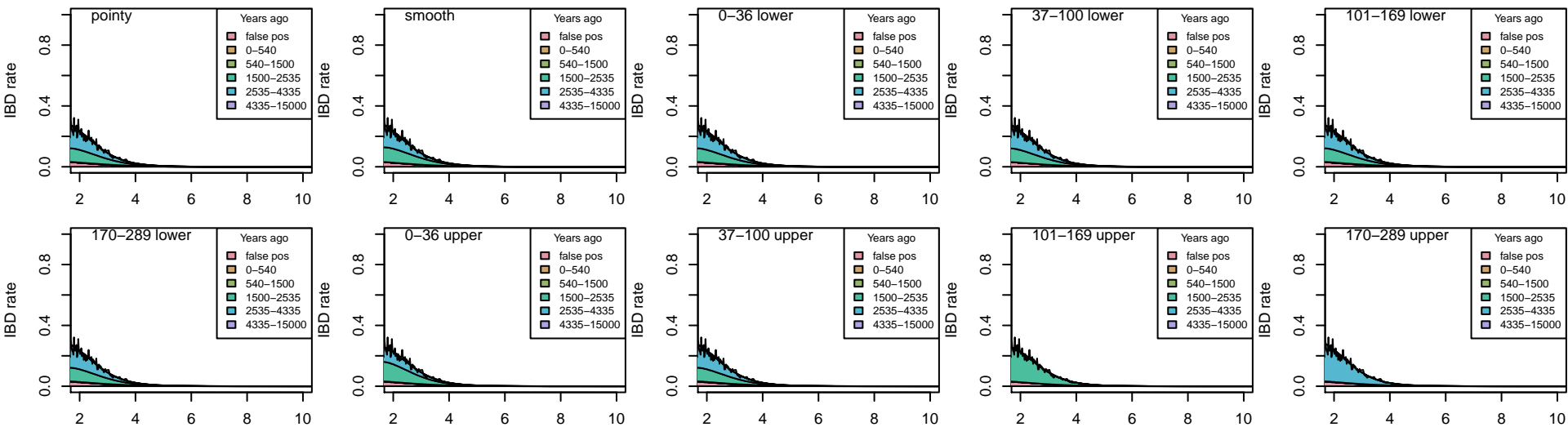

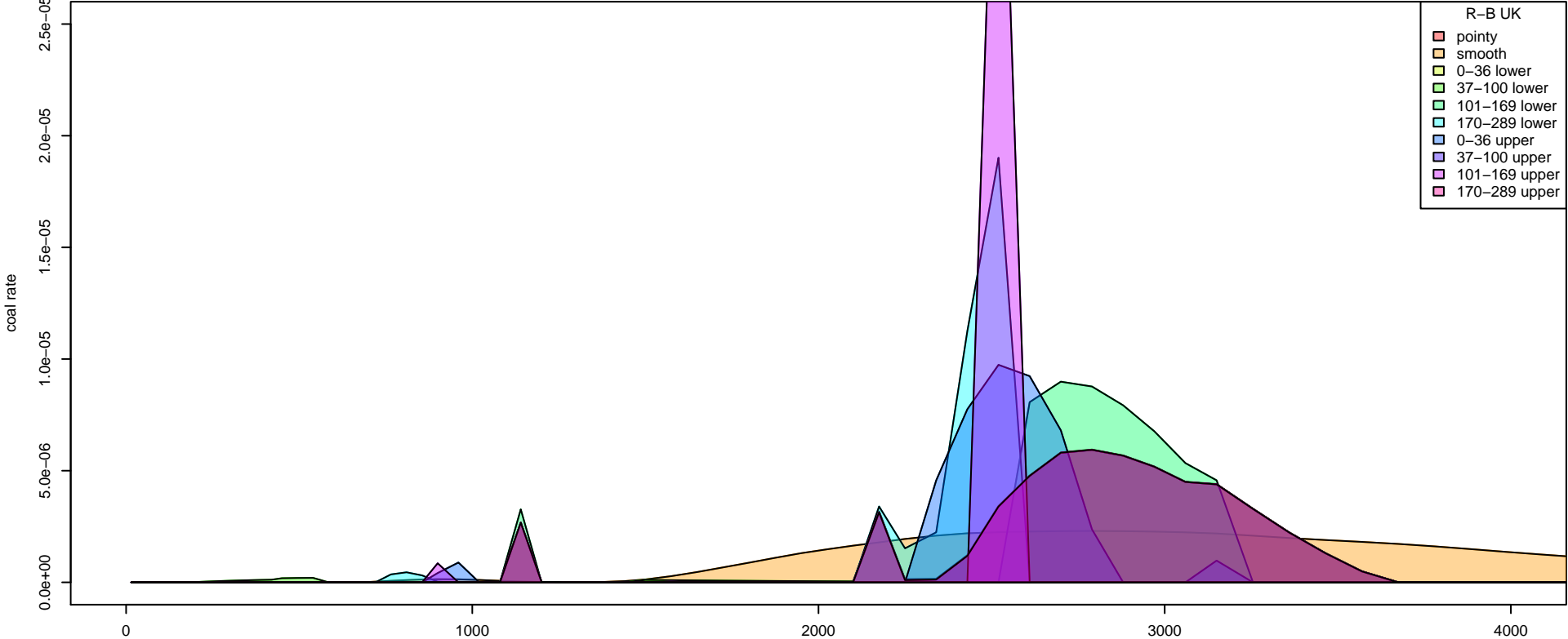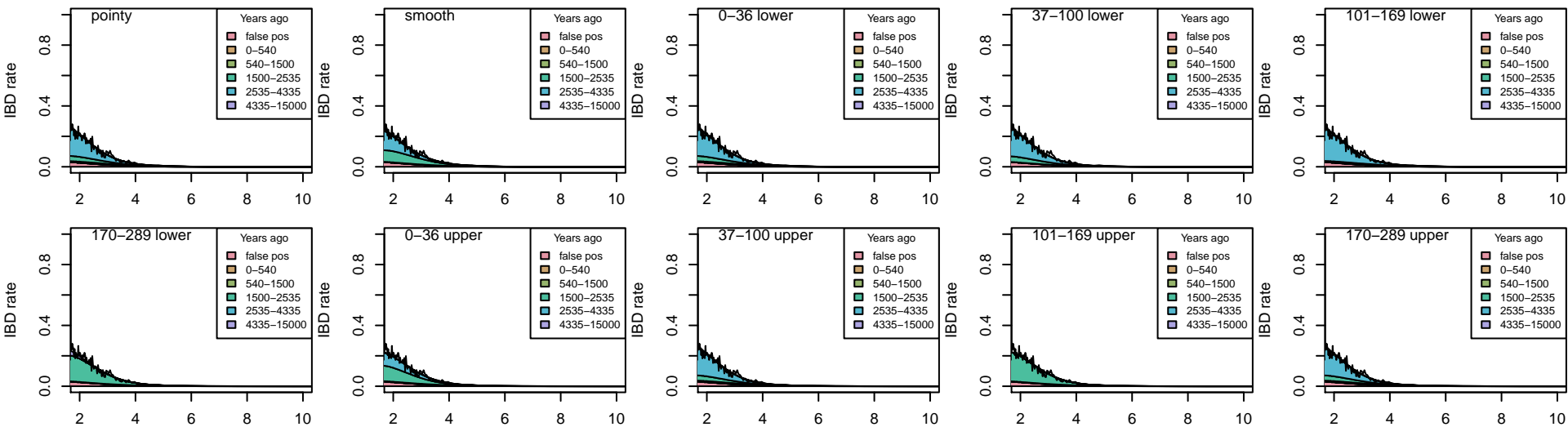

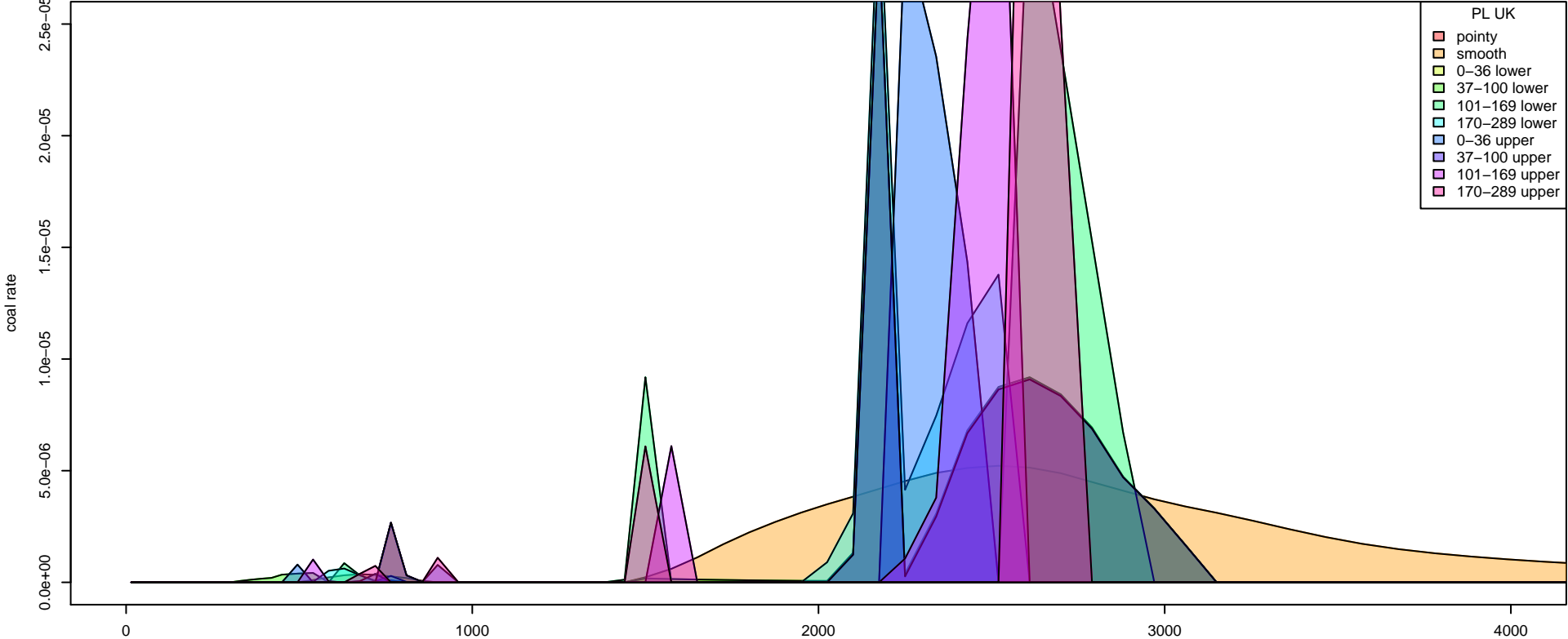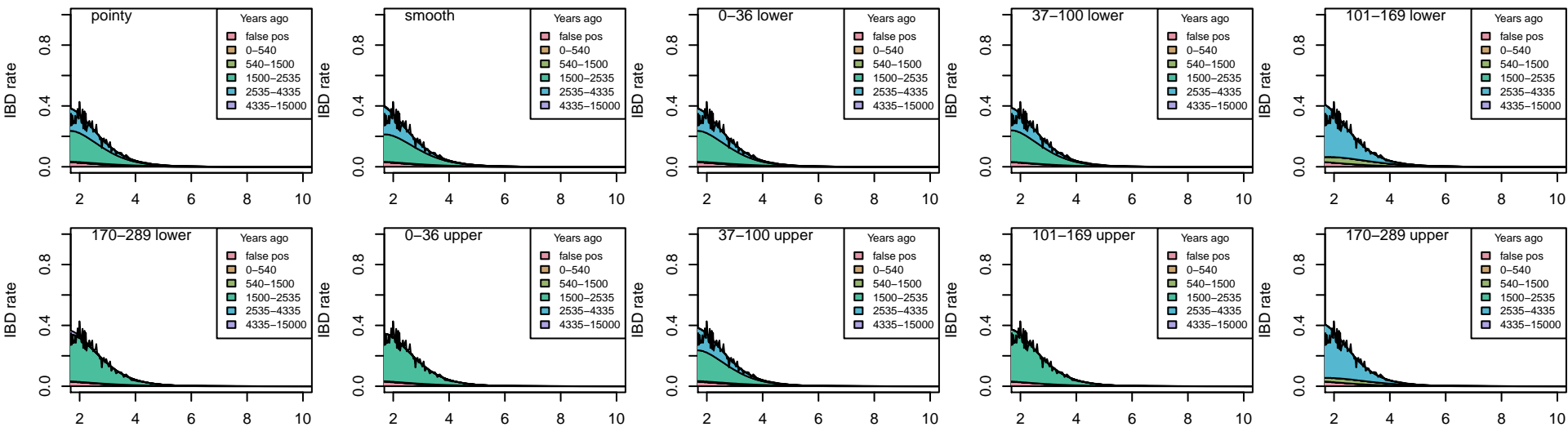

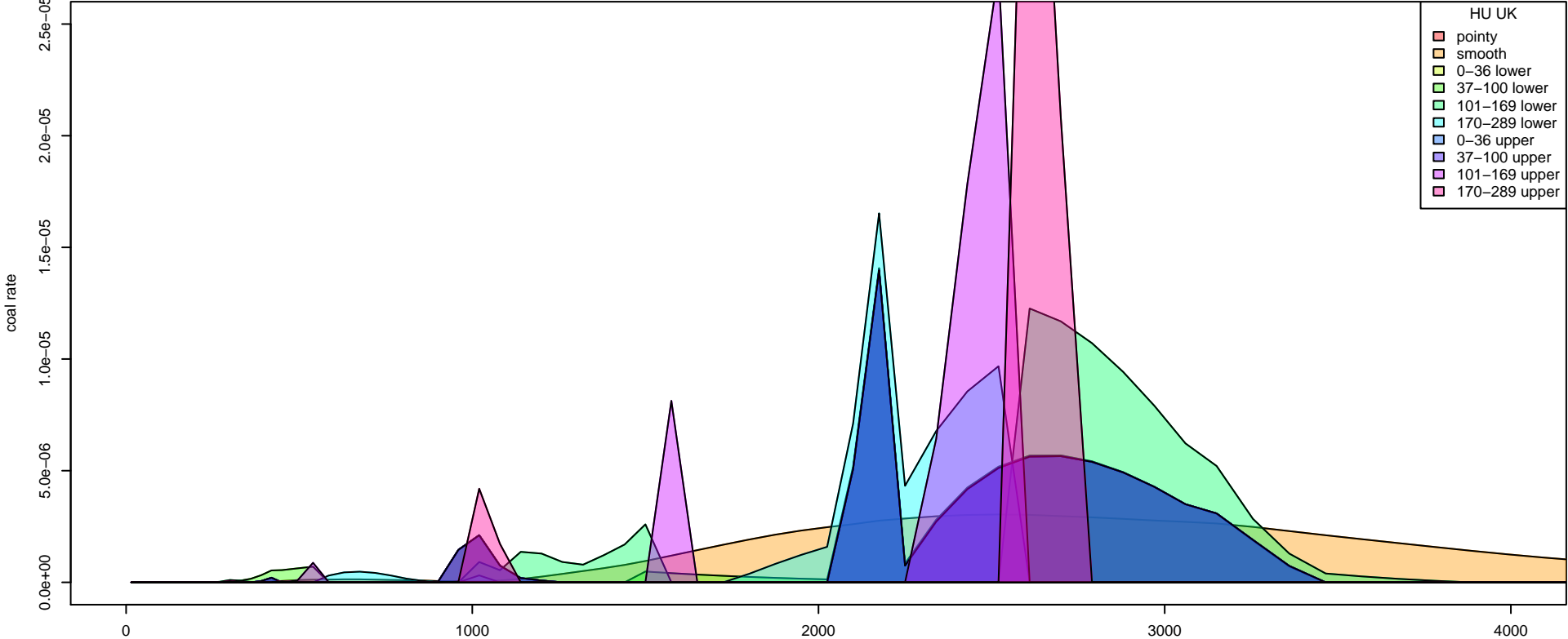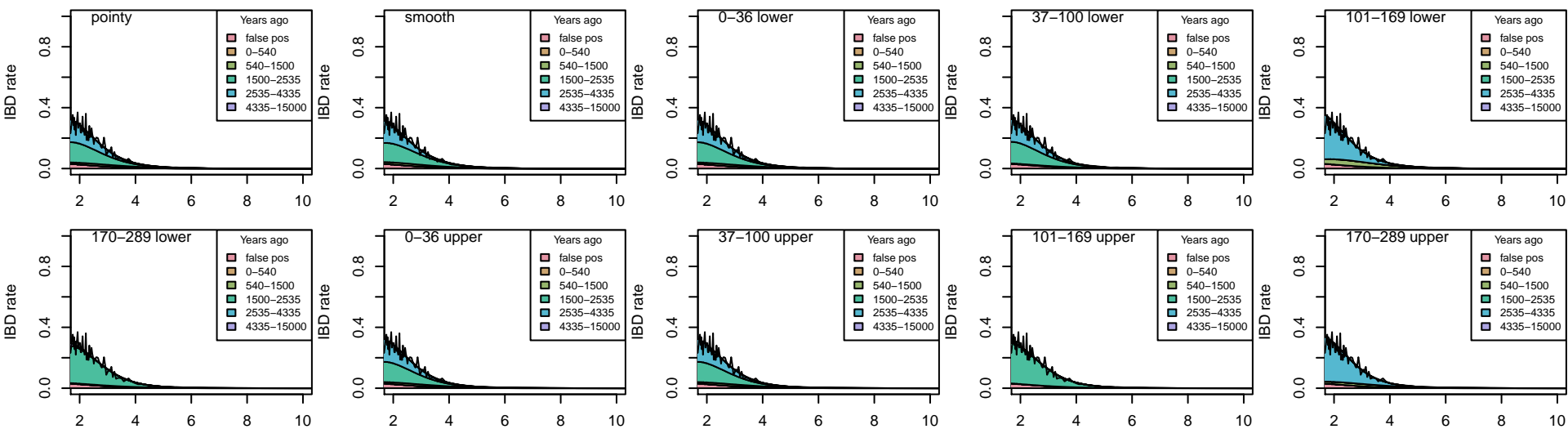

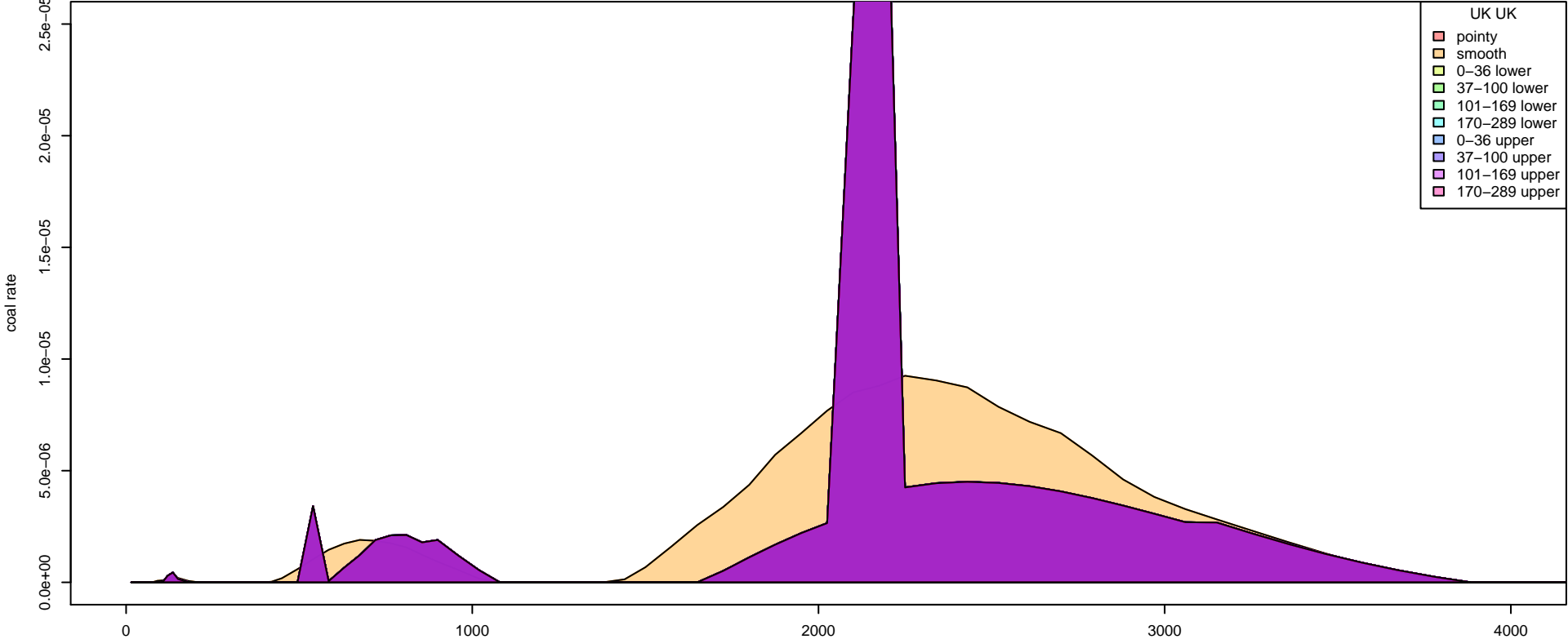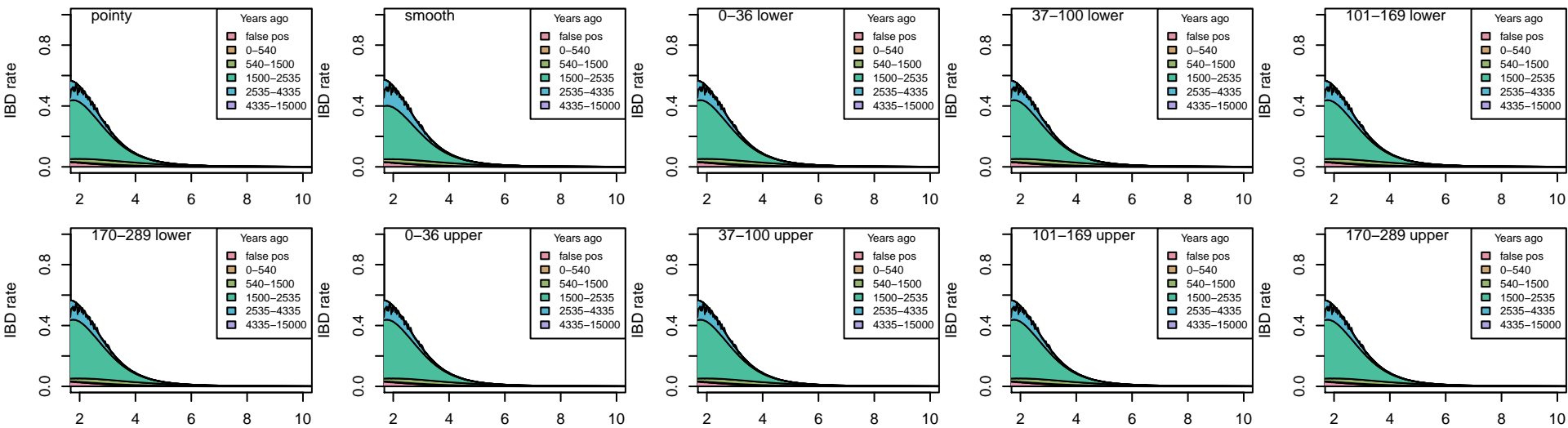

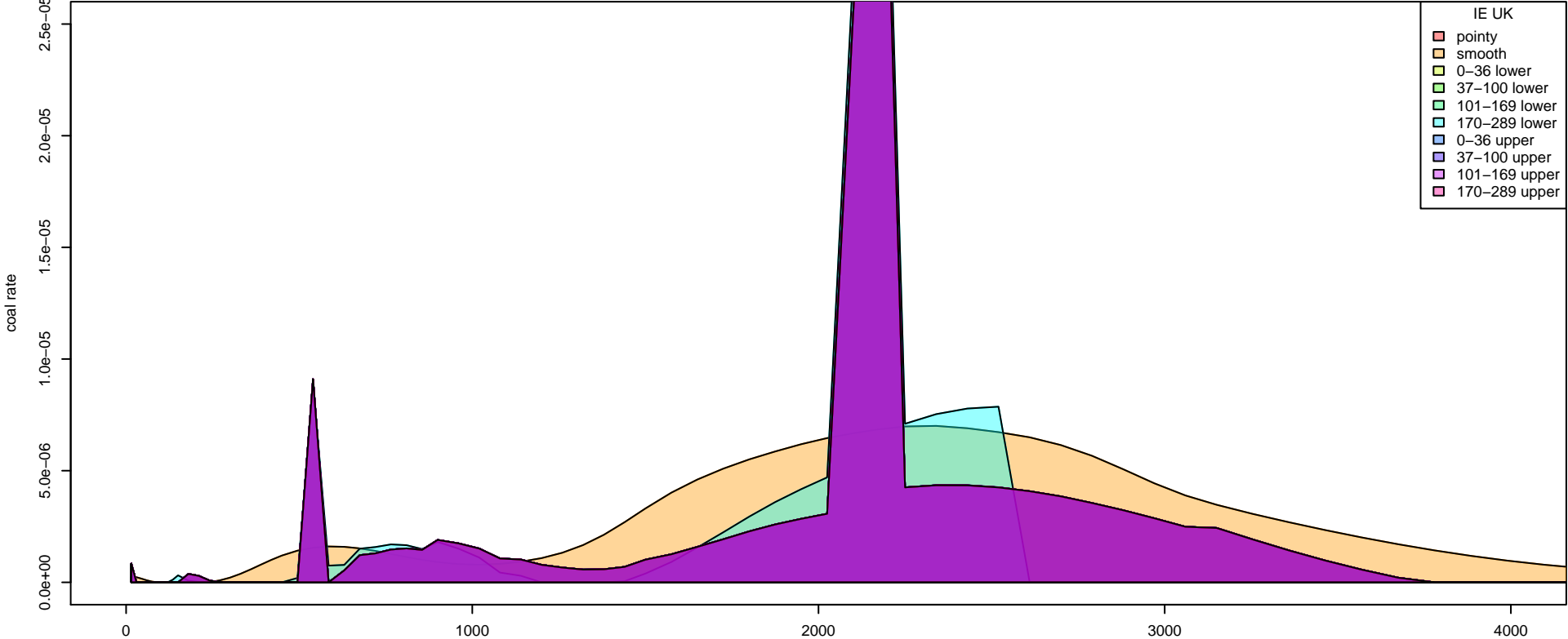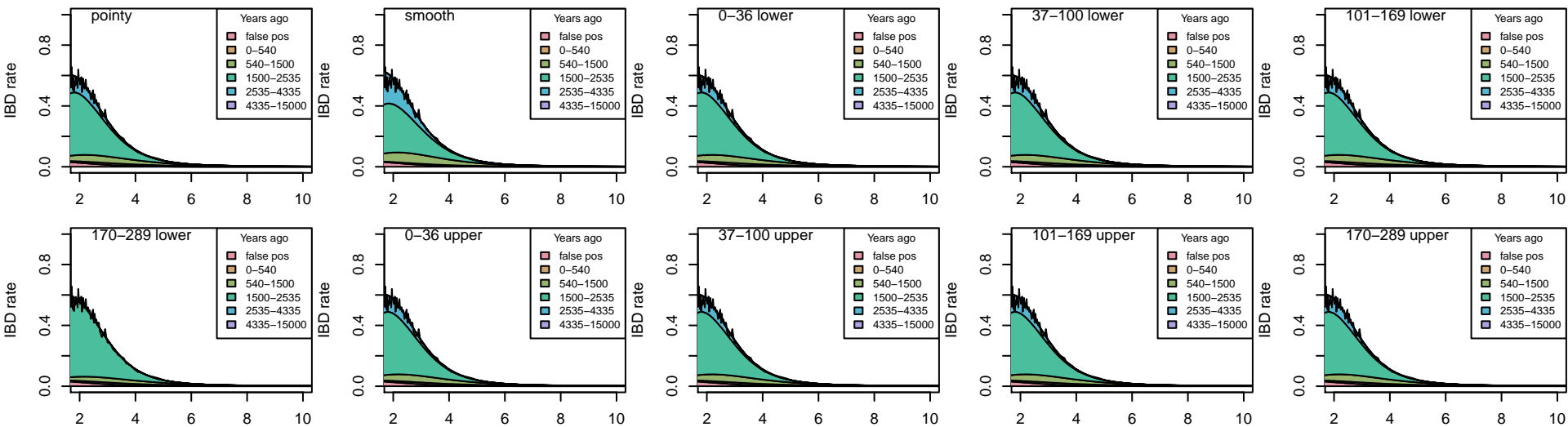

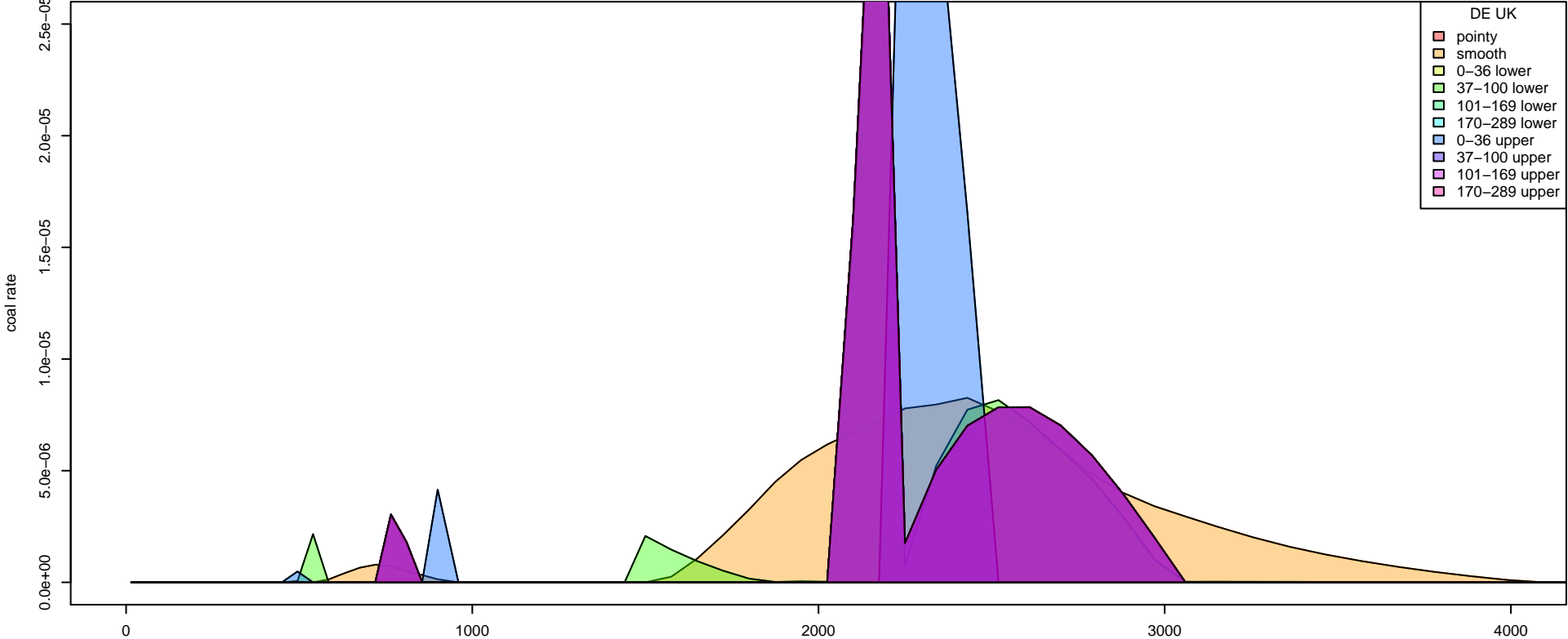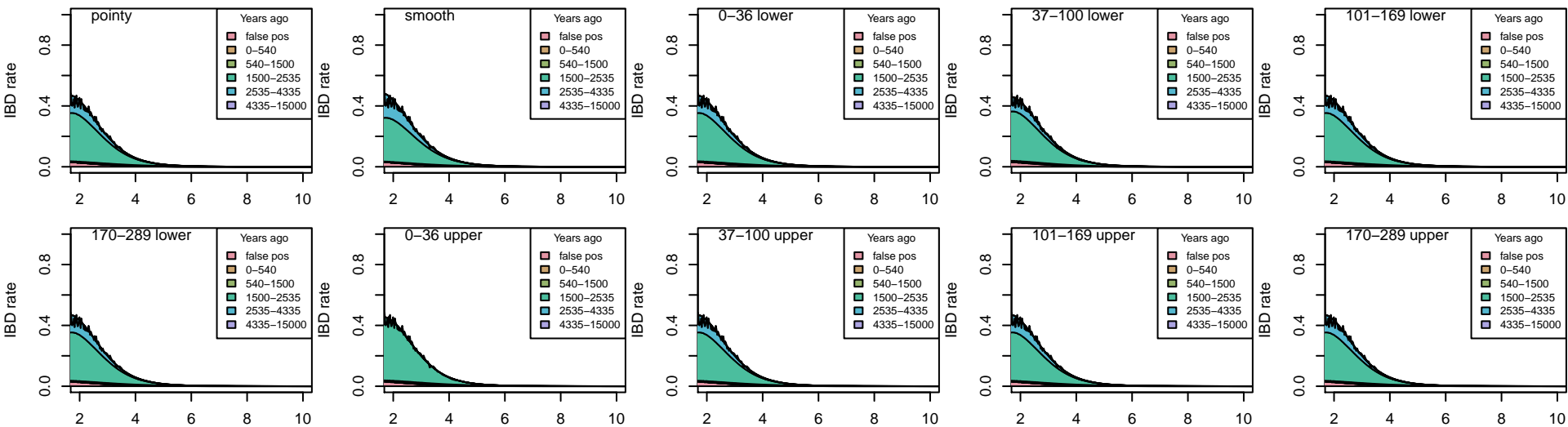

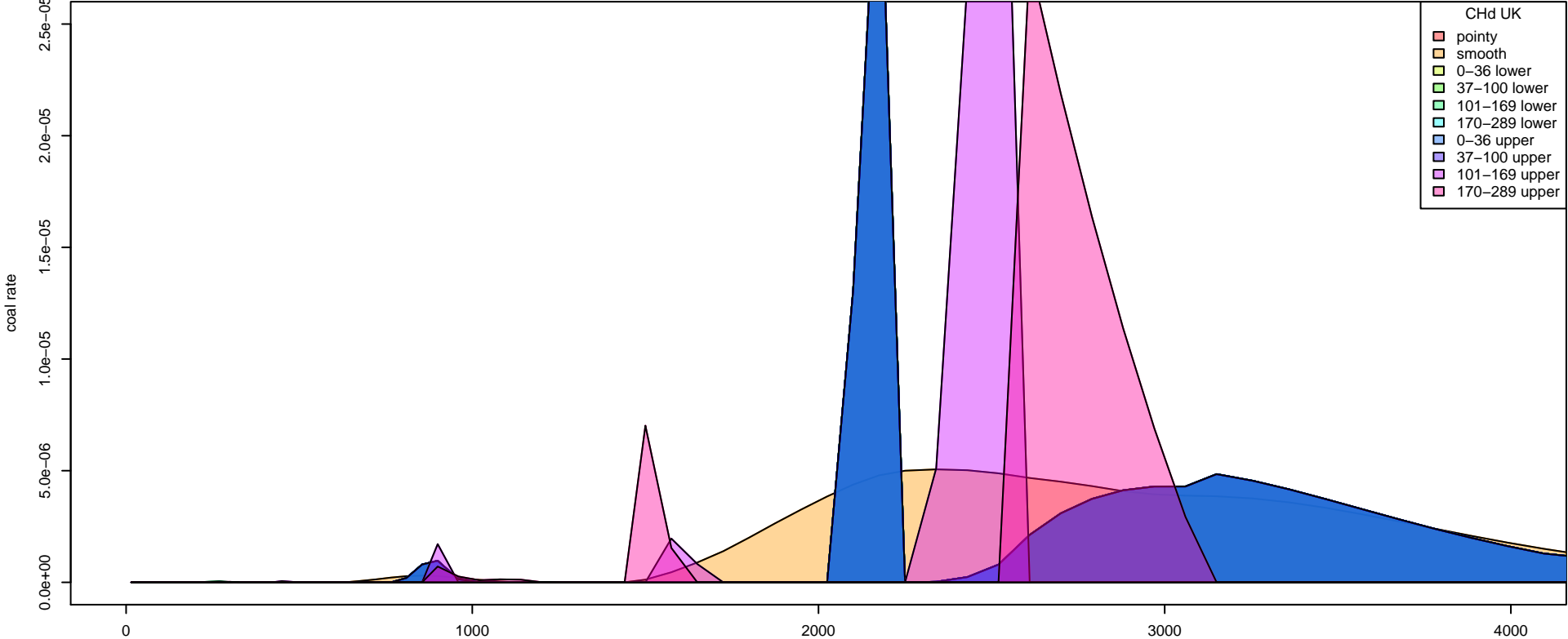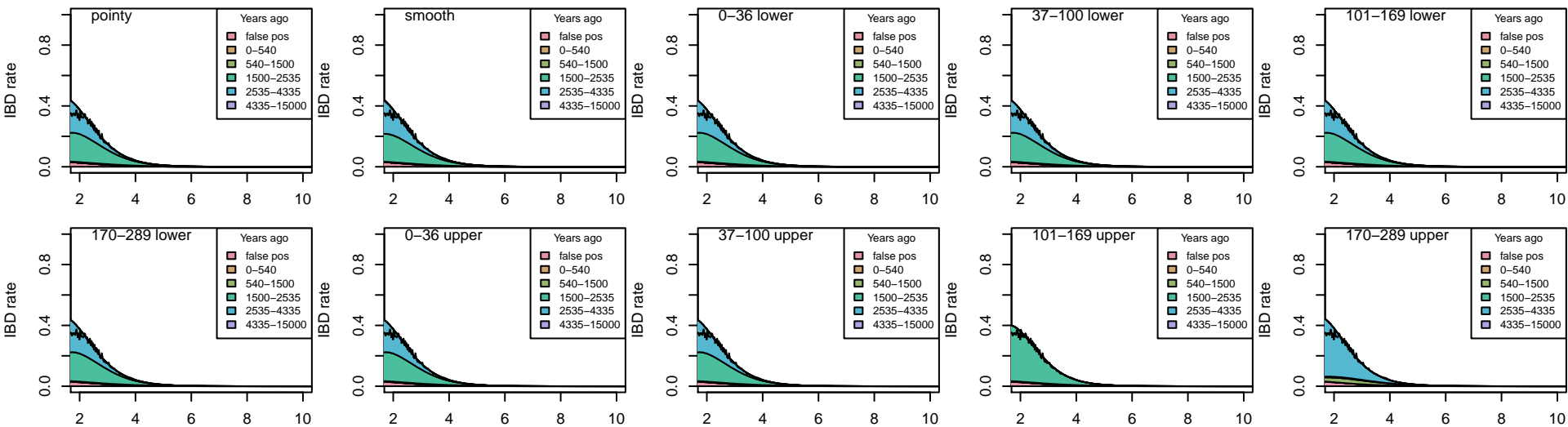

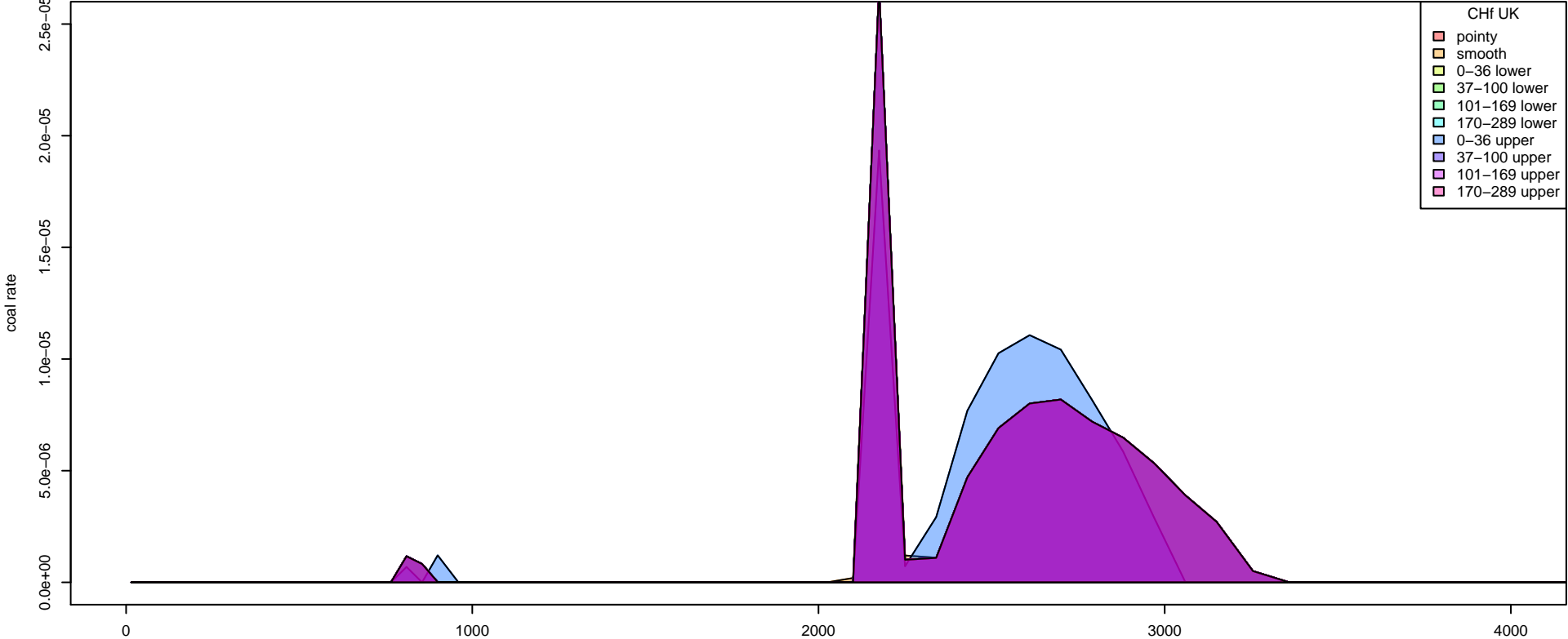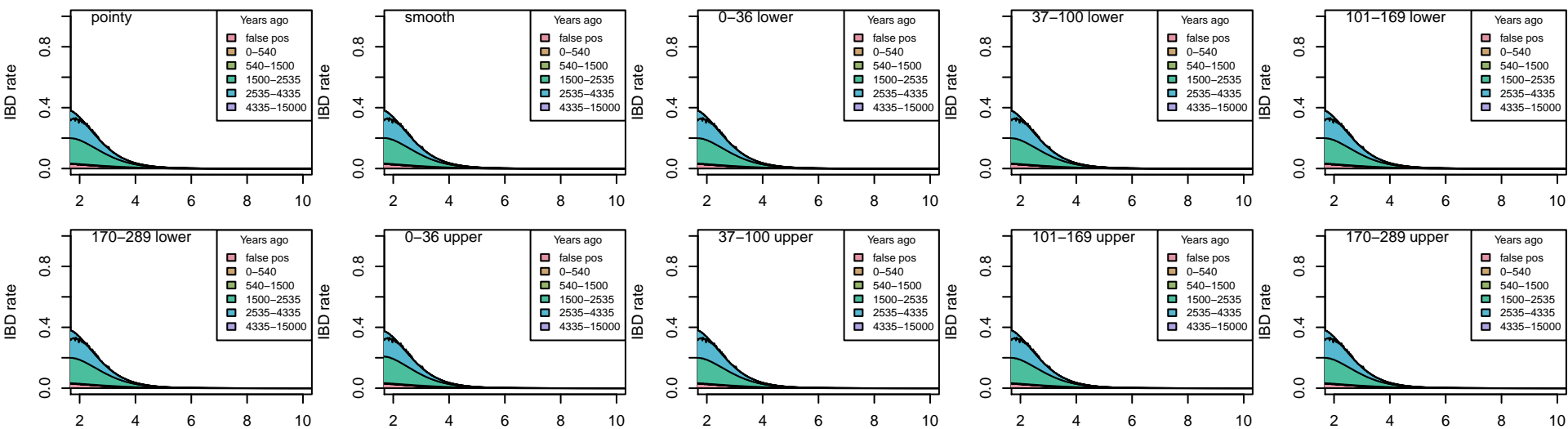

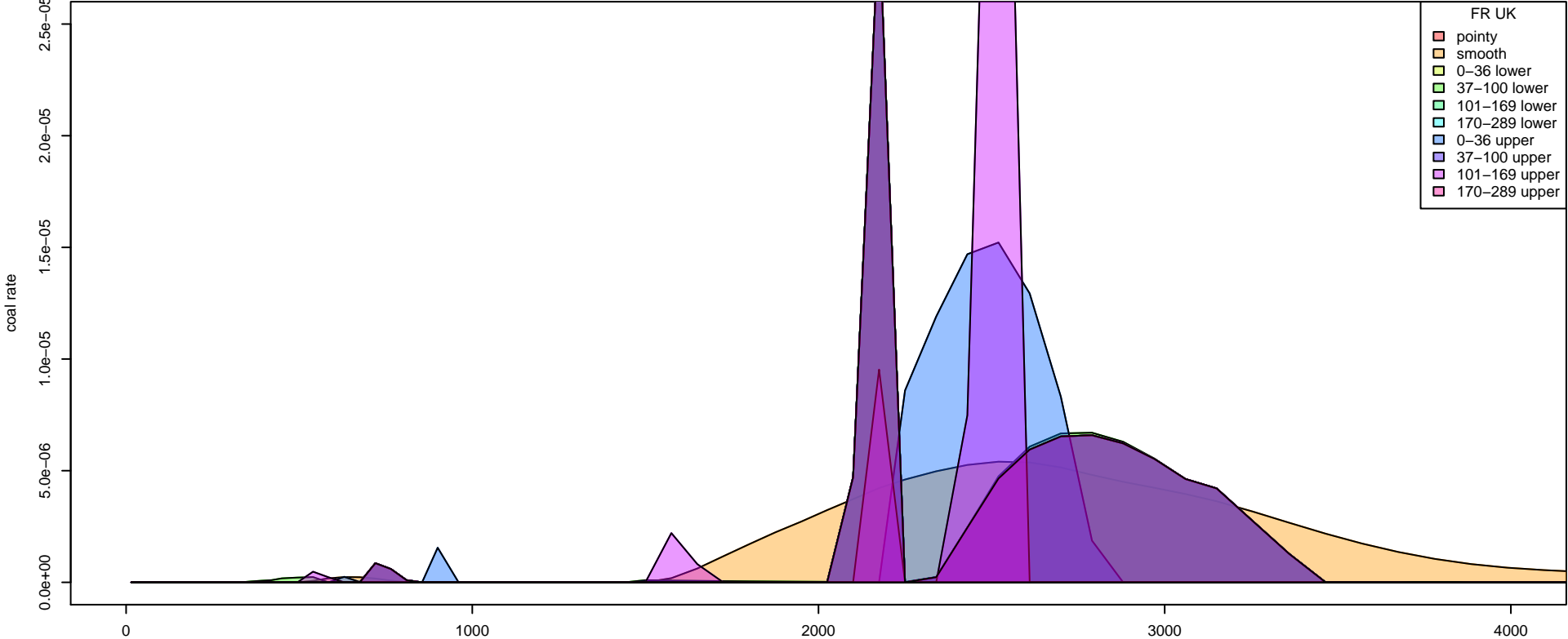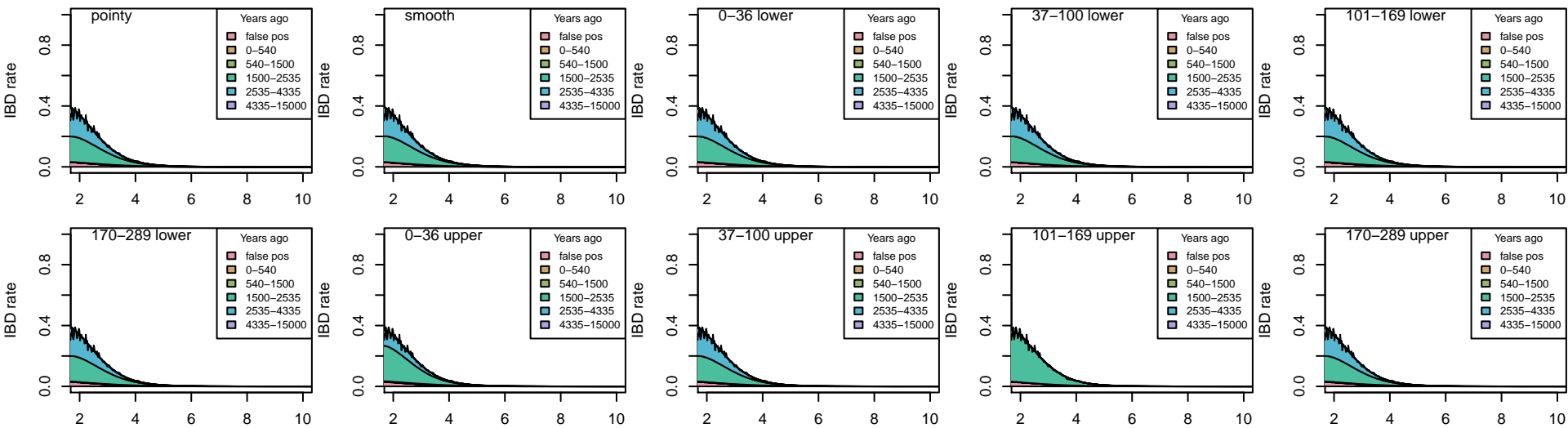

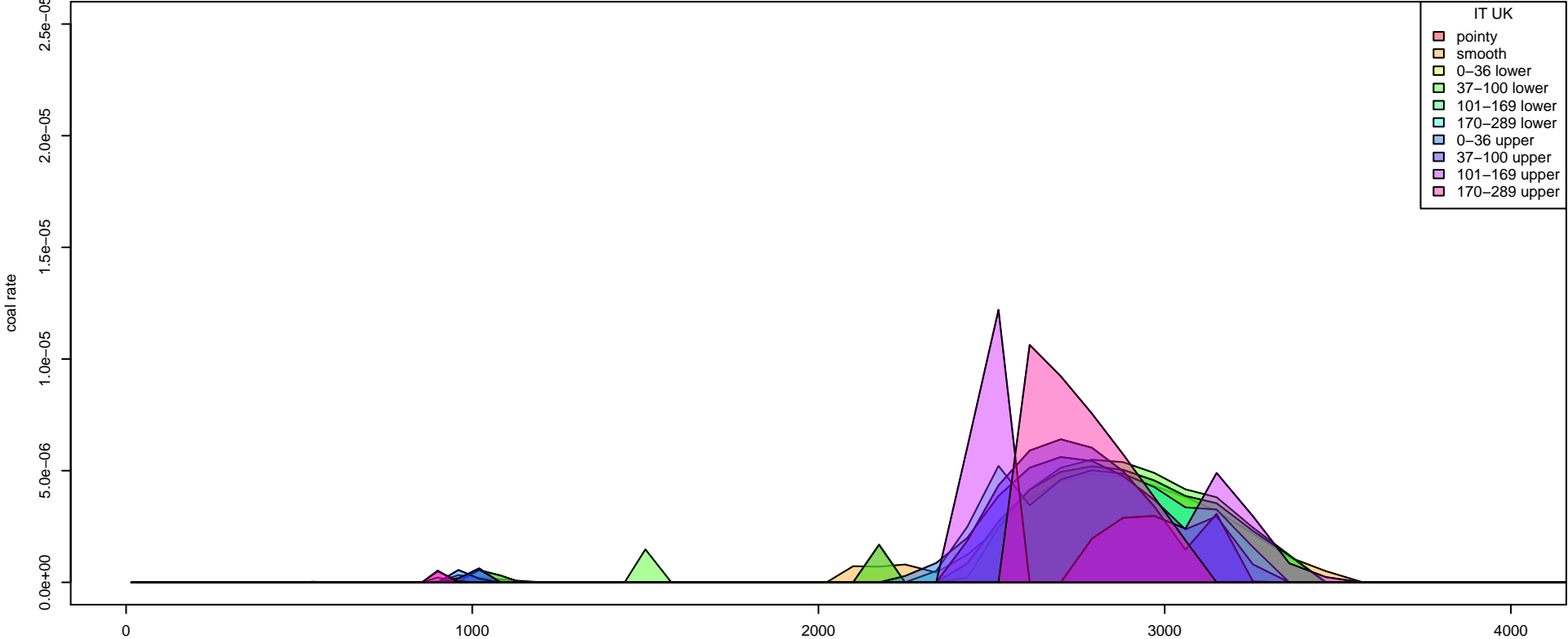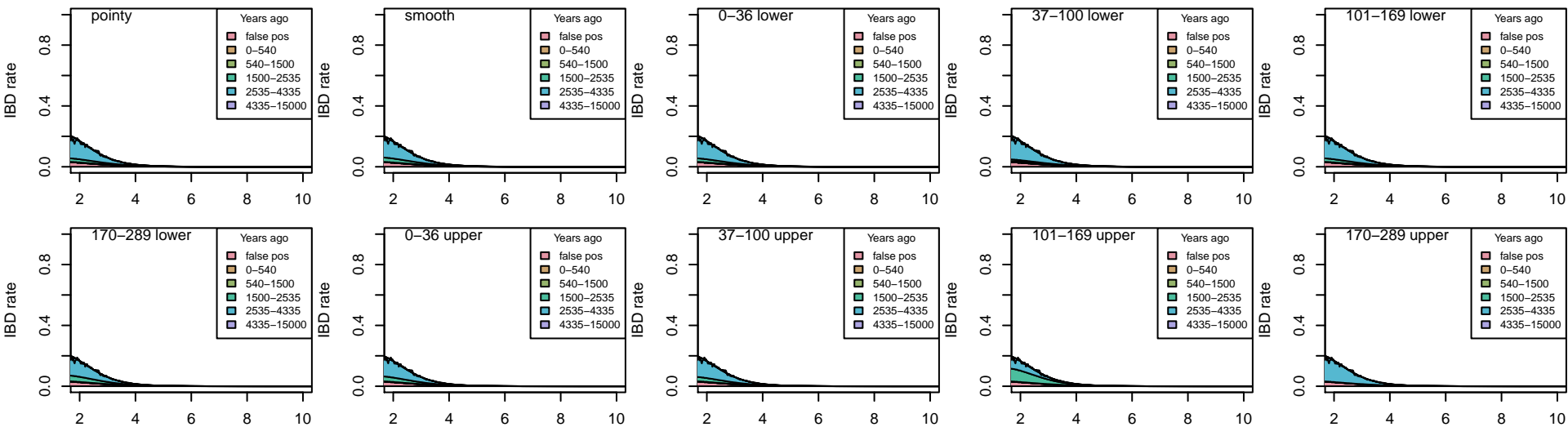

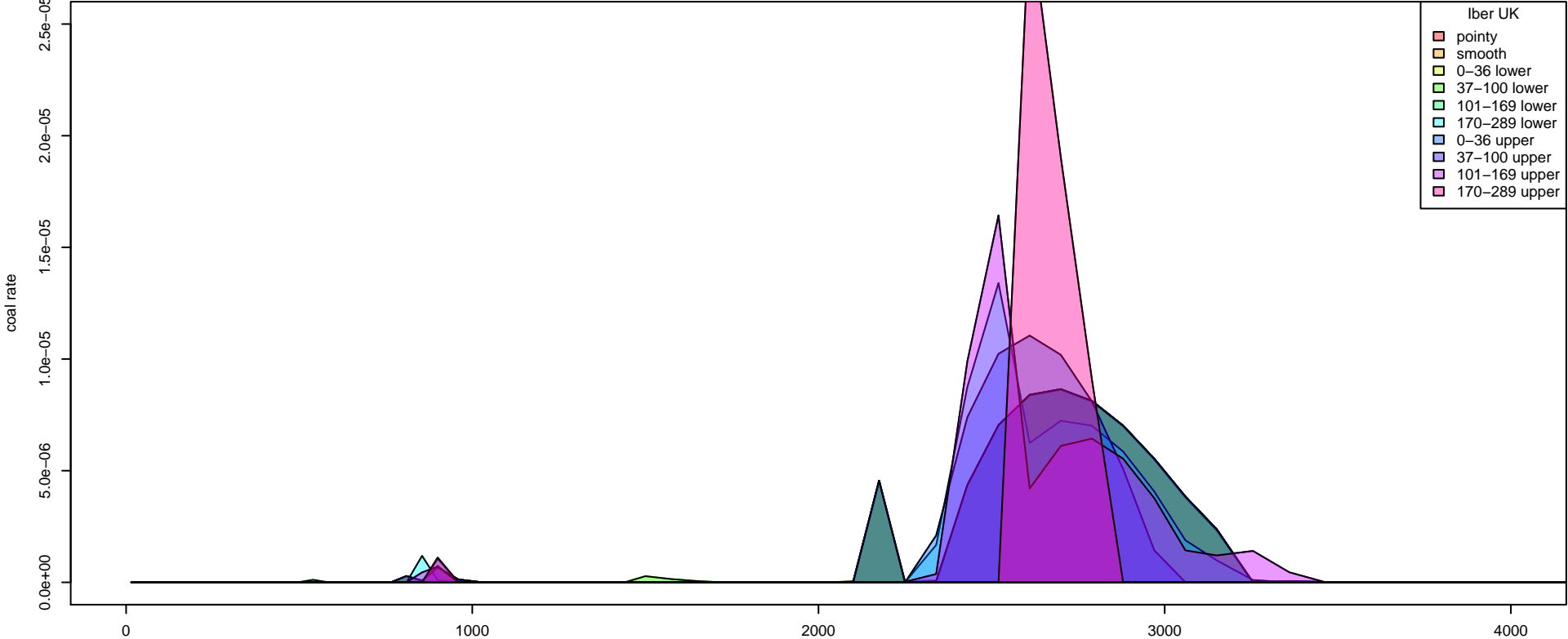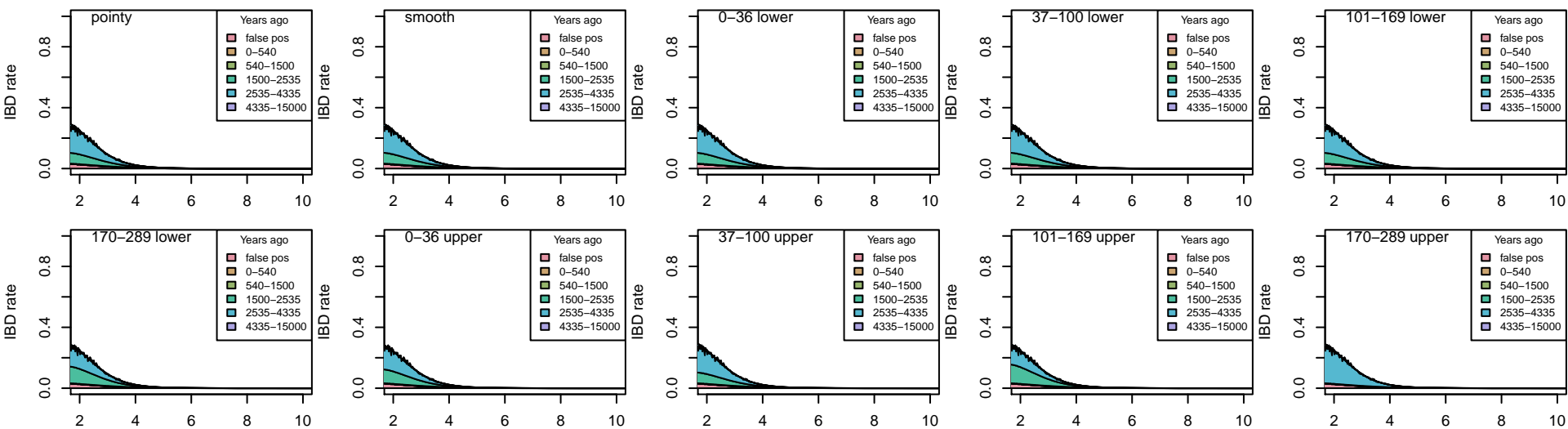

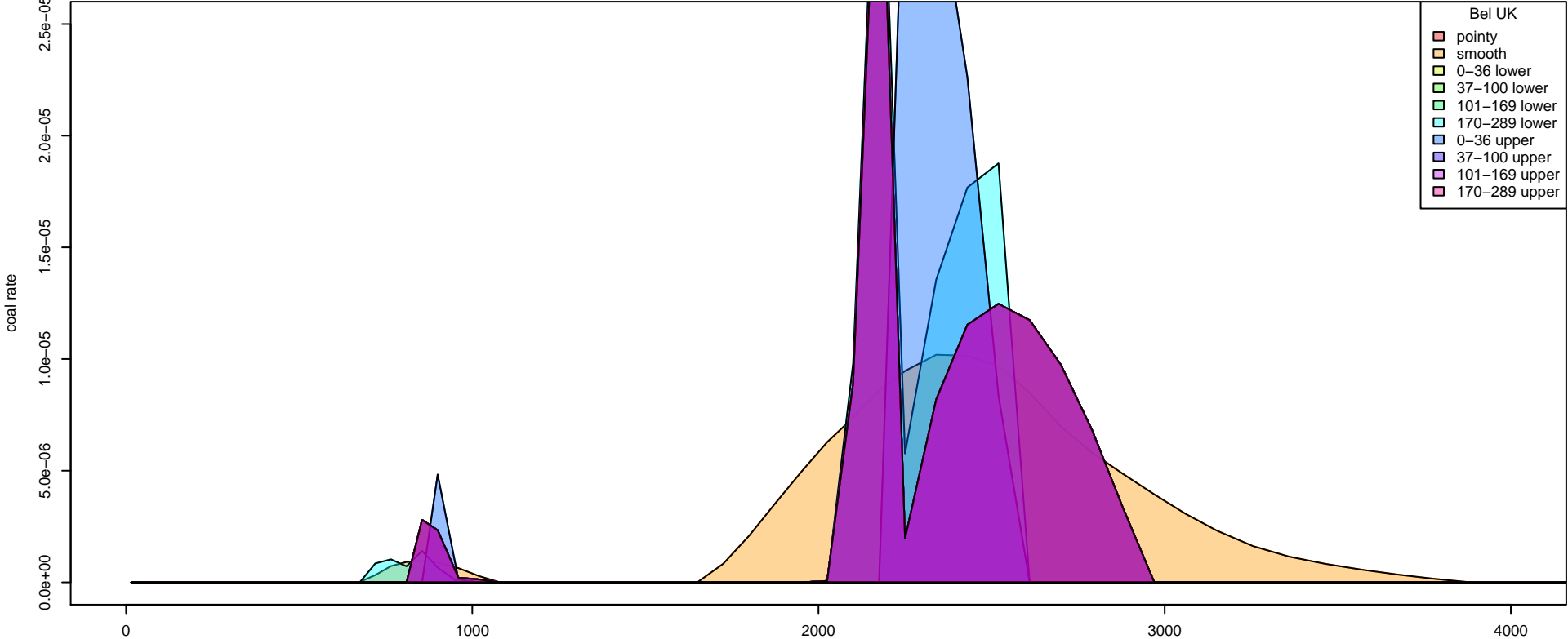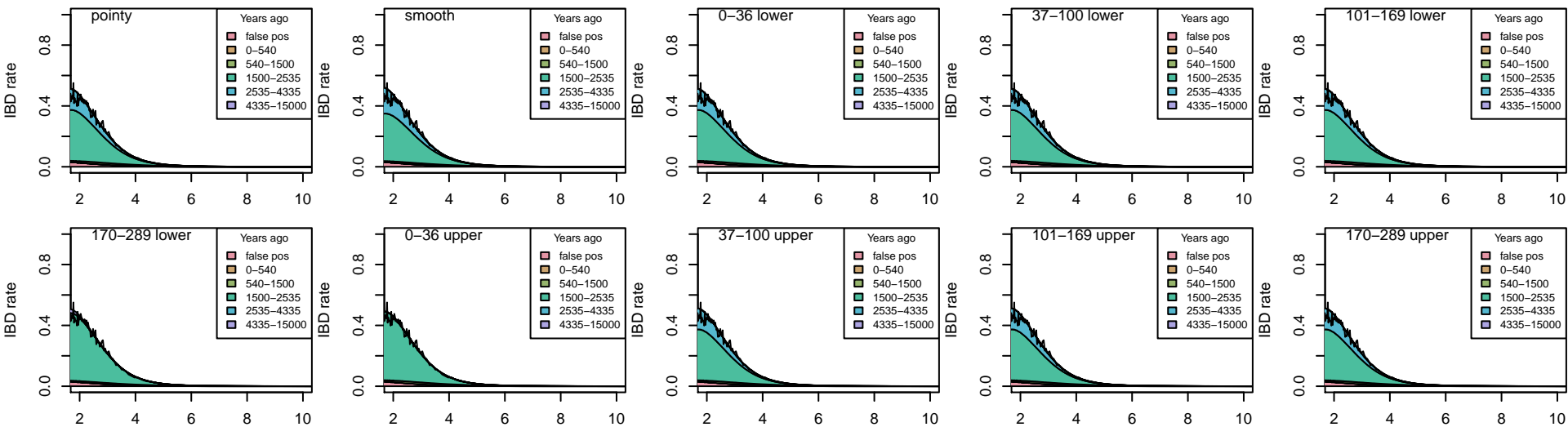

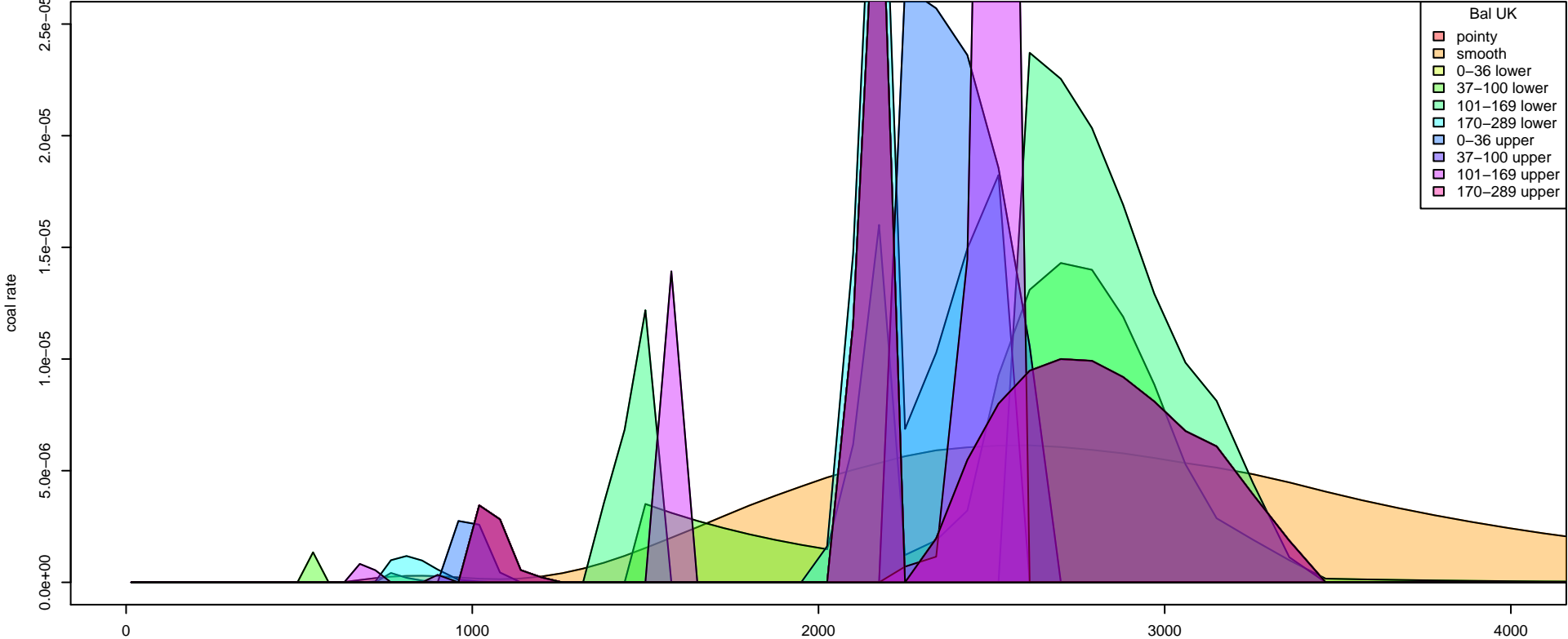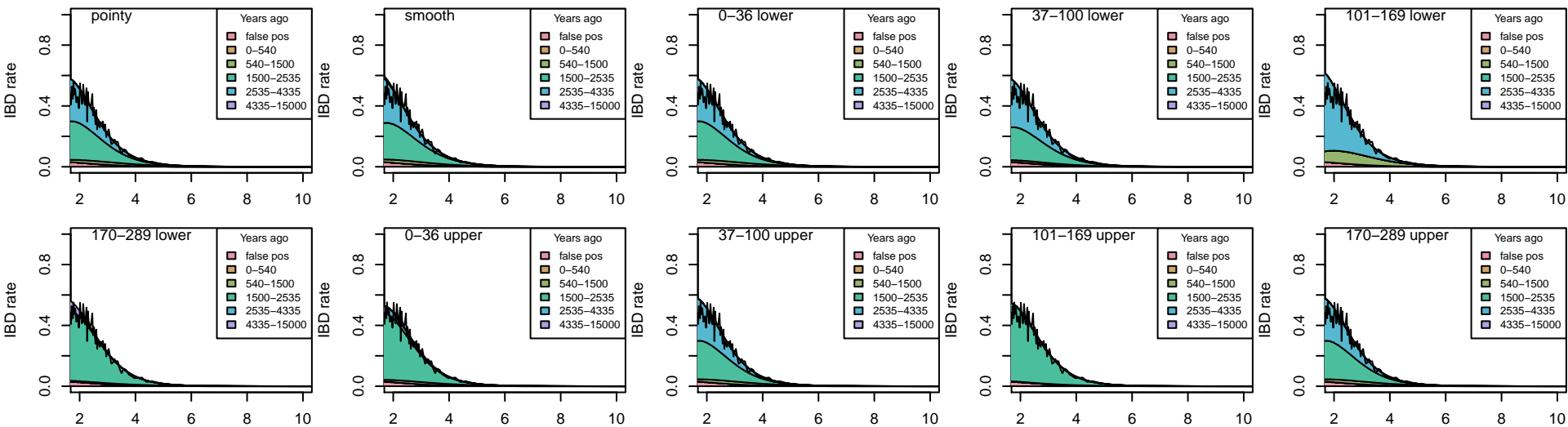

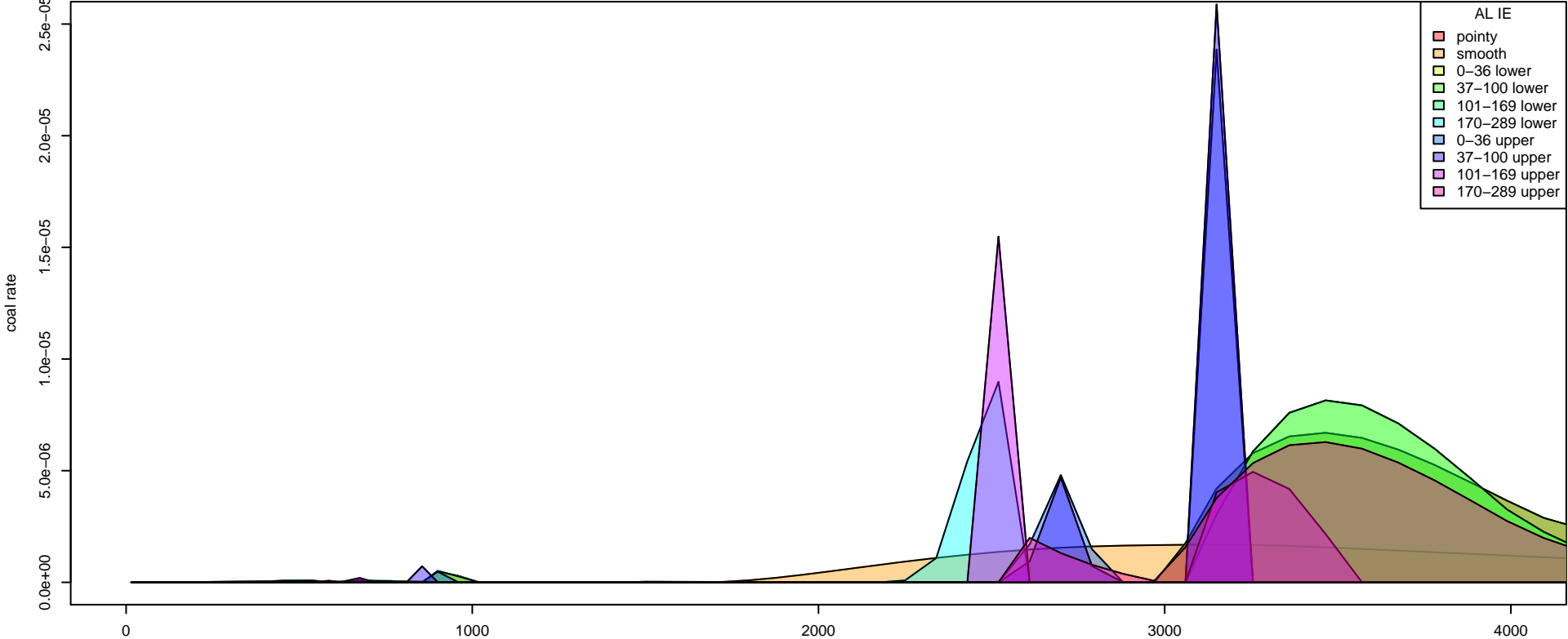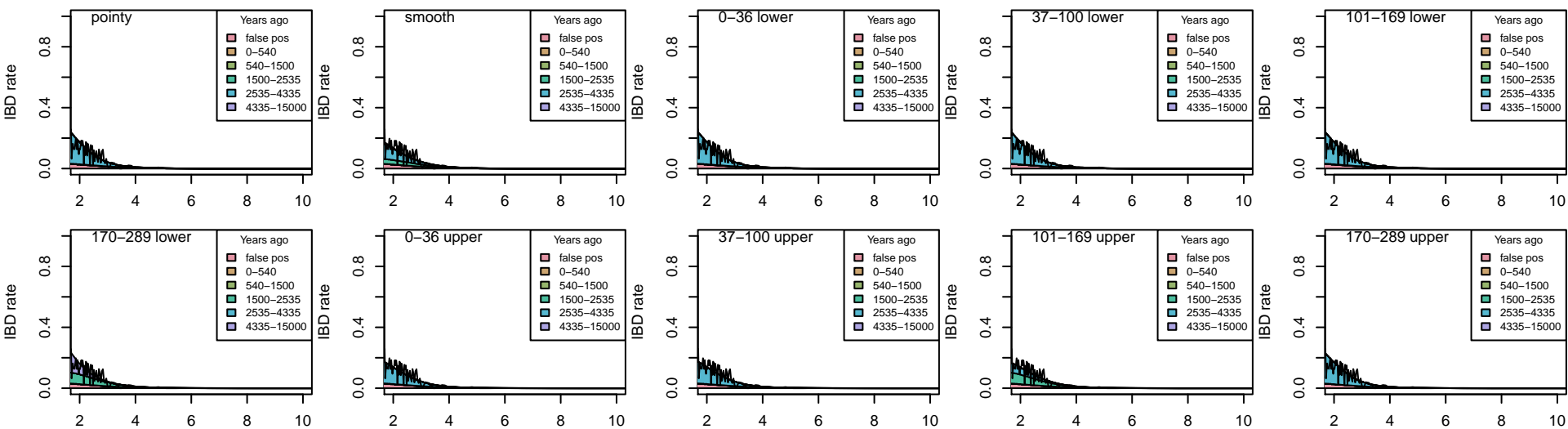

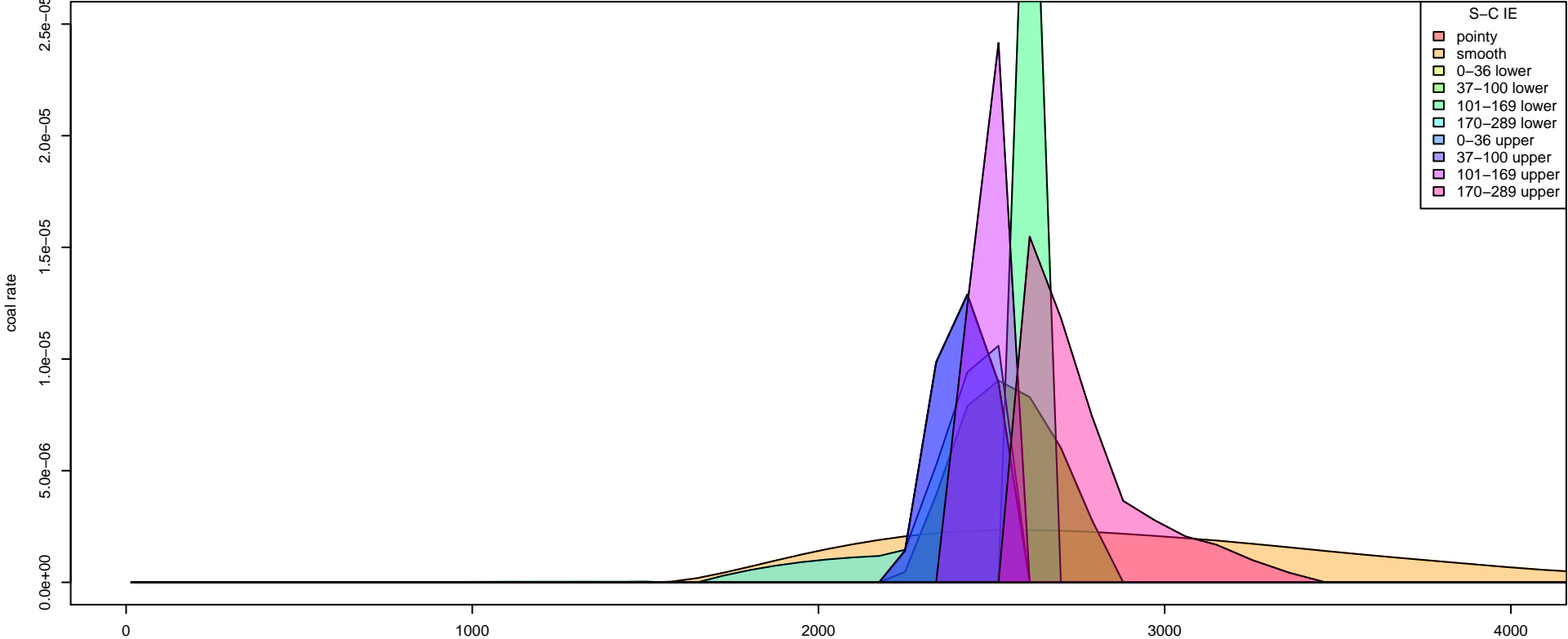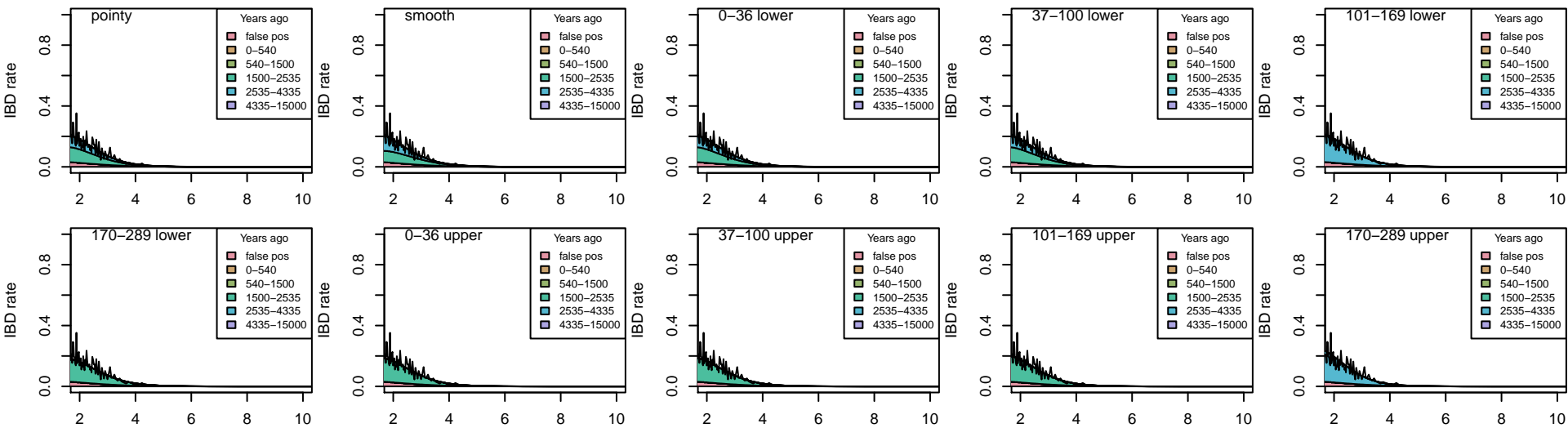

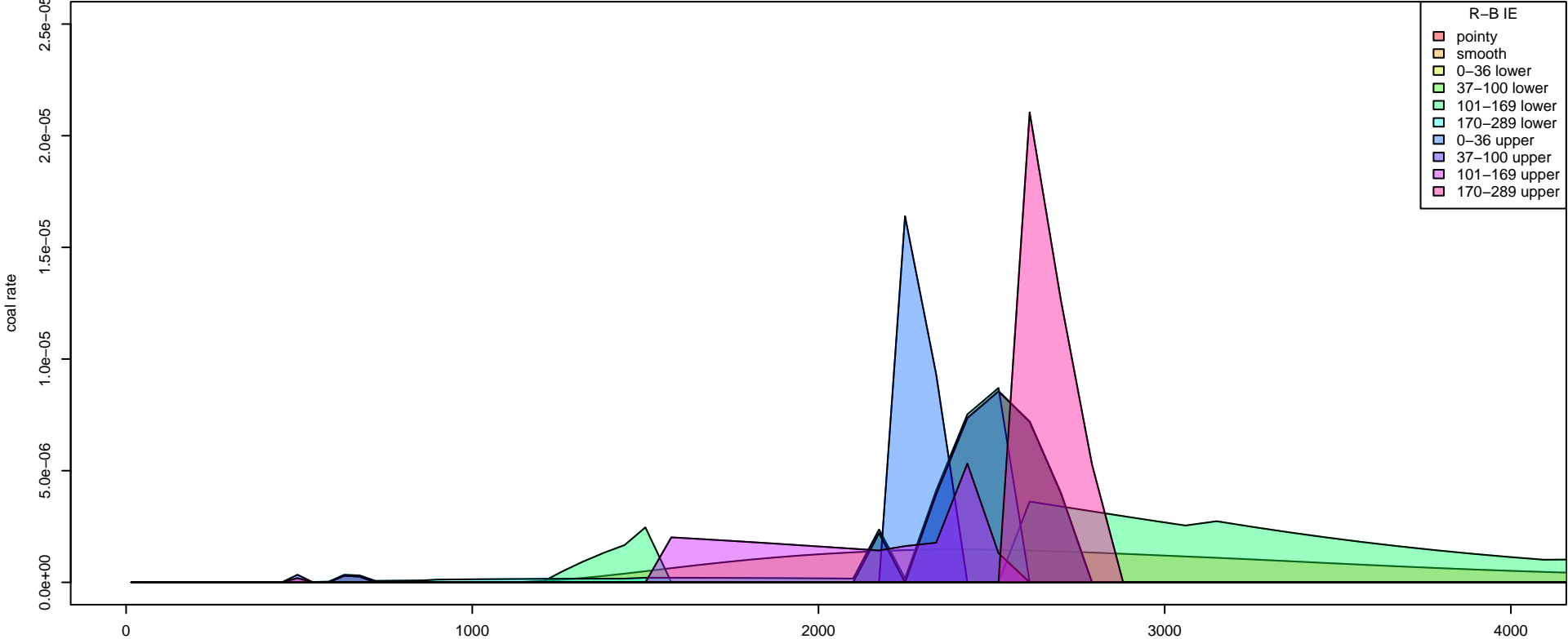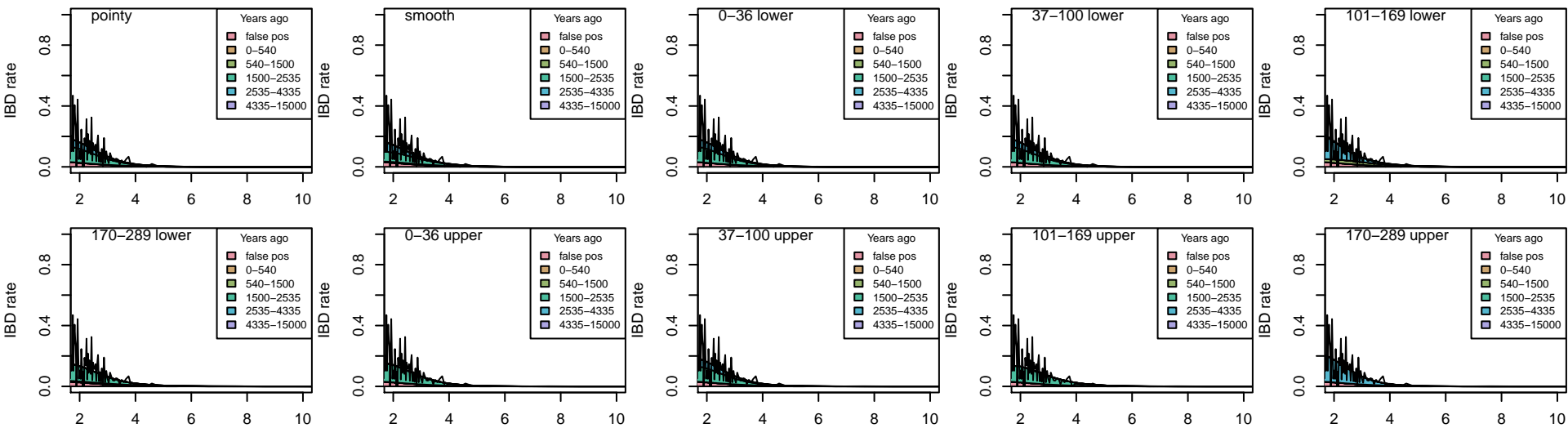

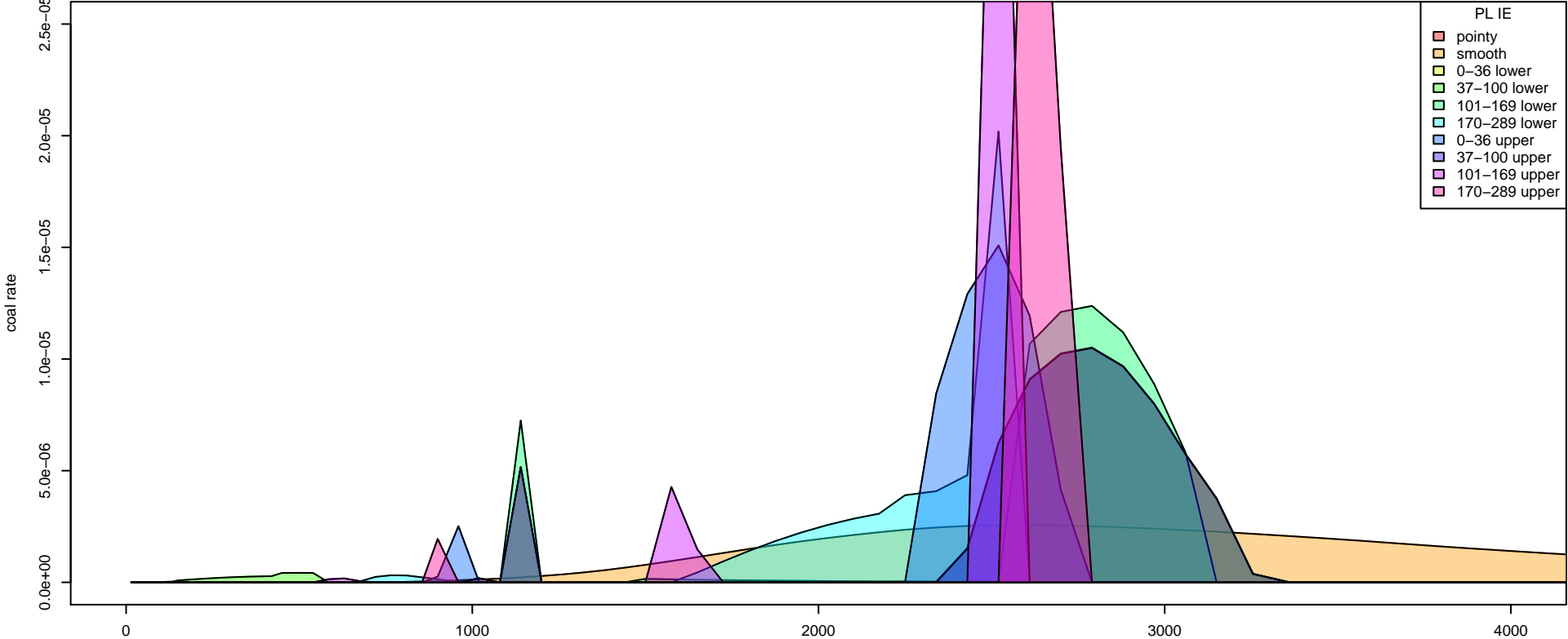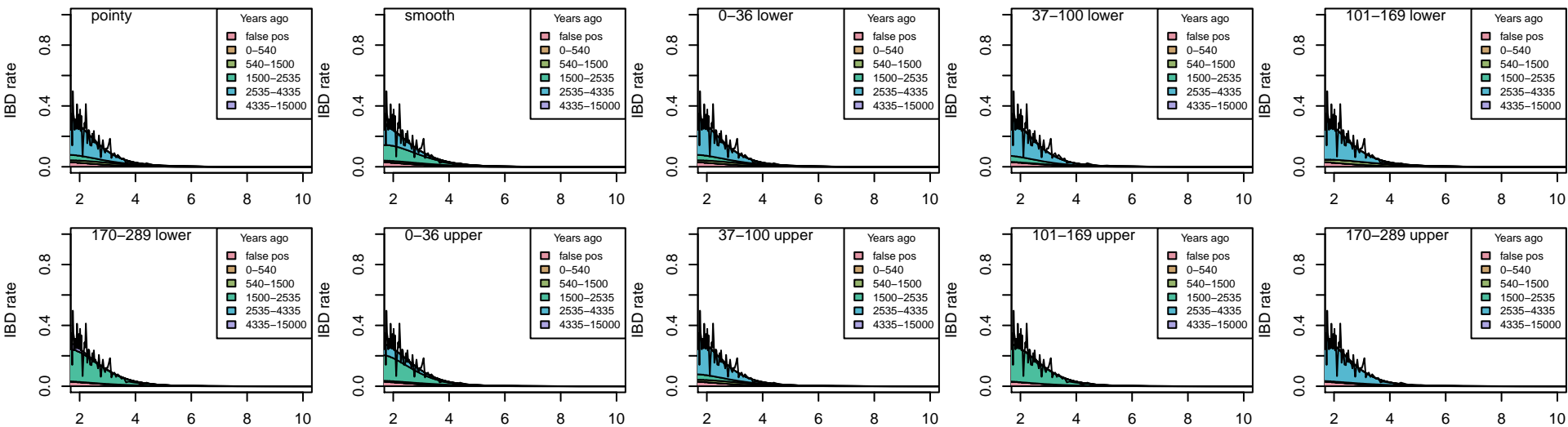

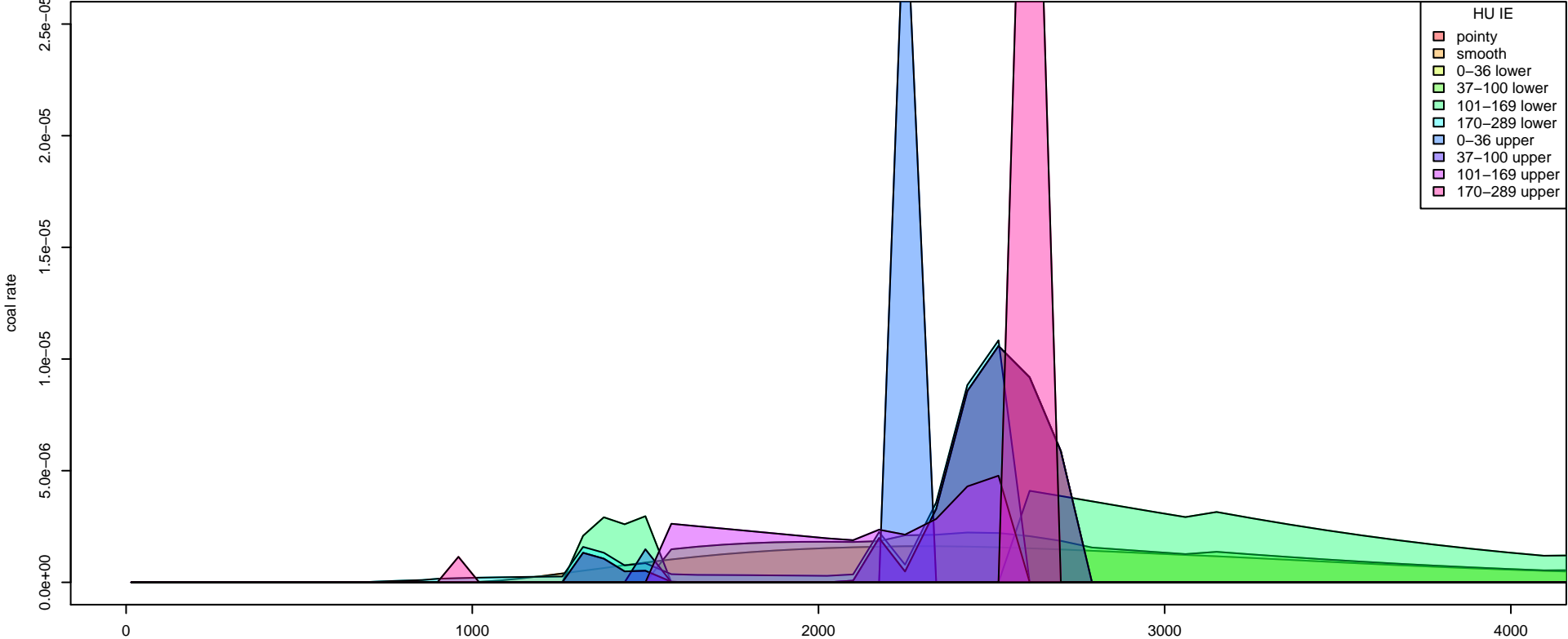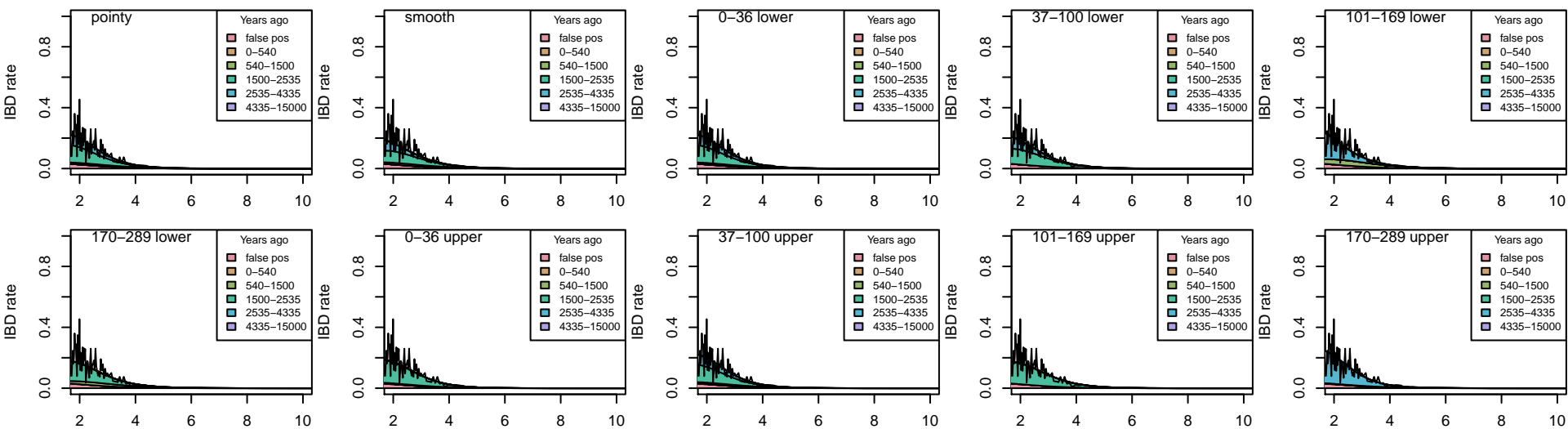

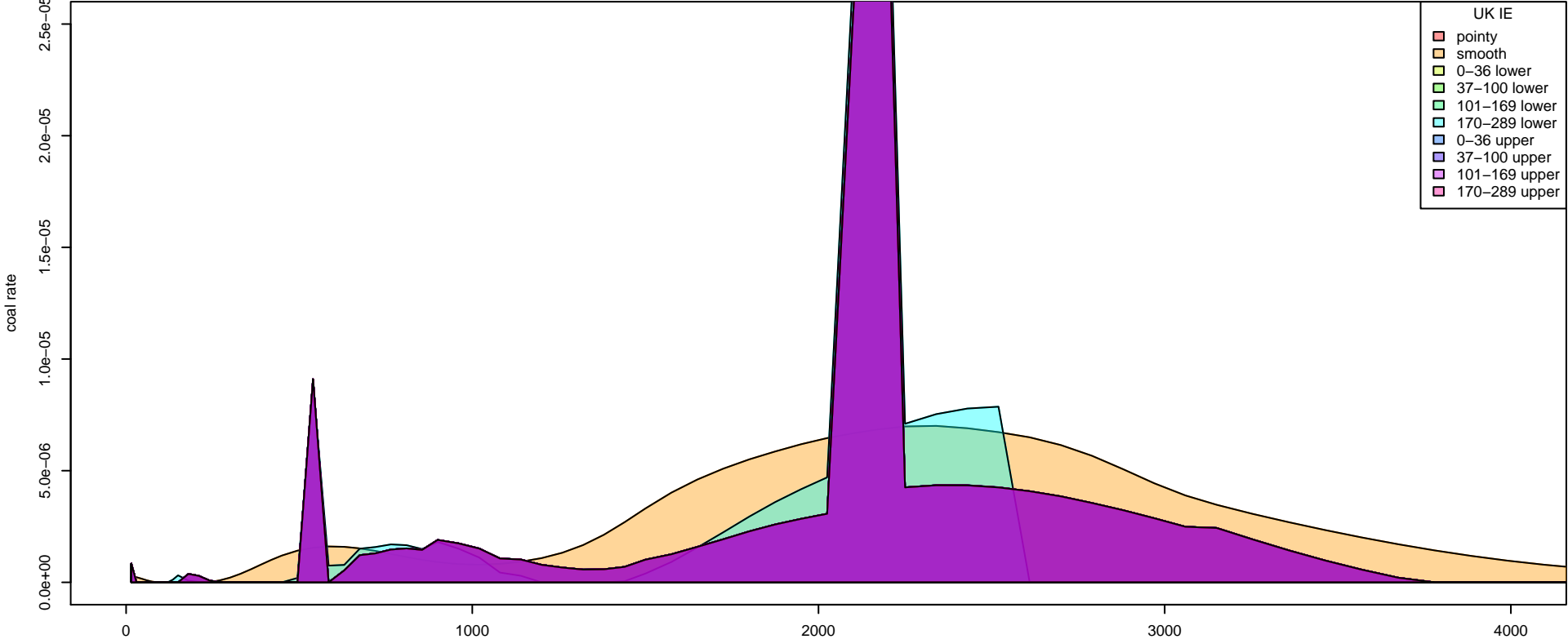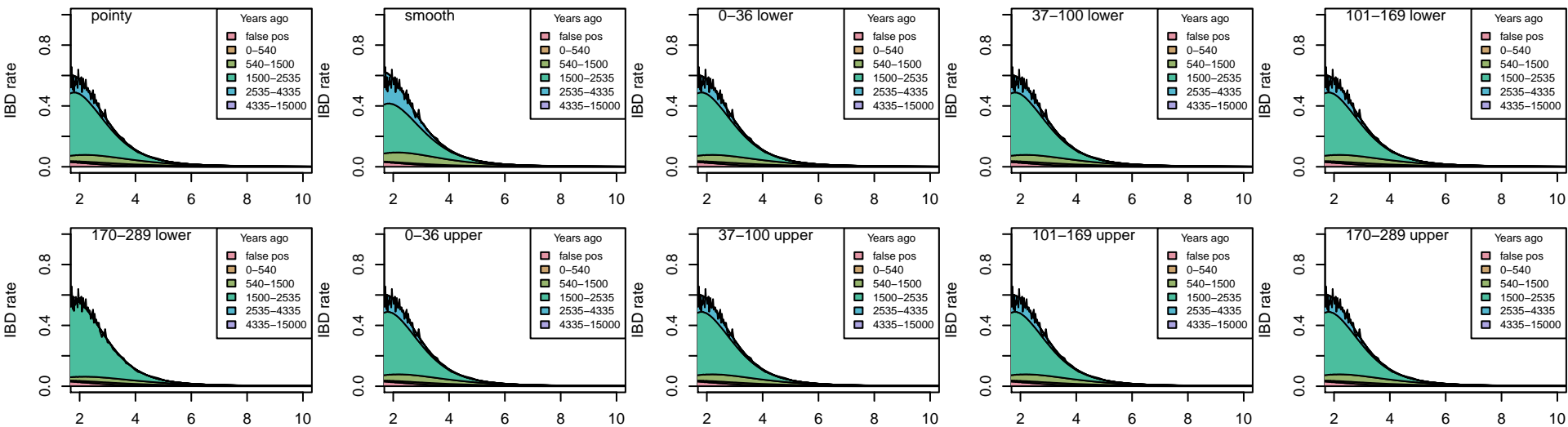

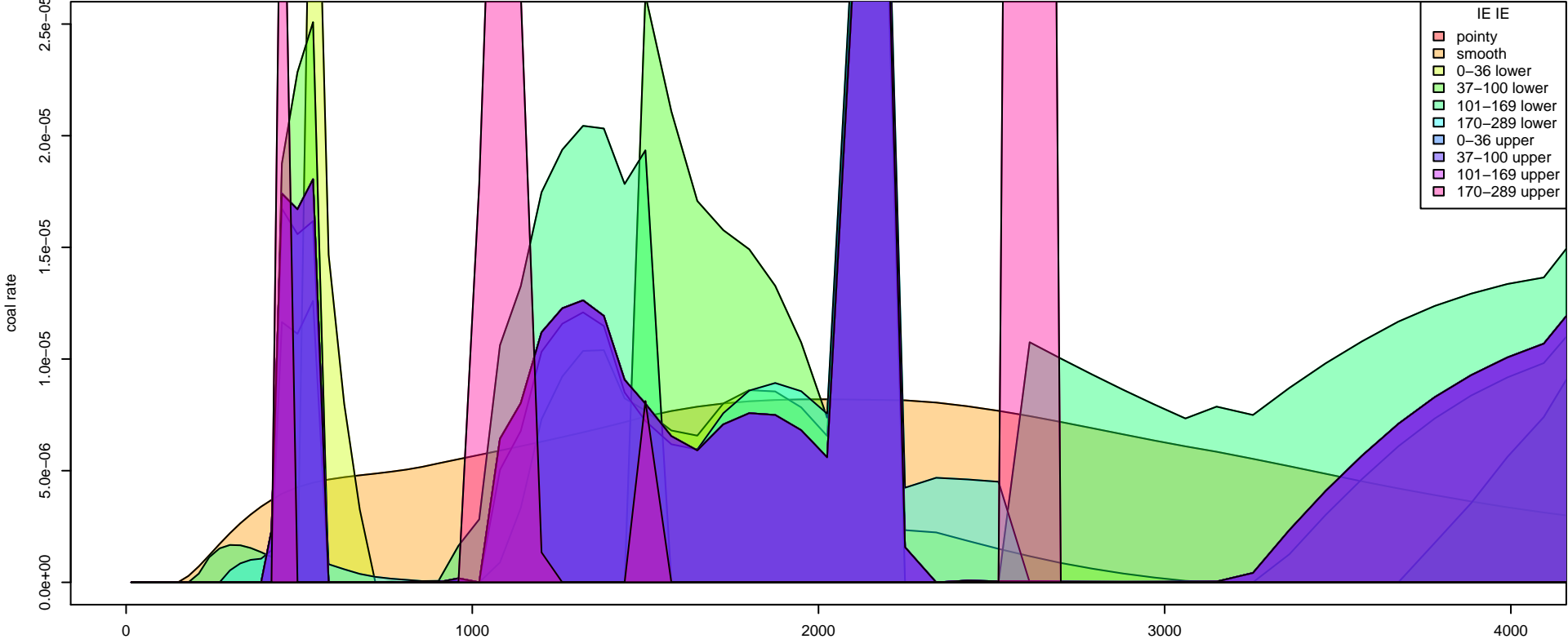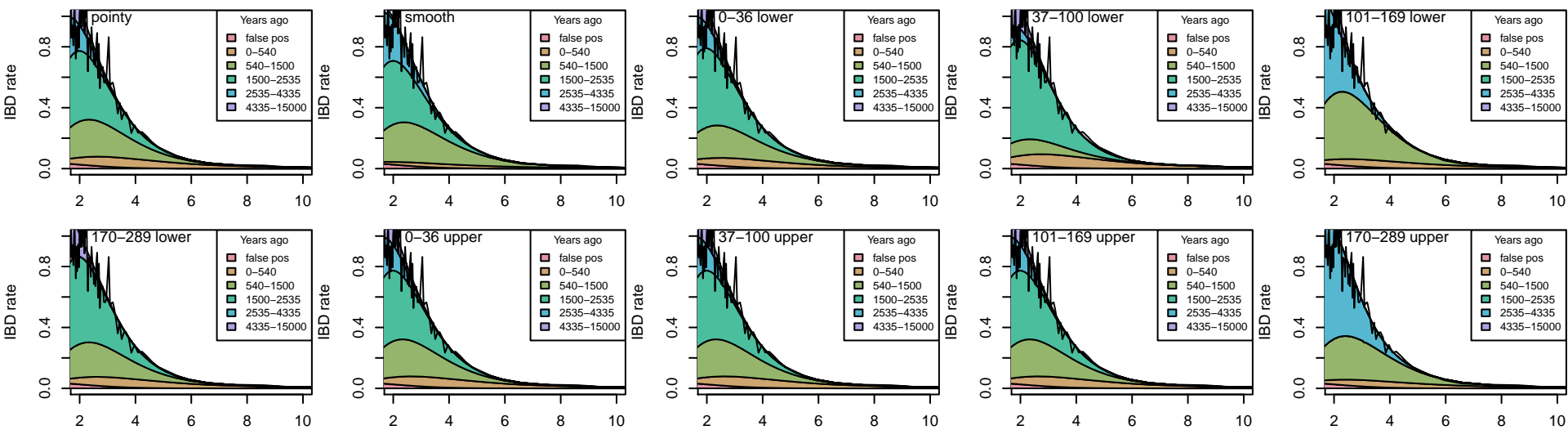

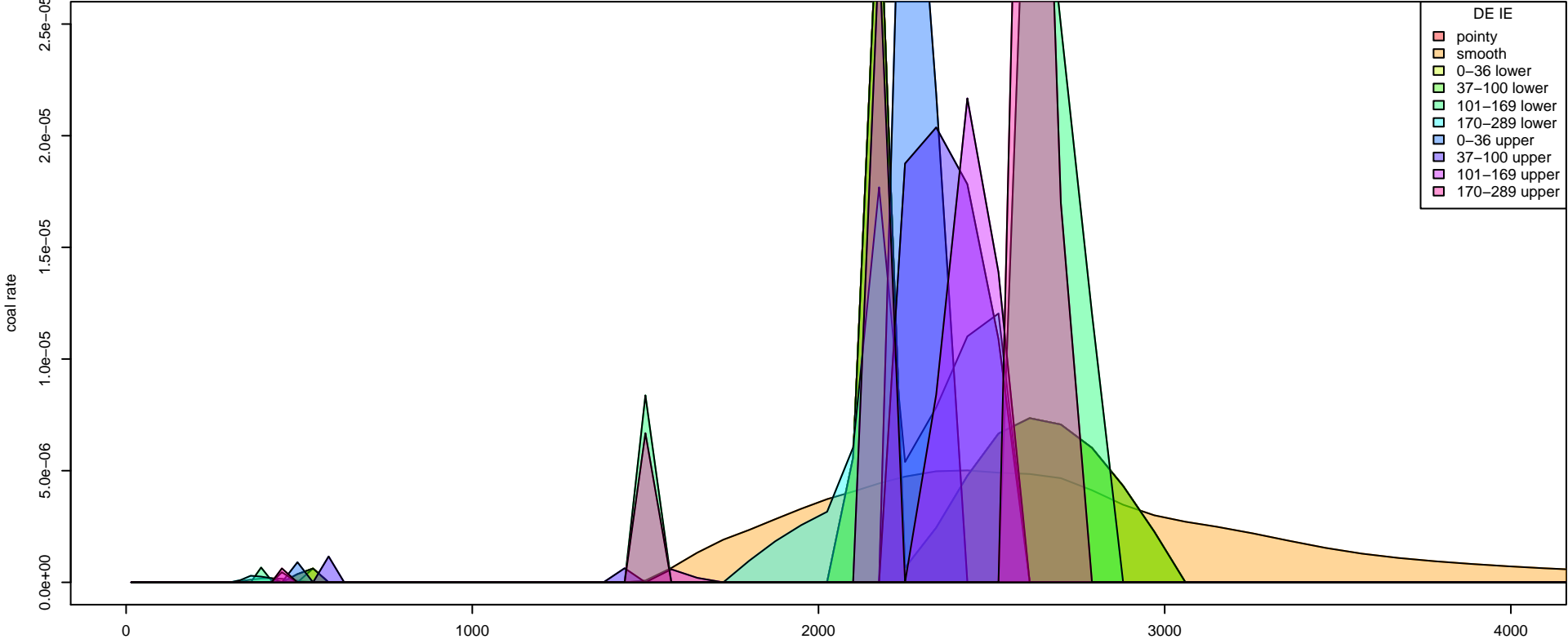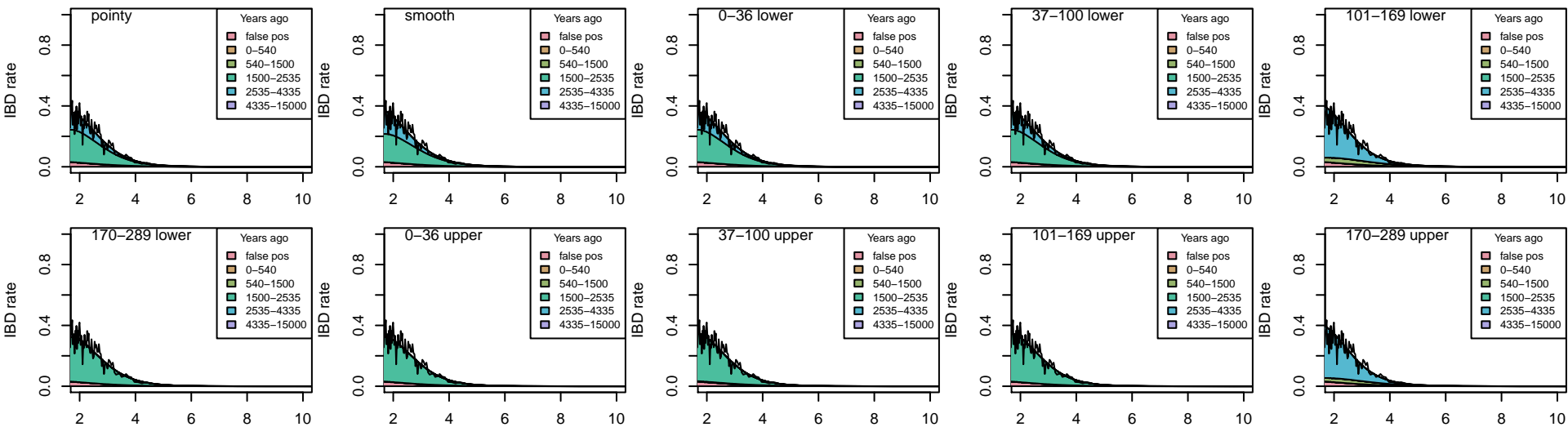

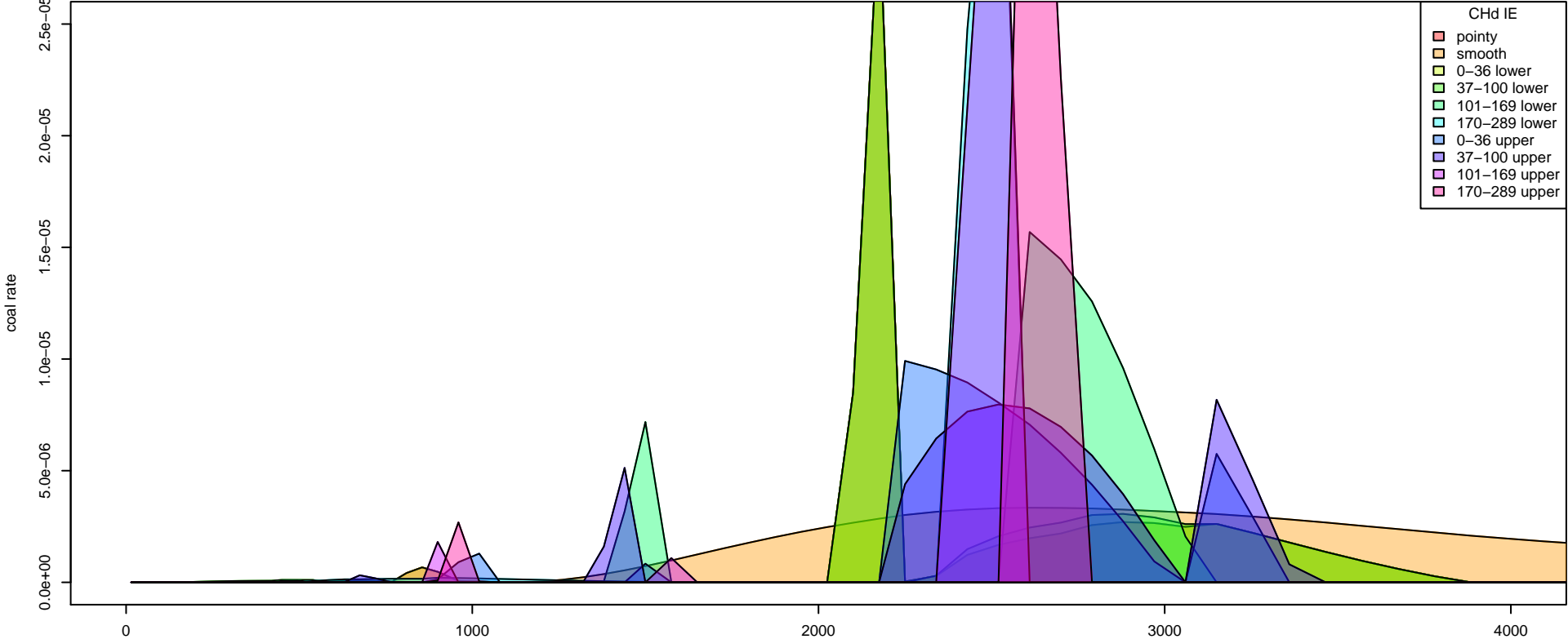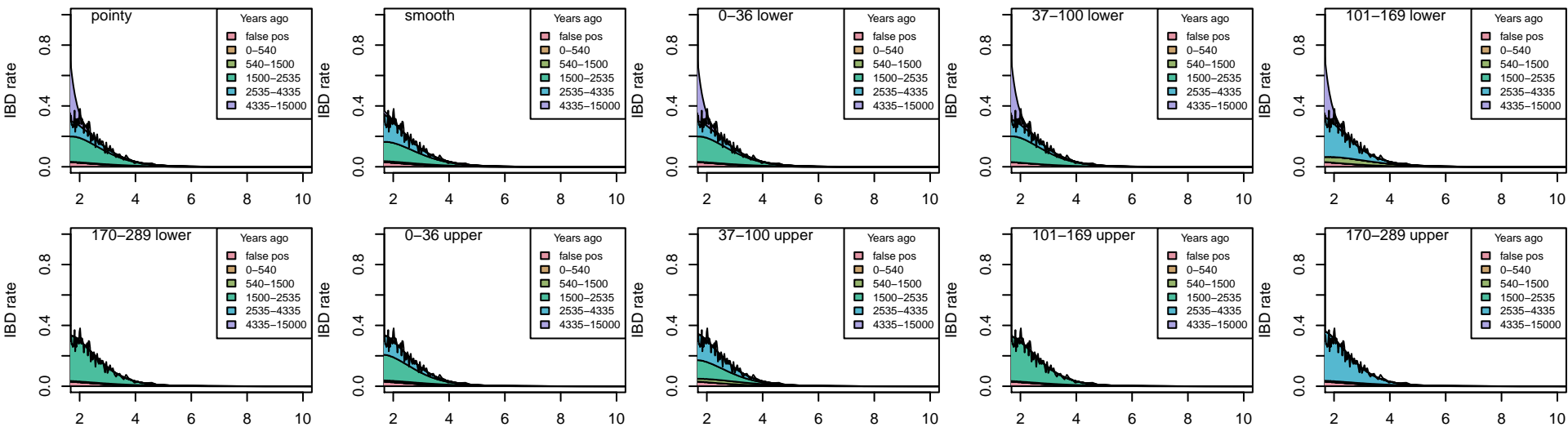

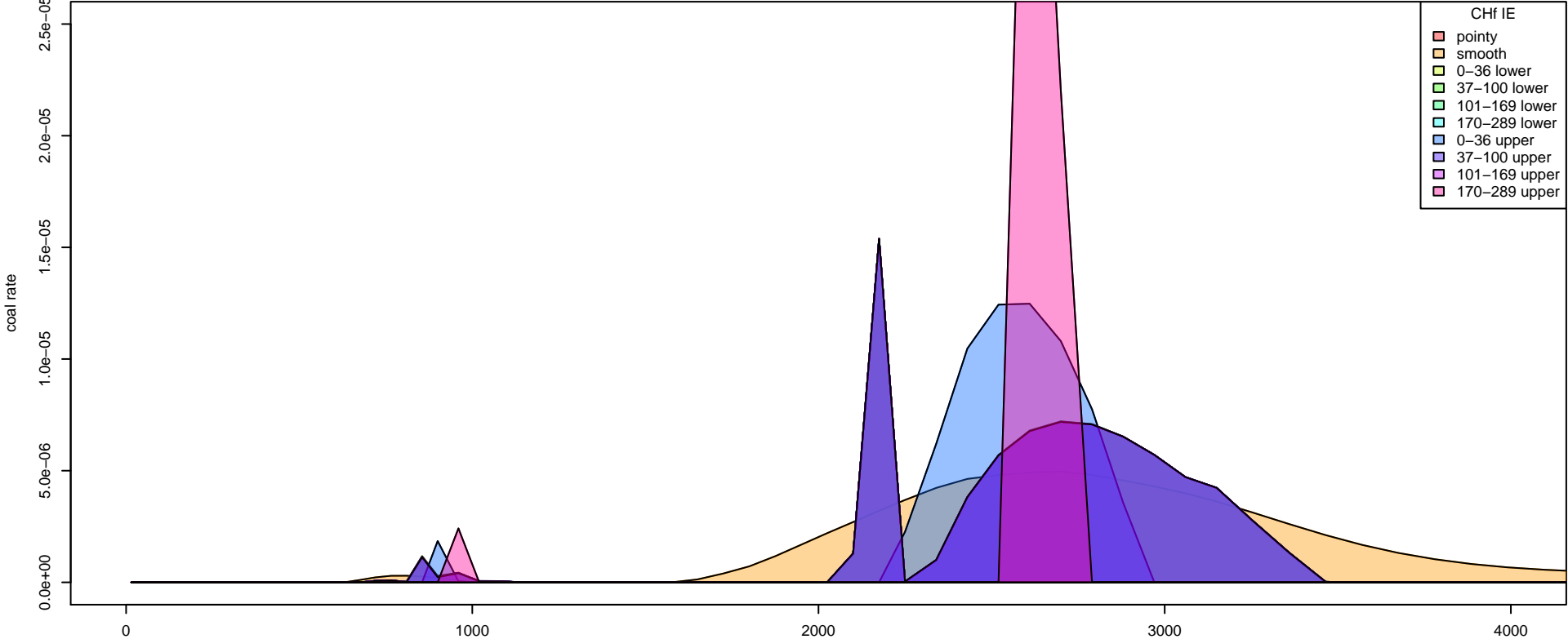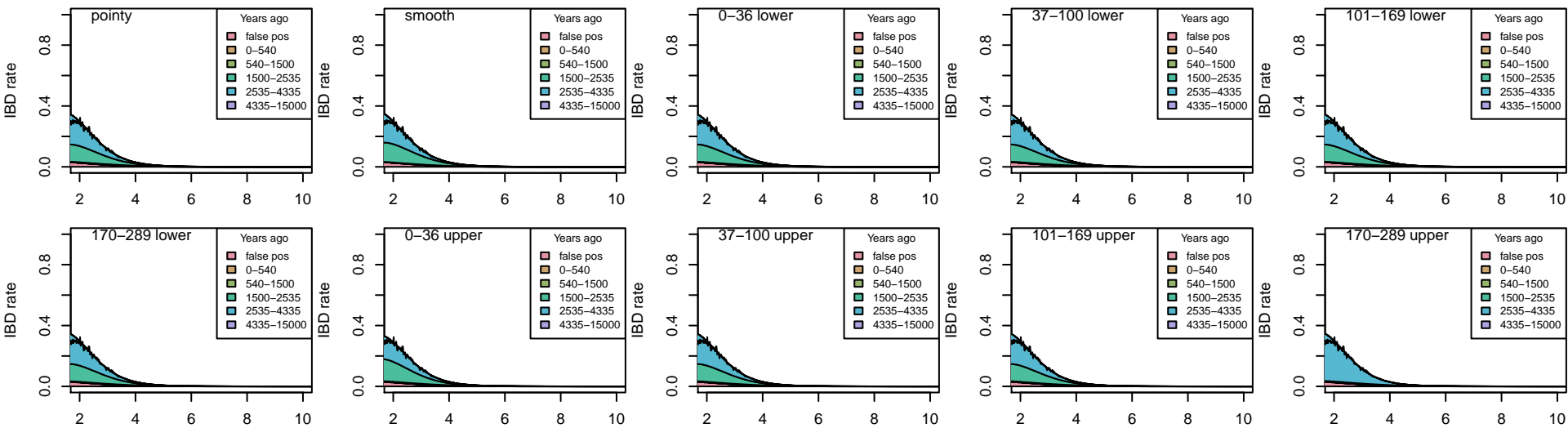

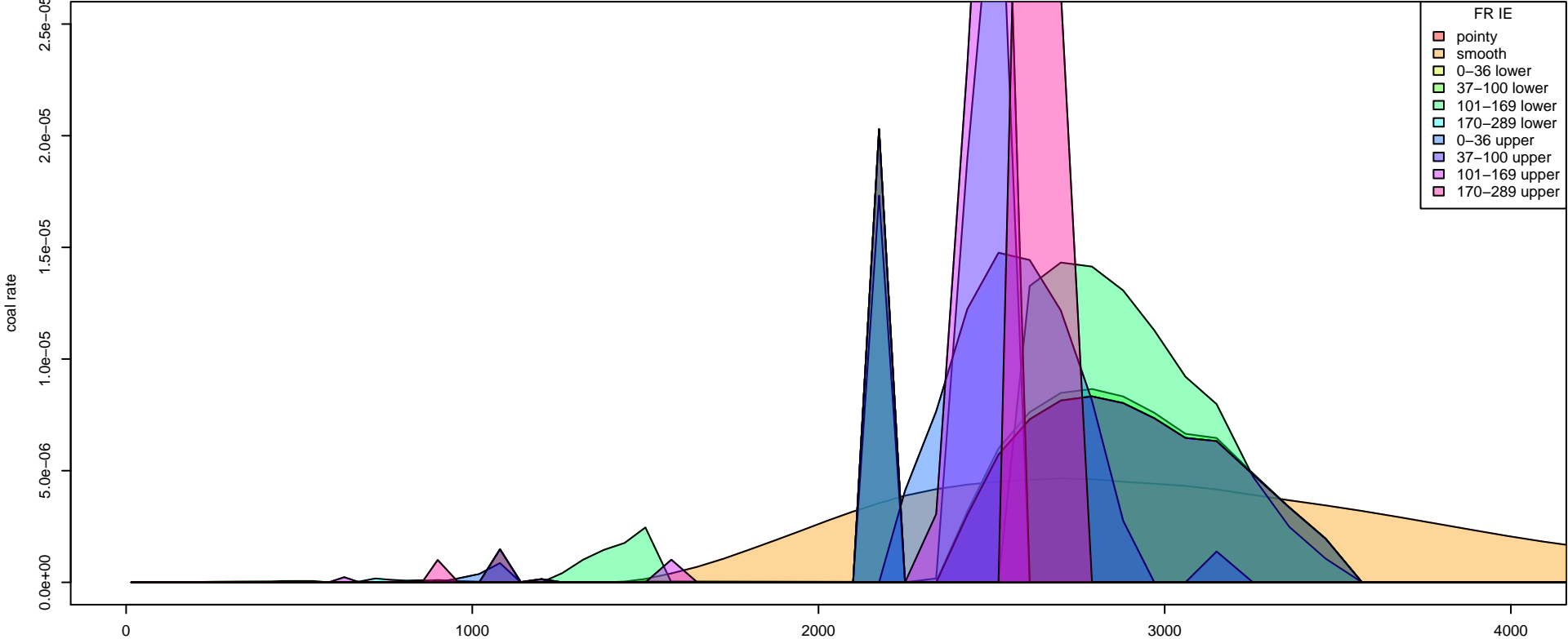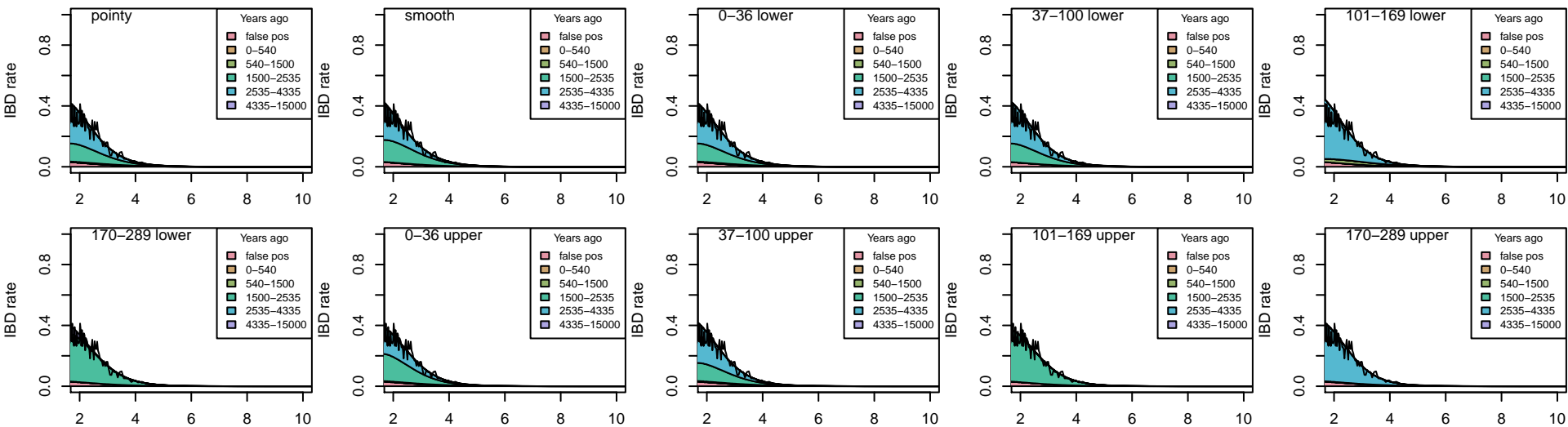

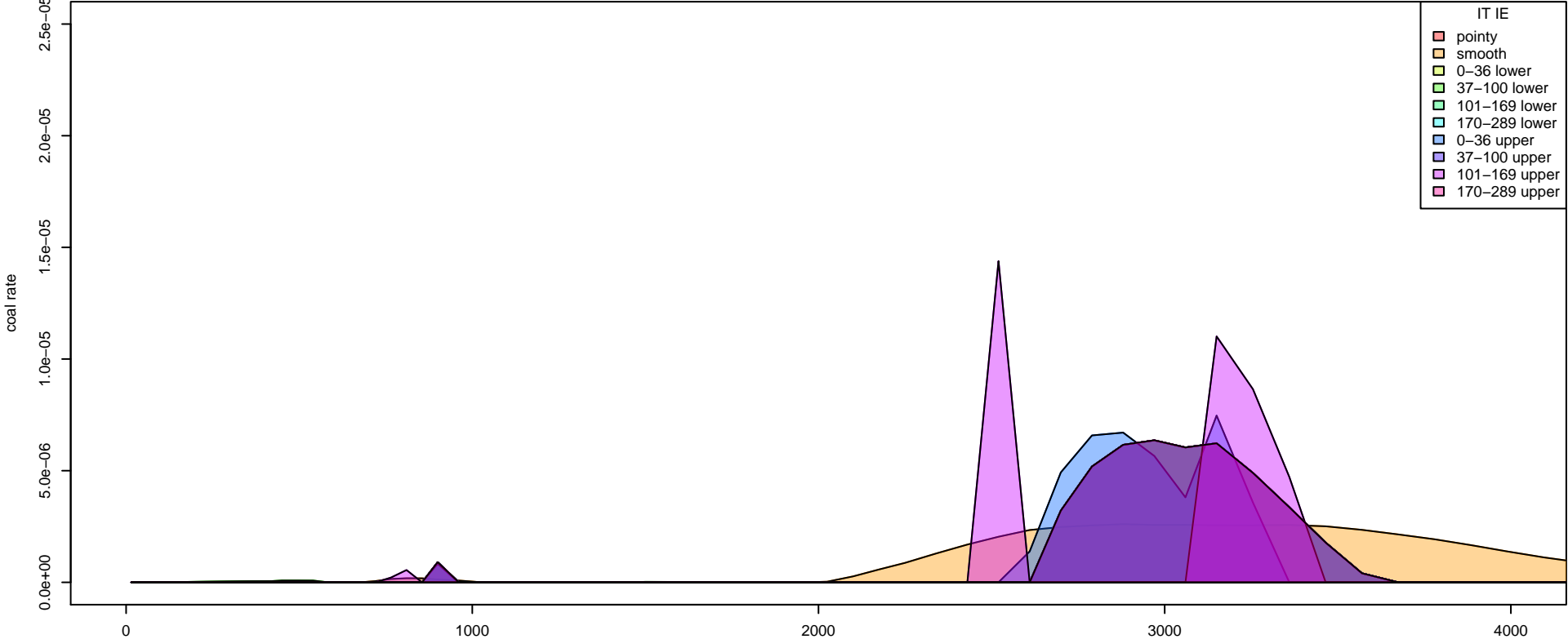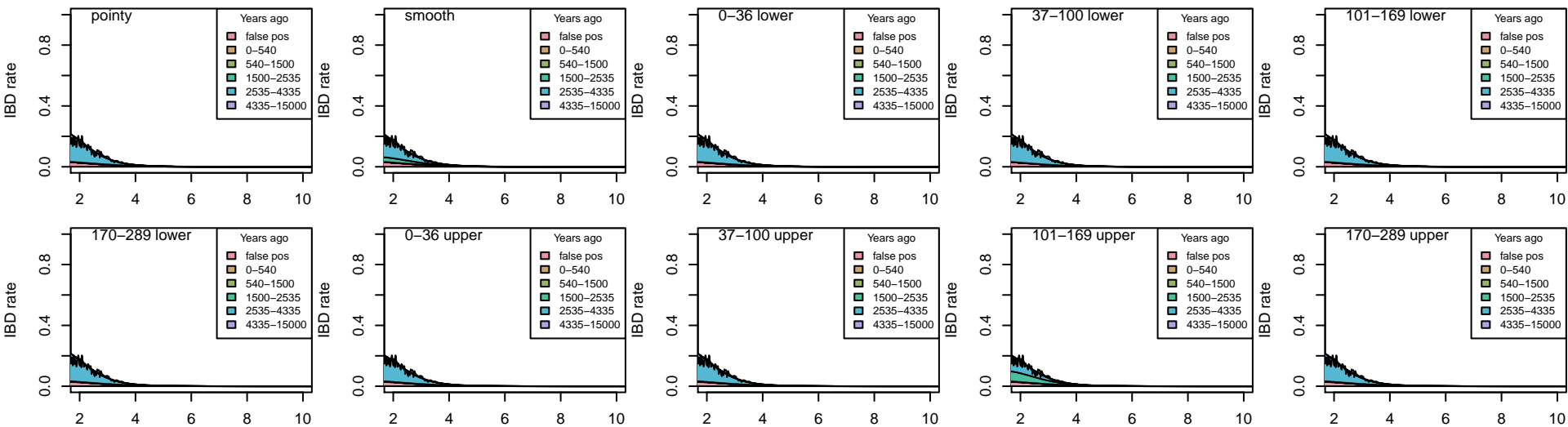

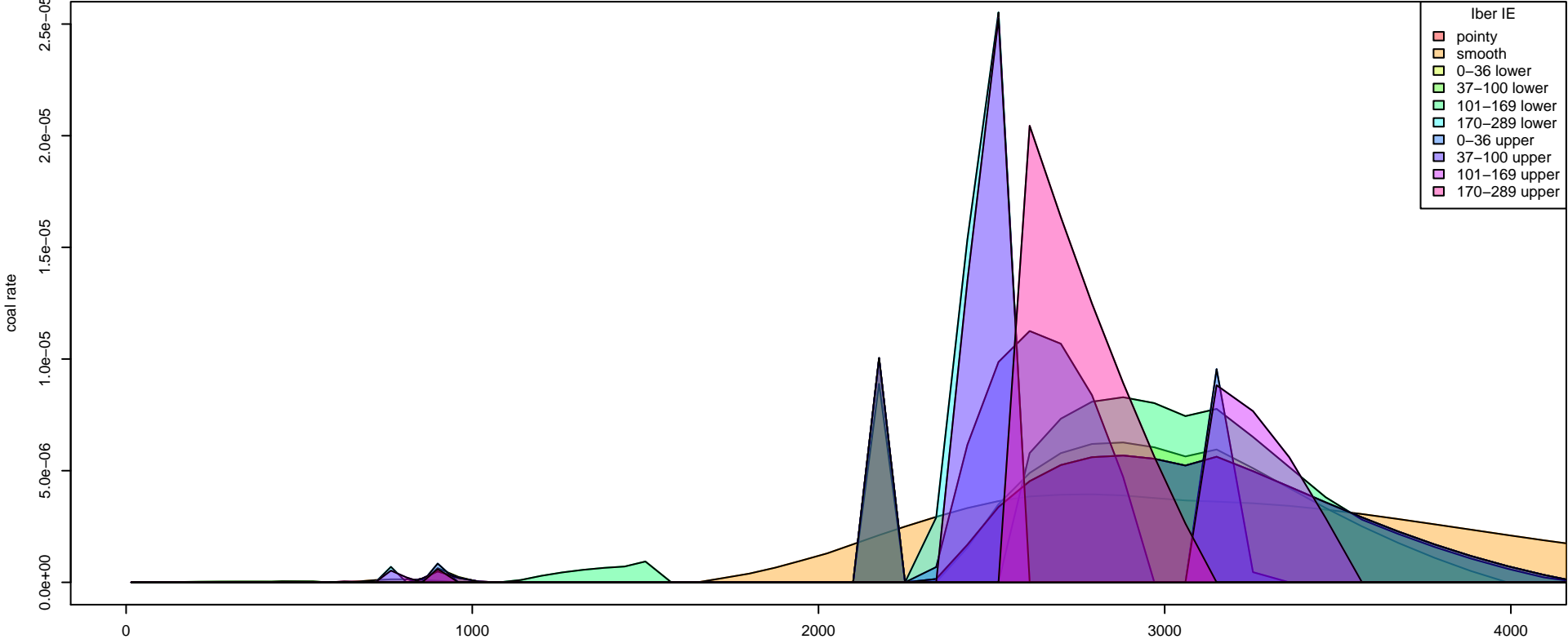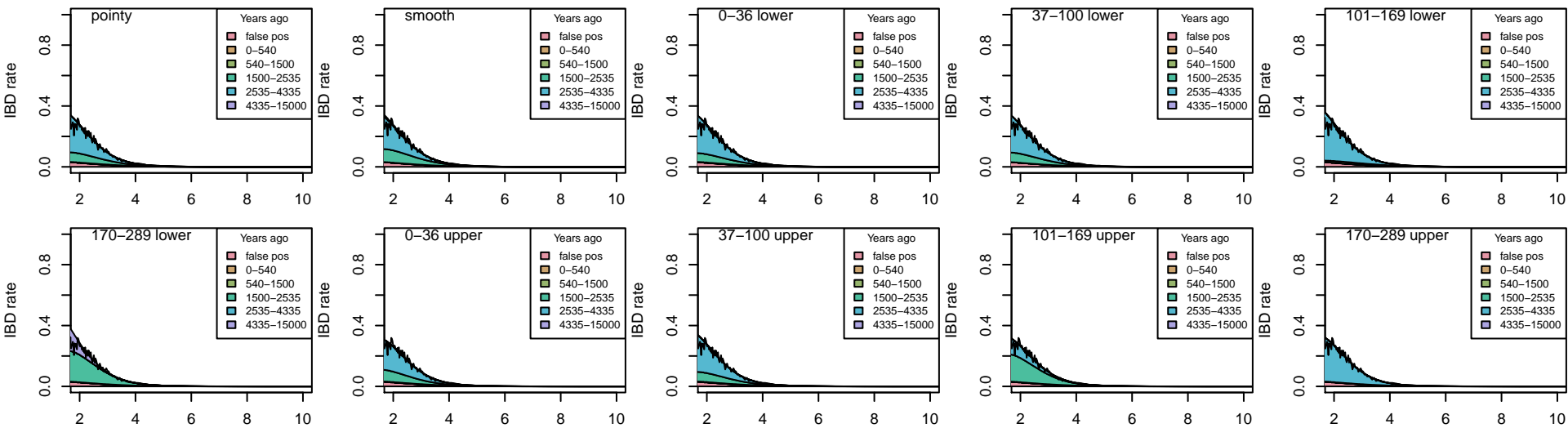

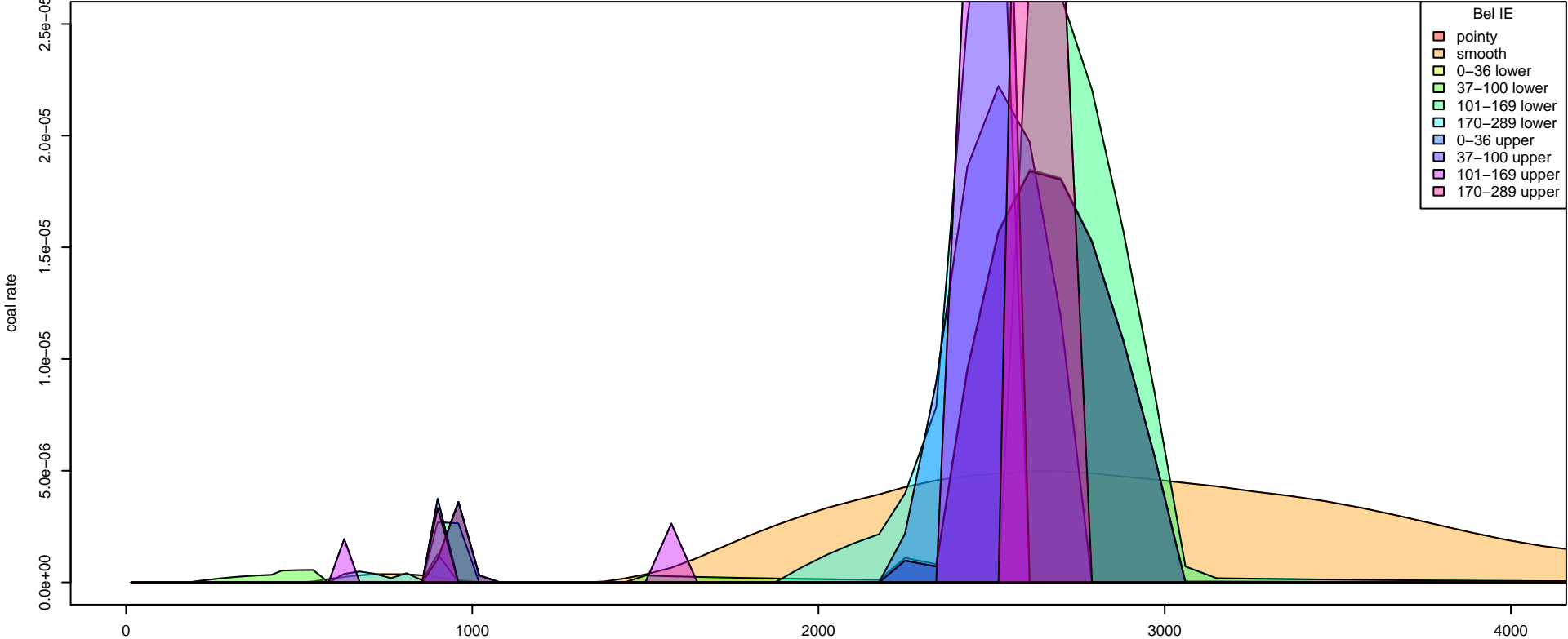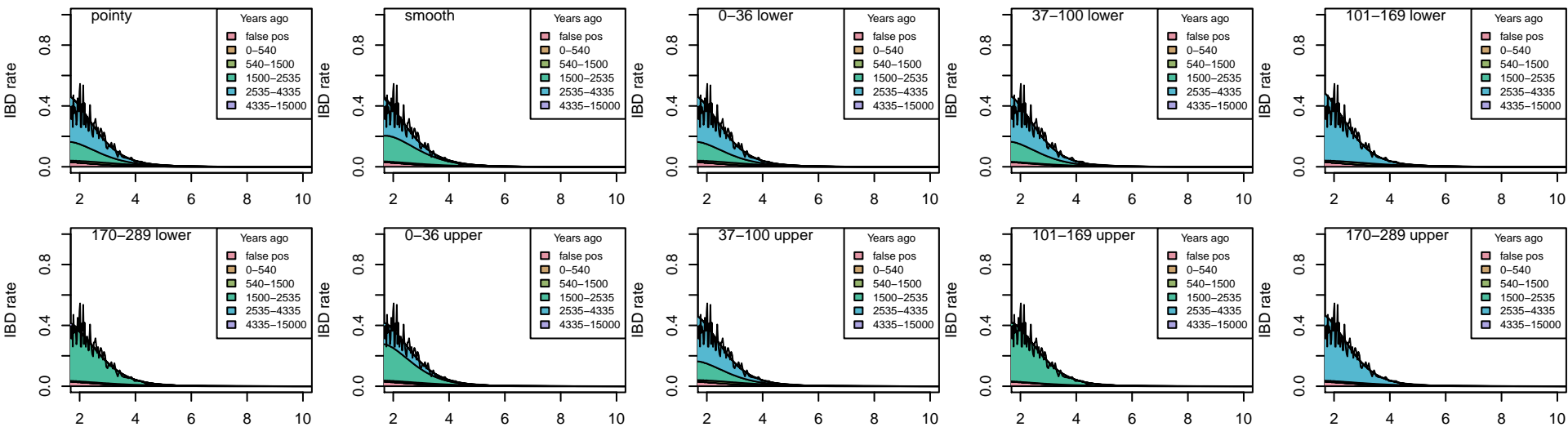

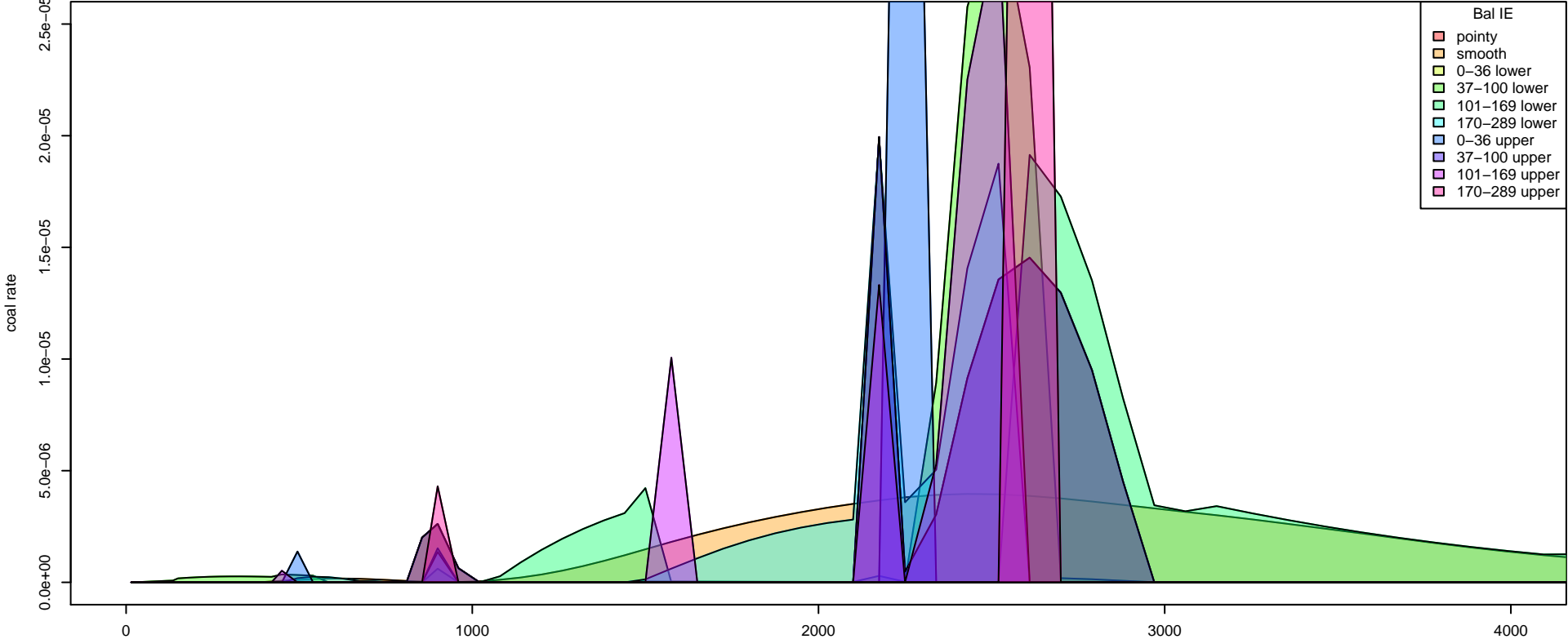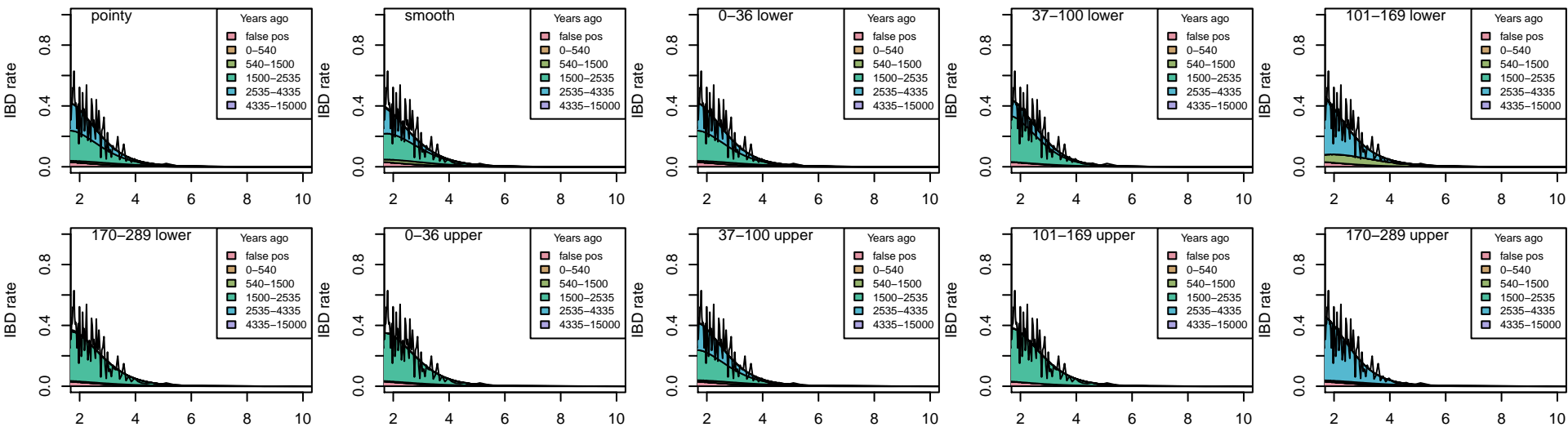

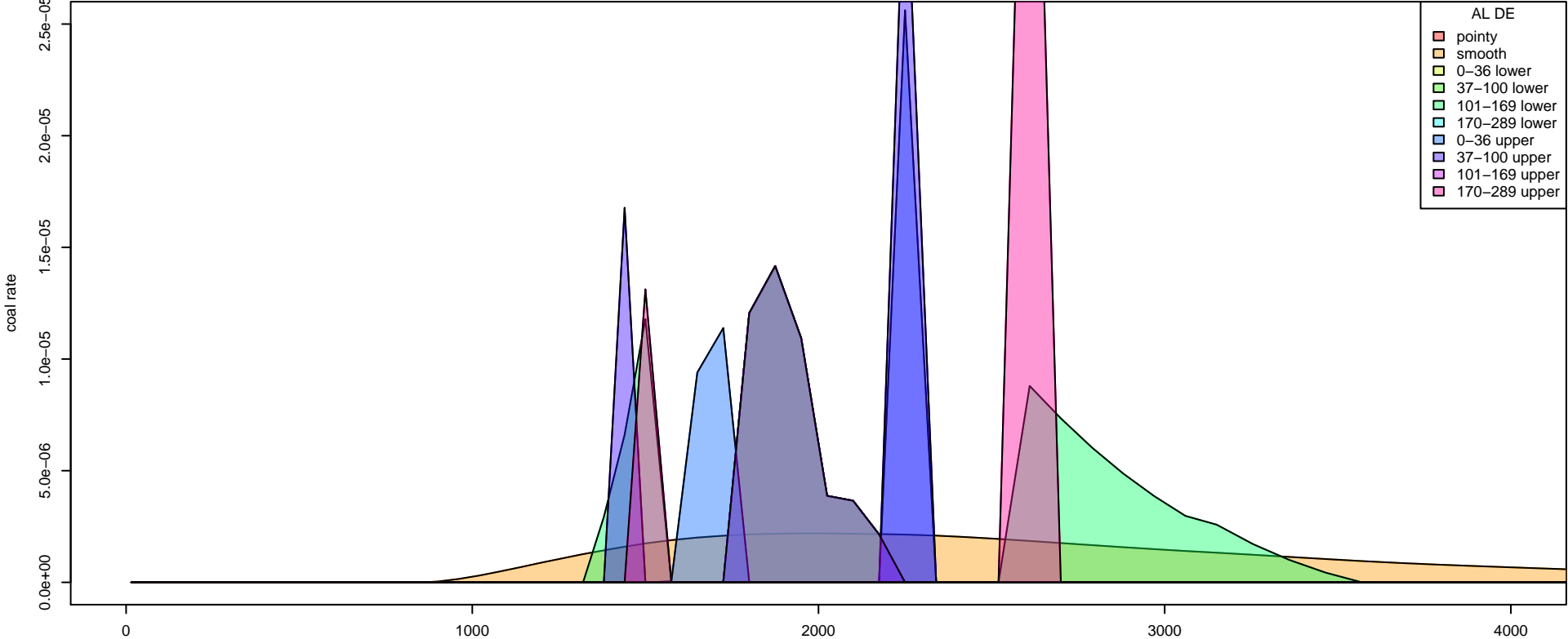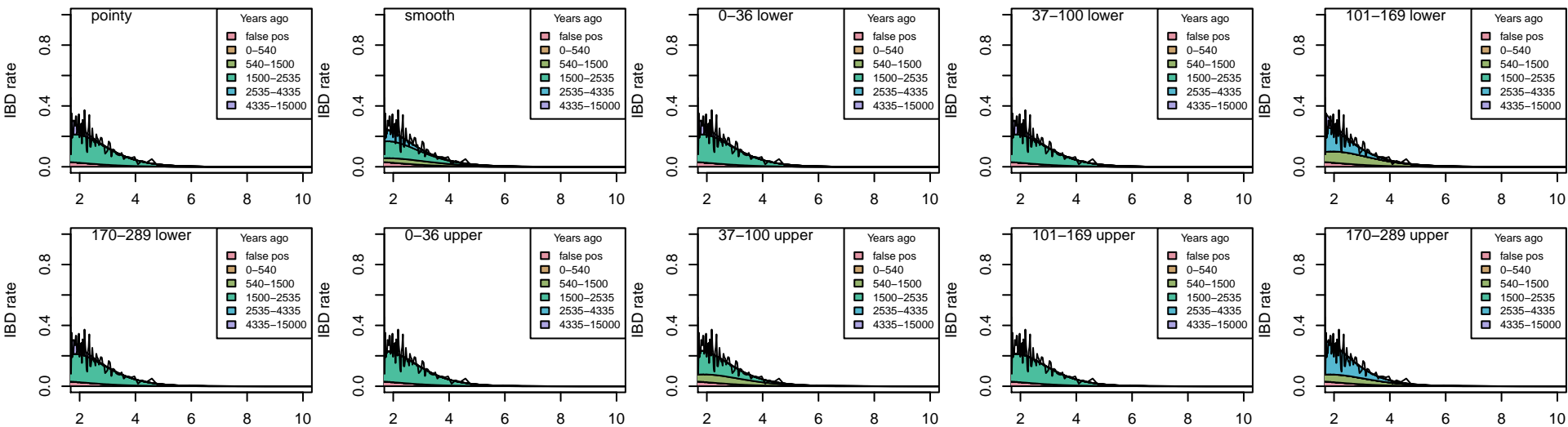

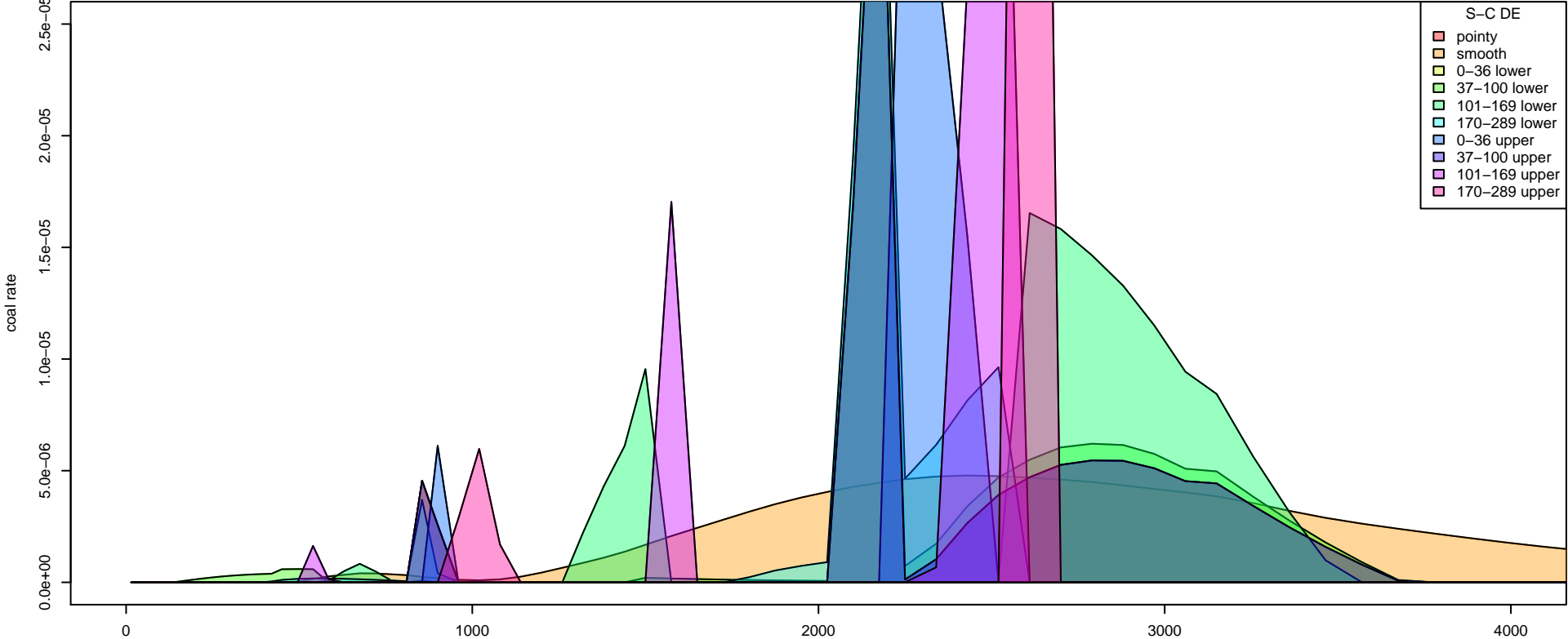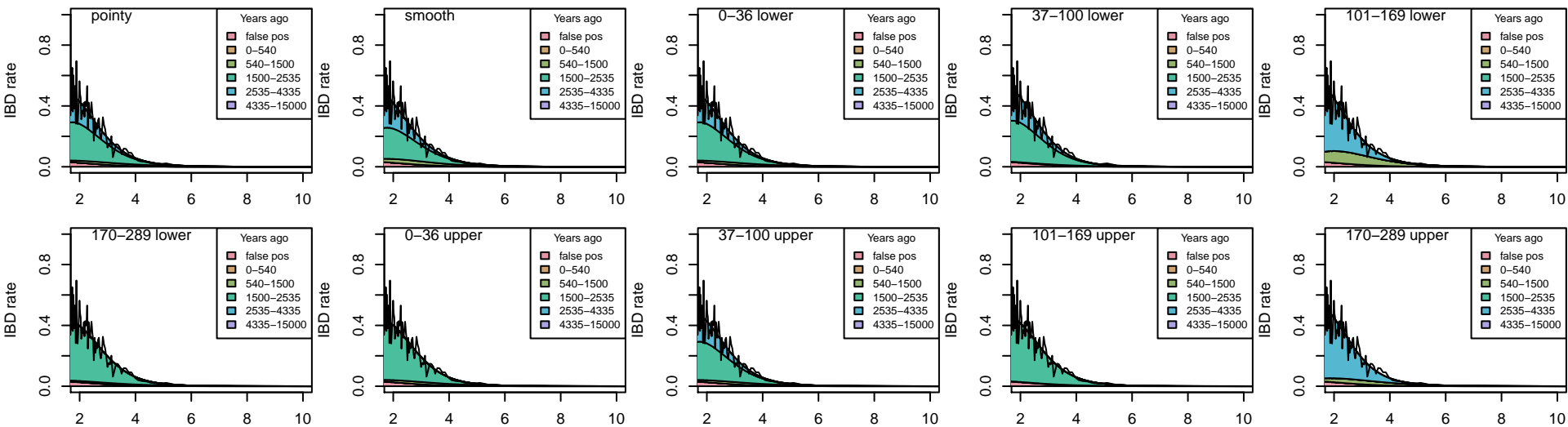

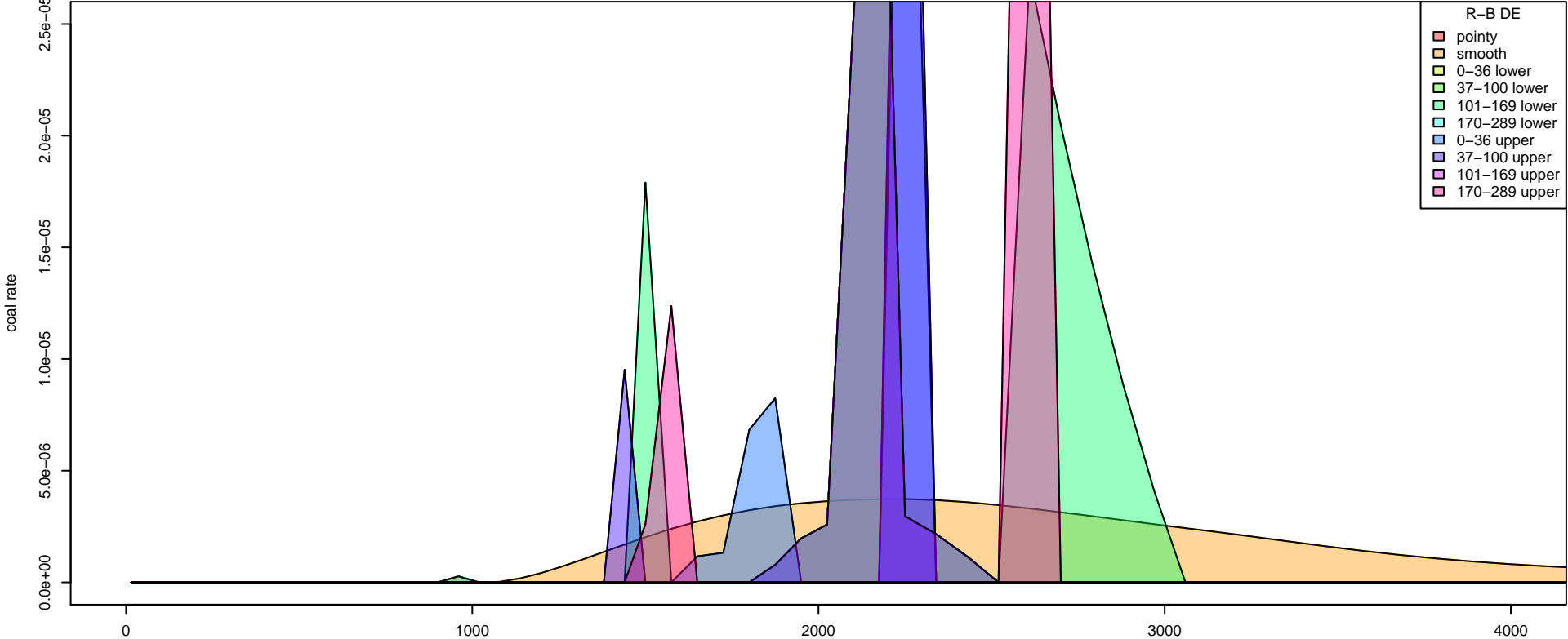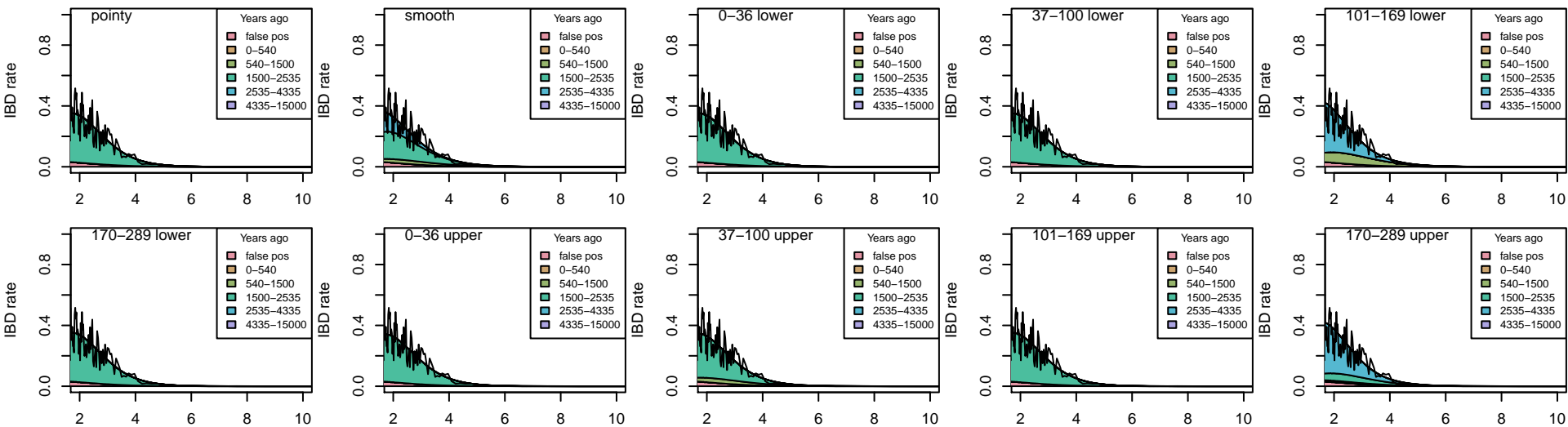

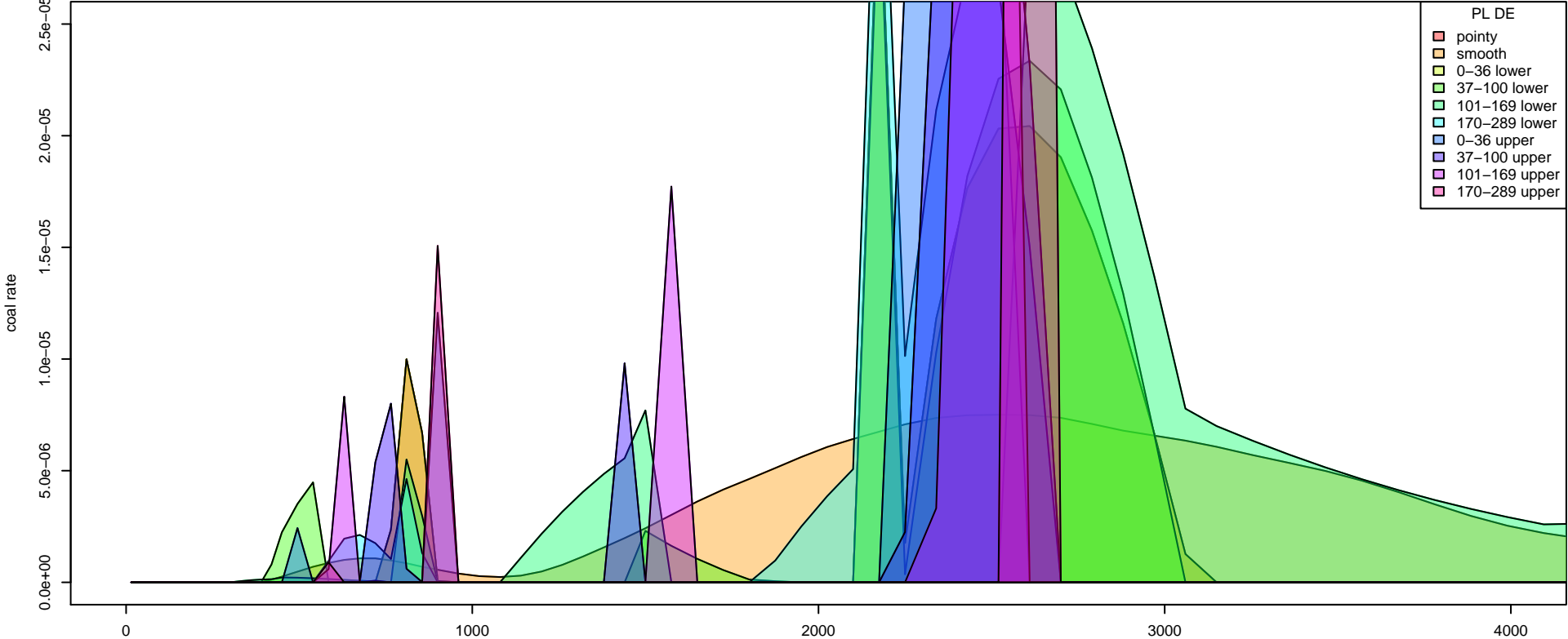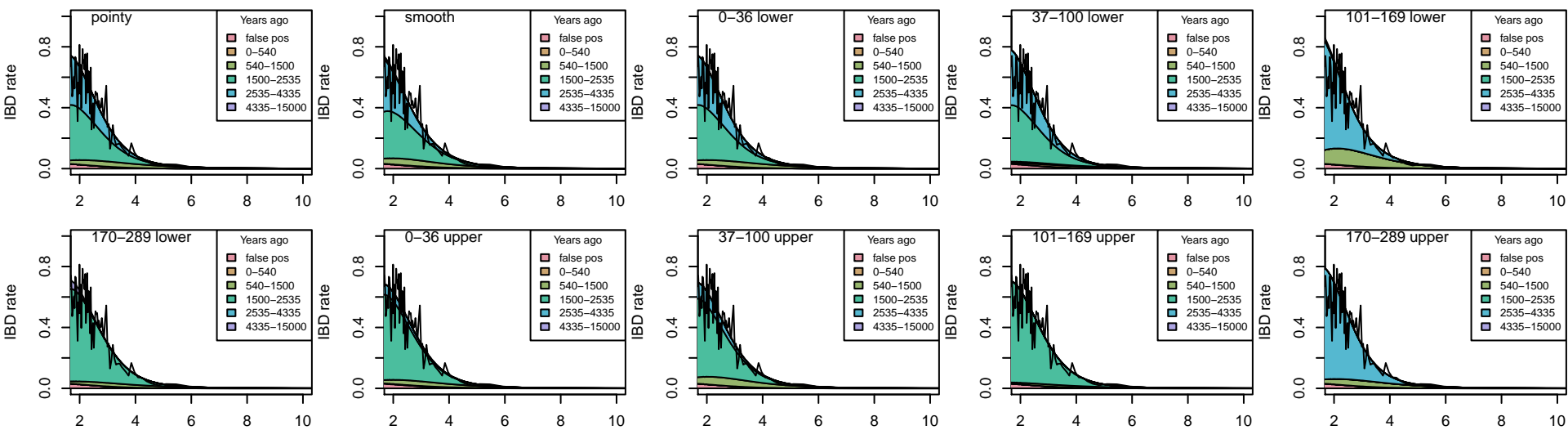

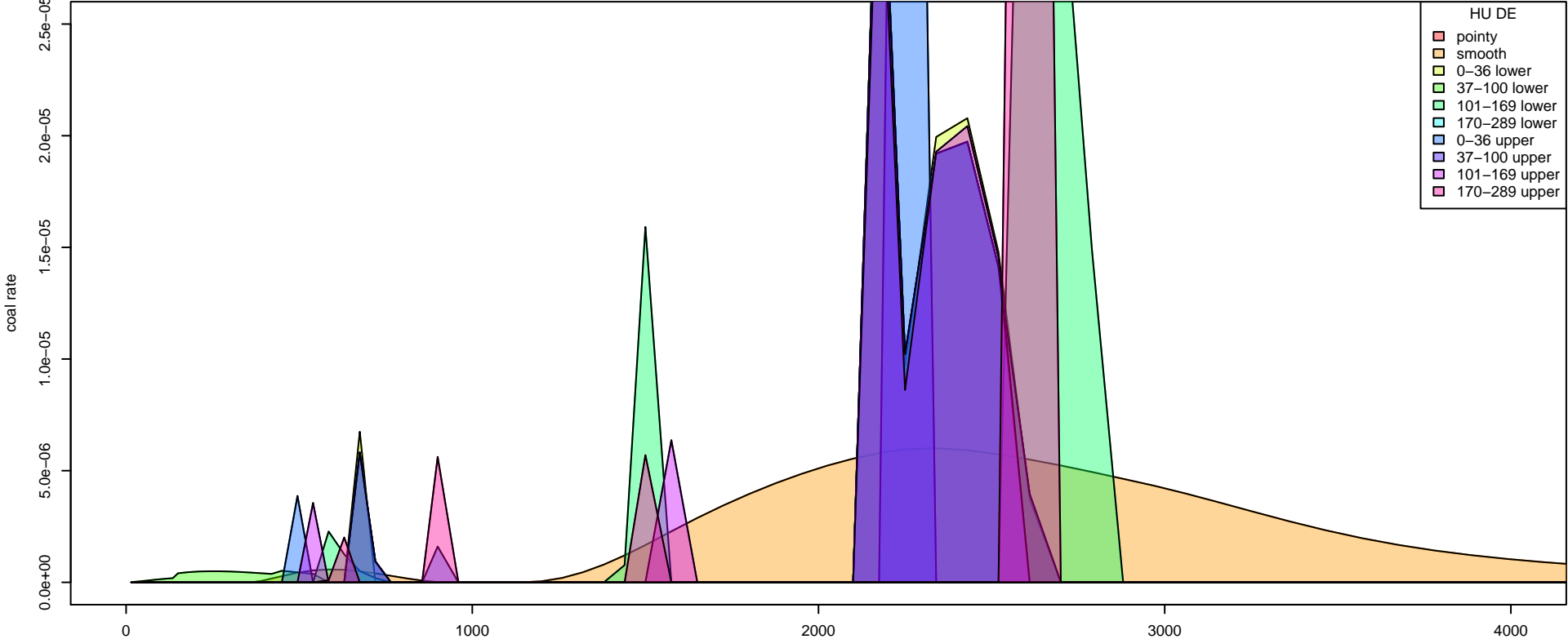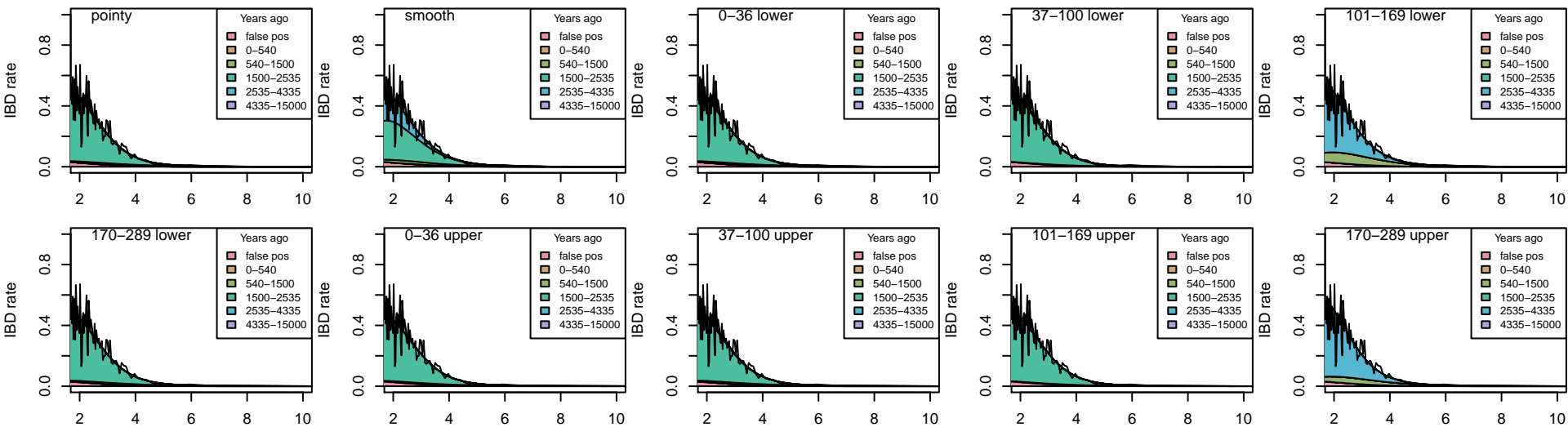

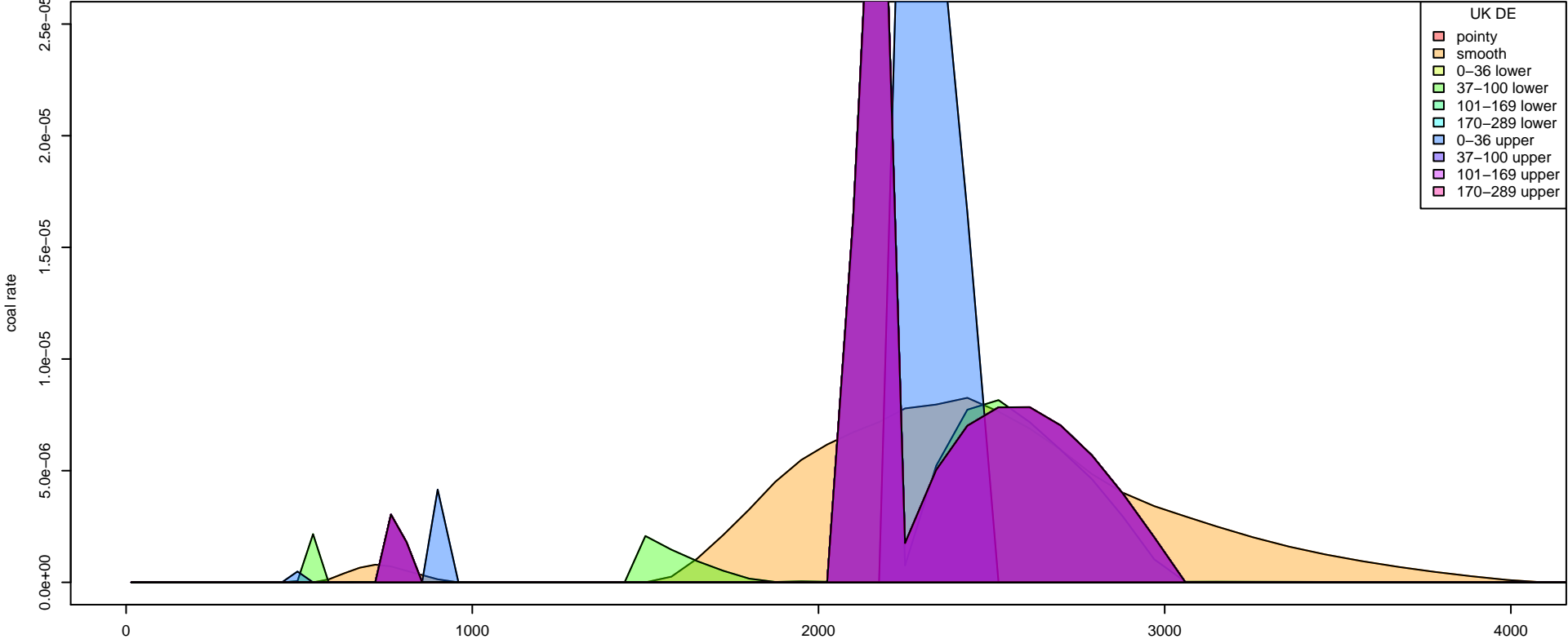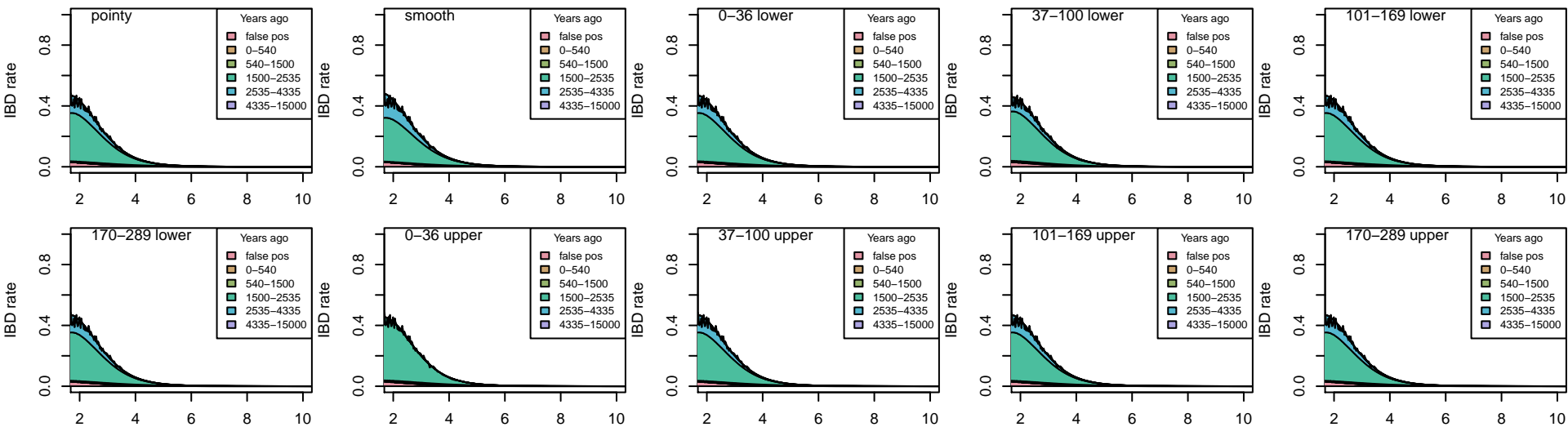

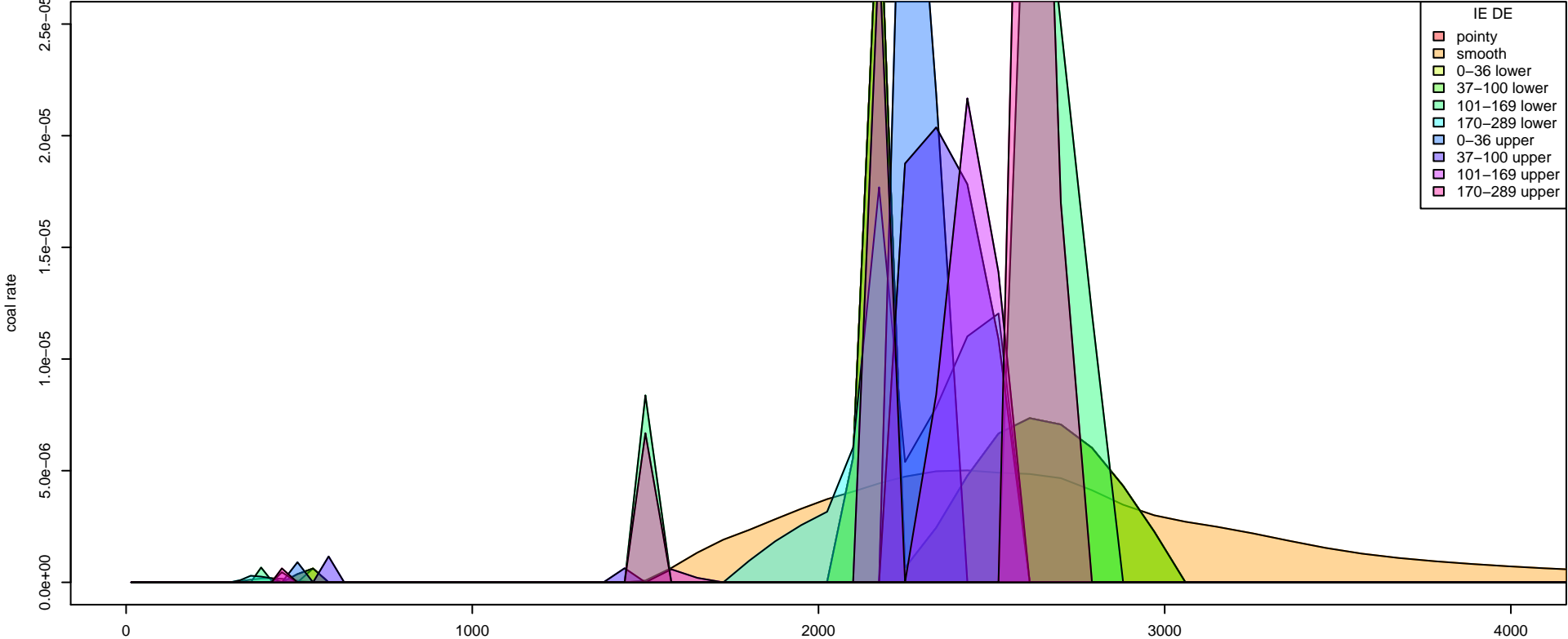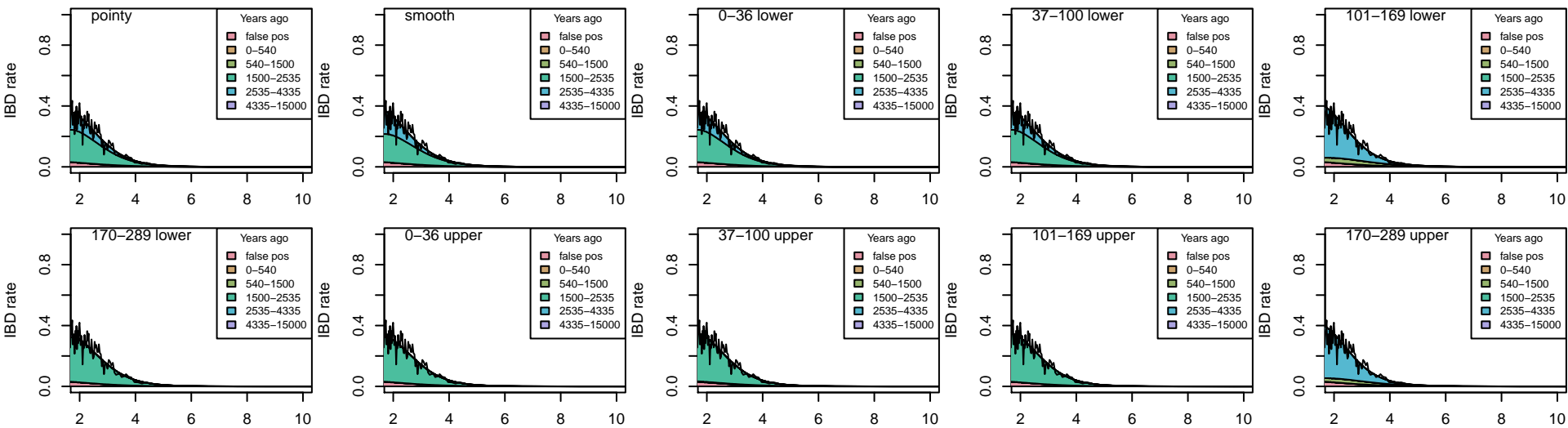

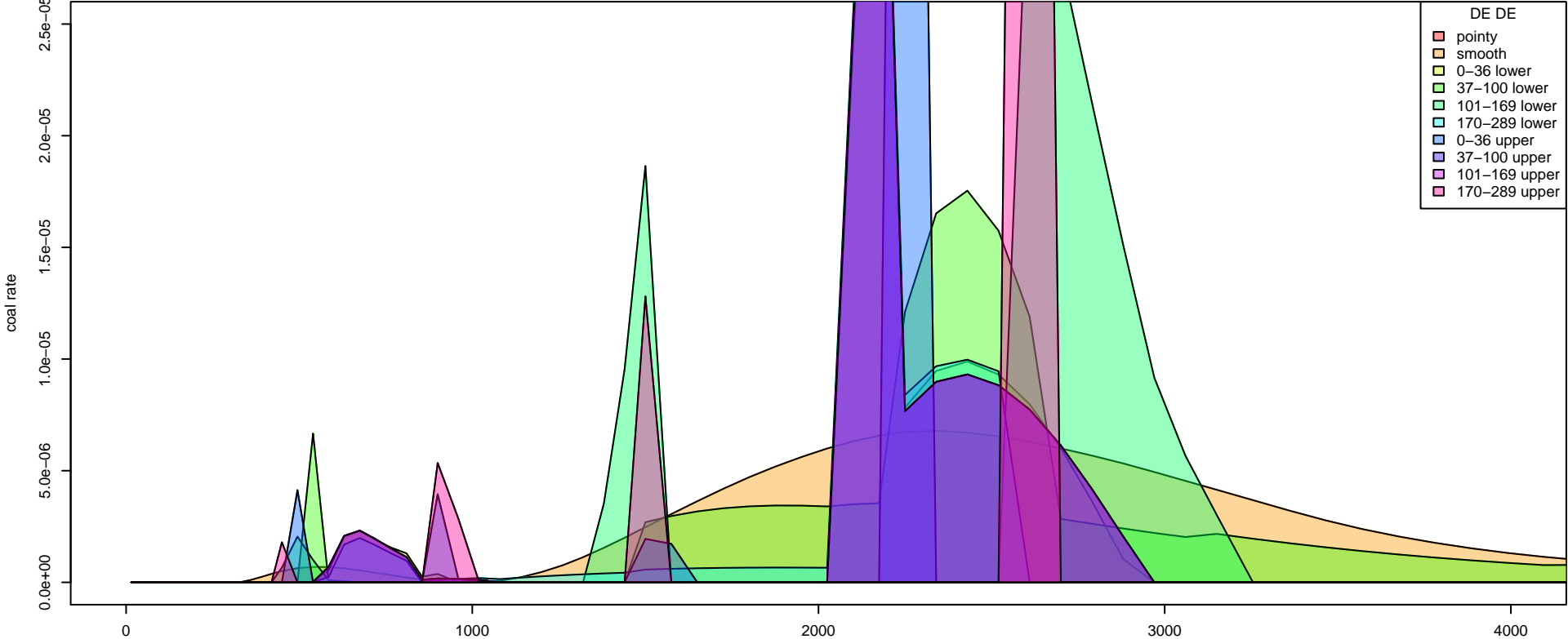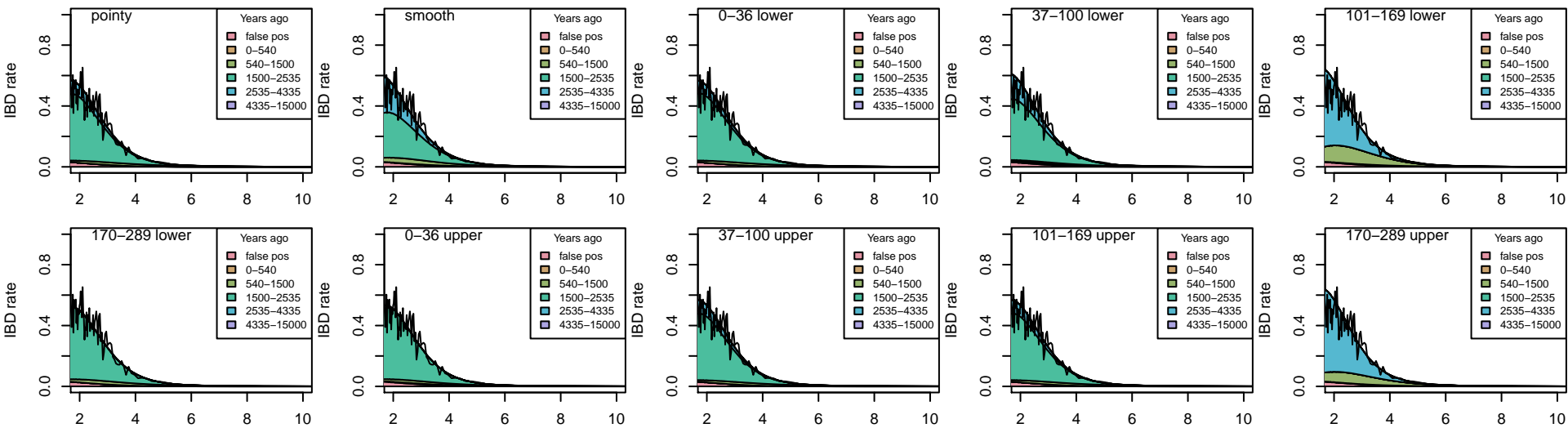

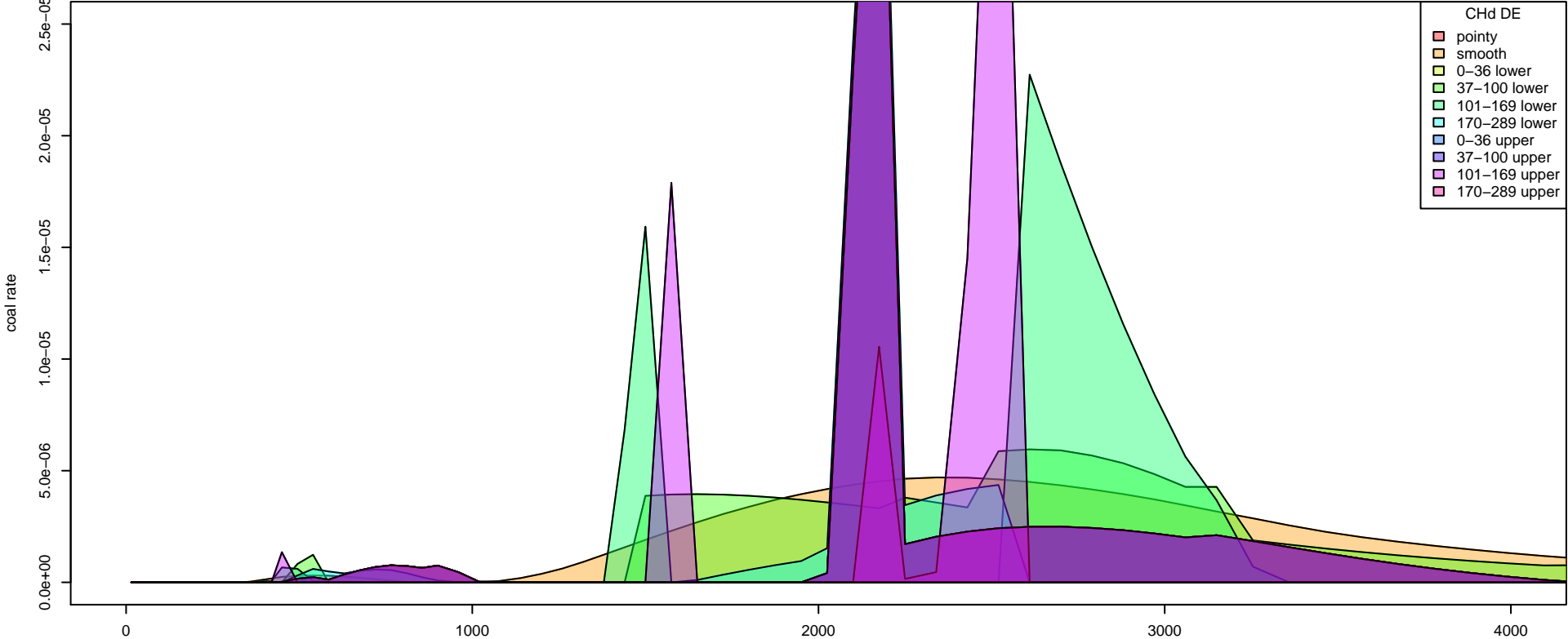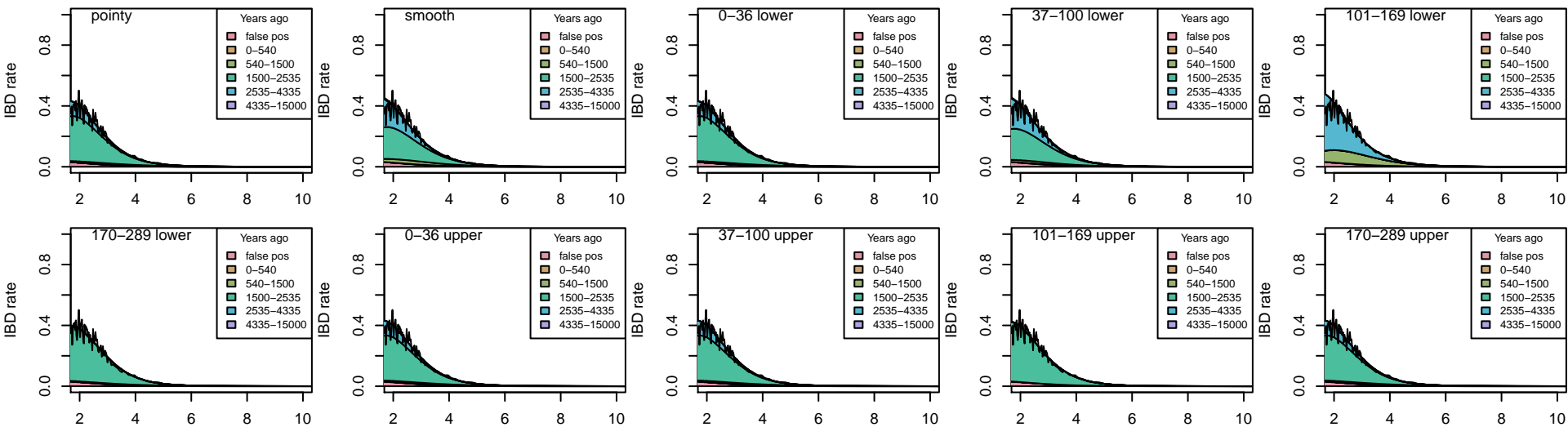



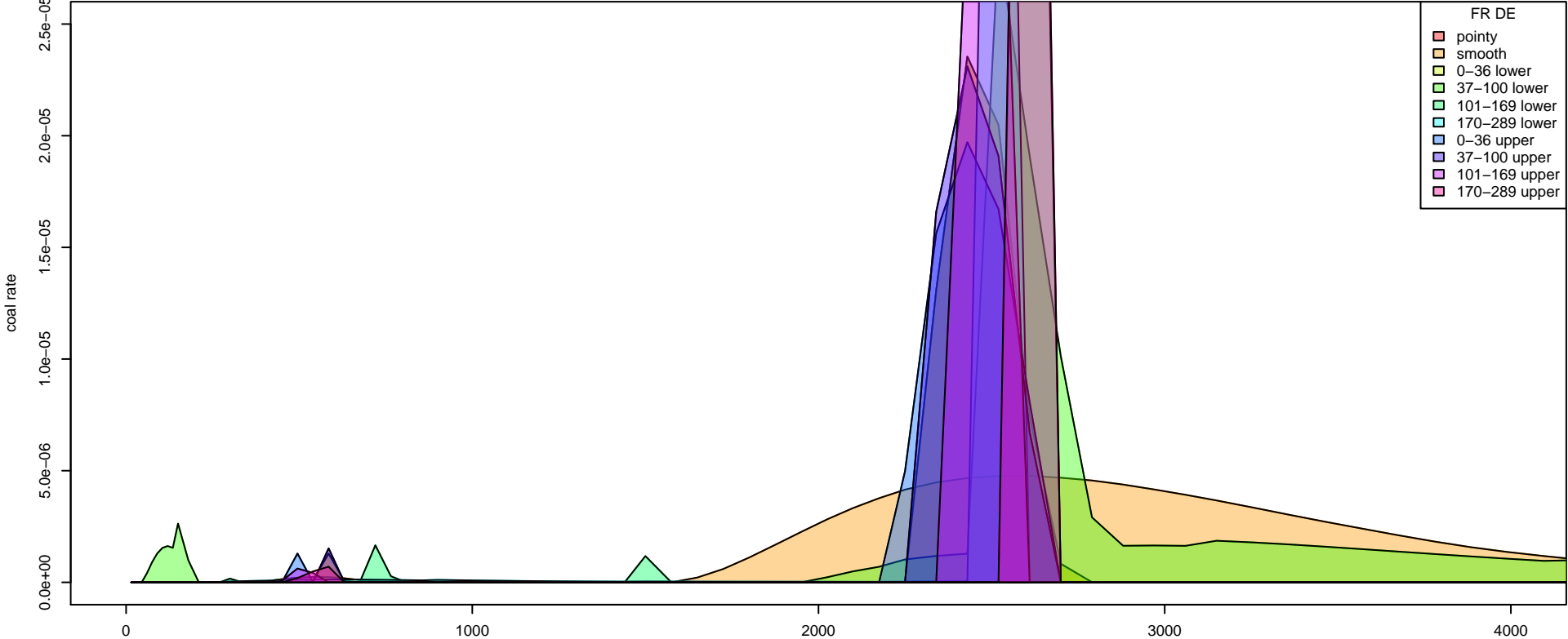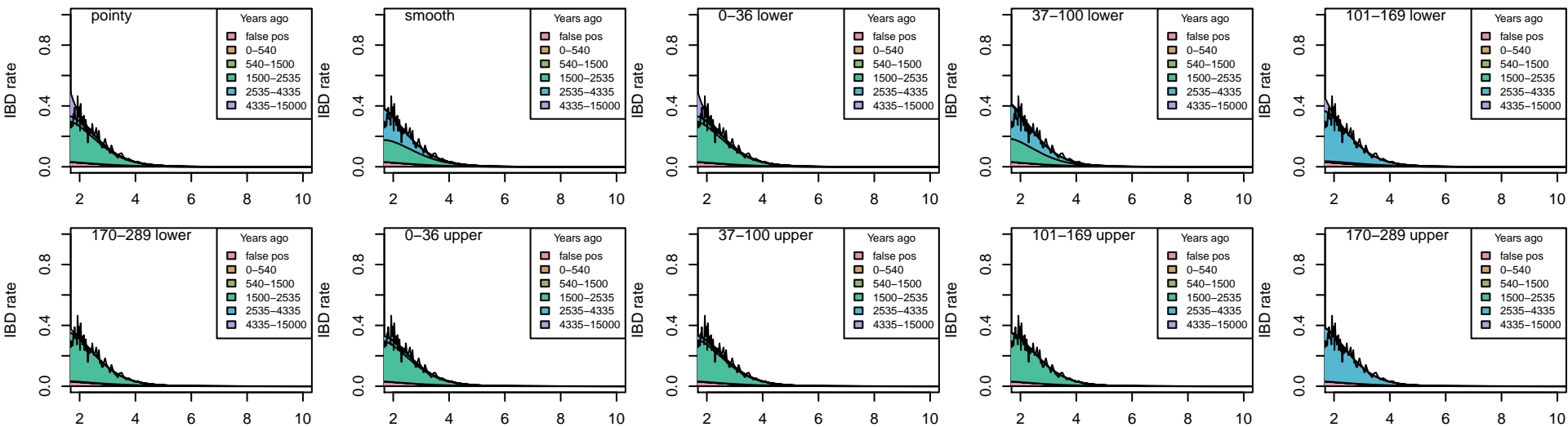

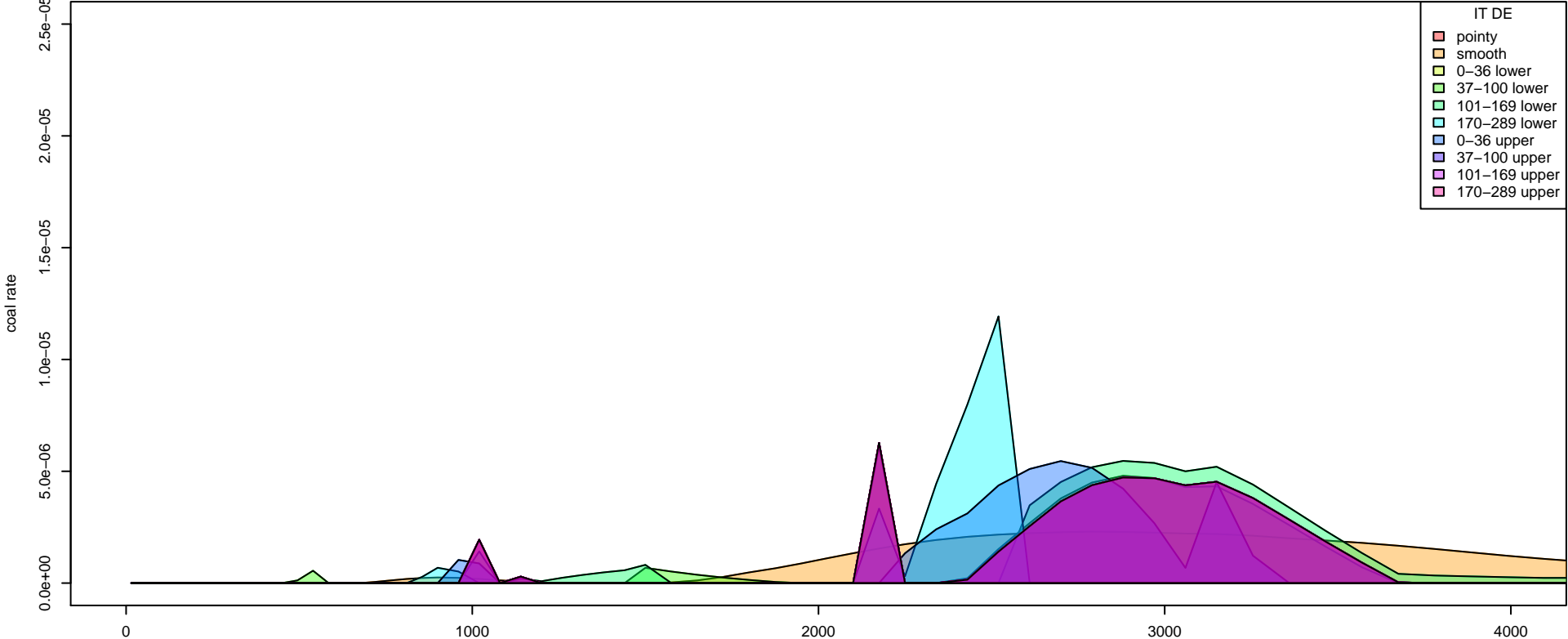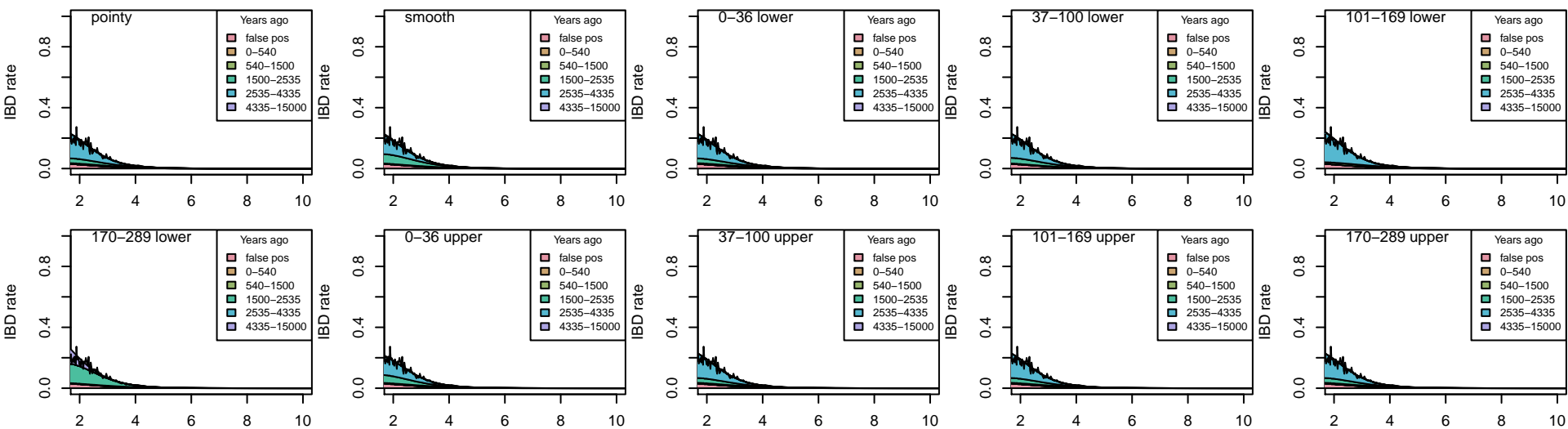

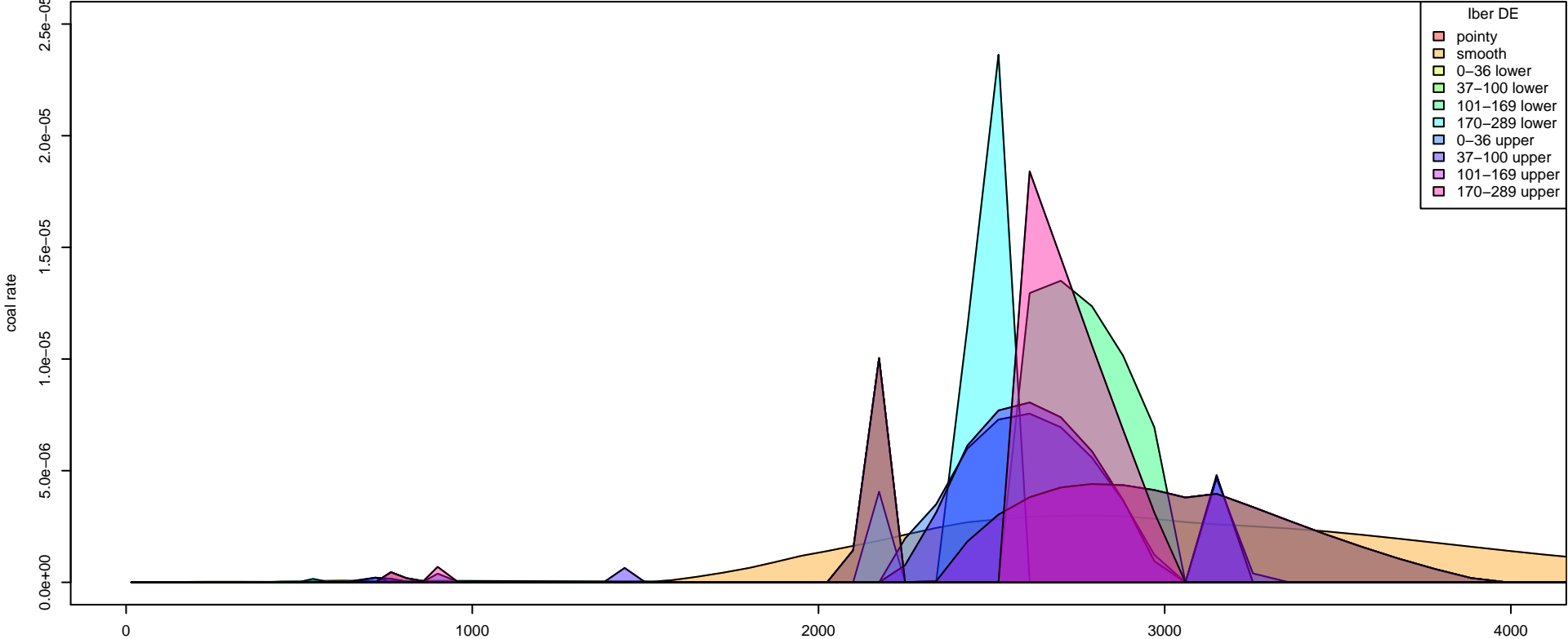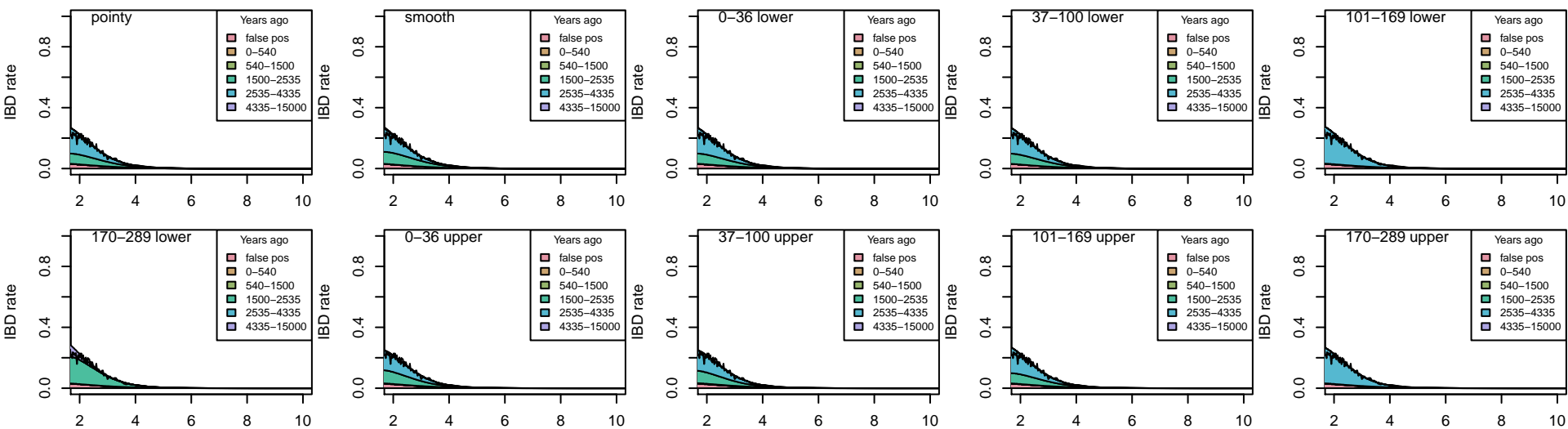

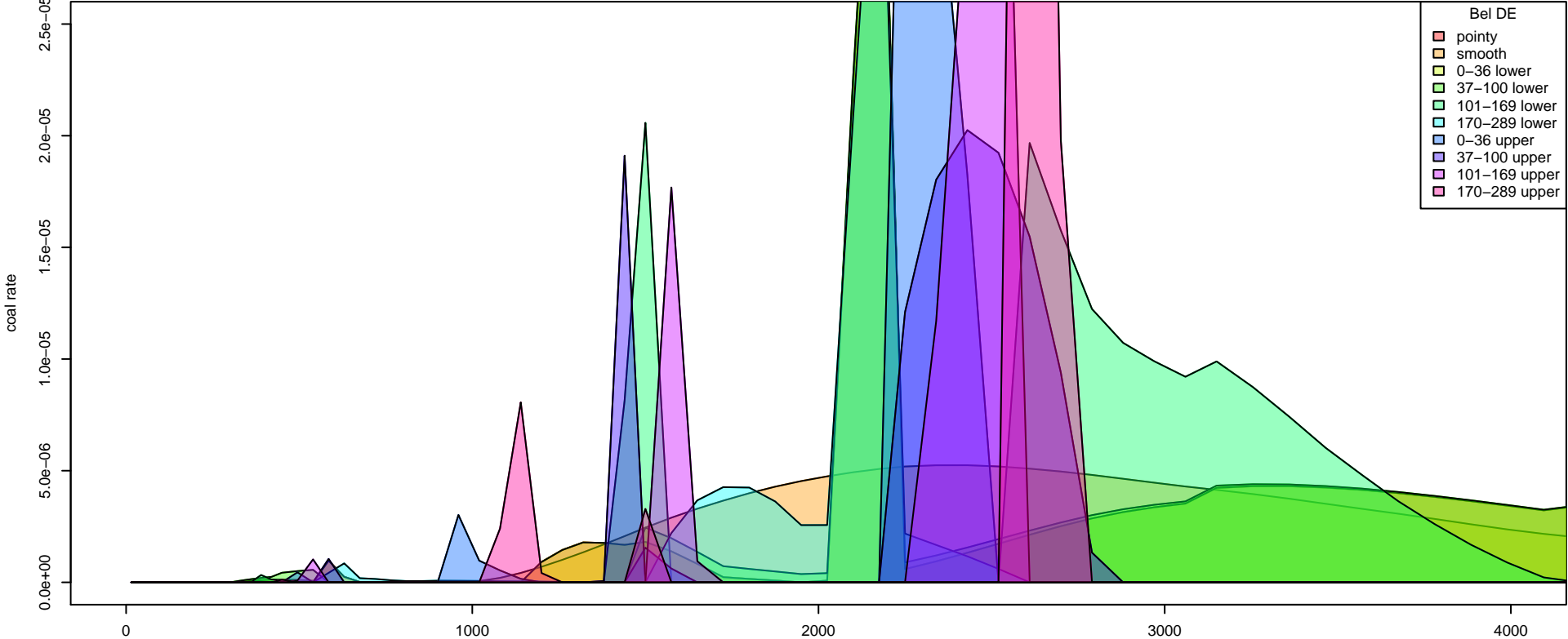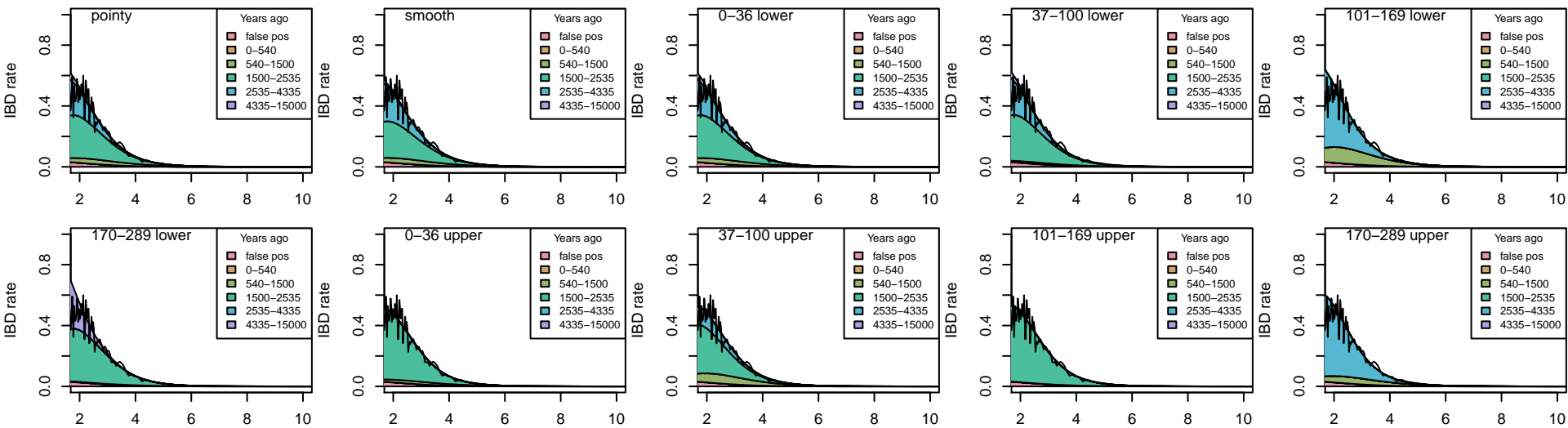

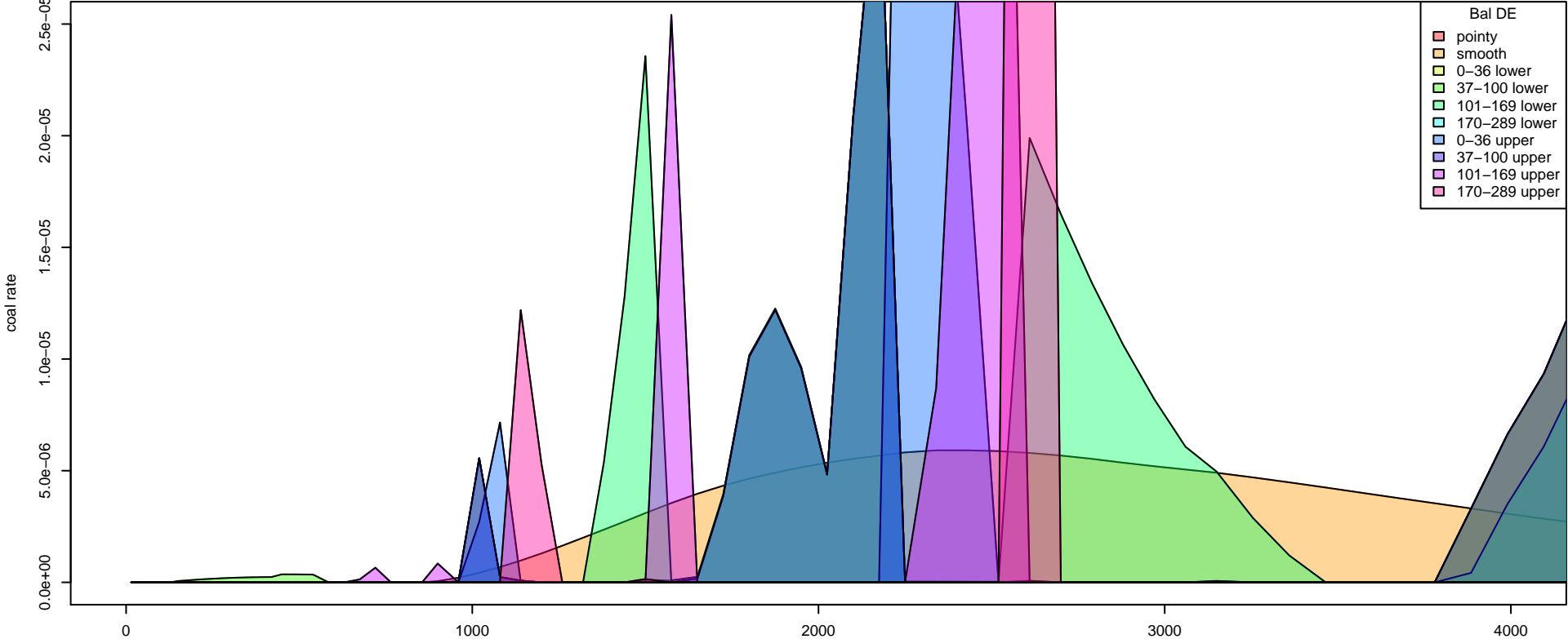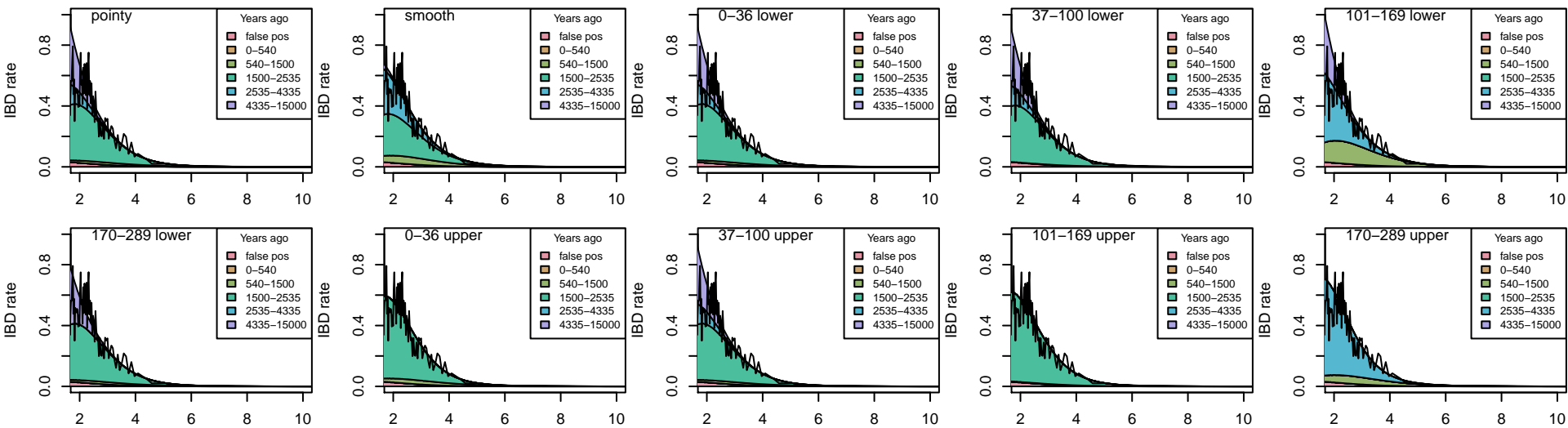



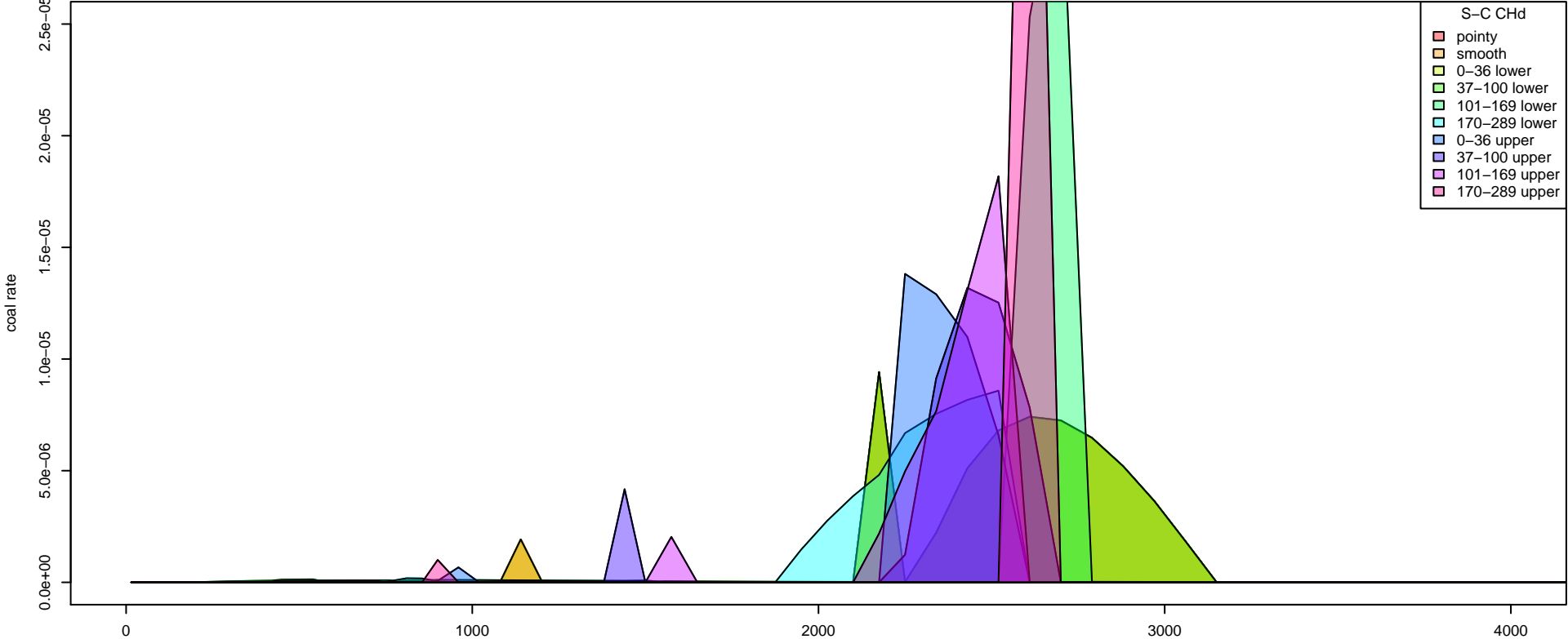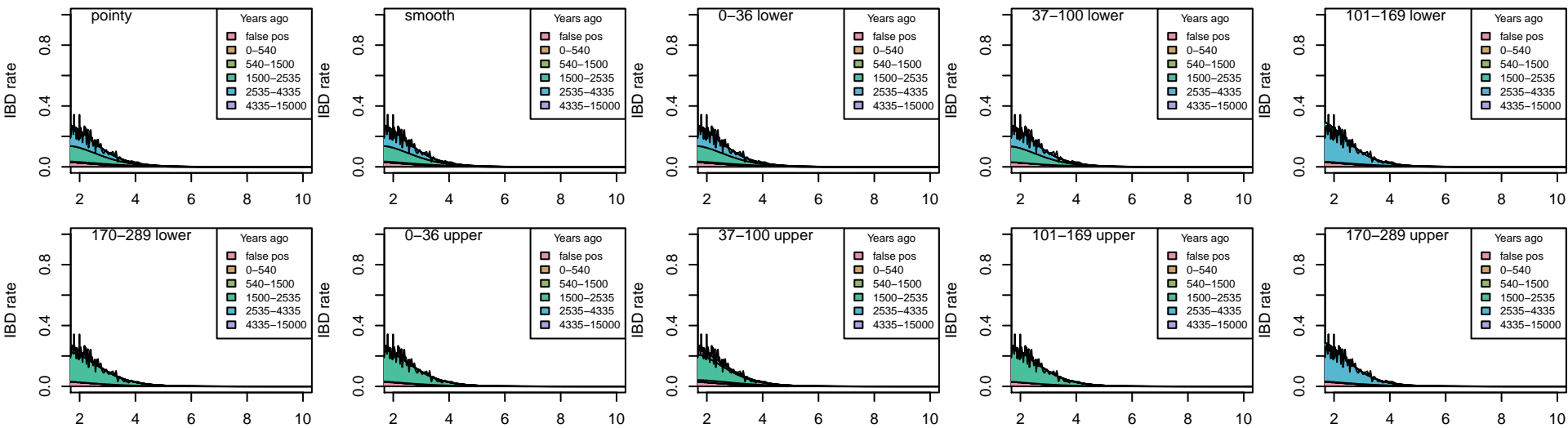

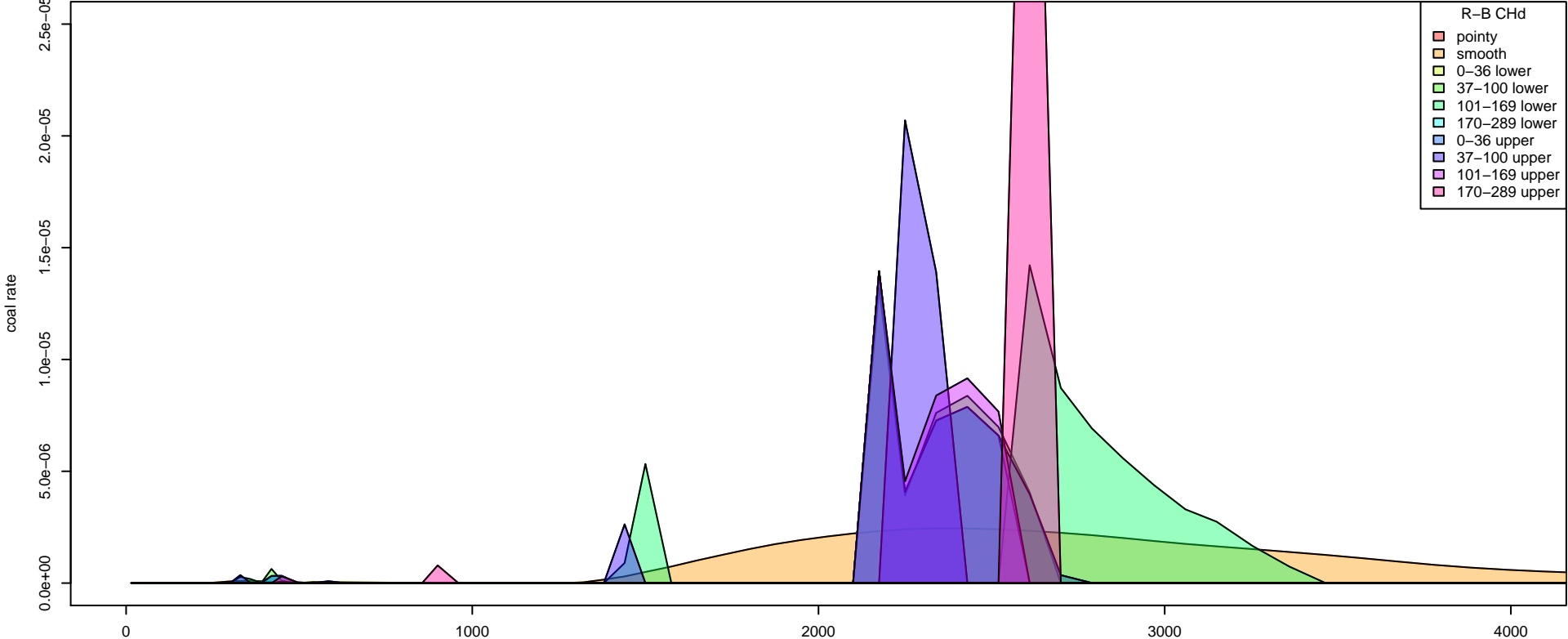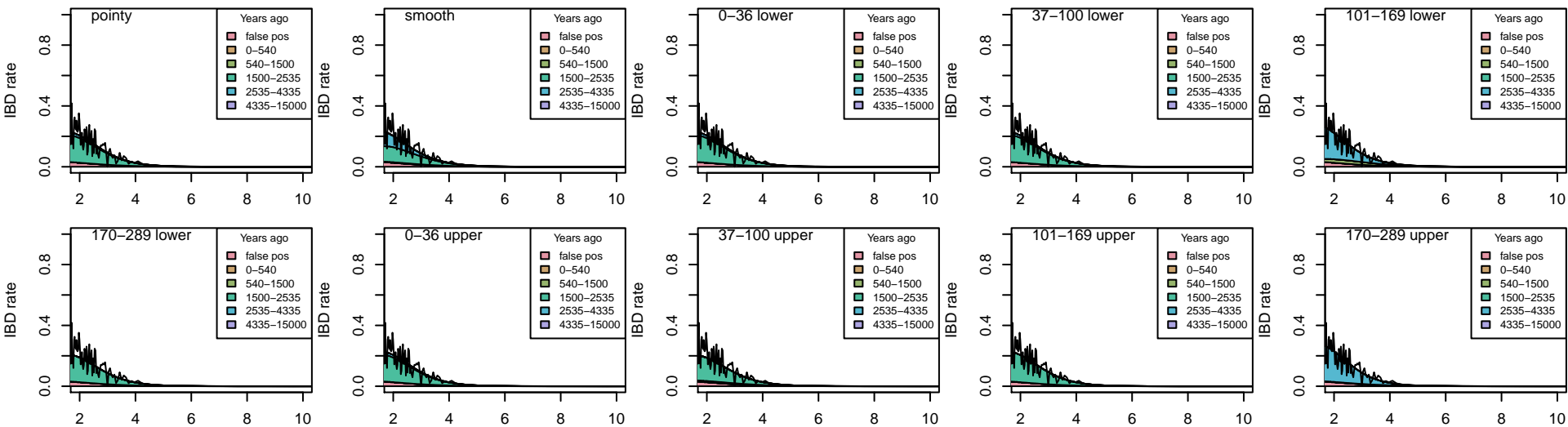

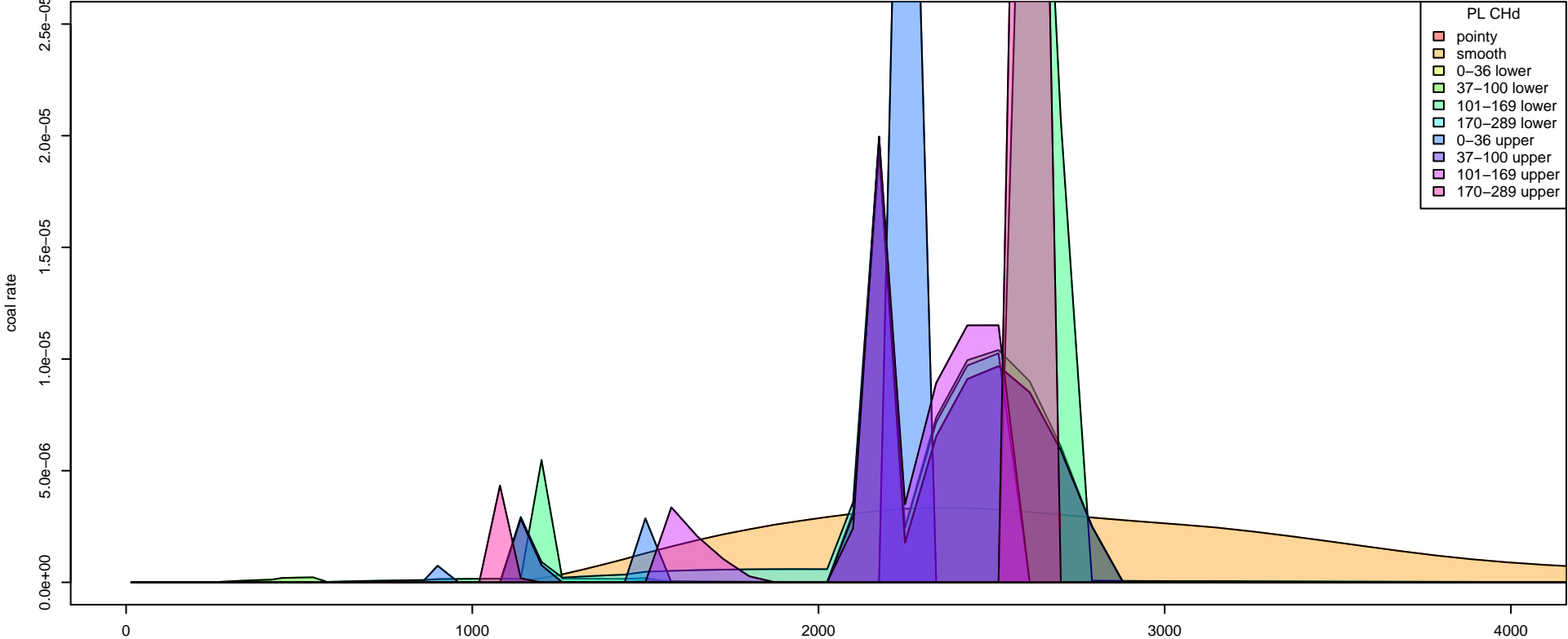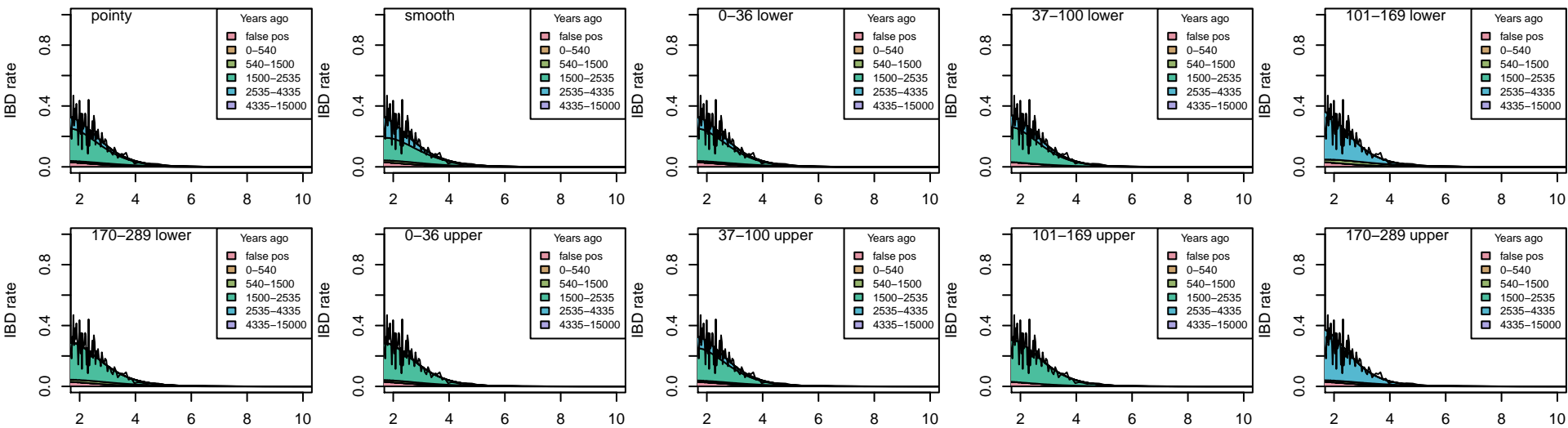

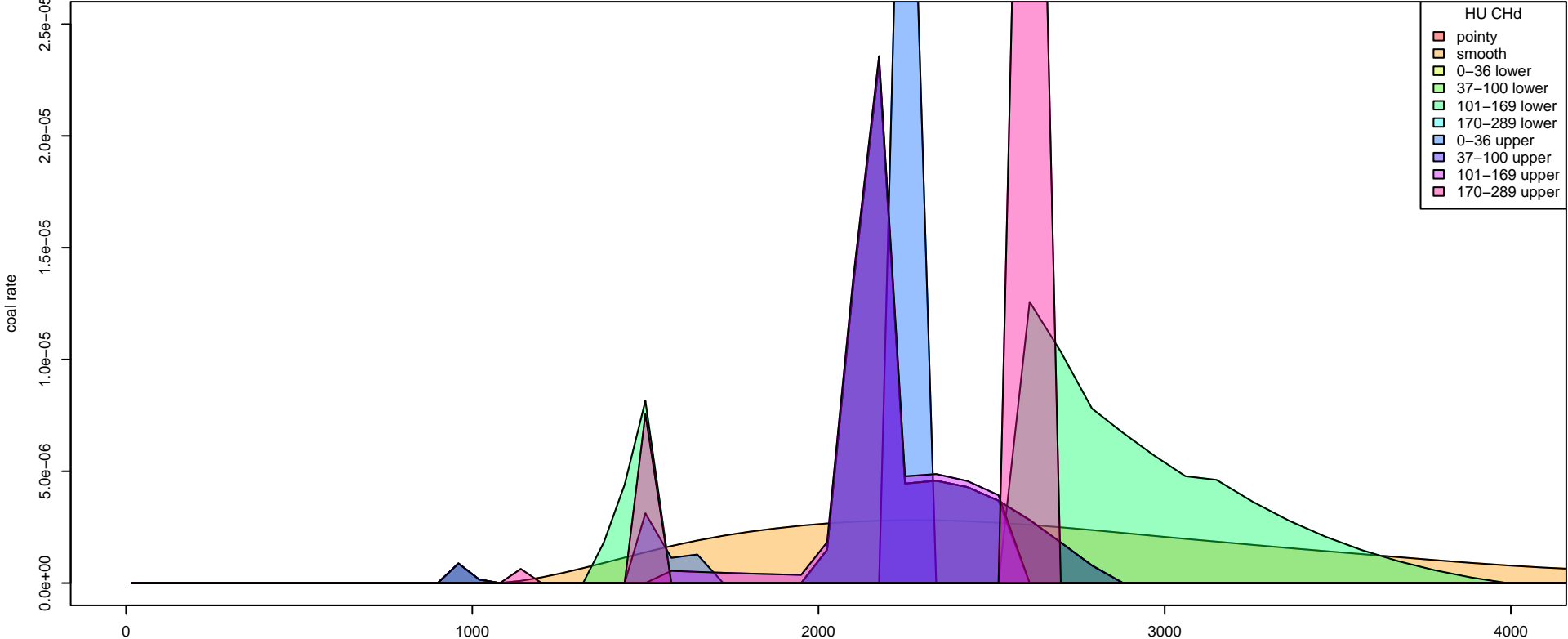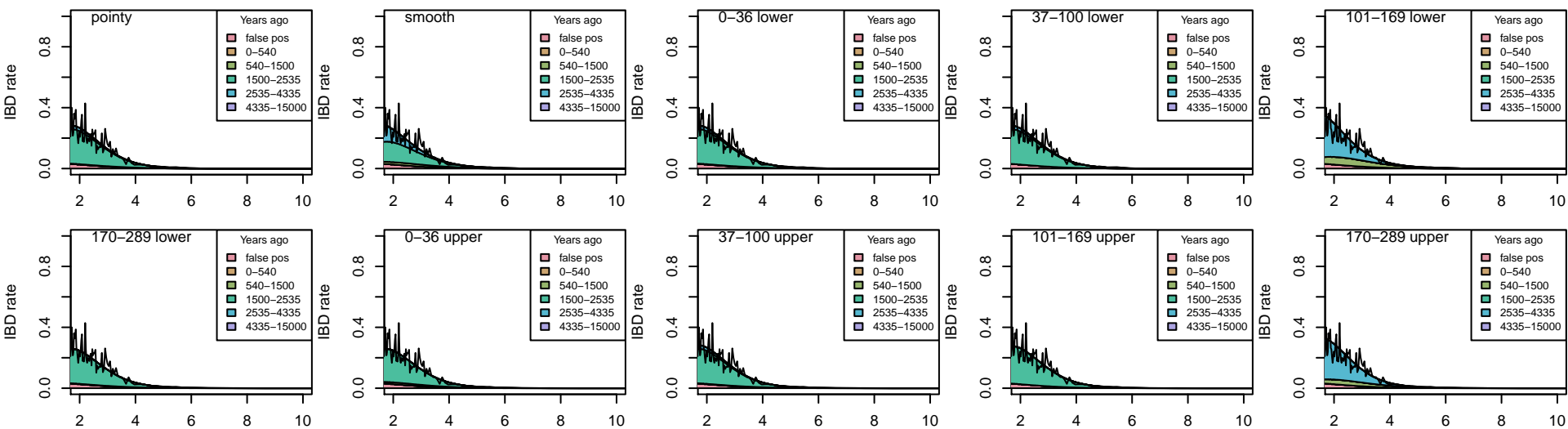

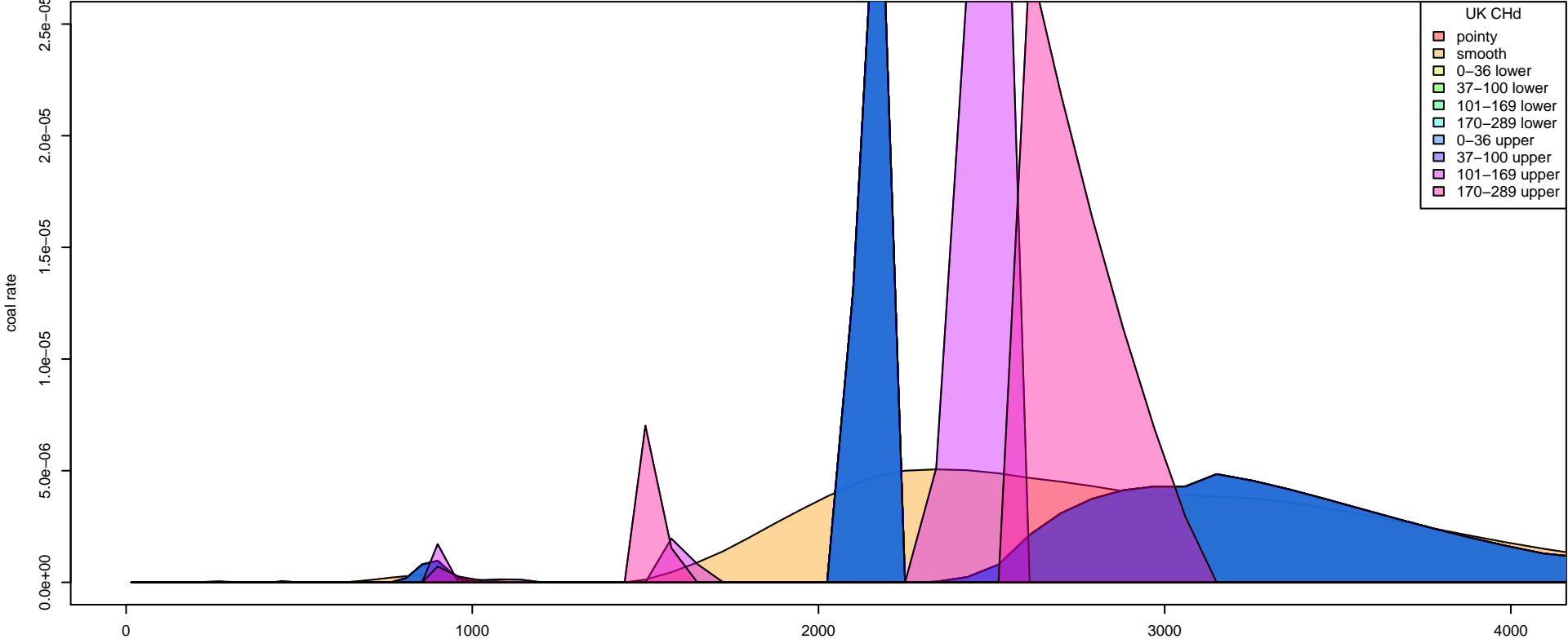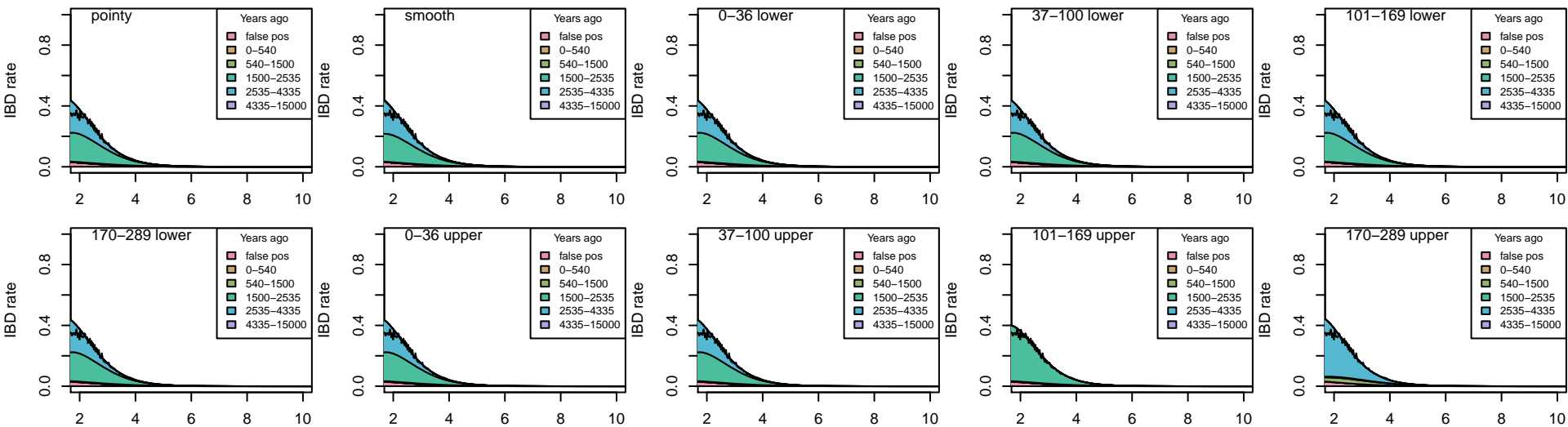

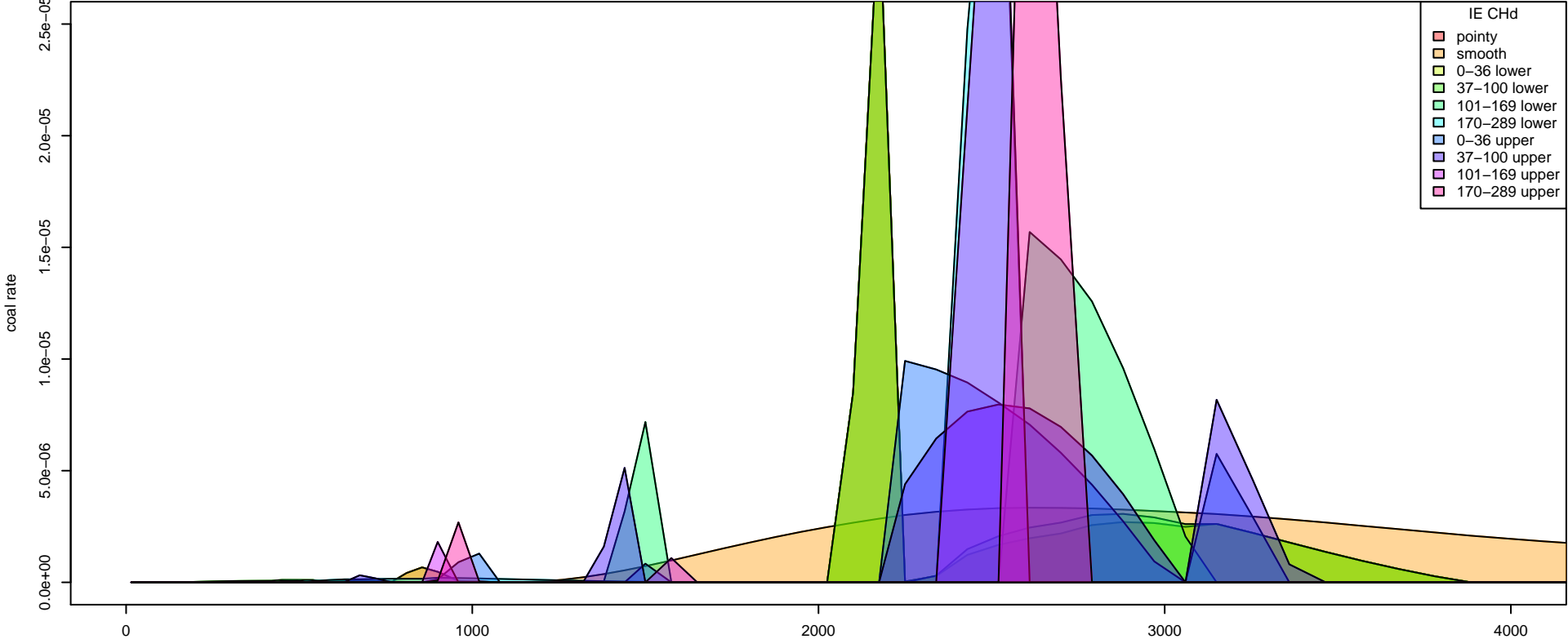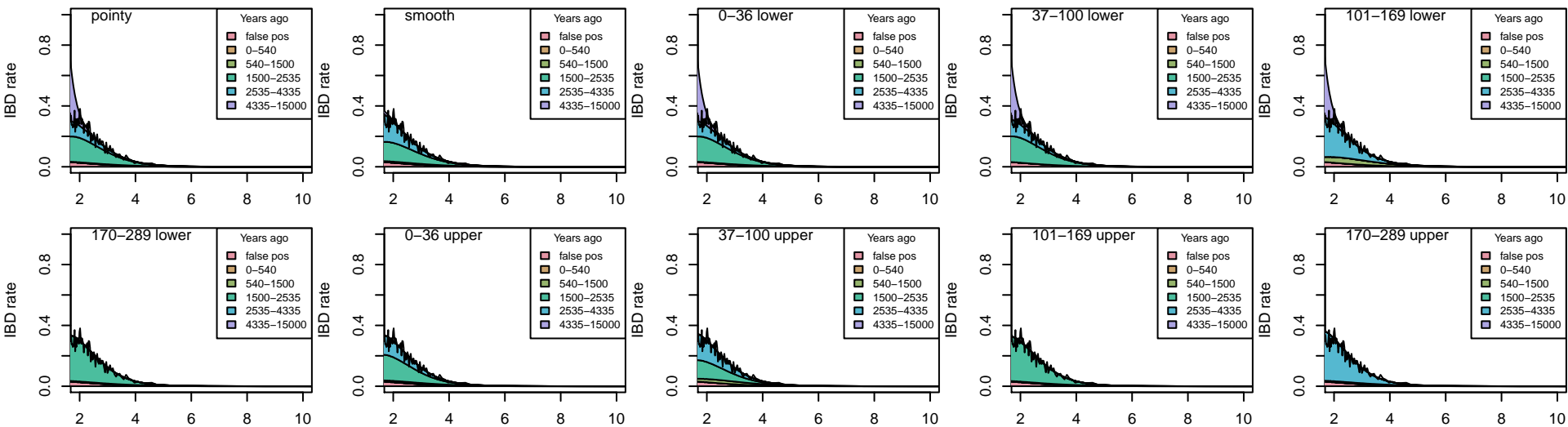

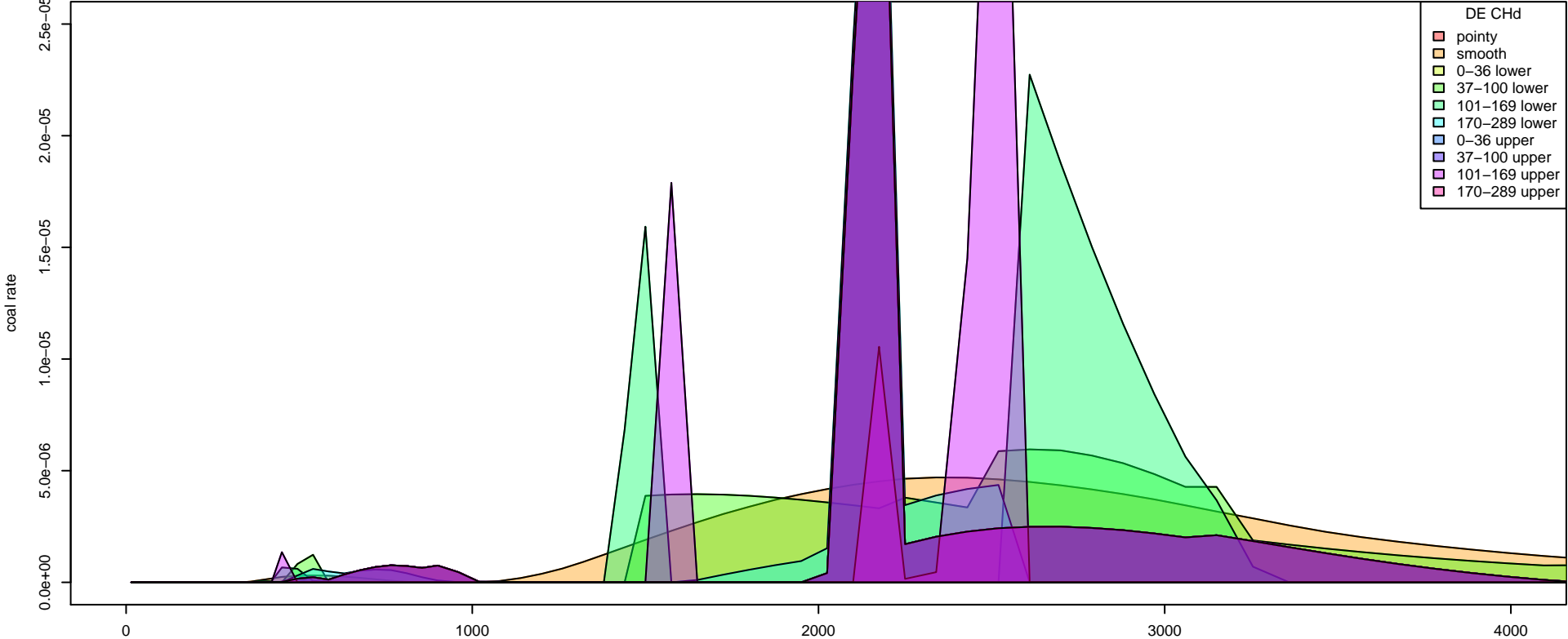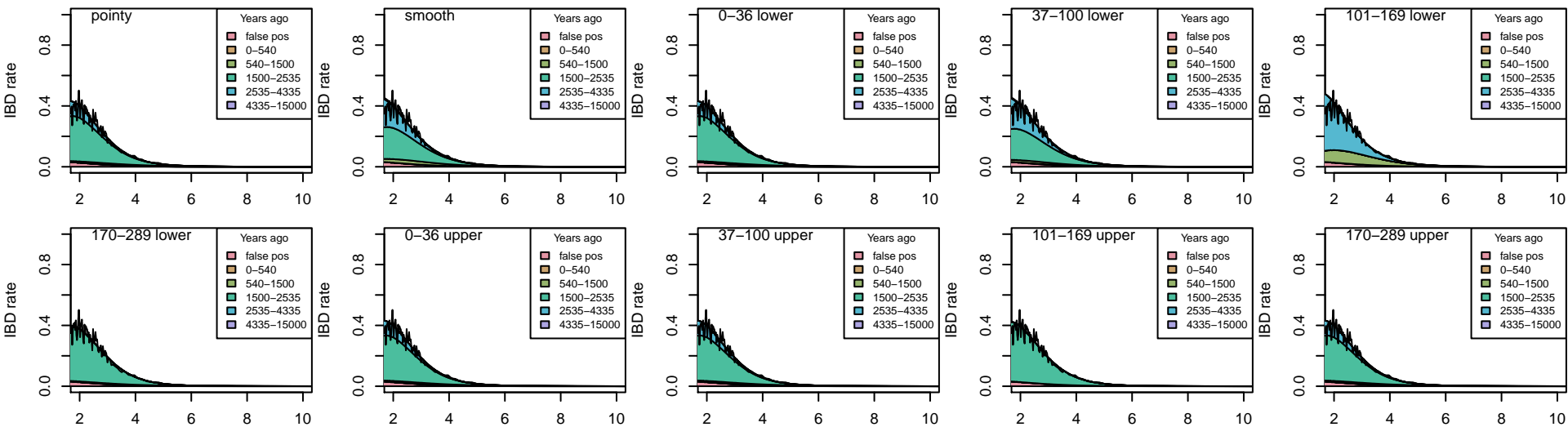

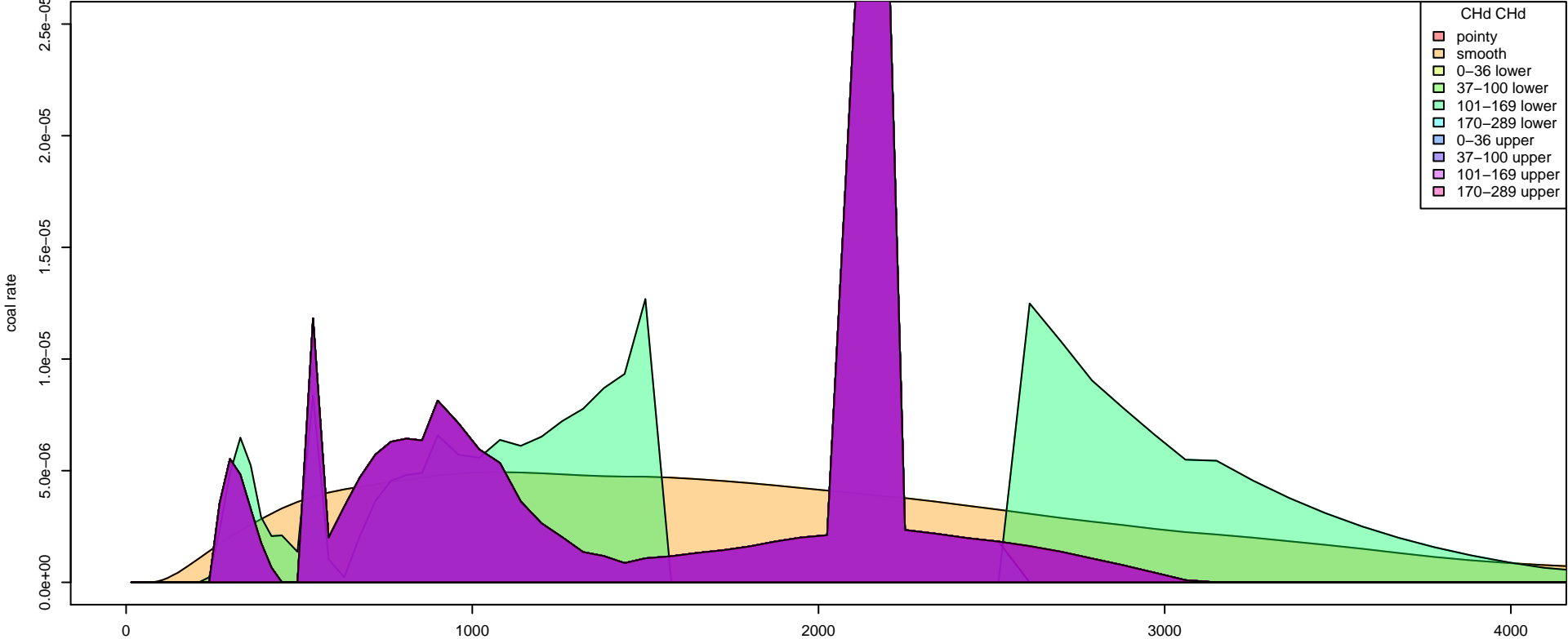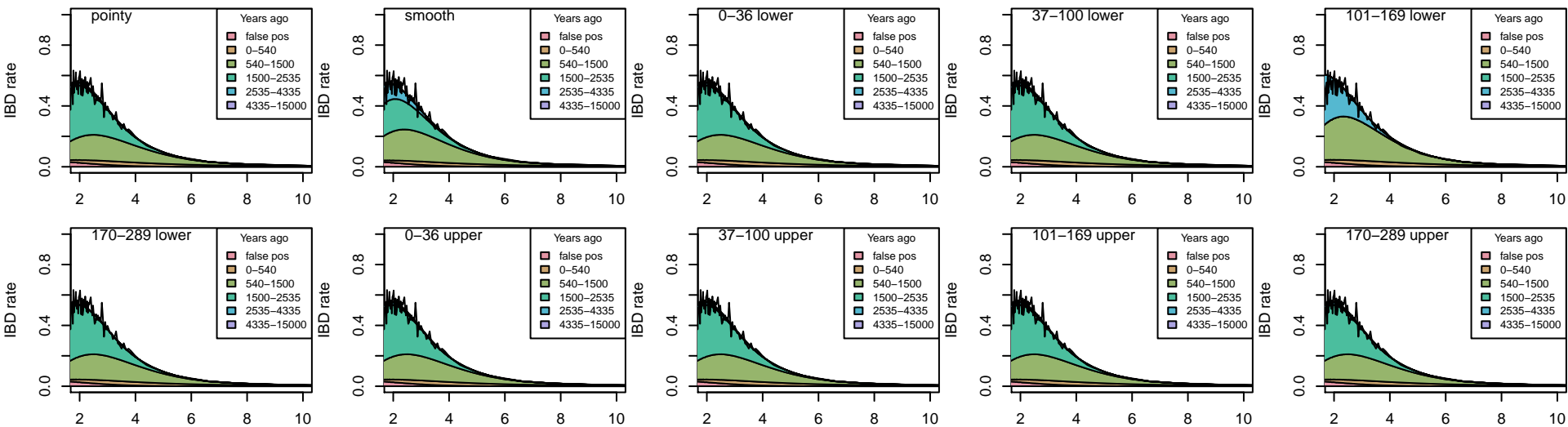

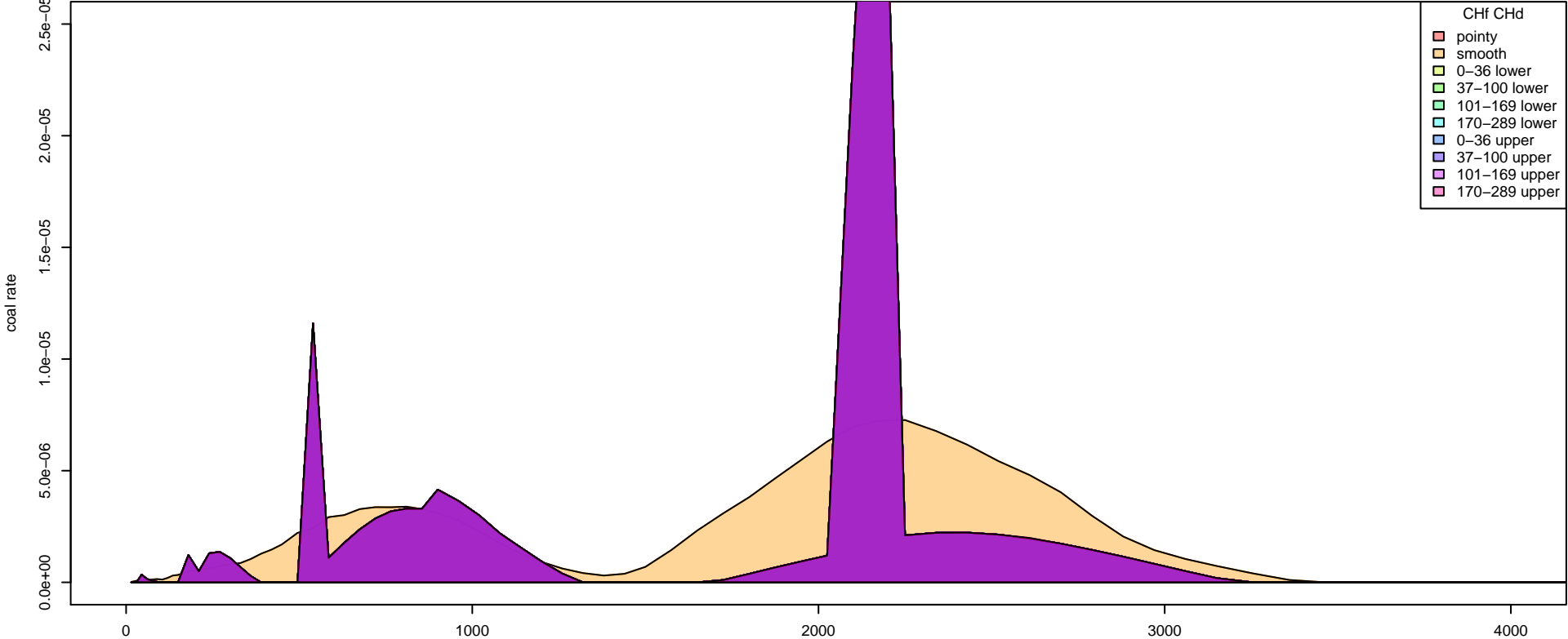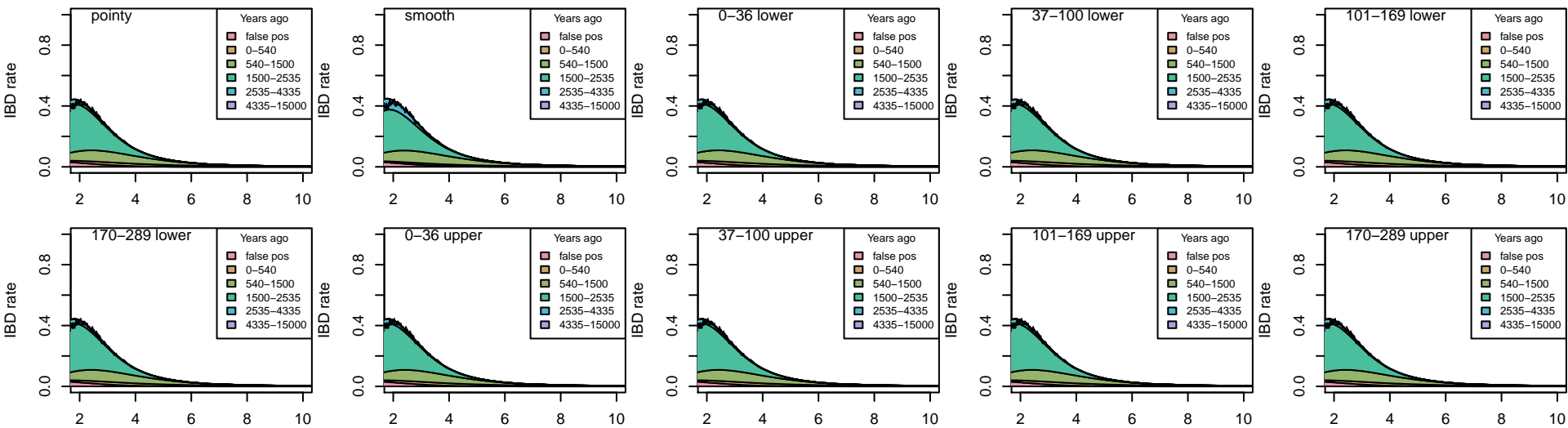

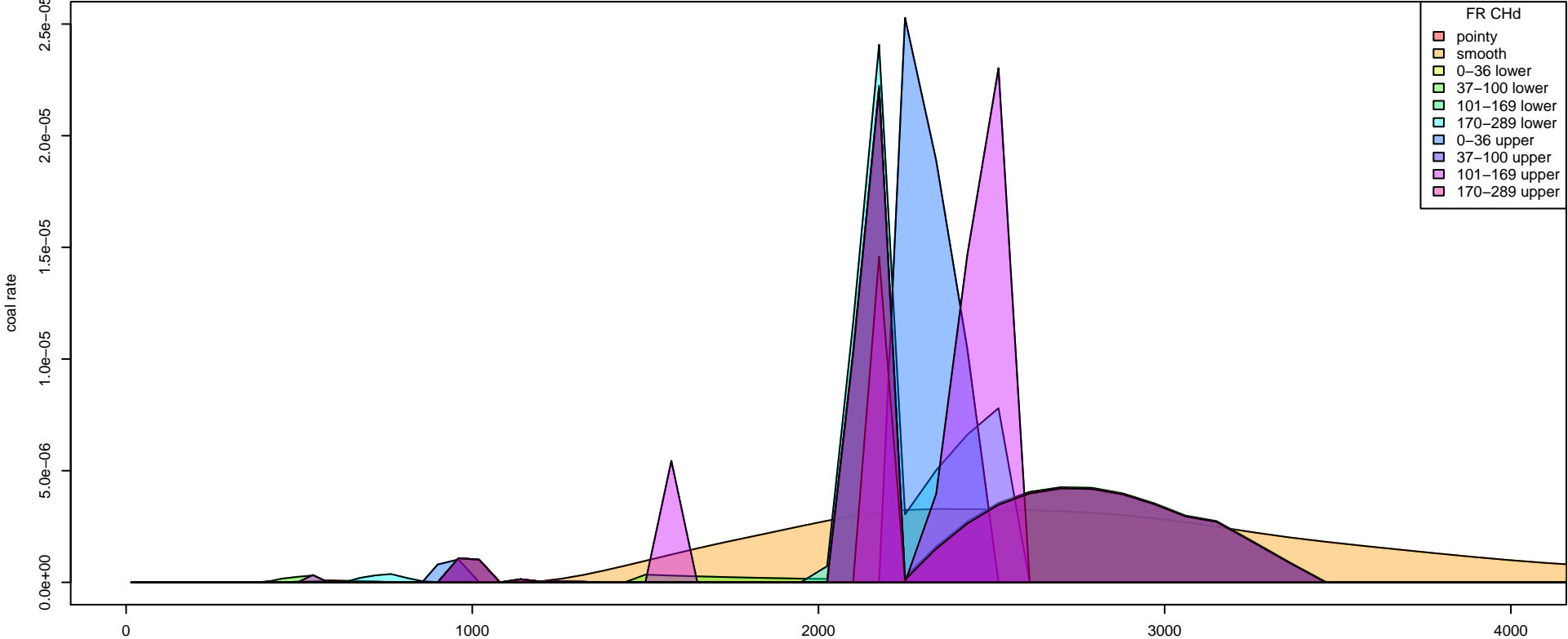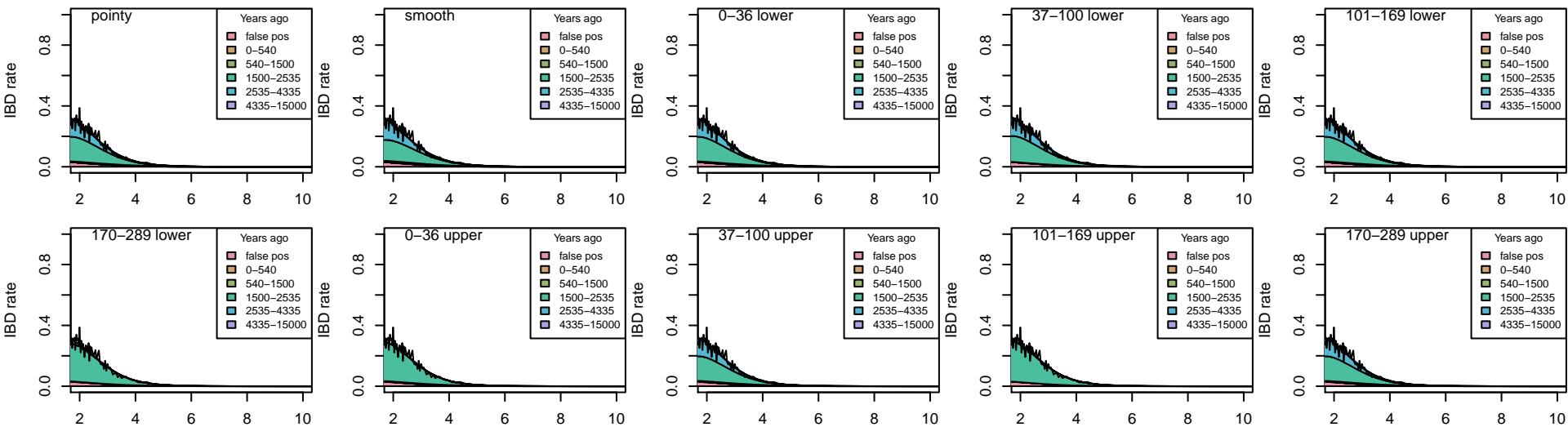

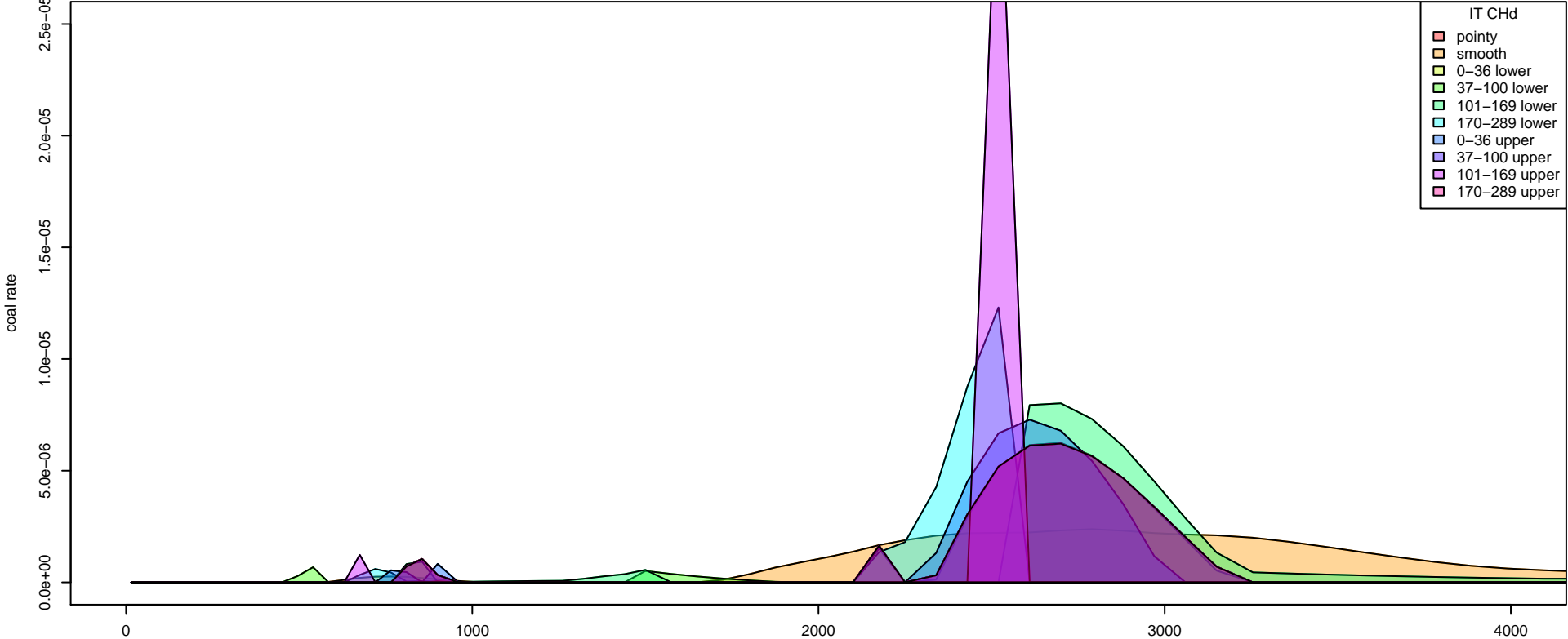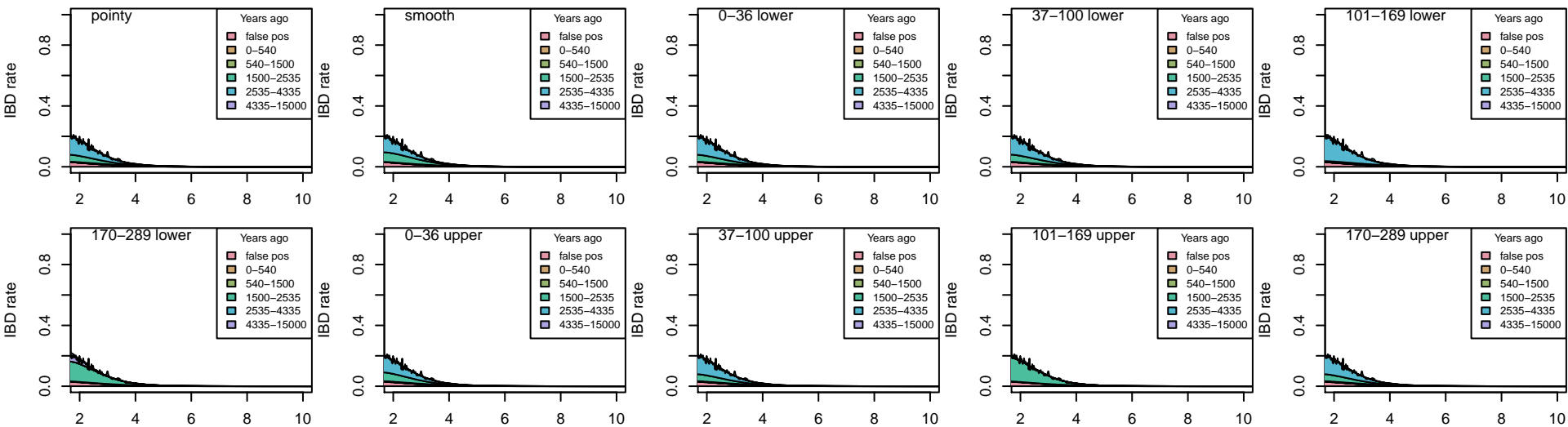

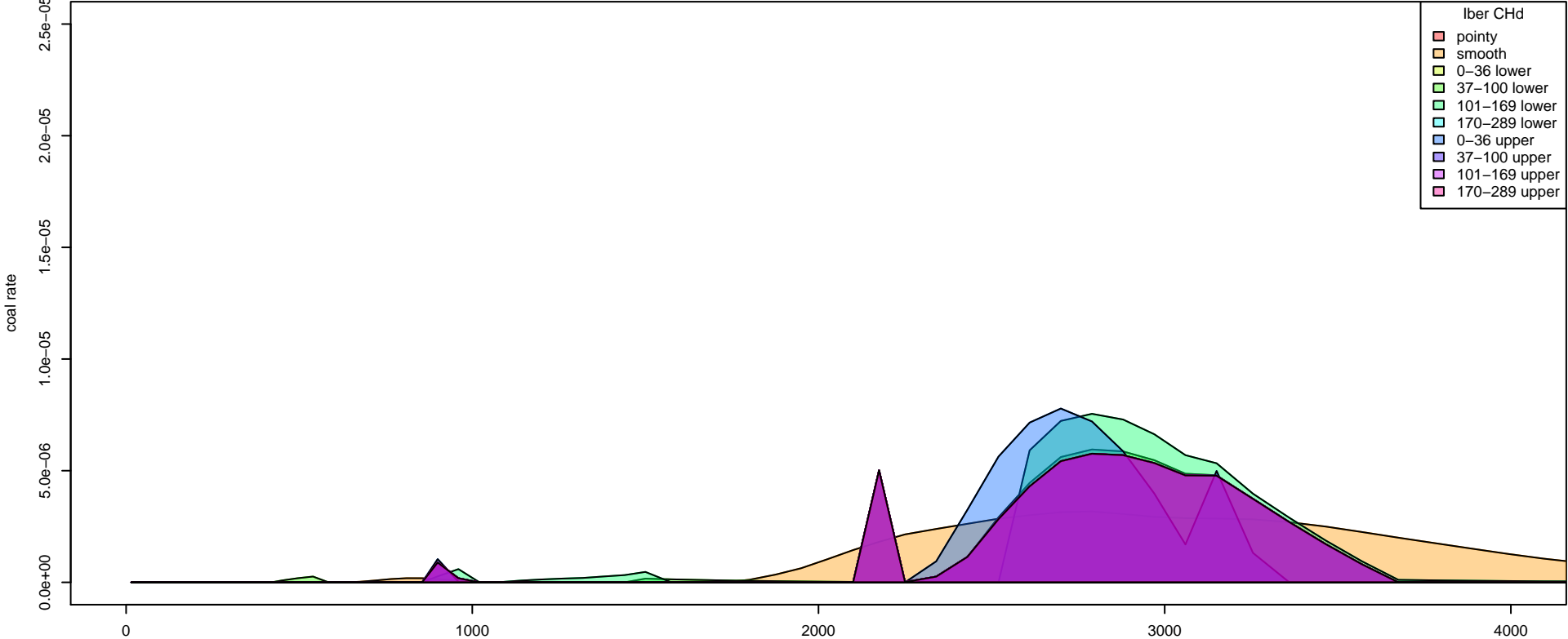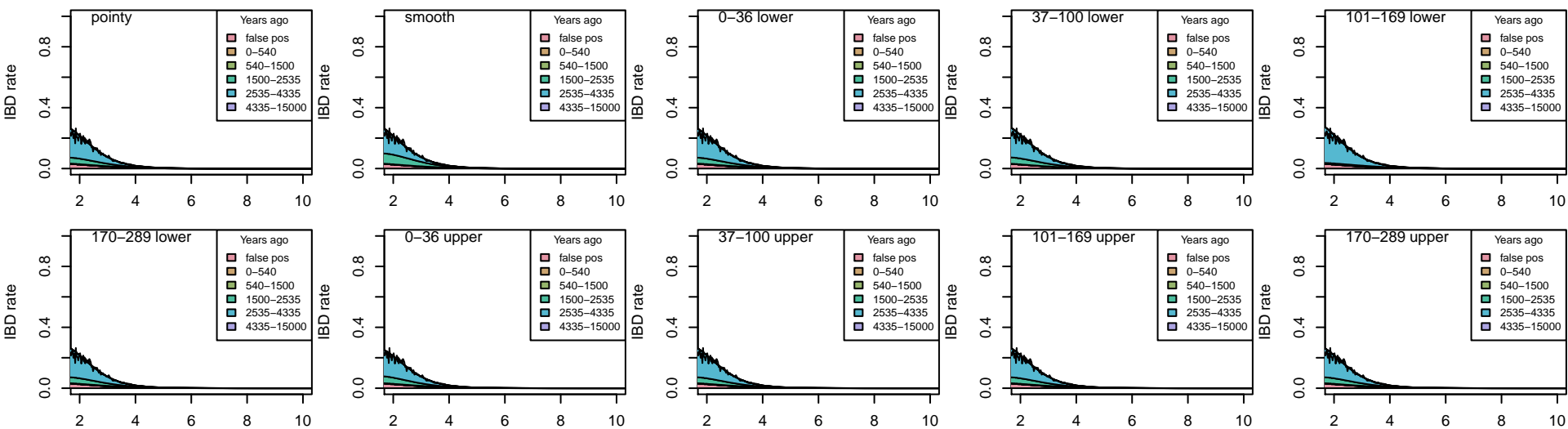

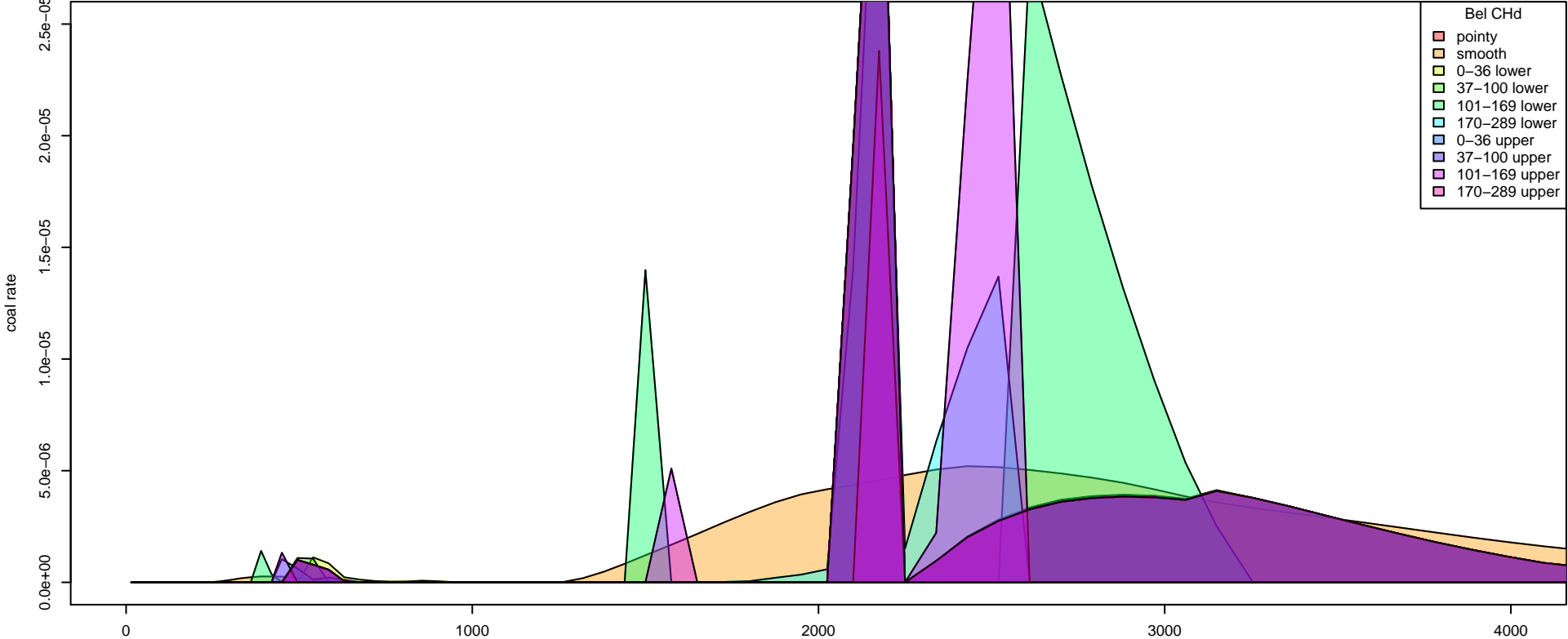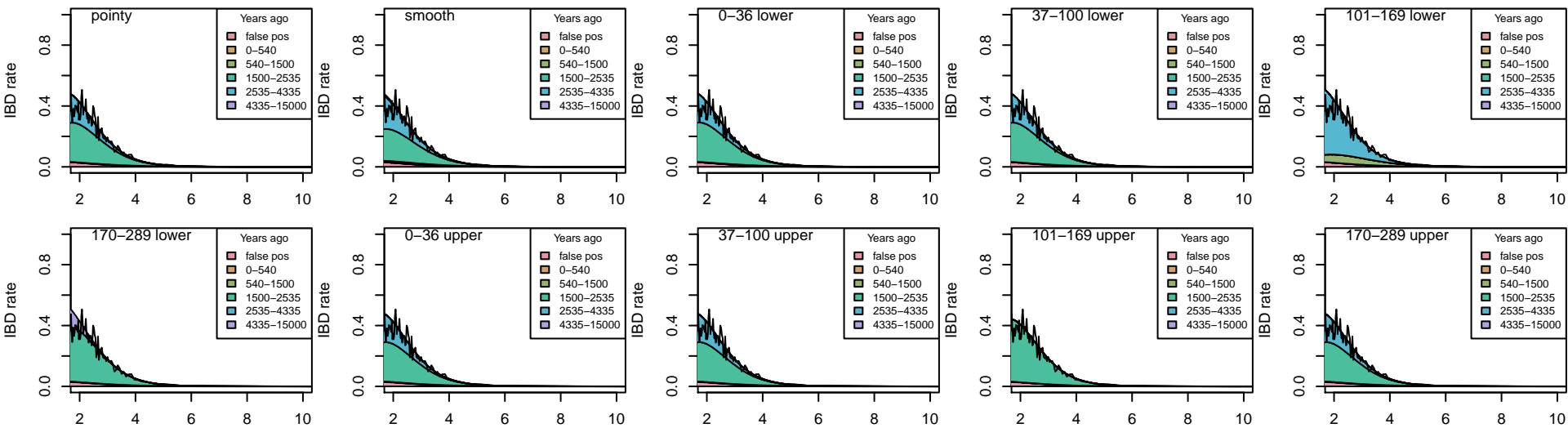

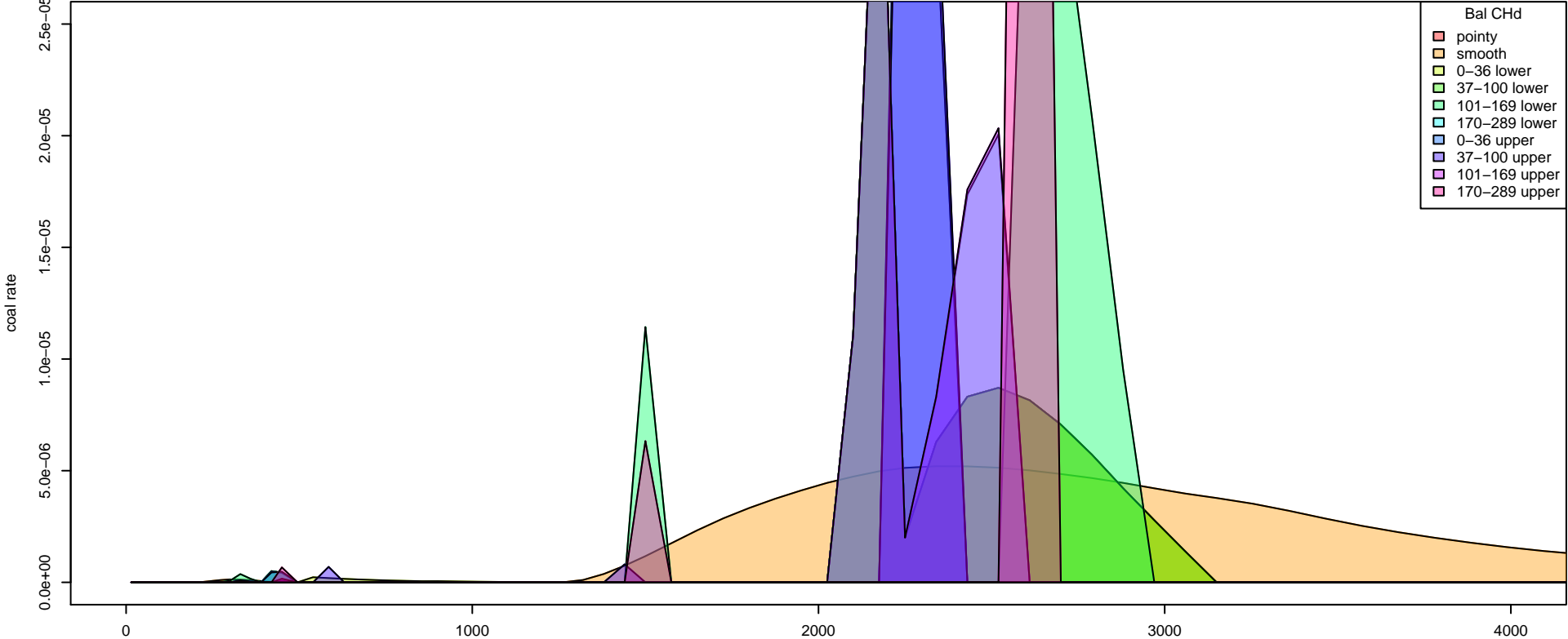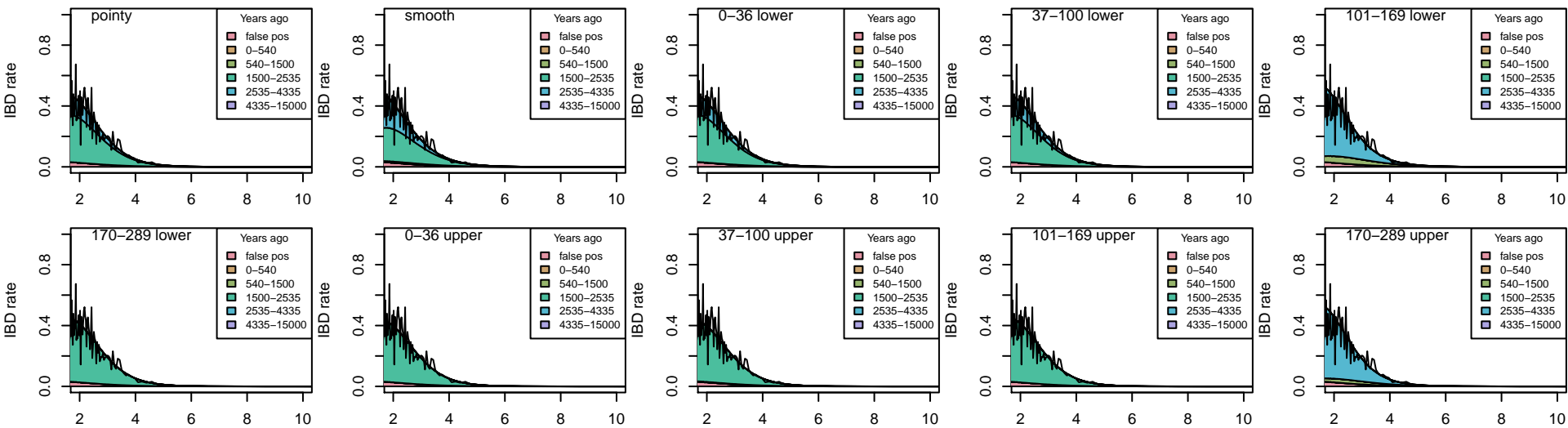

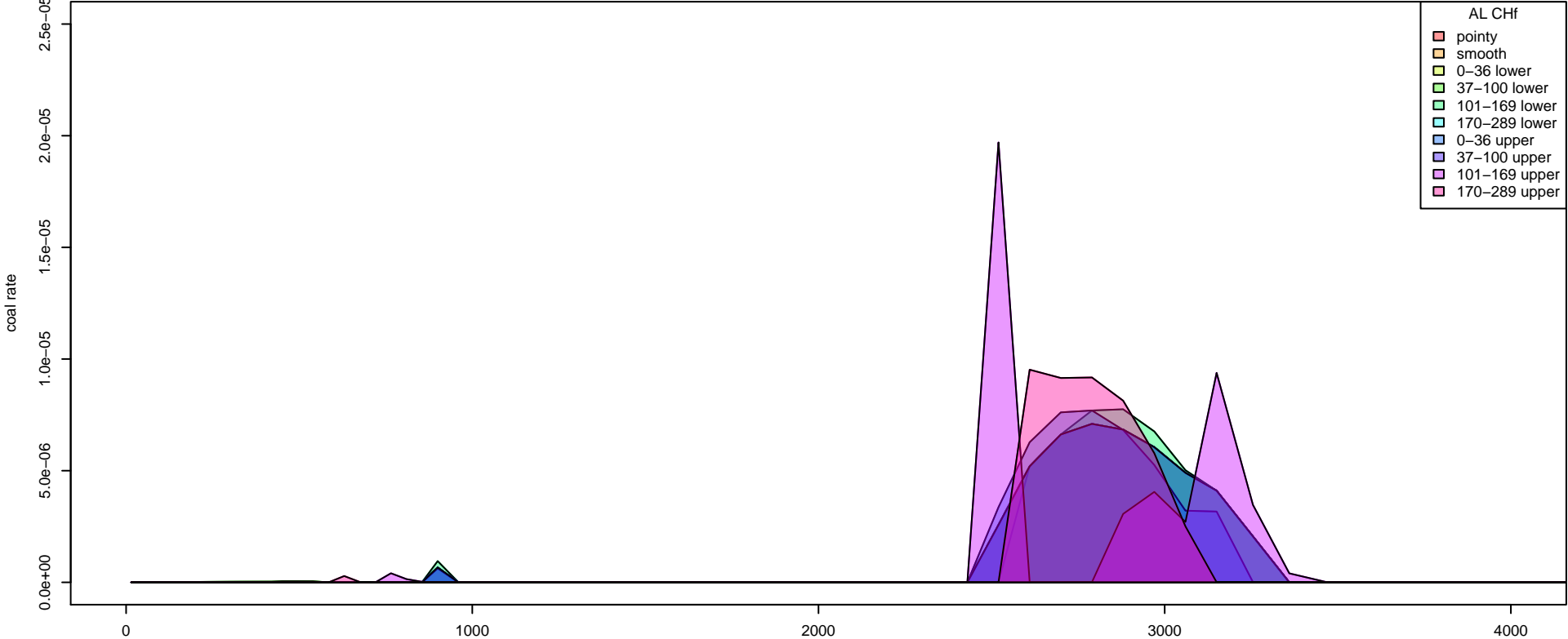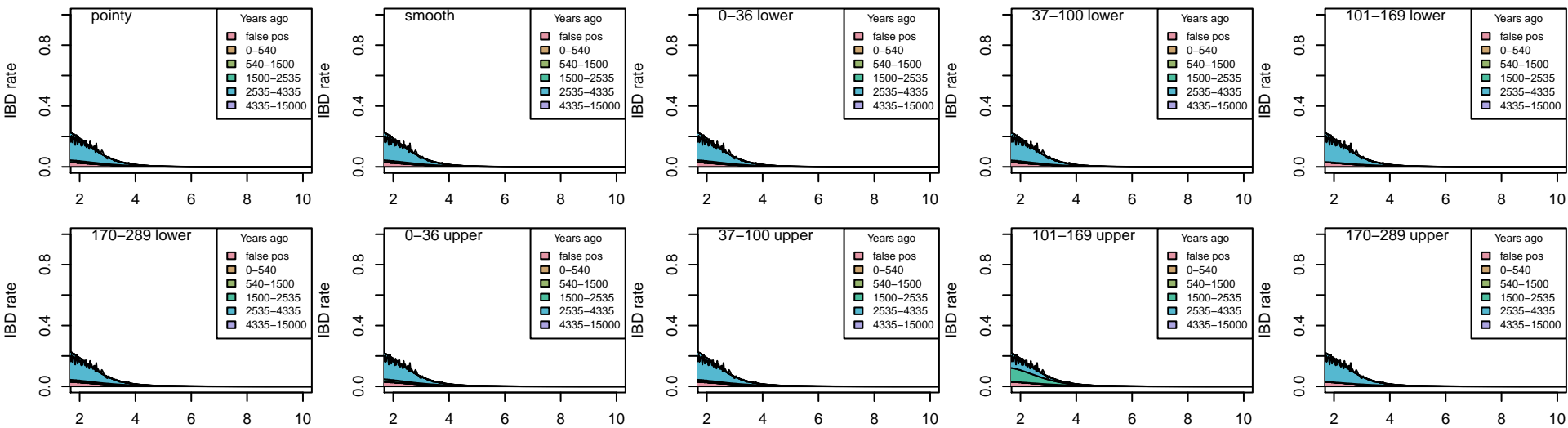

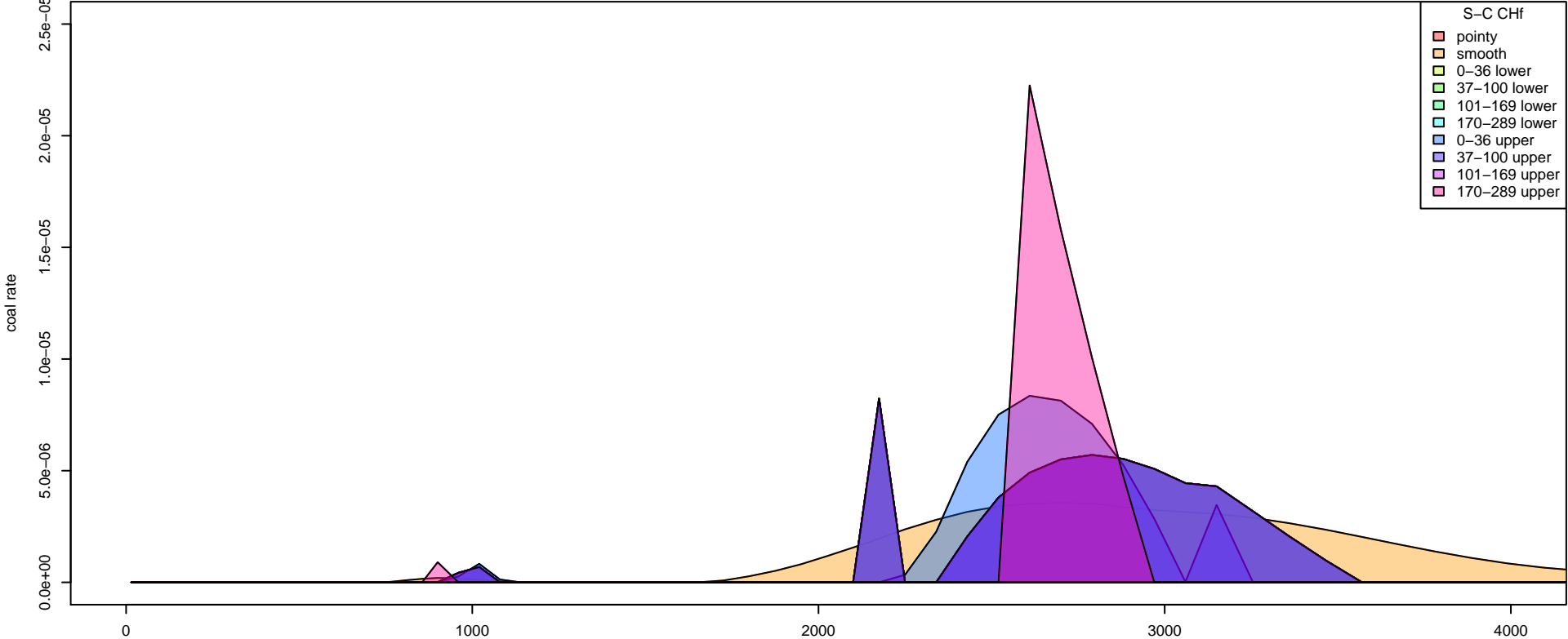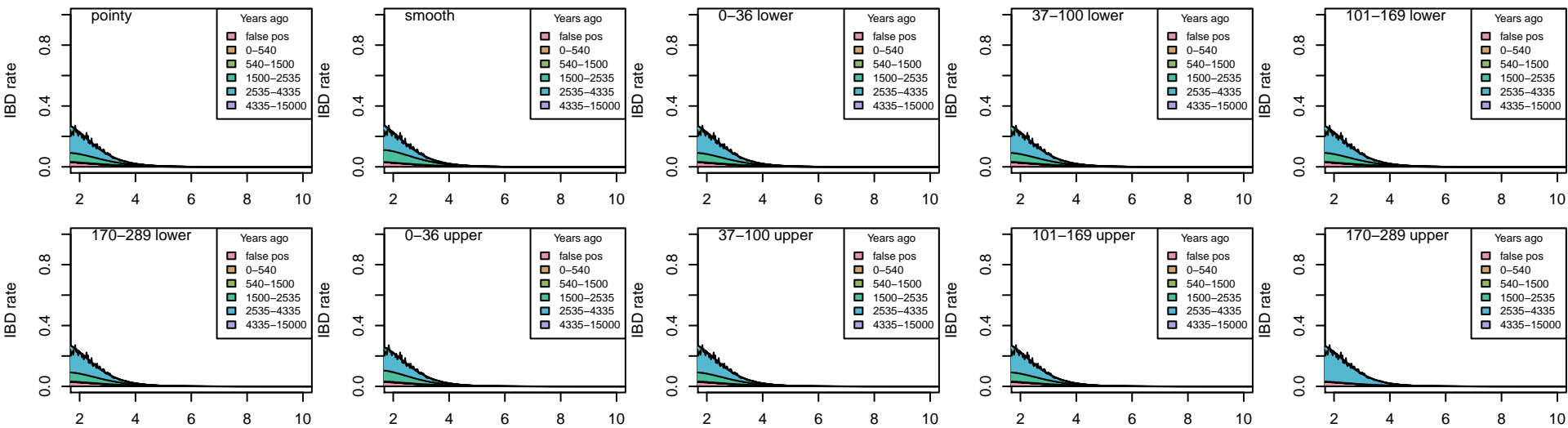

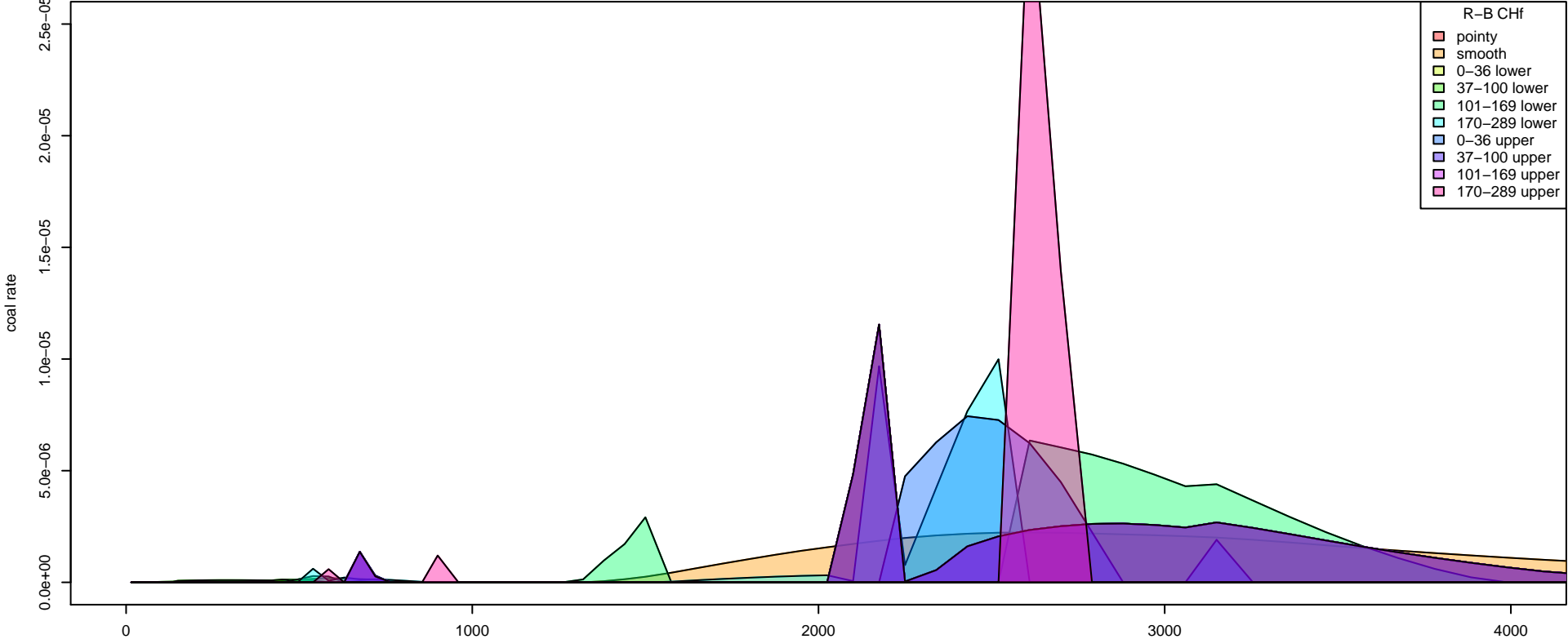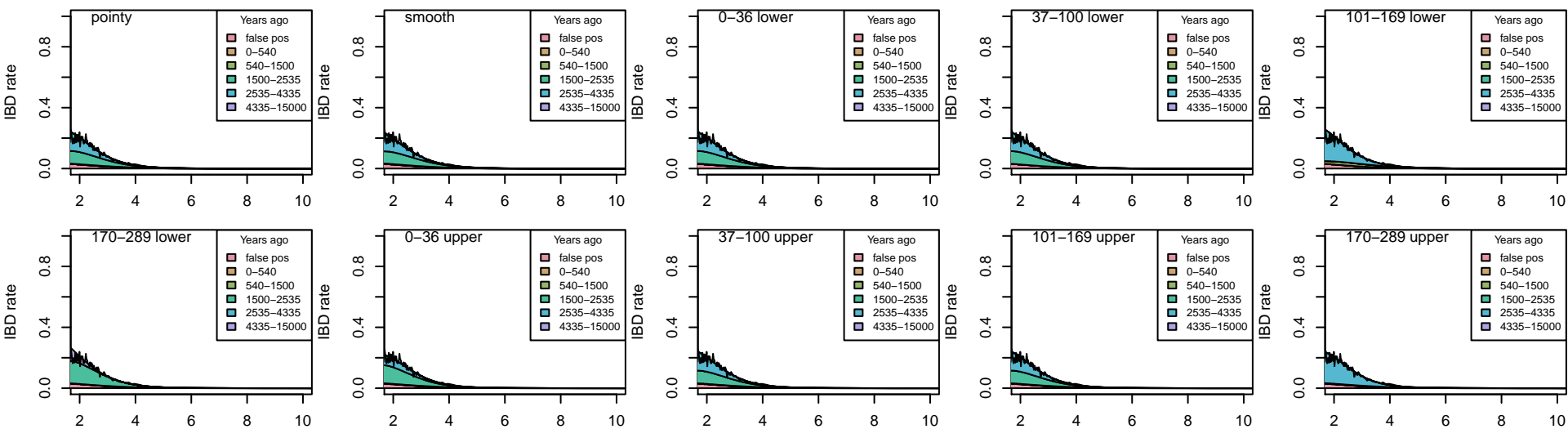

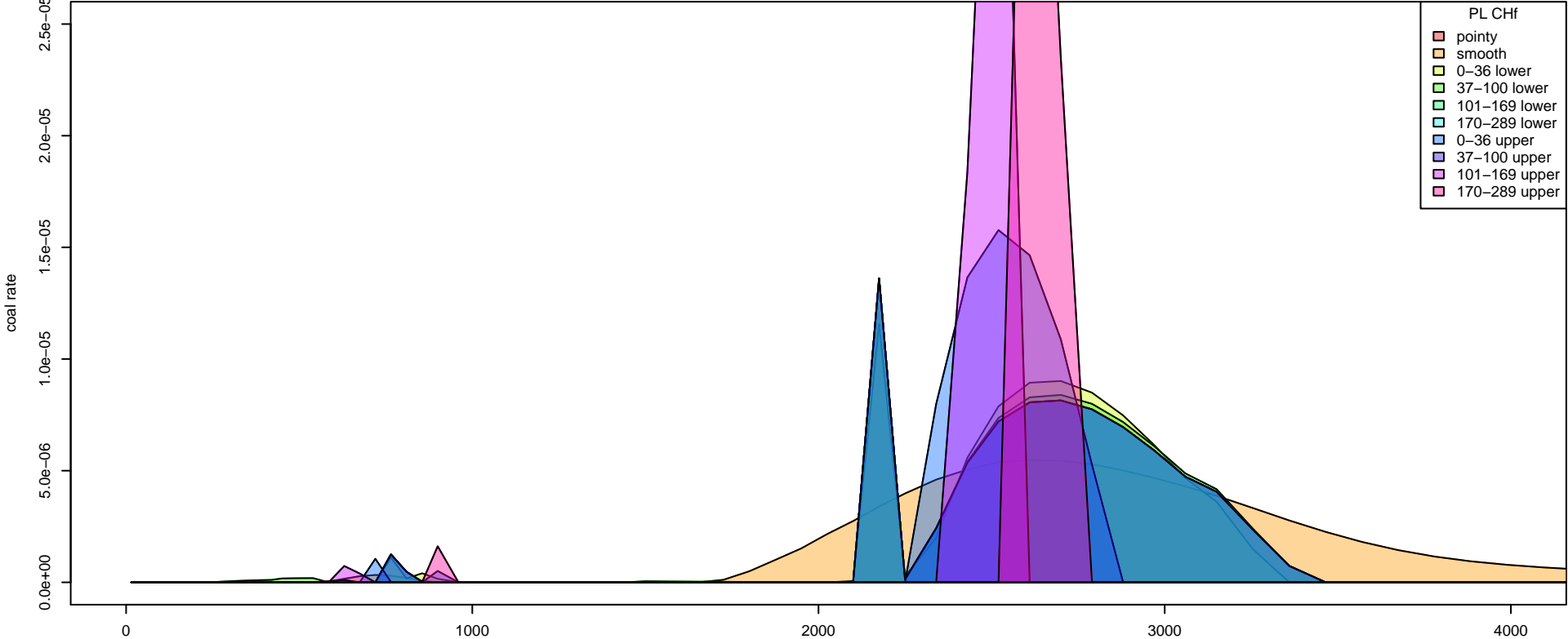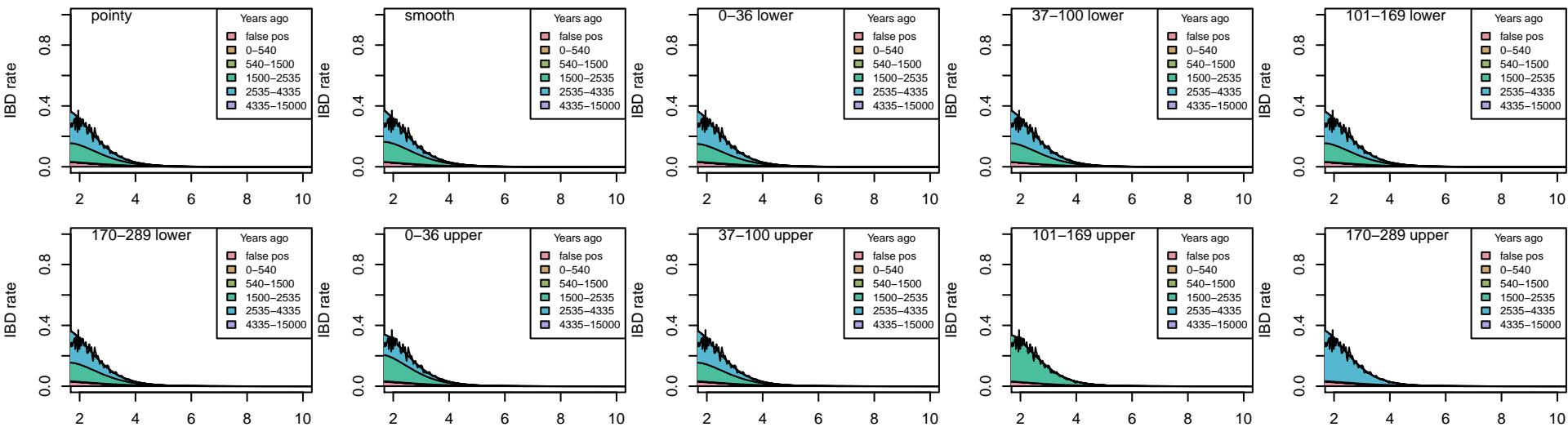

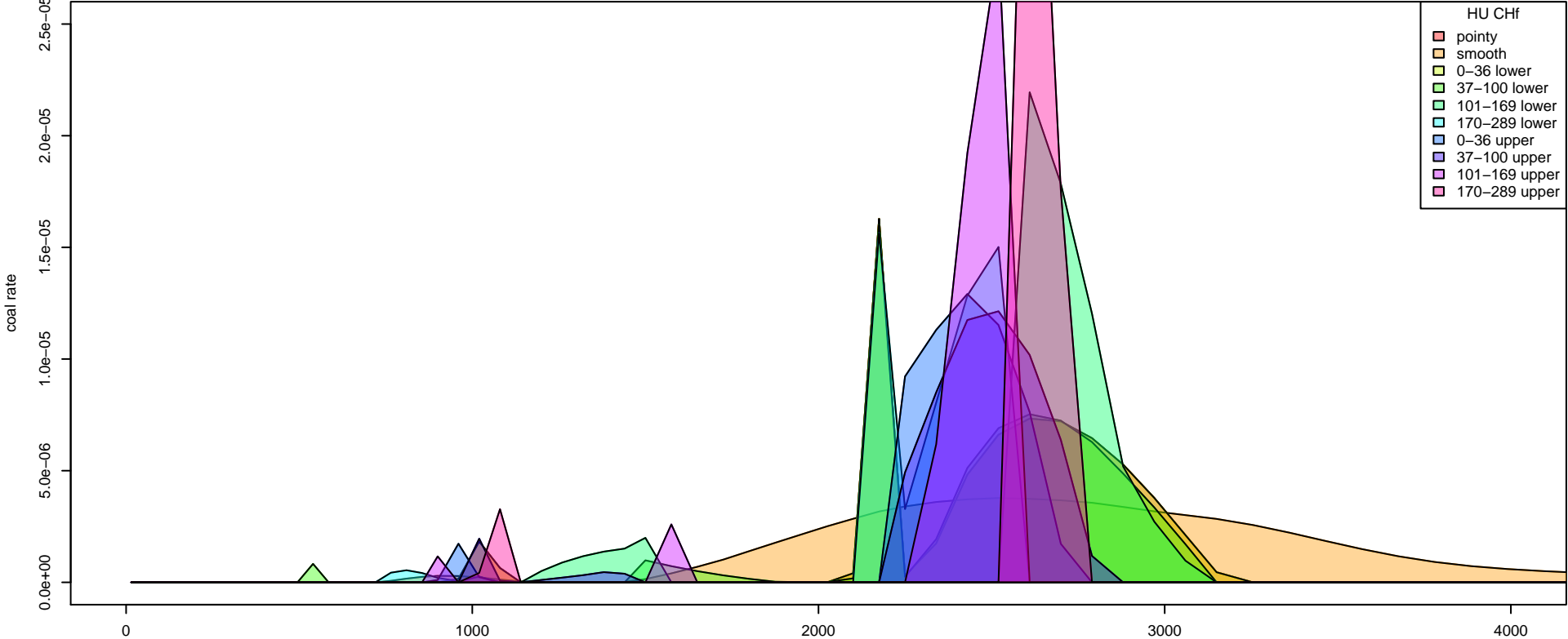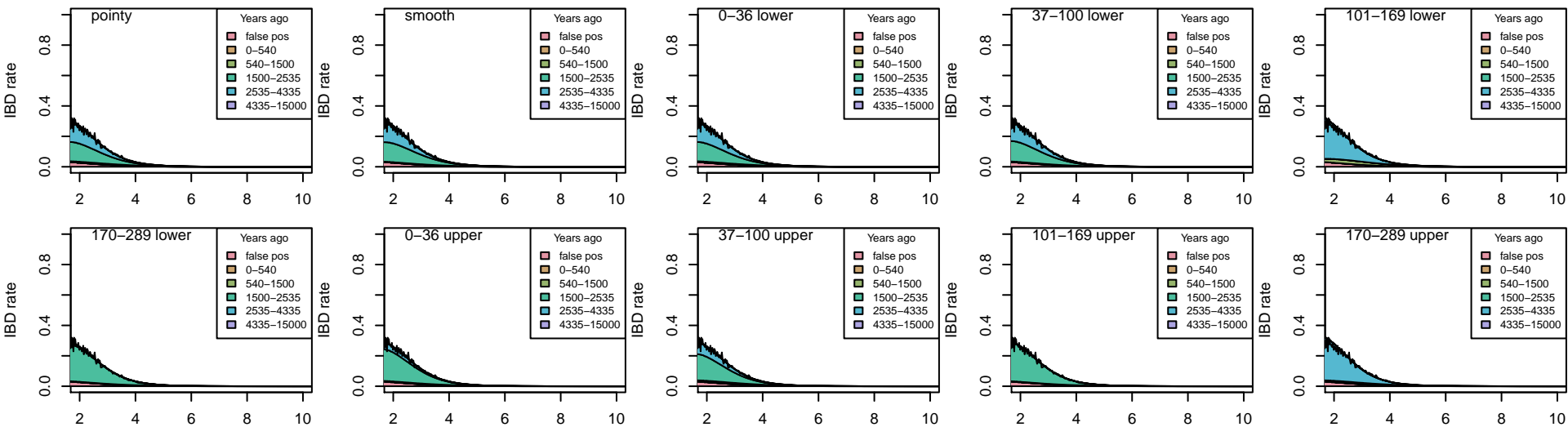

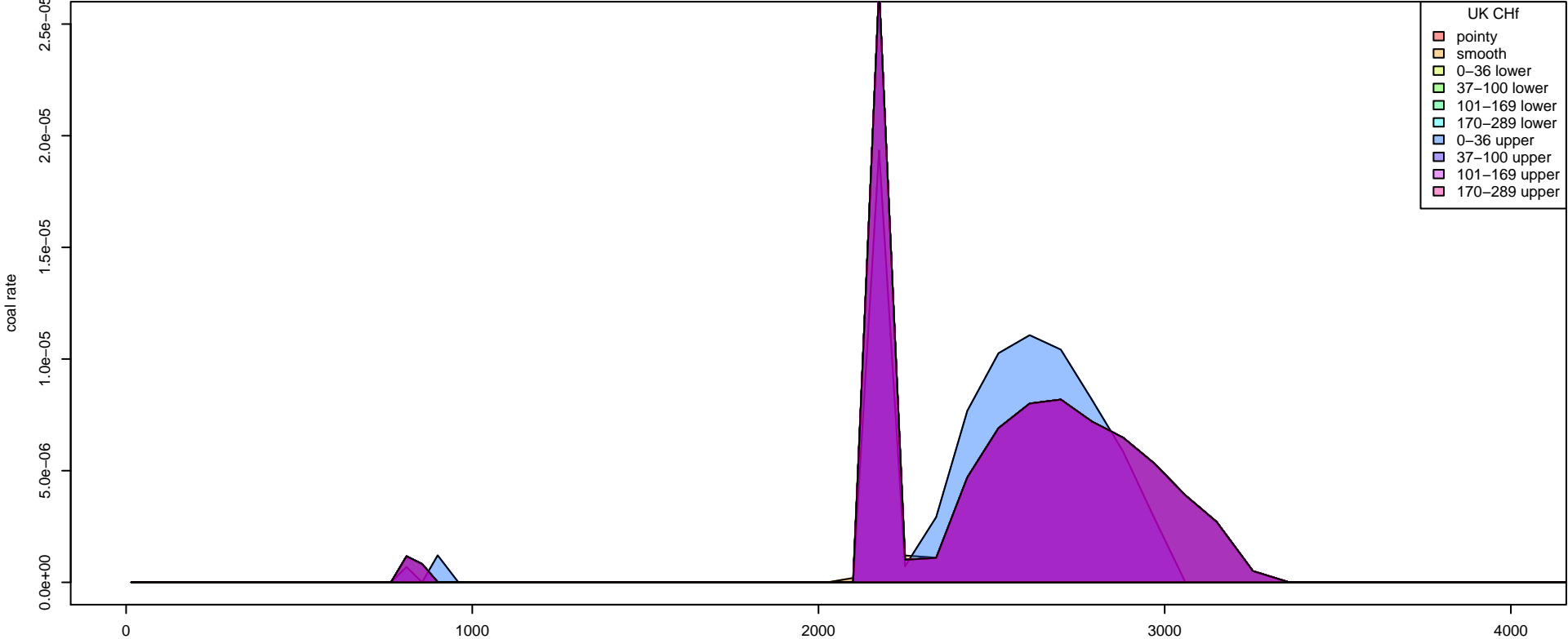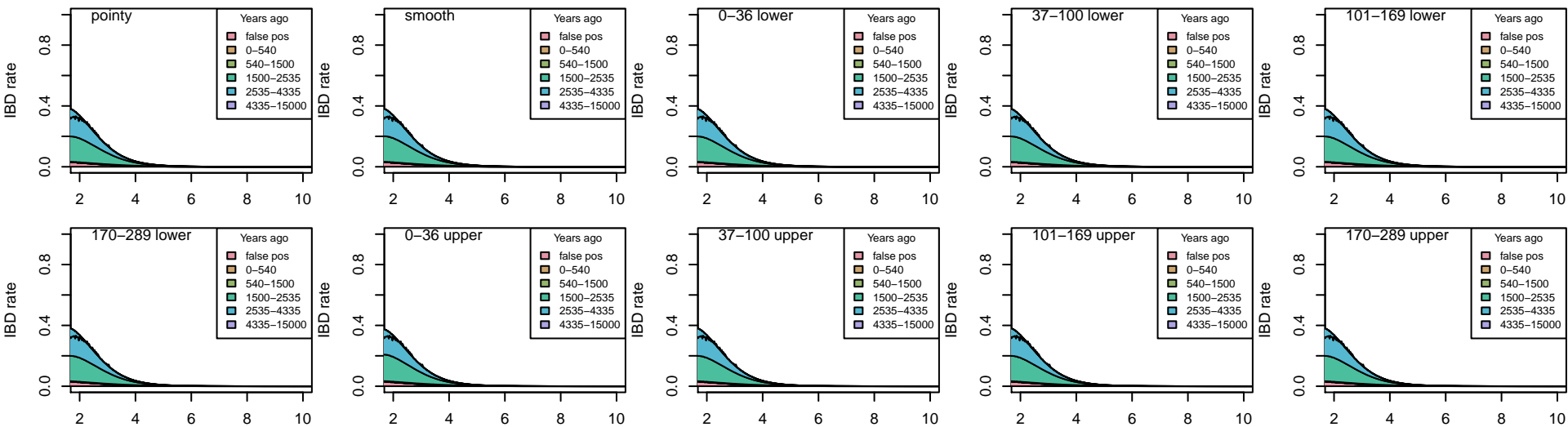

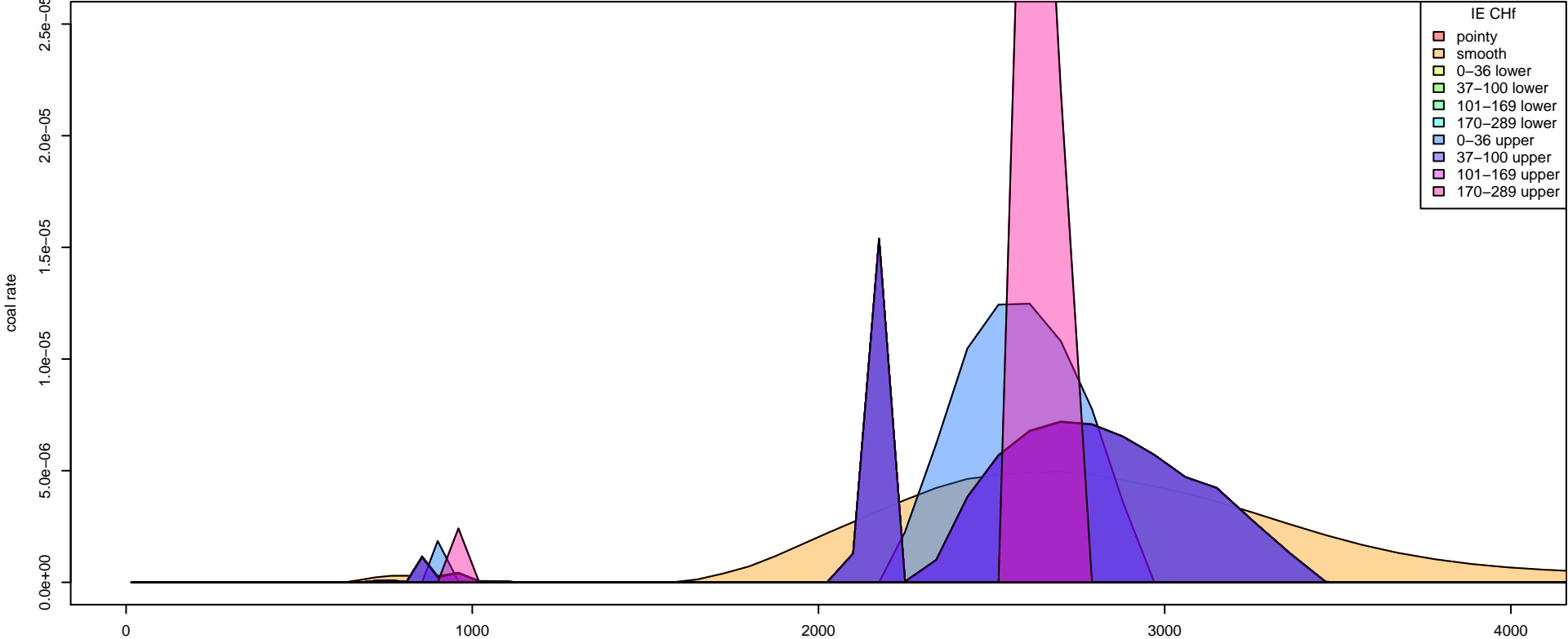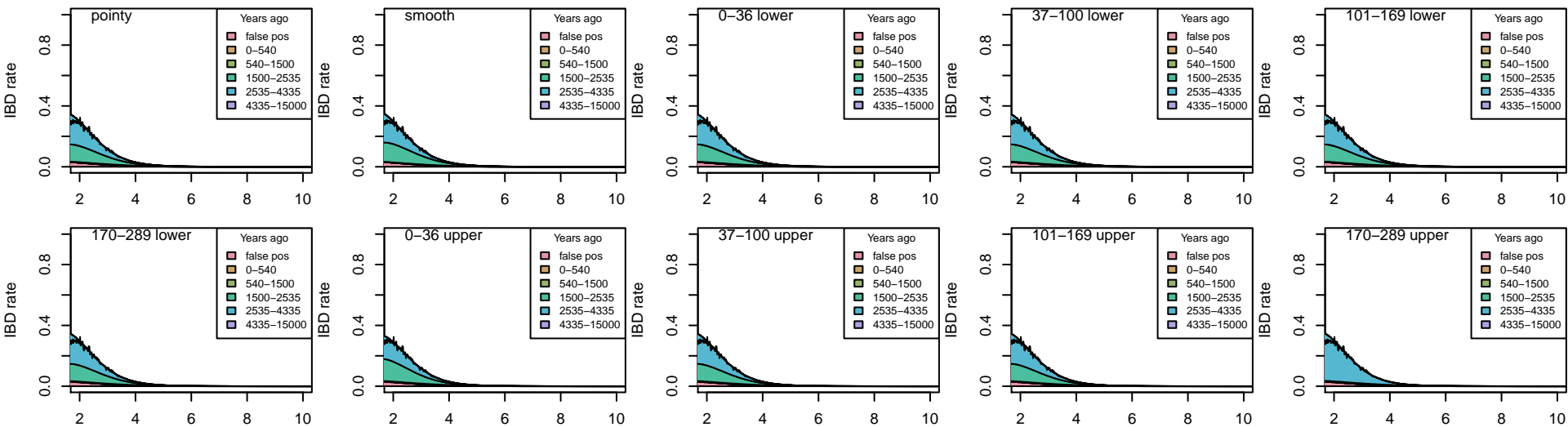

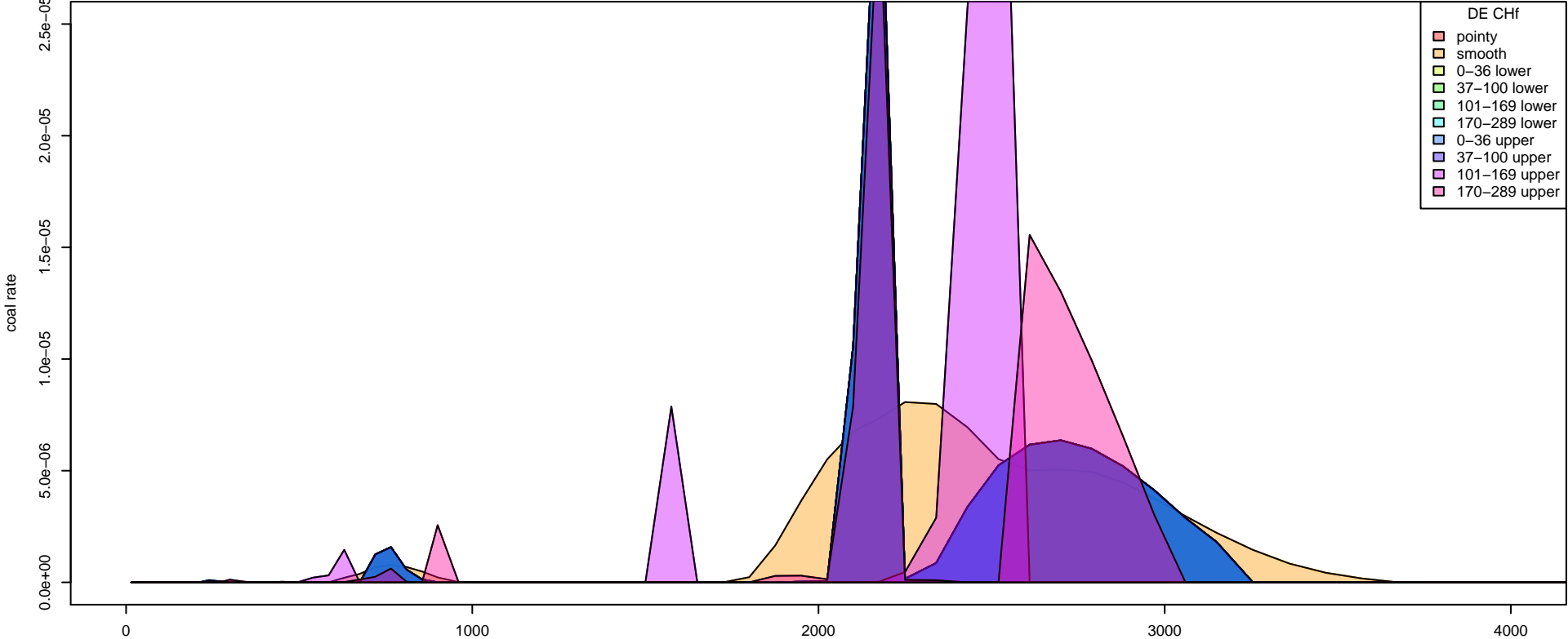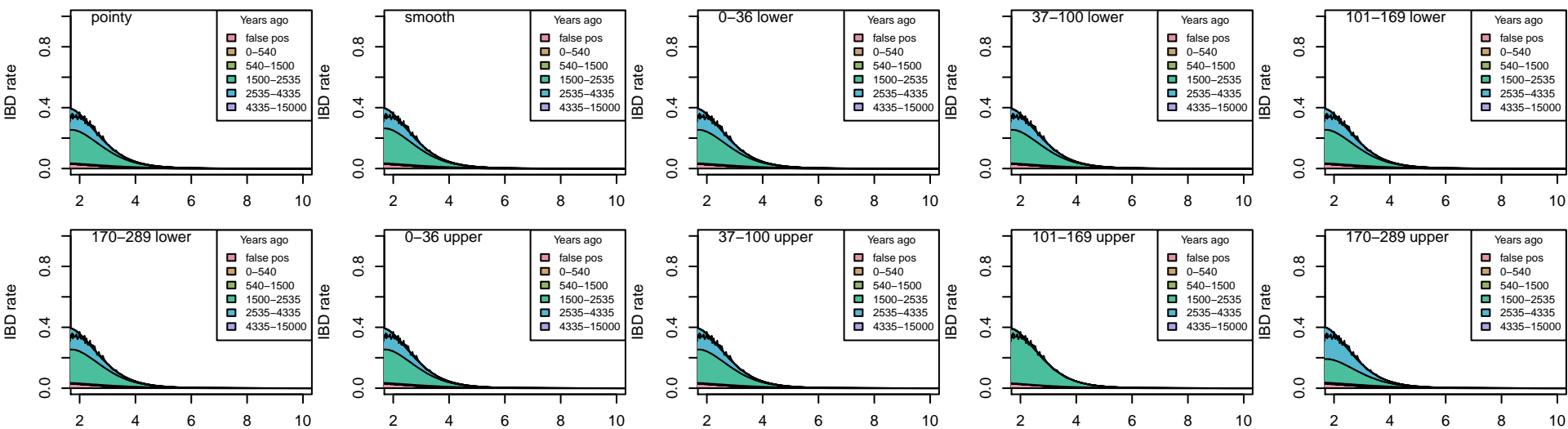

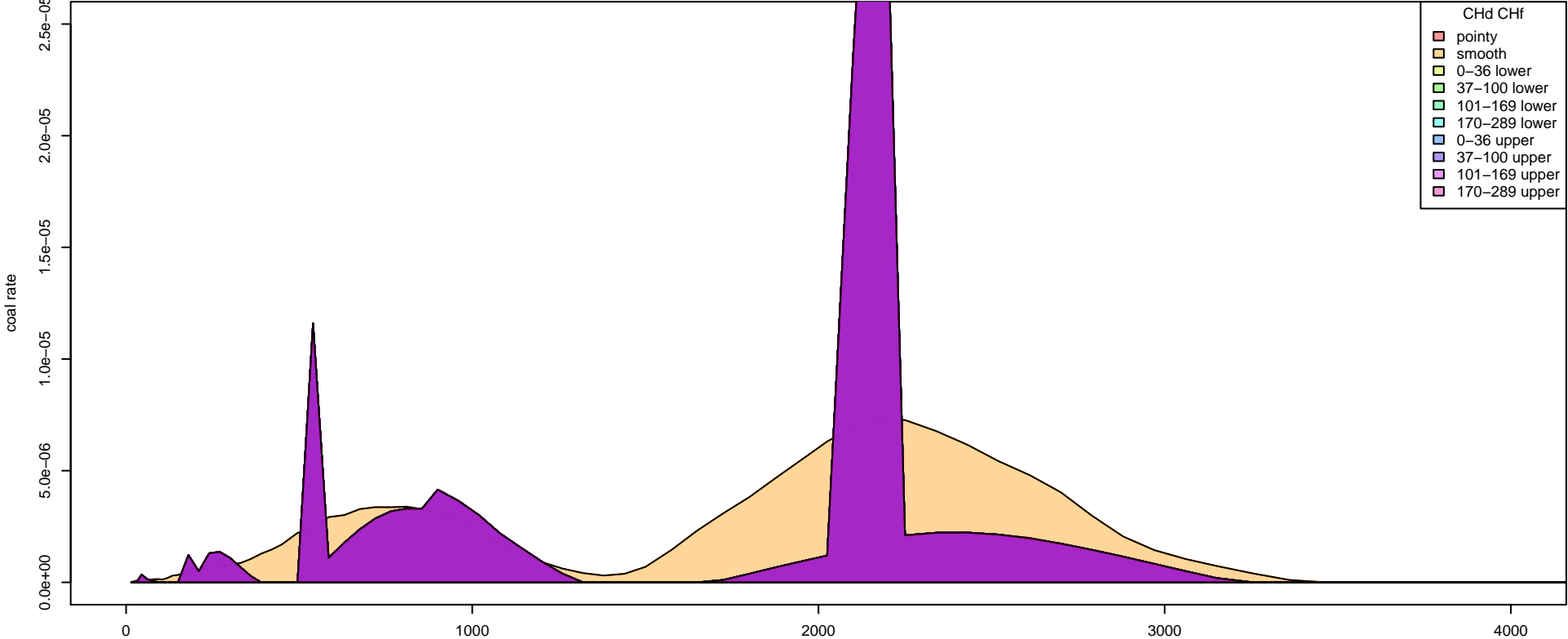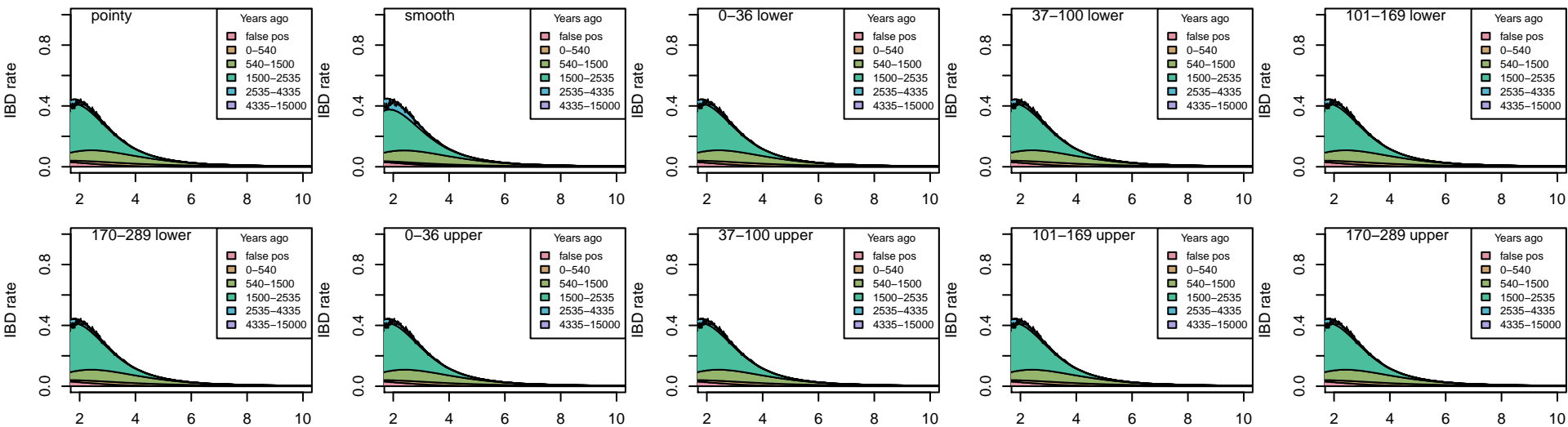

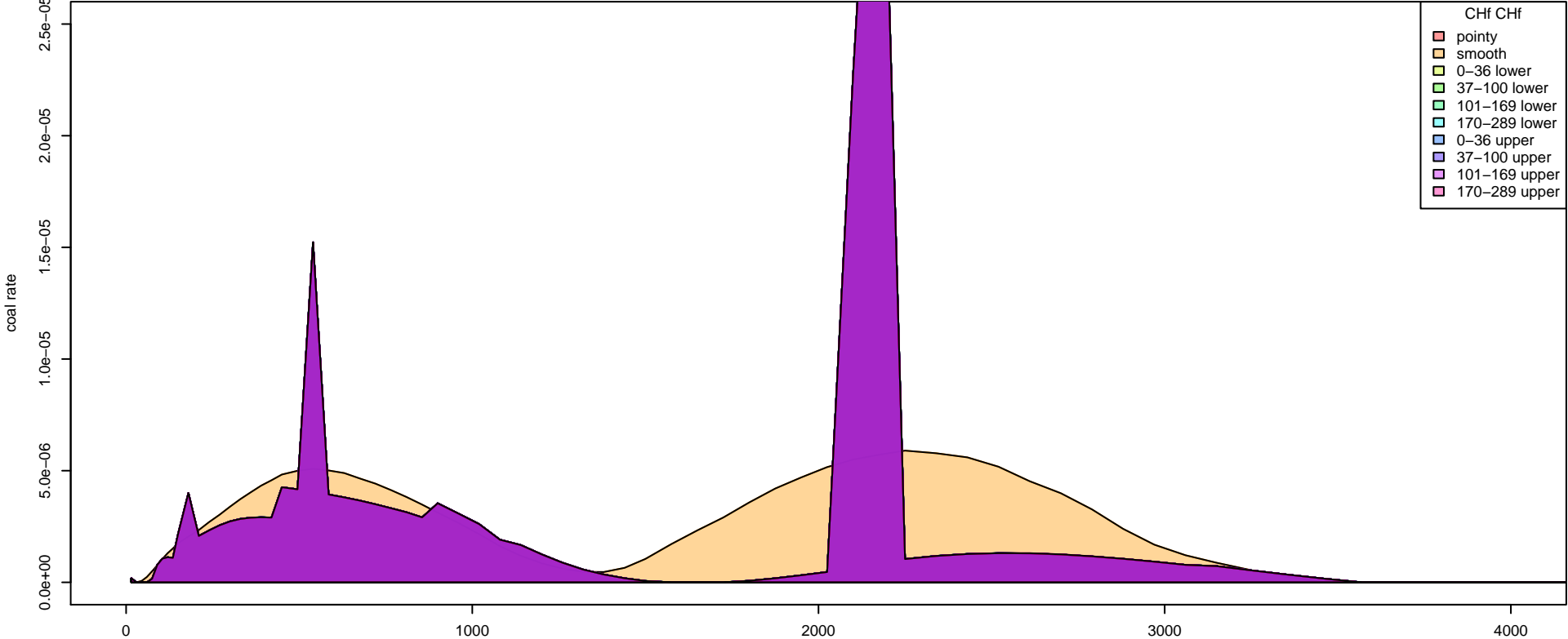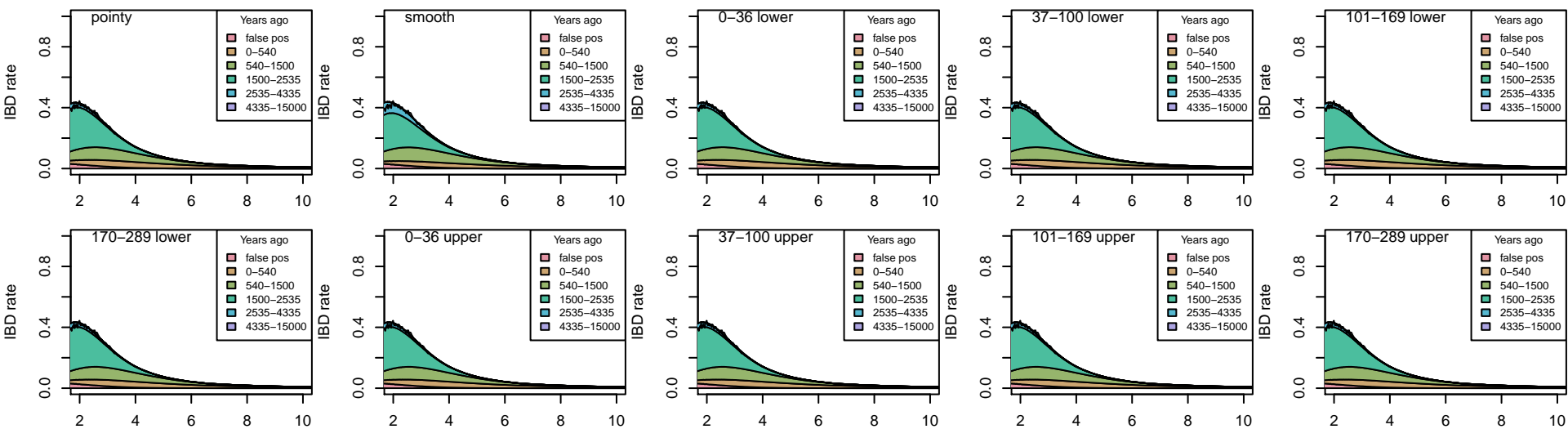

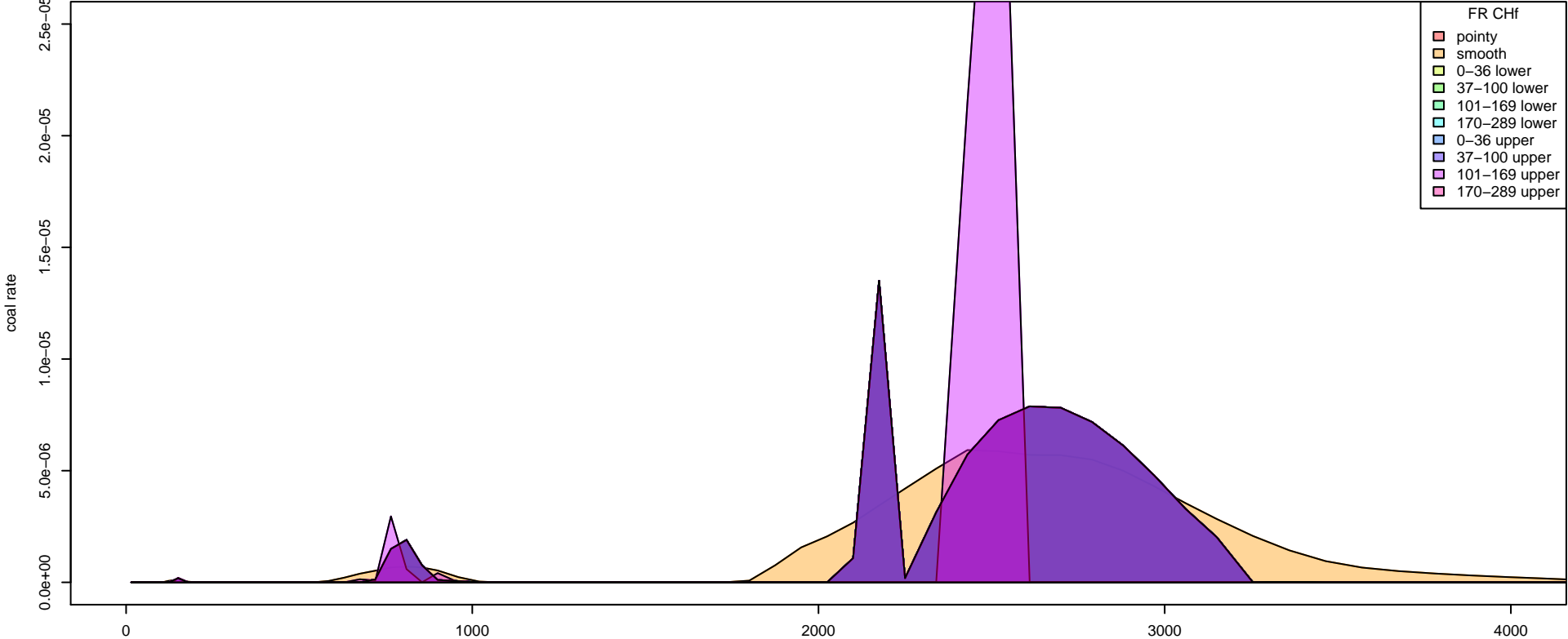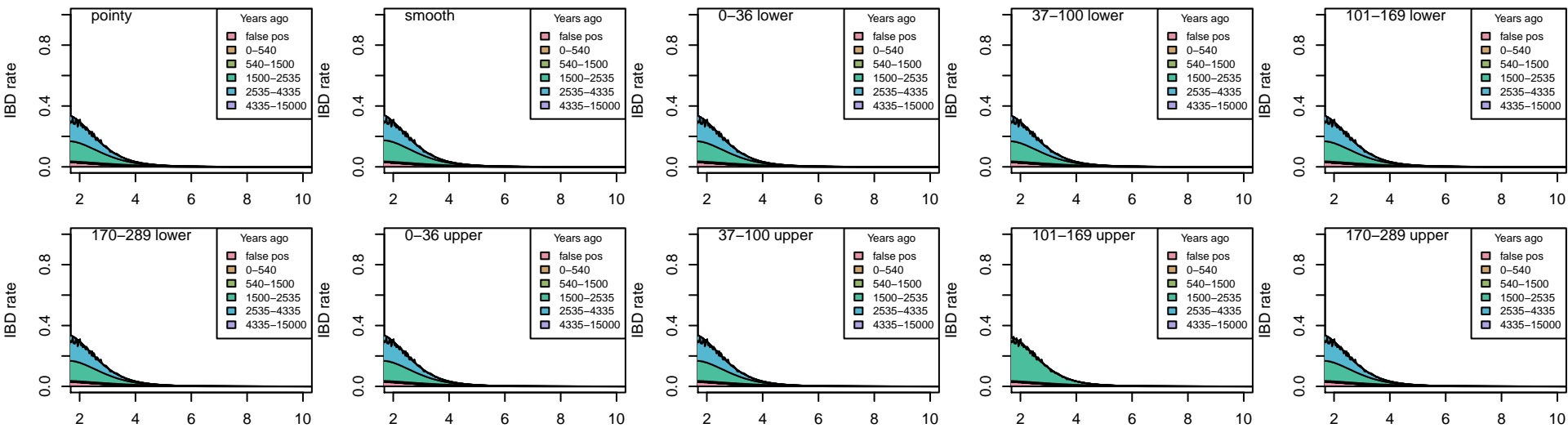

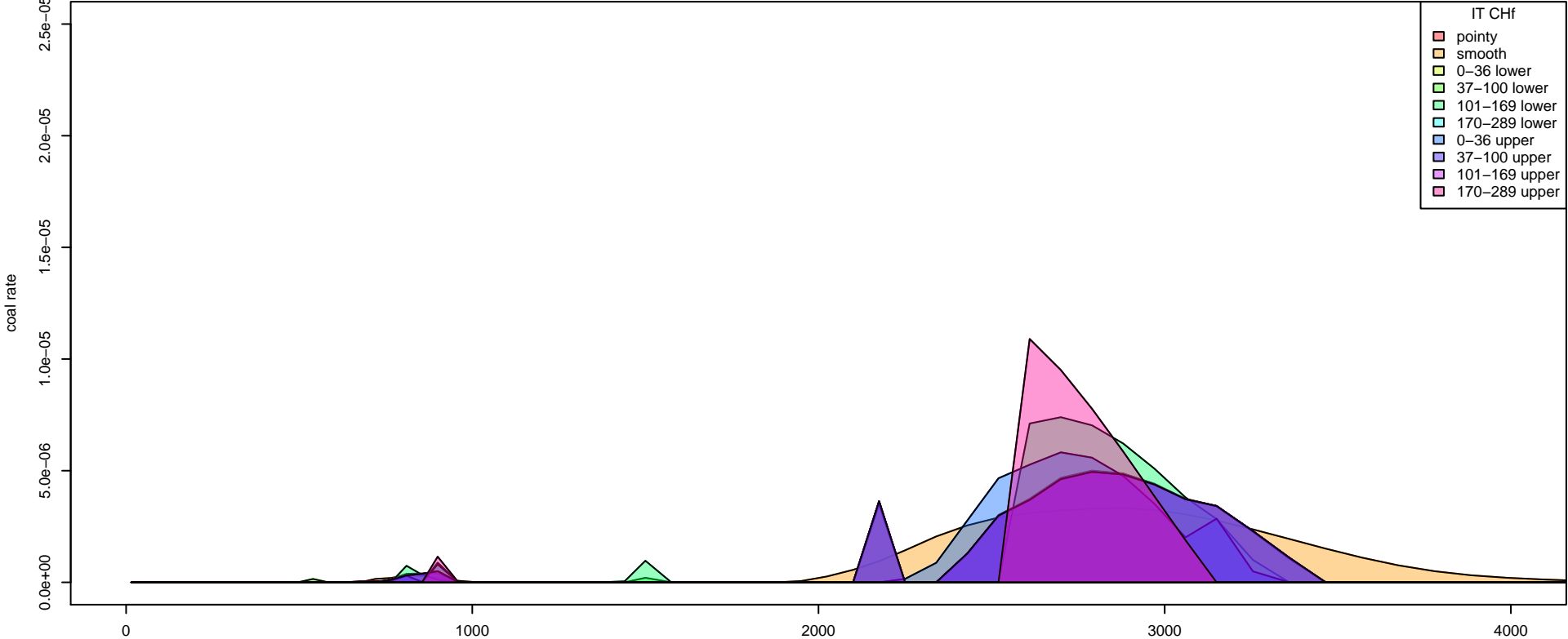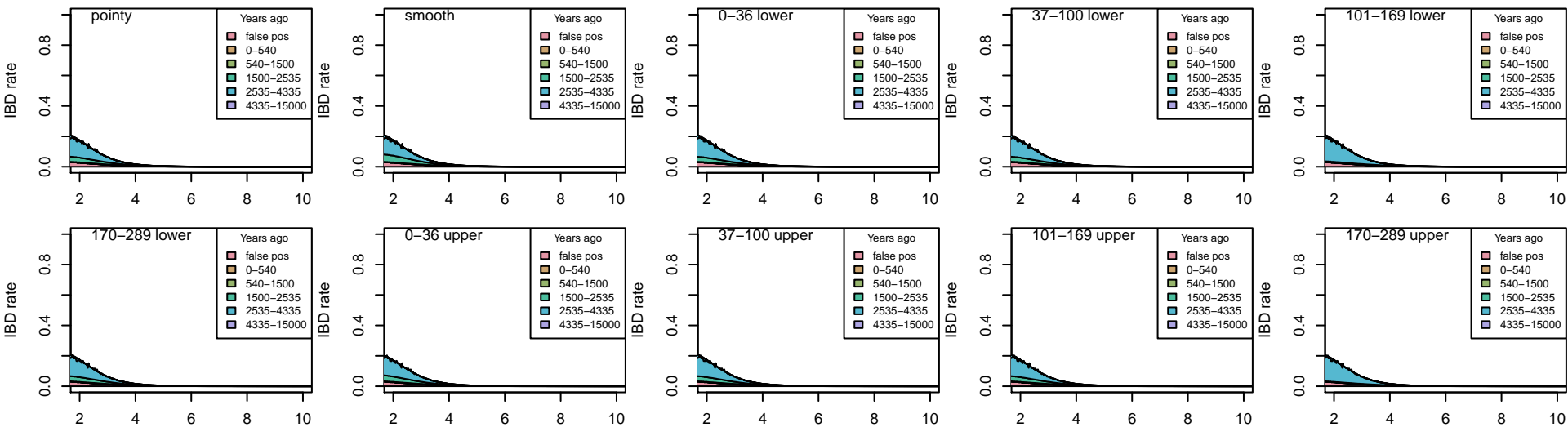

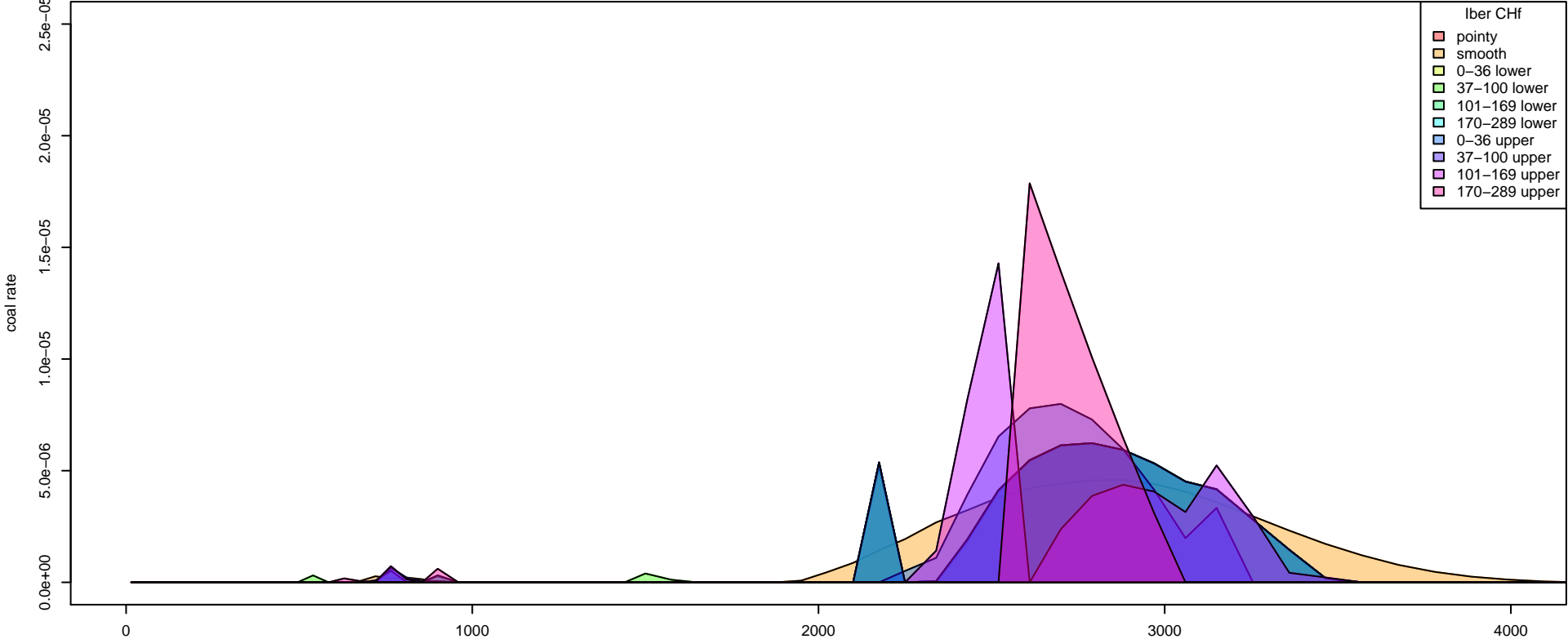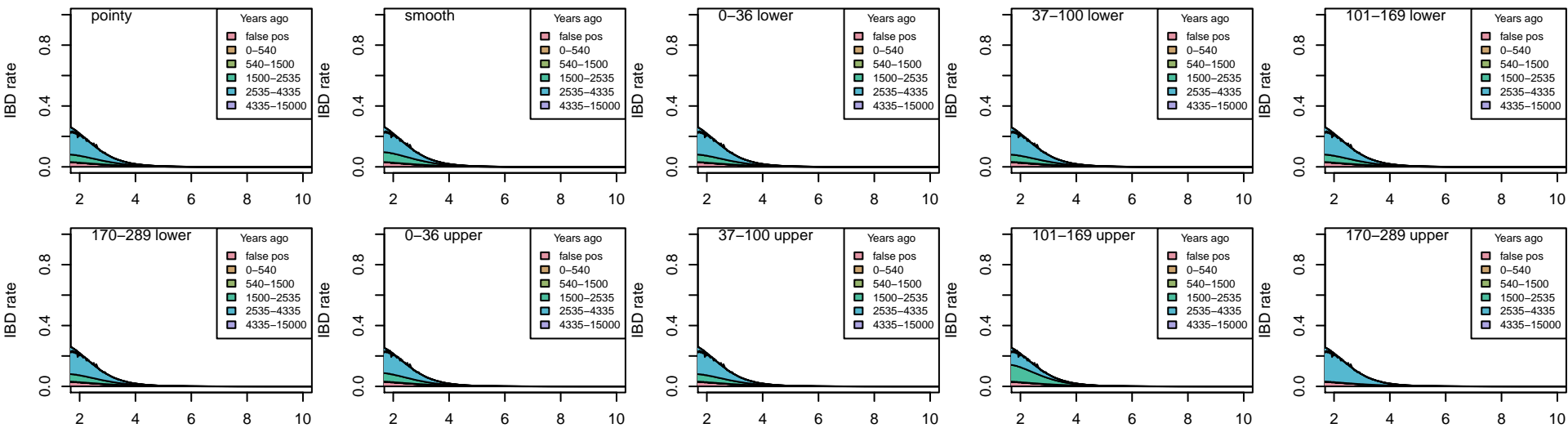

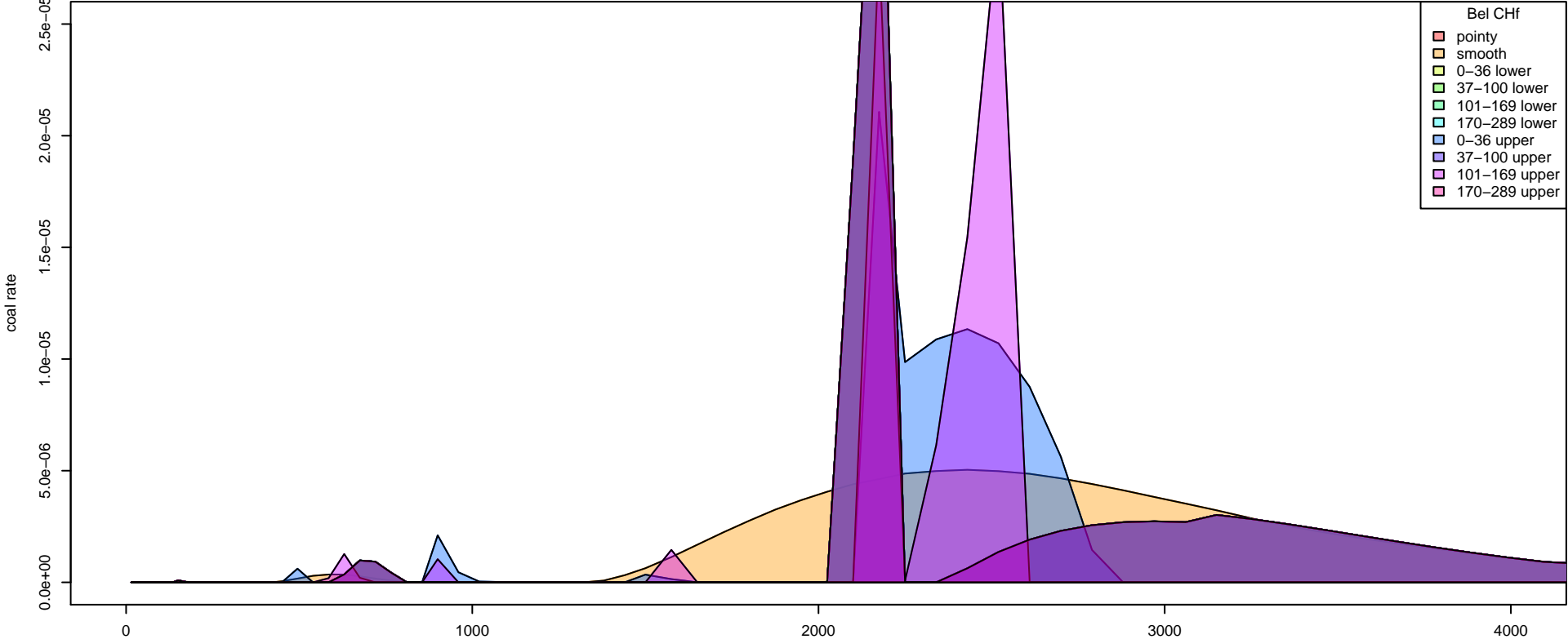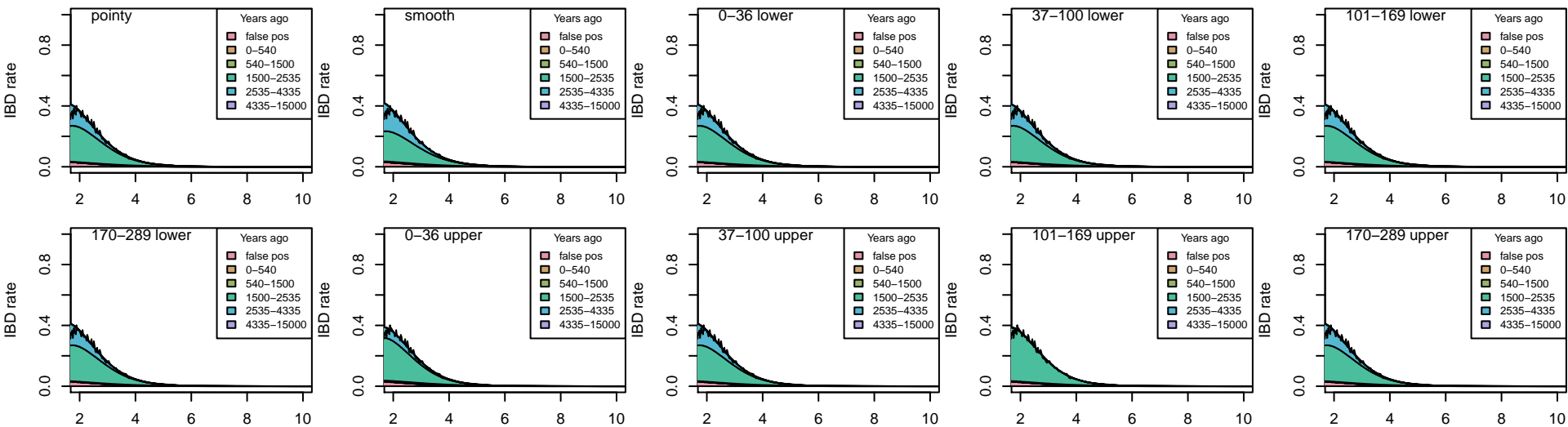

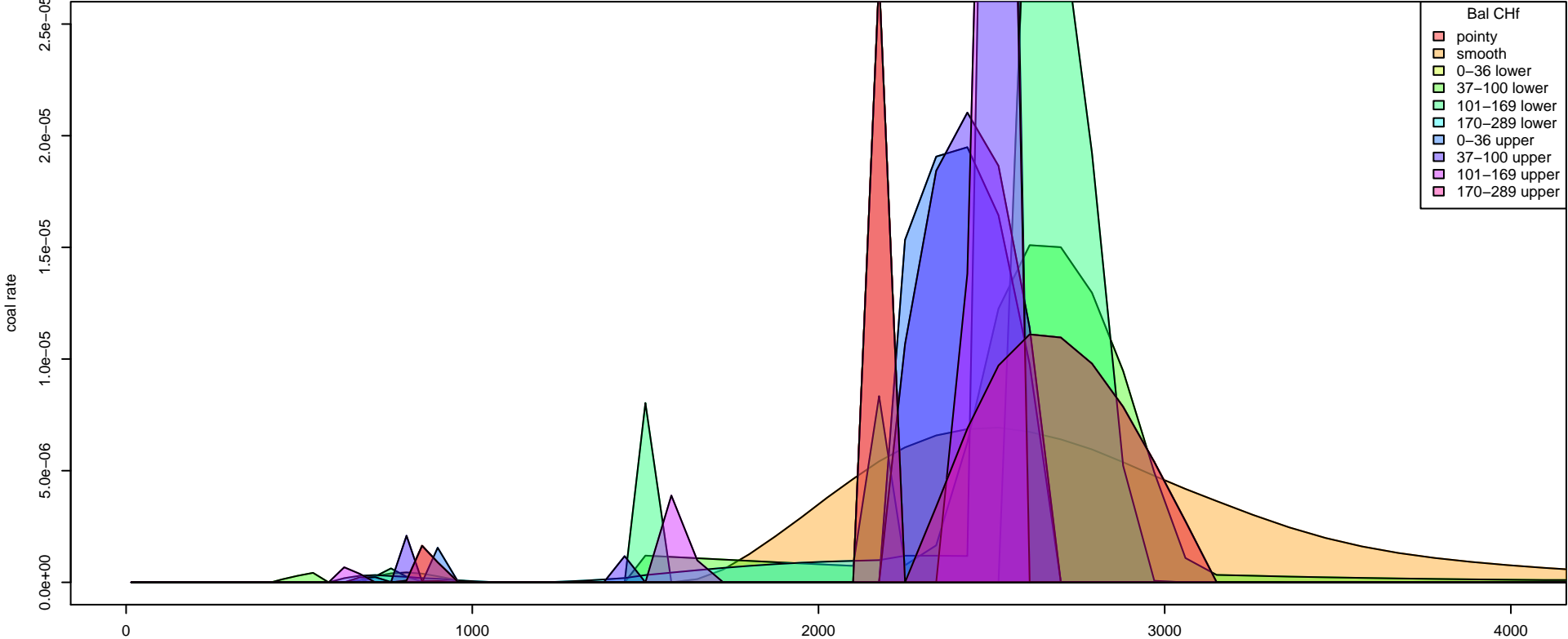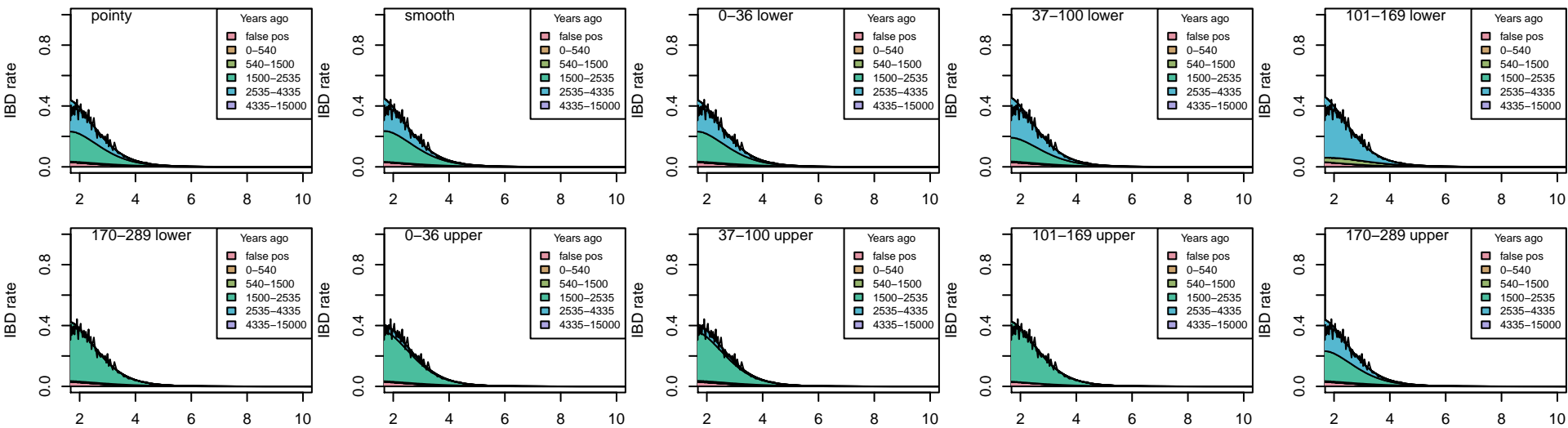

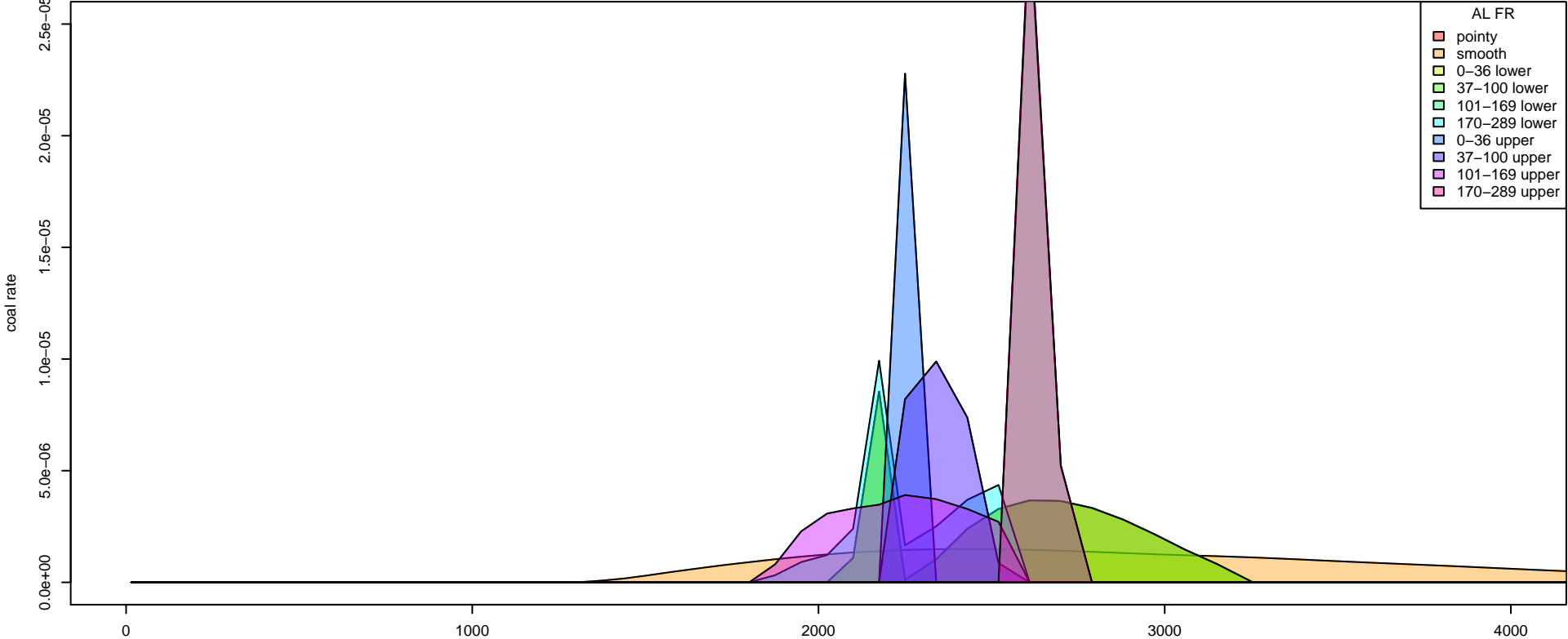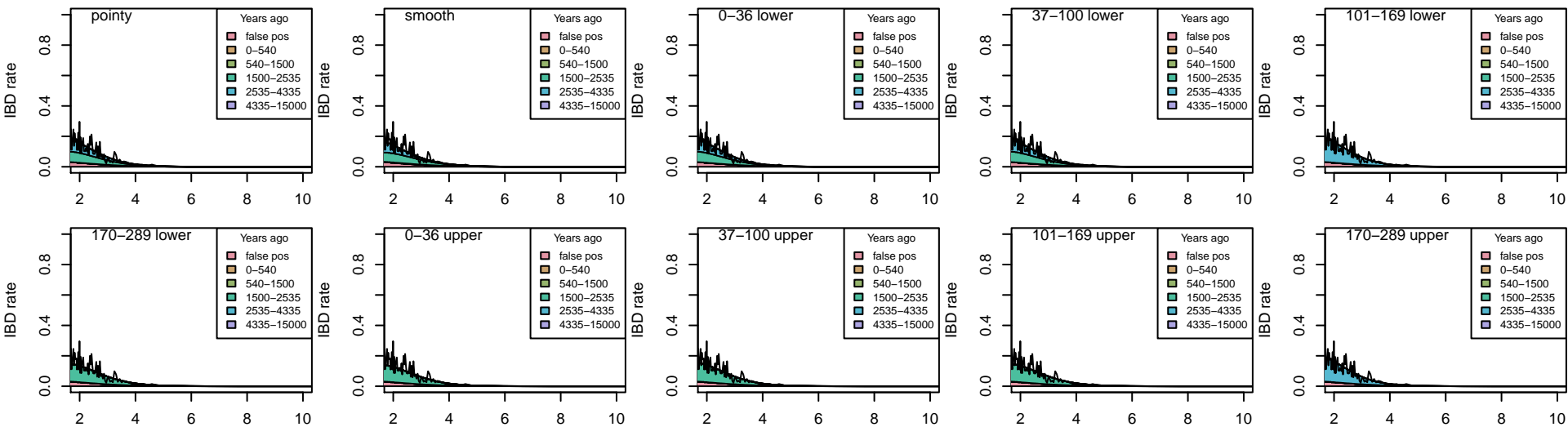

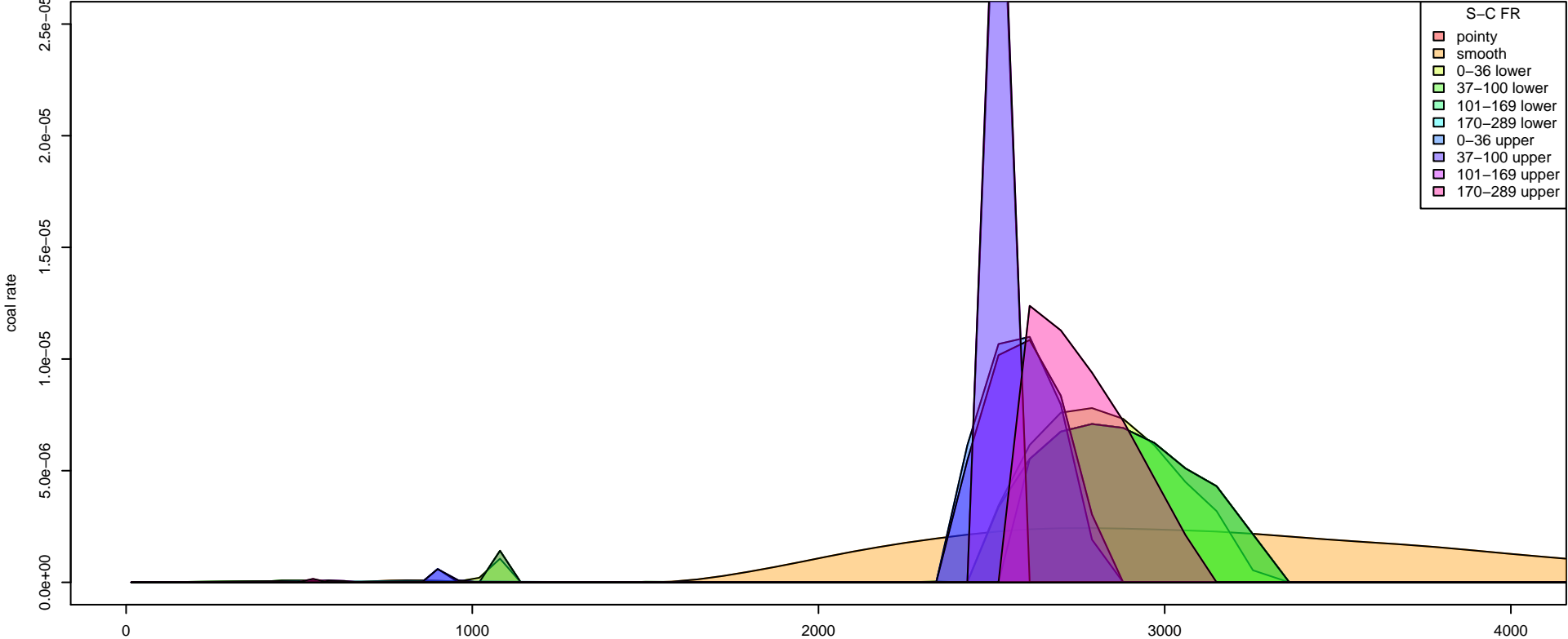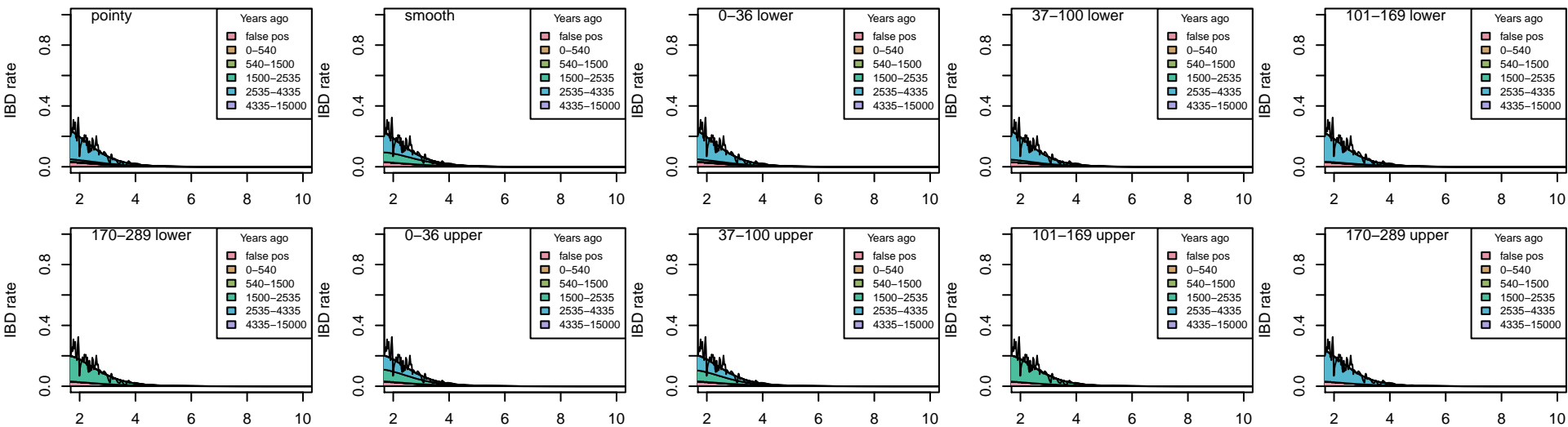

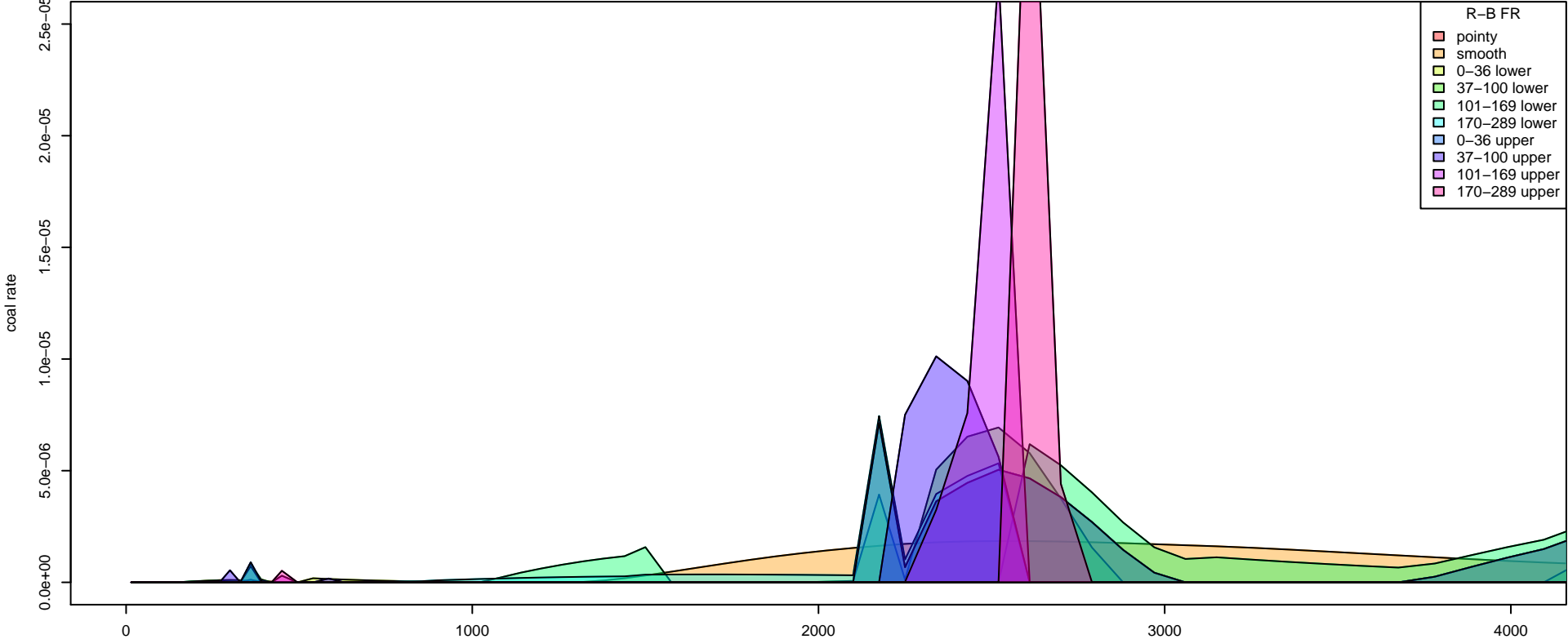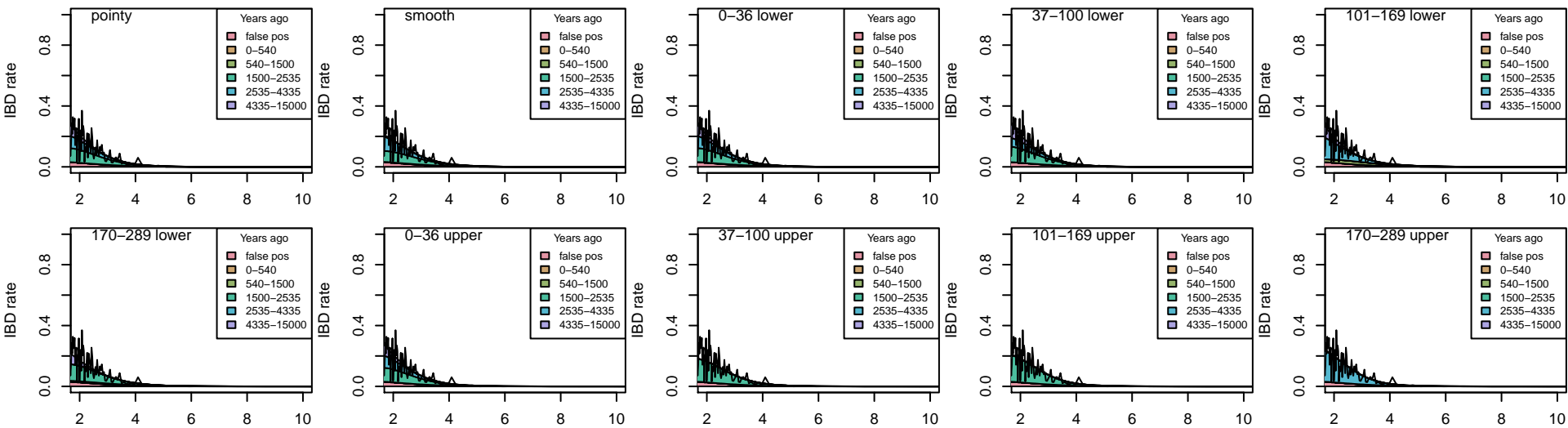

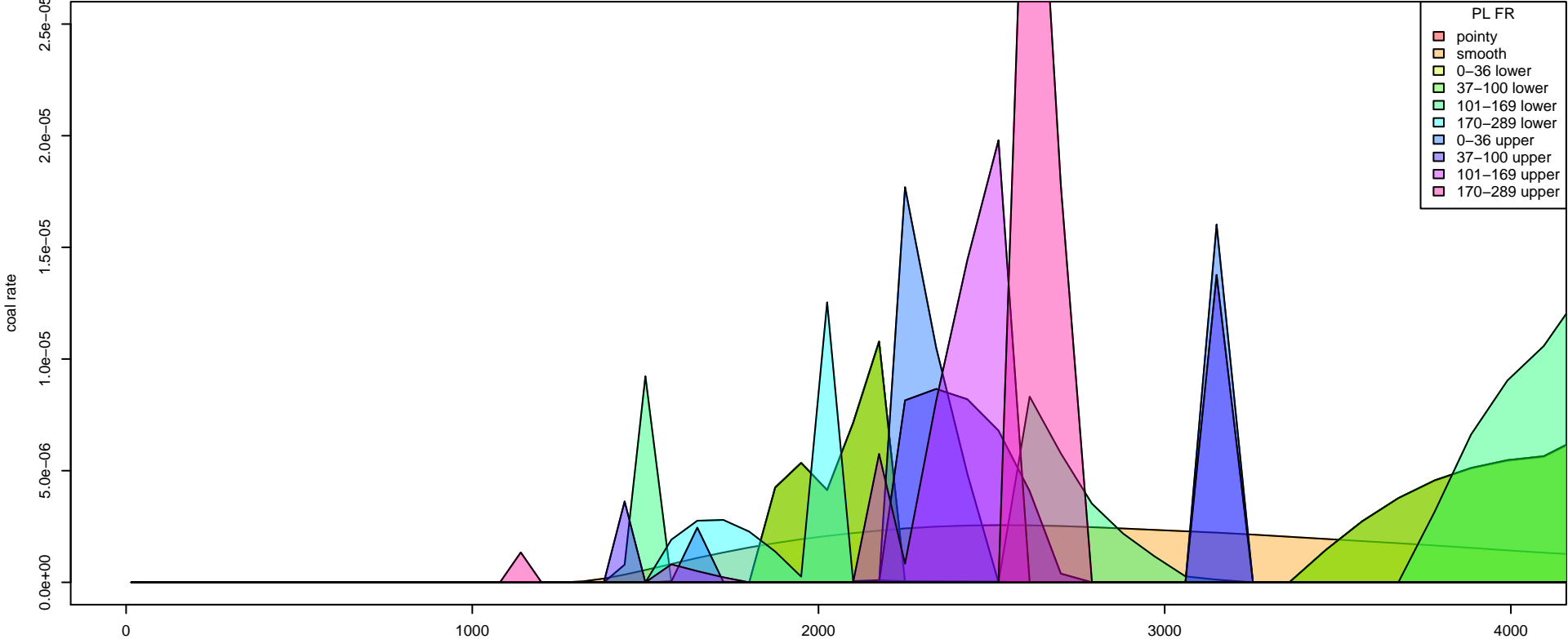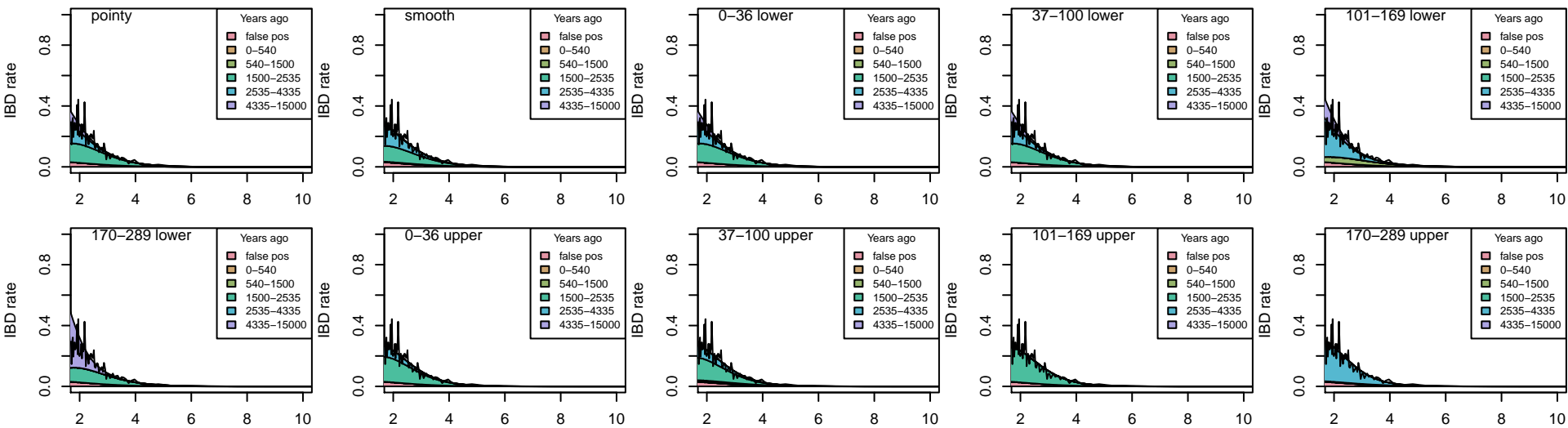

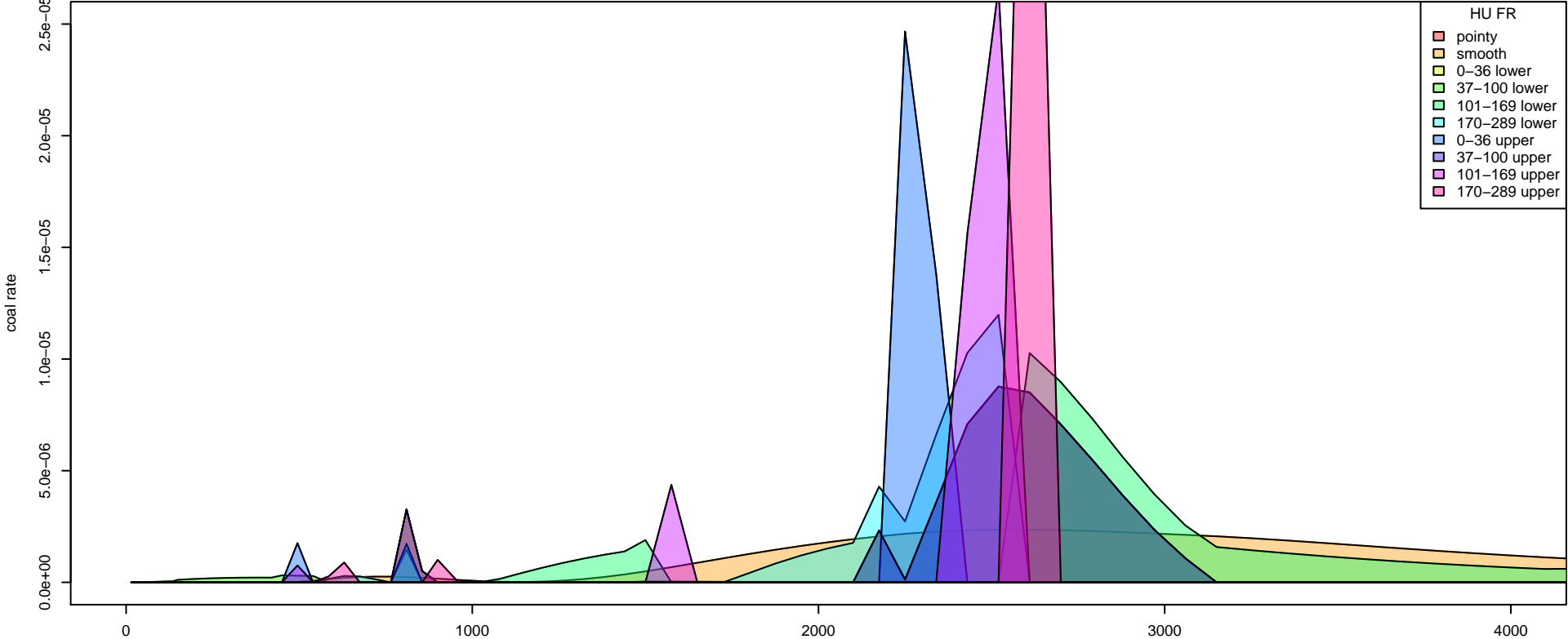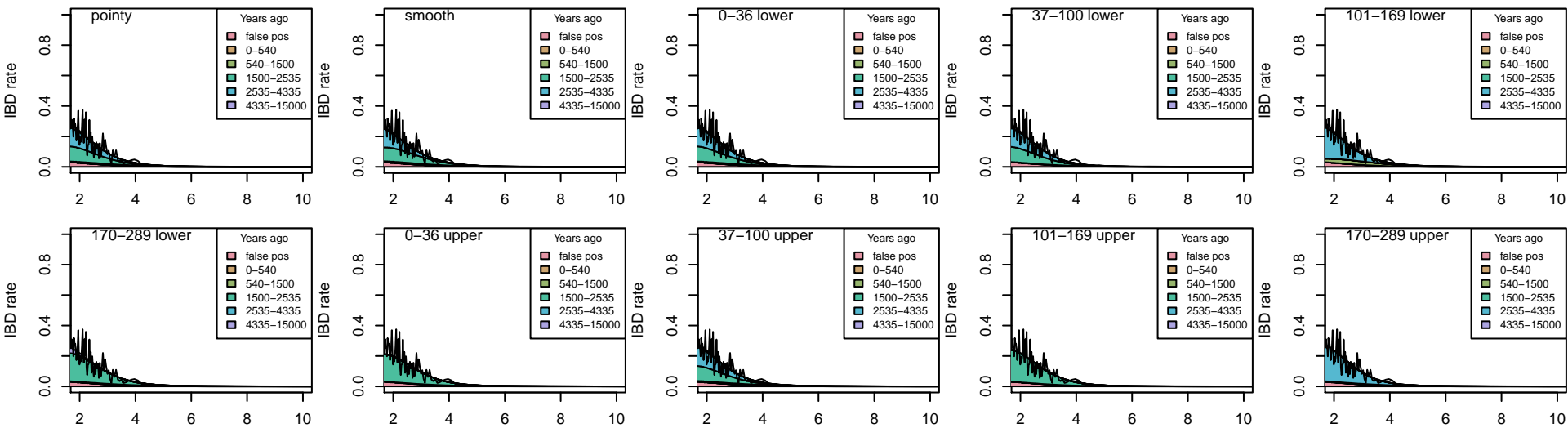

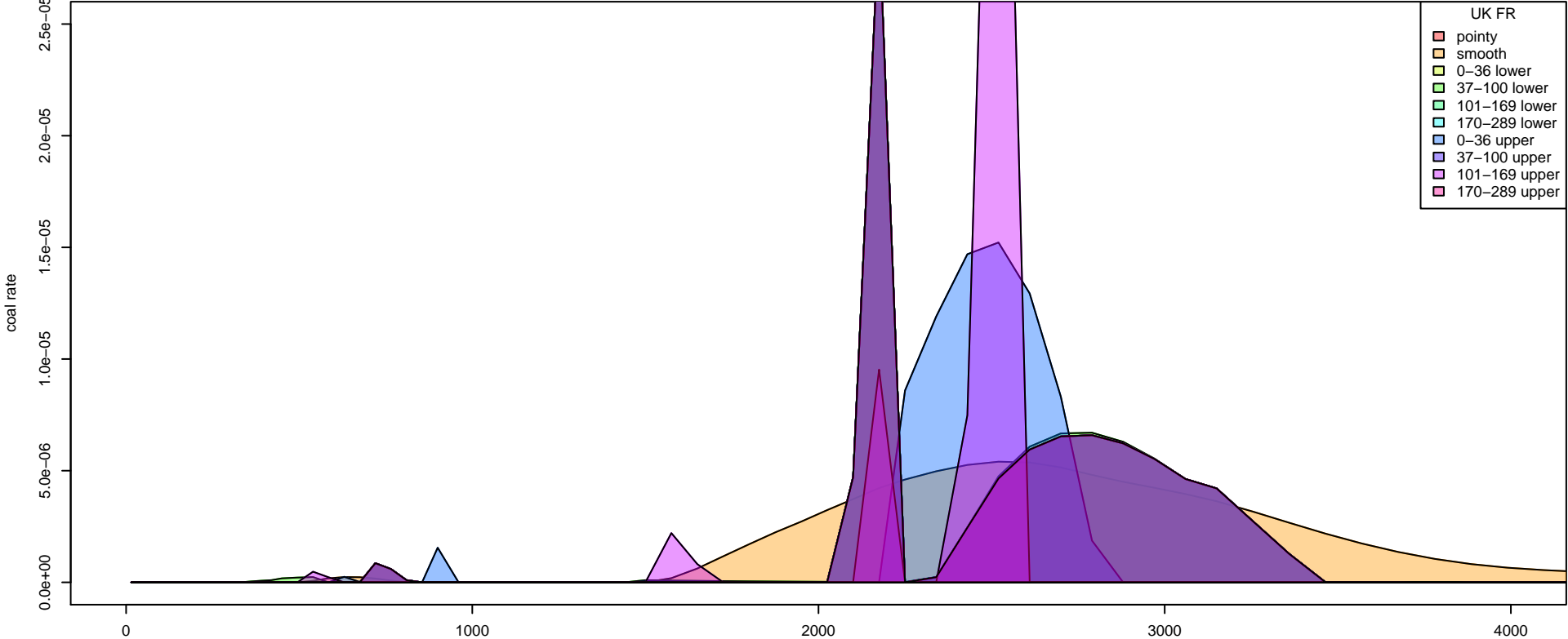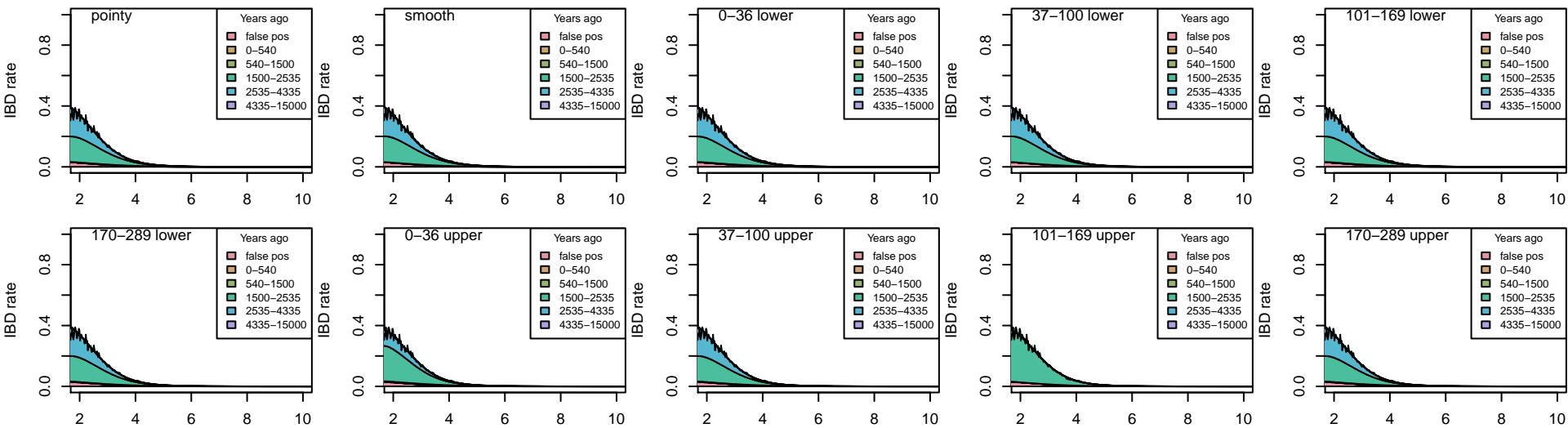

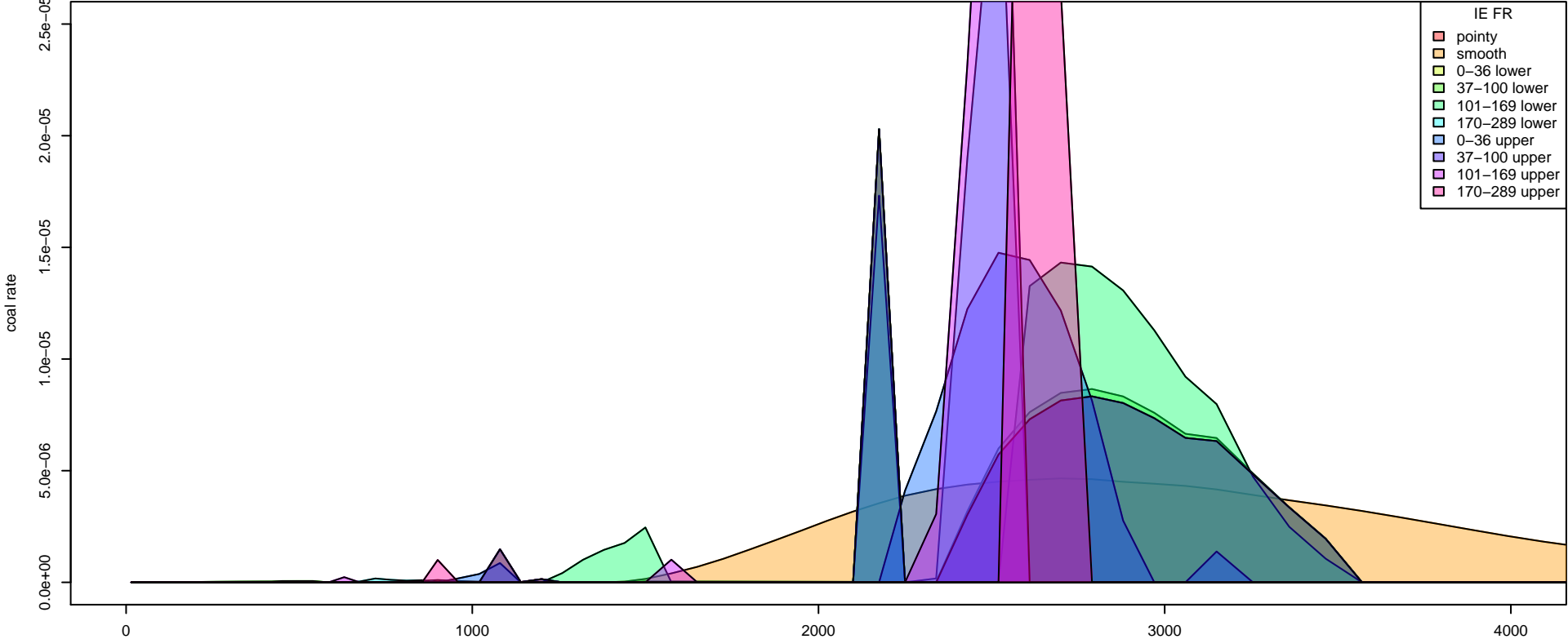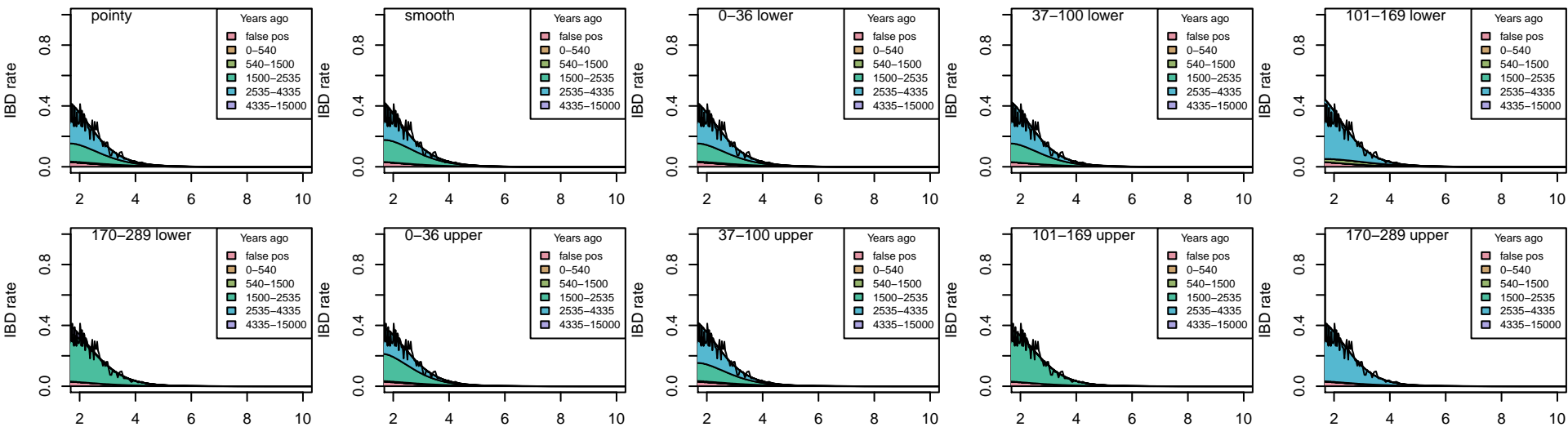

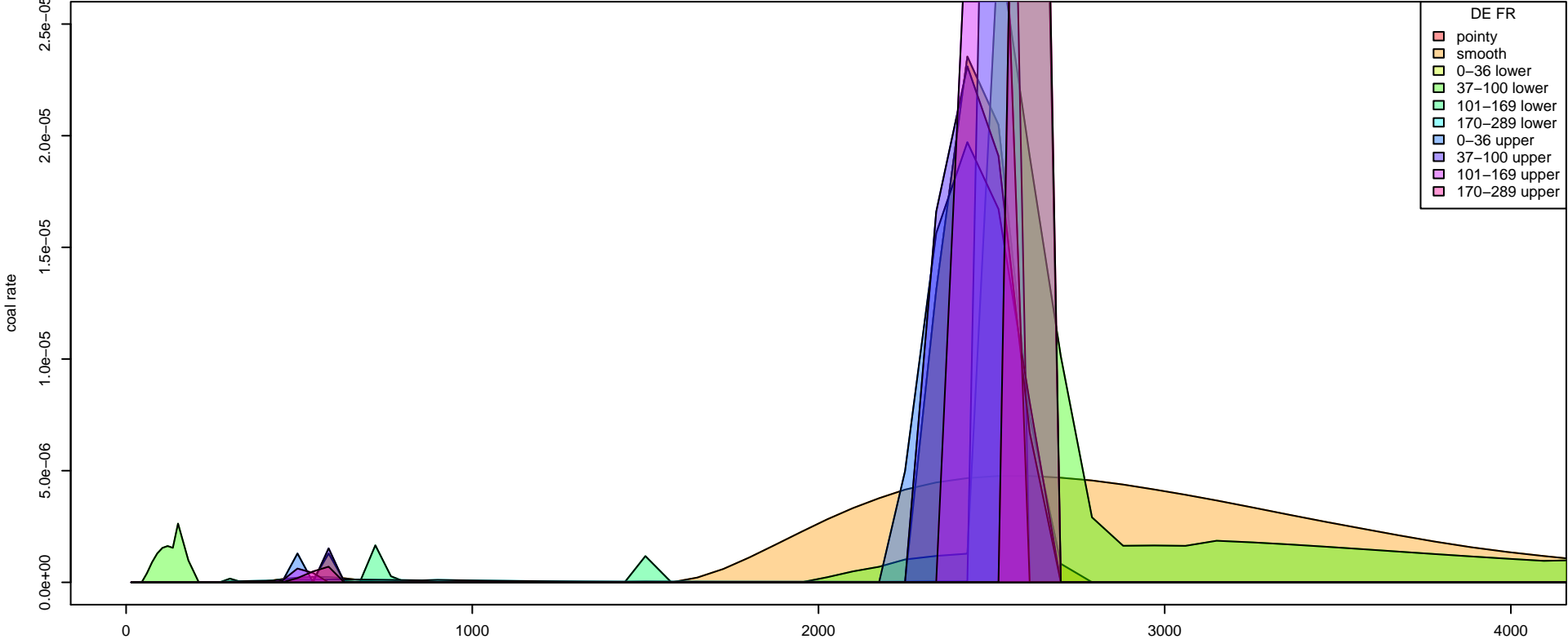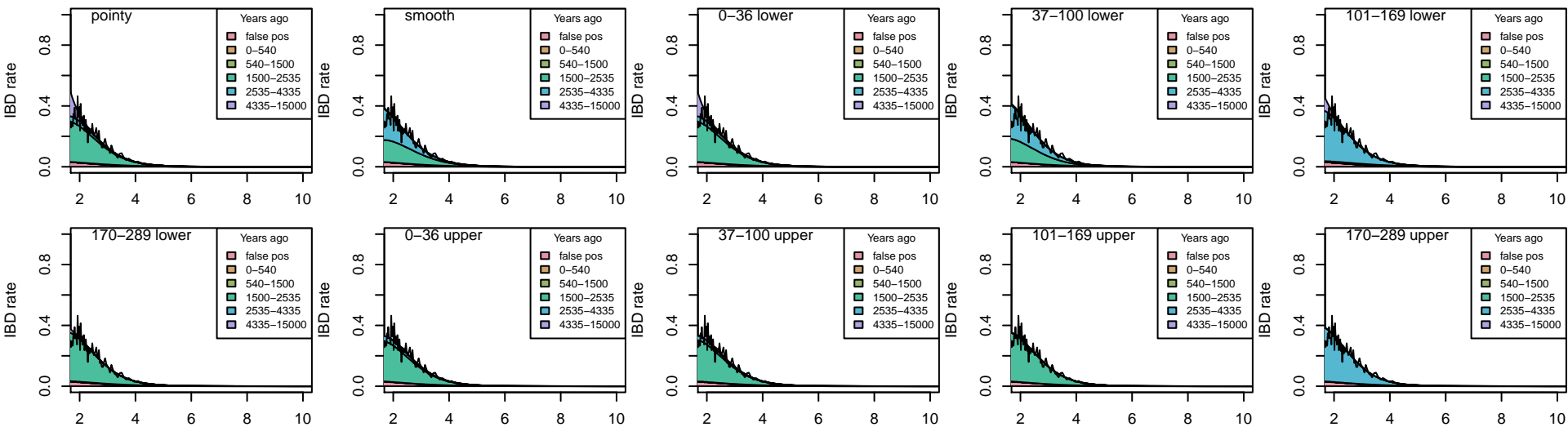

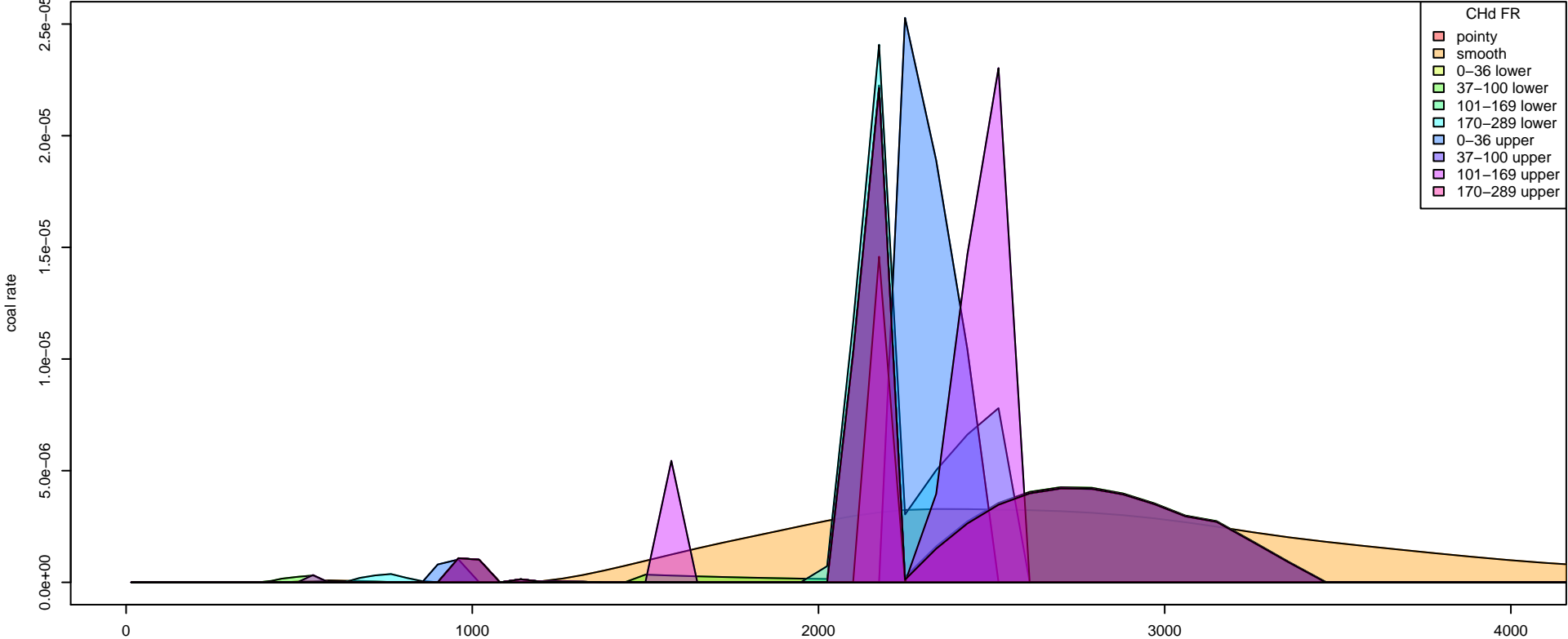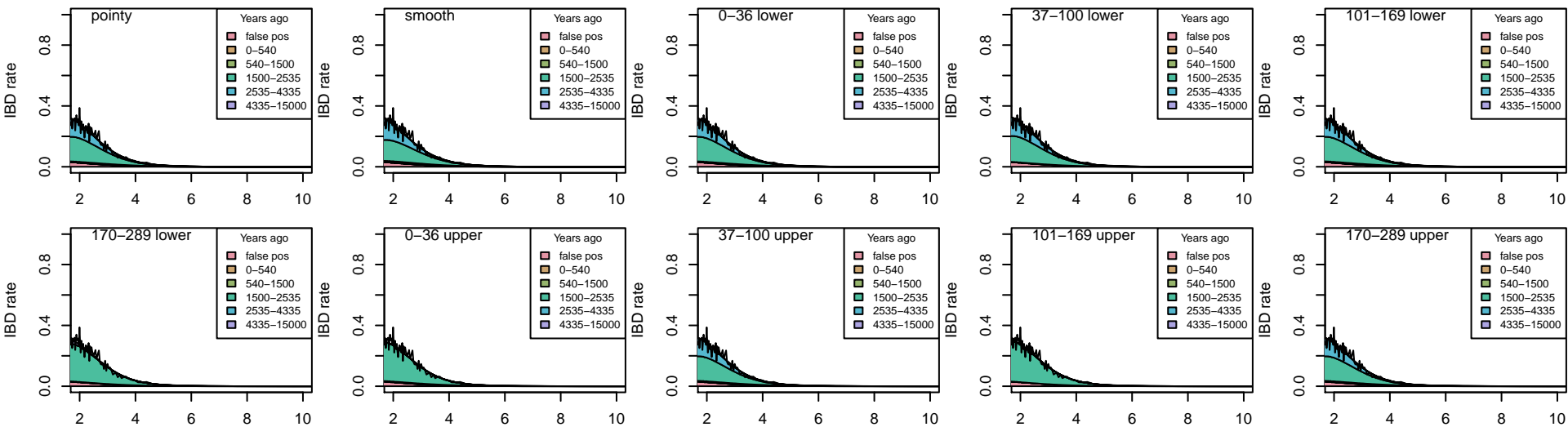

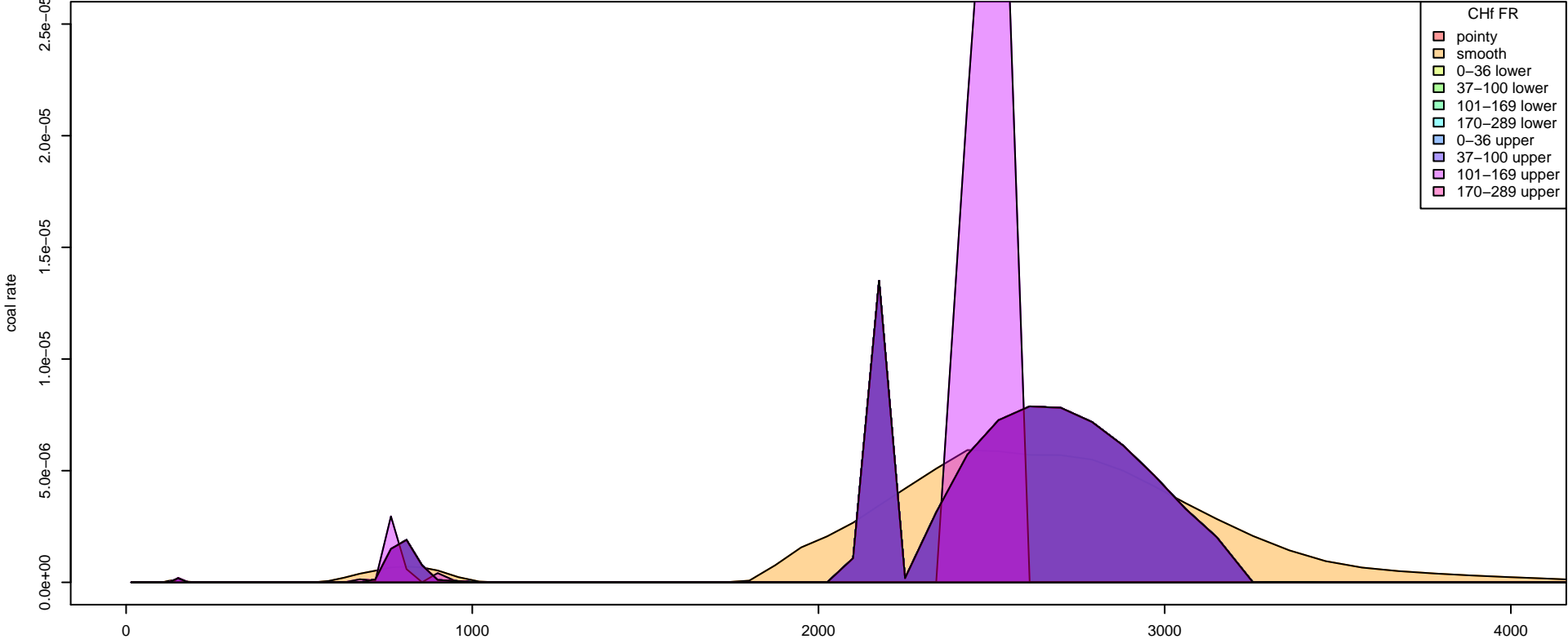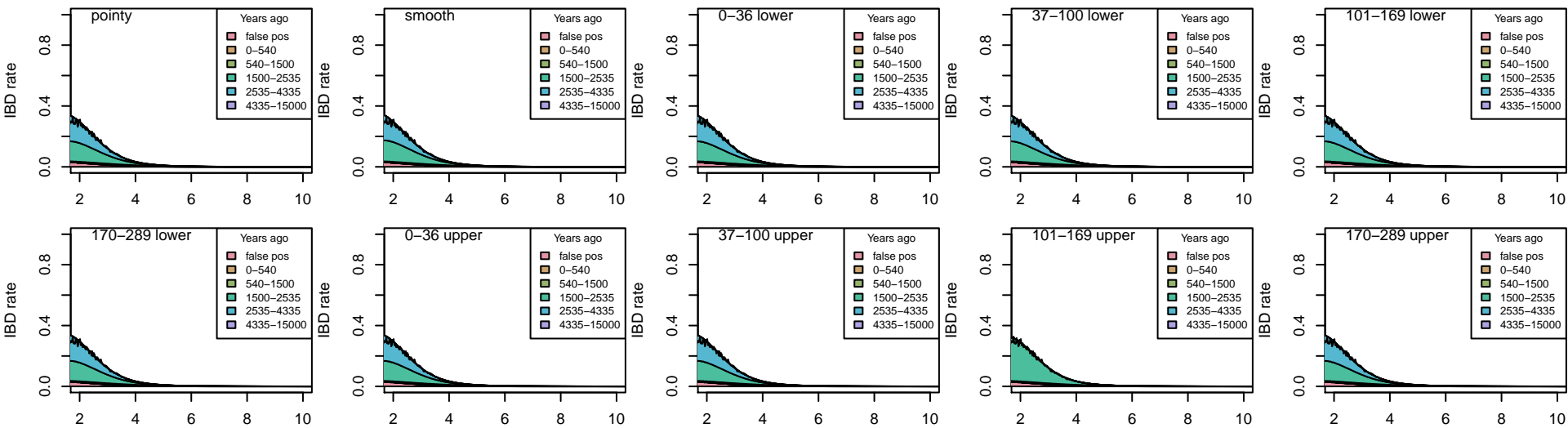

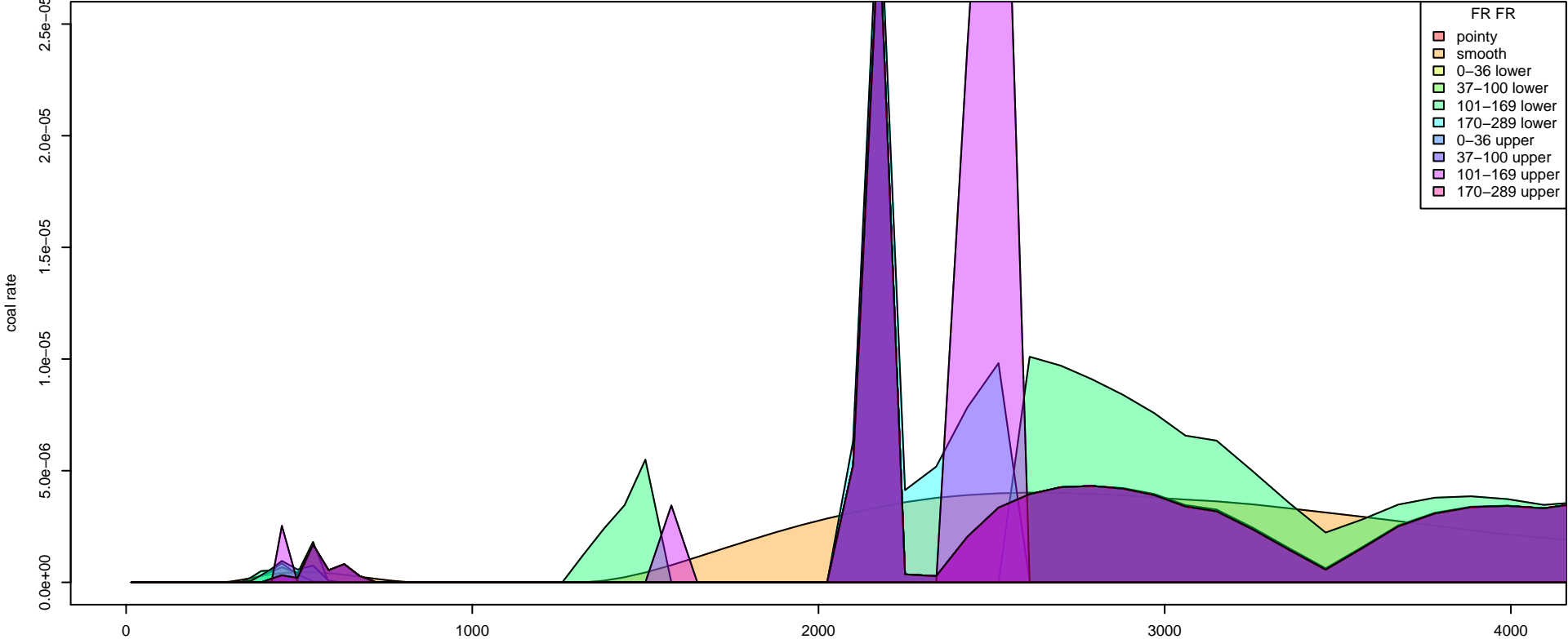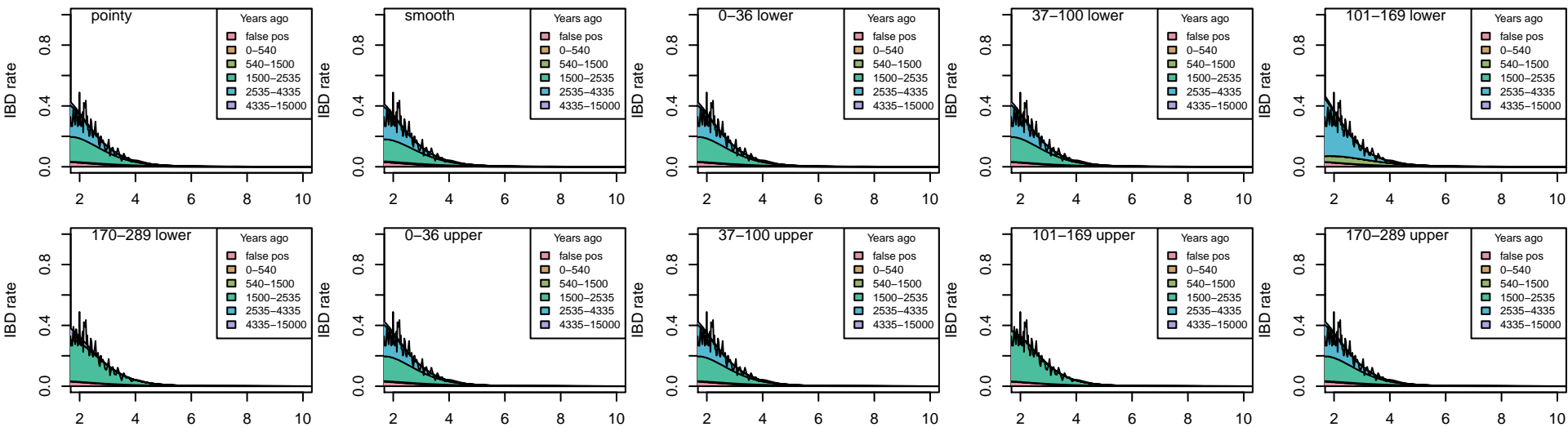

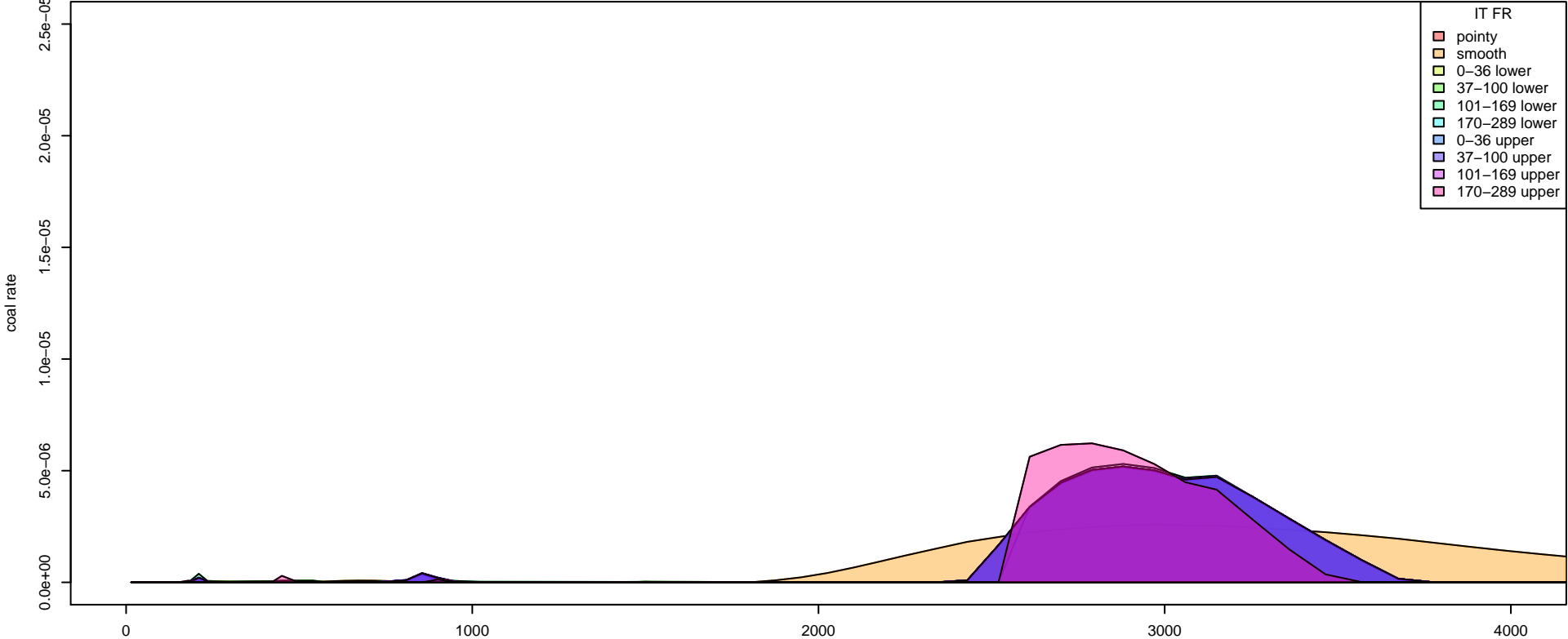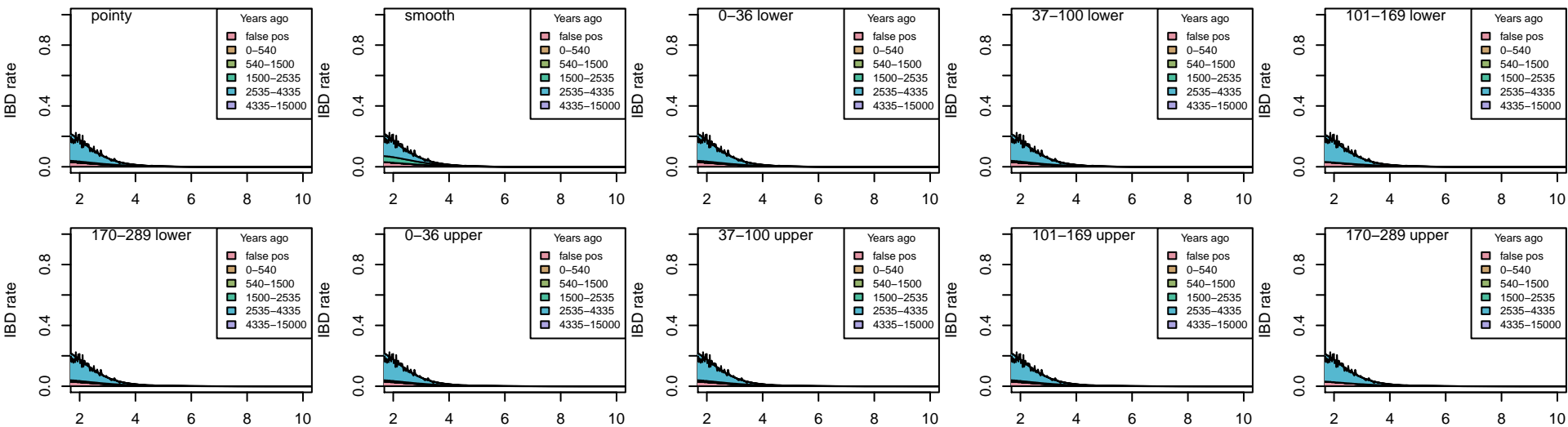

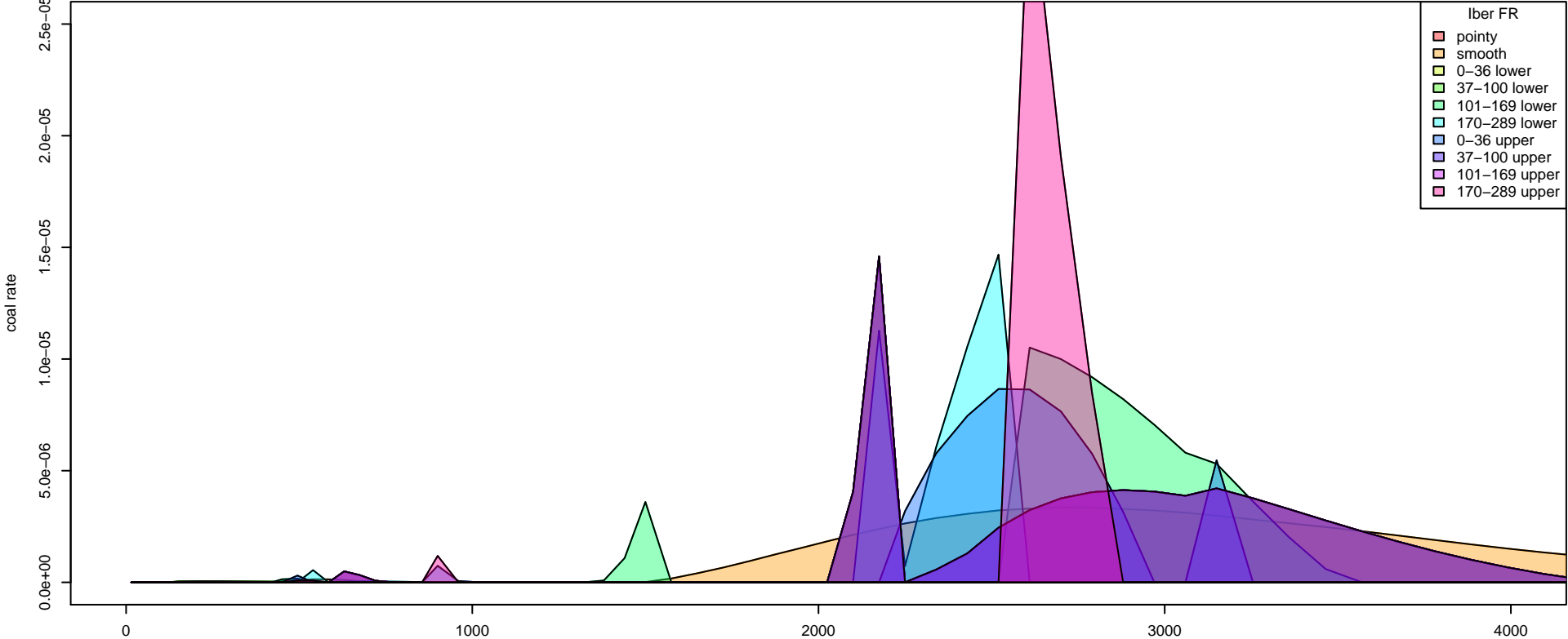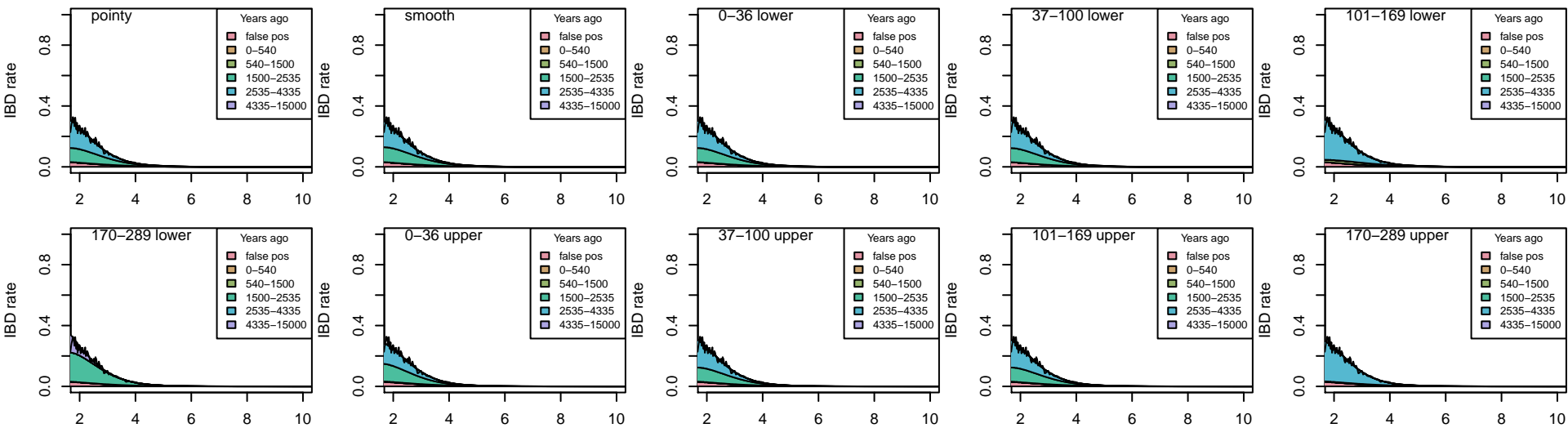

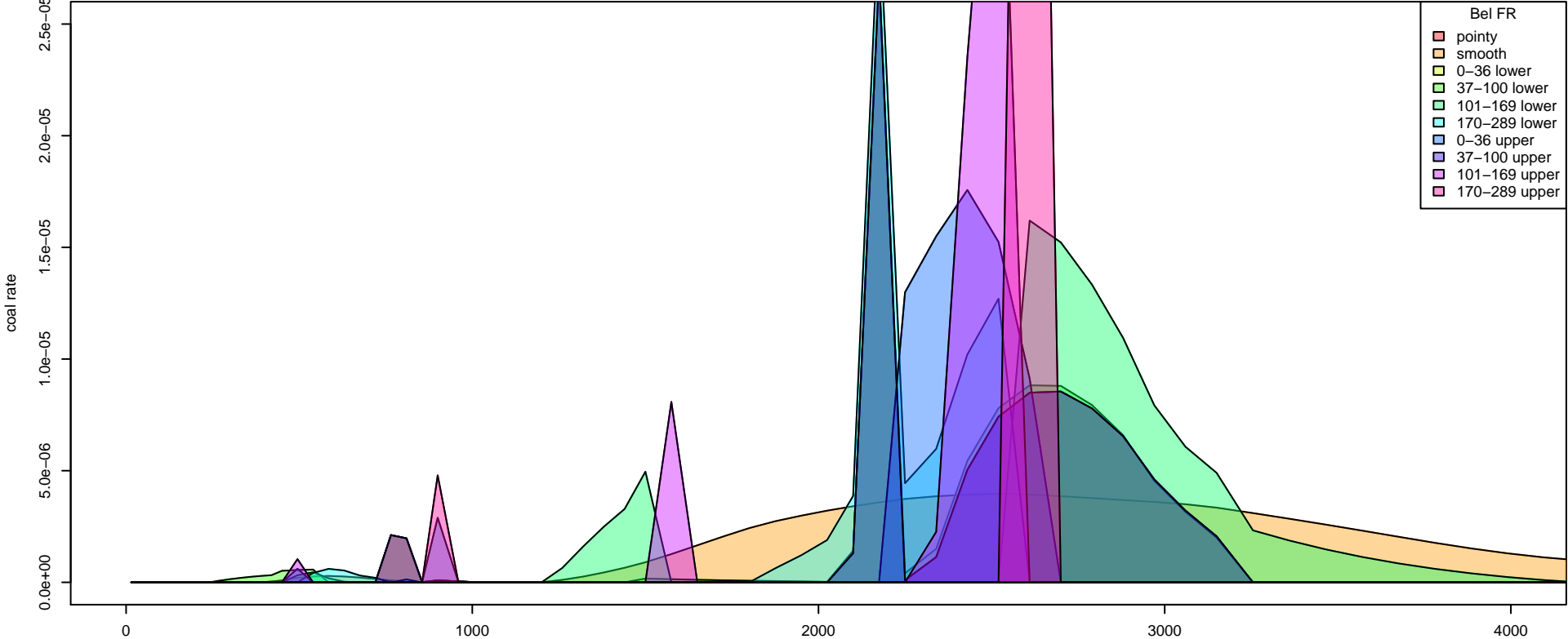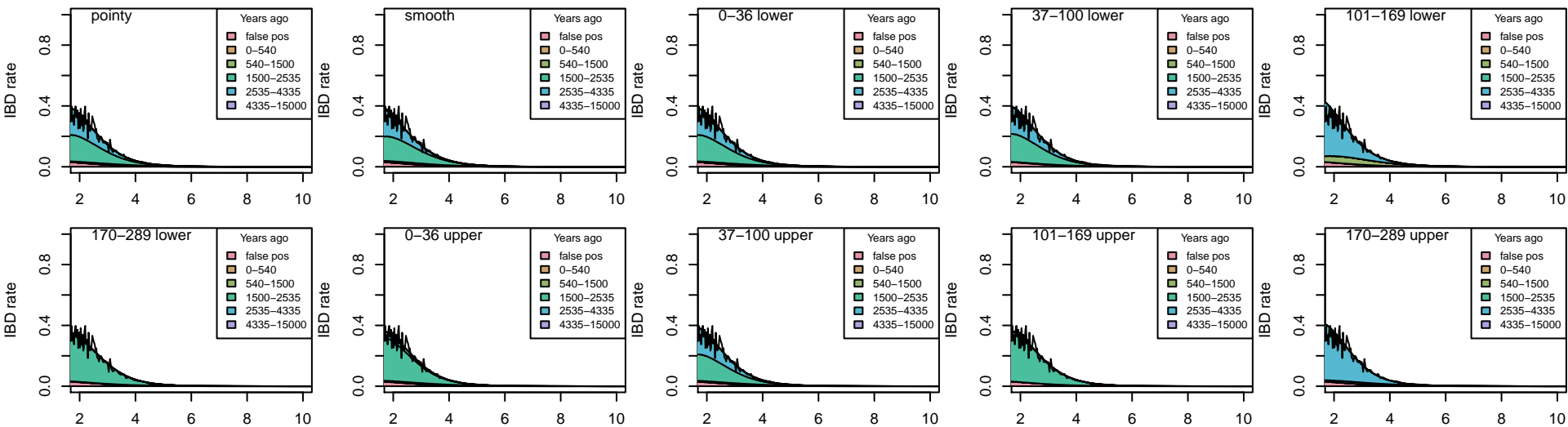

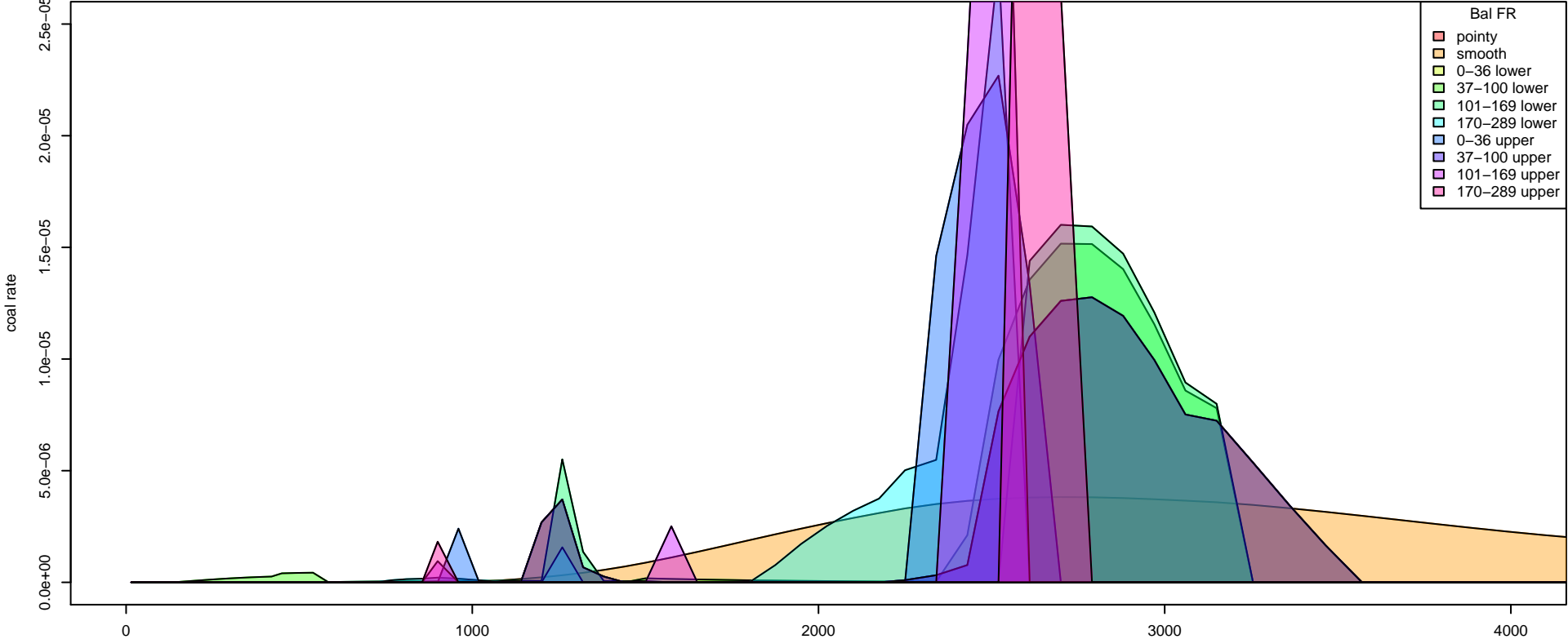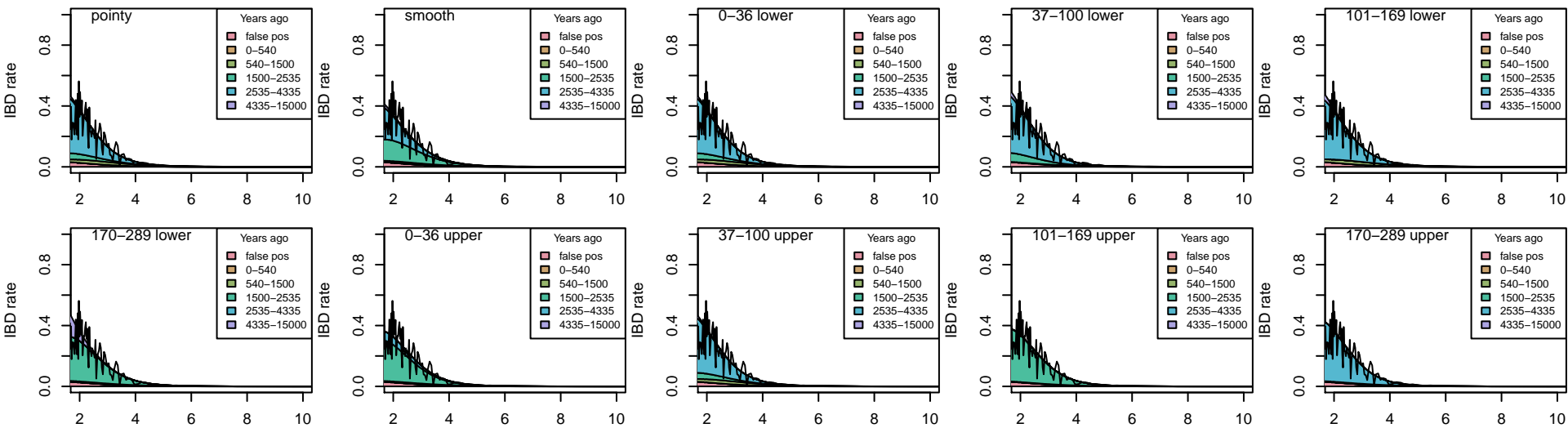

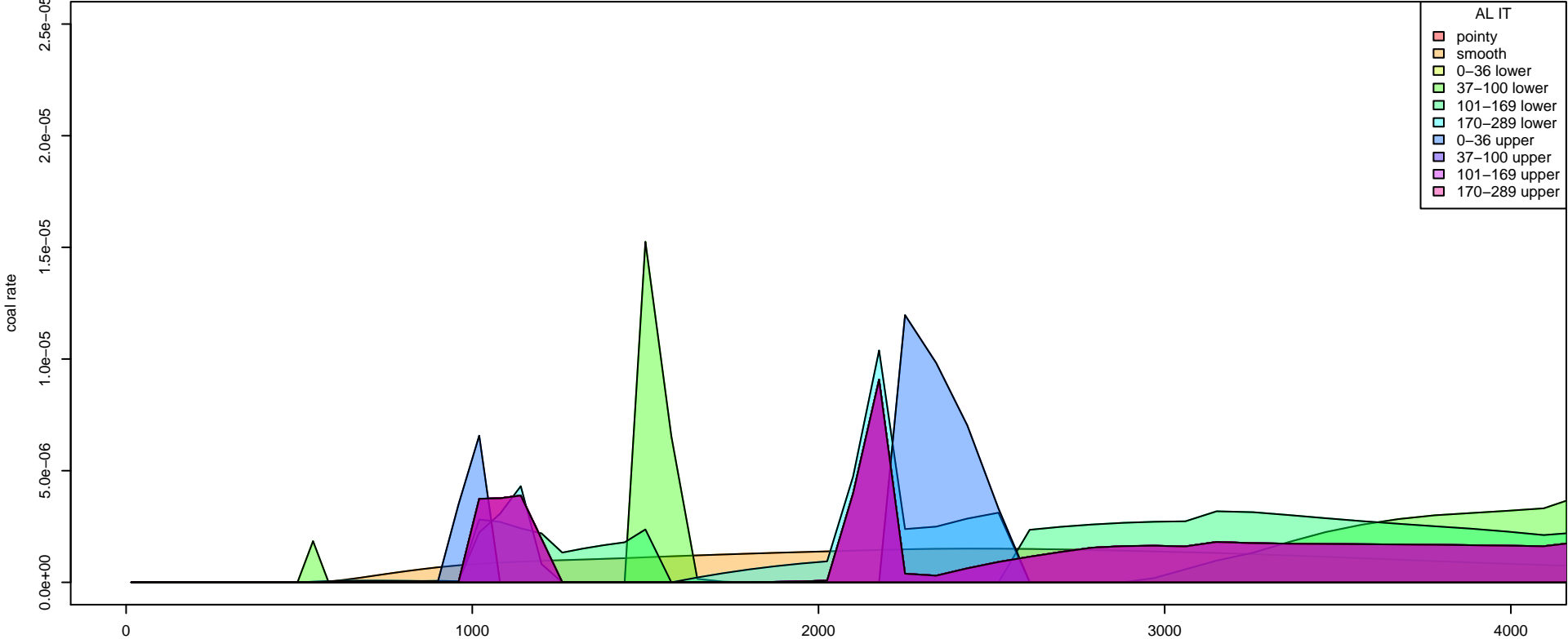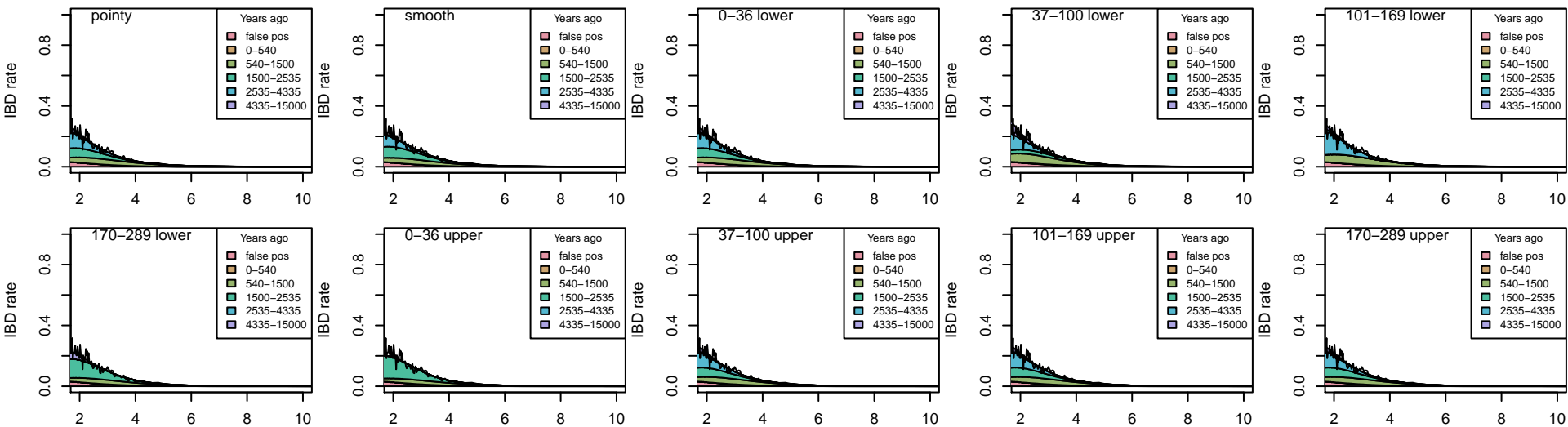

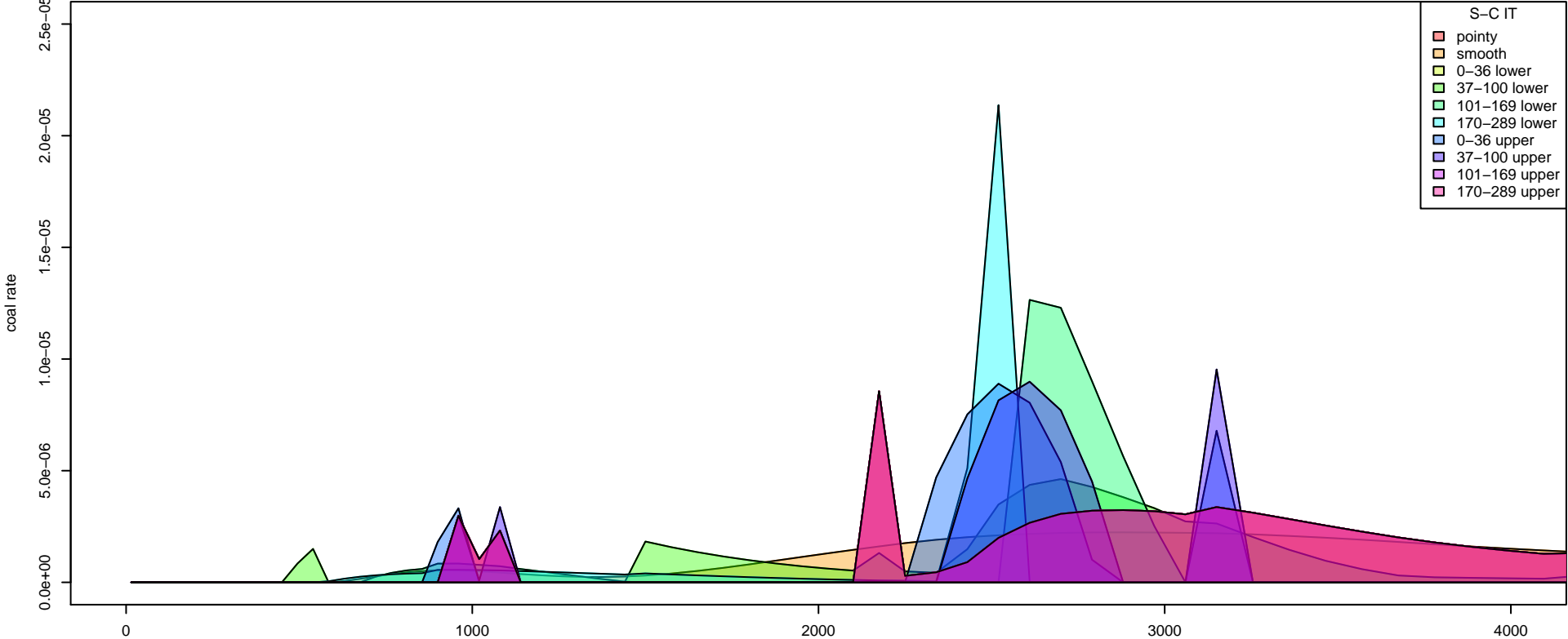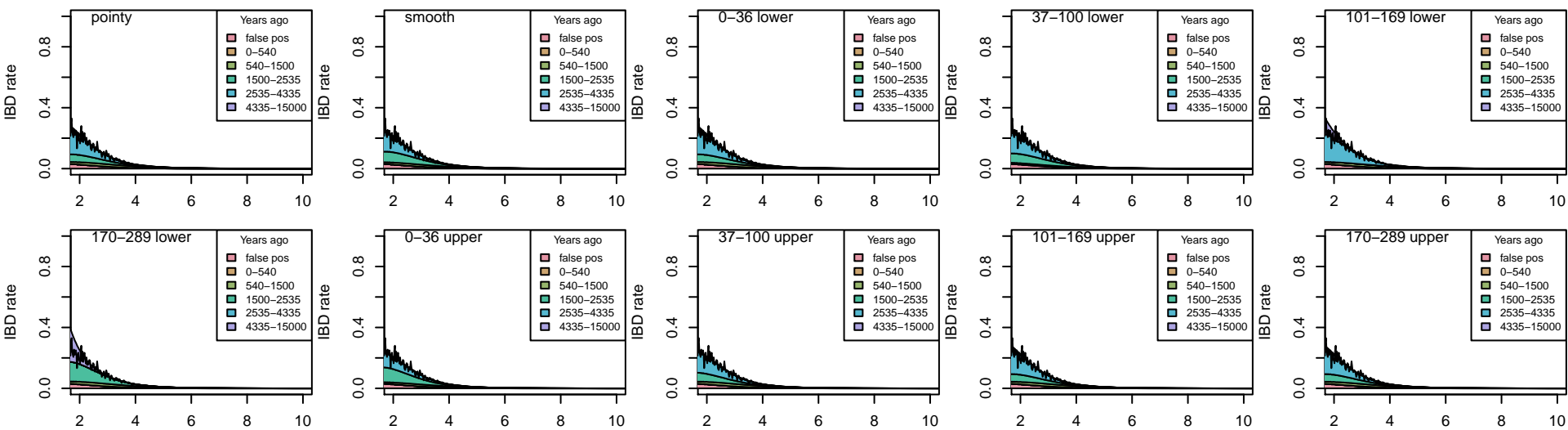

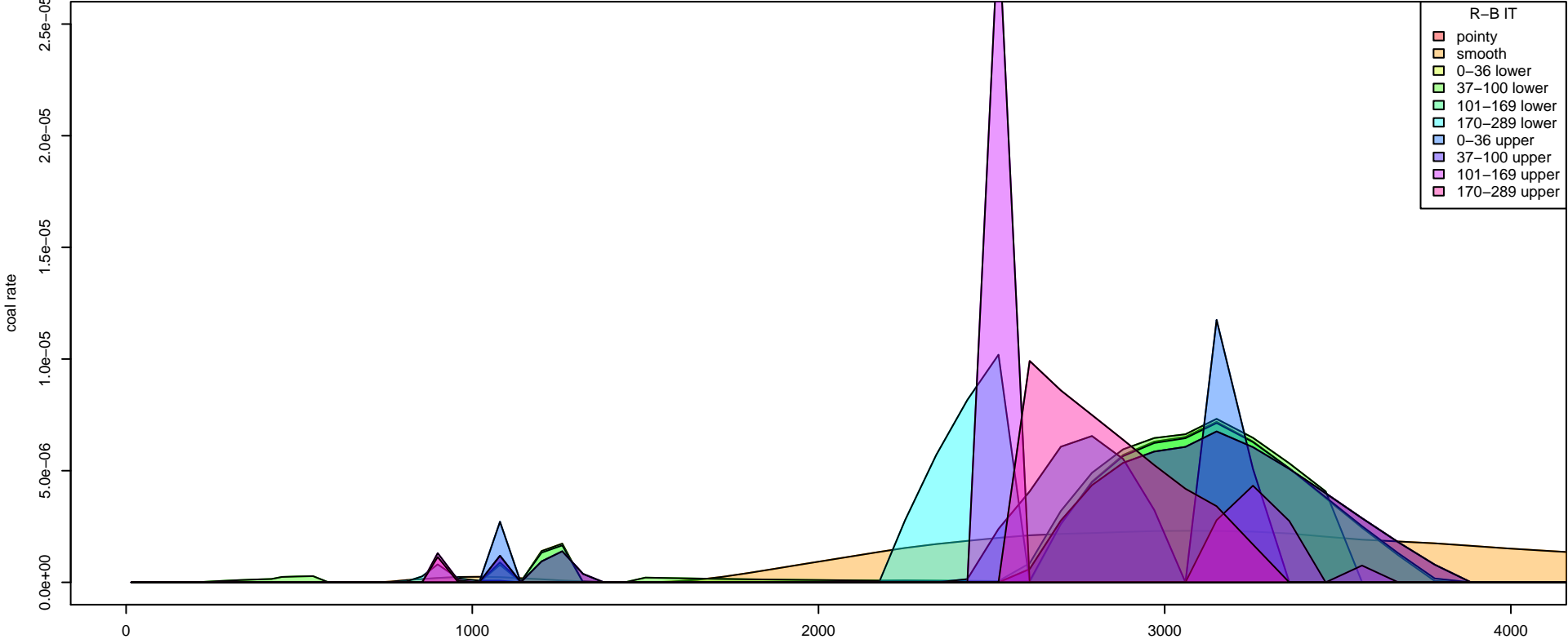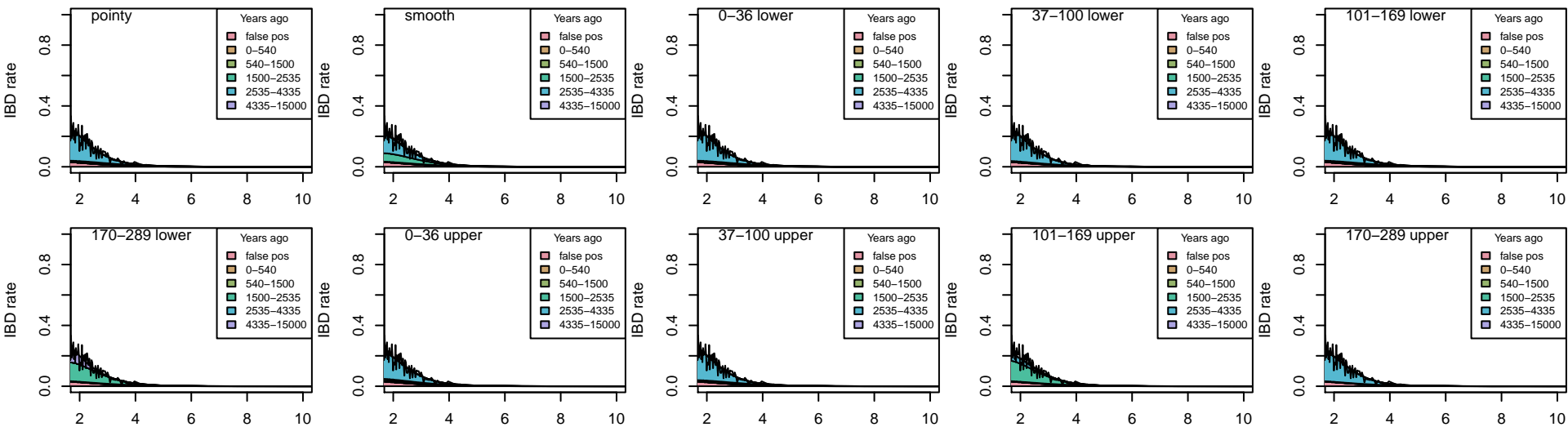

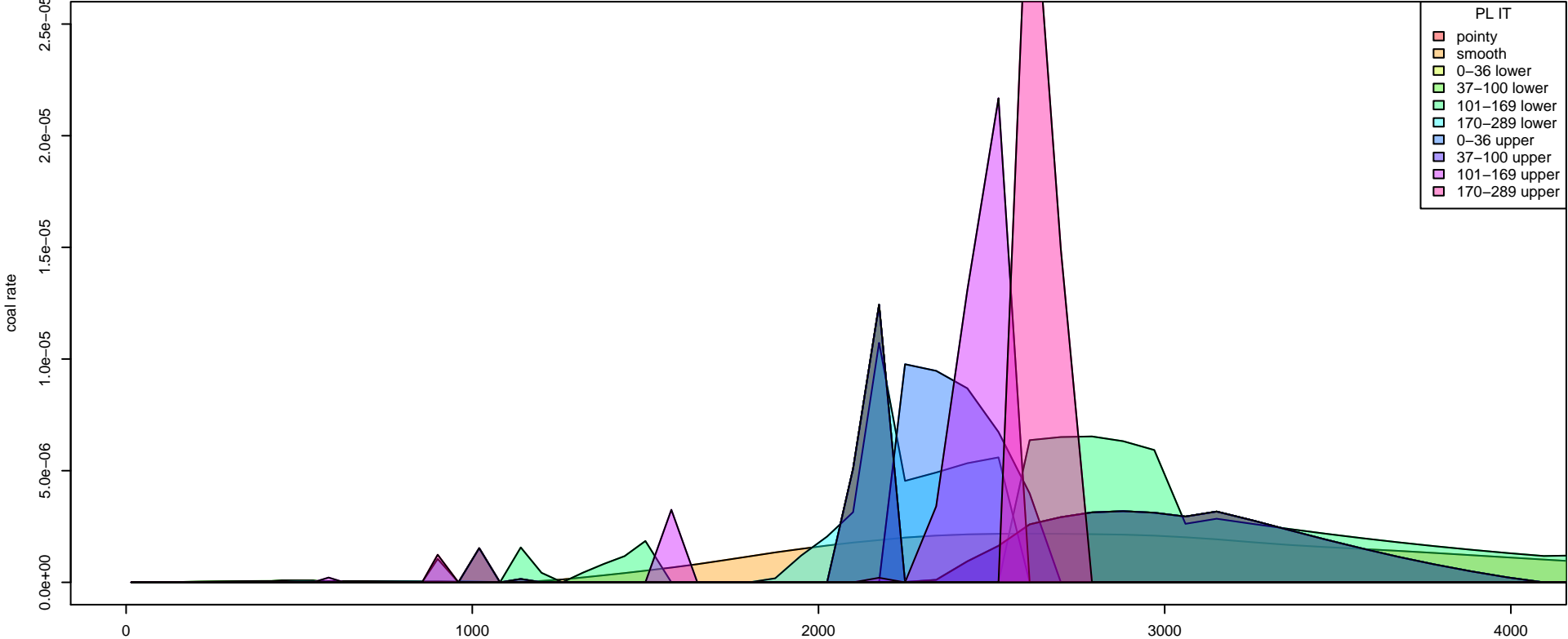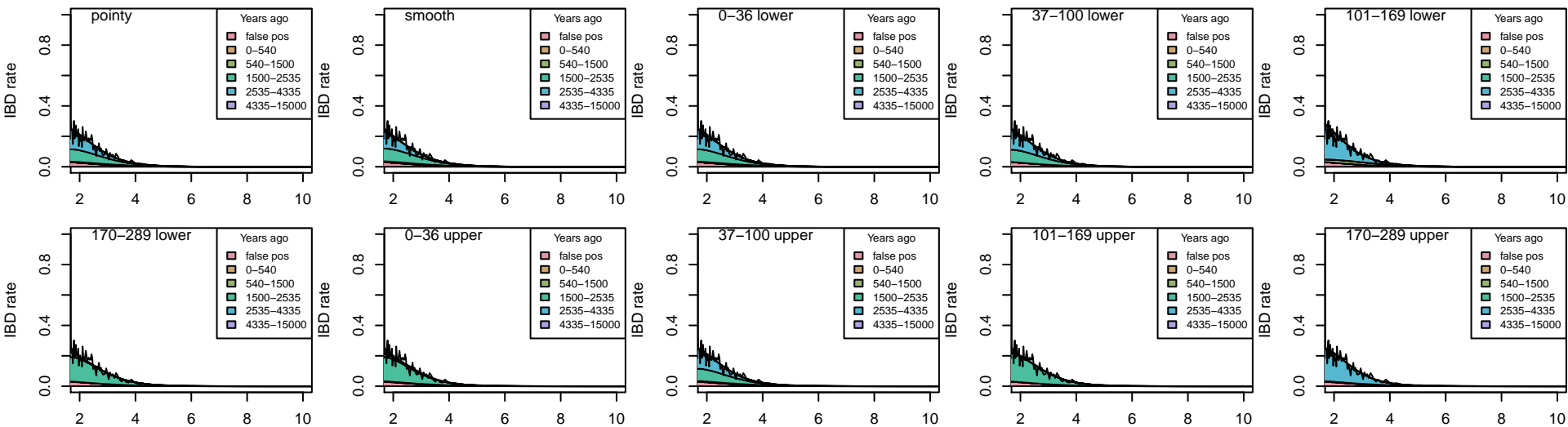

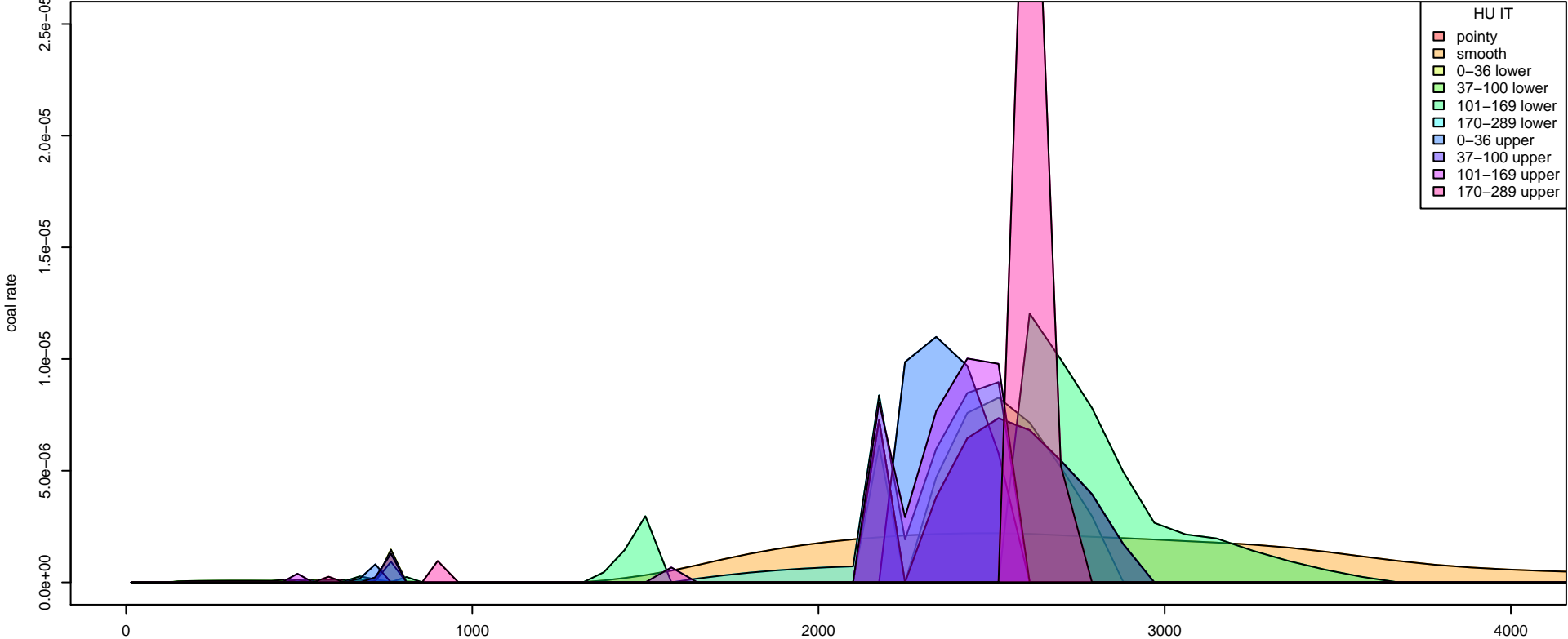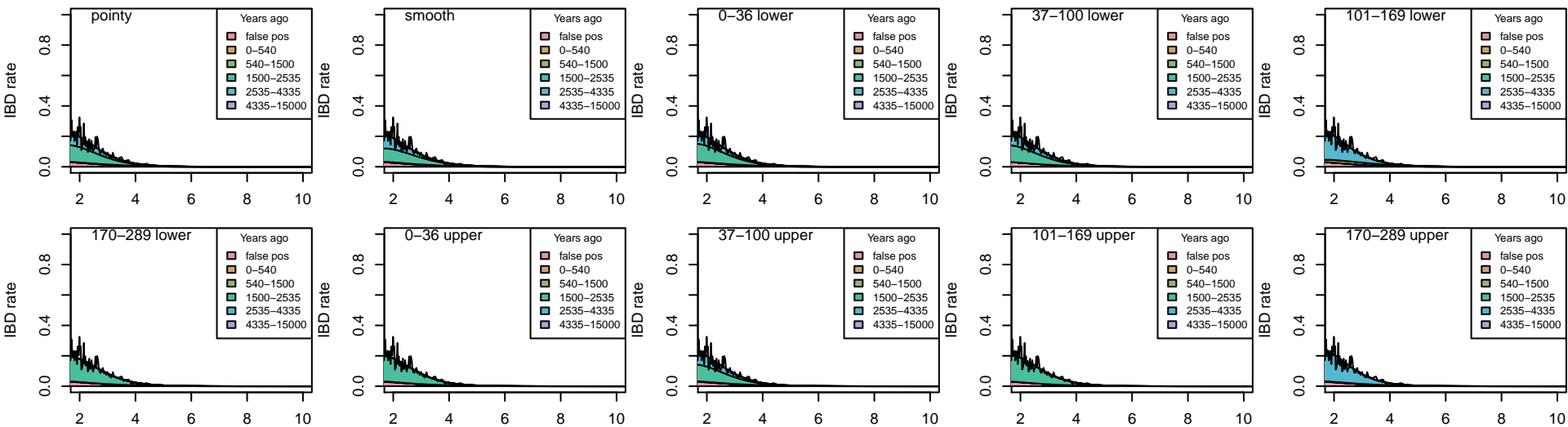

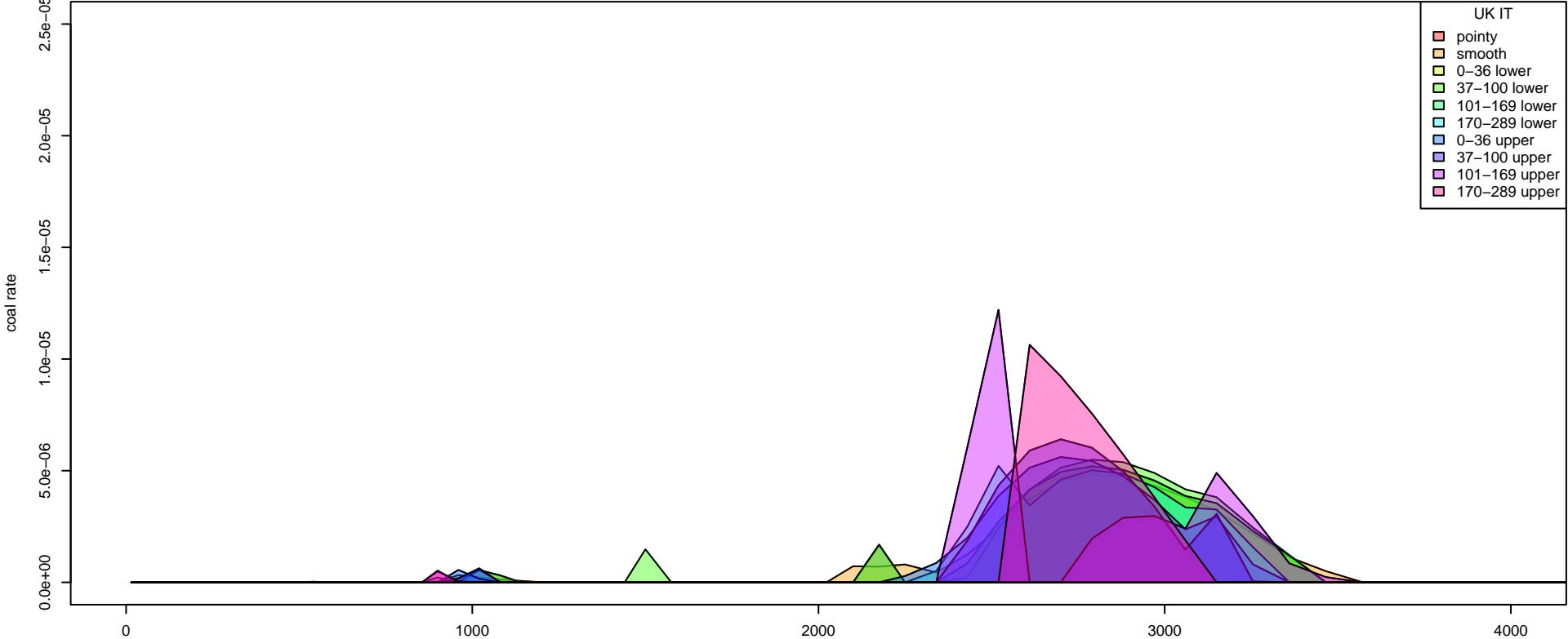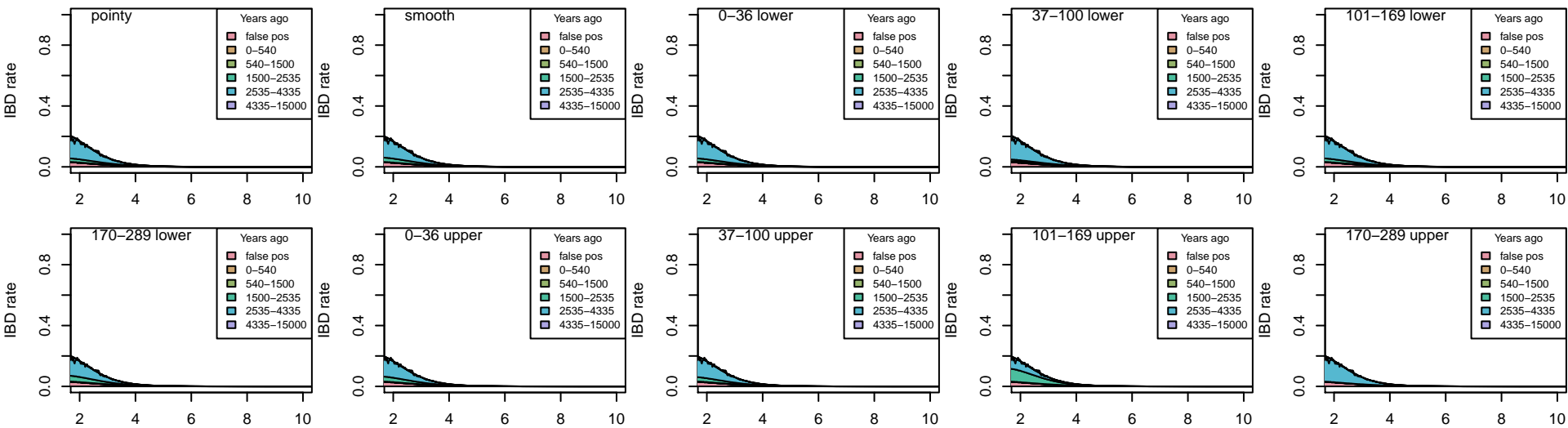

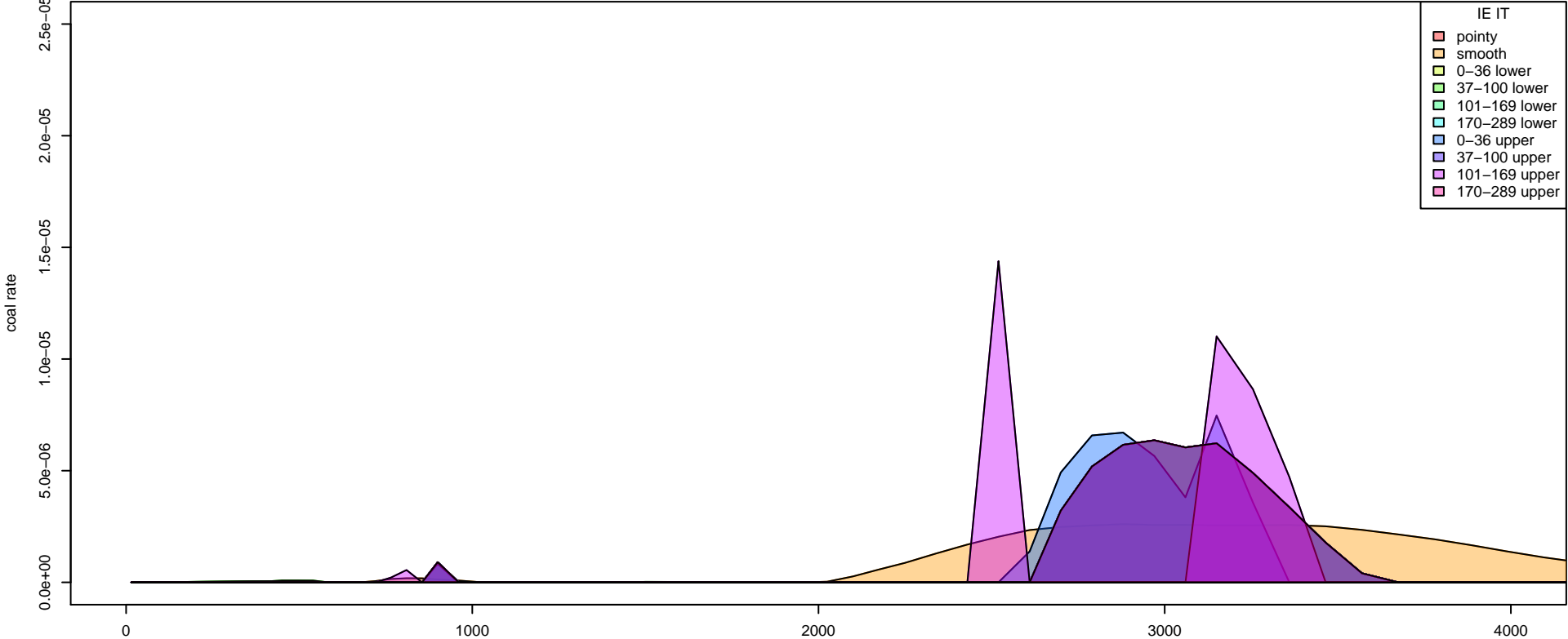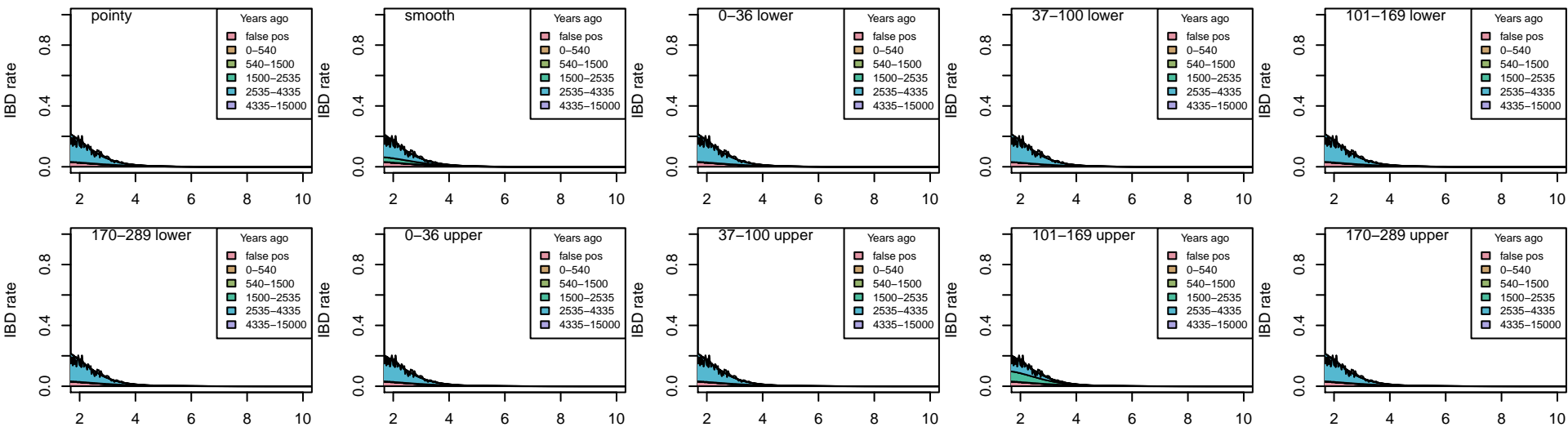

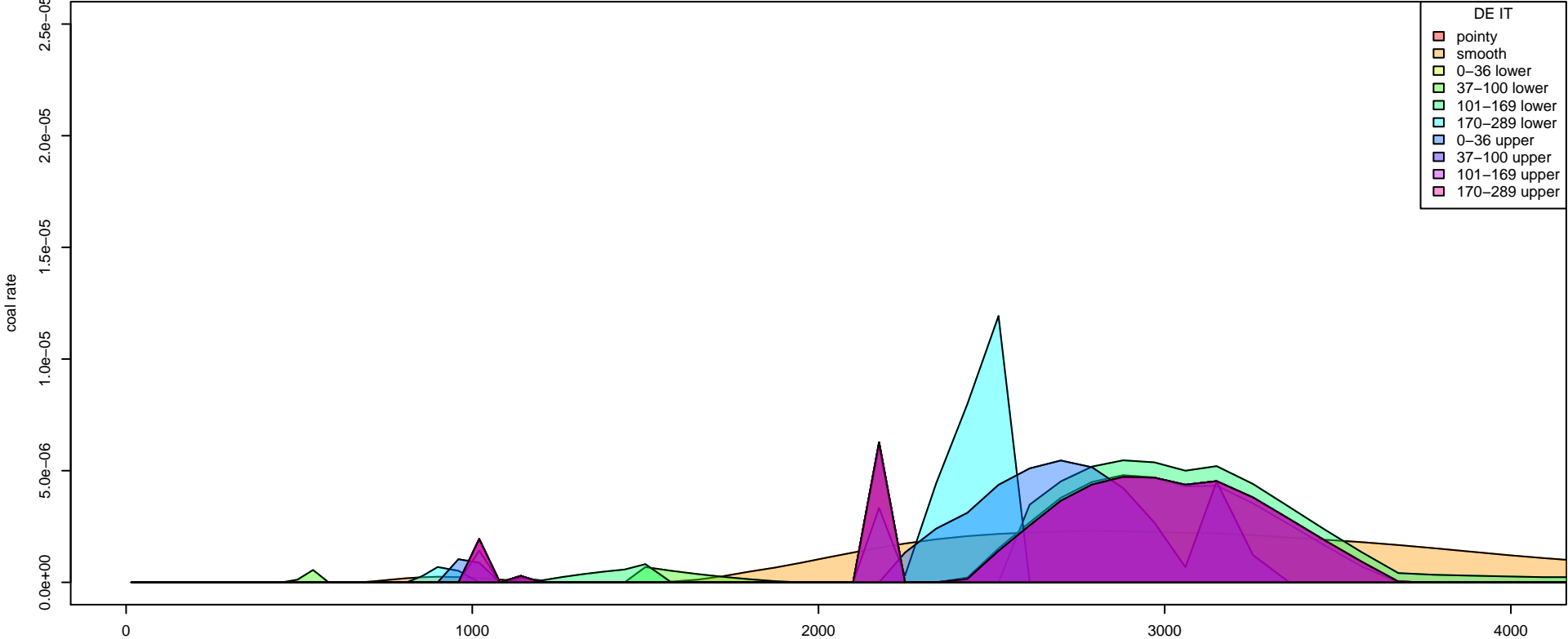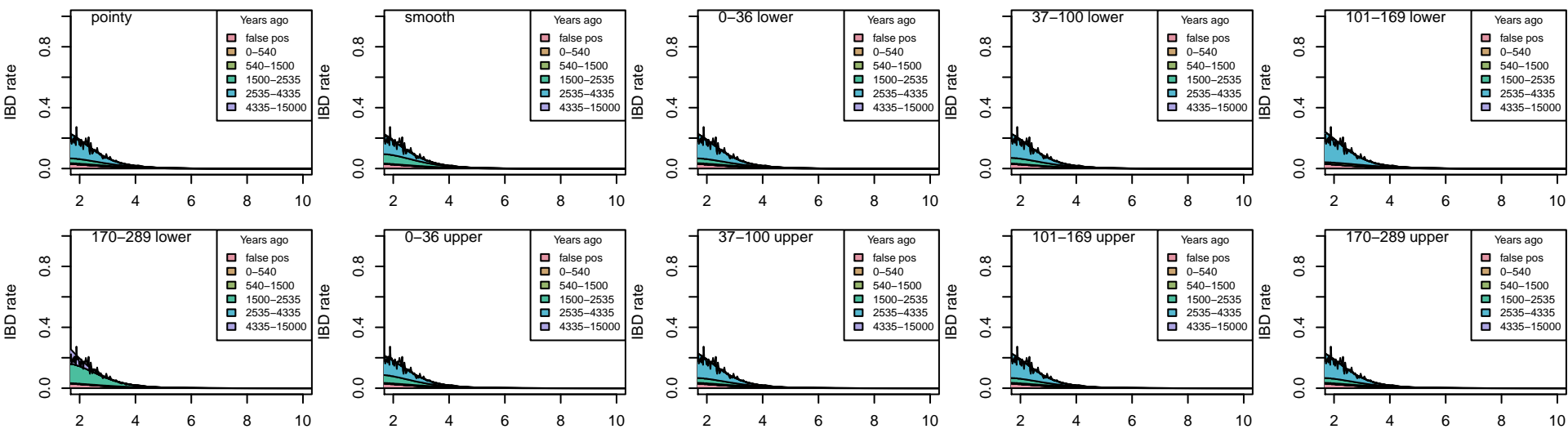

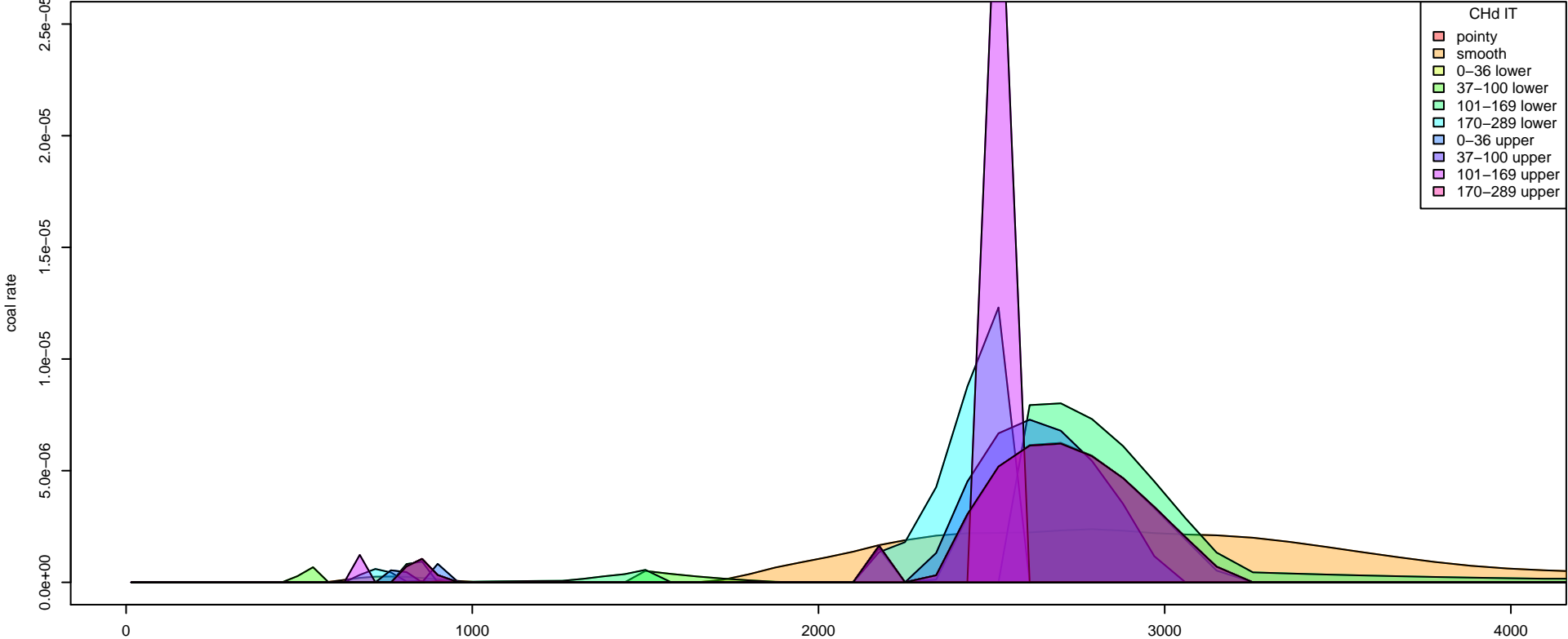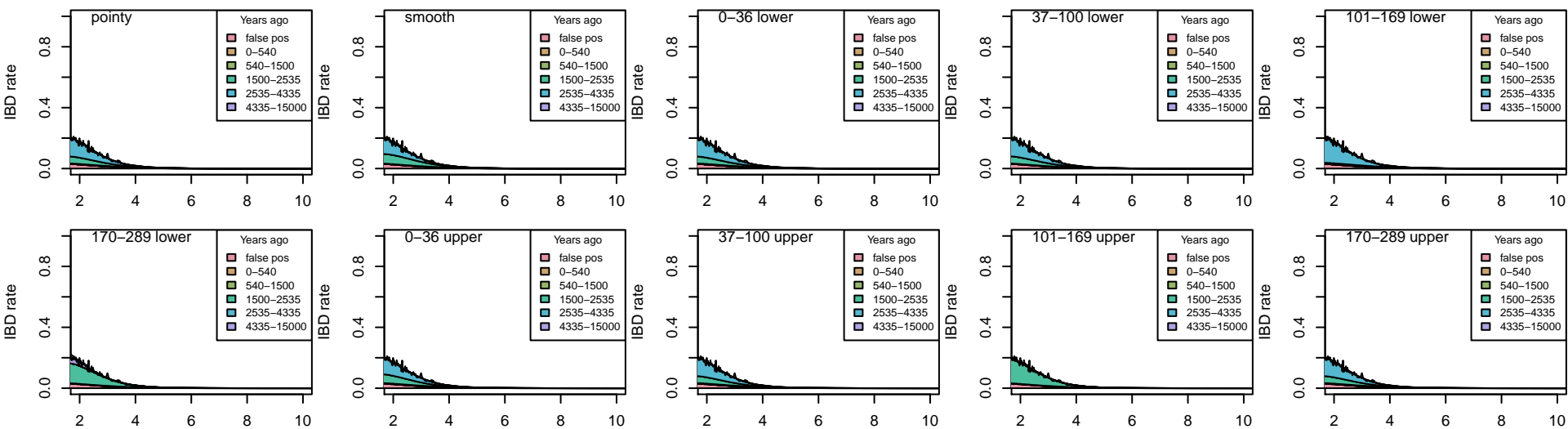

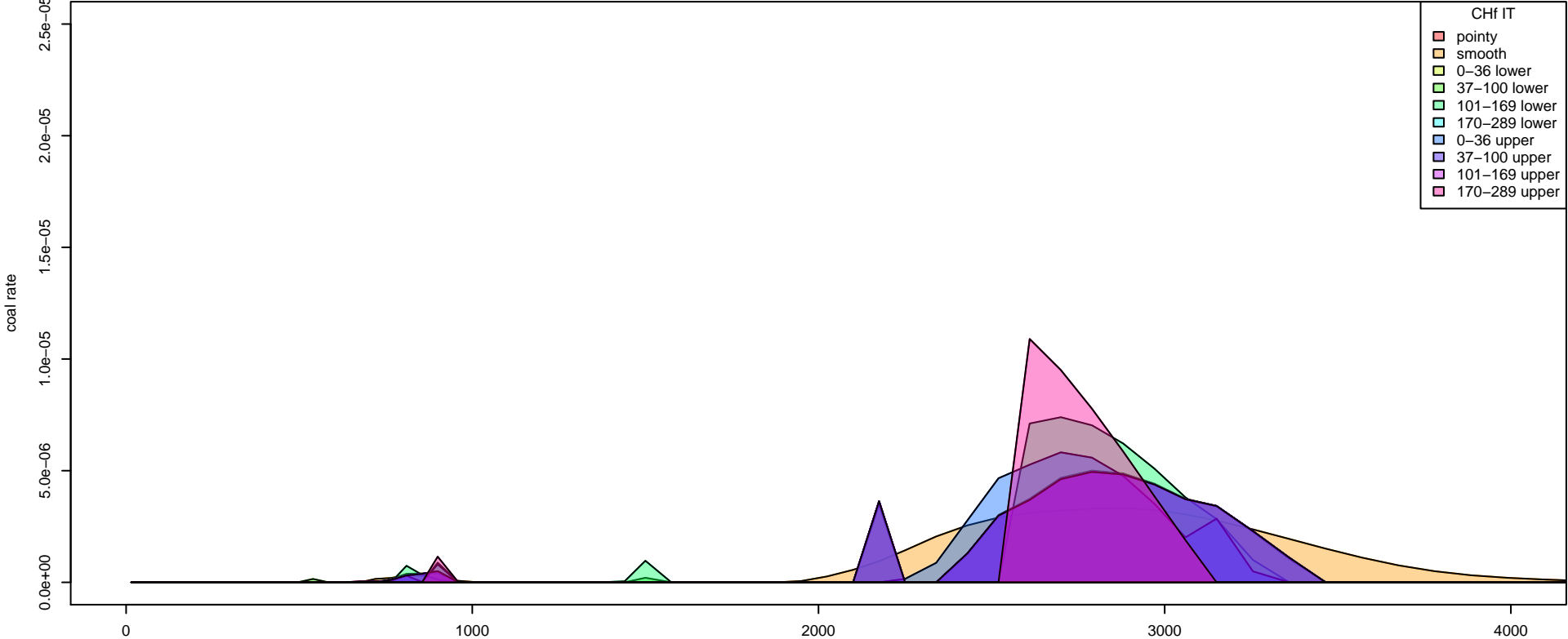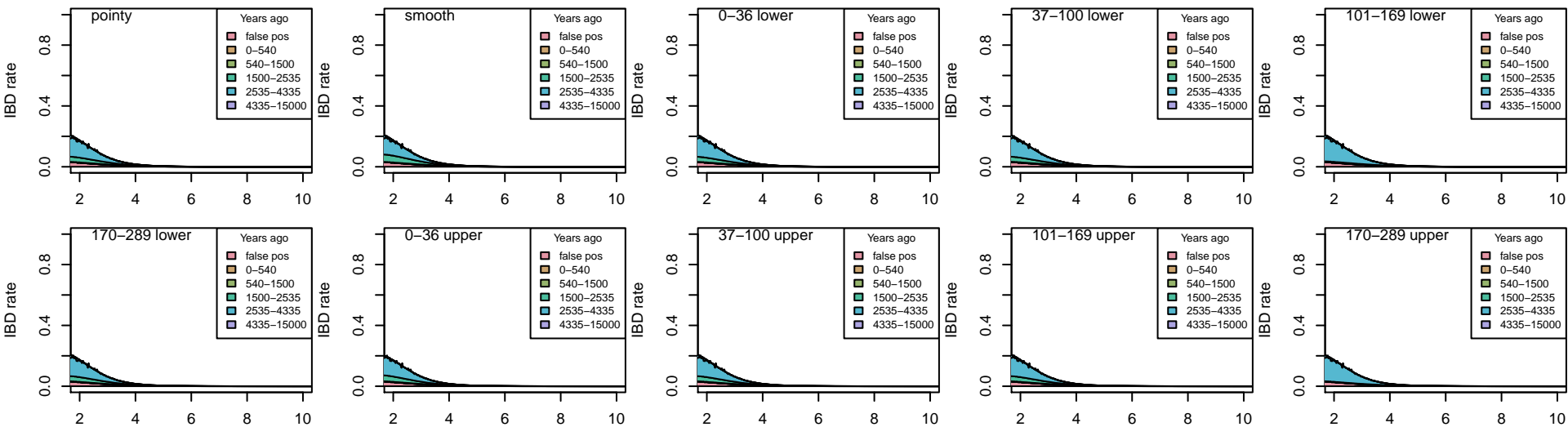

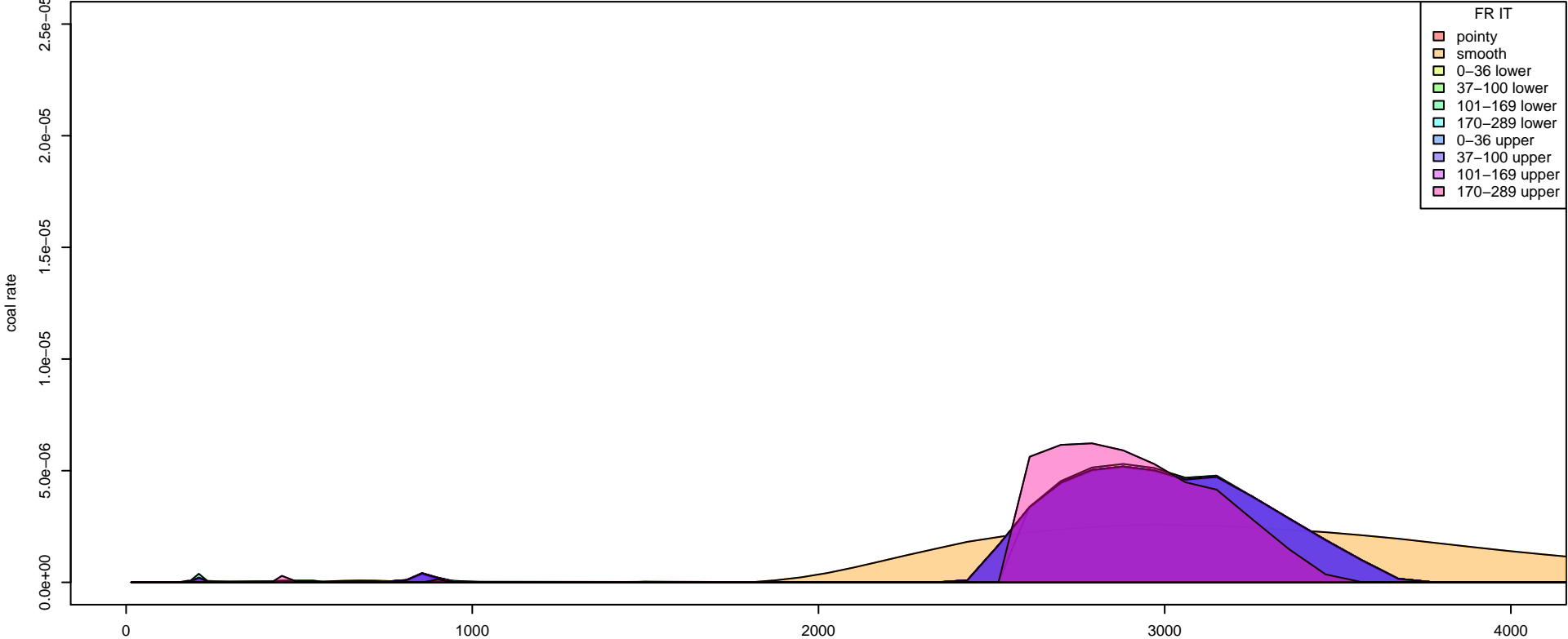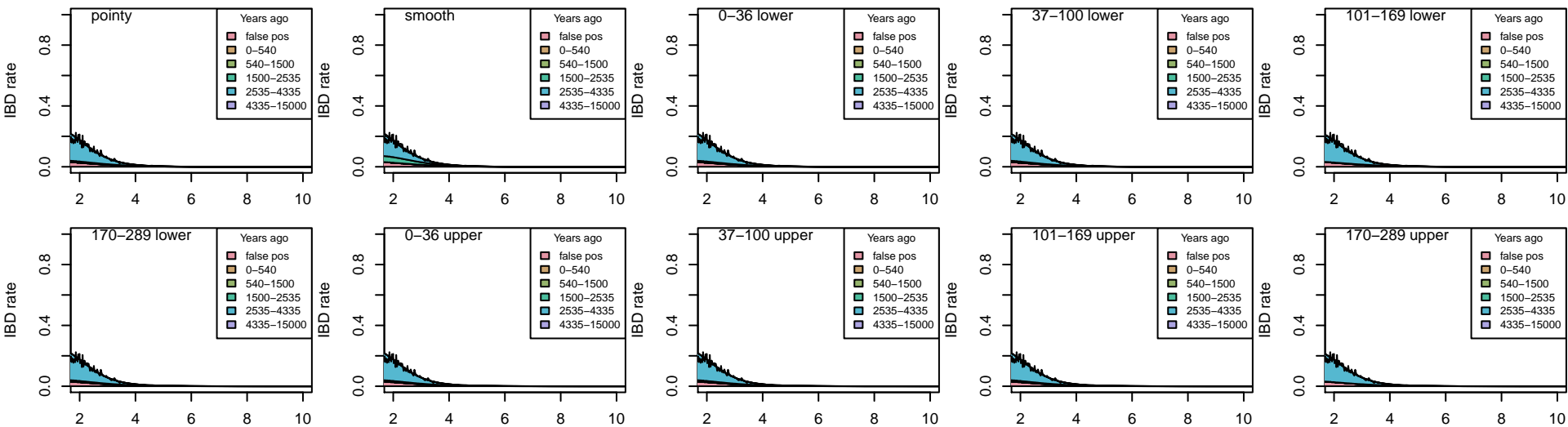

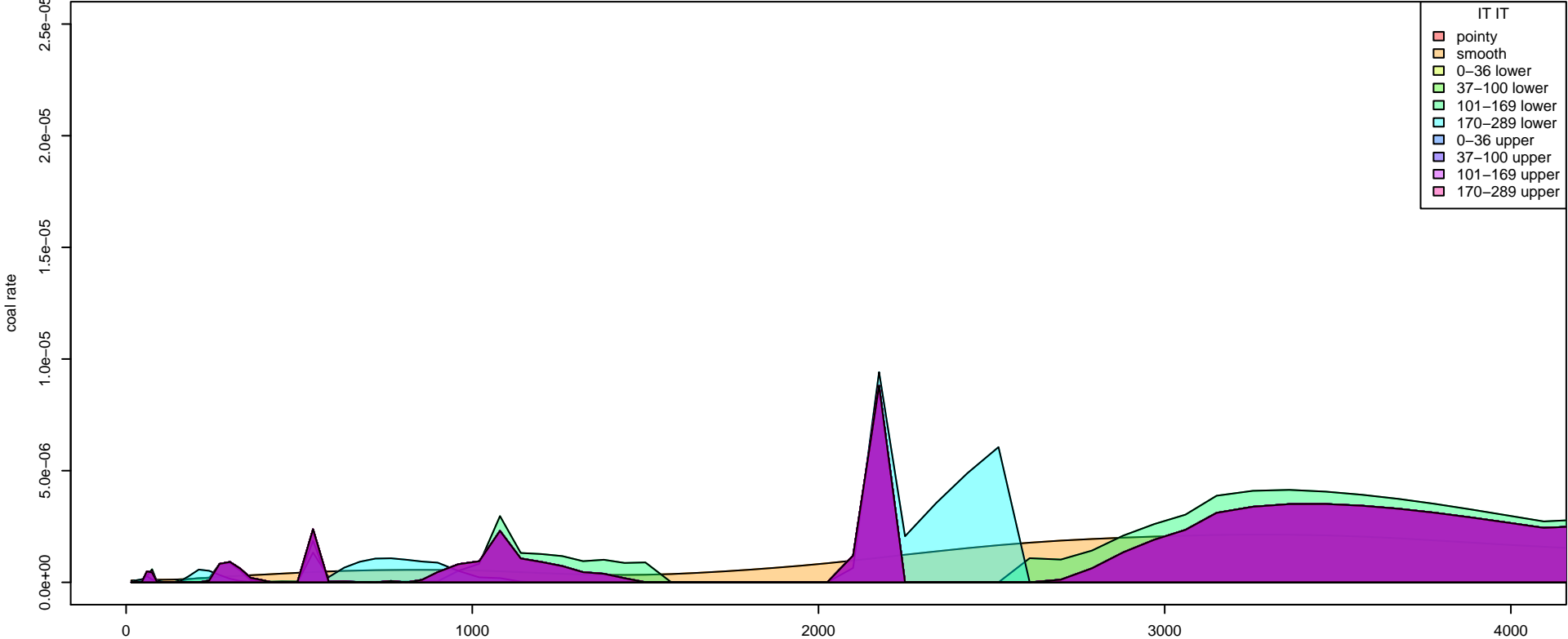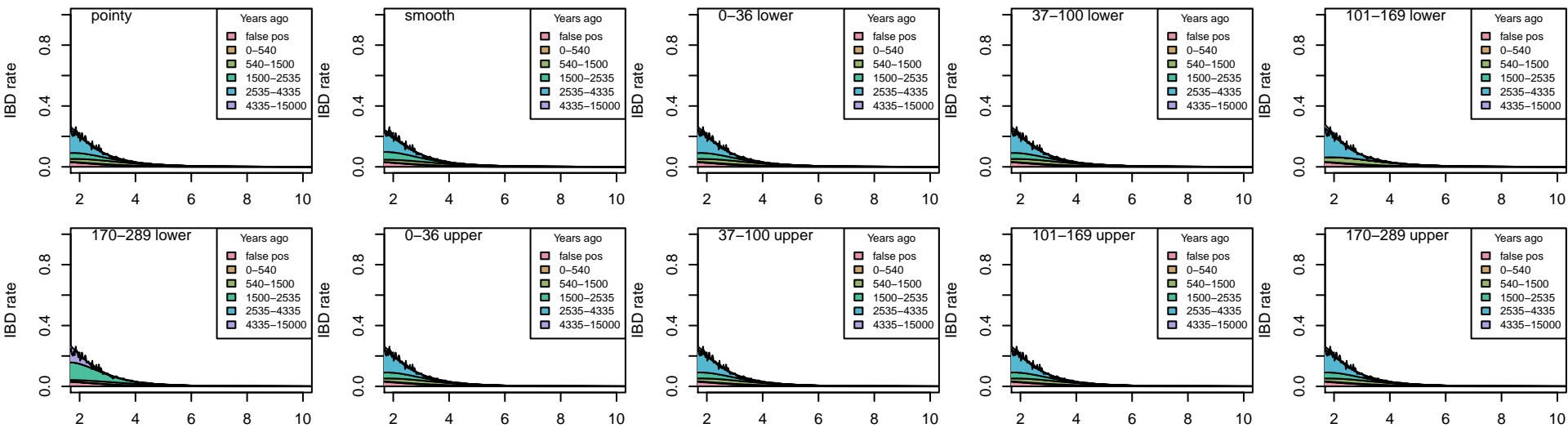

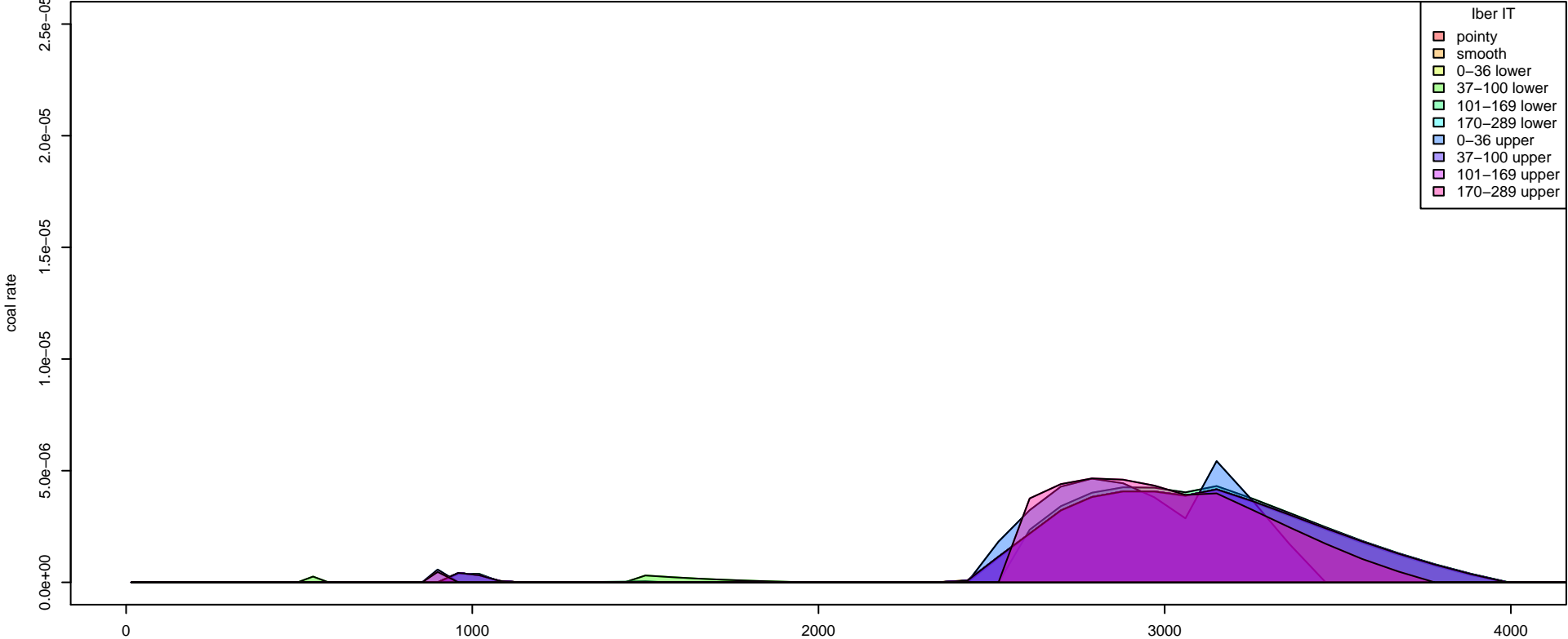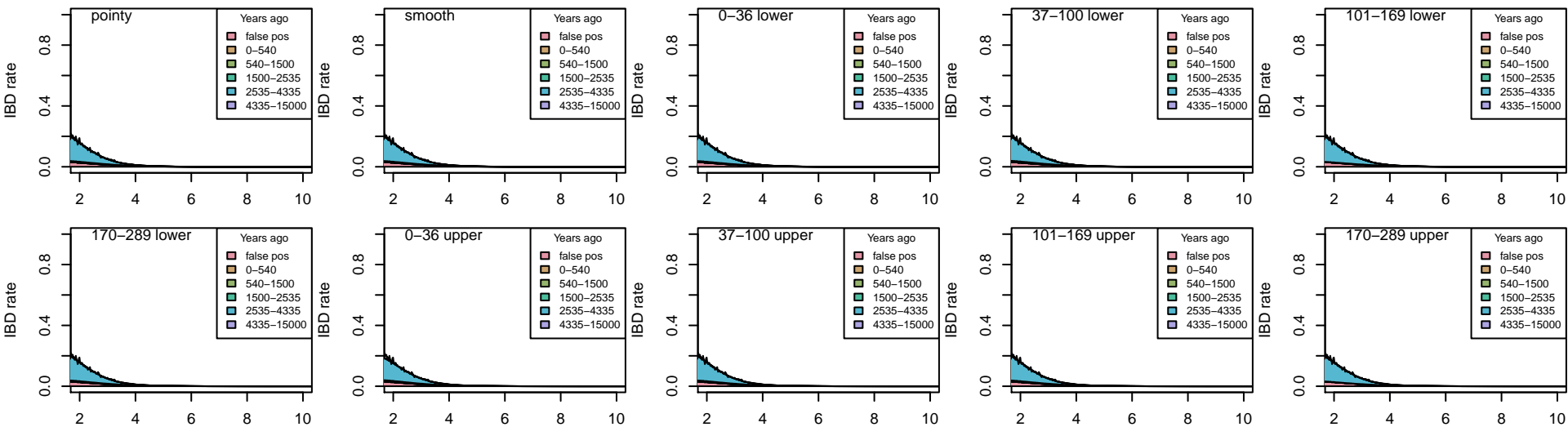

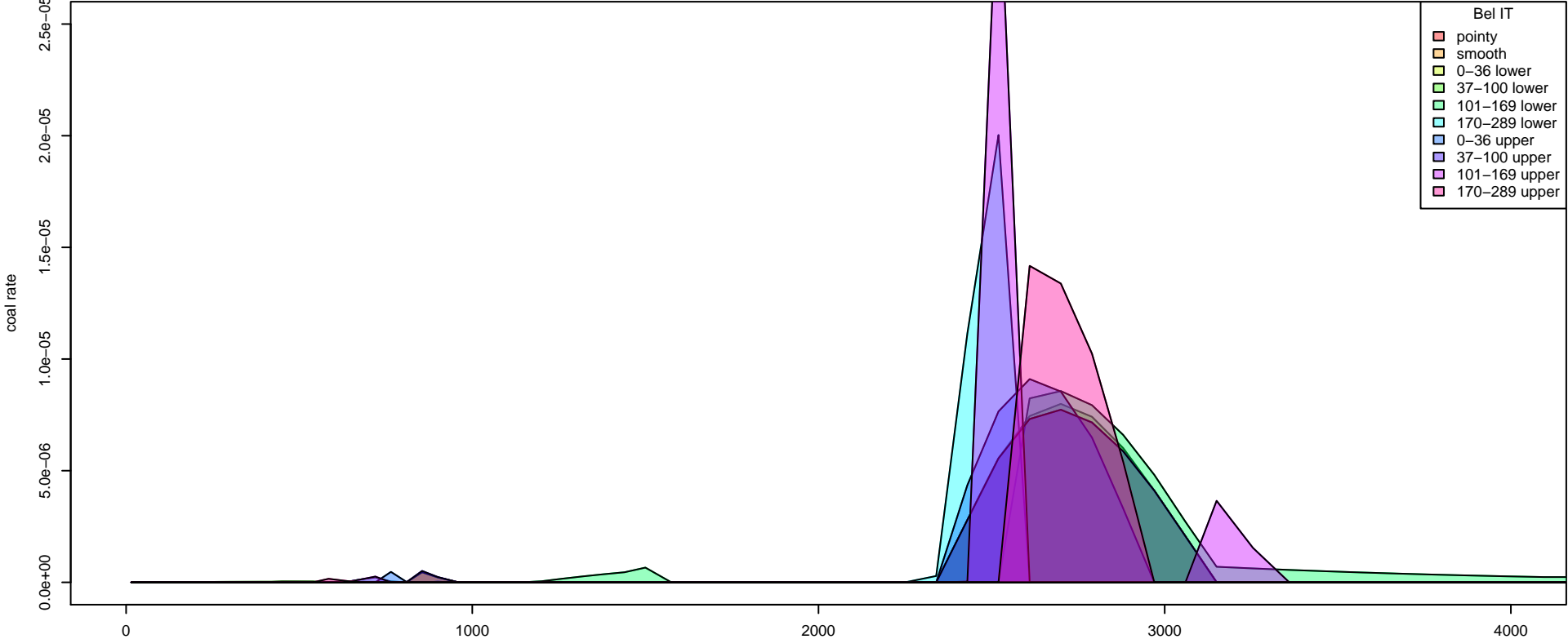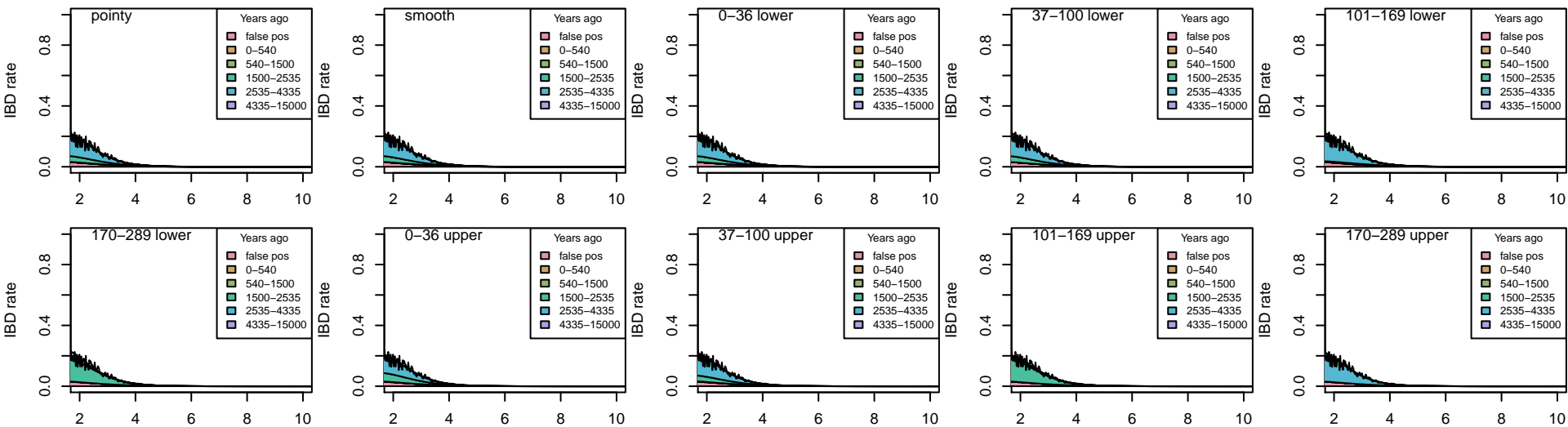

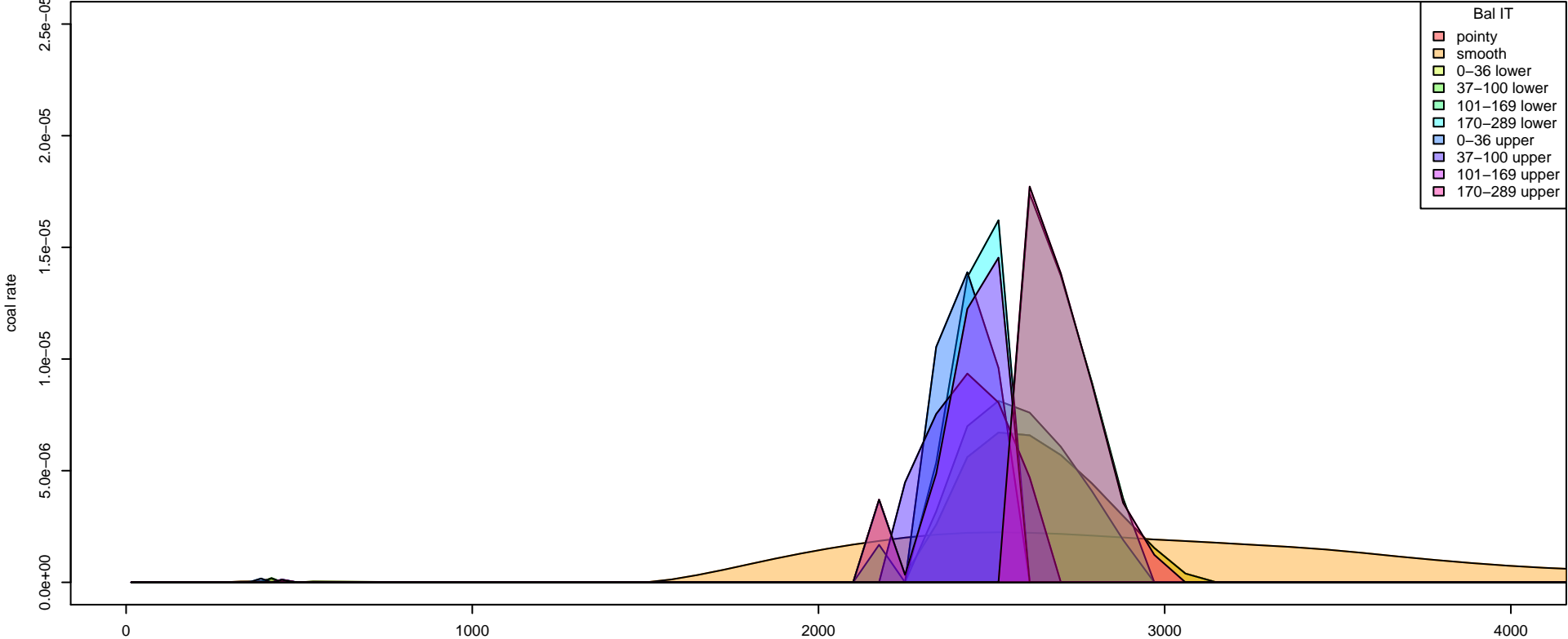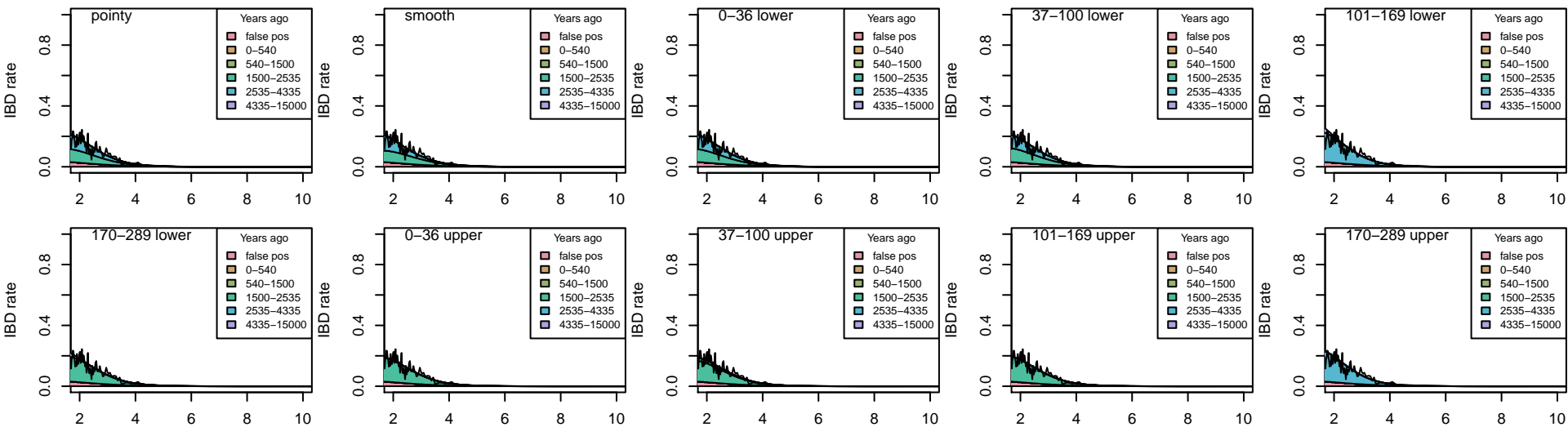

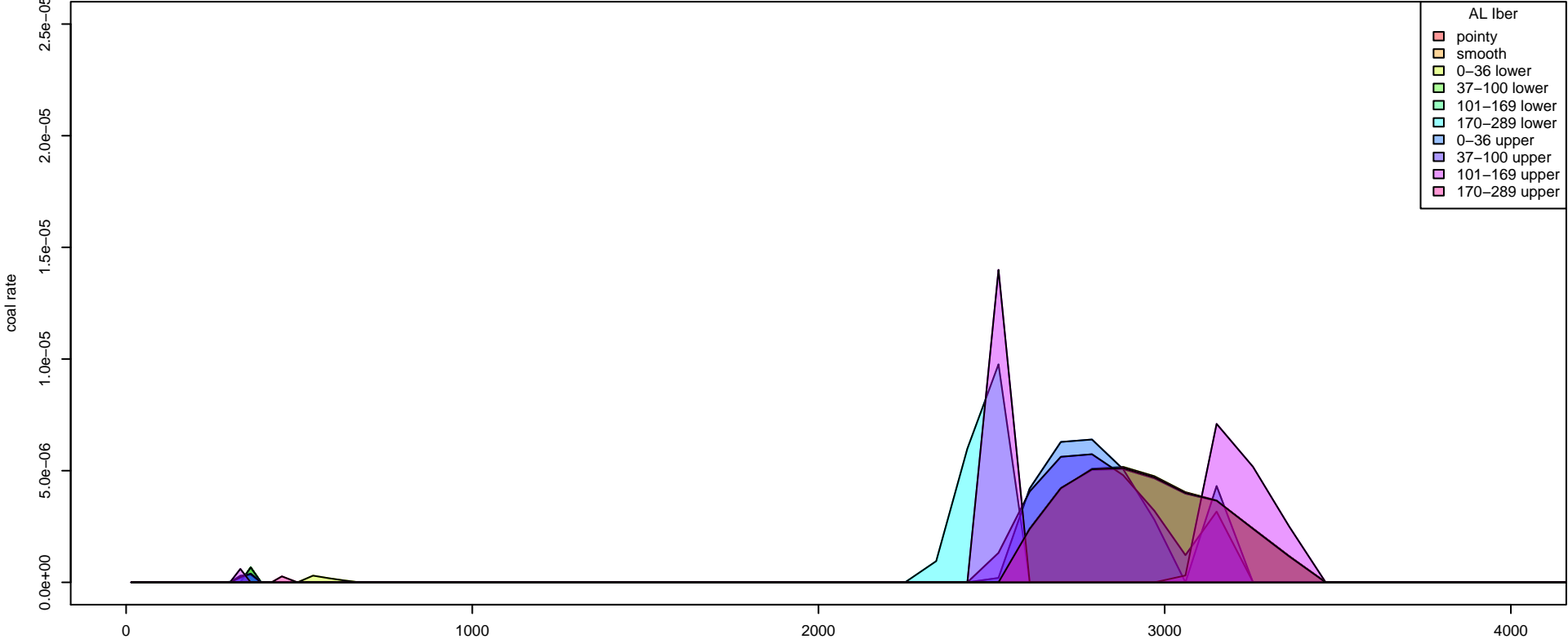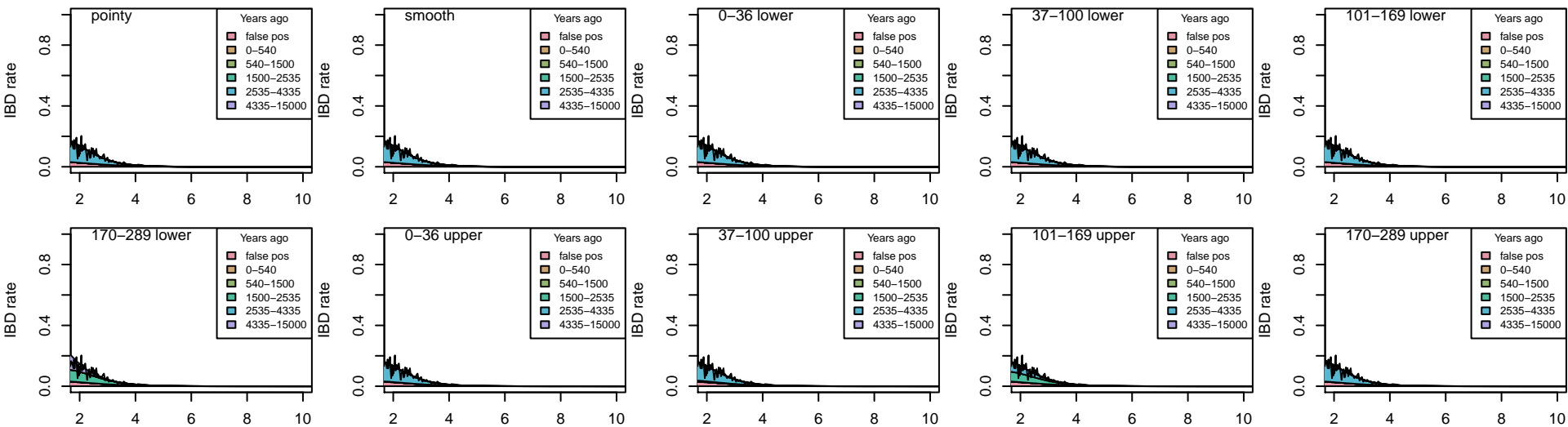

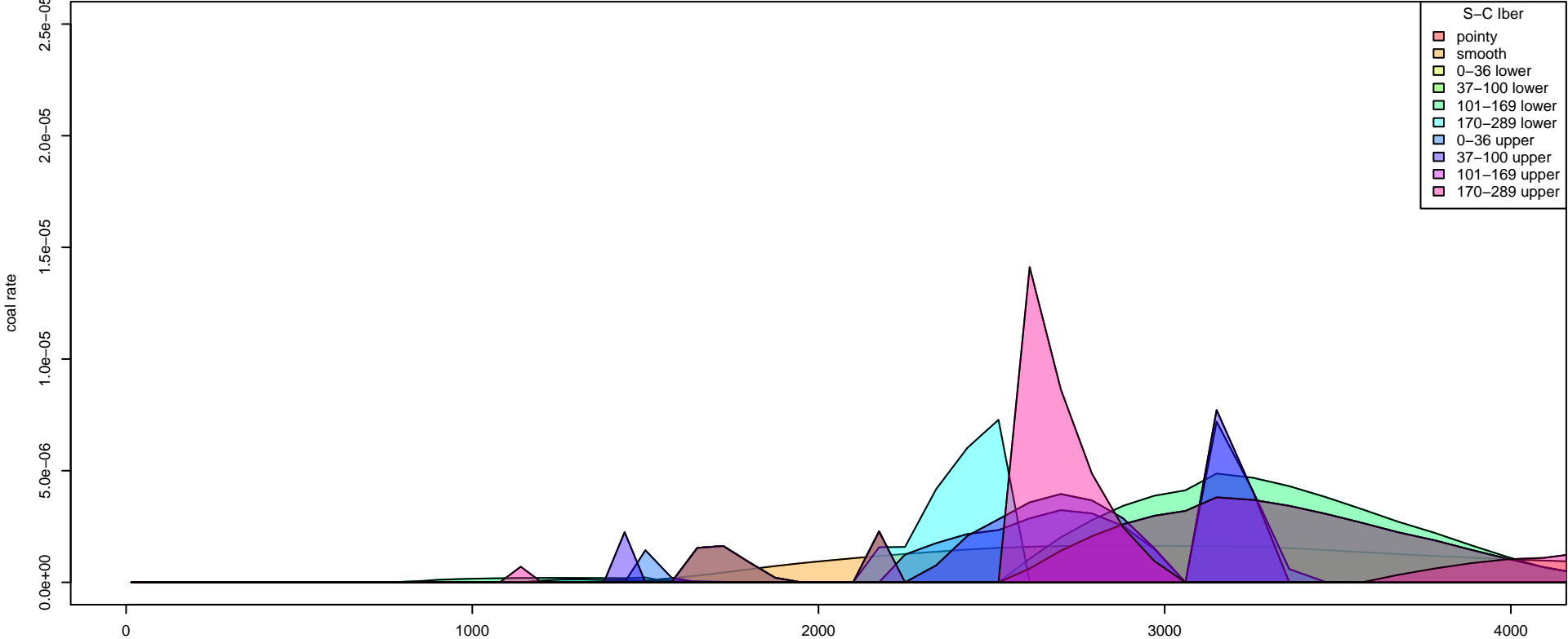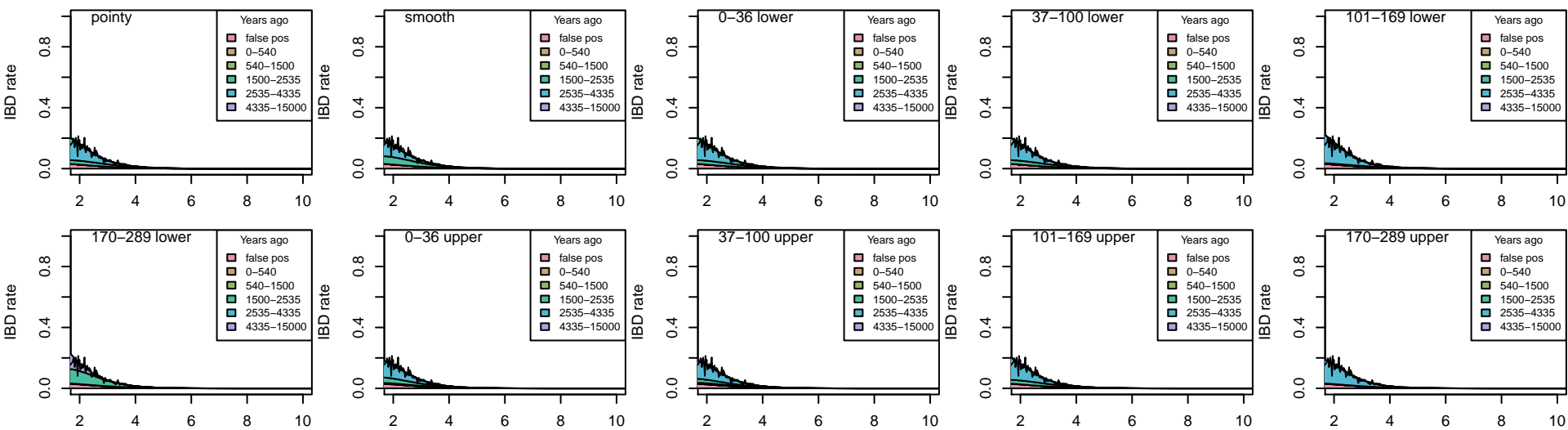

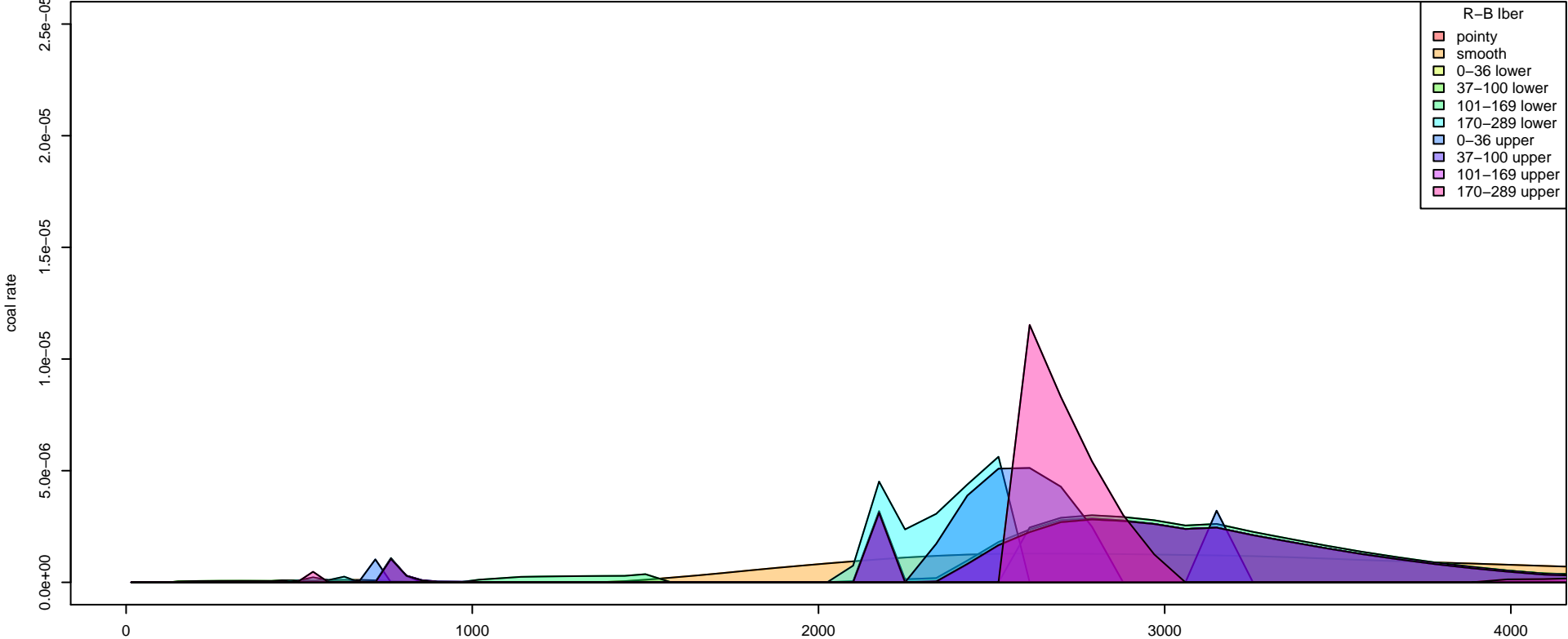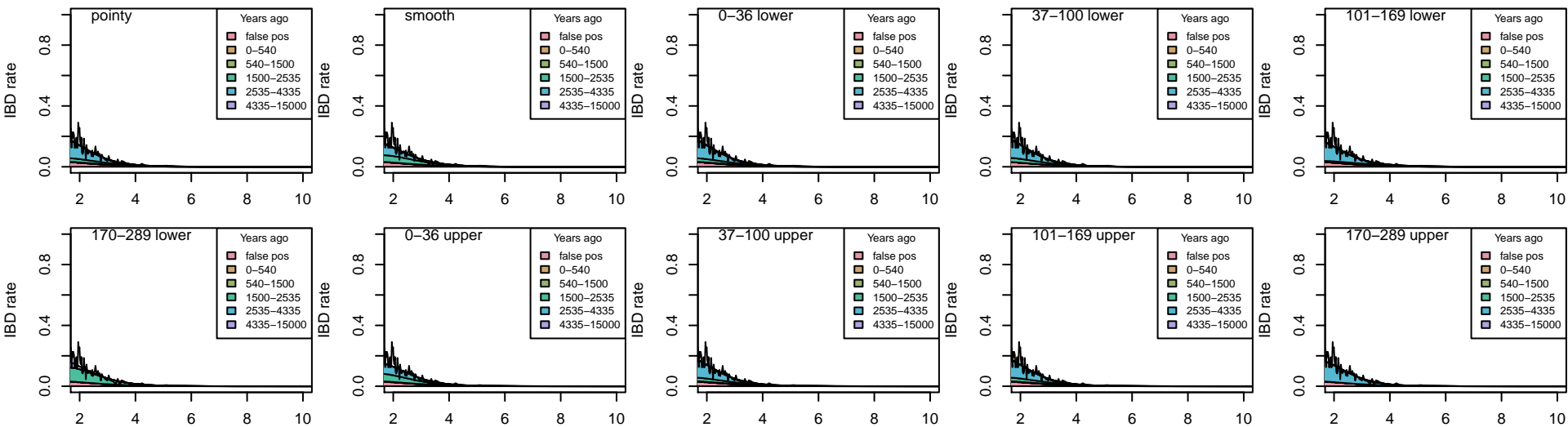

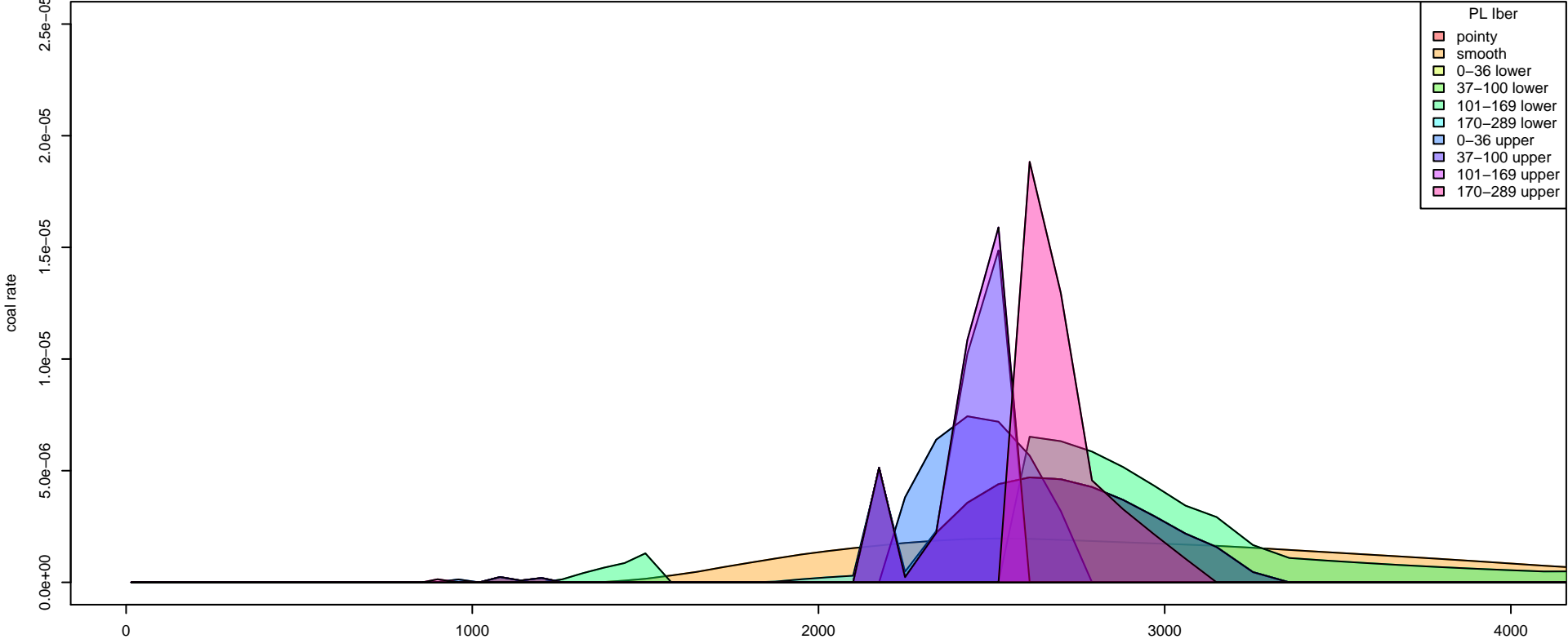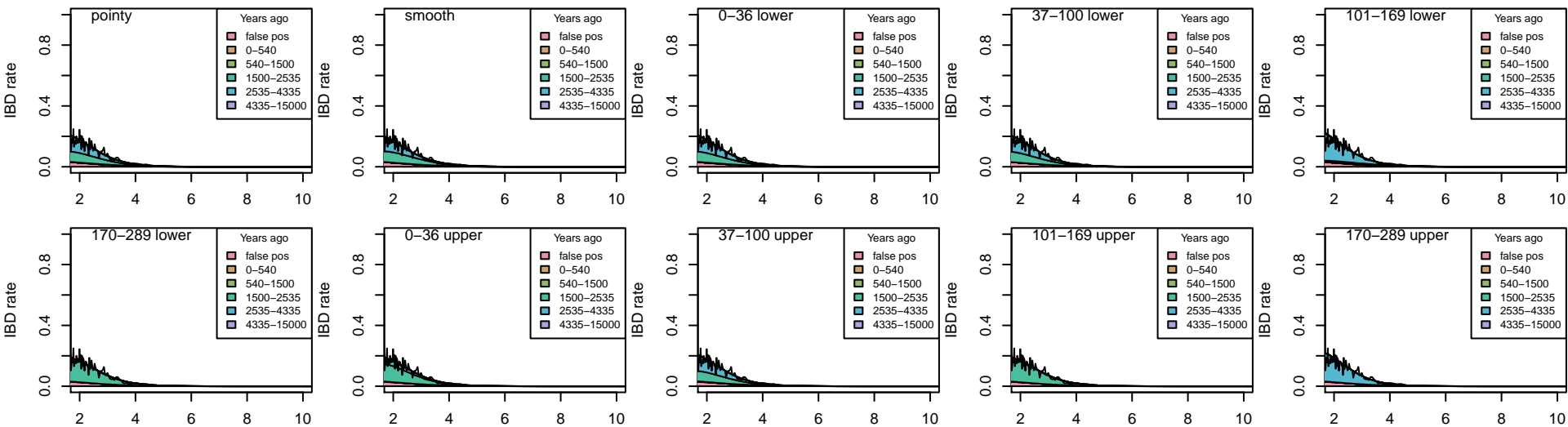

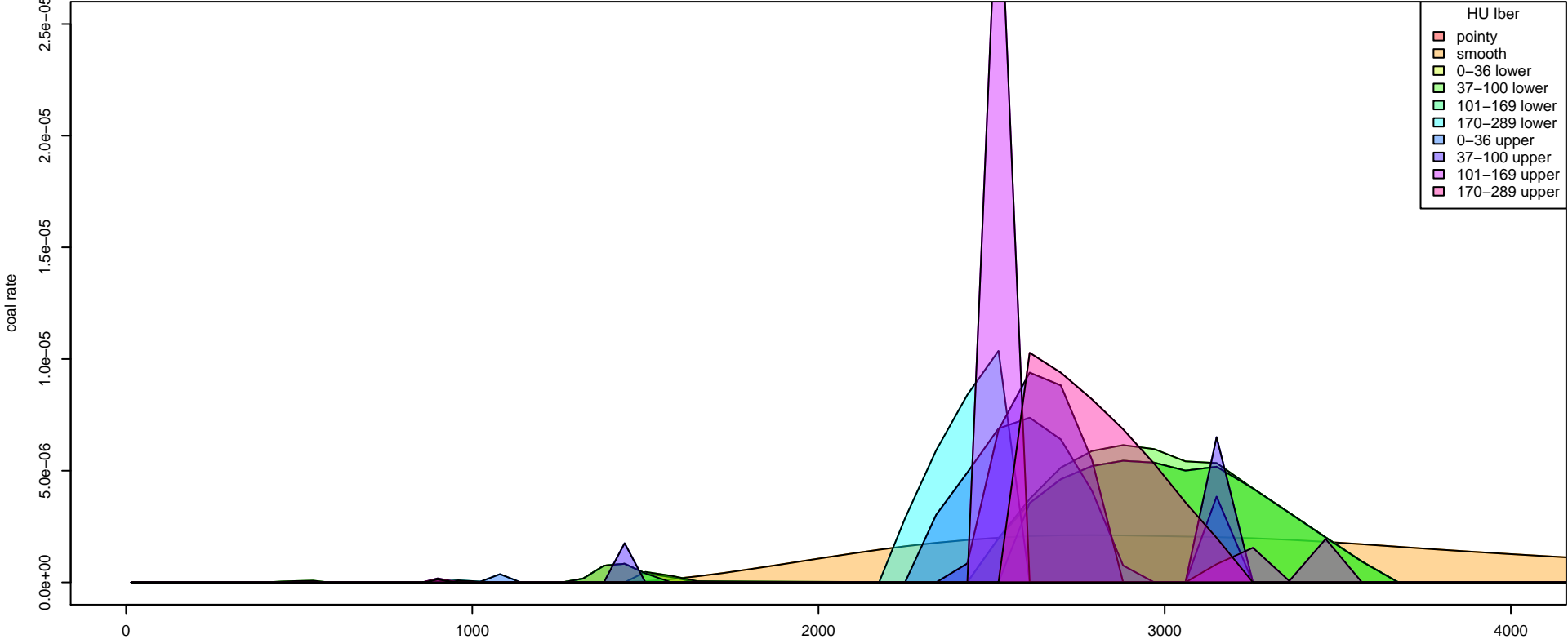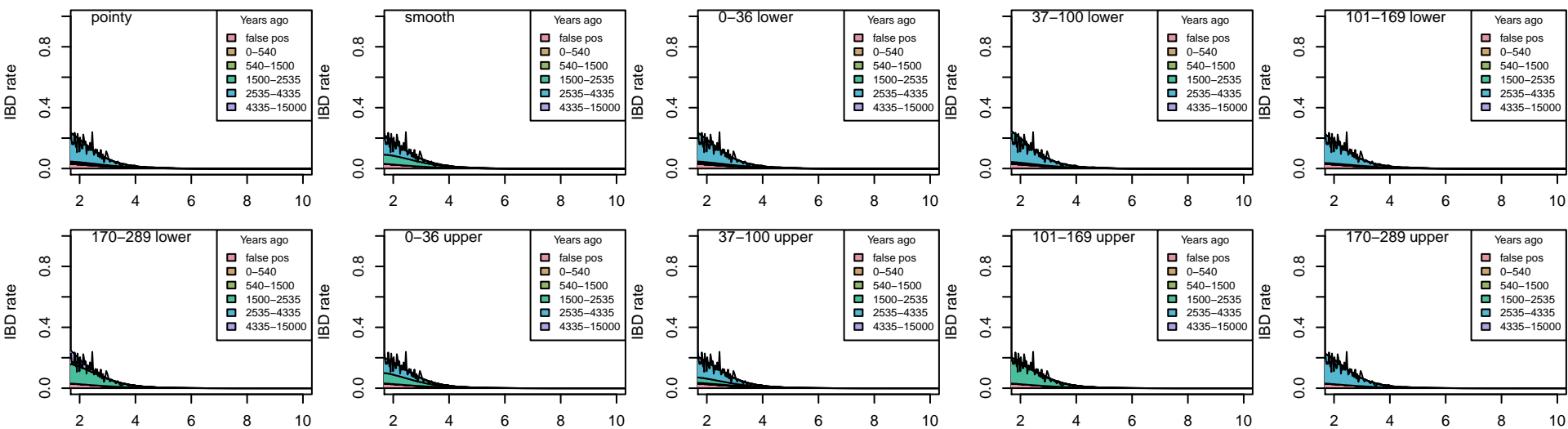

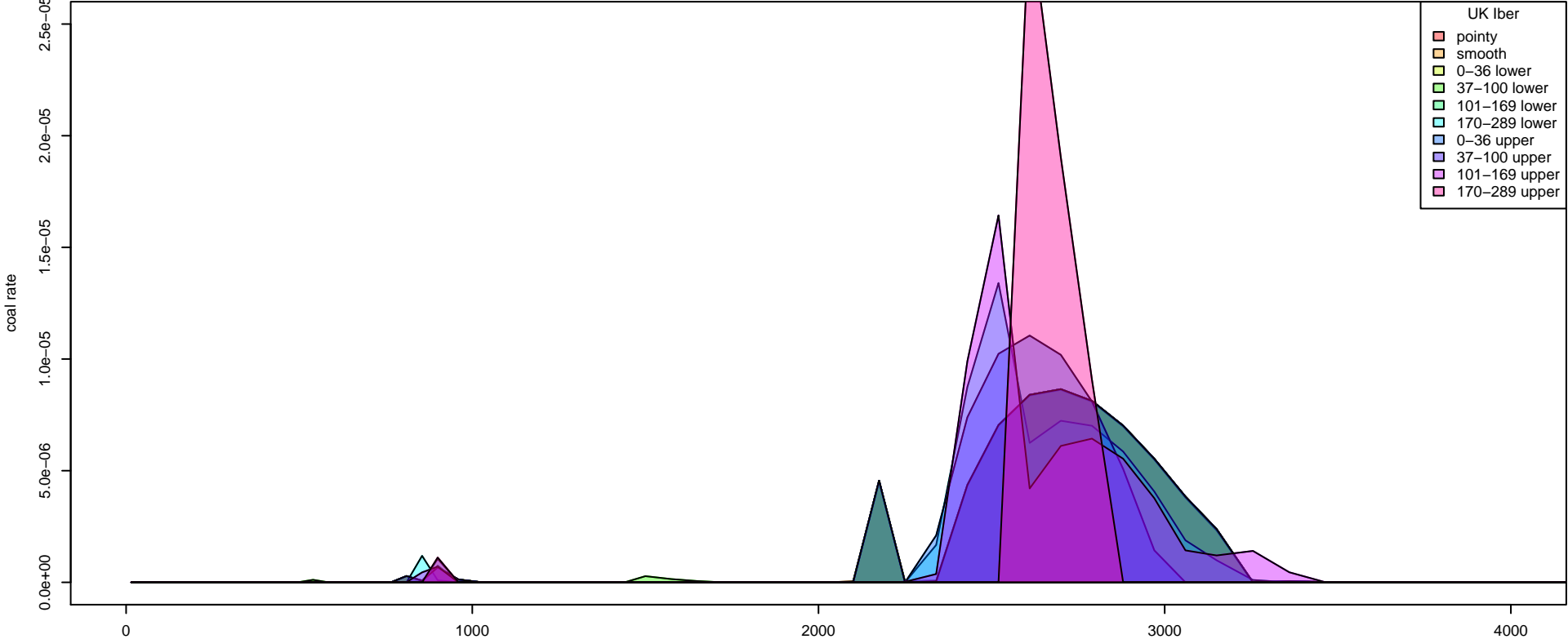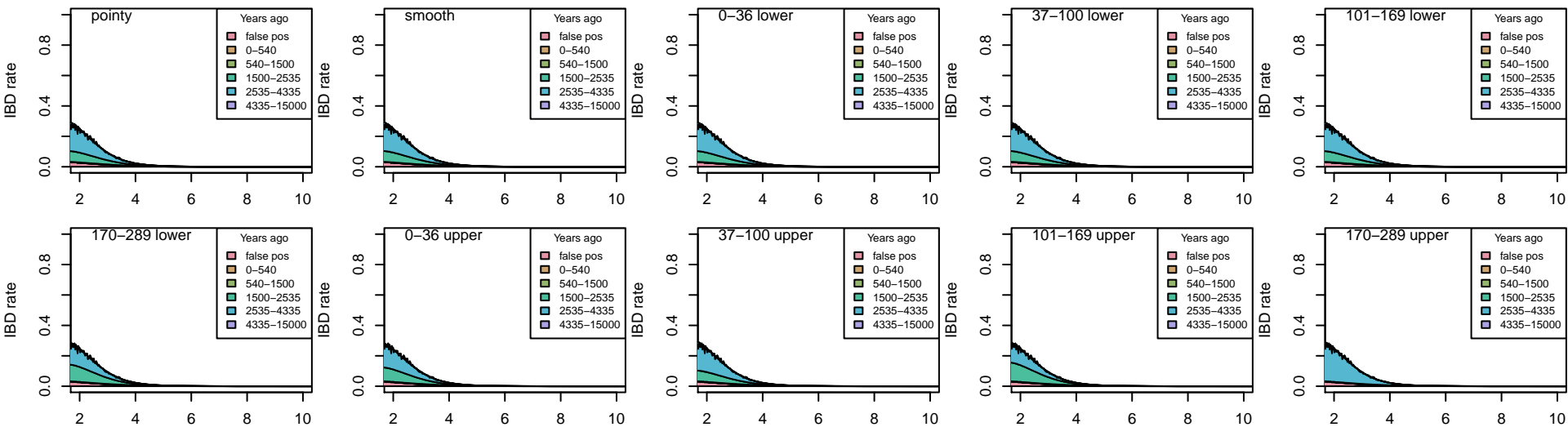

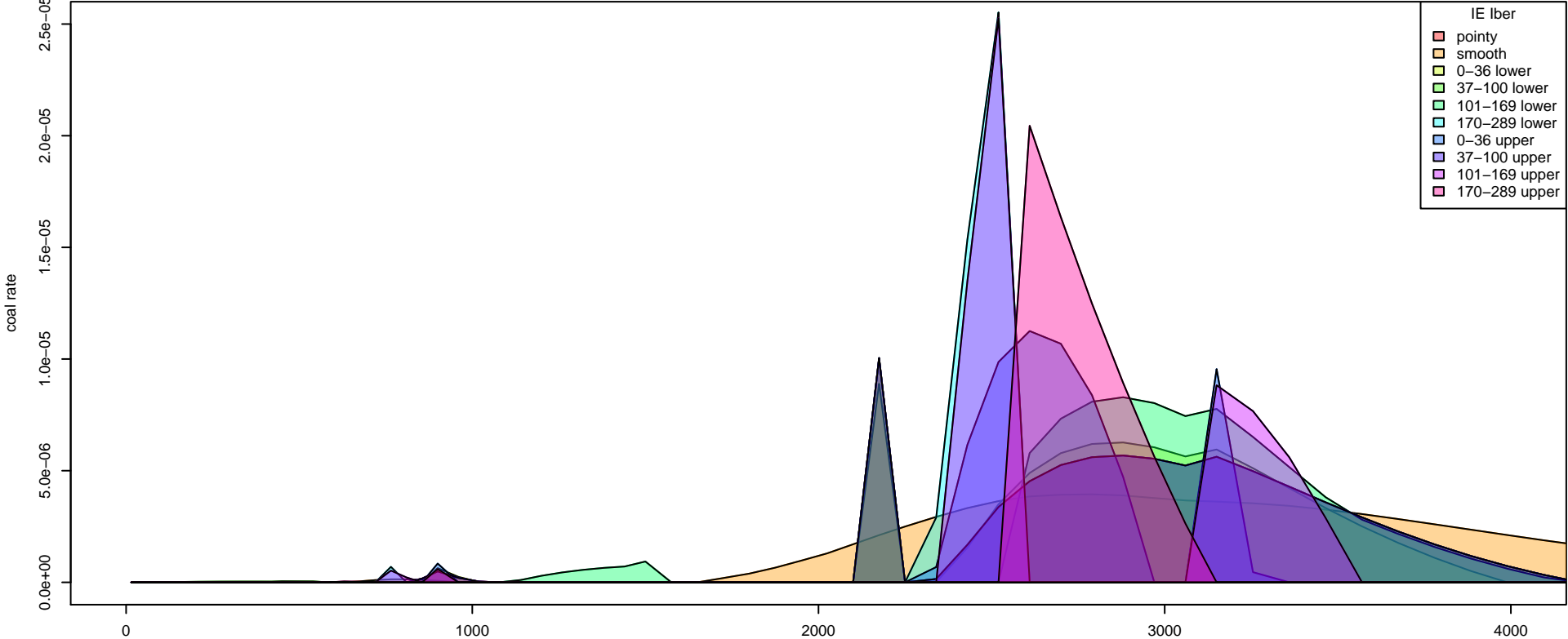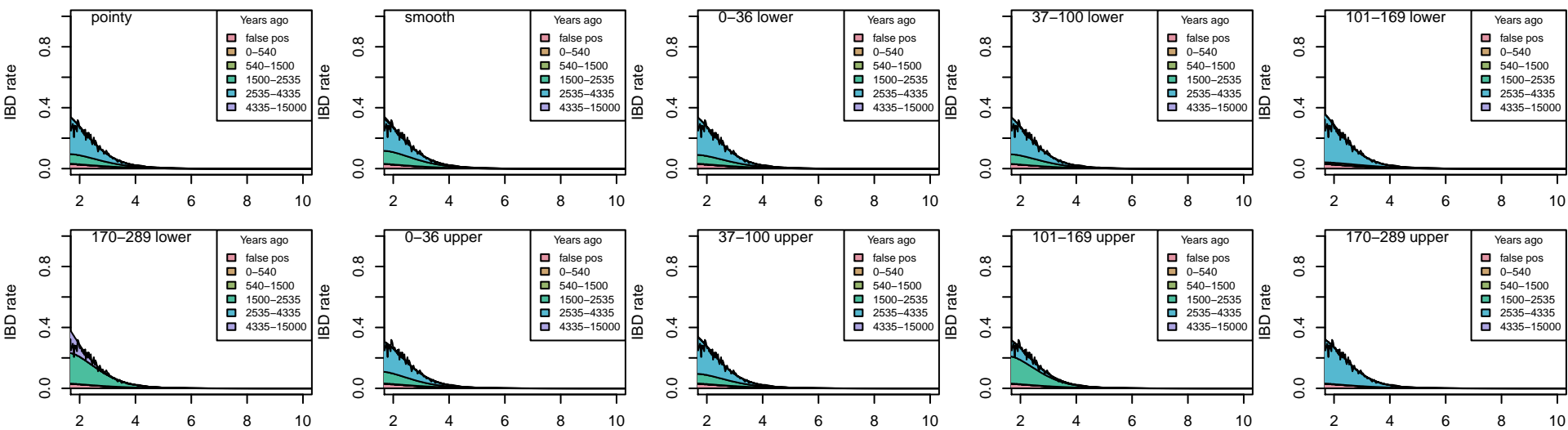

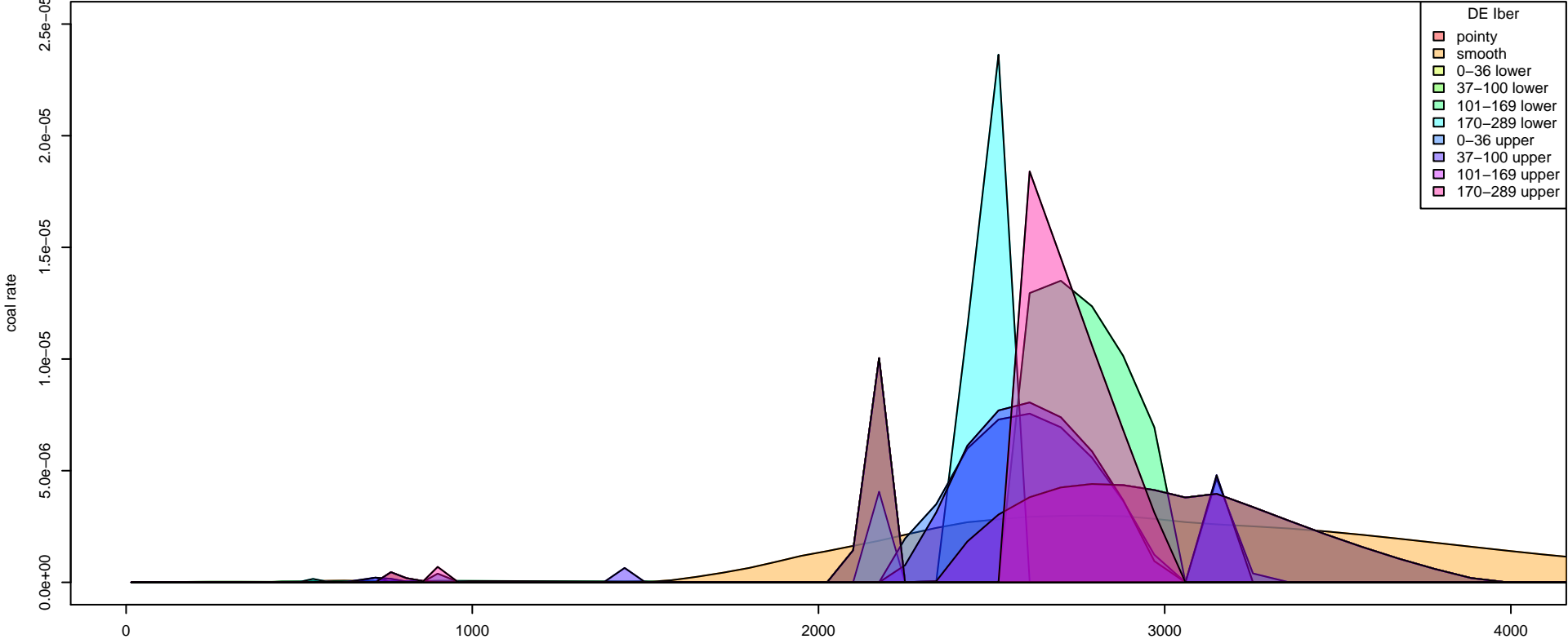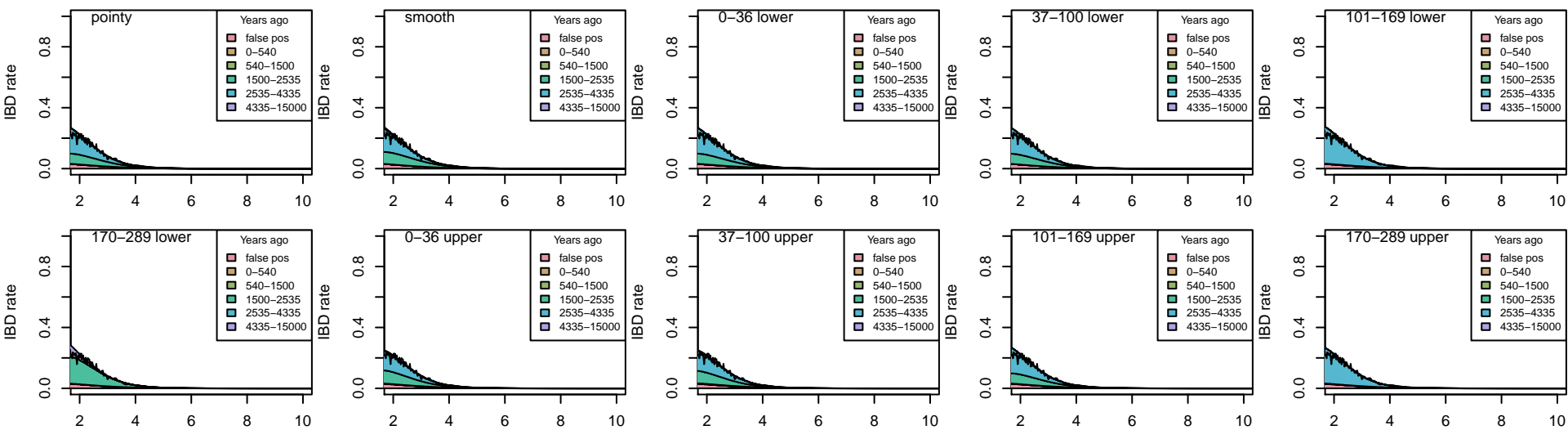

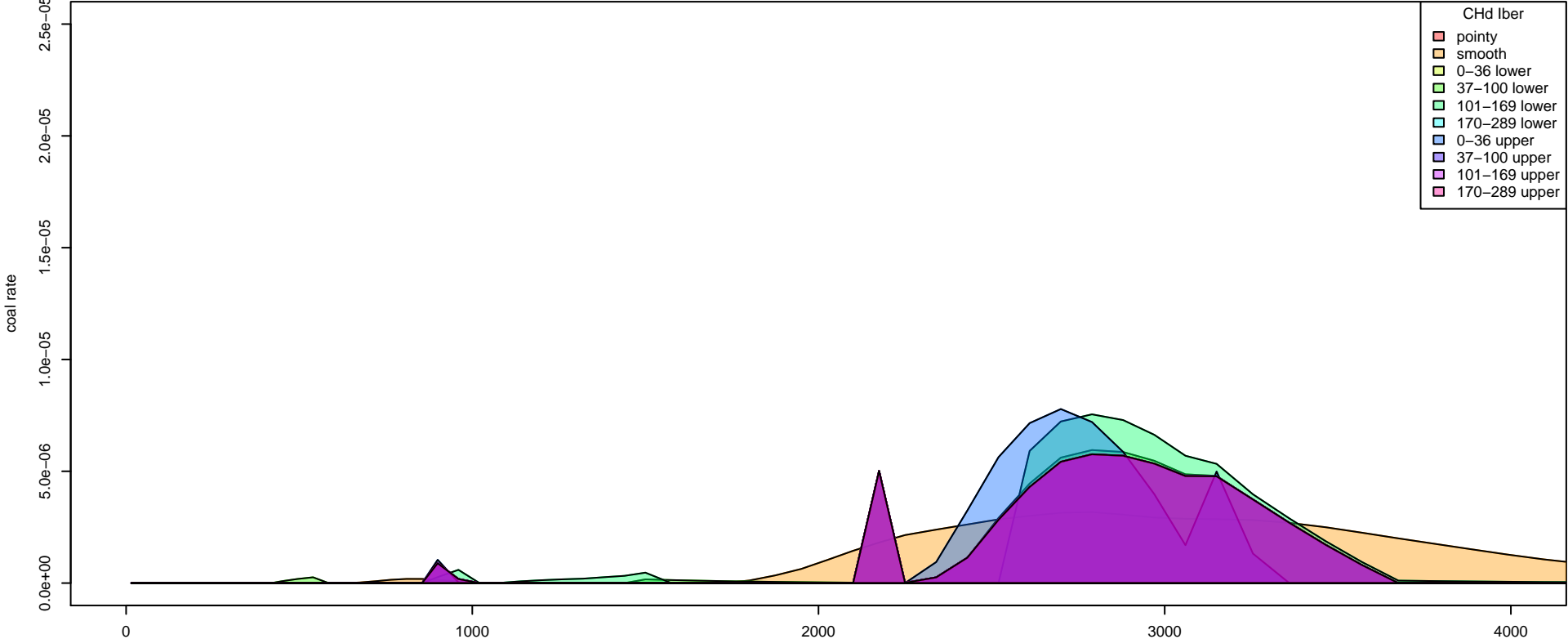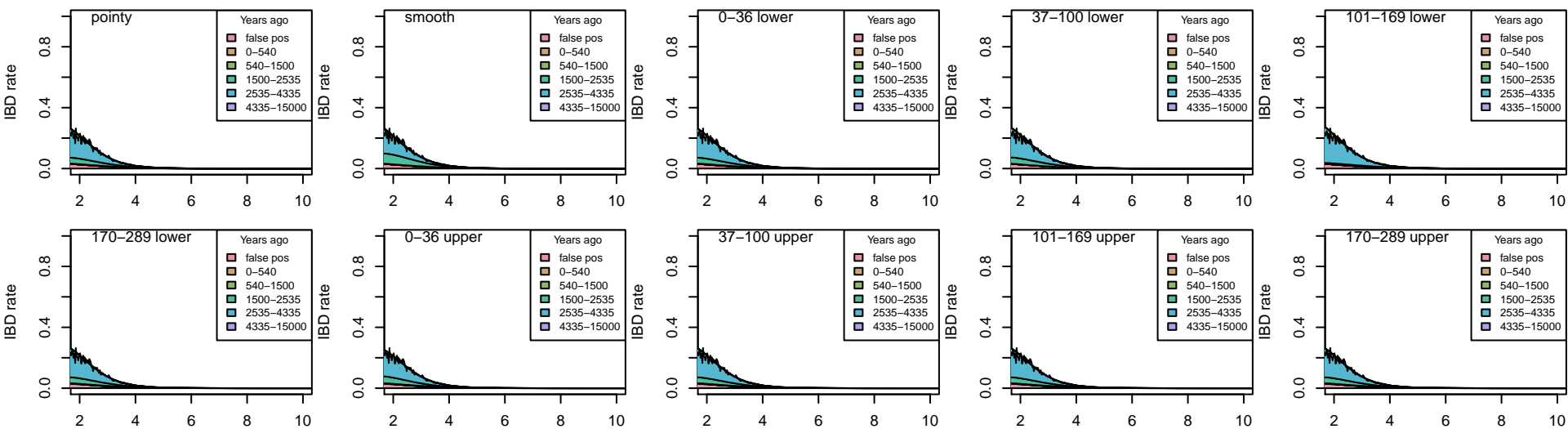

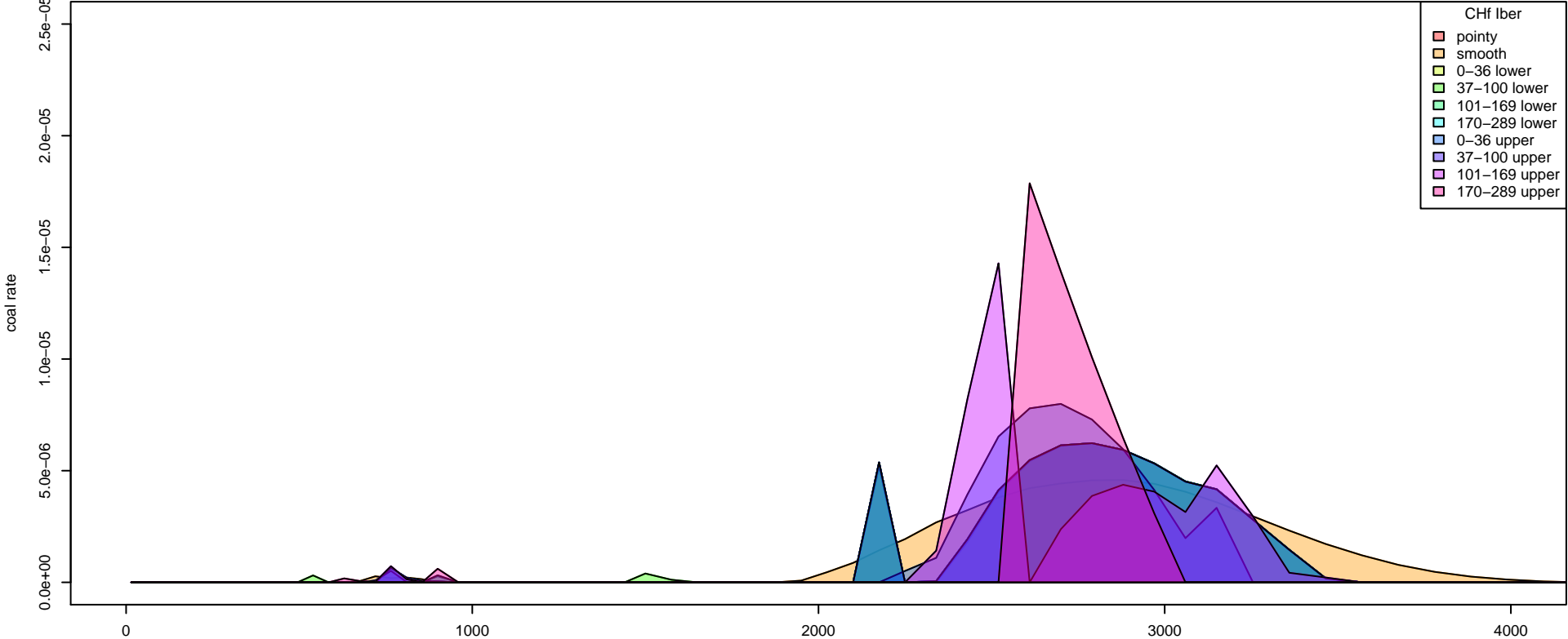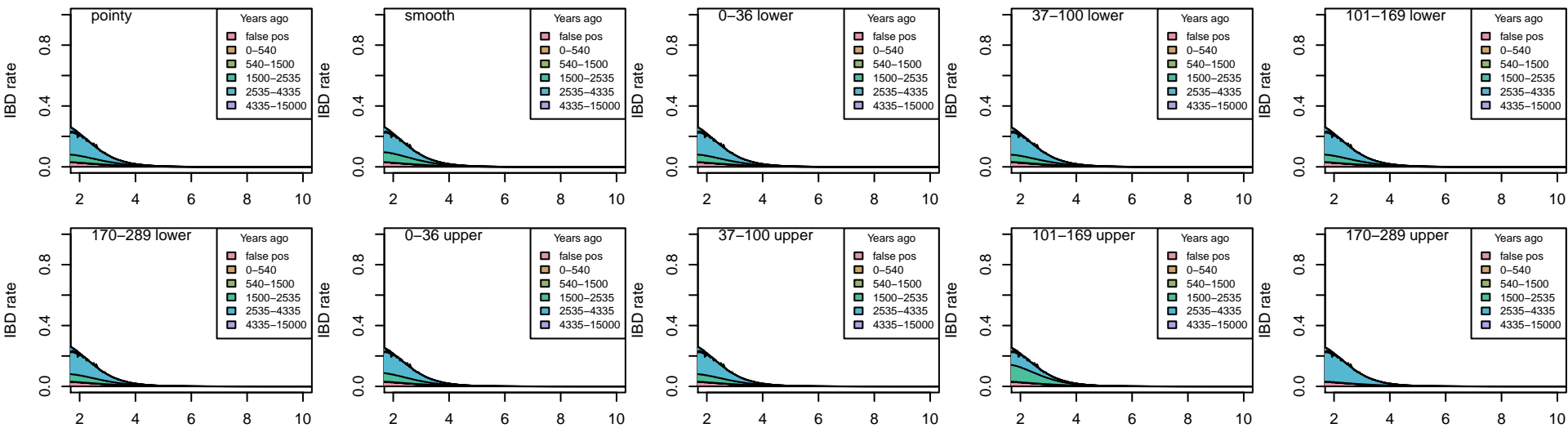

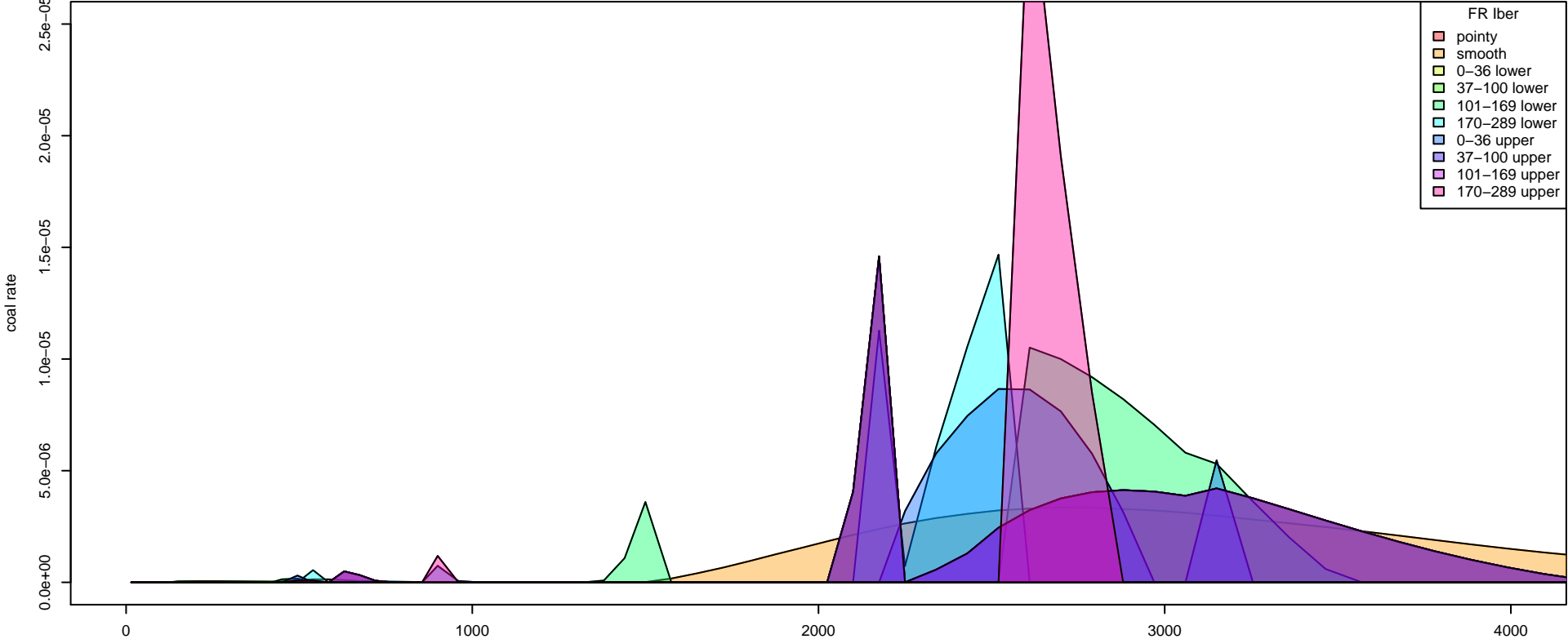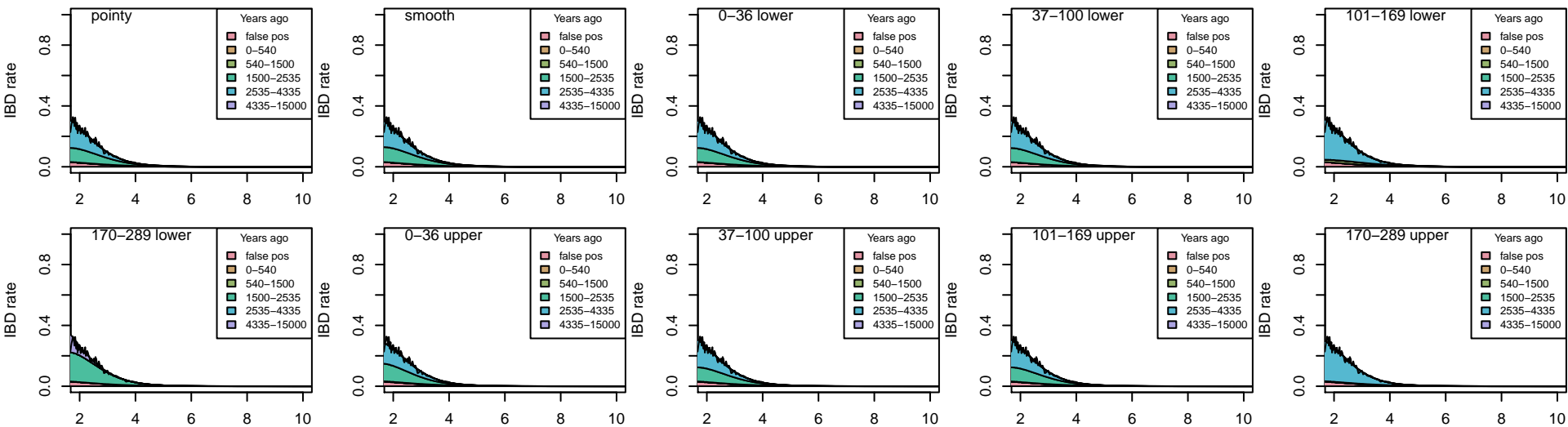

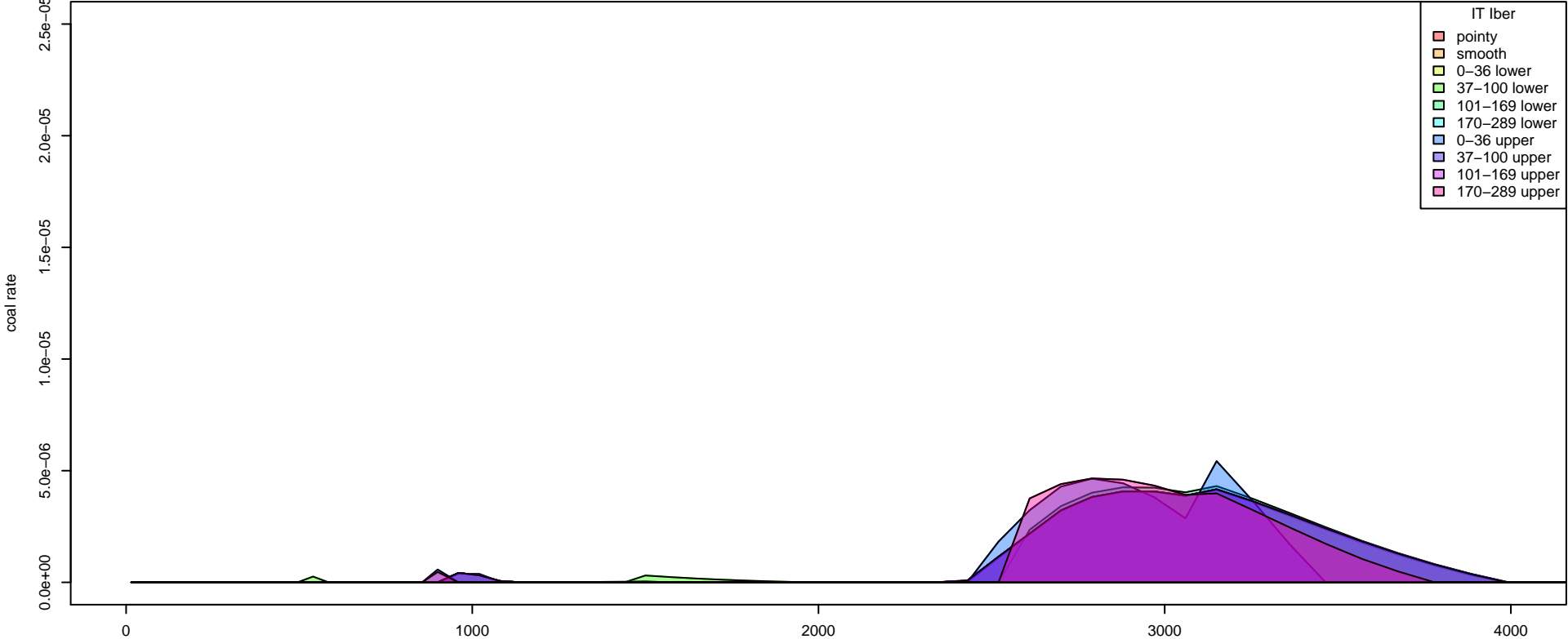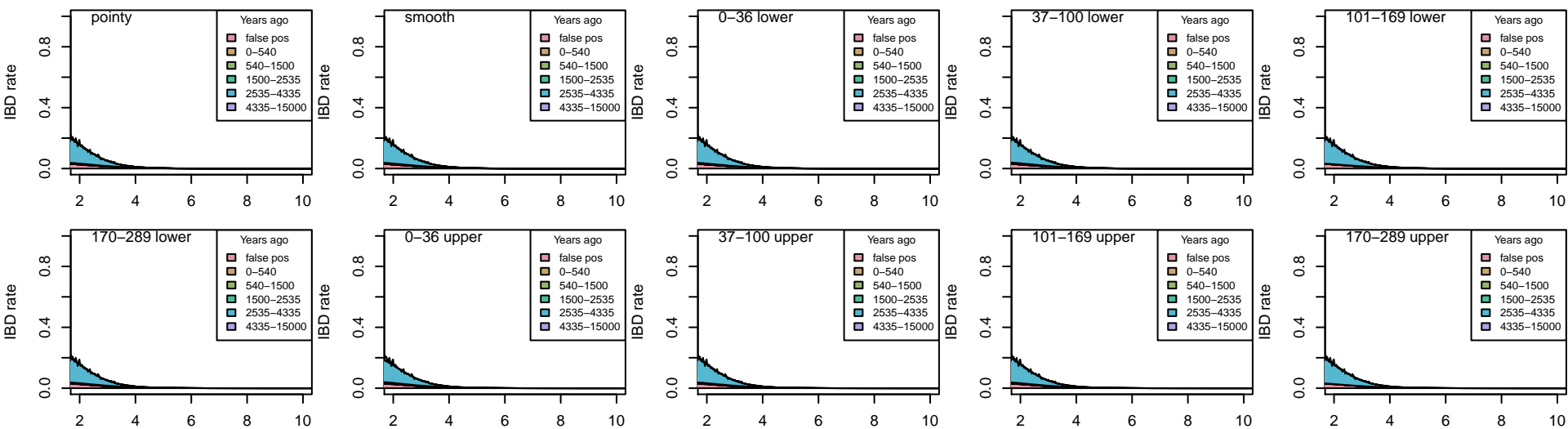

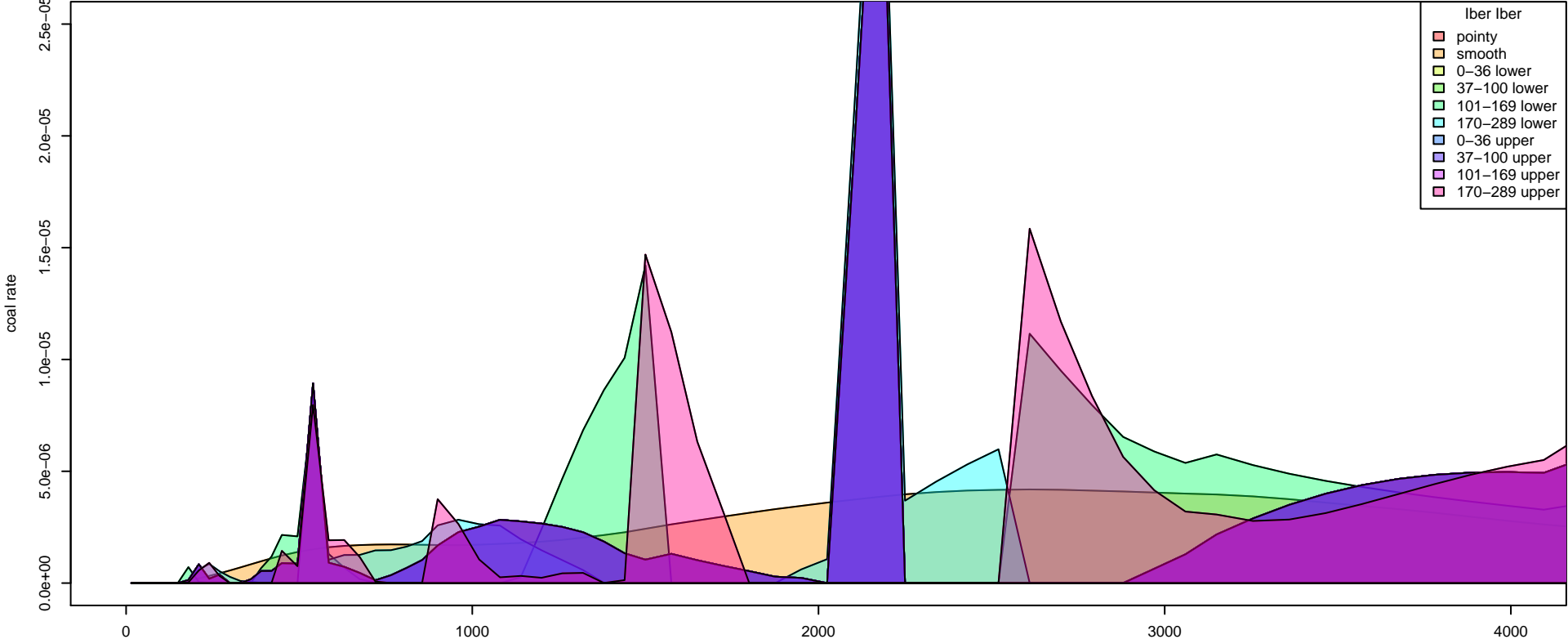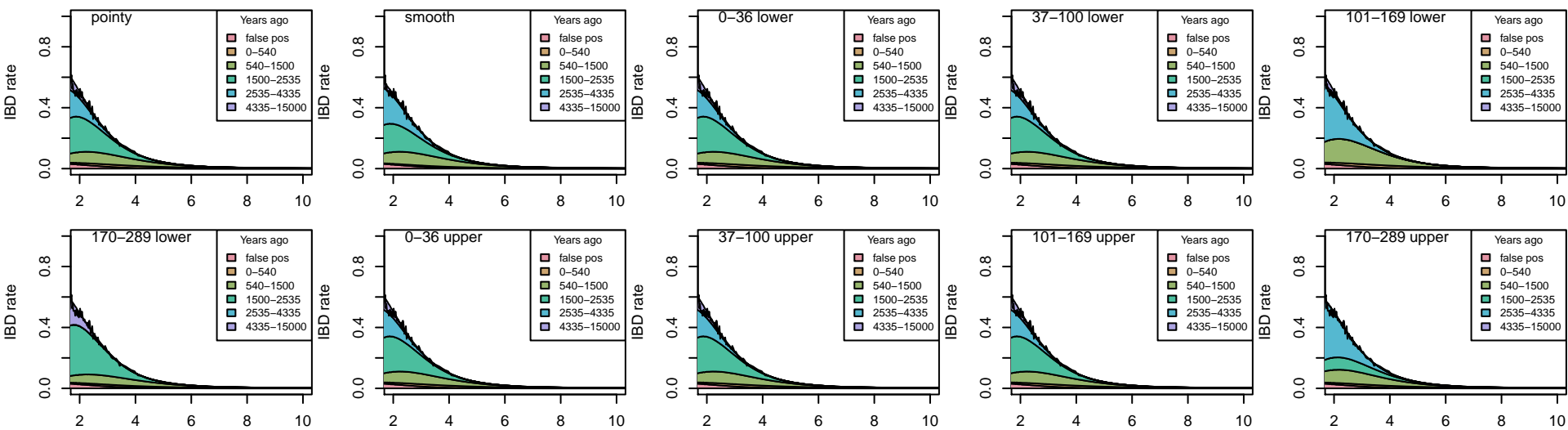

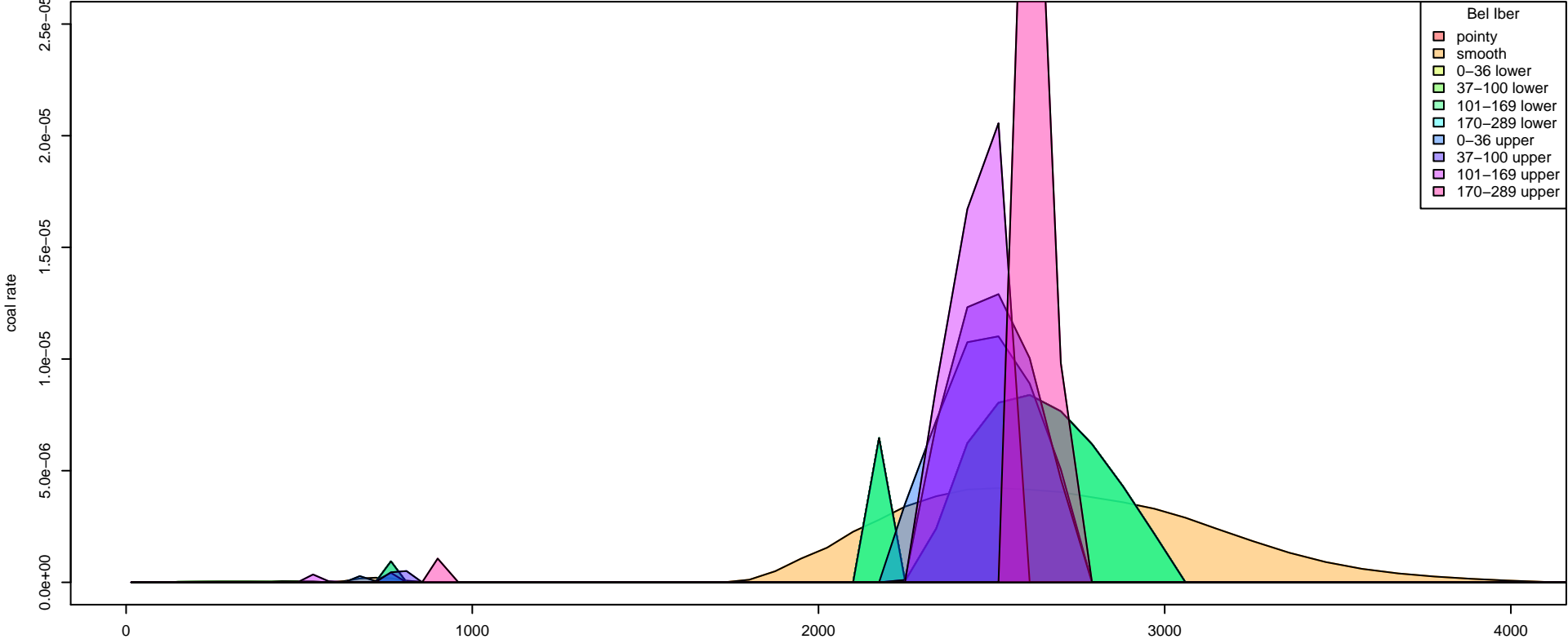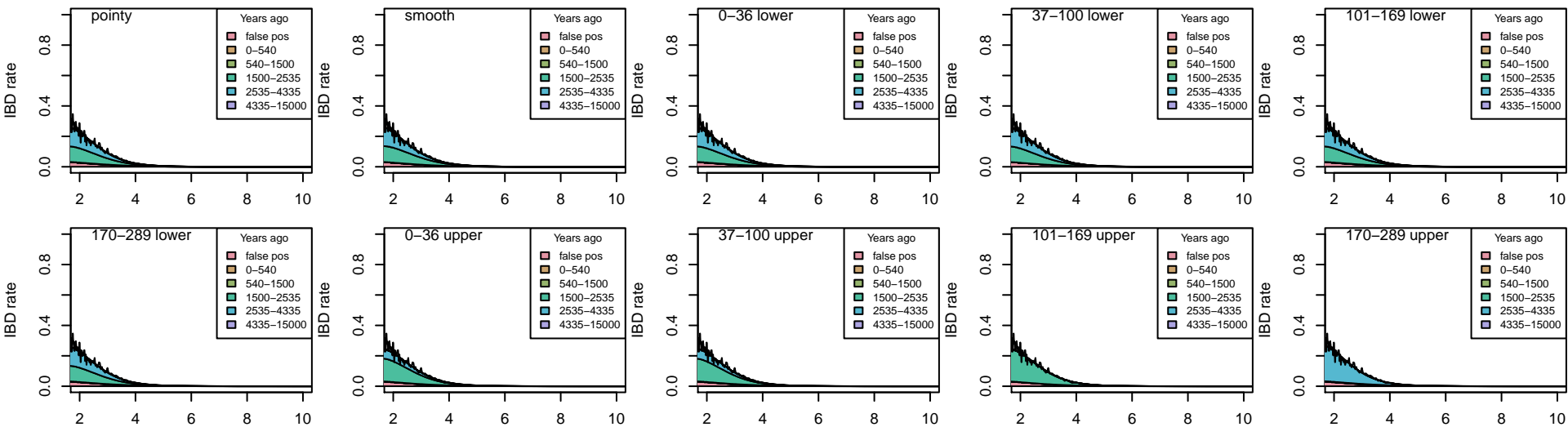

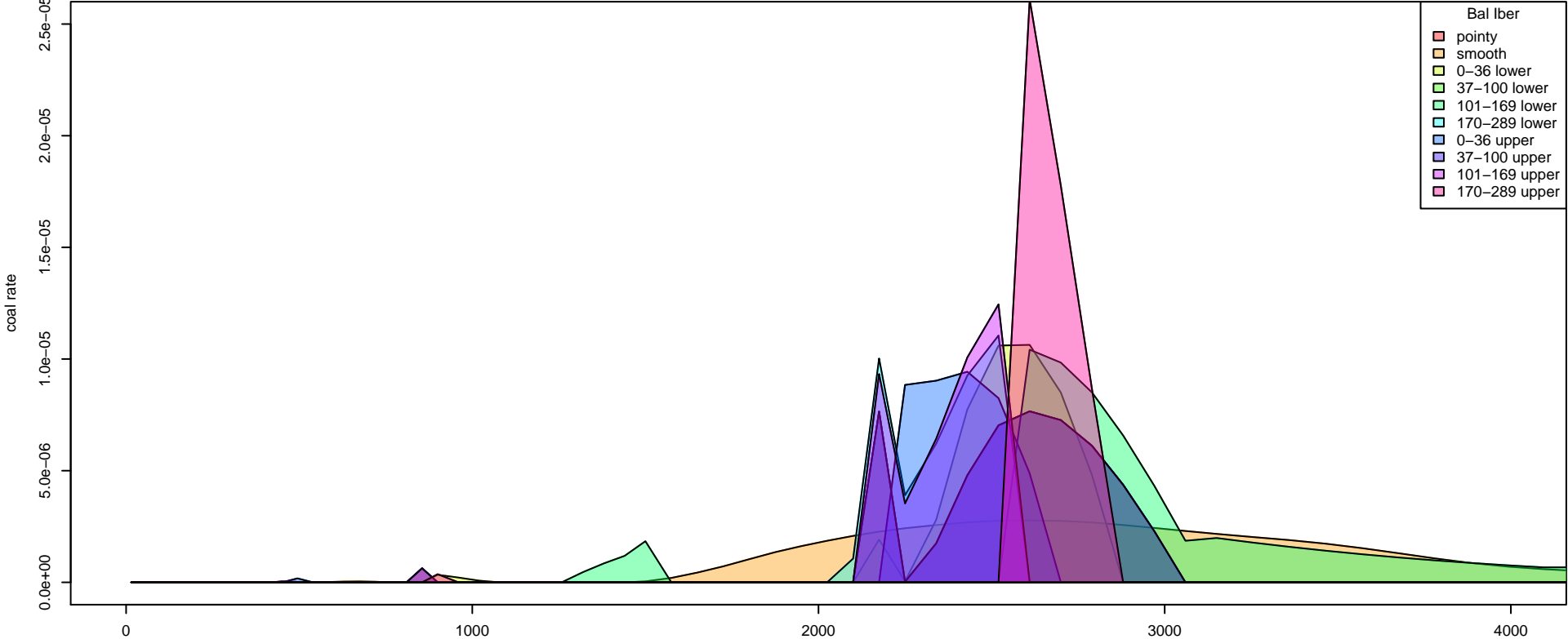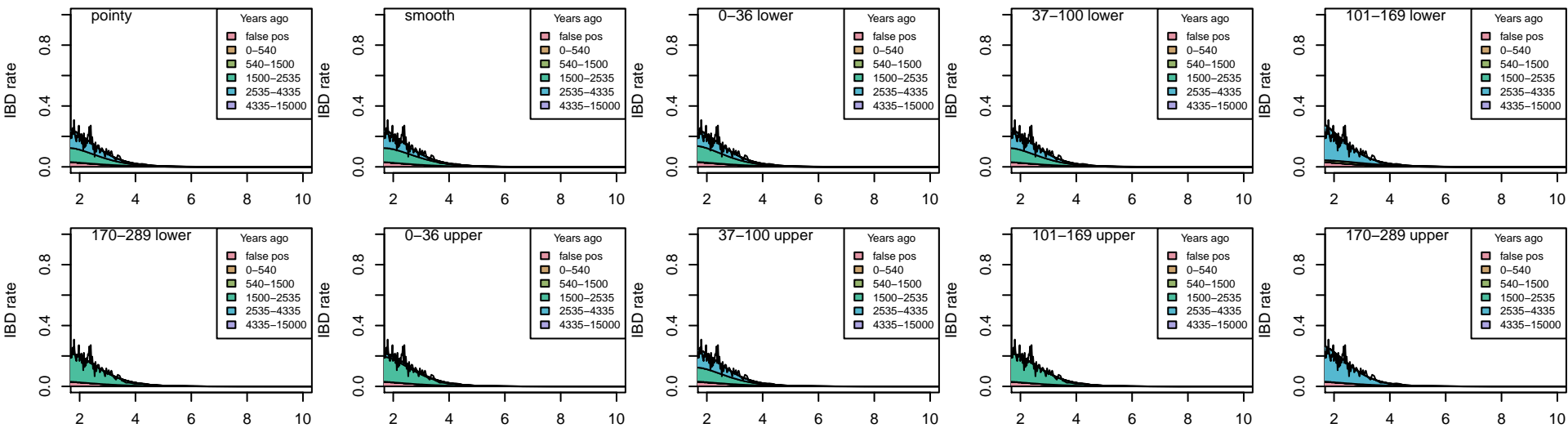

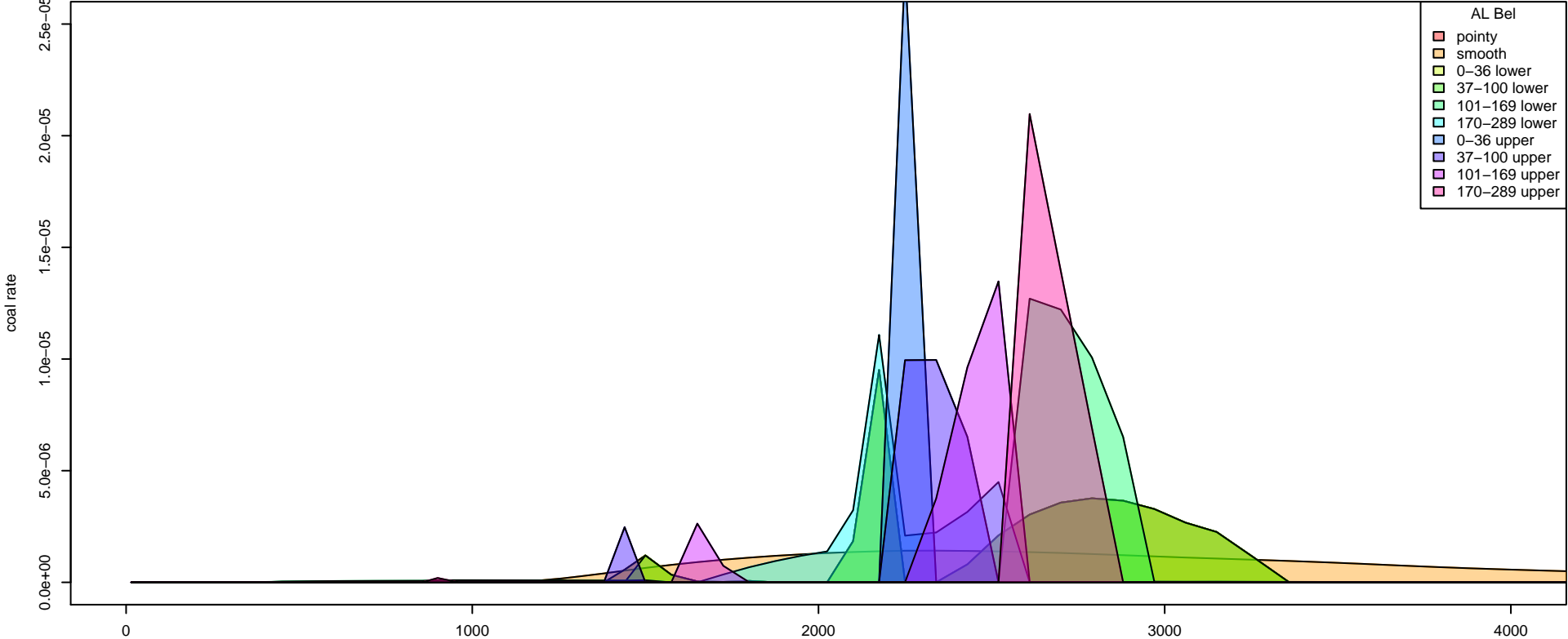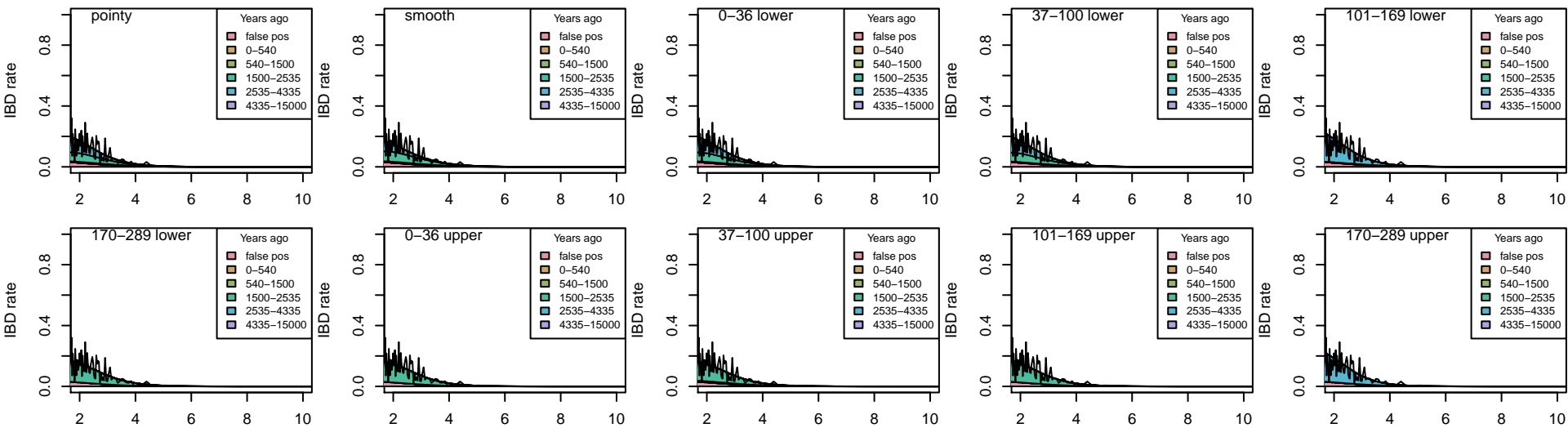

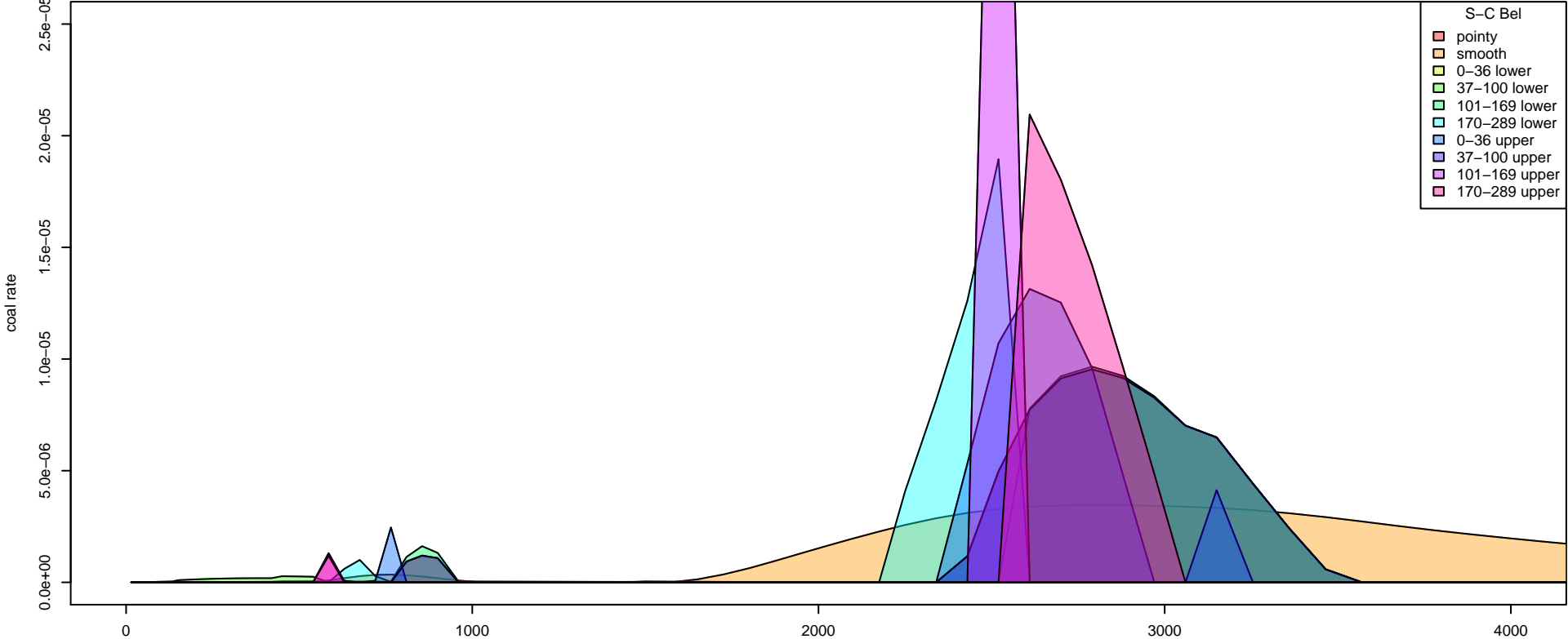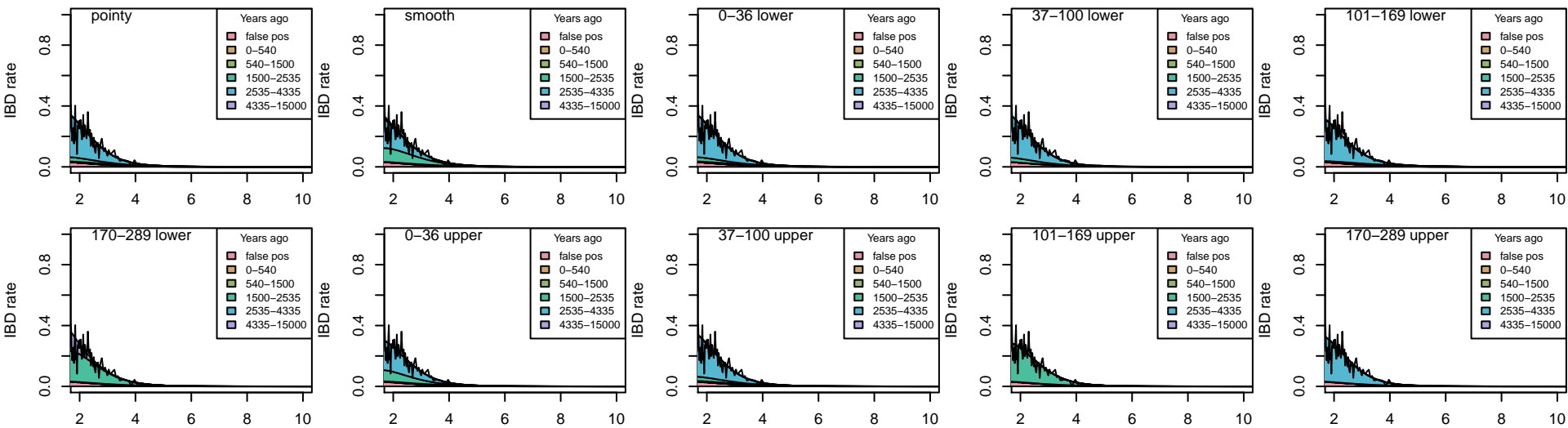

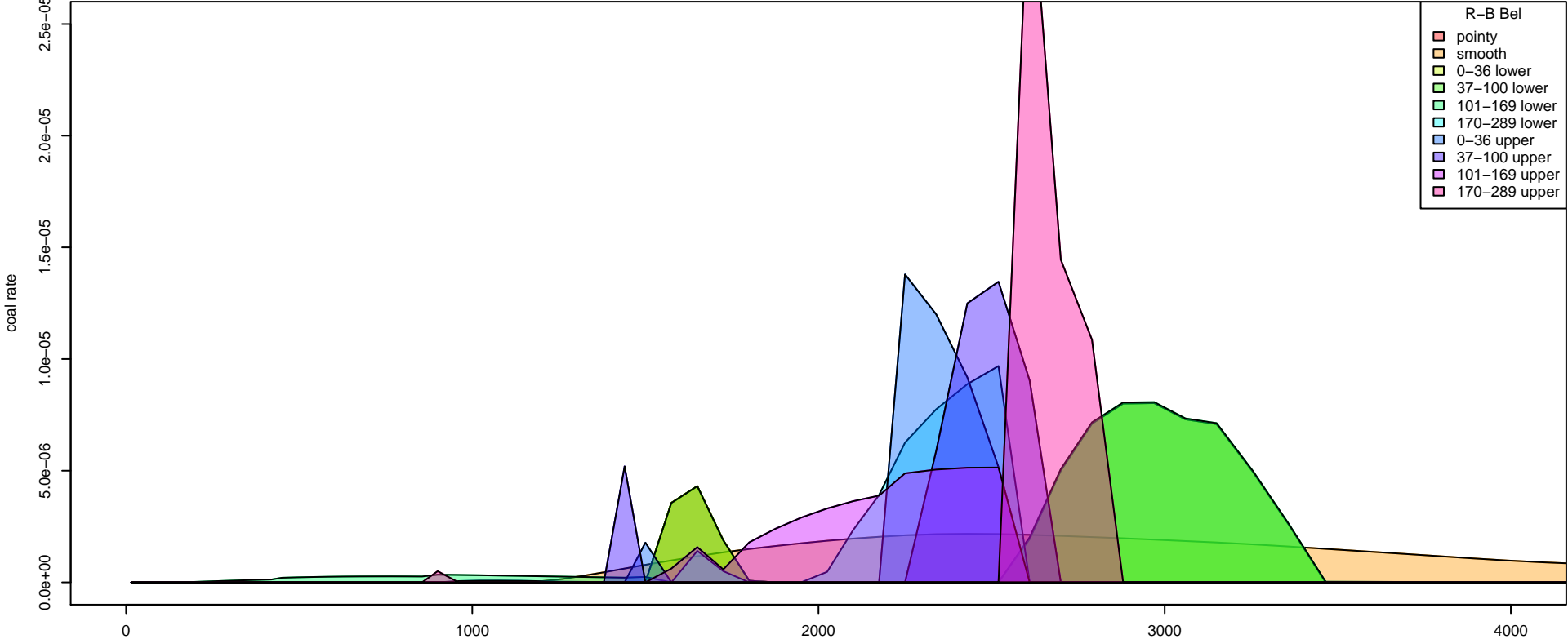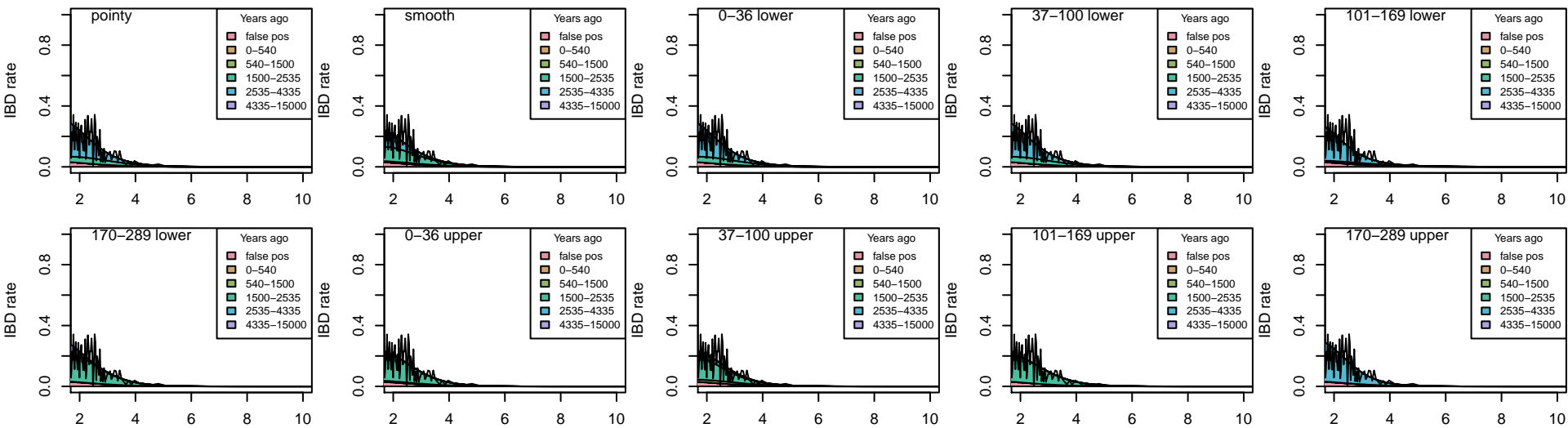

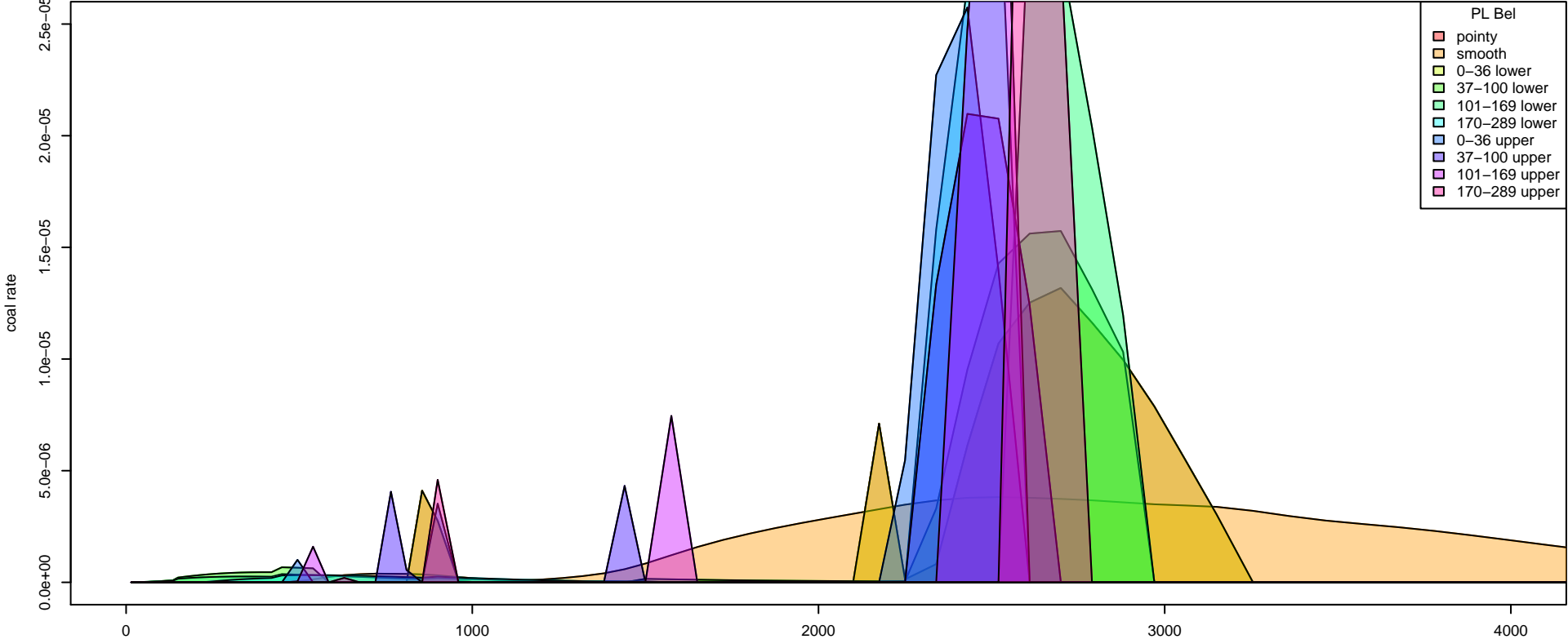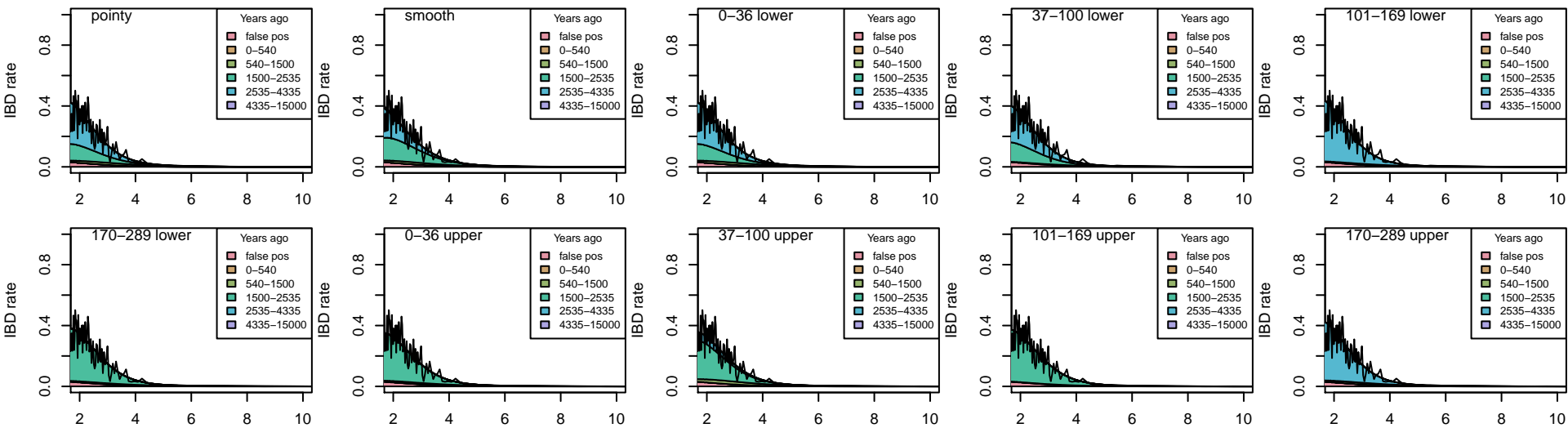

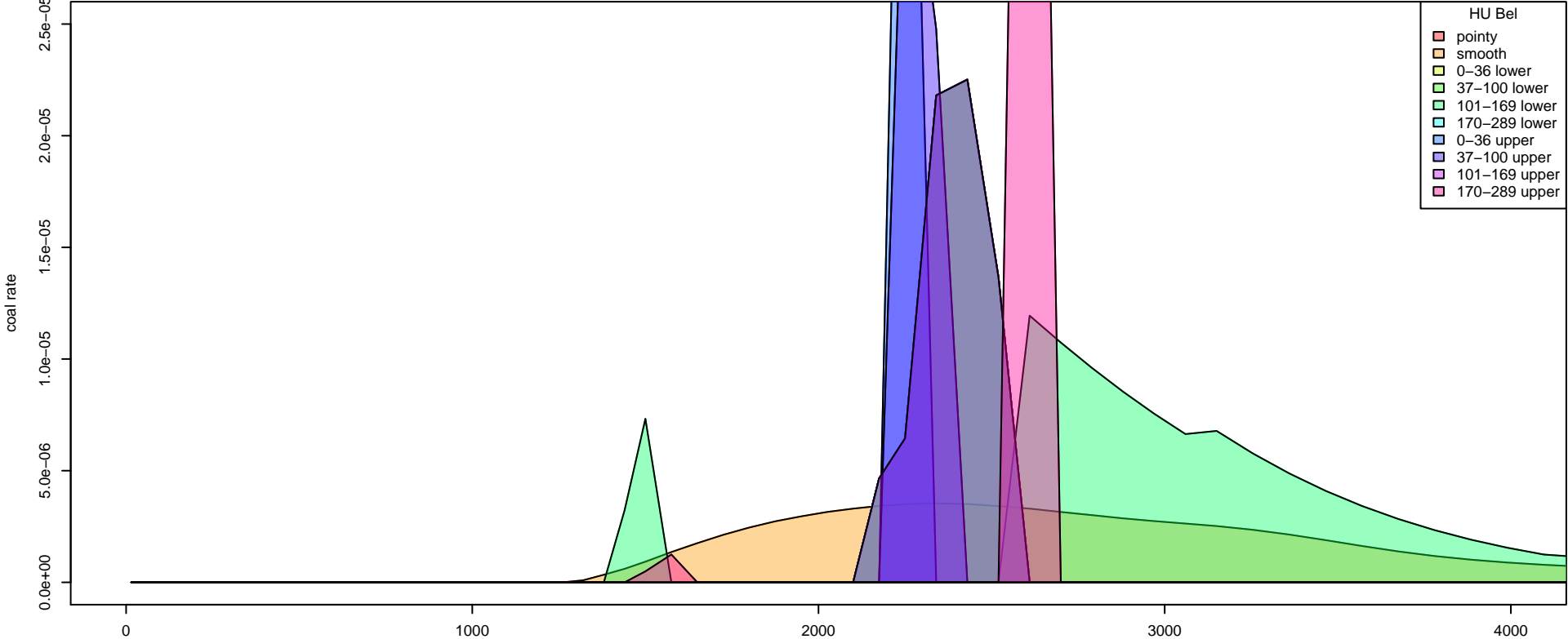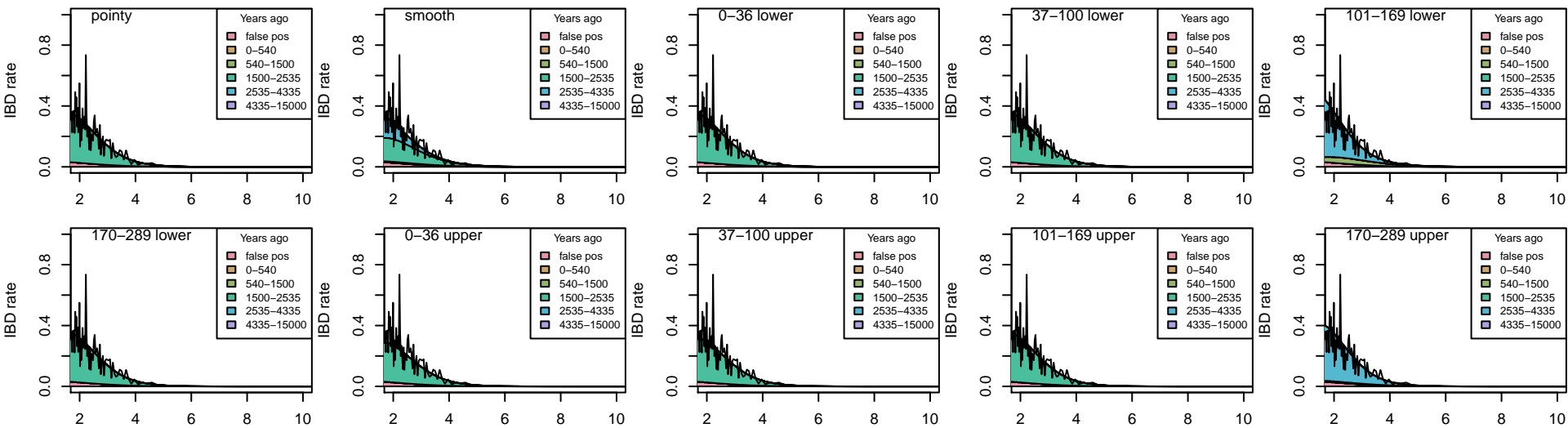

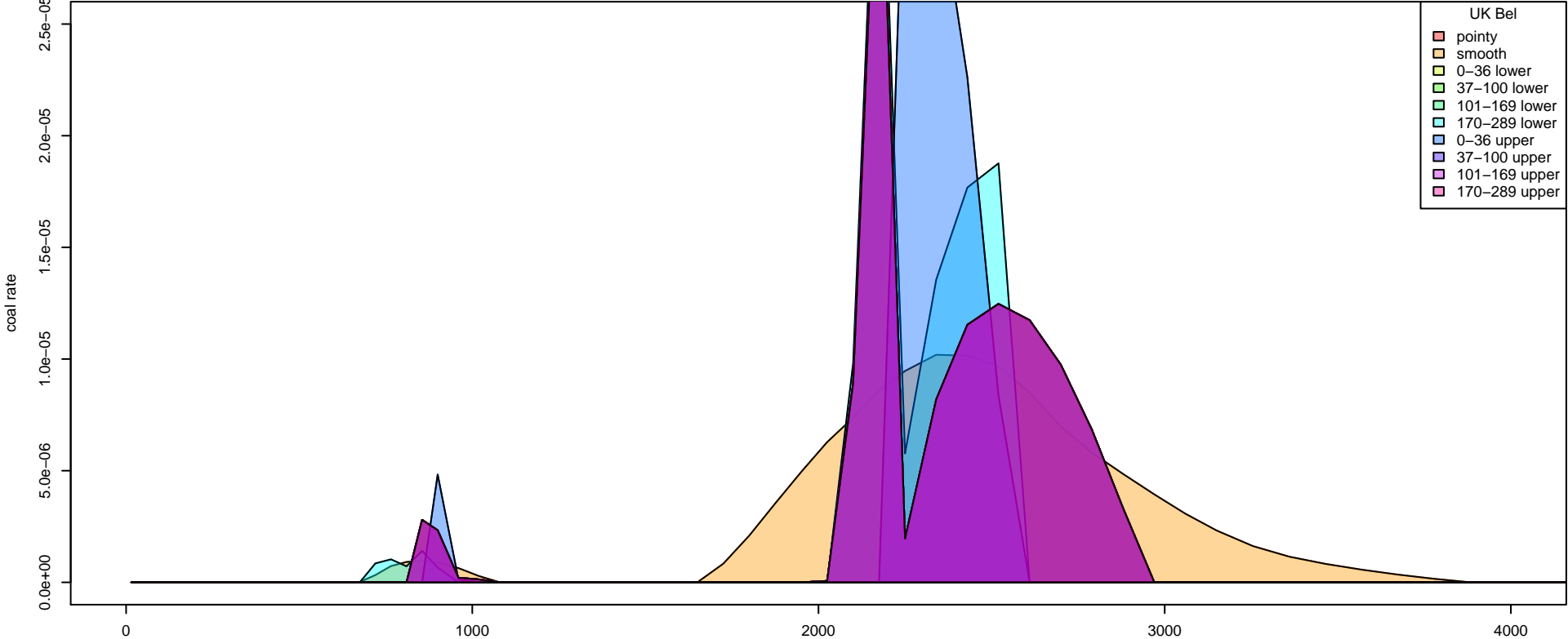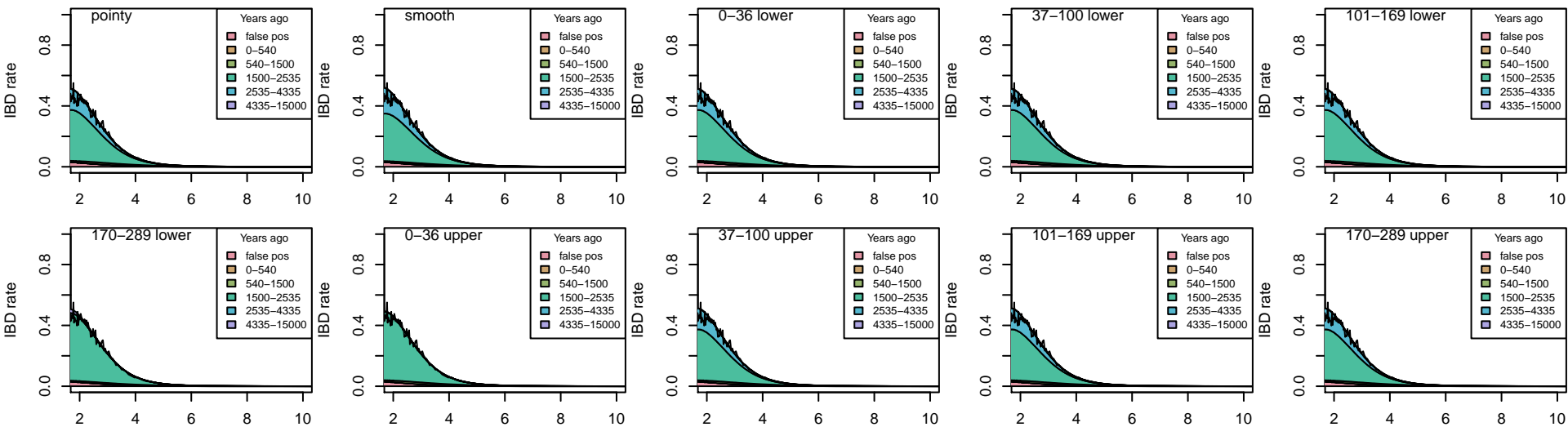

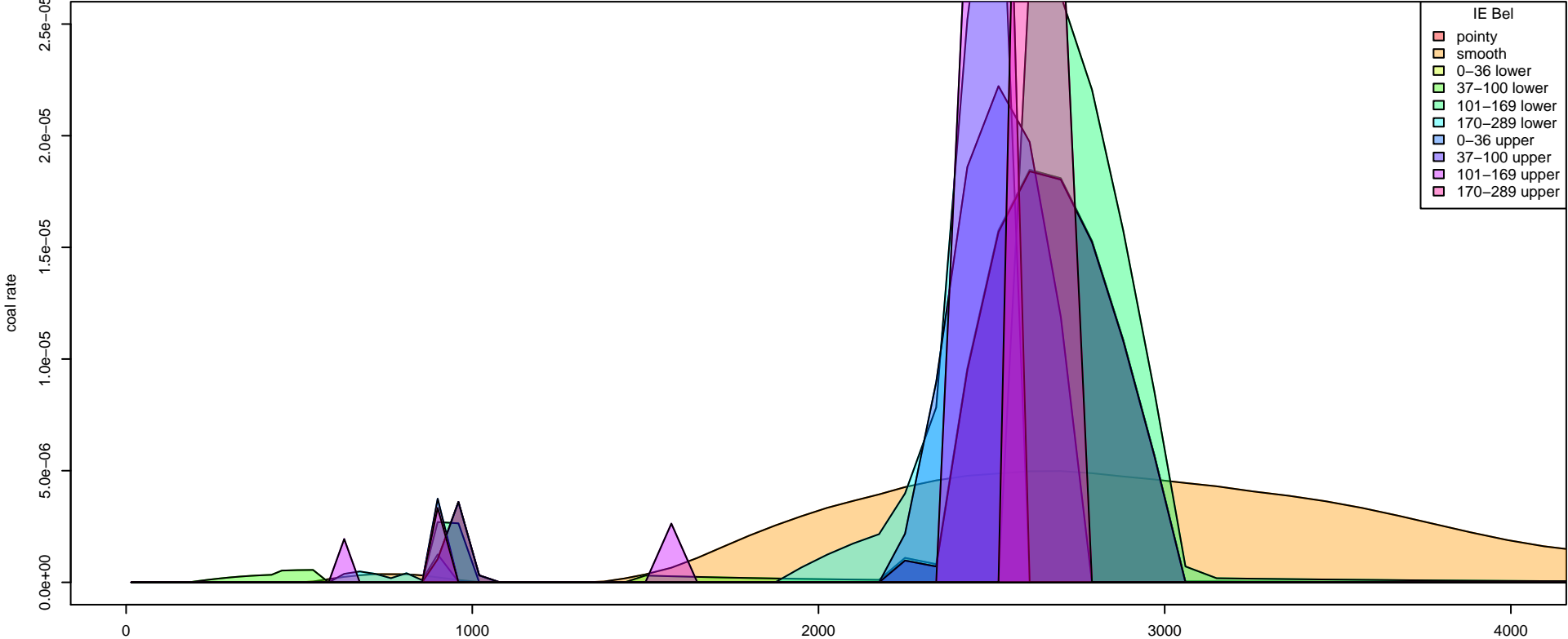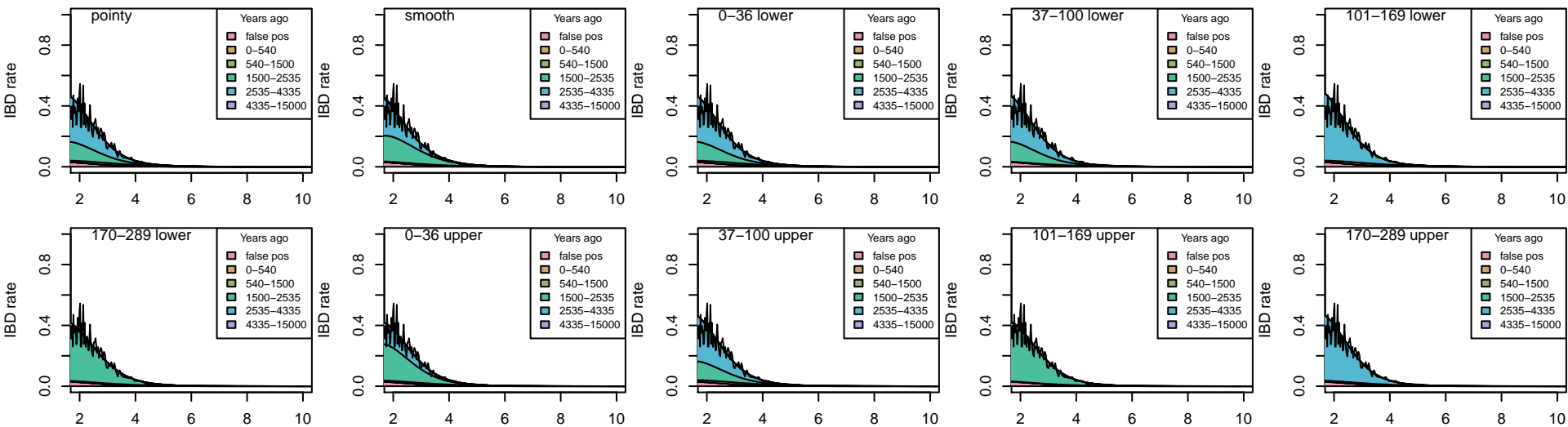

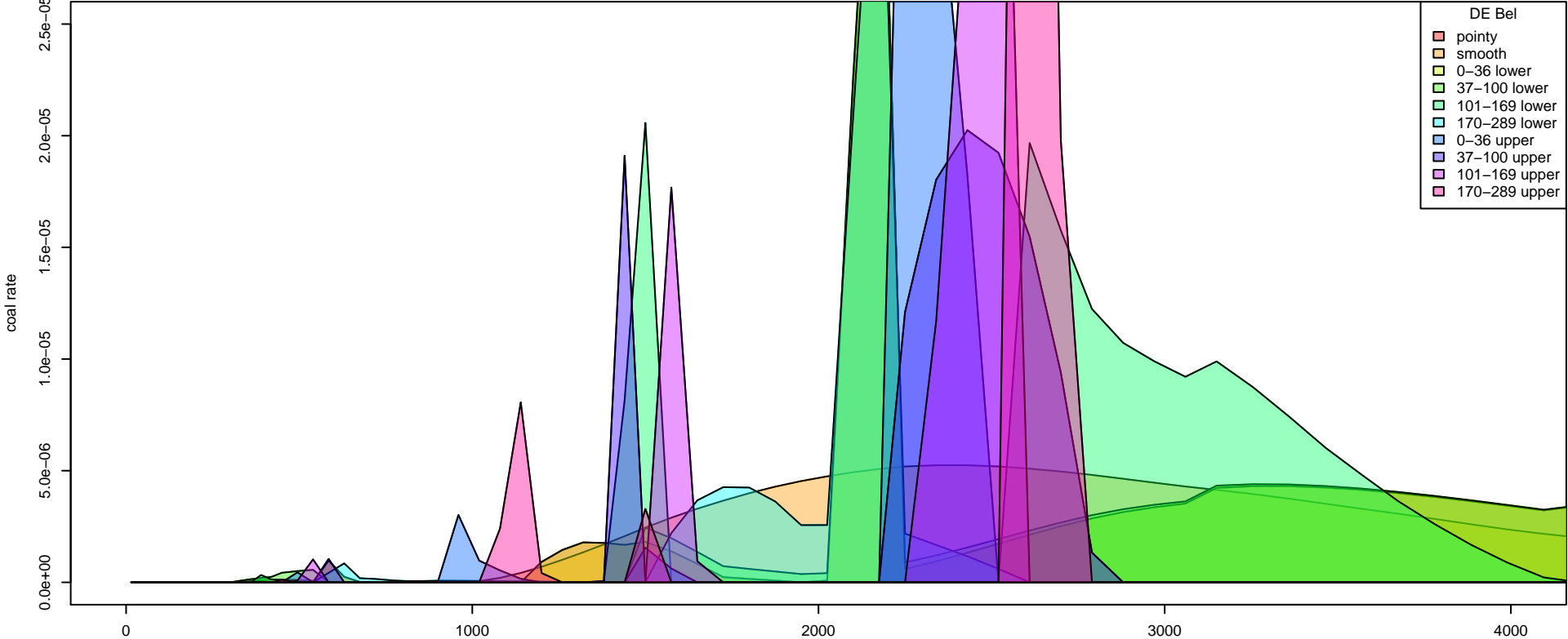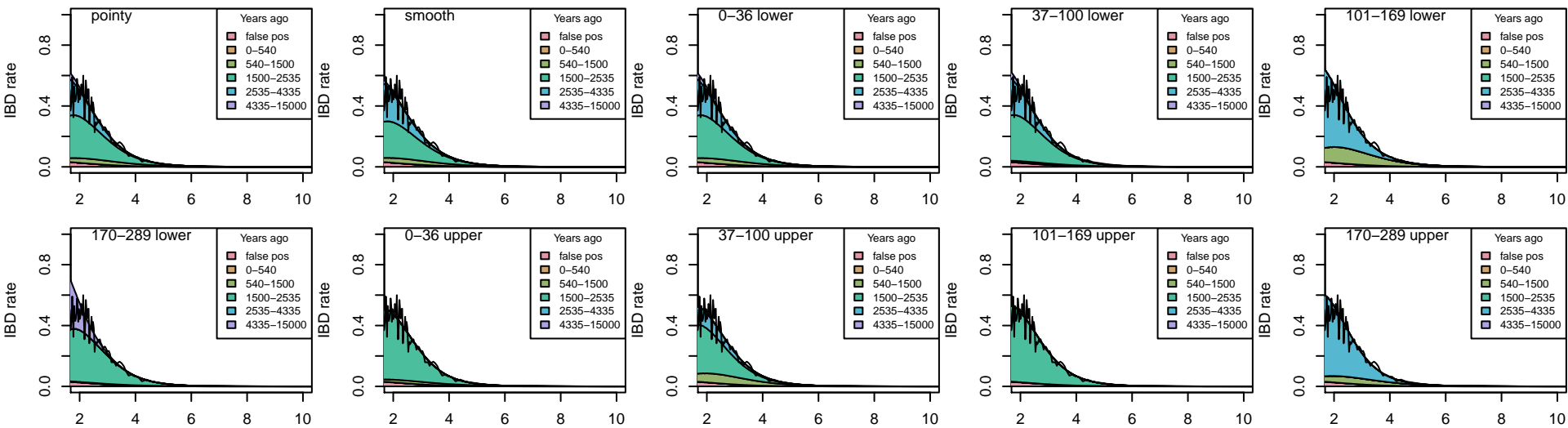



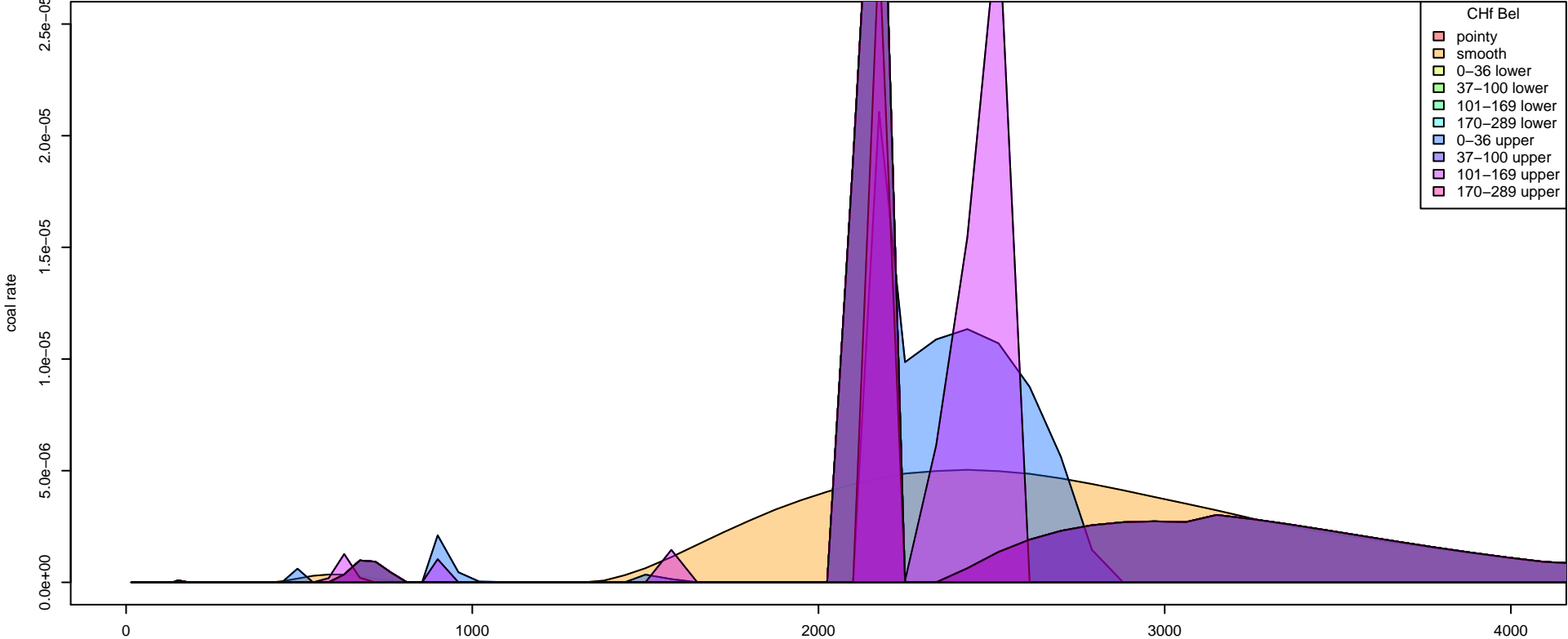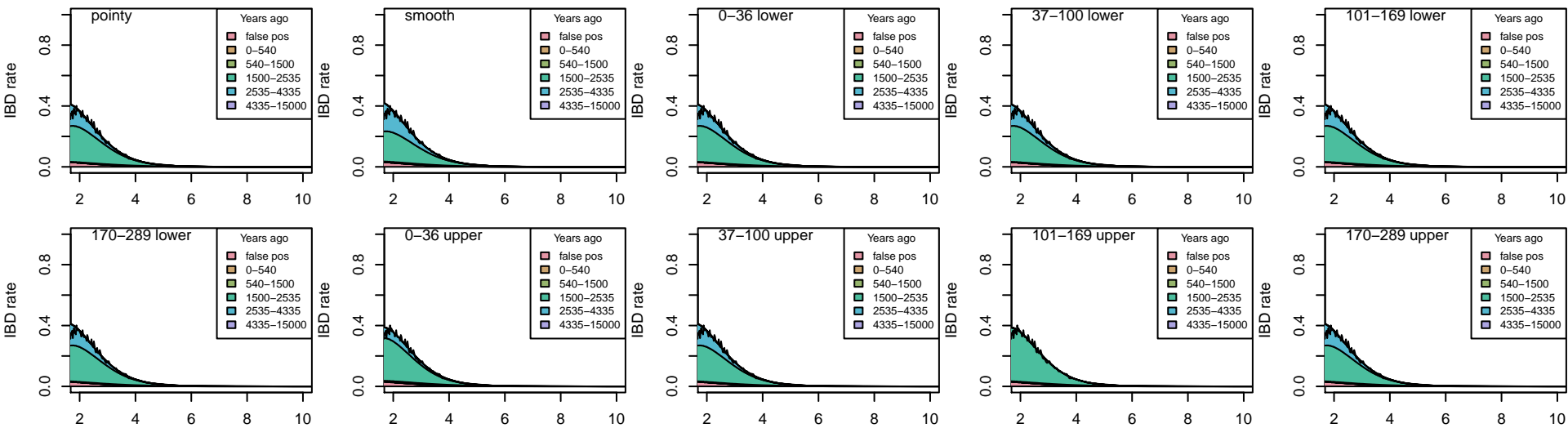

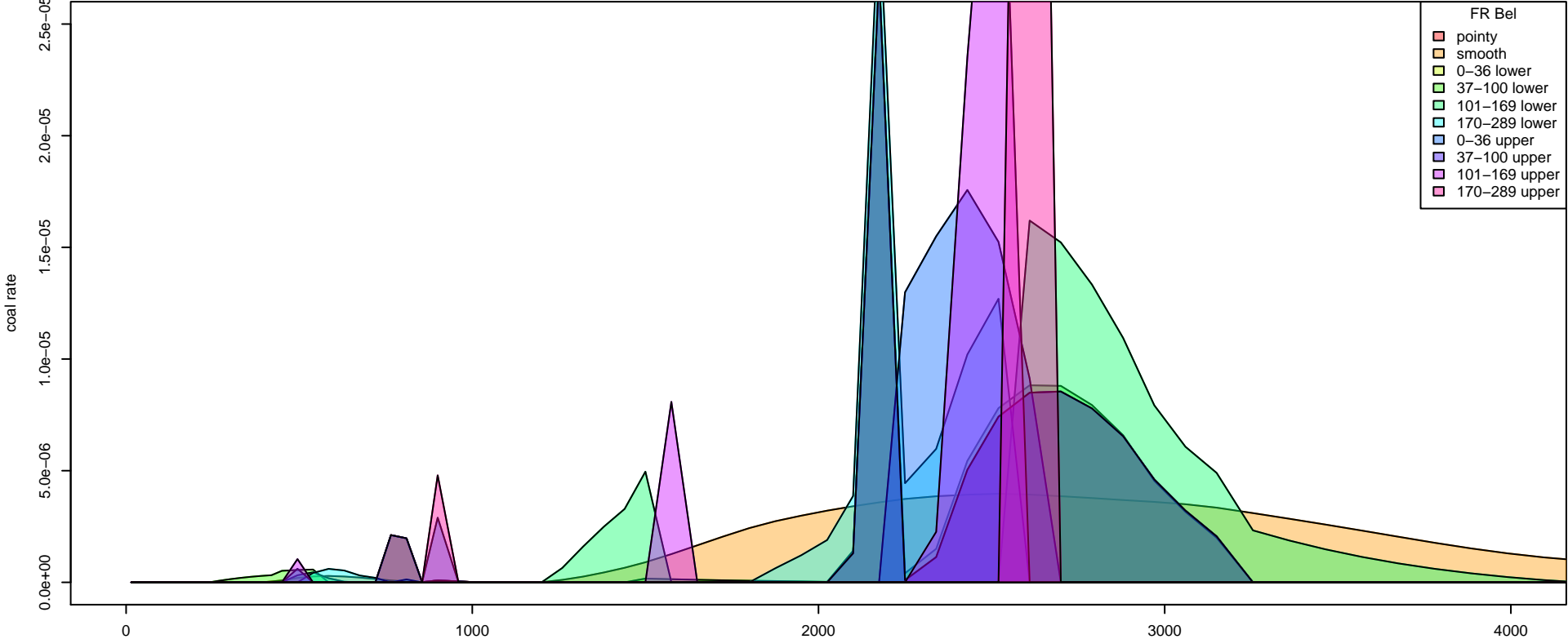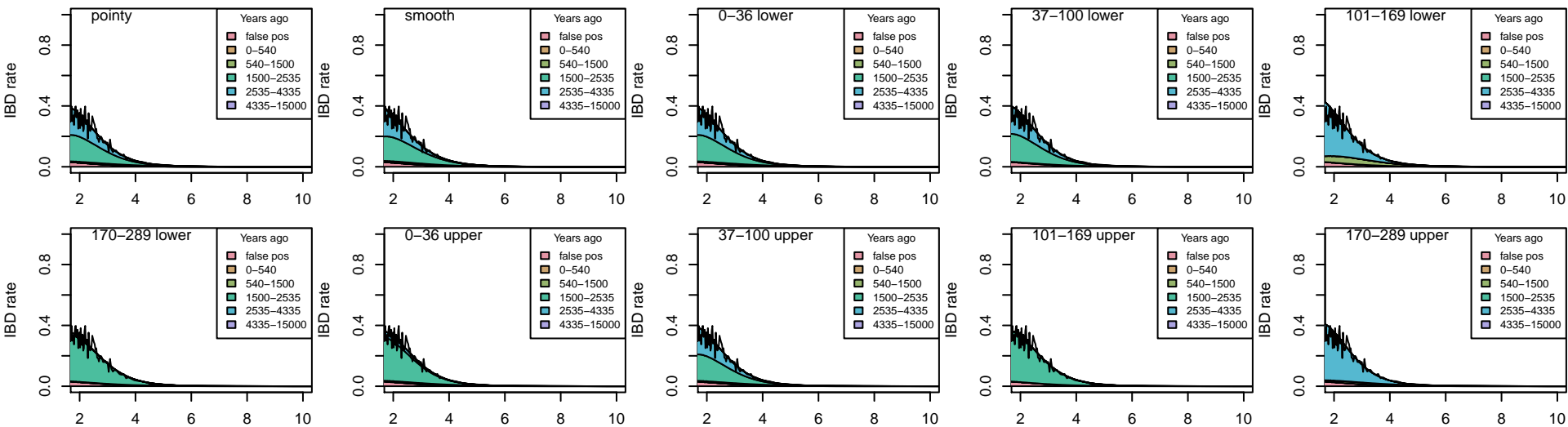

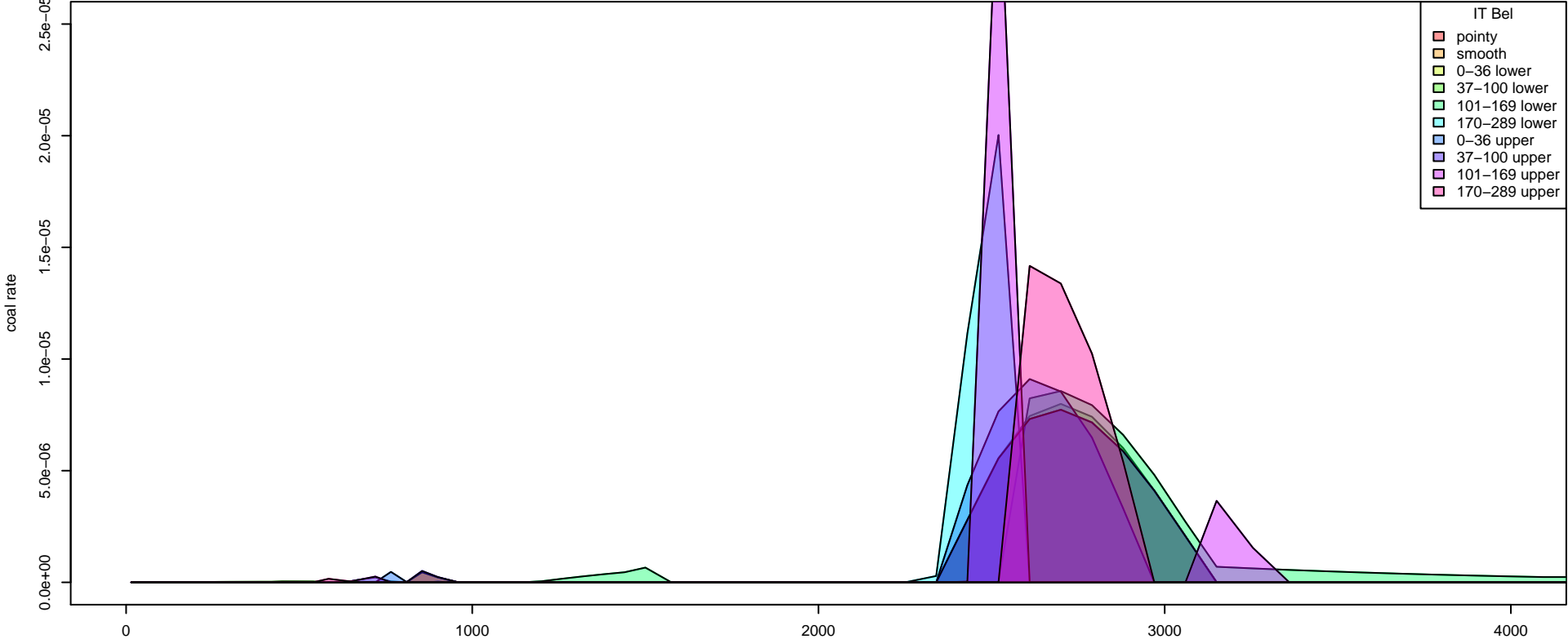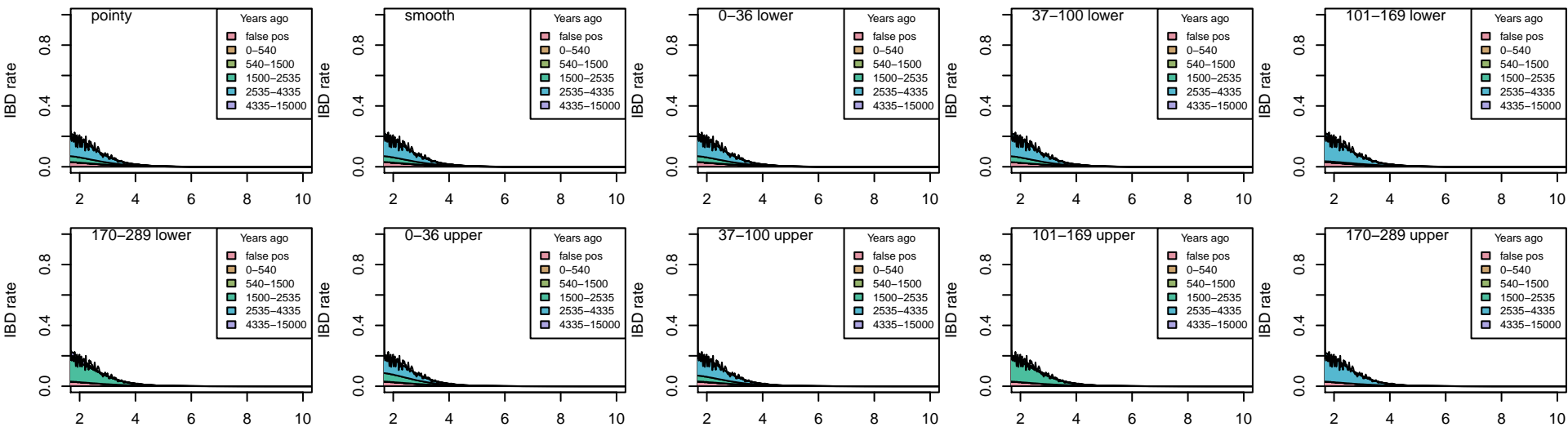

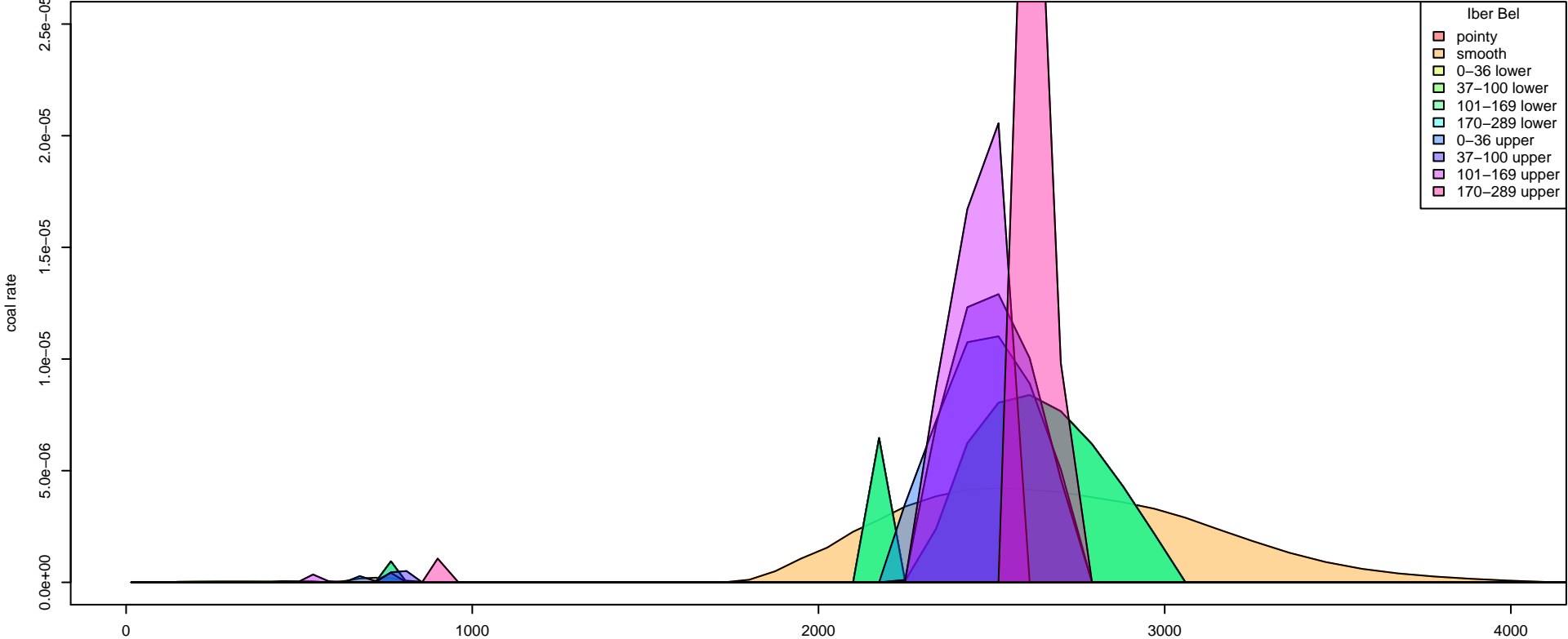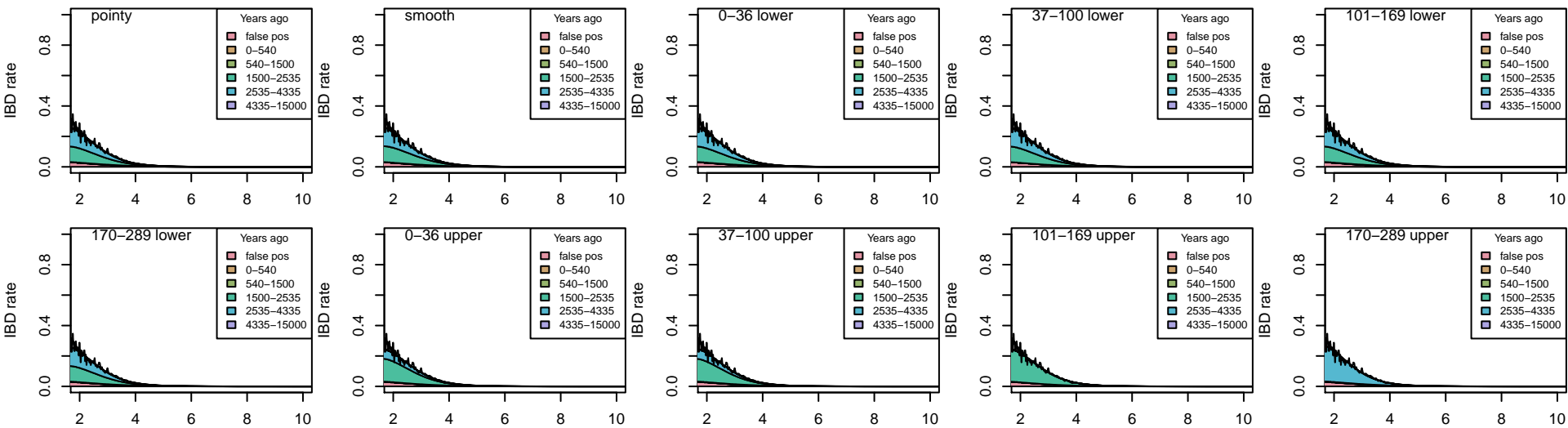

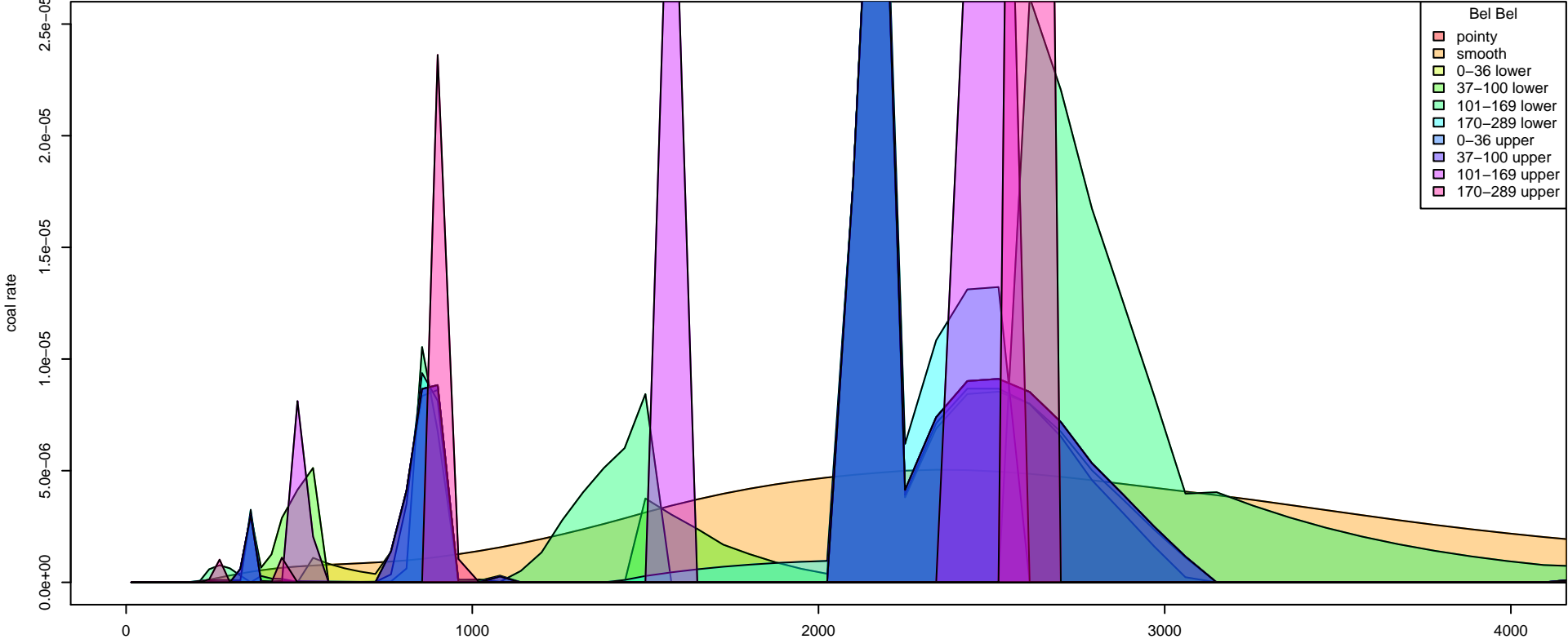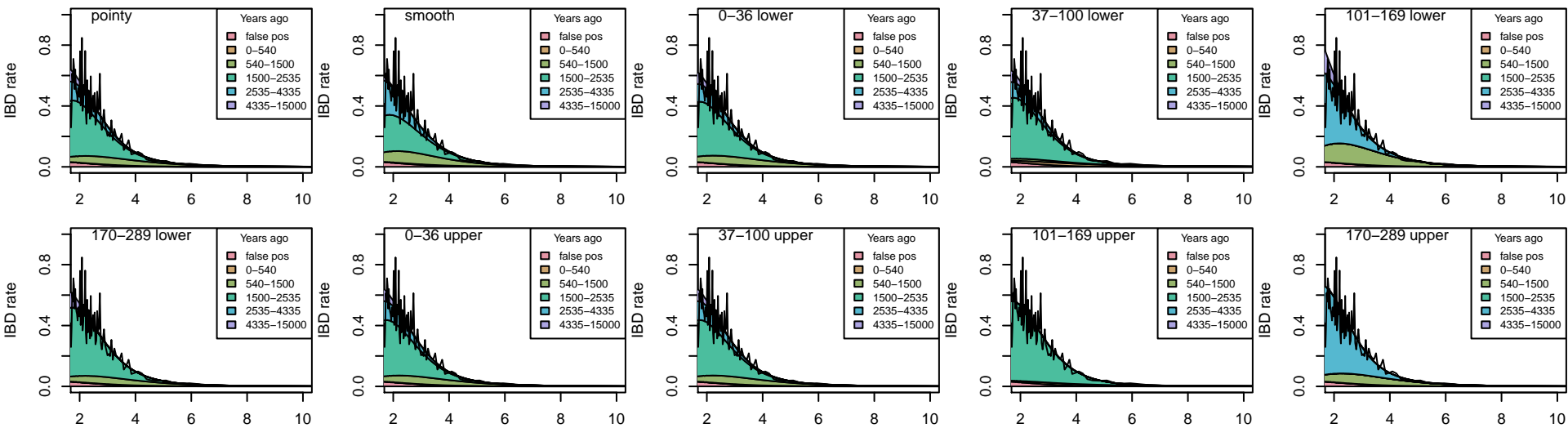

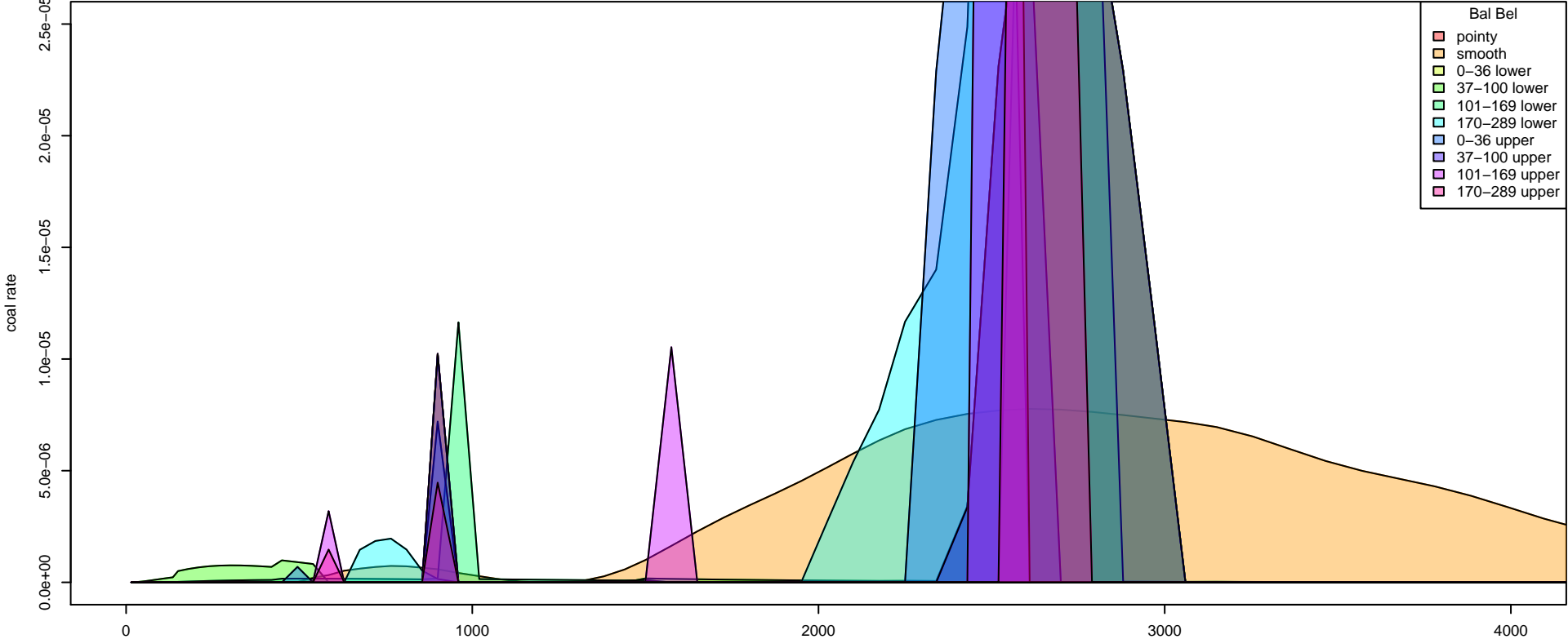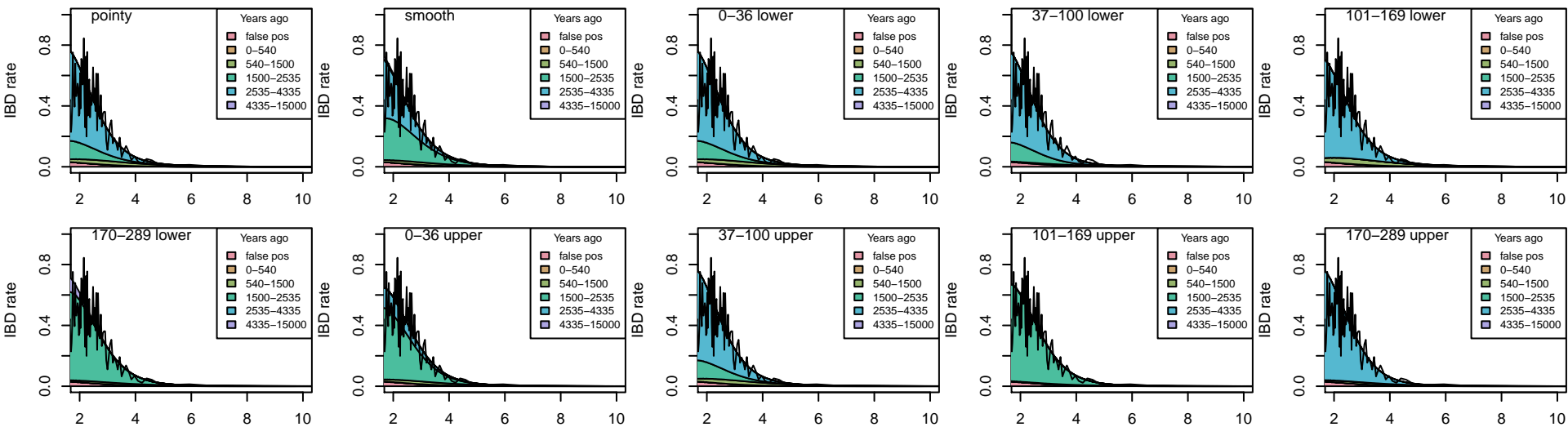

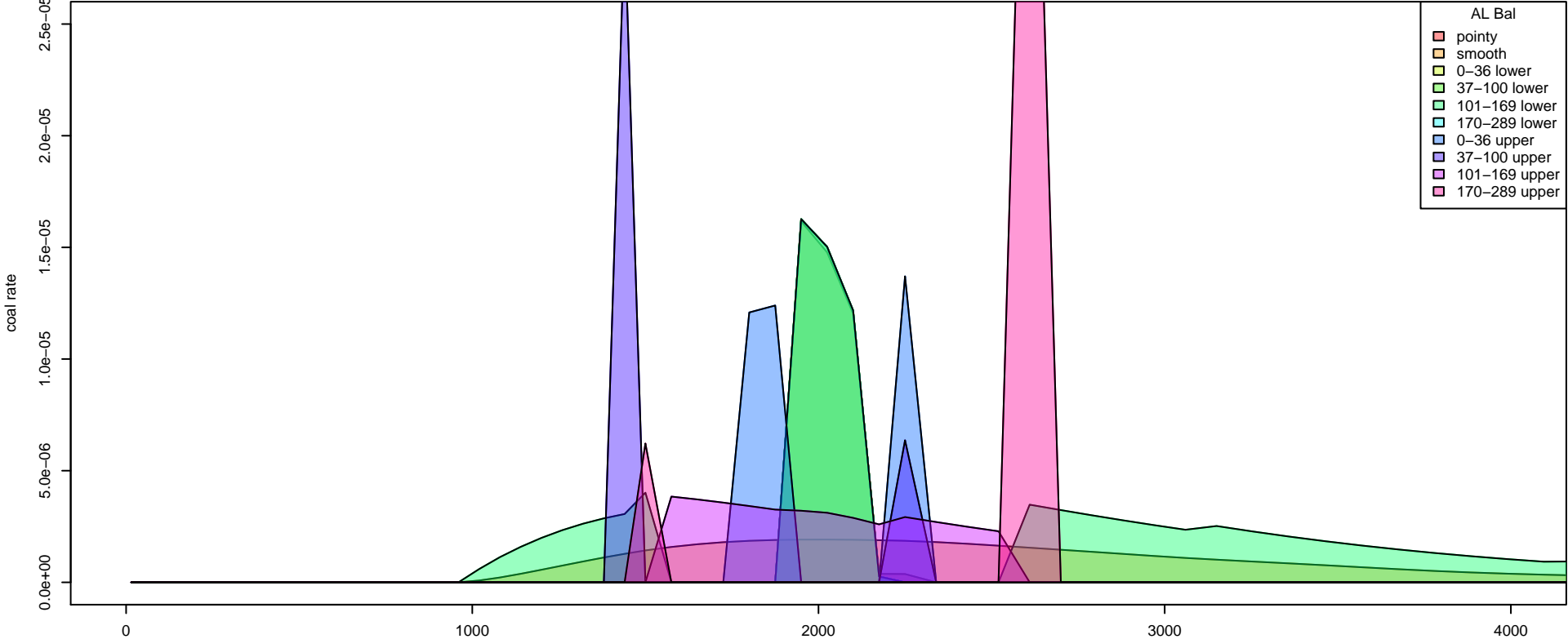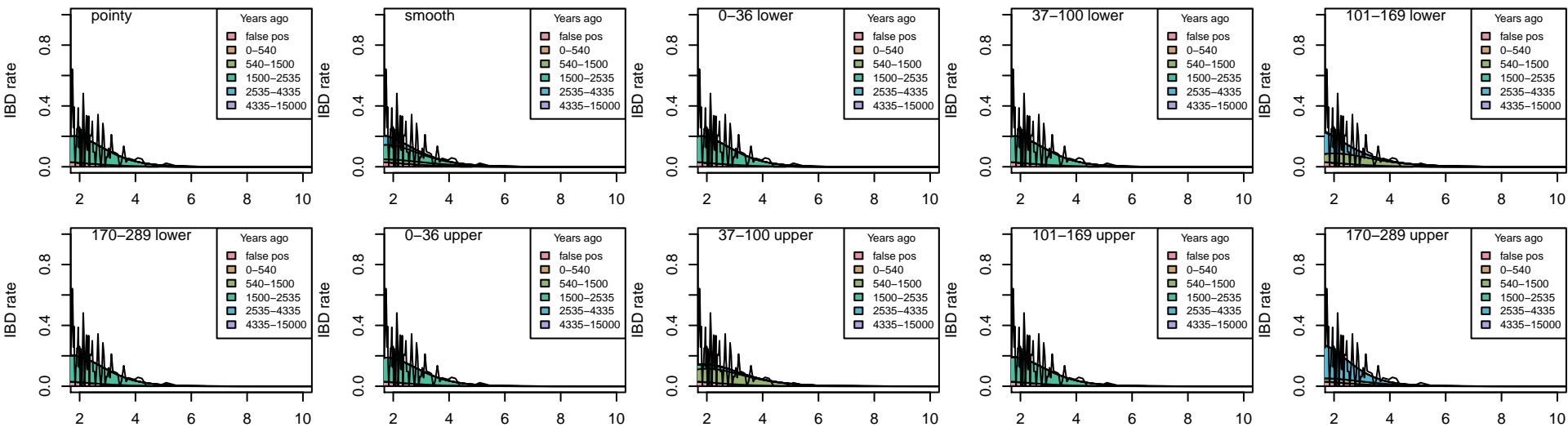

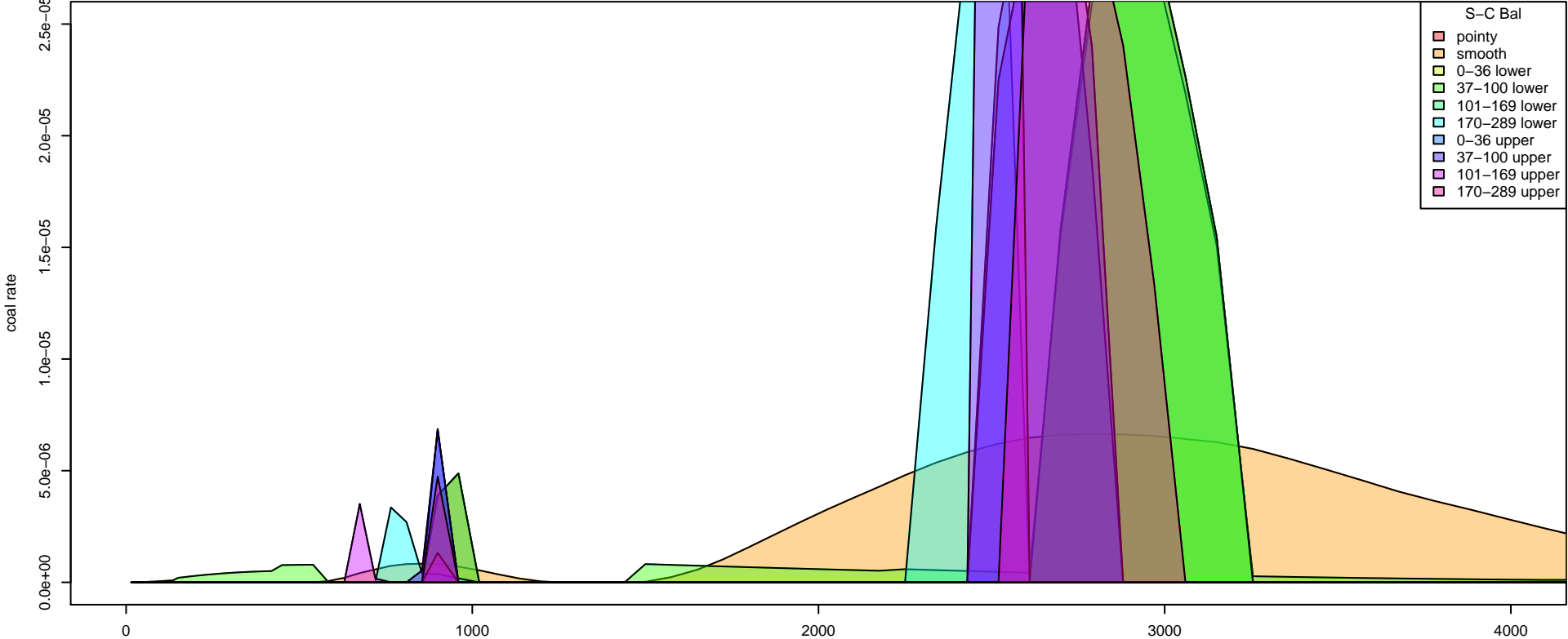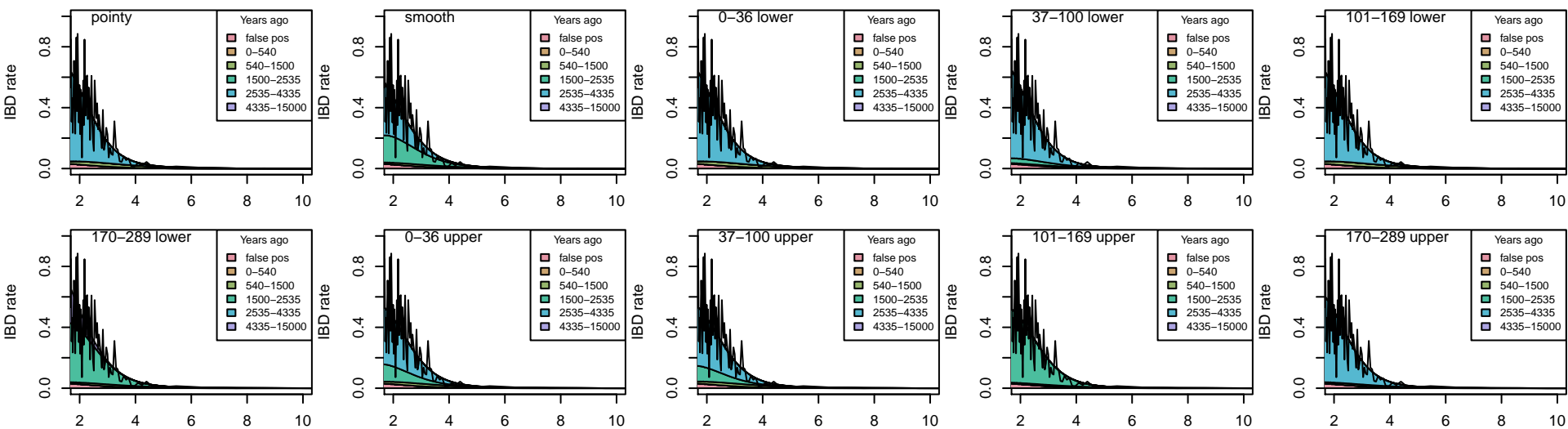

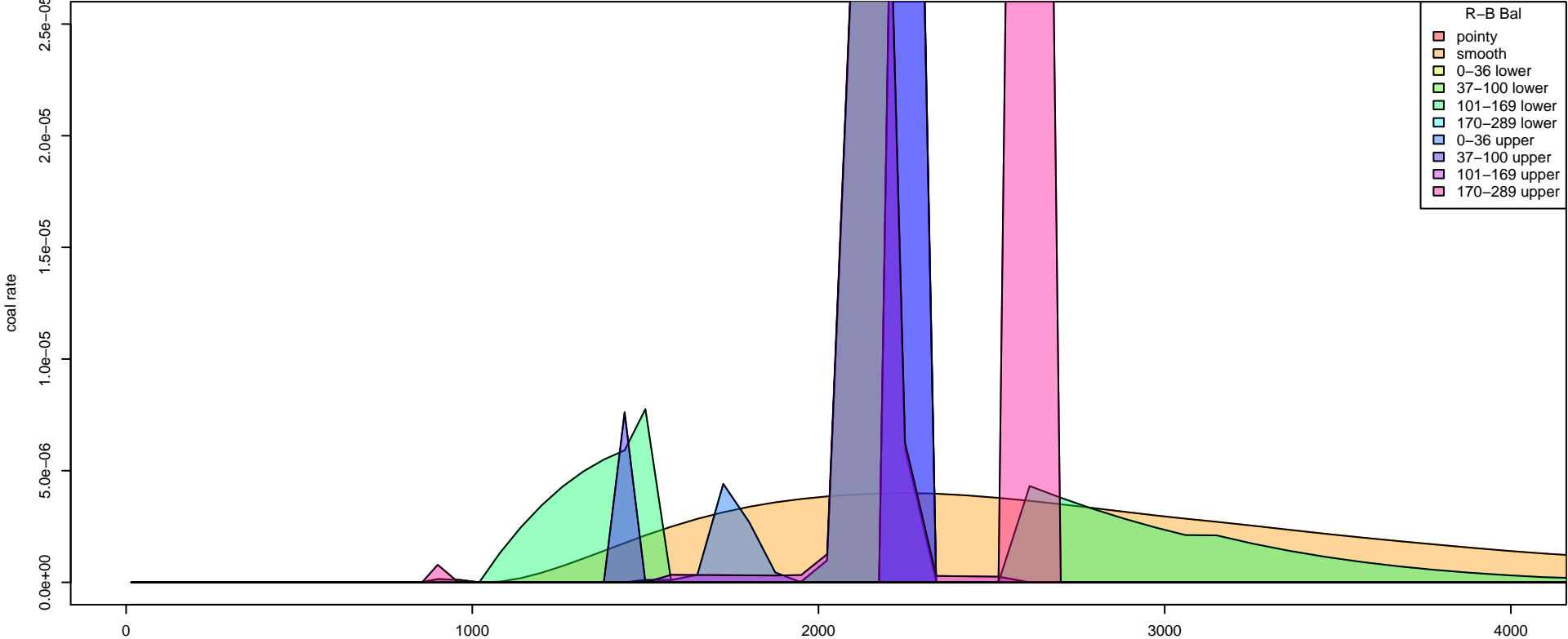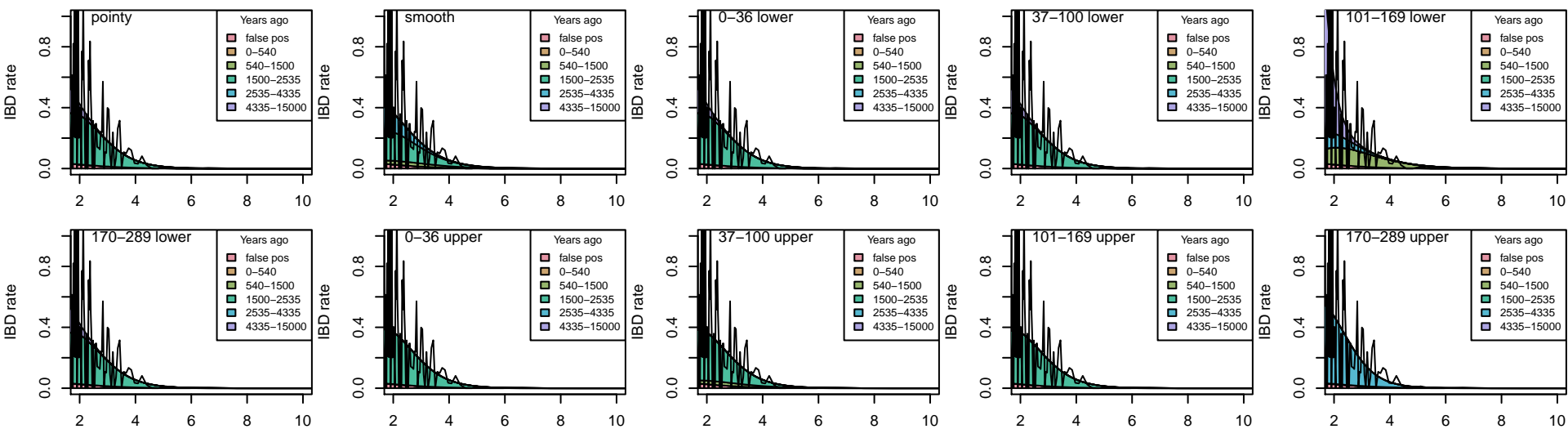



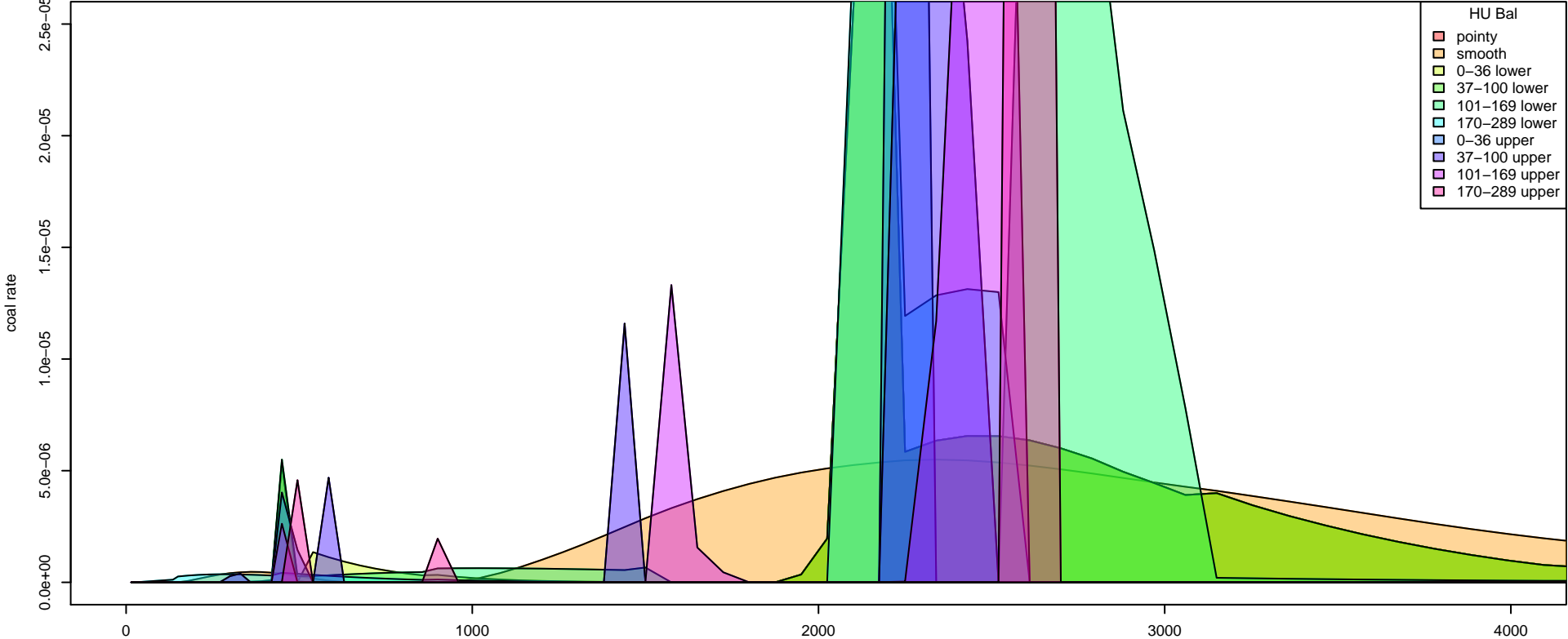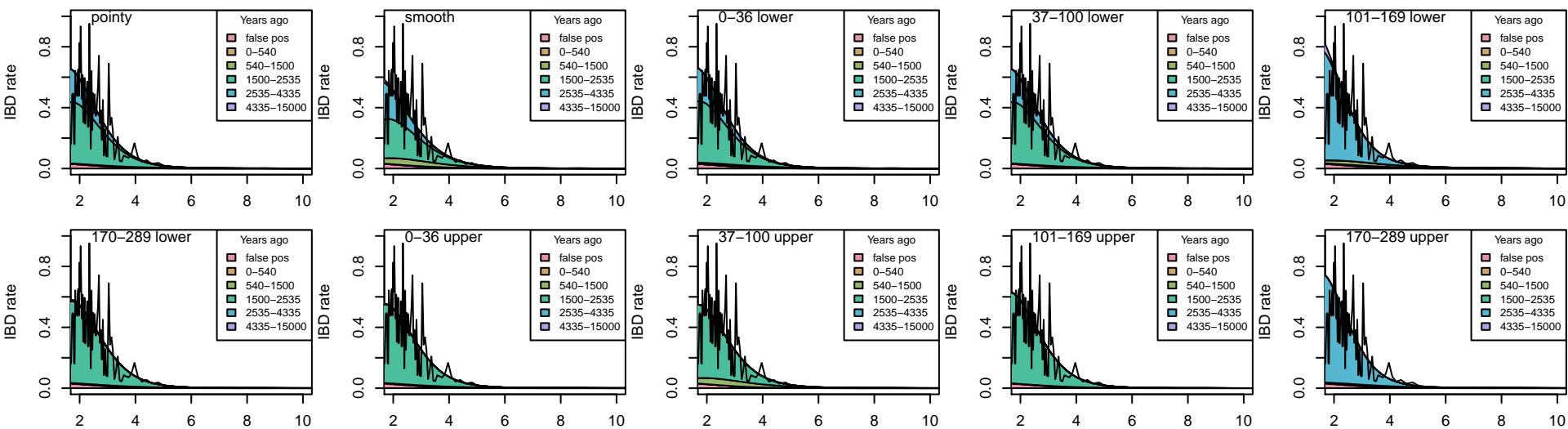

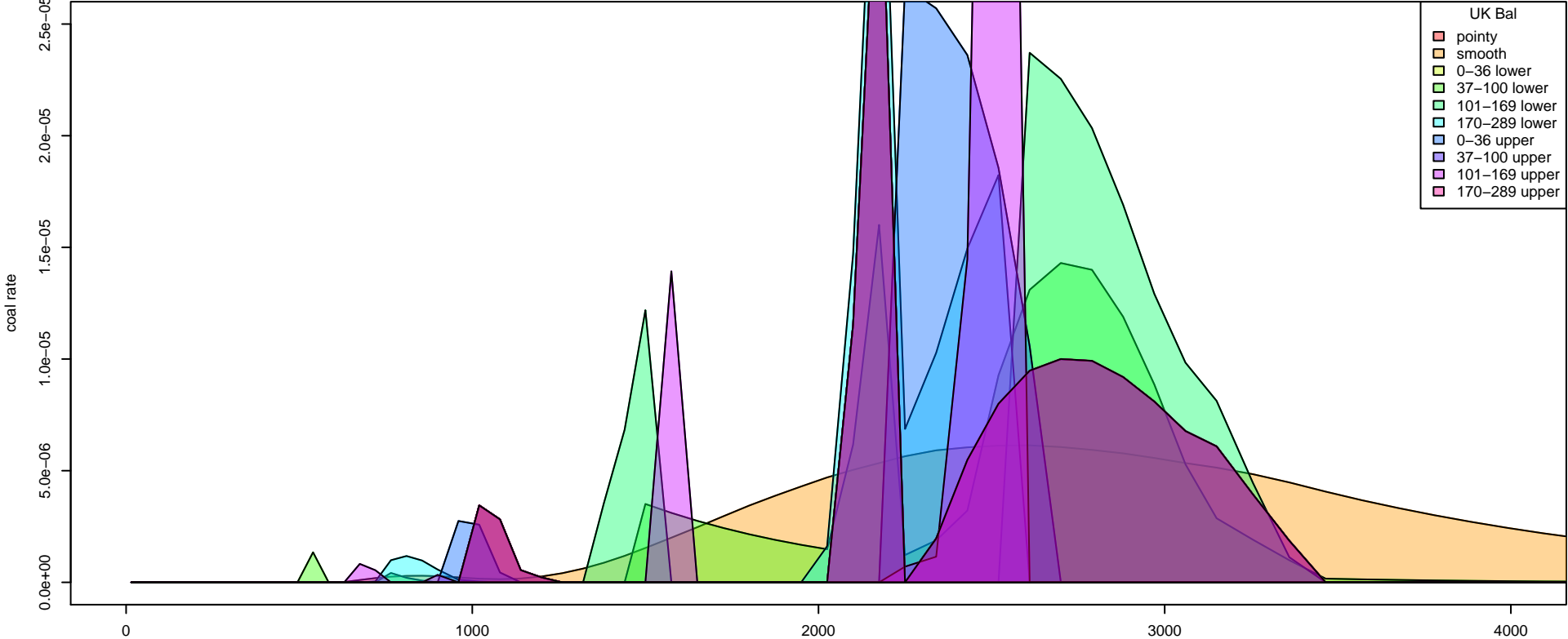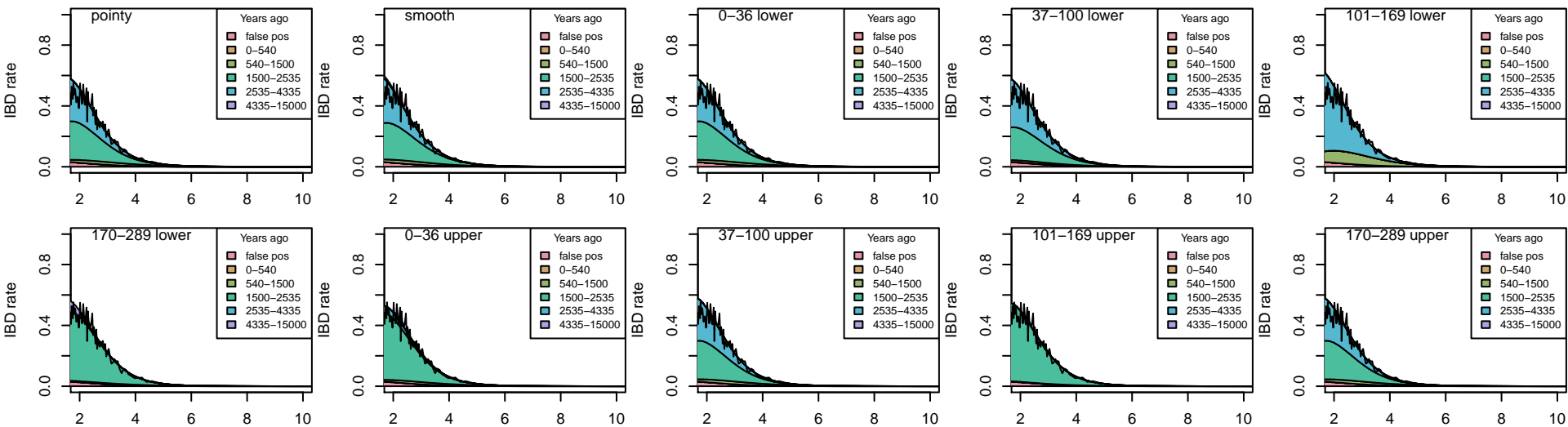

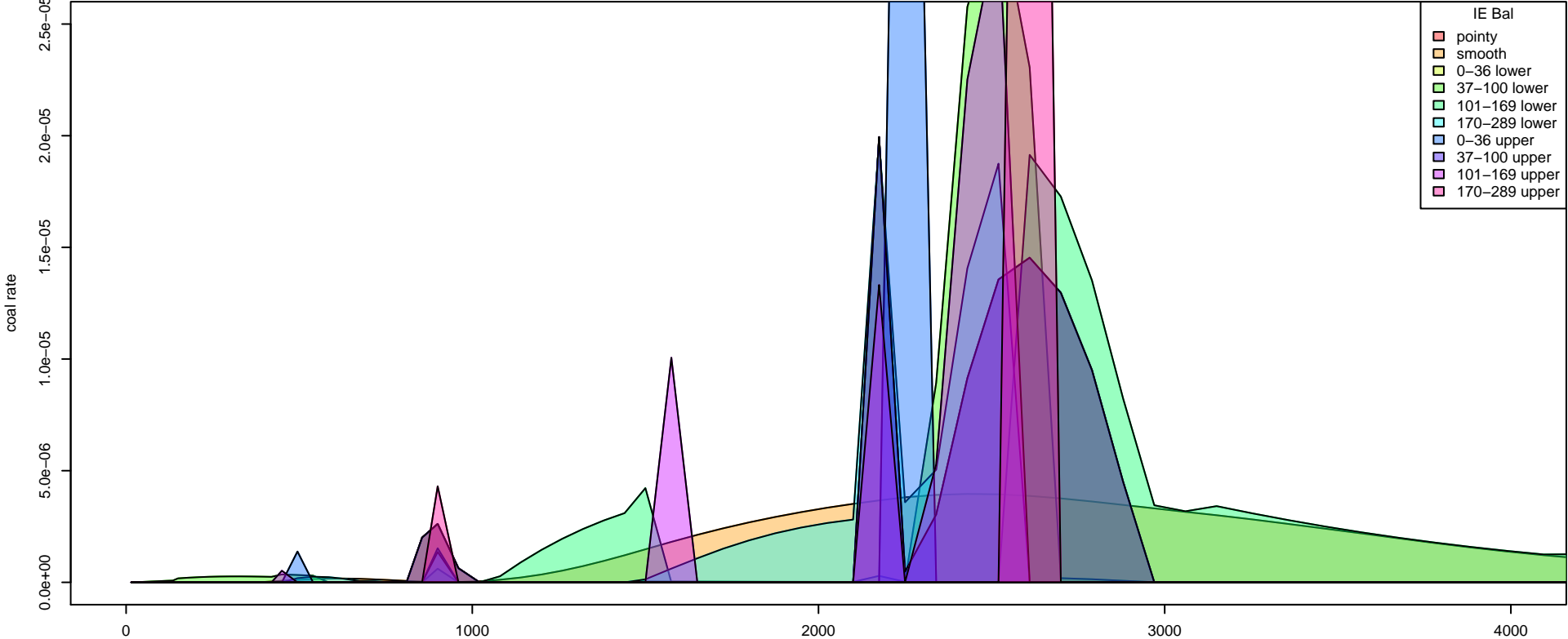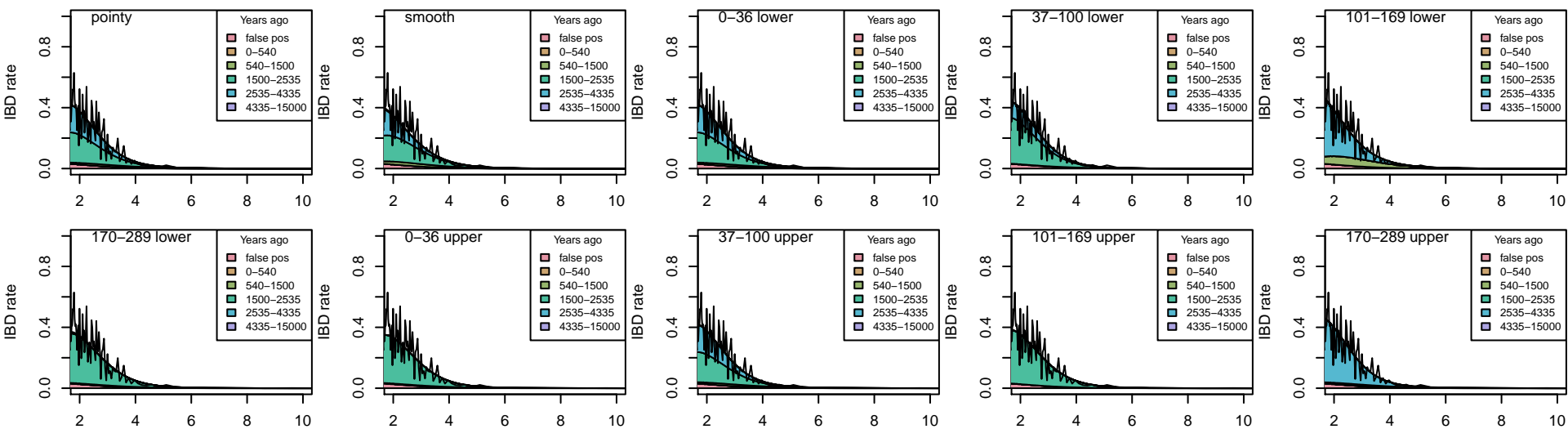

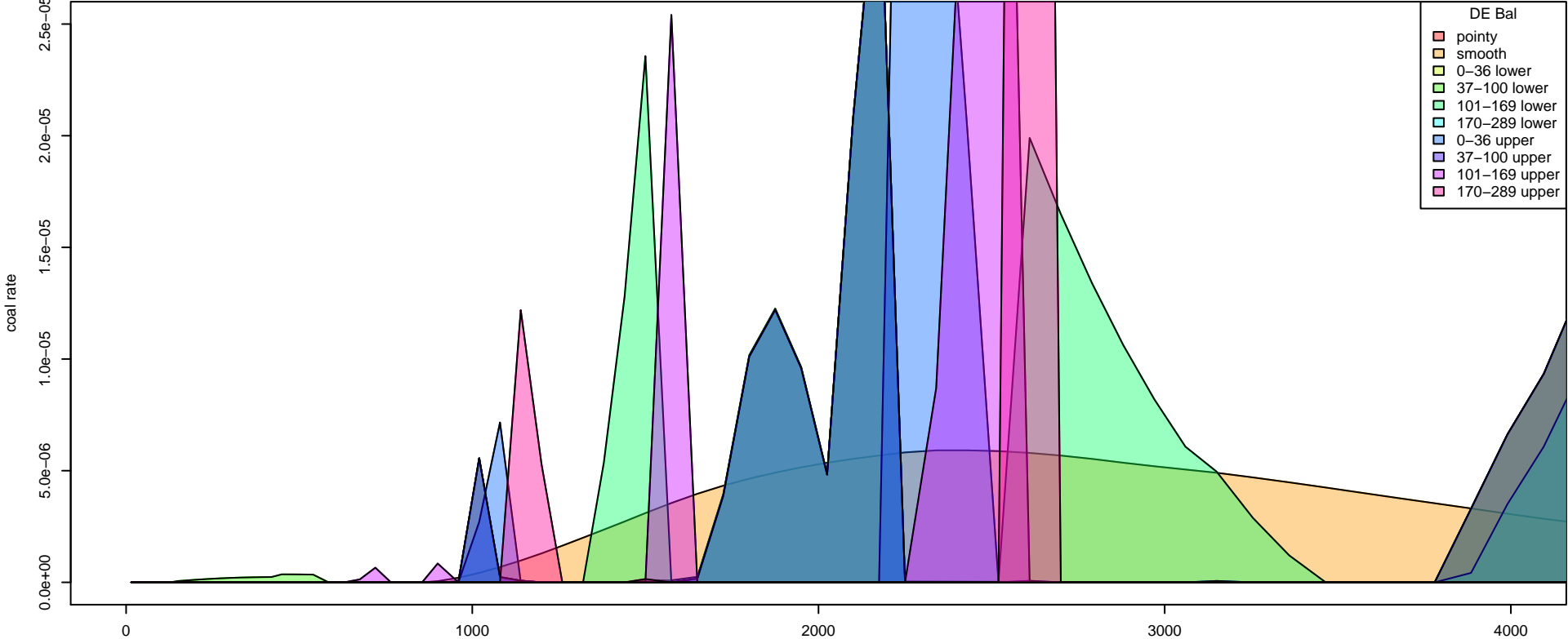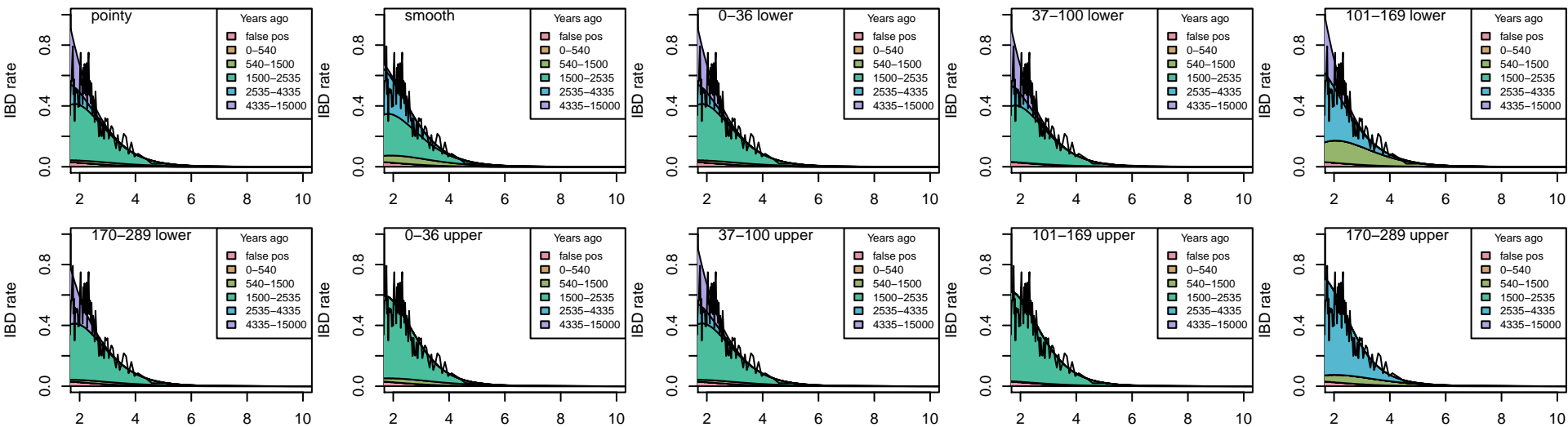

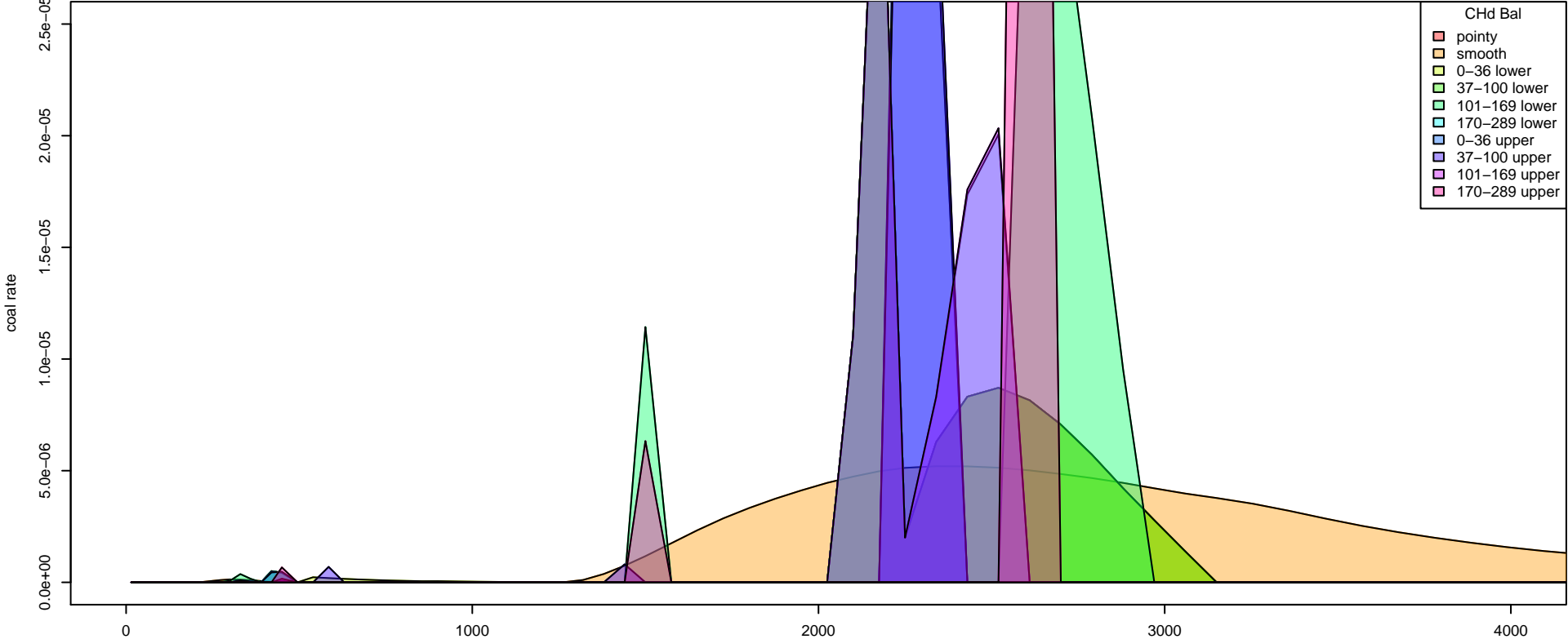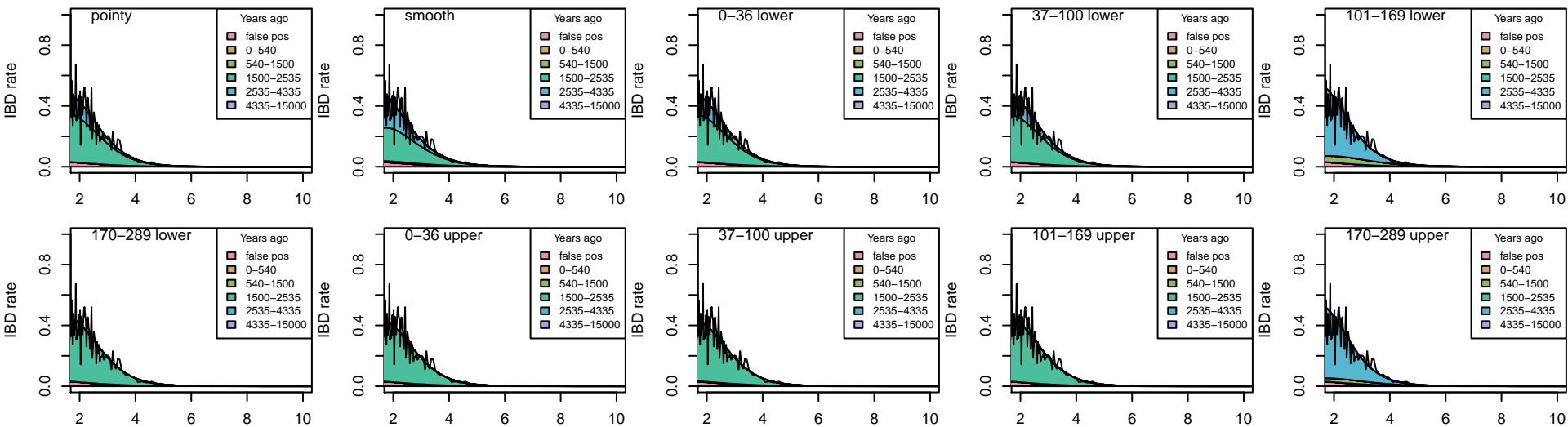

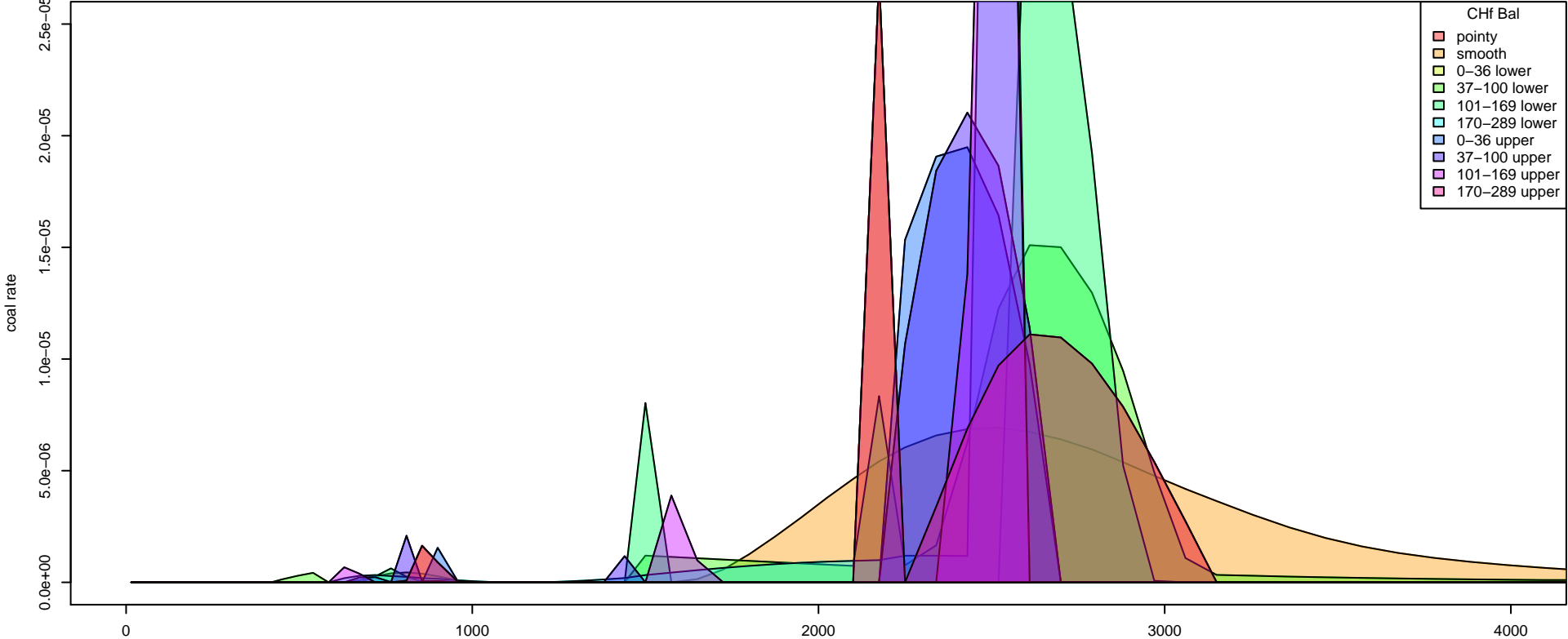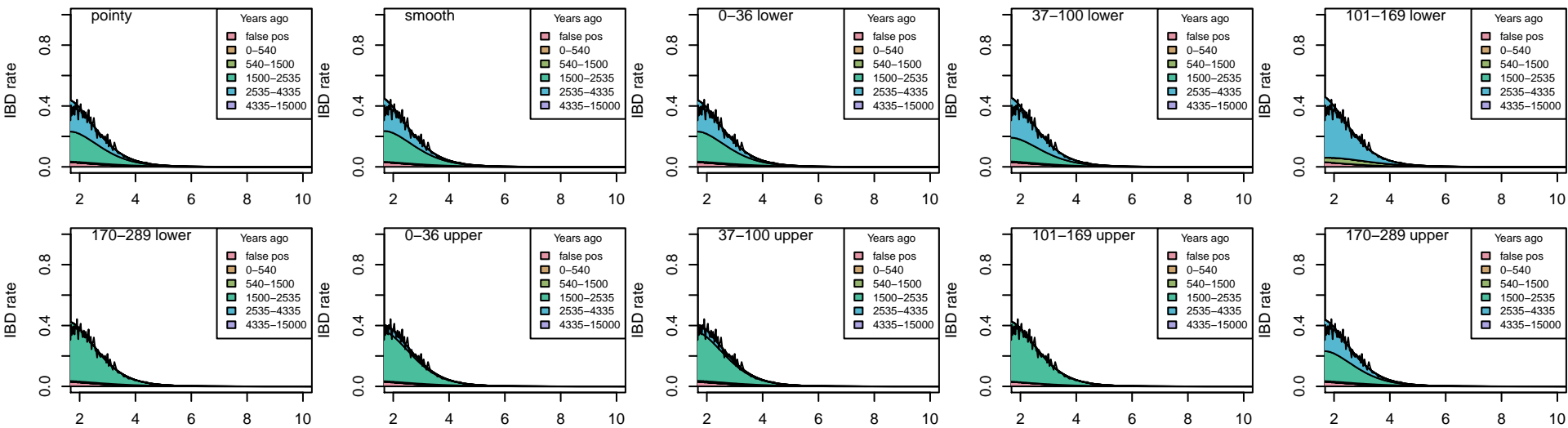

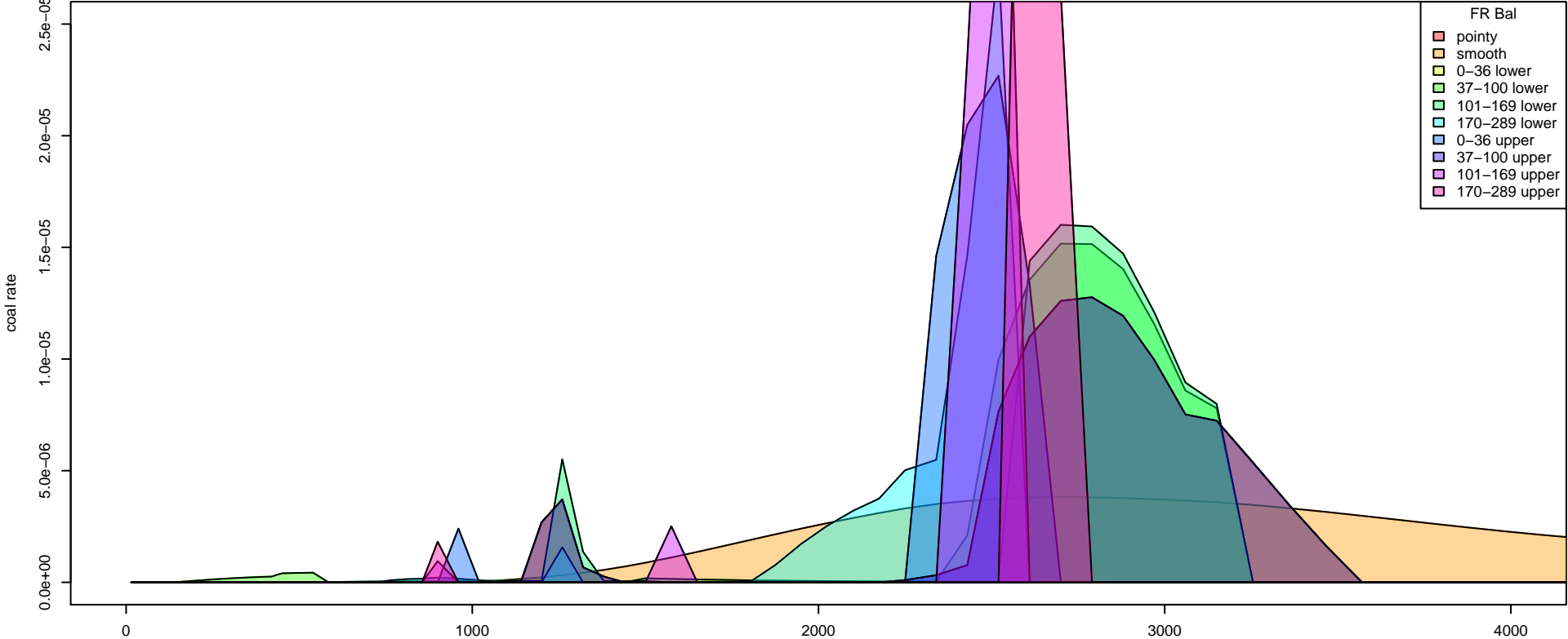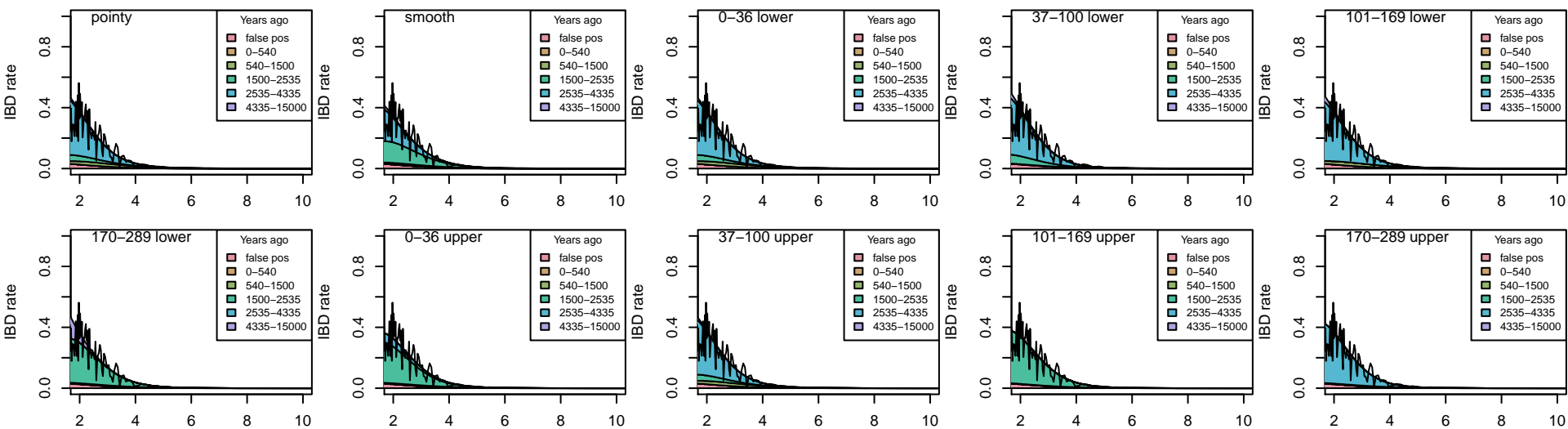

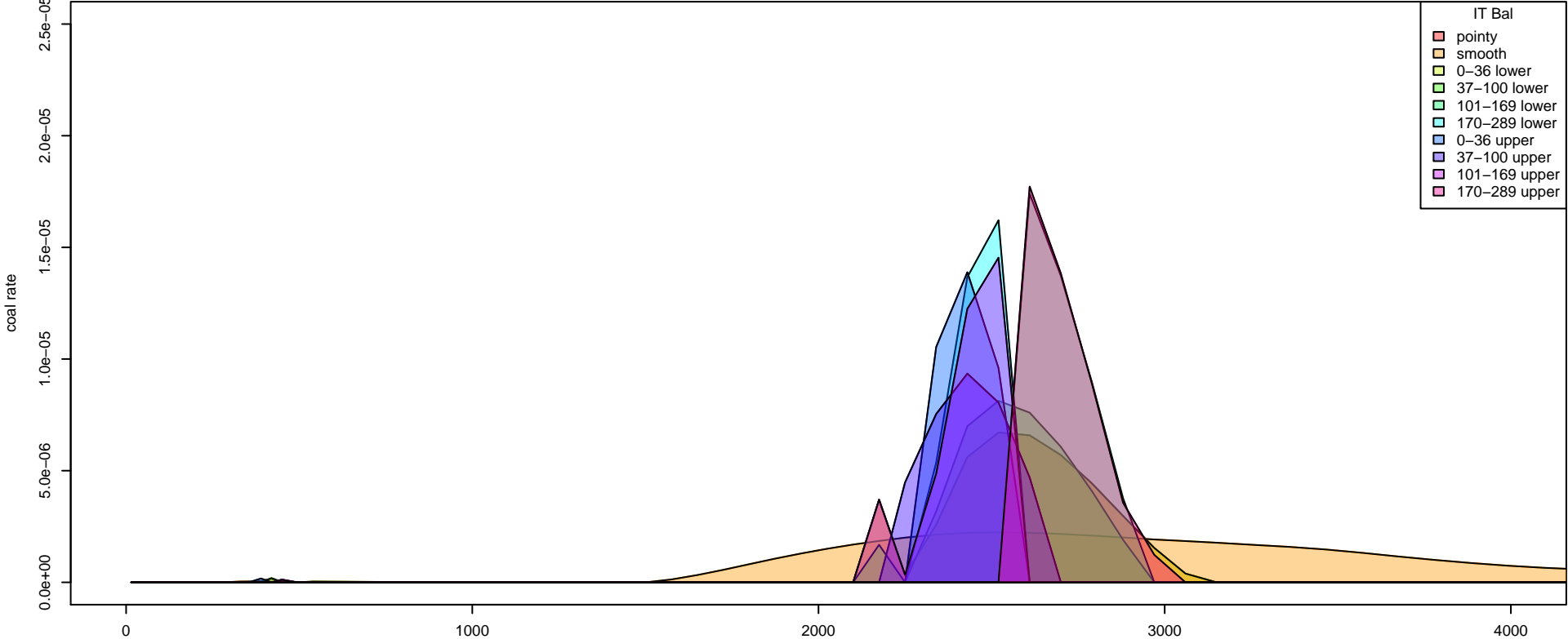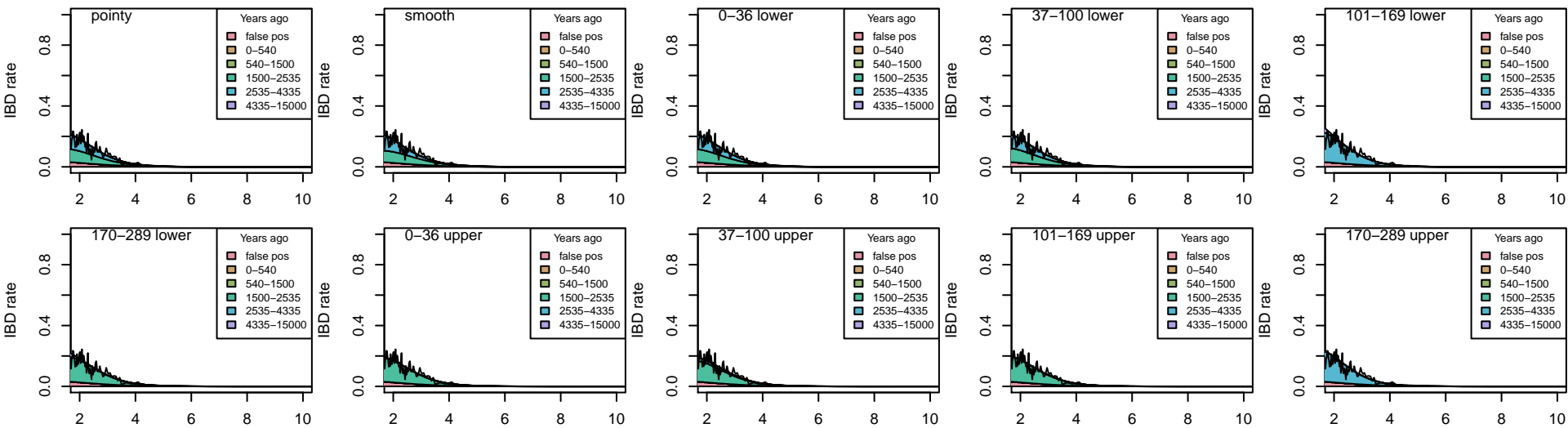

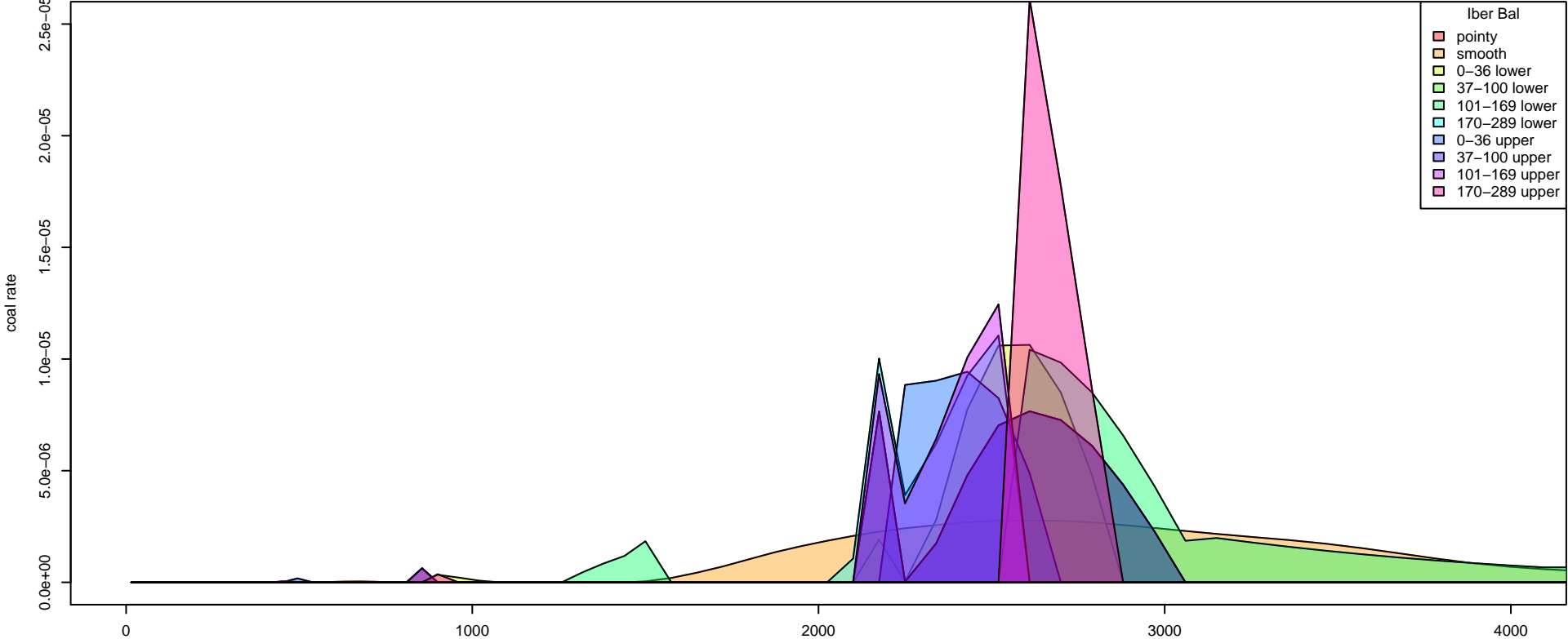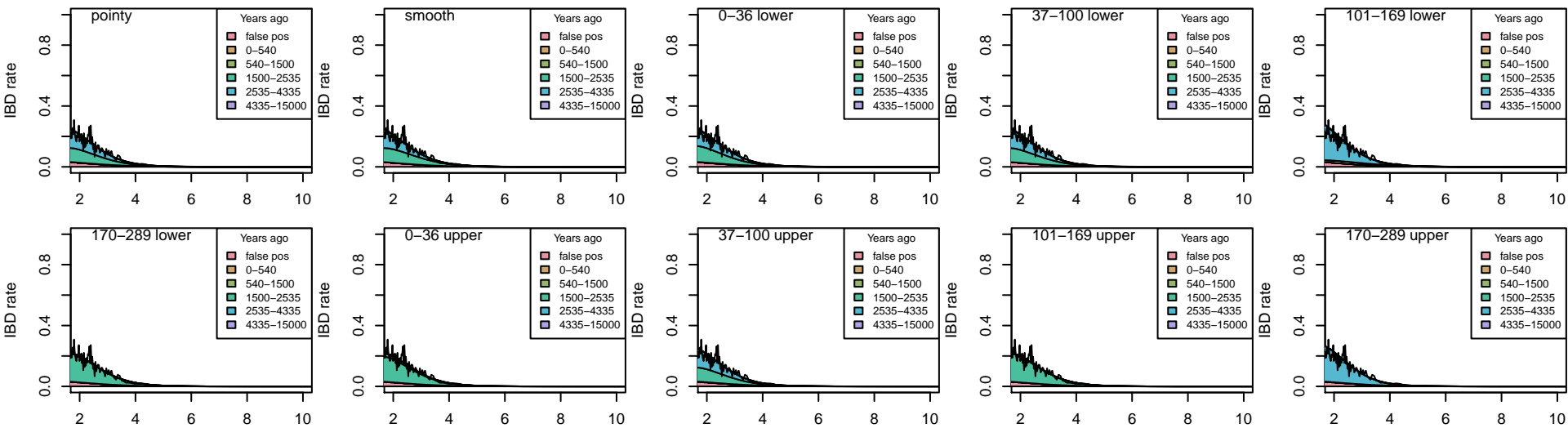

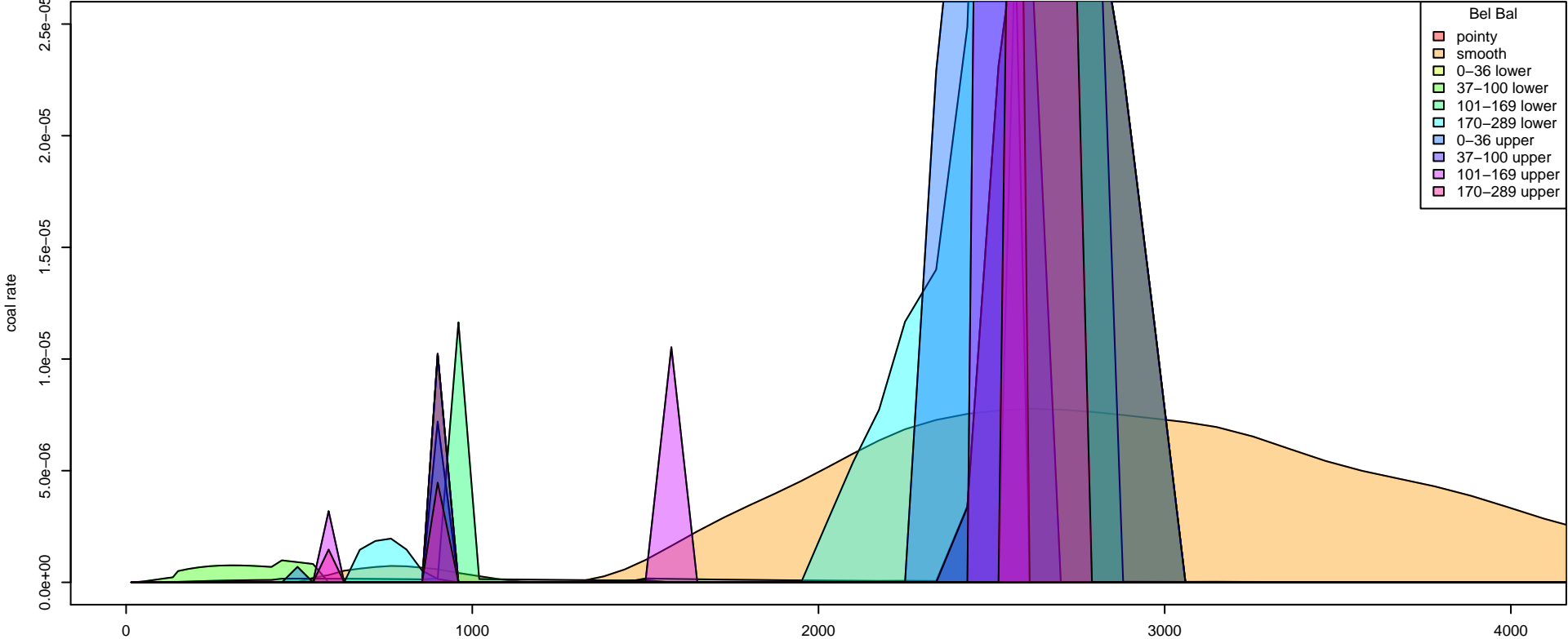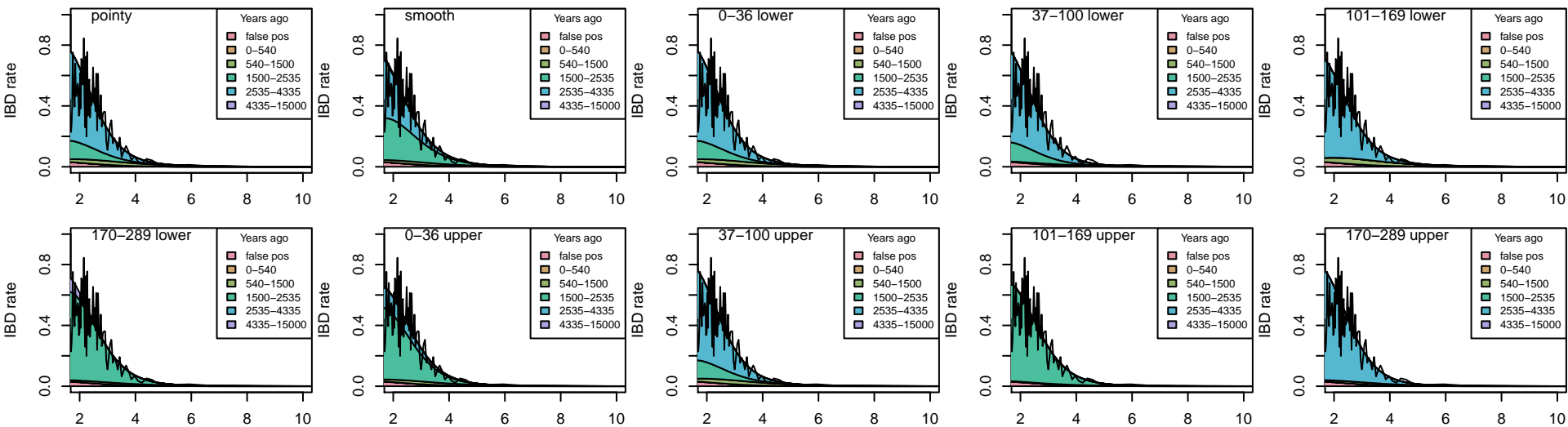

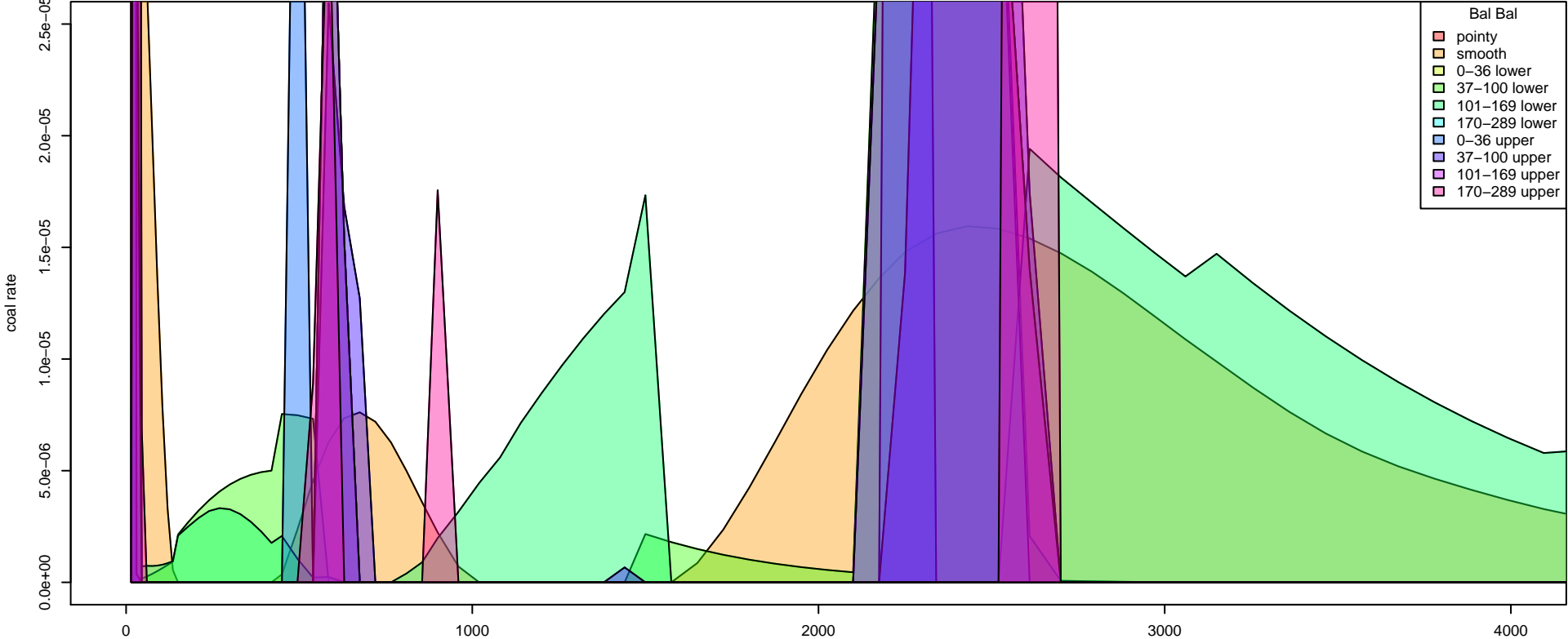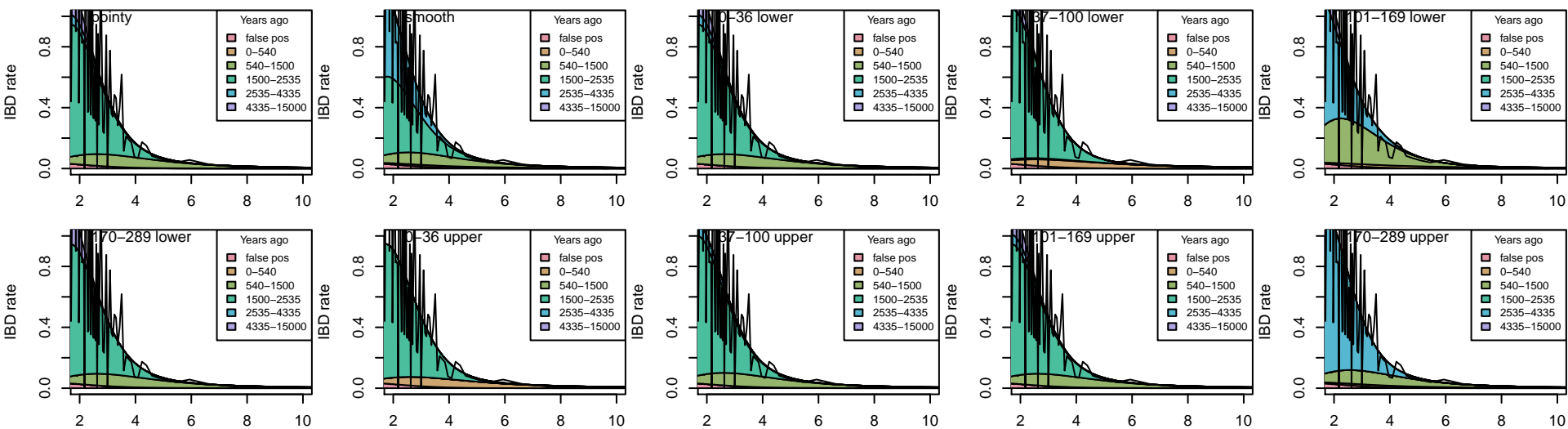

Supplement: Figure S17 — All inversions shown in Figure S16, one per page (225 pages total). There is one page per pair of comparisons used in Figure 5. On each page, there is one large plot, showing 10 distinct consistent histories (numbers of genetic ancestors back through time), and below are 10 histograms of IBD block length, one for each consistent history, showing both the observed distribution and the partitioning of blocks into age categories predicted by that history. The names of the two groupings are shown in the upper right: “pointy” is the unconstrained maximum likelihood solution; “smooth” is the smoothest consistent history; “a–b lower” is the history used to find the lower bound for the time period a–b generations ago in Figure 5; and “a–b upper” is the history used to find the corresponding upper bound. Each of these are described in more detail in the Materials and Methods section. (PDF) [file pbio.1001555.s017.pdf]
