# Supplementary material for: The Geography of Recent Genetic Ancestry across Europe
Source: PLoS Biol. 2013 May 7;11(5):e1001555. doi: 10.1371/journal.pbio.1001555 (PMC3646727; doi:10.1371/journal.pbio.1001555)
Supplement: Figure S7 — Correlations in IBD rates, for six different length windows (omitted length windows are similar). If there are n populations, I(x,y) is the mean number of blocks in the given length range shared by a pair from populations x and y, and , shown is . (PDF) [file pbio.1001555.s007.pdf]

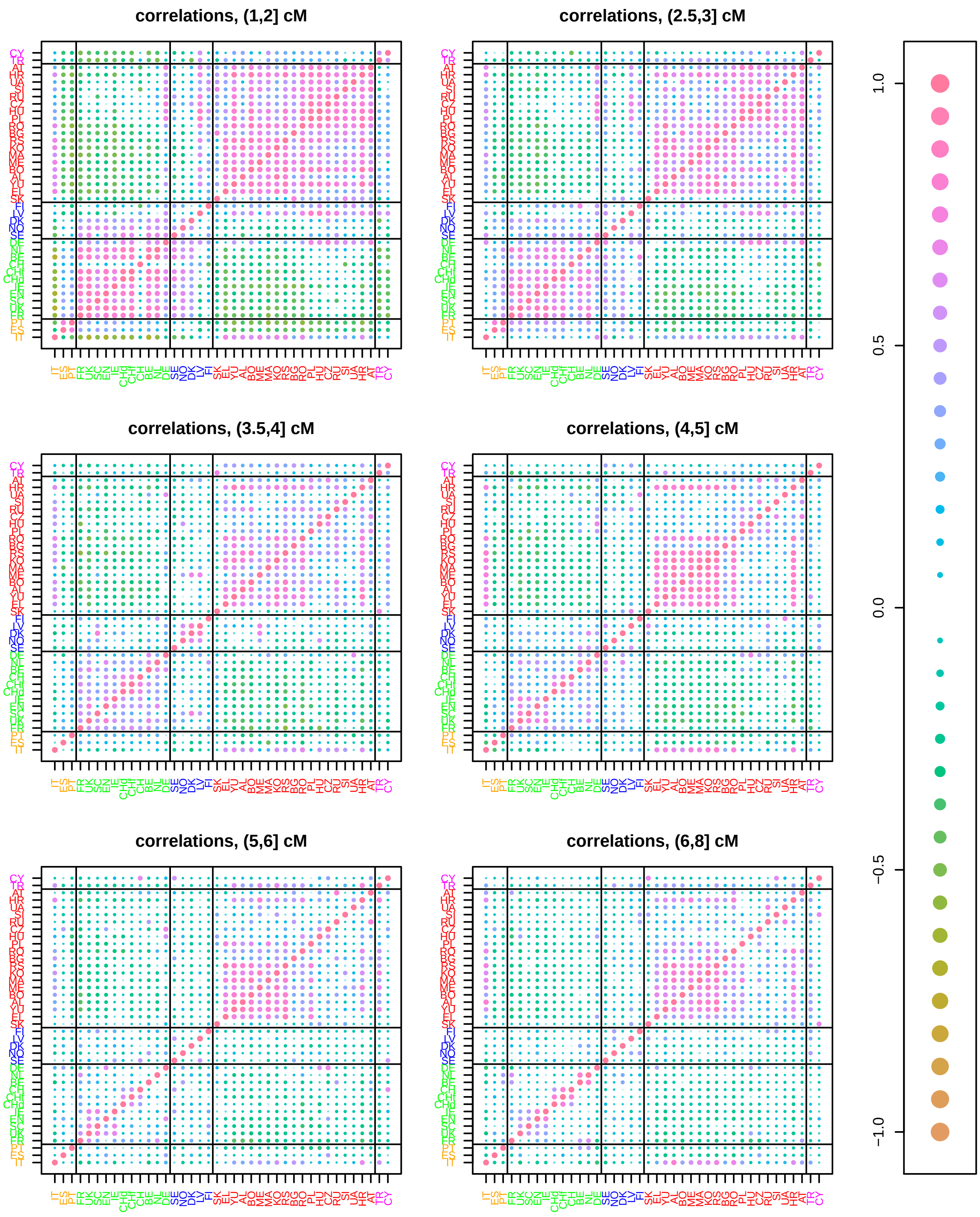
correlations, (1,2] cM
correlations, (2.5,3] cM
correlations, (3.5,4] cM
correlations, (4,5] cM
correlations, (5,6] cM
correlations, (6,8] cM
IT ES PT FR UK SC EN IE CHd CHf CH BE NL DE SE NO DK LV FI SK EL YU AL BO ME MA KO RS BG RO PL HU CZ RU SI UA HR AT TR CY
1.0
0.5
0.0
−0.5
−1.0
