# Supplementary material for: The Geography of Recent Genetic Ancestry across Europe
Source: PLoS Biol. 2013 May 7;11(5):e1001555. doi: 10.1371/journal.pbio.1001555 (PMC3646727; doi:10.1371/journal.pbio.1001555)
Supplement: Text S1 — Description of validation by simulation of the age inference method. (PDF) [file pbio.1001555.s018.pdf]

Supplemental document – simulation results

1 Simulation methods

To test our inference procedure, we implemented a simple whole-genome pedigree simulator, which we briefly describe here. We simulate in reverse time, keeping track of those parts of the pedigree along which genomic material have actually passed; effectively constructing the ancestral recombination graph [Griffiths and Marjoram, 1997] in its entirety. This is computationally taxing, but it is still feasible to generate all relationships between 100 sampled humans (with the actual chromosome numbers and lengths) going back 300 generations in an hour or so on a modern machine with only 8GB of RAM; or on the same machine, 1000 sampled humans going back 150 generations, overnight. (Since memory use and time to process a generation scale linearly with the number of generations and the number of samples, a machine with more RAM could produce longer or larger simulations.) The demographic scenarios are restricted to (arbitrary) discrete population models with changing population sizes and migration rates. The code (python and R) is freely available at `http://github.org/petrelharp`.

The process we want to simulate is as follows: we have n sampled diploid individuals in the present day, and at some time T in the past, wish to know which of the $2n$ sampled haplotypes inherited which portions of genome from the same ancestral haplotypes (i.e. are IBD by time T). We work with diploid individuals, always resolved into maternal and paternal haplotypes, and only work with the autosomes. We treat all chromosomes in a common coordinate system by laying them down end-to-end, with the chromosomal endpoints $g_1 < \cdots < g_c$ playing a special role. The algorithm iterates through previous generations, and works as follows to produce the state at $t+1$ generations ago from the state at t generations ago. Each sampled haplotype can be divided into segments inheriting from distinct ancestral haplotypes from t generations ago. For each of the sampled haplotypes, indexed by $1 \le i \le 2n$, we record the sequence of genomic locations separating these segments as $b(i,t) = (b_1(i,t), \ldots, b_{B(i,t)}(i,t))$, and the identities of the corresponding ancestors from t generations ago as $a(i,t) = (a_1(i,t), \ldots, a_{B(i,t)}(i,t))$, where $B(i,t)$ is the total number of segments the ith sampled haplotype is divided into t generations ago. The first genomic location $b_1(i,t)$ is always 0, and for notational convenience, let $b_{B(i,t)+1}(i,t) = g_c$ (the total genome length). The meaning of $a(i,t)$ is that if two samples i and j match on overlapping segments, i.e. for some k and ℓ, $a_k(i,t) = a_\ell(i,t)$ and $[x,y] = [b_k(i,t), b_{k+1}(i,t)] \cap [b_\ell j, t, b_{\ell+1} j, t]$, then both have inherited the genomic segment $[x,y]$ from the same ancestral haplotype $a_k(i,t)$ alive at time t, and are thus IBD on that segment from sometime in the past t generations.

As parameters, we are given $N_t(u)$, the effective population size in subpopulation u at time t in the past.

To update from t to $t+1$, we need to pick parents for each generation-t ancestor, choose the recombination breakpoints for the meiosis leading to each generation-t haplotype – so, each unique value of $a(\cdot,t)$ has a corresponding (diploid) parent and a set of recombination breakpoints. These steps are performed iteratively along each haplotype, checking if parents and recombination breakpoints have been chosen already for each ancestor, and randomly generating these if not. Recombinations are generated as a Poisson process of unit rate along the genome (so, lengths are in Morgans); to this set each chromosomal endpoint is added independently with probability 1/2 each. To choose a parent, stretches of genome between alternating recombination breakpoints are assigned to the two haplotypes of the parent. For instance, if the recombination breakpoints of a generation-t haplotype labeled a are at $r_1 < \cdots < r_R$, and the maternal and paternal haplotypes of the parent of a are labeled h_m and h_p respectively, any $a_k(i,t)$ with $b_k(i,t) < r_1$ would be changed to h_m, while those with $r_1 \le b_k(i,t) < r_2$ would be changed to h_p, and new segments are added when breakpoints fall inside of an existing segment $(a_k(i,t) < r_1 \le a_{k+1}(i,t))$.

The algorithm is run for a given number of generations, after which an algorithm iterates along all sampled haplotypes in parallel, writing out any pairwise blocks of IBD longer than a given threshold. An IBD block here is a contiguous piece of a single chromosome over which both sampled chromosomes share the same state.

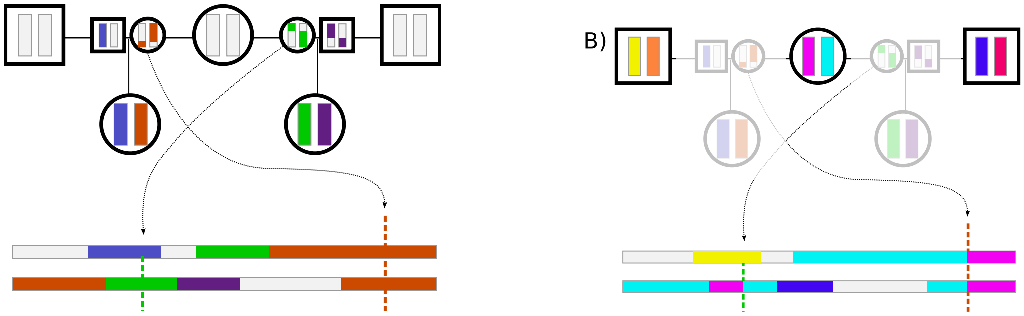

Figure 1: An illustration of the update procedure, moving to the previous generation. At the bottom of **(A)** is the current state in generation t, with haplotype segments colored by which generation-t ancestral haplotype they derive from (e.g. $a(i,t)$), only showing colors for segments inheriting from the depicted two ancestors (all segments in fact have labels). In **(B)** these have been updated to be colored corresponding to which of the generation-$(t+1)$ haplotypes they derive from. The two depicted ancestors are half sibs. The smaller symbols with partially-colored chromosomes depict the location of recombination breakpoints, which are located on the sampled haplotypes by arrows and vertical dotted lines.

2 Test of inference methods

We simulated from three simple demographic scenarios, with parameters chosen to roughly match the mean number of IBD blocks per pair longer than 2cM that we see in the data. The scenarios are as follows:

(A) Constant effective population size 10^5 – average 0.79 IBD blocks longer than 2cM per pair.

(B) Exponential growth, starting from (constant) effective population size 1.5×10^4 prior to 100 generations ago, and approaching 3×10^6 exponentially, as $N_e(t) = 3\times10^6-(3\times10^6-1.5\times10^4)\exp(-0.077(100-t))$ – average 0.51 IBD blocks longer than 2cM per pair.

(C) Exponential growth as in B, except expanding only 50 generations ago, and beginning with an effective population size of 3×10^4 – average 1.11 IBD blocks longer than 2cM per pair.

(D) A more complex scenario: constant size 4×10^4 older than 60 generations ago; growing logistically to 8×10^5 between 60 and 30 generations ago; decreasing logistically to 3×10^4 between 30 and 15 generations ago; constant between 5 and 15 generations ago; and growing again to 4×10^6 until the present – average 1.09 IBD blocks longer than 2cM per pair. (Imagine a population that grows large, has a small group split off gradually, which then grows in the present day; not motivated by any specific history, but chosen to test the methods when the true history is more "bumpy".)

For computational convenience, we simulated only up to 300 generations ago, and retained only blocks longer than 0.5cM (but often restricted analysis to those longer than 2cM); as in the paper we merged any blocks separated by a gap that was shorter than at least one adjacent block and shorter than 5cM. Coalescent rates and block length distributions are shown in figure 2. Even though we have not modeled gap removal, the results still closely match theory, since very few blocks fell so close to each other.

For each scenario, we applied the inversion procedure described in the text to the full, error-free set of blocks as well as to various subsets and modifications of it. The inversion procedure we followed for each was as follows. We chose a discretization for block lengths as described in the text, by starting with the percentiles of the distribution, and refining further so that the largest bin length was 1cM. We then computed the matrix L as described in the text, except with no error distribution or false positive rate, so that if the ith length bin is $[x_i, x_{i+1})$, then $L_{in} = \sum_{g=1}^{22} K_g(n, \min(x_{i+1}, G_g)) - K(n, \min(x_i, G_g))$, with G_g the length of the gth chromosome and $K_g(n,x) = (n(G_g - x) + 1)\exp(-nx)$. For most simulations, we

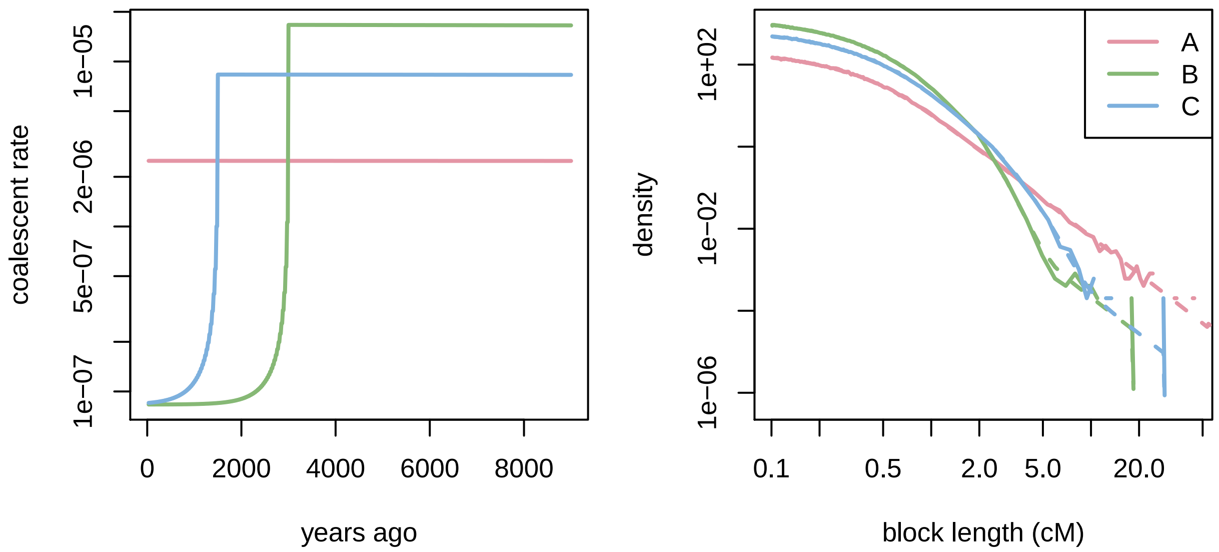

Figure 2: Coalescent rate **(left)** and IBD length spectra **(right)** for the three scenarios. For the length distributions, the value given is observed blocks per pair and per centiMorgan; for each, the dotted line gives the theoretical value predicted from the theoretical coalescent time distribution, and the solid line is the observed distribution.

did not discretize time any further, but allowed n to range from 1 up to 300 generations. We then used constrained optimization as implemented in the `R` package `optim` [L-BFGS-B method, R Development Core Team, 2012] to maximize the penalized likelihoods described in the text, beginning at the solution to the natural approximating least-squares problem [using `quadprog`, Turlach and Weingessel, 2011]. For each case, we show the "maximum likelihood solution" (estimated by adding a small amount of smoothness penalty to ensure numerical uniqueness, allowing the algorithm to converge), and the "smoothest consistent solution" – the largest γ so that the solution has decreased in log-likelihood by no more than 2 units.

In each case, we also show the exact coalescent distribution, as well as the block length spectrum predicted by theory from the true coalescent distribution and each coalescent distribution found by penalized maximum likelihood.

Note that we could have incorporated false positives, missed blocks, or length misestimation into the simulations, and subsequently modified the kernel L to incorporate these rates, but this would only add additional layers of simulation code, and would not make the task of inference more difficult, since we account for these effects analytically. The sensitivity of the methods to *misestimation* of these rates is a concern, but this amounts to misestimation of the kernel L, which we investigate below.

In figure 3 we show the results of the inference procedure applied to the full set of blocks longer than 0.5cM; figure 4 is the same, except using only blocks longer than 2cM, and figure 5 shows the results for scenario D separately. Comparing these, we see that the short blocks 0.5–2cM does not significantly improve the resolution in recent times, but does allow better estimation of coalescent rates longer ago in time. Using blocks longer than 2cM gives us good resolution on the time scale we consider (the past 100 generations), and including those down to 0.5cM does not make the likelihood much less ridged (as expected from theory).

One counter-intuitive result we obtained was that the coalescent history could have a dramatic effect on the estimation of ages of blocks given their lengths. In figure 6 we show the probability distribution of the ages of blocks of various lengths under the four scenarios, i.e. how many generations ago the ancestors lived from whom the samples inherited blocks of that length. These are counter-intuitive because a block inherited from n generations ago has mean length $50/n$ cM, but the age distributions of blocks in practice

show that the converse is not true – blocks x cM long are usually much older than $50/n$ generations. This is computed simply as follows: the mean number of IBD blocks of length x per unit of coalescence from $n/2$ generations ago (from paths of n meioses) is $K(n,x) = \sum_{i=1}^{2} 2n(n(G_i - x) + 1)\exp(-nx)$; so the probability that a block of length x came from $n/2$ generations ago is $K(n,x)/\sum_m K(m,x)$.

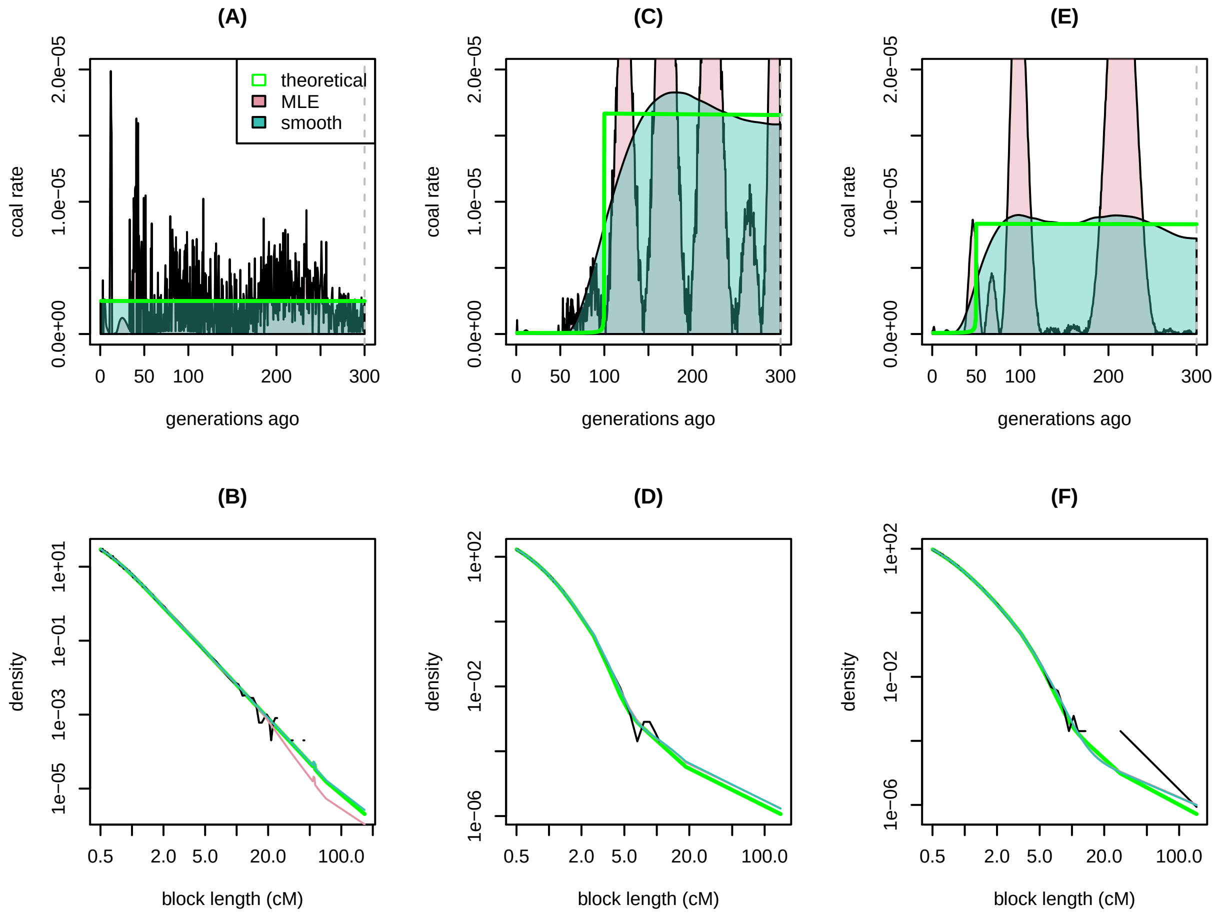

Figure 3: Results of the inference procedure applied to all data (all blocks at least 0.5cM) with 300 generations as the upper limit. Above are true (green) and inferred (shaded) coalescent rates; below are block length distributions (density per pair), observed (black) and predicted by the inferred coalescent distributions in the respective upper panel. **(A–B)**, scenario A; **(C–D)**, scenario B; and **(E–F)**, scenario C. The dangling line at the end of several plots is due to a few rare long blocks and is not a significant deviation from the expectation.

3 Sensitivity analysis

We also used these simulations to evaluate our sensitivity to error. Of particular concern is error arising due to misestimation of the false positive rate – we have seen that false positive rate at short lengths can vary somewhat by population – we estimate as much as 10% around 2cM. To evaluate the effect on inference, we added to the numbers of blocks observed in each category some number of "false positives", but applied the

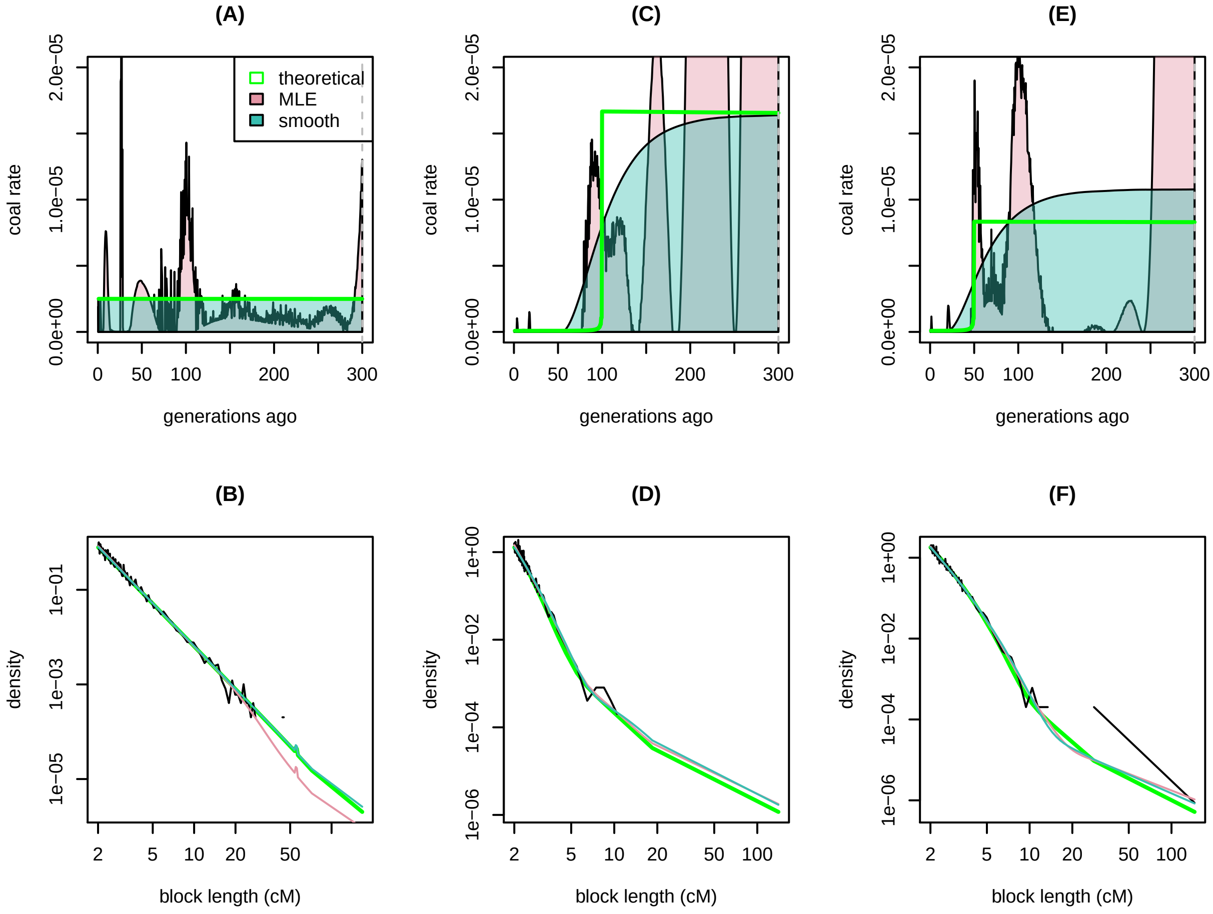

Figure 4: **Longer blocks only** – as in figure 3, except in each case we have only used blocks above at least 2cM.

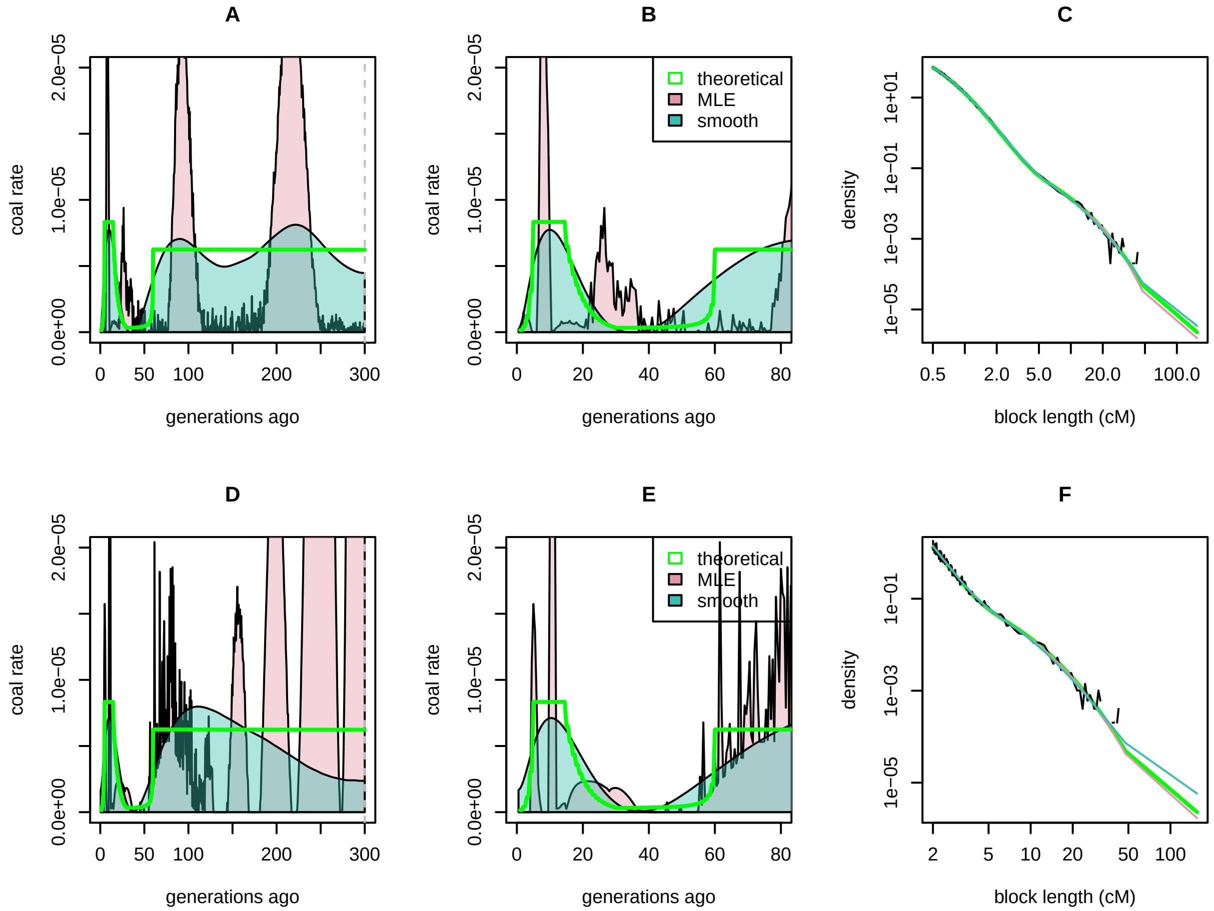

Figure 5: **Scenario D:** Here we show results for the more complex demographic scenario. **(A–C)** use all blocks (down to 0.5cM), with (A) and (C) the same as in figure 3, and (B) the same as (A) except zooming in on more recent times. **(D–F)** is as (A–C), except using only blocks longer than 2cM.

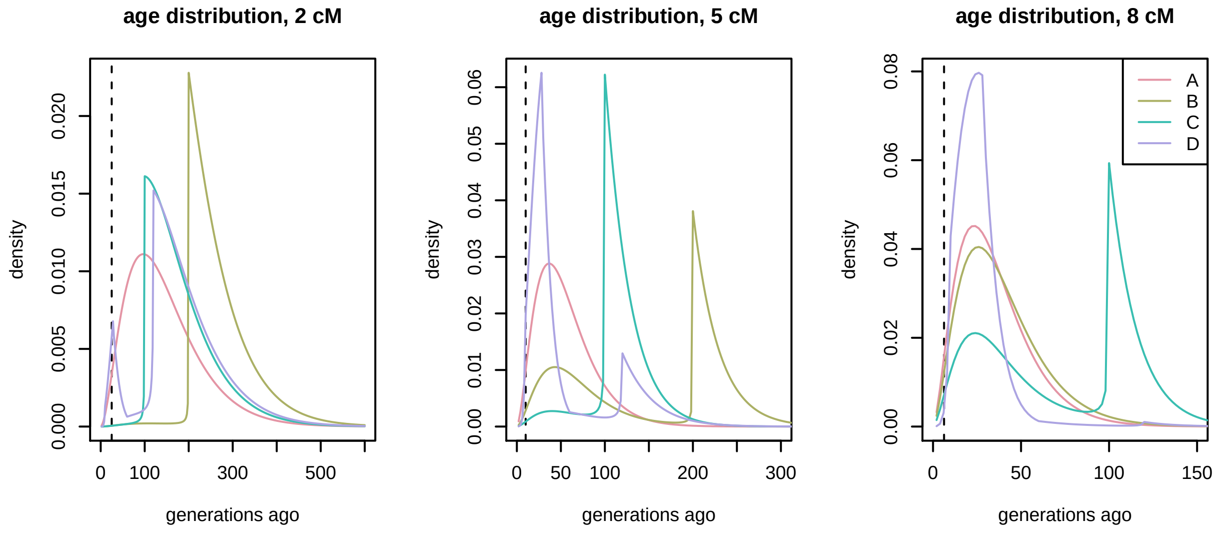

Figure 6: Age distributions of blocks under each of the four scenarios – each curve shows the probability distribution for the age of a 2cM, 5cM, and 8cM block under each of the four scenarios. For the age distribution of blocks x cM long, the vertical dotted line is at $50/x$ generations, the naive expectation for the typical age of such blocks.

inference methods without accounting for these (so we still have $f = 0$). The numbers of false positives added to each length bin are Poisson with mean equal to the theoretical mean predicted for that bin, multiplied by a factor that depends on the length and decreases (so there is an artificial inflation of short blocks). The results for three different false positive rates are shown in figure 7. From these, we see that if IBD rate is only increased by a maximum of 10% – even if the effect extends out to 6 or 8cM – the effect on the inferred coalescent distribution is minor. It is also useful to add a unrealistically high level of unaccounted-for false positives, as it is natural to suspect that an excess of short blocks will only increase the coalescent rate at relatively older time periods. This is indeed the case – doubling the distribution at the short end (about 2–4cM) only affects inferred coalescent rates beyond about 100 generations, because this is when the bulk of the 2–4cM blocks have come from.

References

R. C. Griffiths and P. Marjoram. An ancestral recombination graph. In *Progress in population genetics and human evolution (Minneapolis, MN, 1994)*, volume 87 of *IMA Vol. Math. Appl.*, pages 257–270. Springer, New York, 1997. URL `http://www.math.canterbury.ac.nz/~r.sainudiin/recomb/ima.pdf`.

R Development Core Team. *R: A Language and Environment for Statistical Computing.* R Foundation for Statistical Computing, Vienna, Austria, 2012. URL `http://www.R-project.org/`. ISBN 3-900051-07-0.

B. A. Turlach and A. Weingessel. *quadprog: Functions to solve Quadratic Programming Problems.*, 2011. URL `http://CRAN.R-project.org/package=quadprog`. R package version 1.5-4; S original by Berwin A. Turlach, R port by Andreas Weingessel.

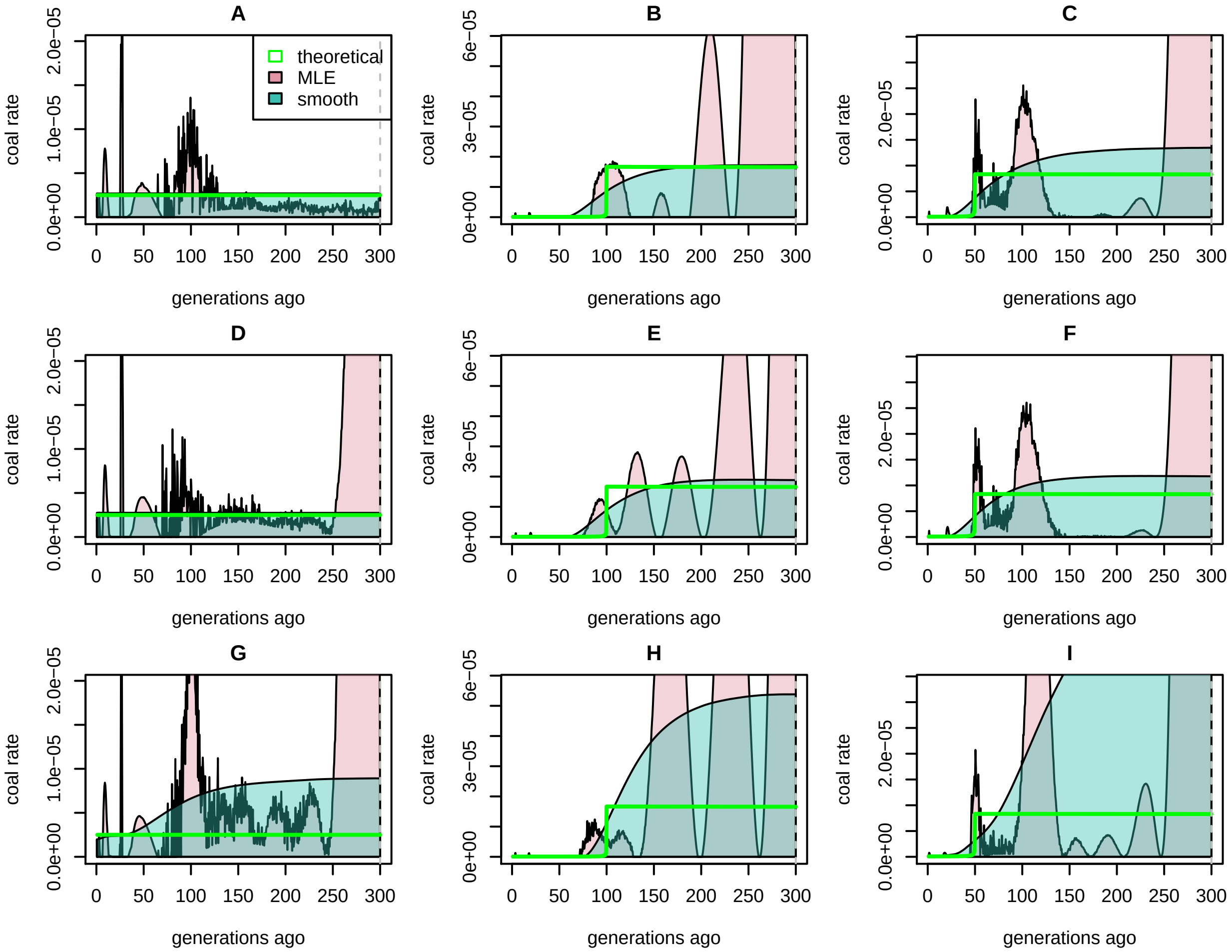

Figure 7: **False positives** – each row shows inference results from the three scenarios with different amounts of spurious false positives added on. If the predicted number of blocks in the bin with length midpoint xcM is $m(x)$, we added a Poisson number of blocks with mean $h(x)m(x)$ to the number observed, with h varying. In the first row **(A–C)**, $h(x) = 0.1 \exp(2 - x)$, in the second row **(D–F)**, $h(x) = 0.1 \exp((2 - x)/4)$, and in the third row **(G–I)**, $h(x) = \exp(2 - x)$. The third scenario is not thought to be realistic, but demonstrates that misestimation at short lengths only affects inference at older times.
