# Supplementary material for: The Geography of Recent Genetic Ancestry across Europe
Source: PLoS Biol. 2013 May 7;11(5):e1001555. doi: 10.1371/journal.pbio.1001555 (PMC3646727; doi:10.1371/journal.pbio.1001555)
Supplement: Table S1 — The composition of our populations. “COUNTRY_SELF” is the reported country of origin; “COUNTRY_GFOLX” is the country of origin of all reported grandparents (individuals with reported grandparents from different countries were removed); “PRIMARY_LANGUAGE” is the reported primary language; “Population” is our population label; and n gives the number of individuals falling in this category. (PDF) [file pbio.1001555.s020.pdf]

COUNTRY_SELF	COUNTRY_GFOLX	PRIMARY_LANGUAGE	Population	n
Albania	Albania	Albanian	Albania	3
Yugoslavia	Serbia	Albanian	Albania	1
Yugoslavia	Yugoslavia	Albanian	Albania	5
Austria		German	Austria	3
Austria	Austria	German	Austria	10
Spain	Austria	German	Austria	1
Belgium	Belgium	Dutch	Belgium	4
Belgium	Belgium	Flemish	Belgium	3
Belgium	Belgium	French	Belgium	28
Germany	Belgium	French	Belgium	1
Switzerland	Belgium	French	Belgium	1
Bosnia	Bosnia	Bosnian	Bosnia	4
Bosnia	Bosnia	Serbian	Bosnia	1
Bosnia	Bosnia	Serbo-Croatian	Bosnia	4
Bulgaria	Bulgaria	Bulgarian	Bulgaria	1
Croatia		Croatian	Croatia	1
Croatia	Croatia	Croatian	Croatia	6
Yugoslavia	Yugoslavia	Croatian	Croatia	1
Croatia	Croatia	Serbo-Croatian	Croatia	1
Cyprus		English	Cyprus	1
Cyprus		Greek	Cyprus	1
Cyprus	Cyprus	Greek	Cyprus	1
Czech Republic	Czech Republic	Czech	Czech Republic	9
Denmark		Danish	Denmark	1
England	England	English	England	18
Turkey	England	English	England	1
United Kingdom	England	English	England	3
Finland	Finland	Finnish	Finland	1
France		French	France	2
France	France	French	France	82
Germany	France	French	France	1
Switzerland	France	French	France	1
Germany			Germany	1
Germany		English	Germany	2
Germany	Germany	French	Germany	1
Germany		German	Germany	1
Germany	Germany	German	Germany	63
Switzerland	Germany	German	Germany	1
Hungary	Germany	Hungarian	Germany	1
Germany	Germany	Polish	Germany	1
Switzerland	Greece	French	Greece	1
Greece	Greece	Greek	Greece	4

(continued on next page)

COUNTRY_SELF	COUNTRY_GFOLX	PRIMARY_LANGUAGE	Population	n
Hungary	Hungary	French	Hungary	1
Hungary	Hungary	Hungarian	Hungary	17
Hungary	Hungary	Russian	Hungary	1
Ireland			Ireland	19
Ireland		English	Ireland	38
England	Ireland	English	Ireland	1
Ireland	Ireland	English	Ireland	1
Ireland	Ireland	French	Ireland	1
Italy			Italy	1
France	Italy	French	Italy	1
Italy	Italy	French	Italy	8
Switzerland	Italy	French	Italy	9
Italy	Italy	German	Italy	1
Italy		Italian	Italy	3
France	Italy	Italian	Italy	1
Italy	Italy	Italian	Italy	170
Romania	Italy	Italian	Italy	1
Sweden	Italy	Italian	Italy	1
Switzerland	Italy	Italian	Italy	17
Kosovo			Kosovo	1
Yugoslavia	Kosovo	Albanian	Kosovo	10
Yugoslavia	Kosovo	Kosovan	Kosovo	2
Yugoslavia	Kosovo	Serbo-Croatian	Kosovo	2
Latvia	Latvia	Latvian	Latvia	1
Macedonia	Macedonia	Macedonian	Macedonia	4
Yugoslavia	Montenegro	Serbian	Montenegro	1
Netherlands	Netherlands	Dutch	Netherlands	15
Holland		English	Netherlands	1
Netherlands	Netherlands	French	Netherlands	1
Norway	Norway	Norwegian	Norway	2
France	Poland	French	Poland	1
Poland		Polish	Poland	4
France	Poland	Polish	Poland	2
Poland	Poland	Polish	Poland	15
France	Portugal	Portuguese	Portugal	1
Portugal	Portugal	Portuguese	Portugal	114
Romania	Romania	Romanian	Romania	14
Romania	Russia	Romanian	Russia	1
Russia	Russia	Russian	Russia	5
Scotland		English	Scotland	3
Scotland	Scotland	English	Scotland	2
Yugoslavia	Serbia	Hungarian	Serbia	1
Serbia	Serbia	Serbian	Serbia	1
Yugoslavia	Serbia	Serbian	Serbia	4
Yugoslavia	Yugoslavia	Serbian	Serbia	2
Croatia	Serbia	Serbo-Croatian	Serbia	1
Yugoslavia	Serbia	Serbo-Croatian	Serbia	2

(continued on next page)

COUNTRY_SELF	COUNTRY_GFOLX	PRIMARY_LANGUAGE	Population	n
Slovakia	Slovakia	Slovakian	Slovakia	1
Italy	Slovenia	Slovene	Slovenia	1
Slovenia	Slovenia	Slovene	Slovenia	1
Spain	Spain	Columbia	Spain	2
Switzerland	Spain	Columbia	Spain	2
Spain	Spain	French	Spain	5
Switzerland	Spain	French	Spain	2
Spain	Spain	Galician	Spain	2
Spain		Spanish	Spain	4
Spain	Spain	Spanish	Spain	106
Switzerland	Spain	Spanish	Spain	7
Sweden			Sweden	1
Sweden	Sweden	Swedish	Sweden	9
Switzerland		French	Swiss French	1
Belgium	Switzerland	French	Swiss French	1
Czech Republic	Switzerland	French	Swiss French	1
France	Switzerland	French	Swiss French	7
Poland	Switzerland	French	Swiss French	1
Portugal	Switzerland	French	Swiss French	1
Spain	Switzerland	French	Swiss French	1
Switzerland	Switzerland	French	Swiss French	826
Switzerland	Switzerland	German	Swiss German	103
Italy	Switzerland	Italian	Switzerland	2
Switzerland	Switzerland	Italian	Switzerland	12
Switzerland	Switzerland	Patois	Switzerland	1
Switzerland	Switzerland	Romansch	Switzerland	1
Spain	Switzerland	Spanish	Switzerland	1
Turkey	Turkey	Turkish	Turkey	4
Ukraine	Ukraine	Ukranian	Ukraine	1
United Kingdom			United Kingdom	87
United Kingdom		English	United Kingdom	270
United Kingdom	United Kingdom	English	United Kingdom	1
Yugoslavia			Yugoslavia	1
Yugoslavia	Yugoslavia	French	Yugoslavia	1
Yugoslavia	Yugoslavia	Romanian	Yugoslavia	1
Yugoslavia	Yugoslavia	Serbo-Croatian	Yugoslavia	3
Yugoslavia	Yugoslavia	Yugoslavian	Yugoslavia	4

Table S1: The composition of our populations. "COUNTRY_SELF" is the reported country of origin; "COUNTRY_GFOLX" is the country of origin of all reported grandparents (individuals with reported grandparents from different countries were removed); "PRIMARY_LANGUAGE" is the reported primary language; "Population" is our population label; and n gives the number of individuals falling in this category.
