# Supplementary material for: The Geography of Recent Genetic Ancestry across Europe
Source: PLoS Biol. 2013 May 7;11(5):e1001555. doi: 10.1371/journal.pbio.1001555 (PMC3646727; doi:10.1371/journal.pbio.1001555)
Supplement: Table S2 — Estimated coefficients describing the effect of changing population sample size, as described in the text (Materials and Methods, “Differential Sample Sizes”). Stars denote statistical significance: “*” corresponds to p<.05 and “**” corresponds to p<.01. The coefficients are from a binomial GLM with a logit link function, applied to the number of IBD segments detected in the same set of individuals run with and without an additional 812 individuals. For instance, the top three entries in the left column tell us that if F is the number of segments greater than 1 cM found between Albanian and Austrian individuals in analysis with the full dataset, and S is the corresponding number in the analysis with only the subset, that the model predicts that (plus binomial sampling noise). Note that coefficients producing effect sizes larger than 4% (e.g., Austria for 0–1 cM) all correspond to populations with small sample sizes, and are not significant. (PDF) [file pbio.1001555.s021.pdf]

	0-1 cM	1-2 cM	2-4 cM	4-10 cM
(Intercept)	0.08313	0.1436 **	0.07034	-0.0193
Albania	-0.00097	0.0063	-0.04232	-0.0323
Austria	0.36424	0.0365	0.05874	-0.1399
Belgium	-0.02009	0.0863	0.02874	0.0374
Bosnia	-0.13914	-0.0327	0.02277	0.1032
Bulgaria	-0.14857	0.0467	-0.21836	0.2427
Croatia	0.08645	-0.0223	0.02045	-0.0371
Cyprus	-0.15556	-0.0775	0.08306	0.2158
Czech Republic	-0.14301	0.1013	0.02656	0.1607
Denmark	0.66101	0.1265	0.03675	0.3095
England	0.12131	0.0854	0.03784	0.0199
Finland	0.24617	0.1491	-0.09775	0.9455
France	0.05049	0.0533	0.09670 **	0.1655
Germany	0.06837	0.0454	0.08617 **	0.1288
Greece	0.06752	-0.2266	0.00086	0.3340
Hungary	0.04491	-0.0070	0.11499 *	0.1179
Ireland	0.07873	0.0676	0.04292	0.0466
Italy	0.02728	0.0218	0.06694 *	0.1607
Kosovo	0.17256	0.0384	-0.02022	0.0109
Latvia	0.76499	0.1805	-0.07177	-0.0787
Macedonia	-0.12898	0.1414	-0.04692	-0.0093
Montenegro	0.50084	-0.0845	-0.01400	0.2746
Netherlands	0.10448	0.0842	0.11875 *	0.0423
Norway	-0.46112	-0.0176	0.07972	0.4042
Poland	0.21095	0.0021	0.05353	0.1314
Portugal	0.03987	-0.0019	0.06771 *	0.0664
Romania	-0.24251	0.0545	-0.05095	-0.0845
Russia	0.12047	-0.0094	0.00484	0.1568
Scotland	0.25426	0.0183	0.03205	-0.1172
Serbia	0.06965	0.0384	-0.04688	0.1397
Slovakia	-0.00988	-0.0458	0.21389	-0.3070
Slovenia	-0.19993	0.1456	-0.00855	0.4673
Spain	0.02470	0.0268	0.08928 **	0.1084
Sweden	0.02043	-0.0076	0.20759 **	0.2666
Swiss French	0.11099	0.1394 **	0.07782 *	0.0525
Swiss German	0.14555	0.0943 **	0.08075 **	0.0564
Switzerland	0.13339	0.0102	0.04665	0.1135
Turkey	-0.01107	0.0762	0.13784	-0.1003
Ukraine	-0.33535	-0.0543	0.03820	-1.2068
United Kingdom	0.10379	0.0868 **	0.11605 **	0.1309
Yugoslavia	-0.10508	0.0436	-0.00218	0.0018

Table S2: Estimated coefficients describing the effect of changing population sample size, as described in the text (methods, "Differential sample sizes"). Stars denote statistical significance: "*" corresponds to $p < .05$ and "**" corresponds to $p < .01$. The coefficients are from a binomial GLM with a logit link function, applied to the number of IBD segments detected in the same set of individuals run with and without an additional 812 individuals. For instance, the top three entries in the left column tell us that if F is the number of segments less than 1cM found between Albanian and Austrian individuals in analysis with the full dataset, and S is the corresponding number in the analysis with only the subset, that the model predicts that $S/(S+F) \approx (1+\exp(-0.08313+0.00097-0.36424))^{-1} = 0.61$ (plus binomial sampling noise). Note that coefficients producing effect sizes larger than 4% (e.g. Austria for 0–1cM) all correspond to populations with small sample sizes, and are not significant.
